# Supplementary material for: Household cooking fuel estimates at global and country level for 1990 to 2030
Source: Nat Commun. 2021 Oct 4;12:5793. doi: 10.1038/s41467-021-26036-x (PMC8490351; doi:10.1038/s41467-021-26036-x)
Supplement: Supplementary file 1 — Supplementary Information [file 41467_2021_26036_MOESM1_ESM.pdf]

Supplementary Information

Household cooking fuel estimates at global and country level for 1990 to 2030

Contents

- Supplementary Table 1
- Supplementary Table 2
- **A** LMIC Plots
- **B** SDG Region and Global Plots
- **C** WHO Region Plots

Supplementary Table 1: ***Sources of survey data contained within the WHO Household Energy Database as of March 2020.***

Data source	Entity conducting survey	Typical survey question	Number of countries		Number of surveys	
			In database	Used in model	In database	Used in model
**National Living Standards or Other Surveys**		Major fuel used for cooking	105	97	584	478
**Demographic and Health Survey (DHS)**	Funded by USAID; implemented by ICF International	What type of fuel does your household mainly use for cooking?	80	79	233	224
**Census**	National statistical agencies	What is the main source of cooking fuel in your household?	108	96	245	185
**Multi-indicator cluster survey (MICS)**	UNICEF	What type of fuel does your household mainly use for cooking?	82	79	147	142
**World Health Survey**	WHO	What type of fuel does your household mainly use for cooking?	49	44	49	44
**Living Standard Measurement Survey, income expenditure survey, or other national surveys**	National statistical agencies, supported by the World Bank	Which is the main source of energy for cooking?	45	35	89	58
**Survey on global AGEING (SAGE)**	WHO	What is your main source of energy for cooking?	6	5	6	5
**Total**					1353	1136

Supplementary Table 2: ***Survey data exclusions as of March 2020. As some surveys data points fail multiple criteria, the total number of surveys excluded is less than the total of the values below. Please note that survey exclusion criteria are subject to annual review during updates to Sustainable Development Goal indicator 7.1.2. Current exclusion criteria are detailed in the latest version of Tracking SDG7: The Energy Progress Report. In the 2021 edition, these details are reported in the Methodology section of Chapter 2.***

Reason for exclusion from modelling	Number of data rows removed	Number of unique surveys affected	Number of countries with affected surveys
**Unable to assign global burden of disease region**	35	19	9
**Improper entry for urban/rural/overall**	4	3	3
**Values for specific fuels are all equal to 0% or NA**	125	77	52
**Total % of respondents missing, reporting no cooking, or mainly using ‘other’ fuels exceeds 15%**	70	35	16
**Identified by WHO as unsuitable for modelling, e.g. fuel categories are not mutually exclusive**	157	63	43

A

LMIC Plots

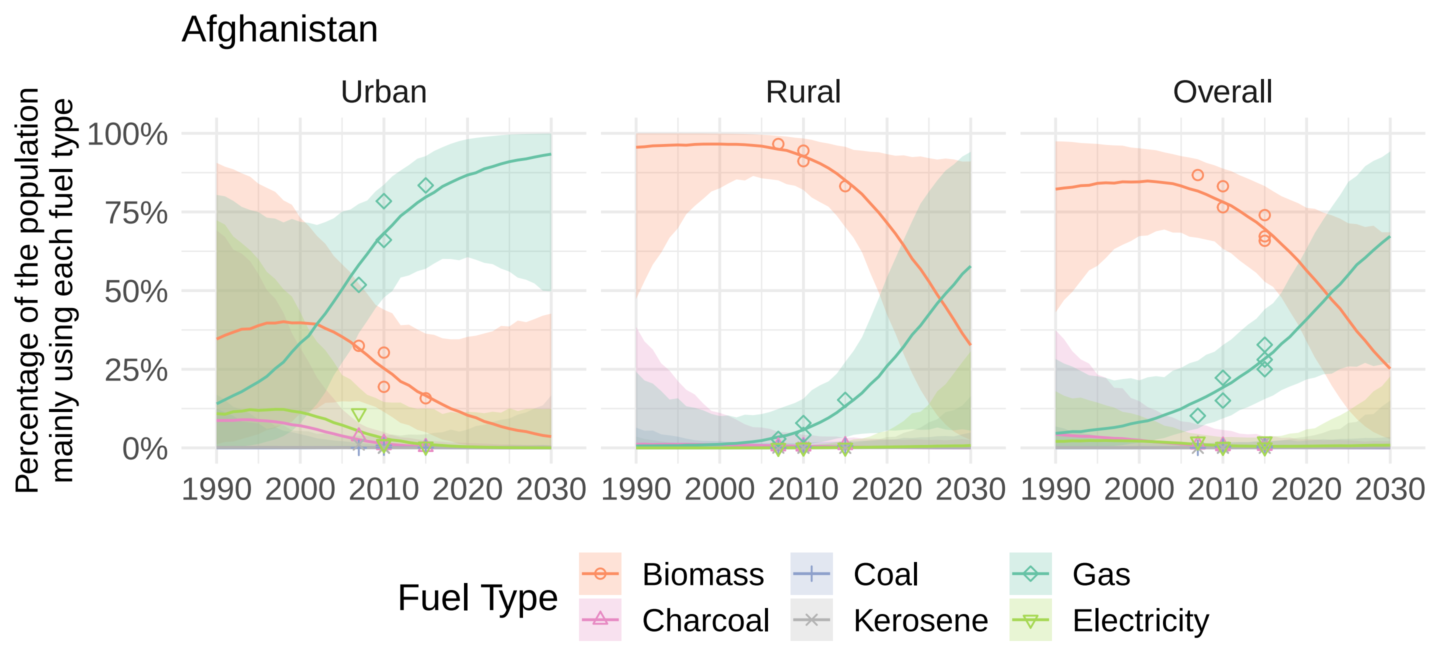

Afghanistan
Urban
Rural
Overall
Percentage of the population mainly using each fuel type
100%
75%
50%
25%
0%
1990
2000
2010
2020
2030
Fuel Type
Biomass
Charcoal
Coal
Kerosene
Gas
Electricity

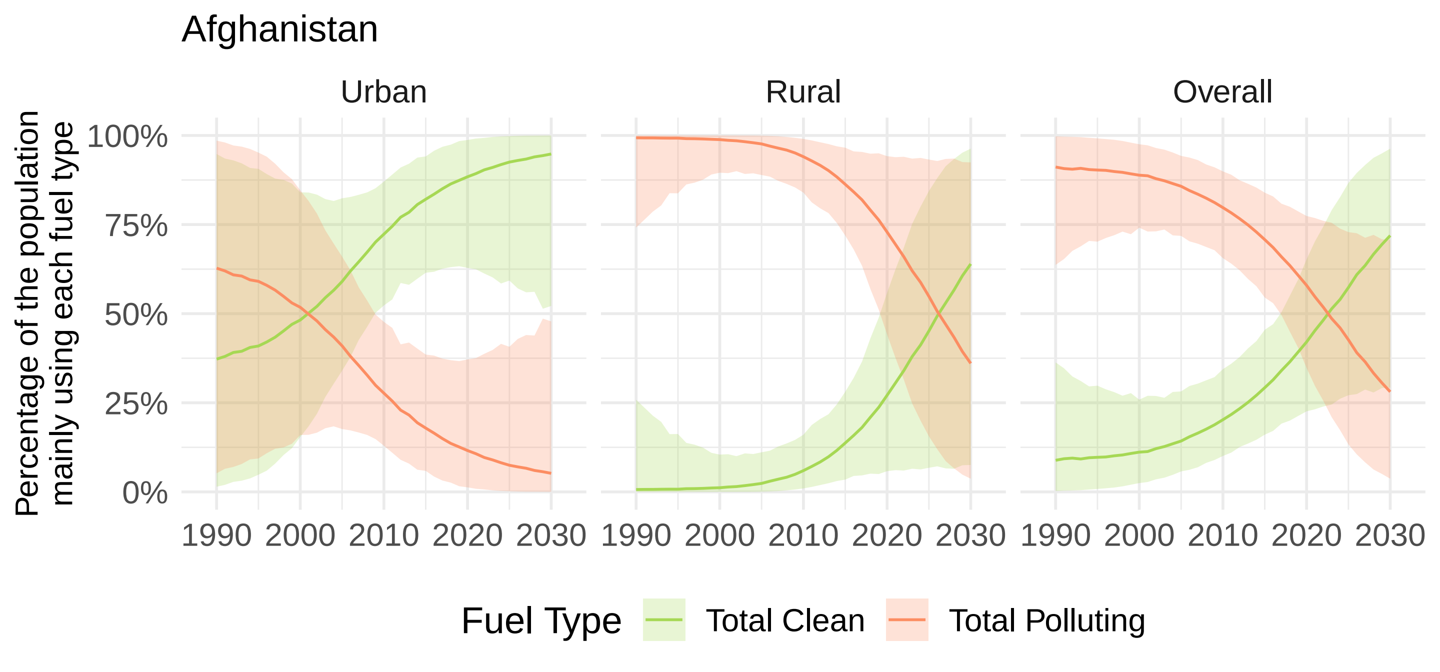
Afghanistan
Urban
Rural
Overall
Percentage of the population mainly using each fuel type
100%
75%
50%
25%
0%
1990
2000
2010
2020
2030
Fuel Type
Total Clean
Total Polluting

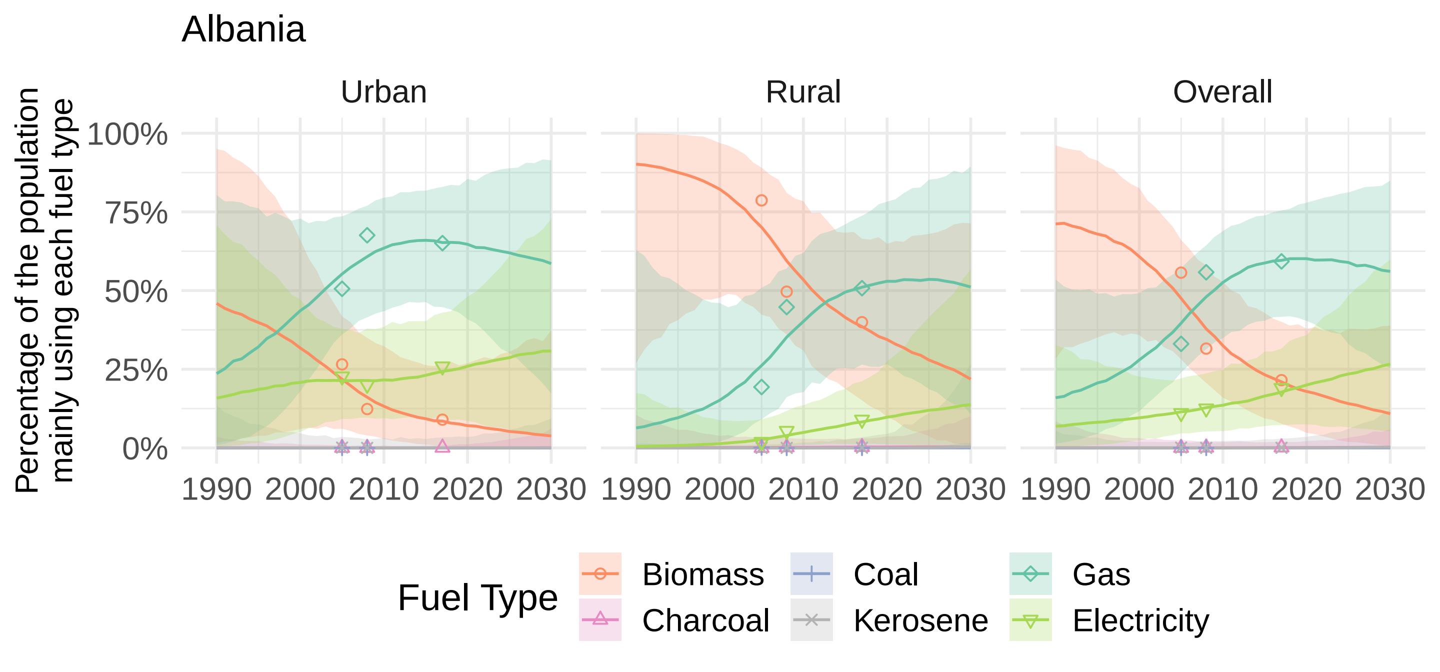

Albania
Urban
Rural
Overall
Percentage of the population mainly using each fuel type
100%
75%
50%
25%
0%
1990
2000
2010
2020
2030
Fuel Type
Biomass
Charcoal
Coal
Kerosene
Gas
Electricity

Albania

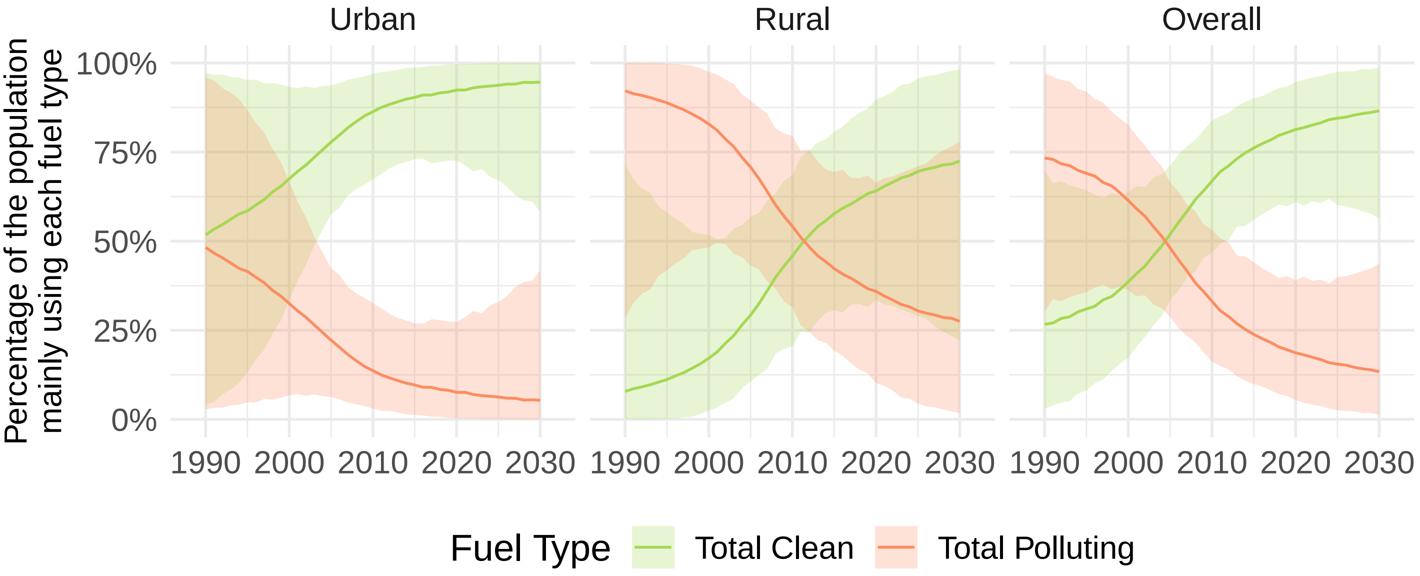

Urban
Rural
Overall
Percentage of the population mainly using each fuel type
100%
75%
50%
25%
0%
1990
2000
2010
2020
2030
Fuel Type
Total Clean
Total Polluting

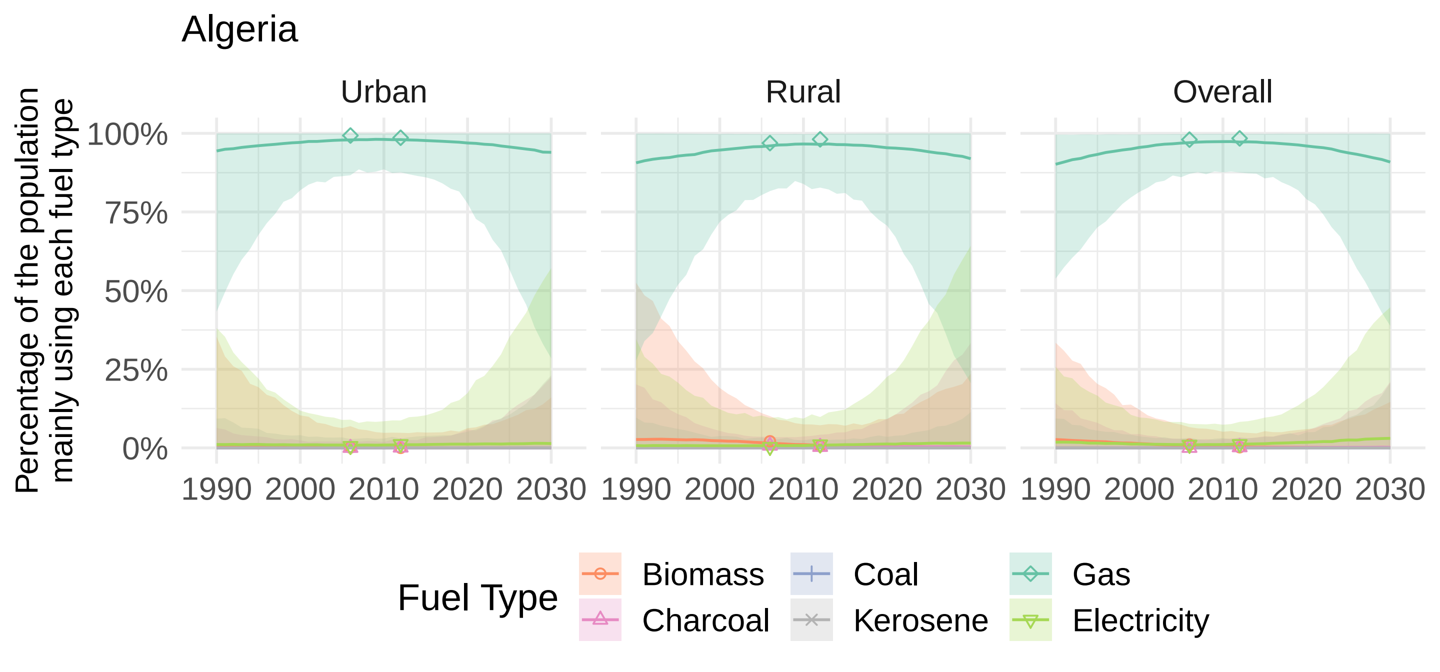

Algeria
Urban
Rural
Overall
Percentage of the population mainly using each fuel type
100%
75%
50%
25%
0%
1990
2000
2010
2020
2030
Fuel Type
Biomass
Charcoal
Coal
Kerosene
Gas
Electricity

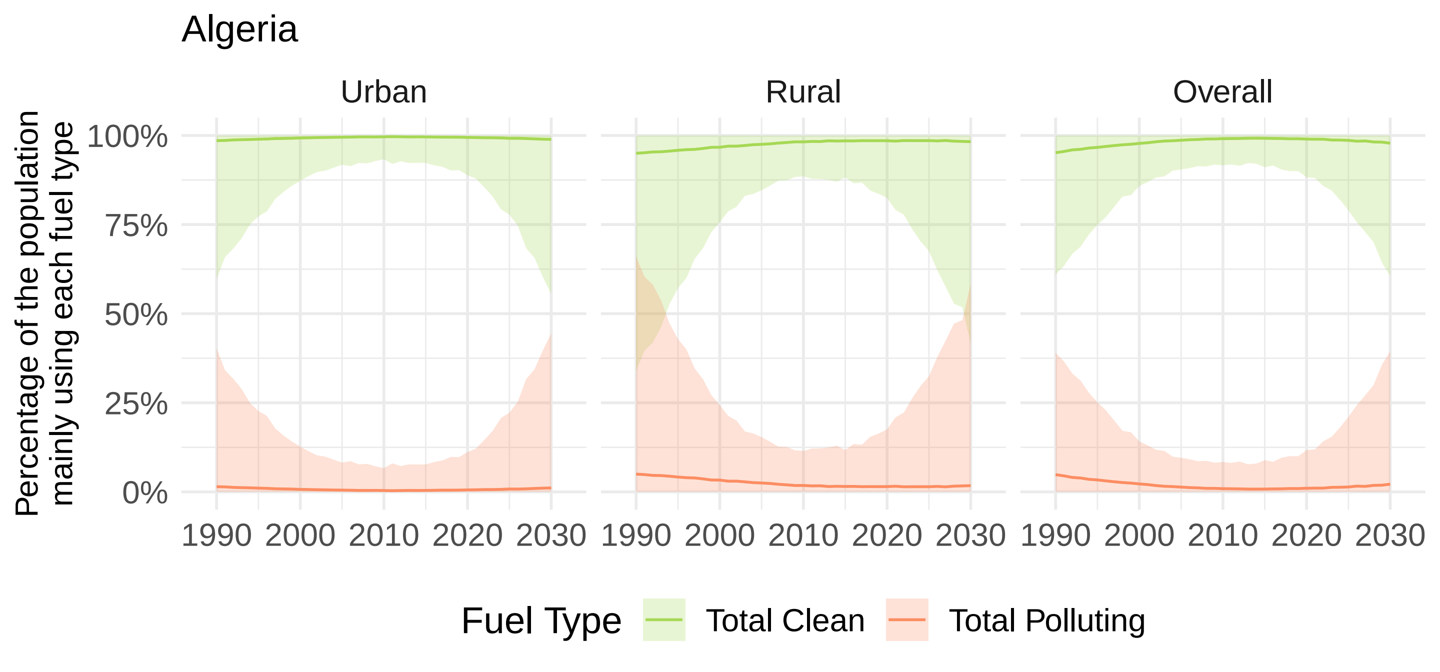
Algeria
Urban
Rural
Overall
Percentage of the population mainly using each fuel type
100%
75%
50%
25%
0%
1990
2000
2010
2020
2030
Fuel Type
Total Clean
Total Polluting

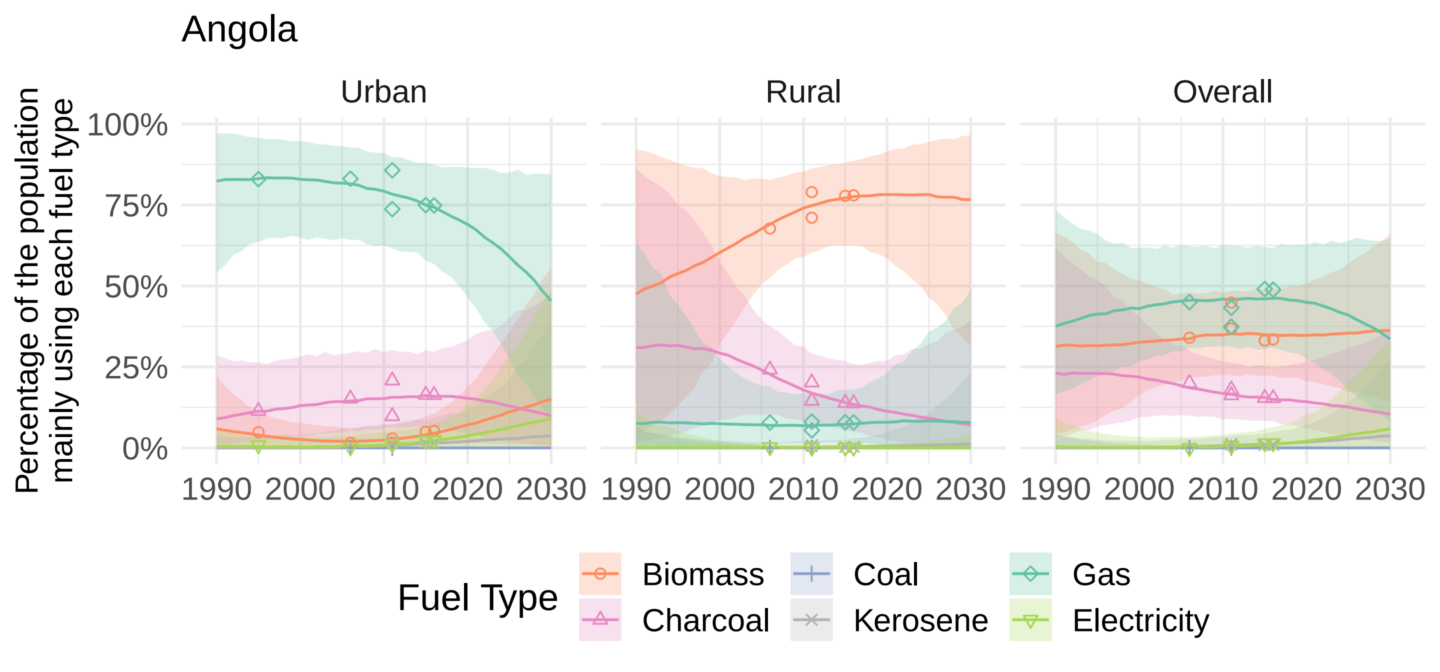

Angola
Urban
Rural
Overall
Percentage of the population mainly using each fuel type
100%
75%
50%
25%
0%
1990
2000
2010
2020
2030
Fuel Type
Biomass
Charcoal
Coal
Kerosene
Gas
Electricity

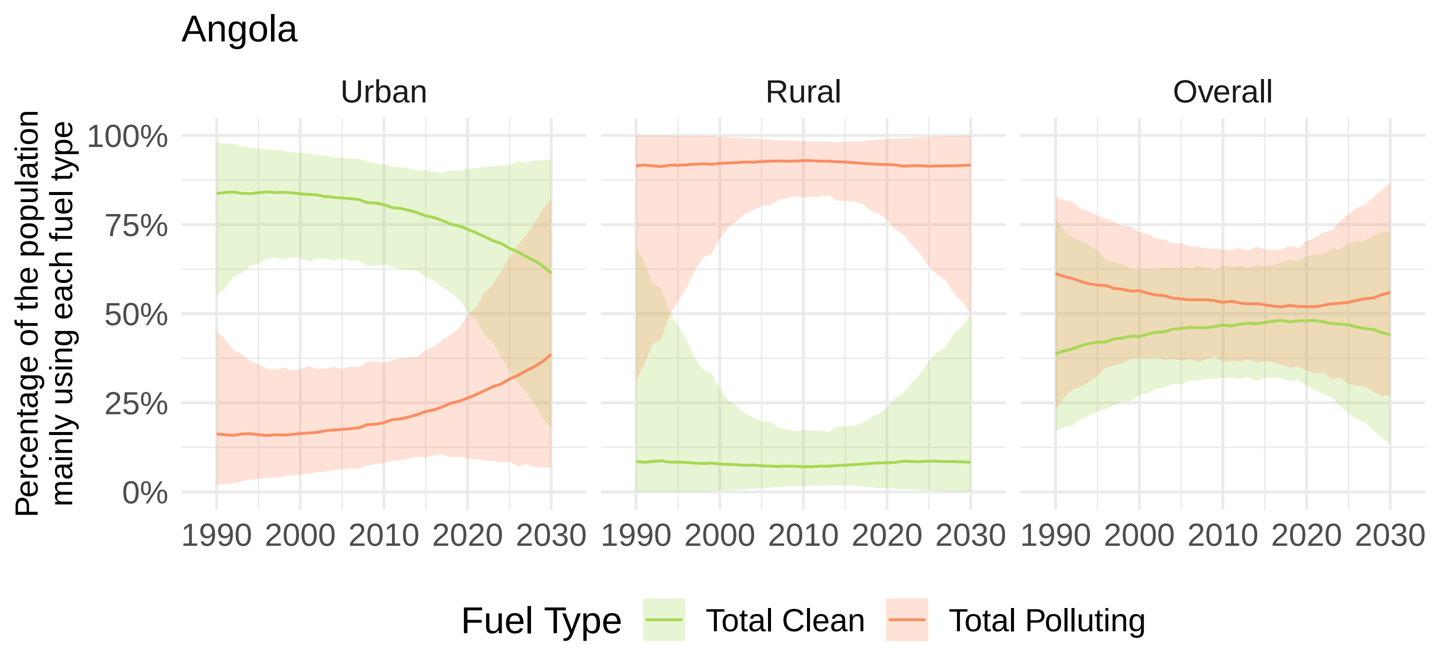
Angola
Urban
Rural
Overall
Percentage of the population mainly using each fuel type
100%
75%
50%
25%
0%
1990
2000
2010
2020
2030
Fuel Type
Total Clean
Total Polluting

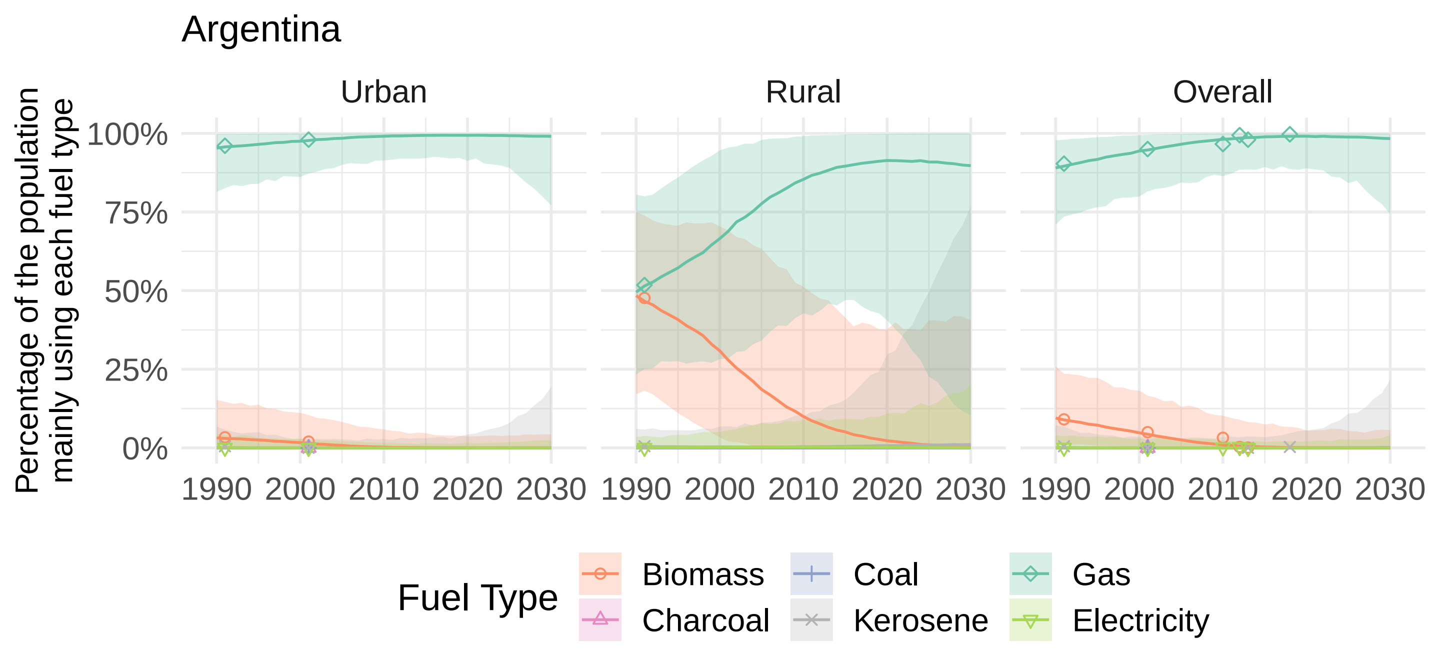

Argentina
Urban
Rural
Overall
Percentage of the population mainly using each fuel type
100%
75%
50%
25%
0%
1990
2000
2010
2020
2030
Fuel Type
Biomass
Charcoal
Coal
Kerosene
Gas
Electricity

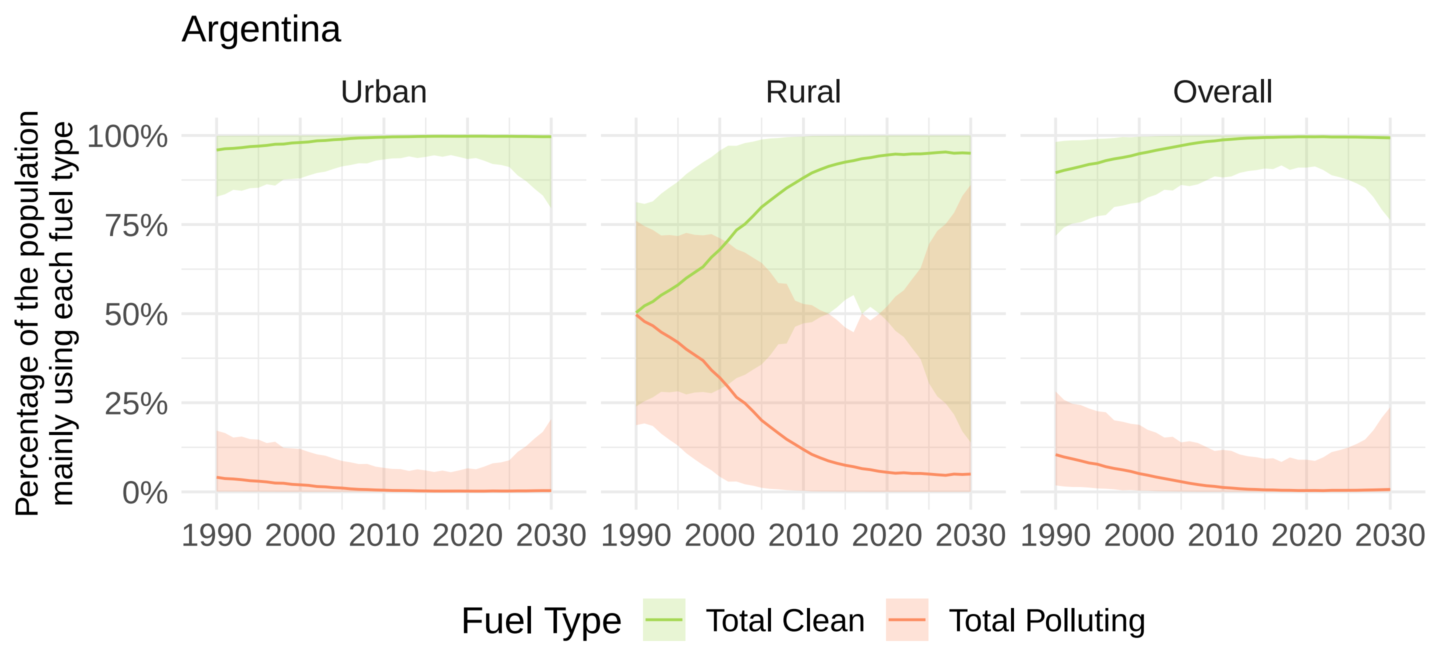

Argentina
Urban
Rural
Overall
Percentage of the population mainly using each fuel type
100%
75%
50%
25%
0%
1990
2000
2010
2020
2030
Fuel Type
Total Clean
Total Polluting

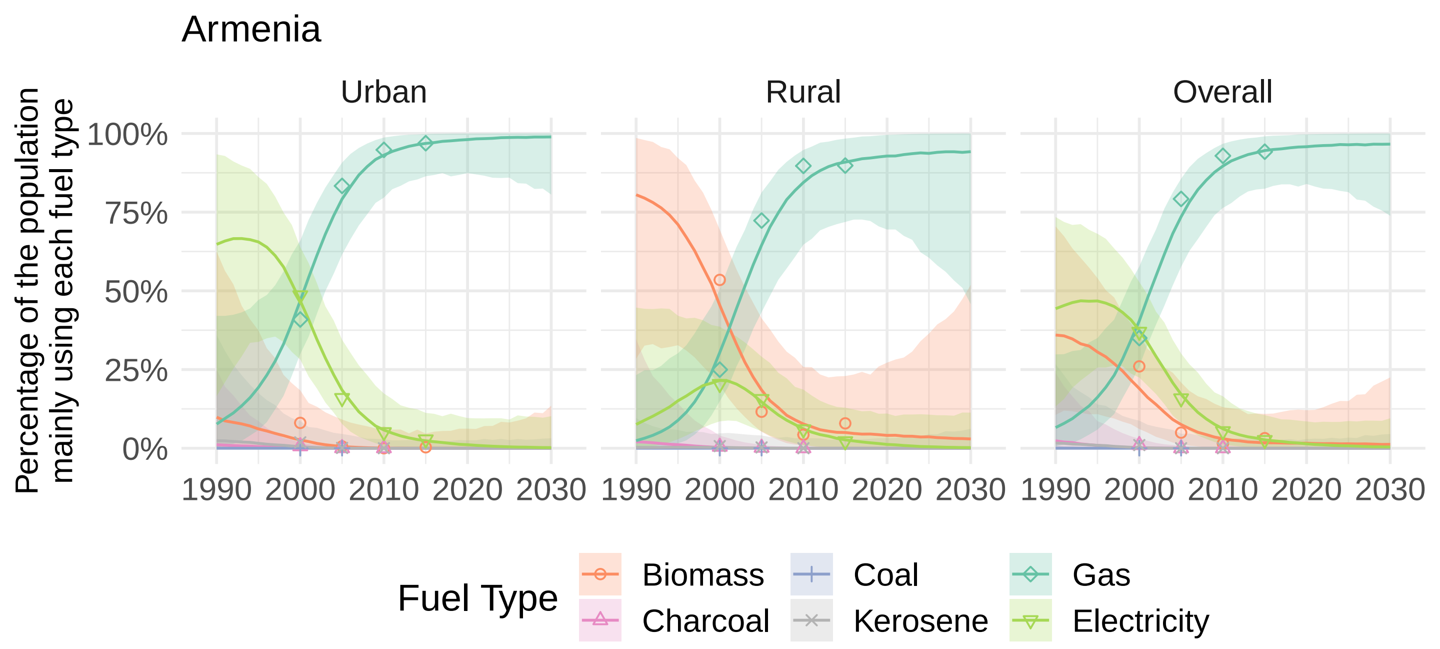
Armenia
Urban
Rural
Overall
Percentage of the population mainly using each fuel type
100%
75%
50%
25%
0%
1990
2000
2010
2020
2030
Fuel Type
Biomass
Charcoal
Coal
Kerosene
Gas
Electricity

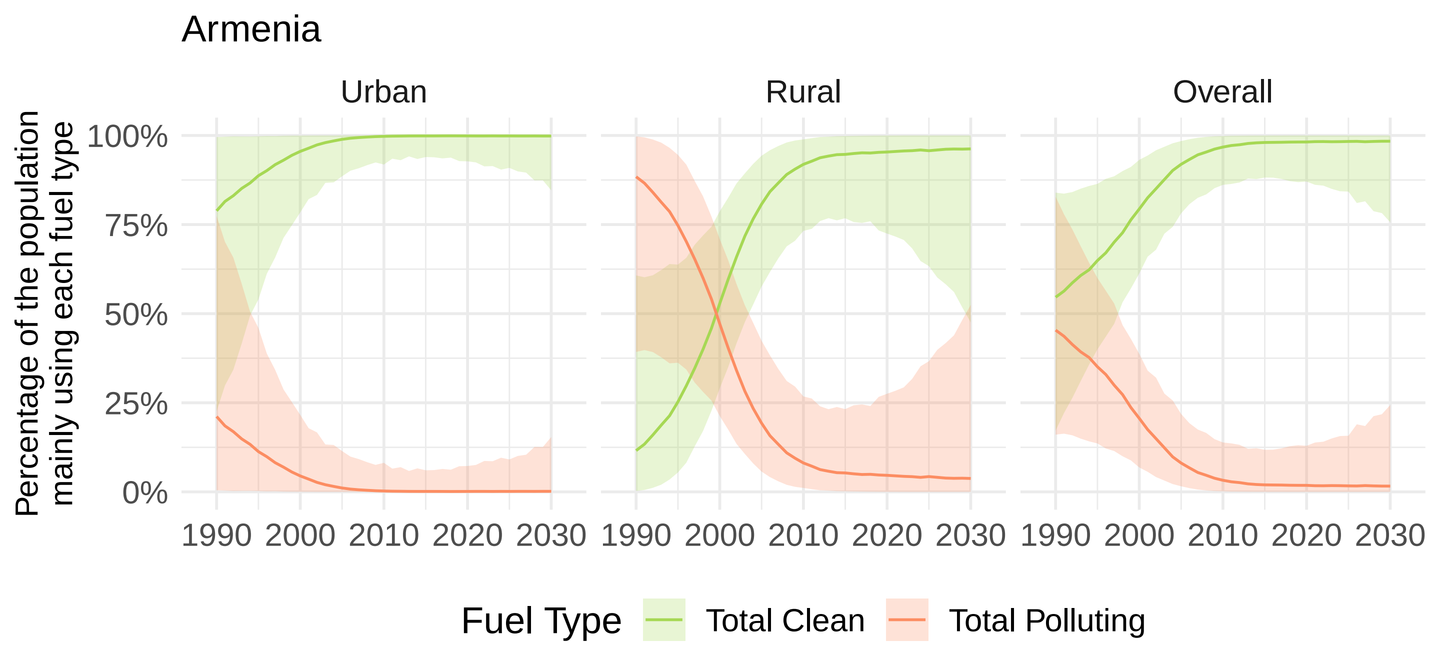
Armenia
Urban
Rural
Overall
Percentage of the population mainly using each fuel type
100%
75%
50%
25%
0%
1990
2000
2010
2020
2030
Fuel Type
Total Clean
Total Polluting

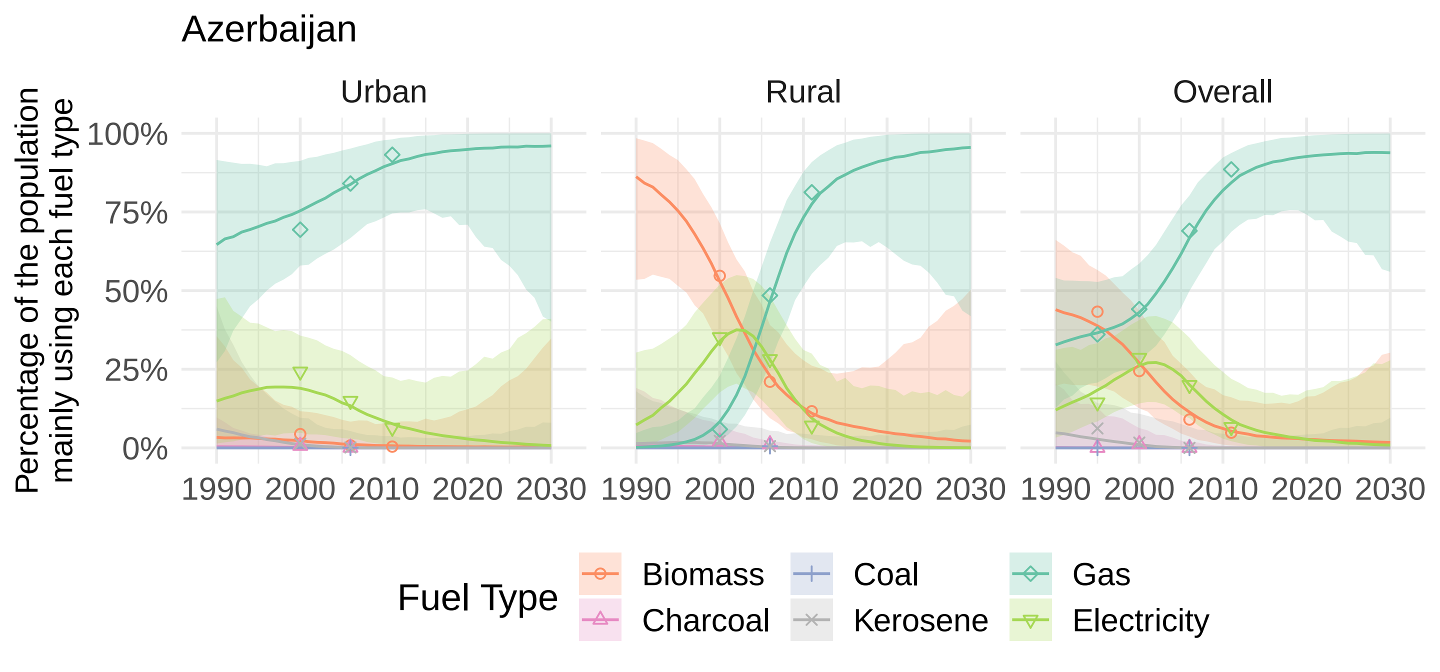

Azerbaijan
Urban
Rural
Overall
Percentage of the population mainly using each fuel type
100%
75%
50%
25%
0%
1990
2000
2010
2020
2030
Fuel Type
Biomass
Charcoal
Coal
Kerosene
Gas
Electricity

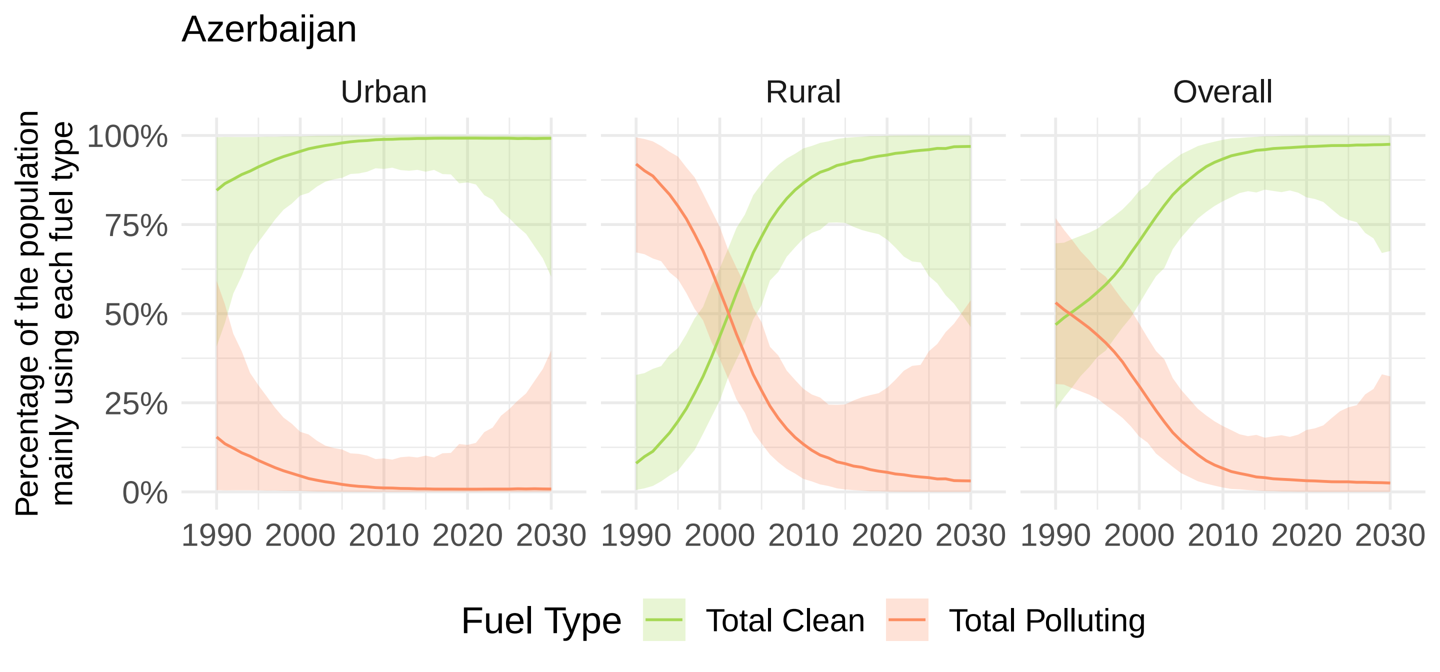
Azerbaijan
Urban
Rural
Overall
Percentage of the population mainly using each fuel type
100%
75%
50%
25%
0%
1990
2000
2010
2020
2030
Fuel Type
Total Clean
Total Polluting

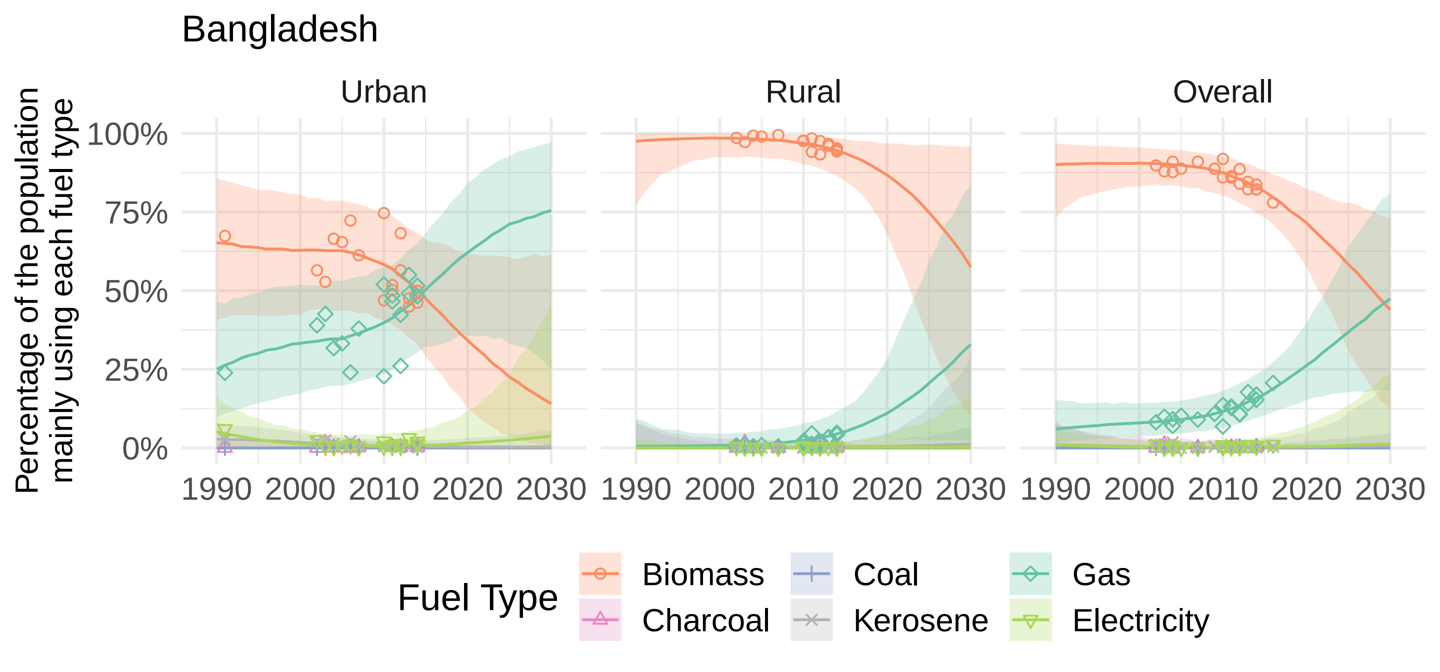

Bangladesh
Urban
Rural
Overall
Percentage of the population mainly using each fuel type
100%
75%
50%
25%
0%
1990
2000
2010
2020
2030
Fuel Type
Biomass
Charcoal
Coal
Kerosene
Gas
Electricity

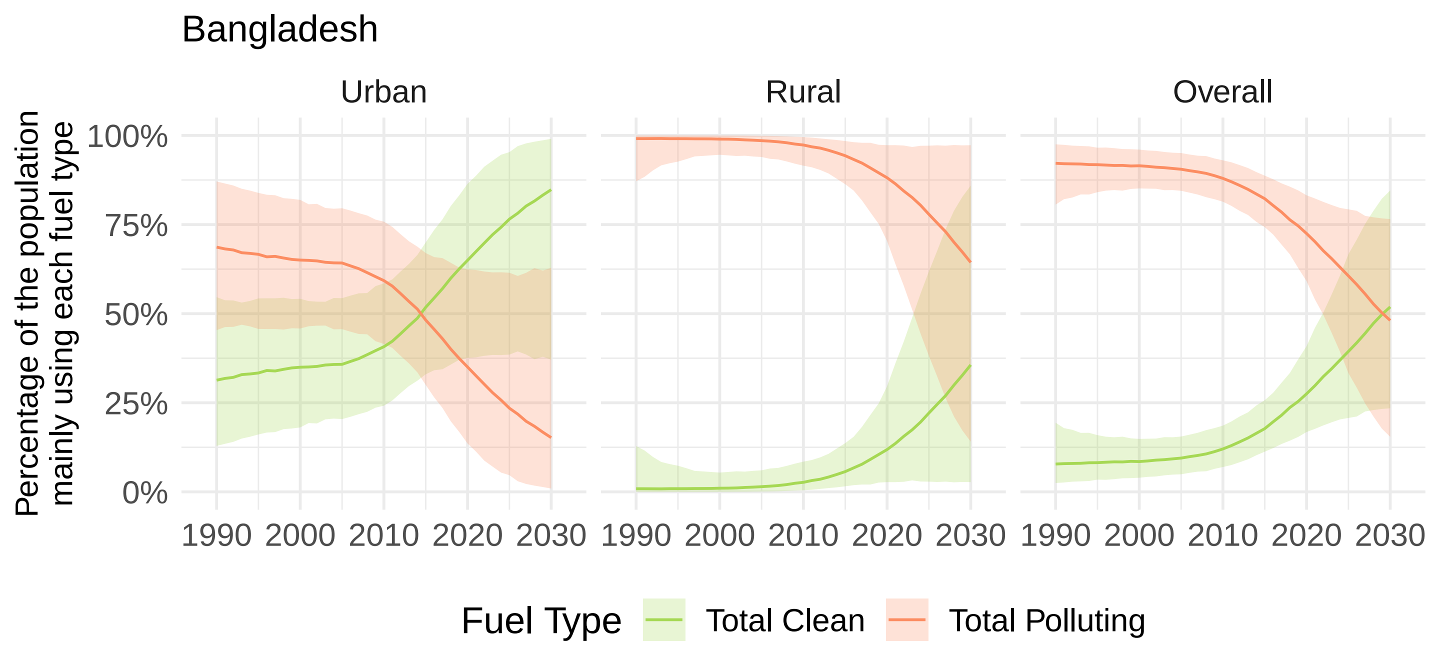
Bangladesh
Urban
Rural
Overall
Percentage of the population mainly using each fuel type
100%
75%
50%
25%
0%
1990
2000
2010
2020
2030
Fuel Type
Total Clean
Total Polluting

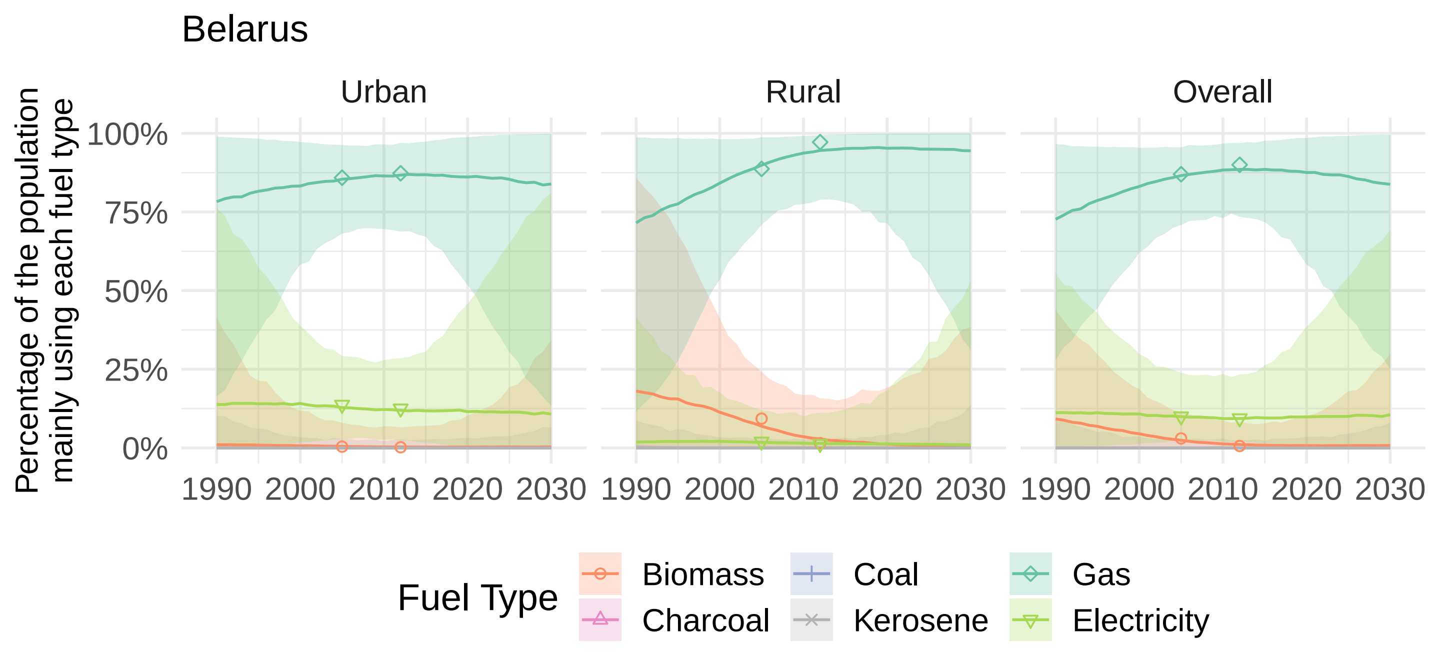
Belarus
Urban
Rural
Overall
Percentage of the population mainly using each fuel type
100%
75%
50%
25%
0%
1990
2000
2010
2020
2030
Fuel Type
Biomass
Charcoal
Coal
Kerosene
Gas
Electricity

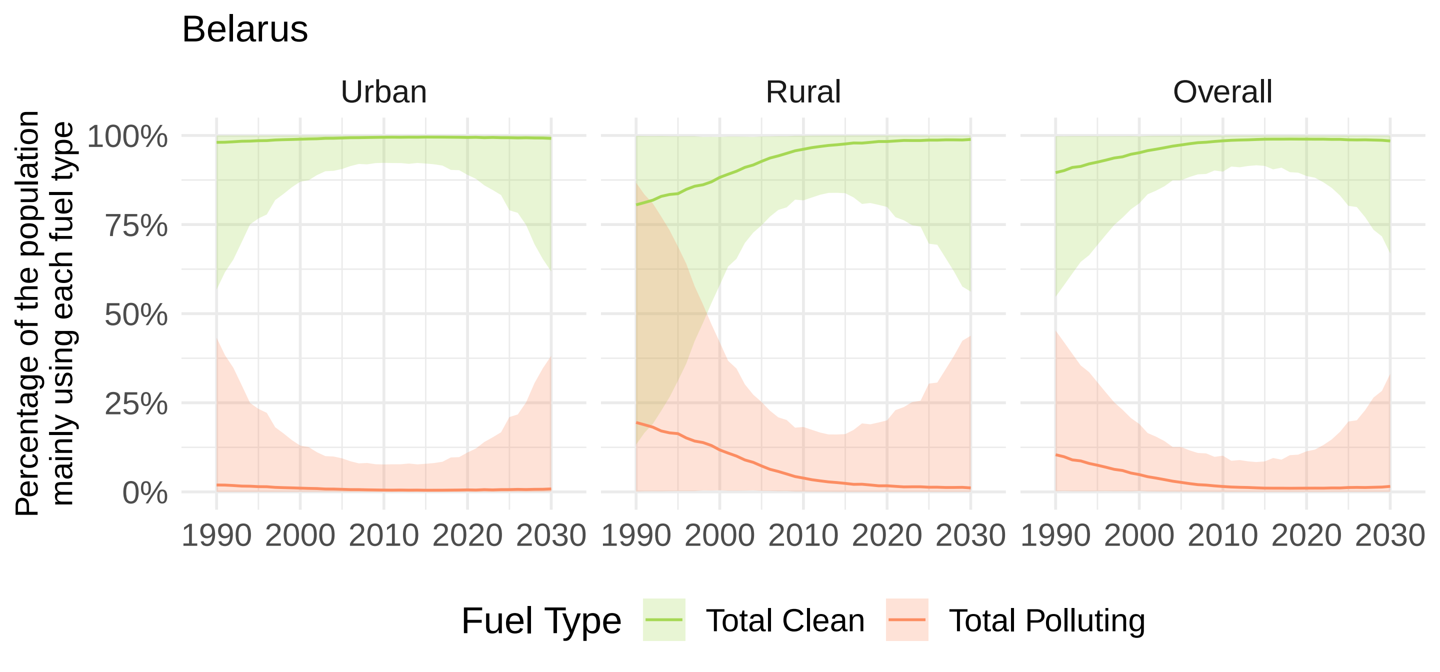

Belarus
Urban
Rural
Overall
Percentage of the population mainly using each fuel type
100%
75%
50%
25%
0%
1990
2000
2010
2020
2030
Fuel Type
Total Clean
Total Polluting

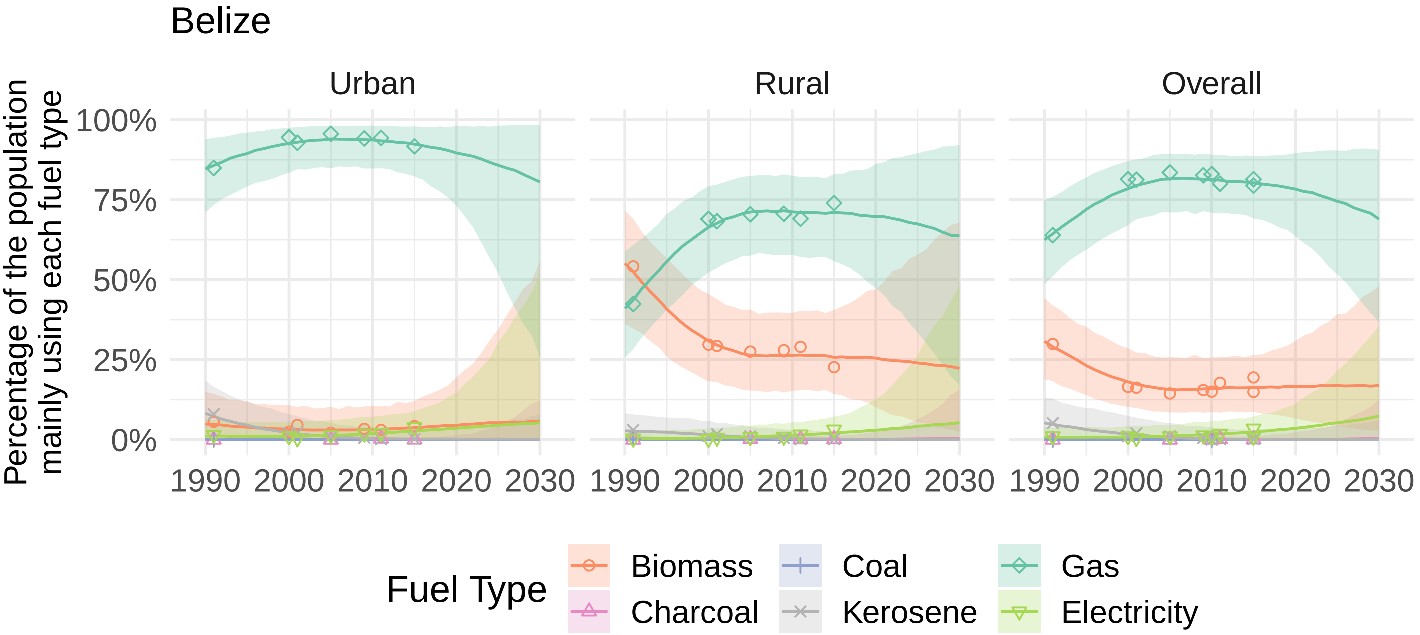

Belize
Urban
Rural
Overall
Percentage of the population mainly using each fuel type
100%
75%
50%
25%
0%
1990
2000
2010
2020
2030
Fuel Type
Biomass
Charcoal
Coal
Kerosene
Gas
Electricity

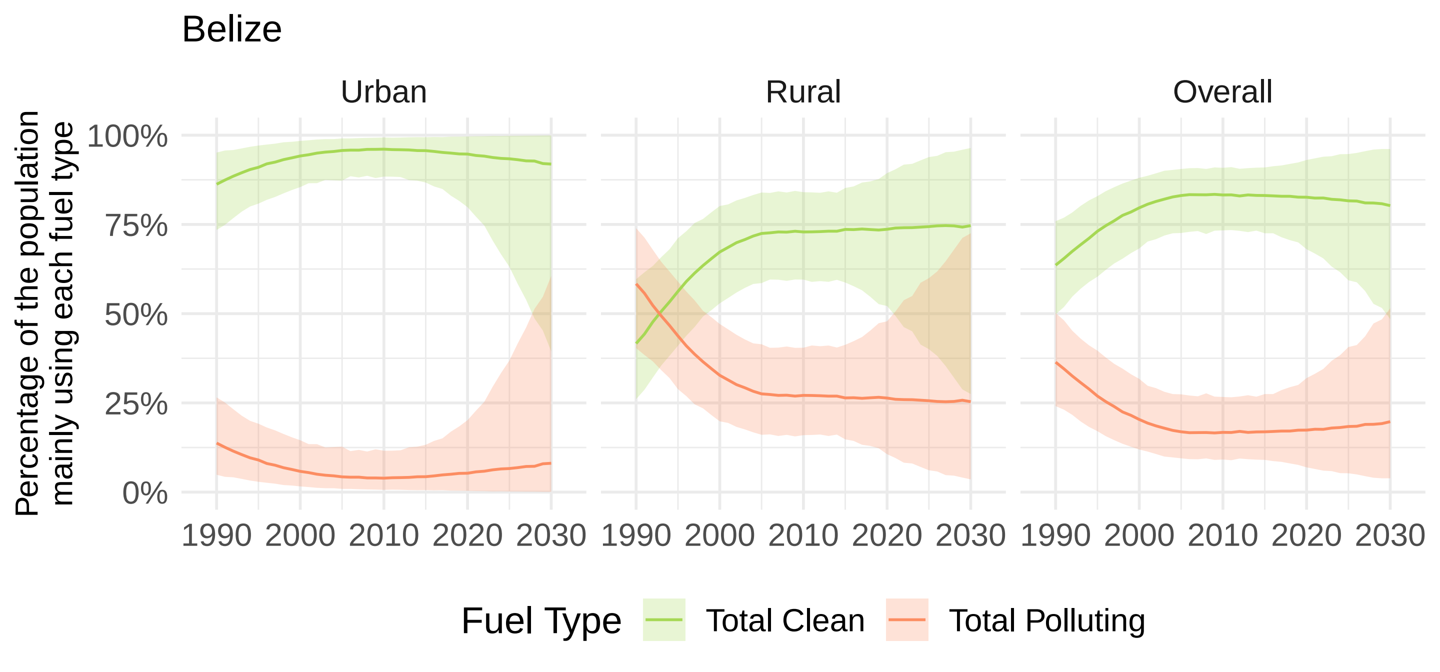
Belize
Urban
Rural
Overall
Percentage of the population
mainly using each fuel type
100%
75%
50%
25%
0%
1990
2000
2010
2020
2030
Fuel Type
Total Clean
Total Polluting

Benin

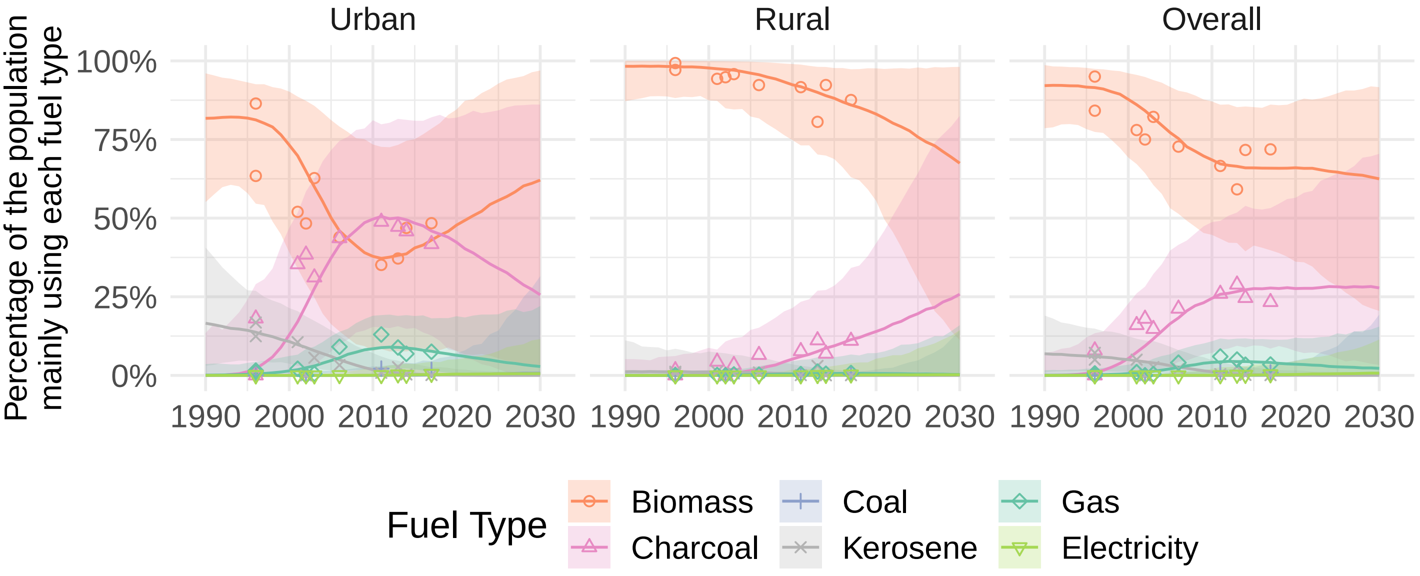

Urban
Rural
Overall
Percentage of the population mainly using each fuel type
100%
75%
50%
25%
0%
1990
2000
2010
2020
2030
Fuel Type
Biomass
Charcoal
Coal
Kerosene
Gas
Electricity

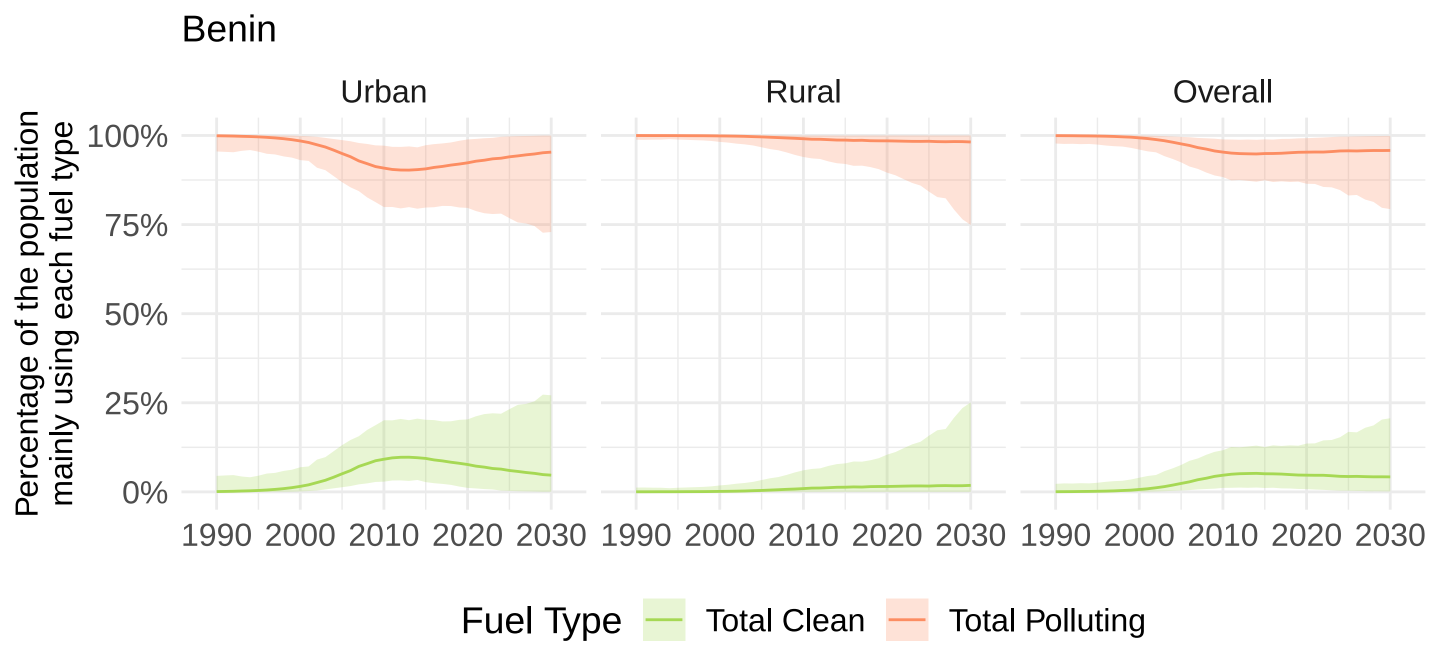

Benin
Urban
Rural
Overall
Percentage of the population mainly using each fuel type
100%
75%
50%
25%
0%
1990
2000
2010
2020
2030
Fuel Type
Total Clean
Total Polluting

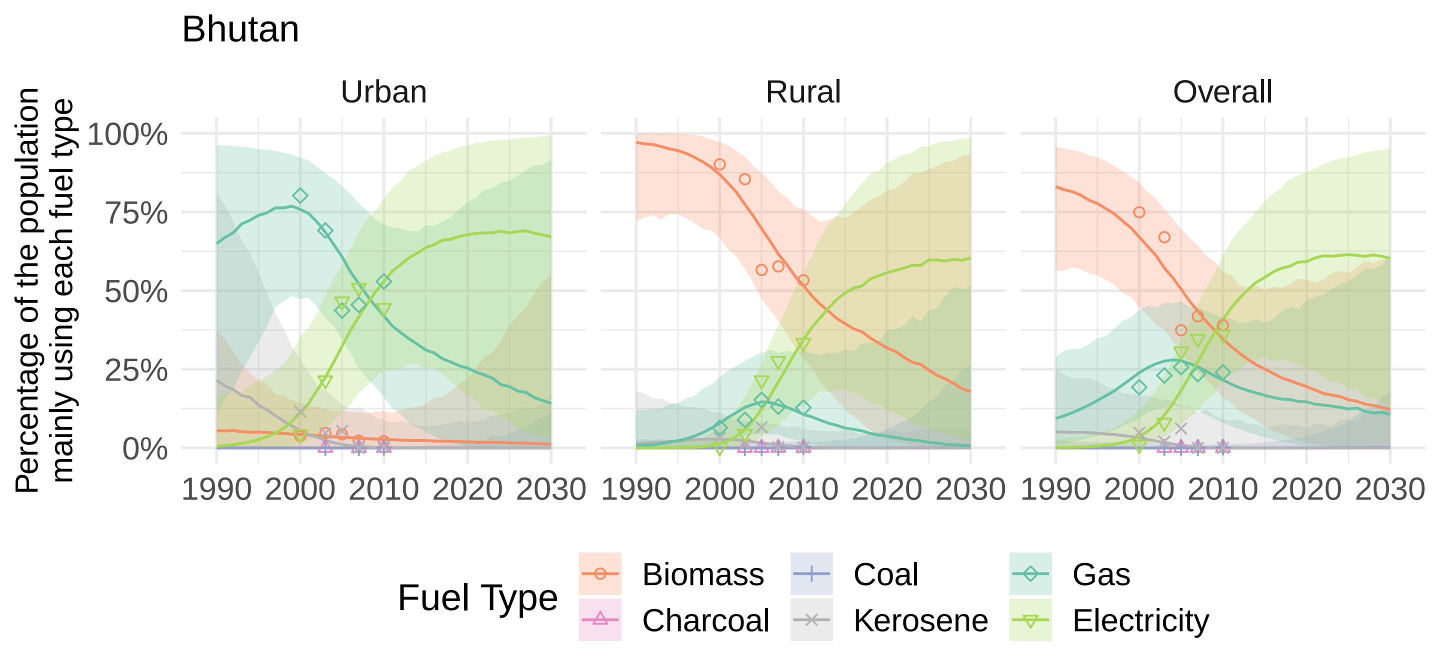
Bhutan
Urban
Rural
Overall
Percentage of the population
mainly using each fuel type
100%
75%
50%
25%
0%
1990
2000
2010
2020
2030
Fuel Type
Biomass
Charcoal
Coal
Kerosene
Gas
Electricity

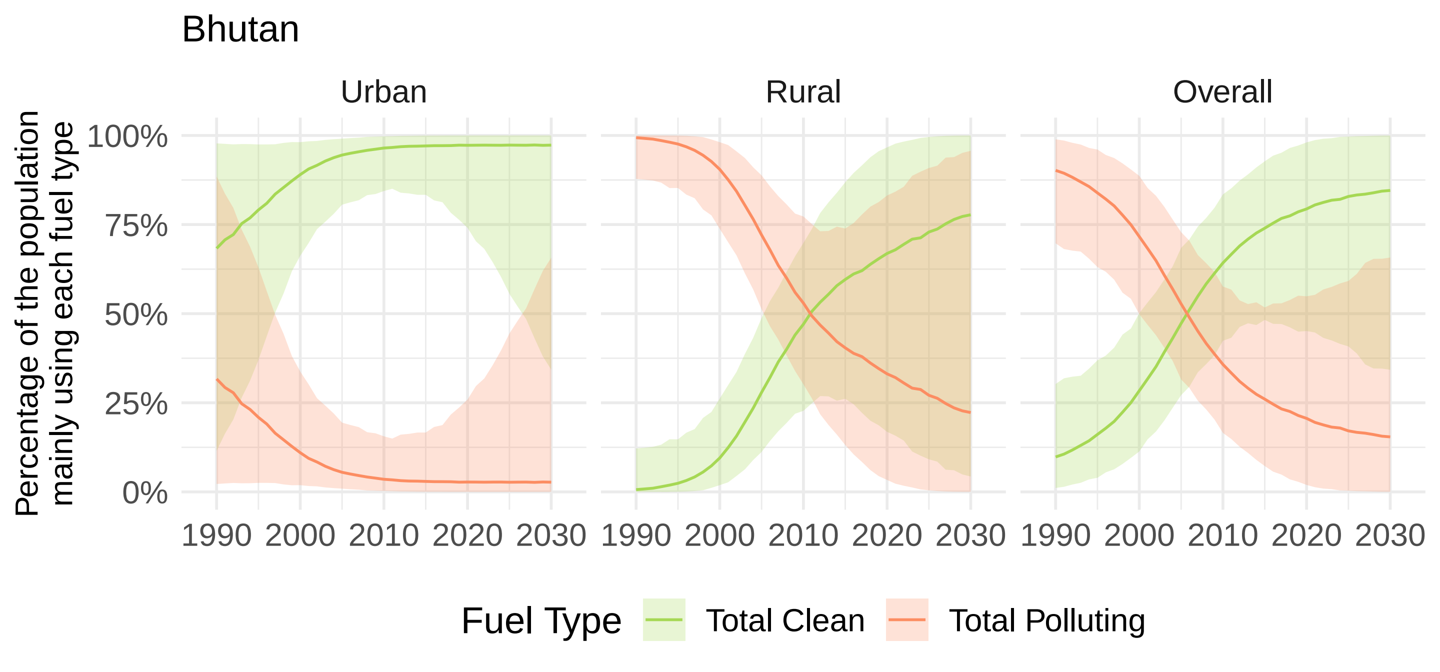

Bhutan
Urban
Rural
Overall
Percentage of the population mainly using each fuel type
100%
75%
50%
25%
0%
1990
2000
2010
2020
2030
Fuel Type
Total Clean
Total Polluting

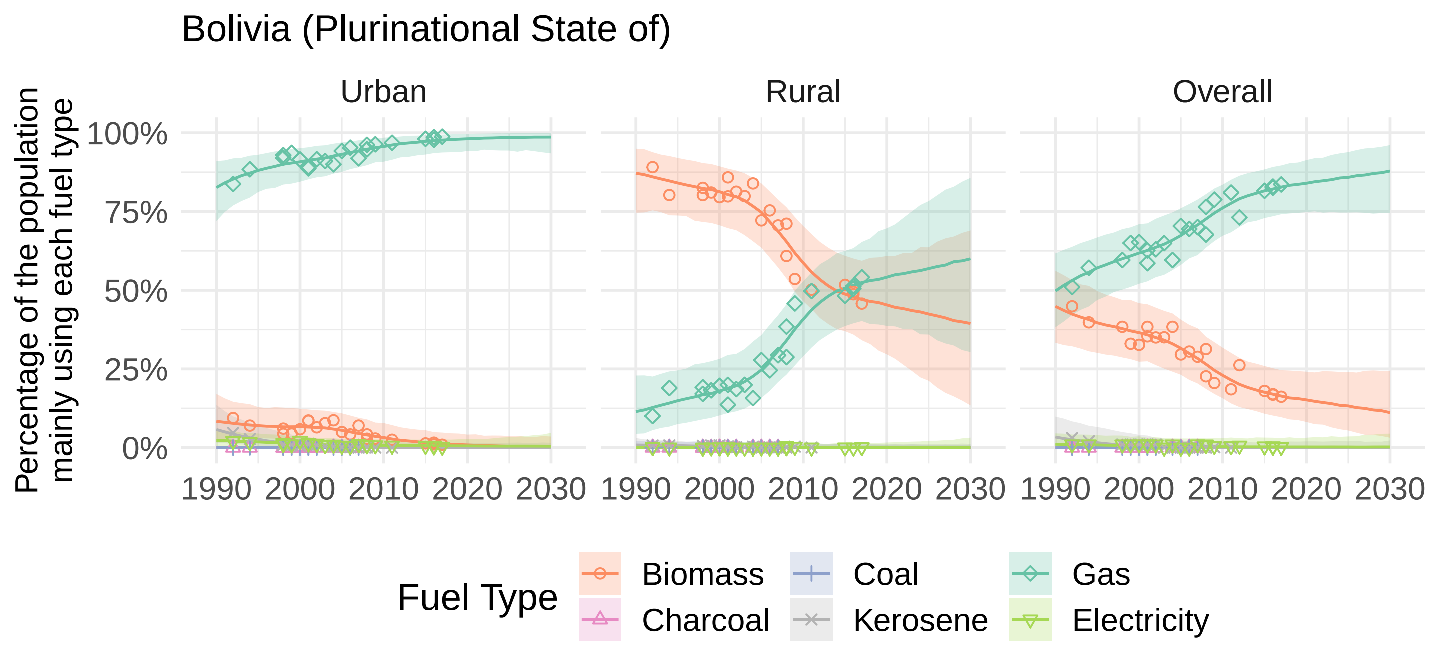

Bolivia (Plurinational State of)
Urban
Rural
Overall
Percentage of the population mainly using each fuel type
100%
75%
50%
25%
0%
1990
2000
2010
2020
2030
Fuel Type
Biomass
Charcoal
Coal
Kerosene
Gas
Electricity

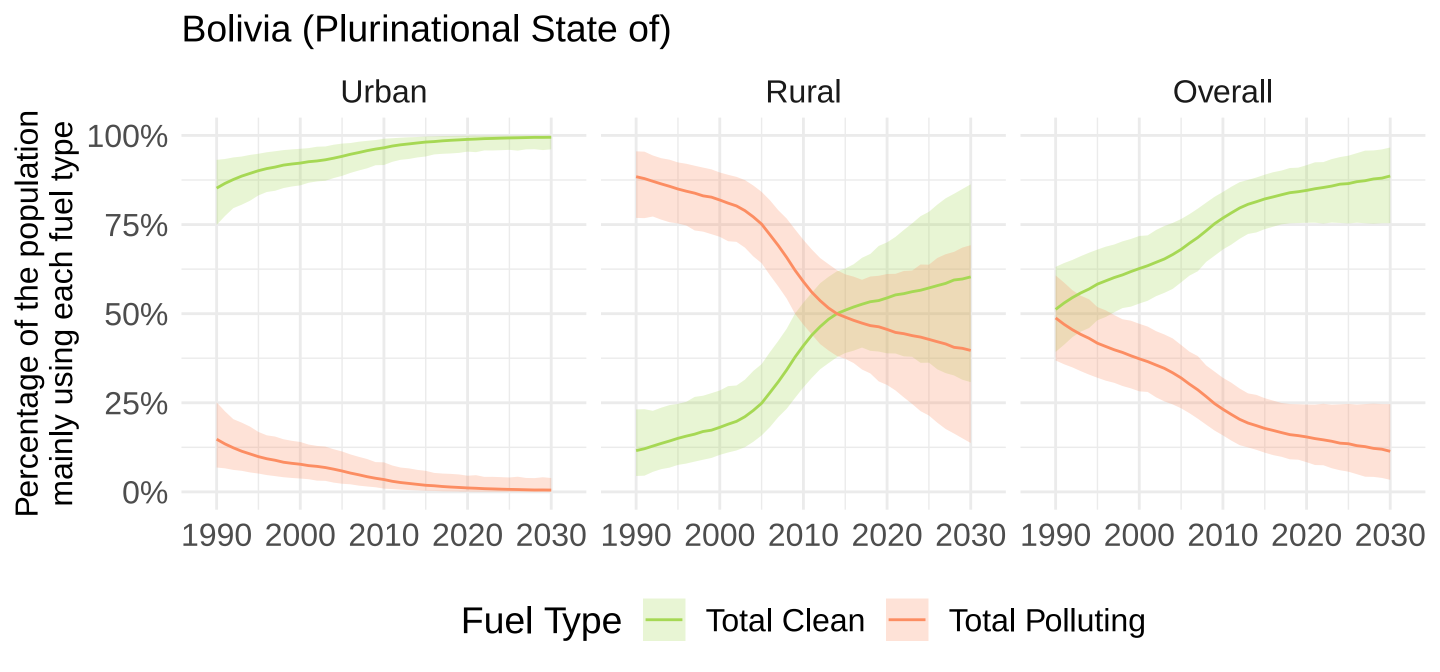

Bolivia (Plurinational State of)
Urban
Rural
Overall
Percentage of the population mainly using each fuel type
100%
75%
50%
25%
0%
1990
2000
2010
2020
2030
Fuel Type
Total Clean
Total Polluting

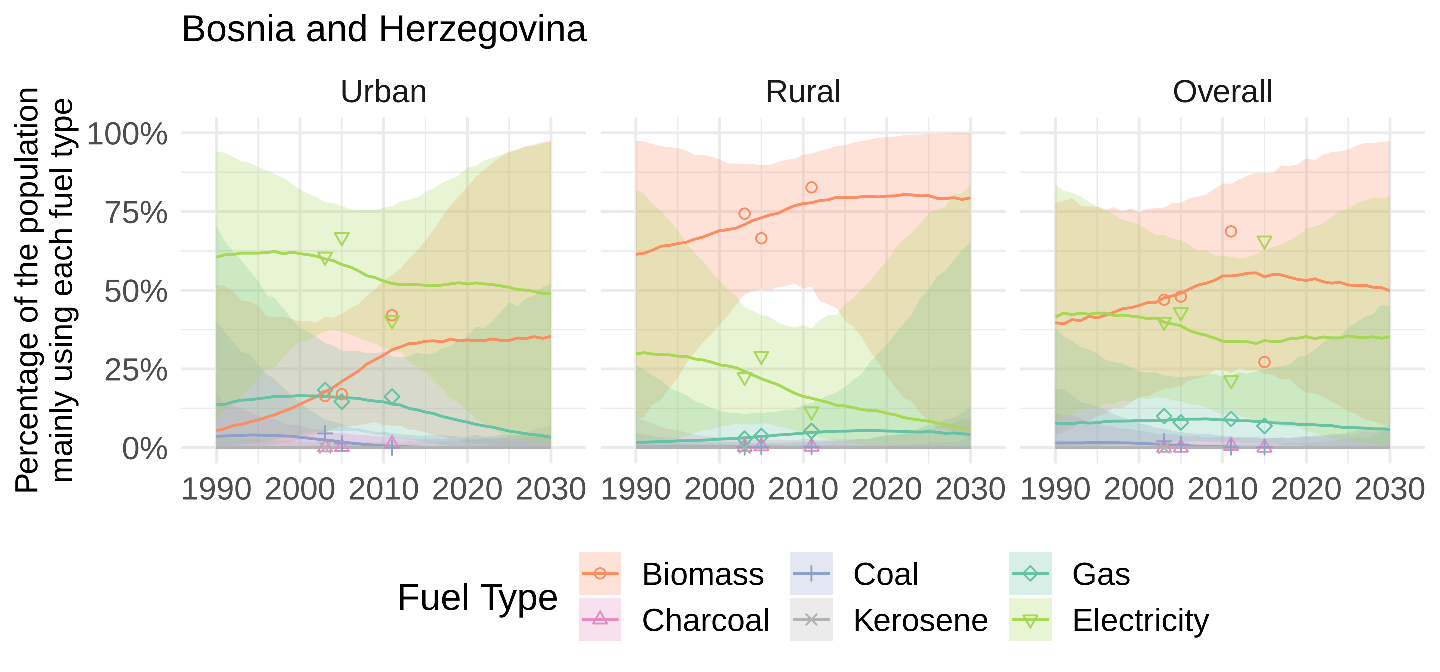
Bosnia and Herzegovina
Urban
Rural
Overall
Percentage of the population mainly using each fuel type
100%
75%
50%
25%
0%
1990
2000
2010
2020
2030
Fuel Type
Biomass
Charcoal
Coal
Kerosene
Gas
Electricity

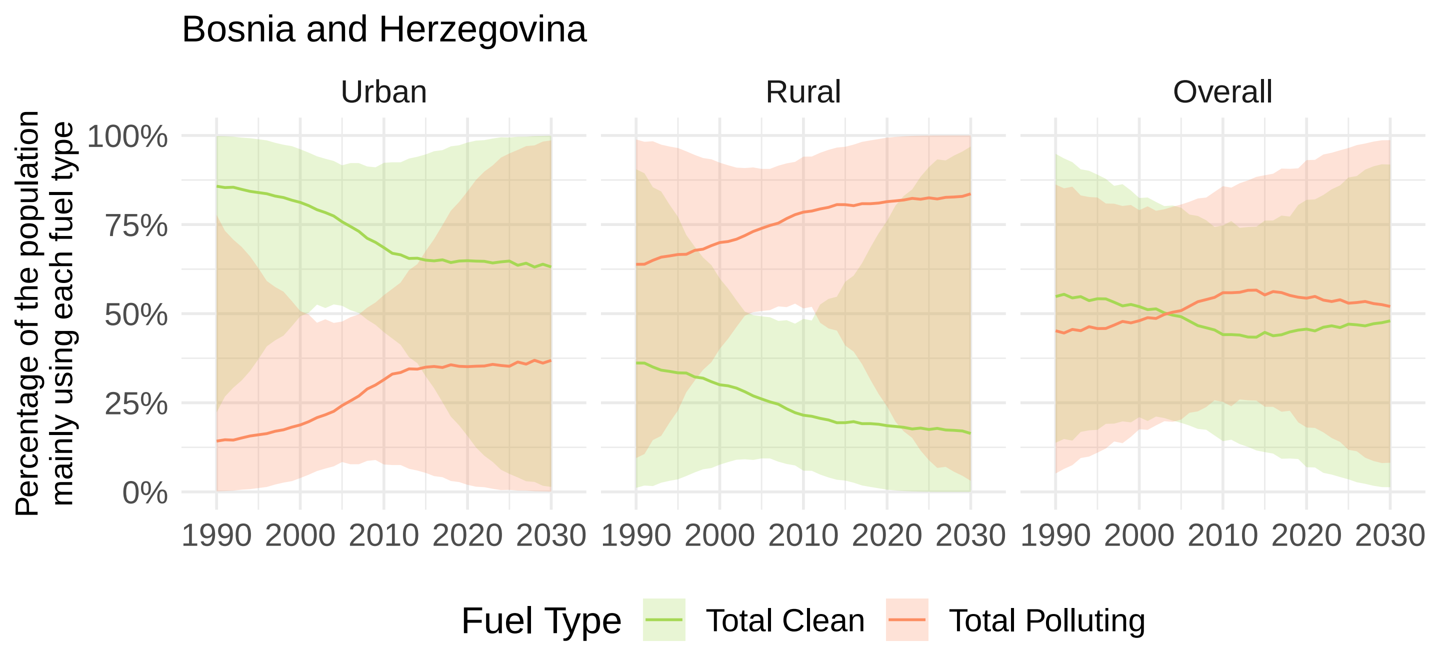
Bosnia and Herzegovina
Urban
Rural
Overall
Percentage of the population mainly using each fuel type
100%
75%
50%
25%
0%
1990
2000
2010
2020
2030
Fuel Type
Total Clean
Total Polluting

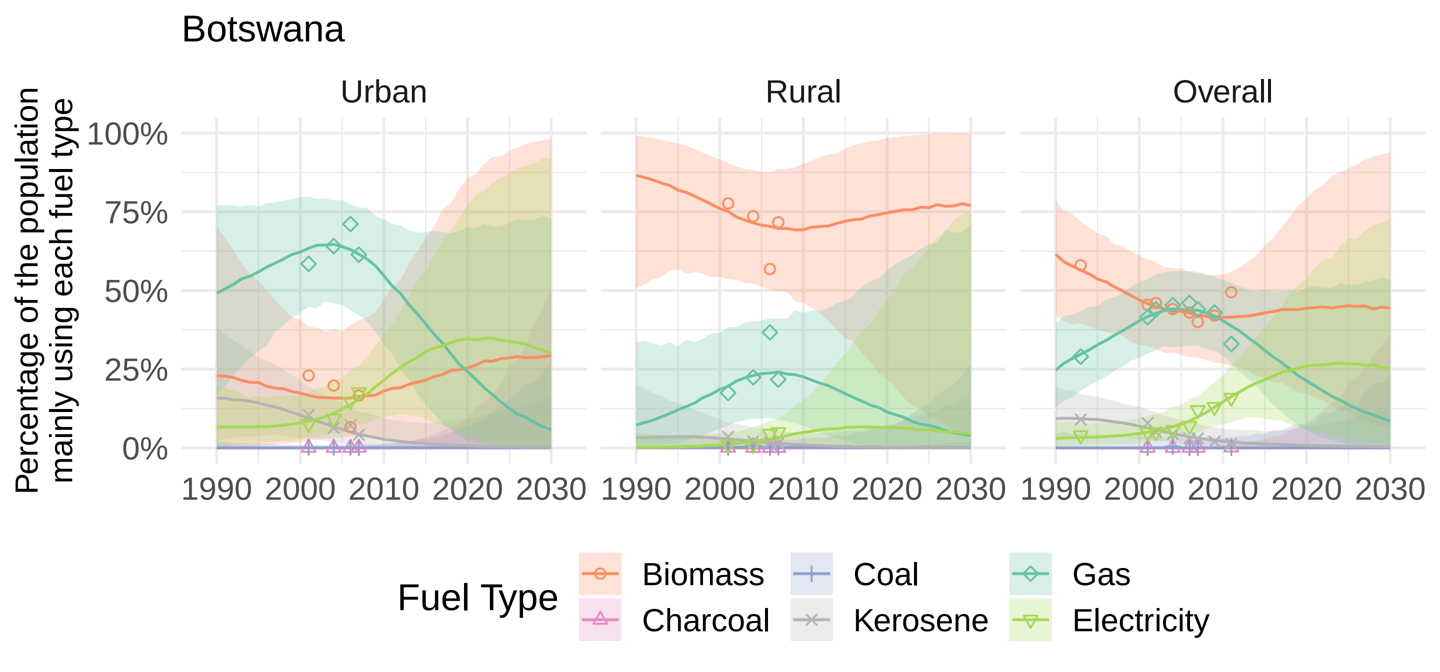

Botswana
Urban
Rural
Overall
Percentage of the population mainly using each fuel type
100%
75%
50%
25%
0%
1990
2000
2010
2020
2030
Fuel Type
Biomass
Charcoal
Coal
Kerosene
Gas
Electricity

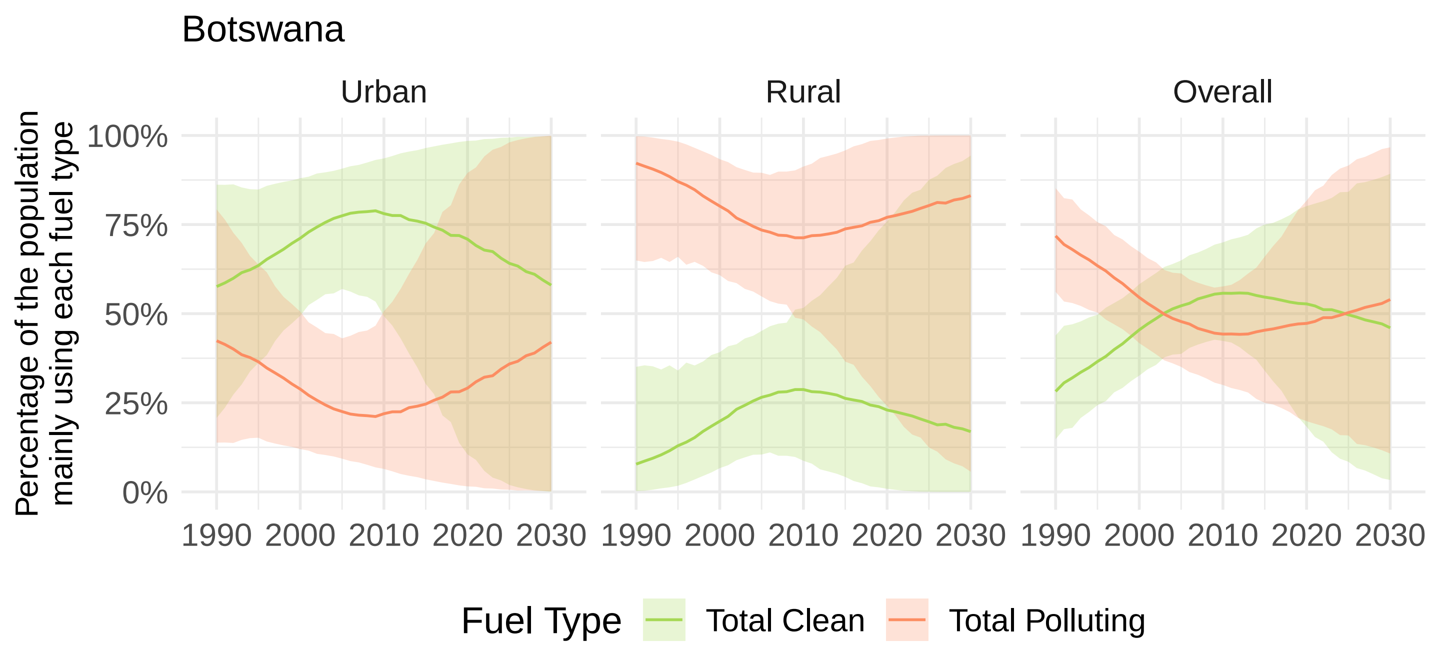
Botswana
Urban
Rural
Overall
Percentage of the population mainly using each fuel type
100%
75%
50%
25%
0%
1990
2000
2010
2020
2030
Fuel Type
Total Clean
Total Polluting

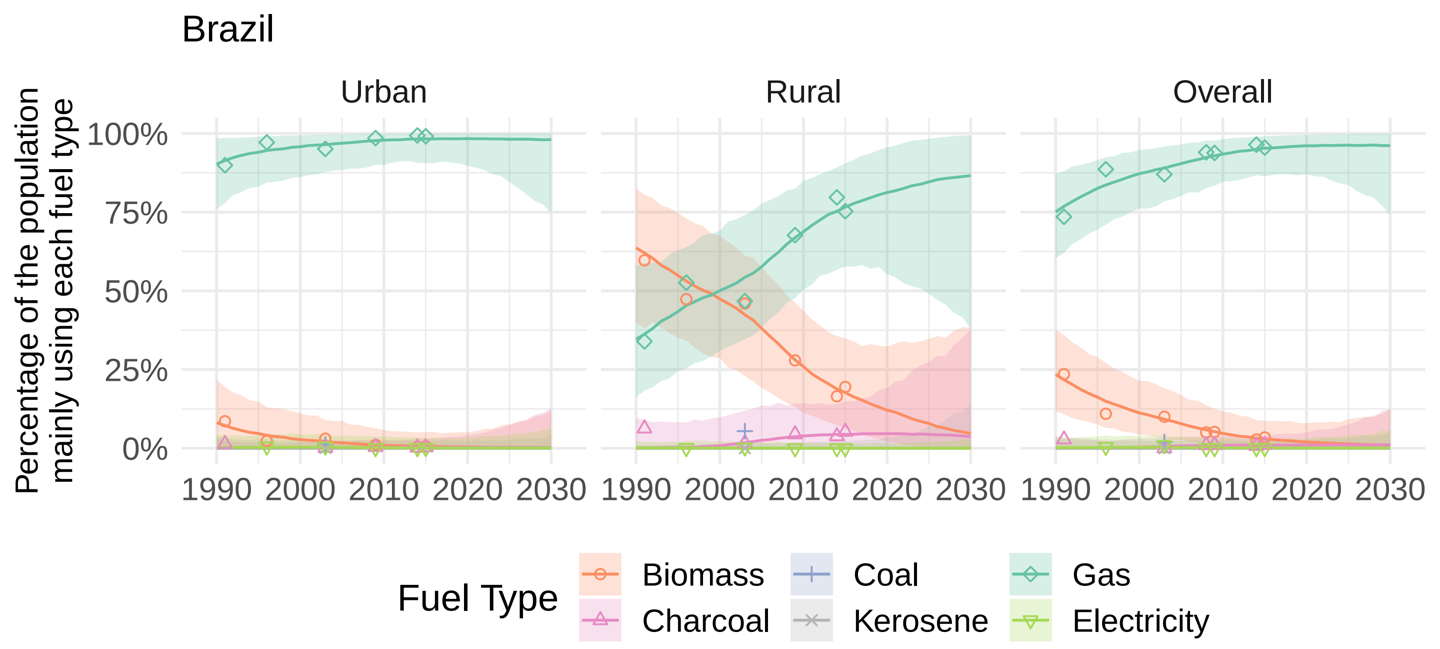
Brazil
Urban
Rural
Overall
Percentage of the population mainly using each fuel type
100%
75%
50%
25%
0%
1990
2000
2010
2020
2030
Fuel Type
Biomass
Charcoal
Coal
Kerosene
Gas
Electricity

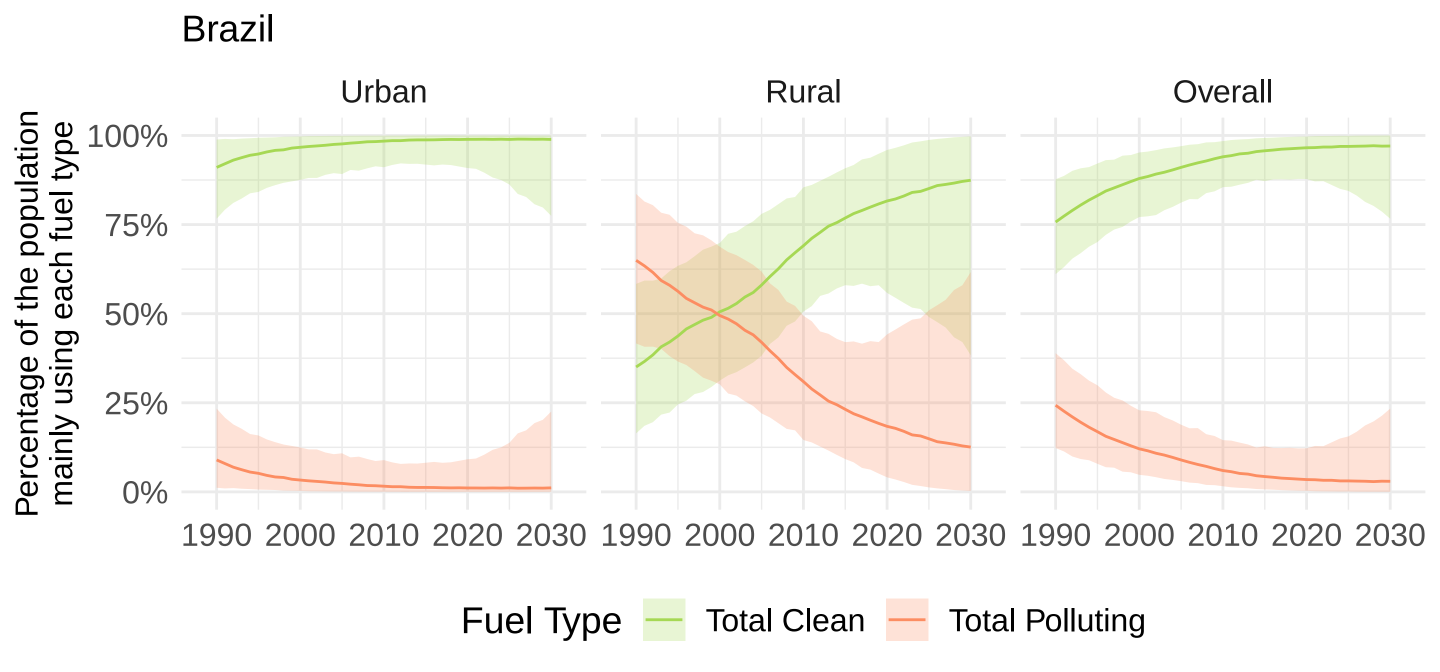

Brazil
Urban
Rural
Overall
Percentage of the population mainly using each fuel type
100%
75%
50%
25%
0%
1990
2000
2010
2020
2030
Fuel Type
Total Clean
Total Polluting

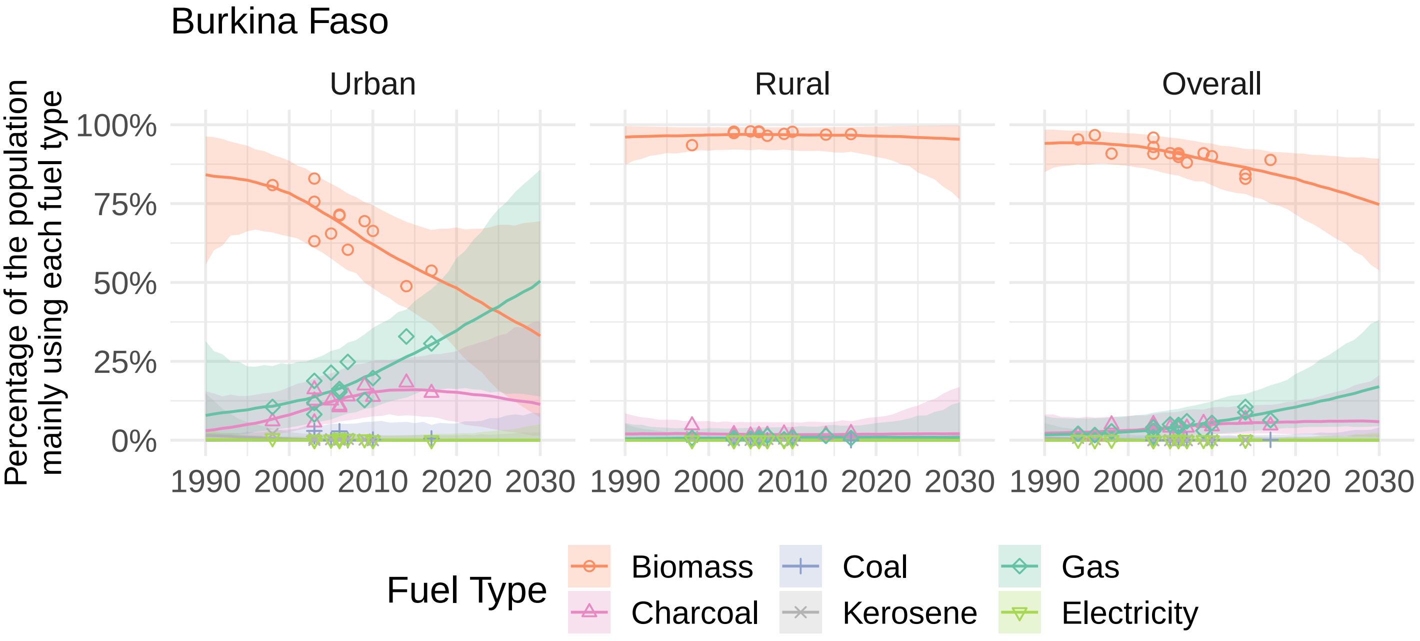
Burkina Faso
Urban
Rural
Overall
Percentage of the population mainly using each fuel type
100%
75%
50%
25%
0%
1990
2000
2010
2020
2030
Fuel Type
Biomass
Charcoal
Coal
Kerosene
Gas
Electricity

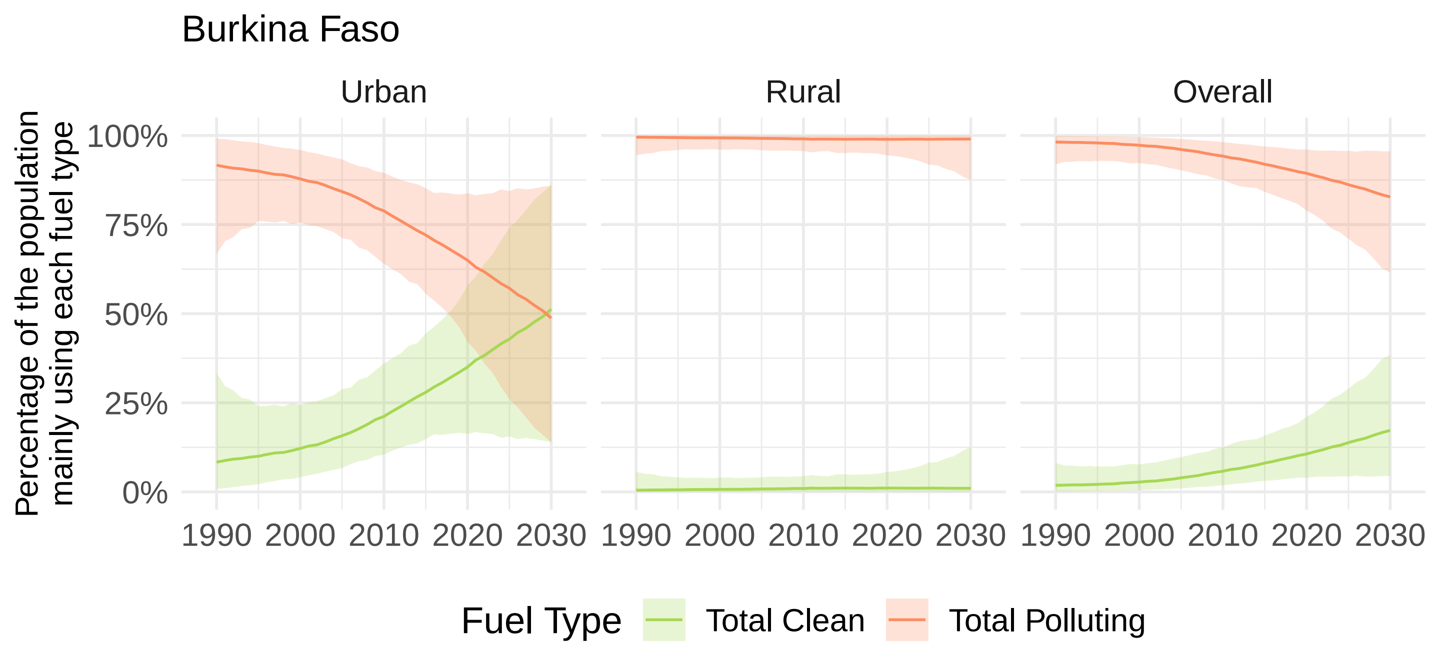

Burkina Faso
Urban
Rural
Overall
Percentage of the population mainly using each fuel type
100%
75%
50%
25%
0%
1990
2000
2010
2020
2030
Fuel Type
Total Clean
Total Polluting

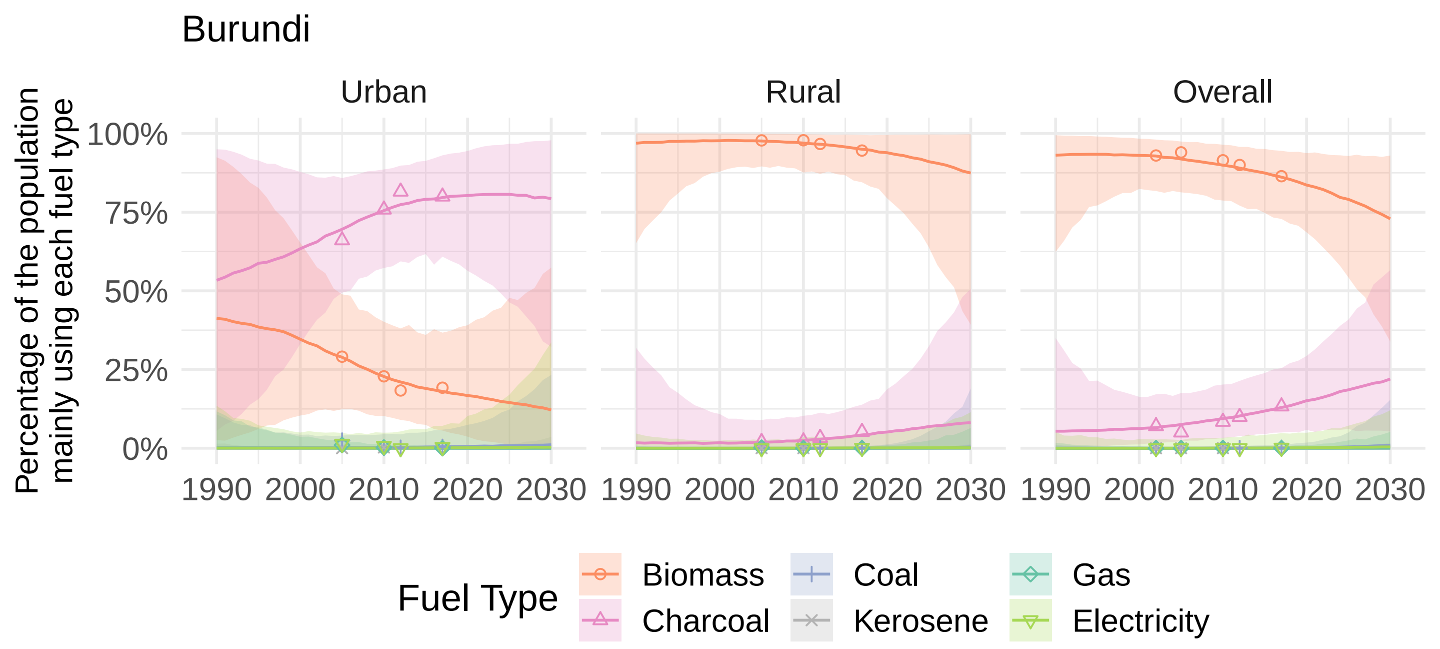
Burundi
Urban
Rural
Overall
Percentage of the population mainly using each fuel type
100%
75%
50%
25%
0%
1990
2000
2010
2020
2030
Fuel Type
Biomass
Charcoal
Coal
Kerosene
Gas
Electricity

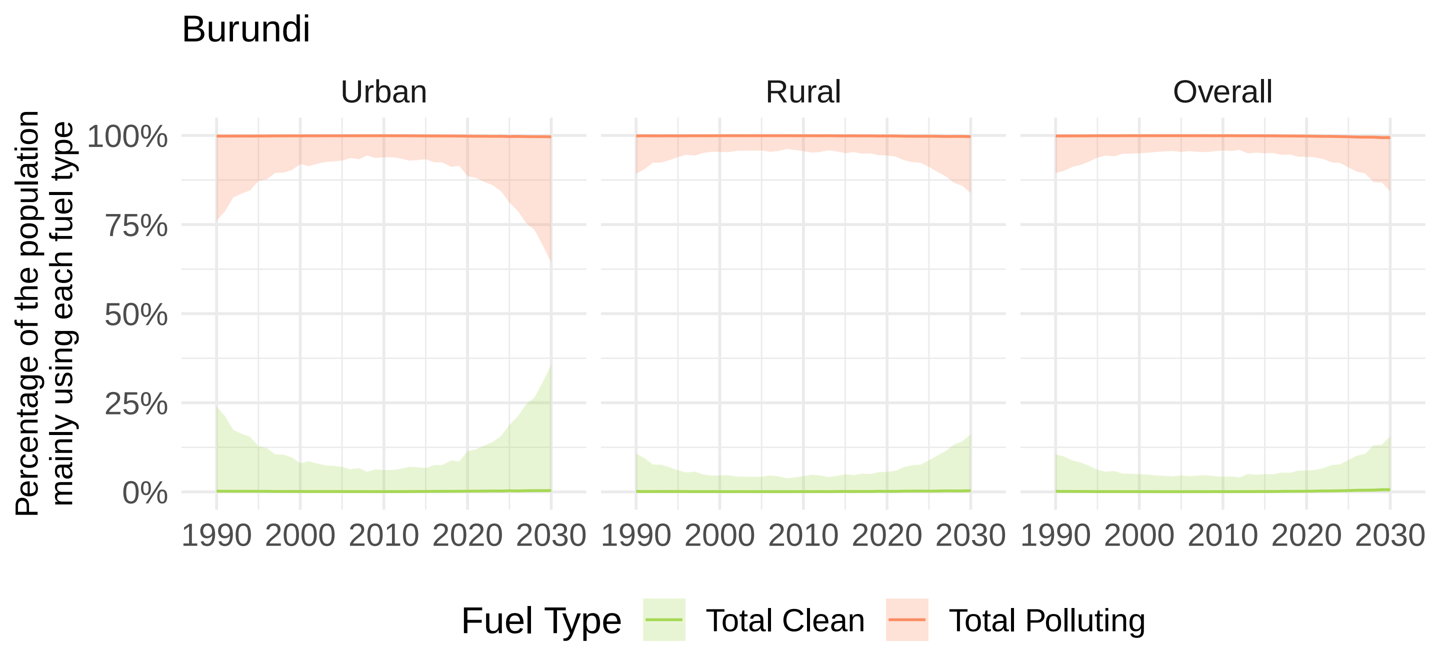

Burundi
Urban
Rural
Overall
Percentage of the population mainly using each fuel type
100%
75%
50%
25%
0%
1990
2000
2010
2020
2030
Fuel Type
Total Clean
Total Polluting

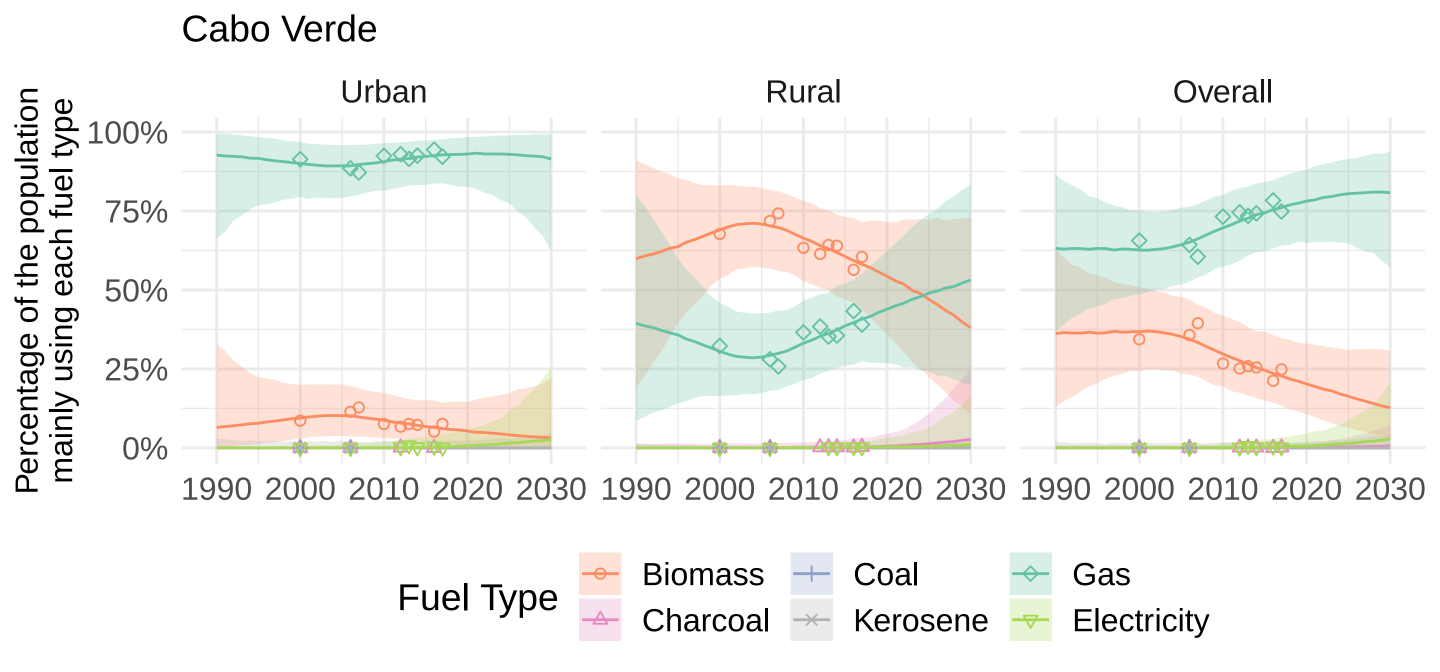
Cabo Verde
Urban
Rural
Overall
Percentage of the population mainly using each fuel type
100%
75%
50%
25%
0%
1990
2000
2010
2020
2030
Fuel Type
Biomass
Charcoal
Coal
Kerosene
Gas
Electricity

Cabo Verde

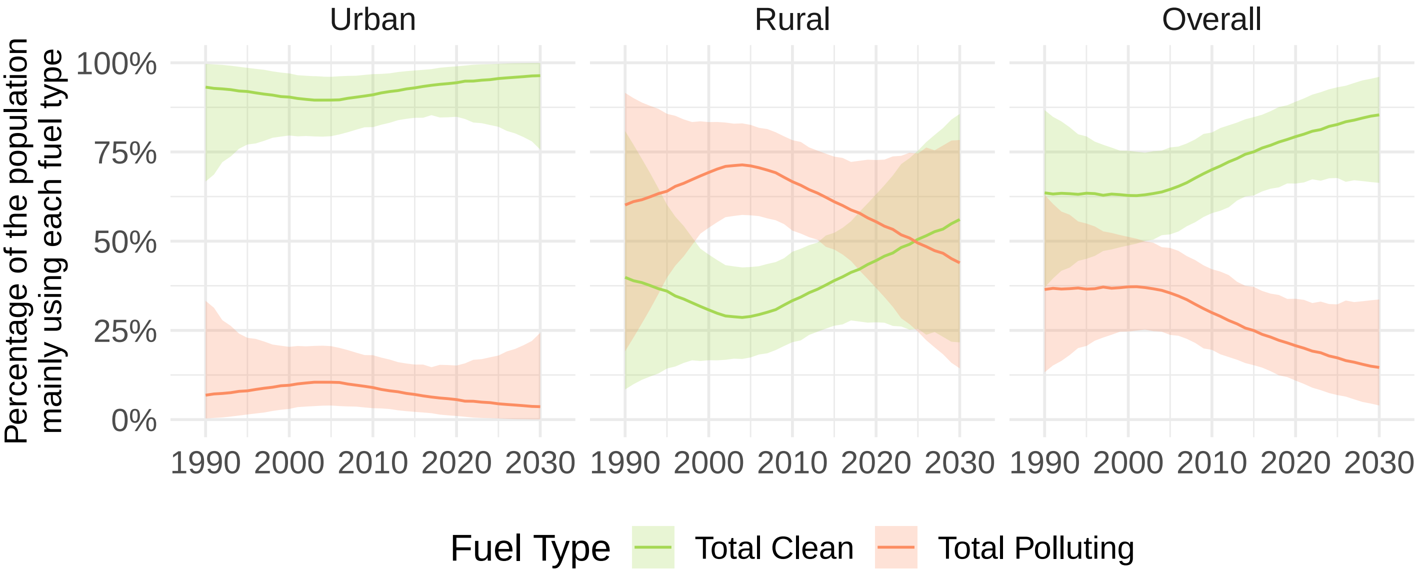
Urban
Rural
Overall
Percentage of the population mainly using each fuel type
100%
75%
50%
25%
0%
1990
2000
2010
2020
2030
Fuel Type
Total Clean
Total Polluting

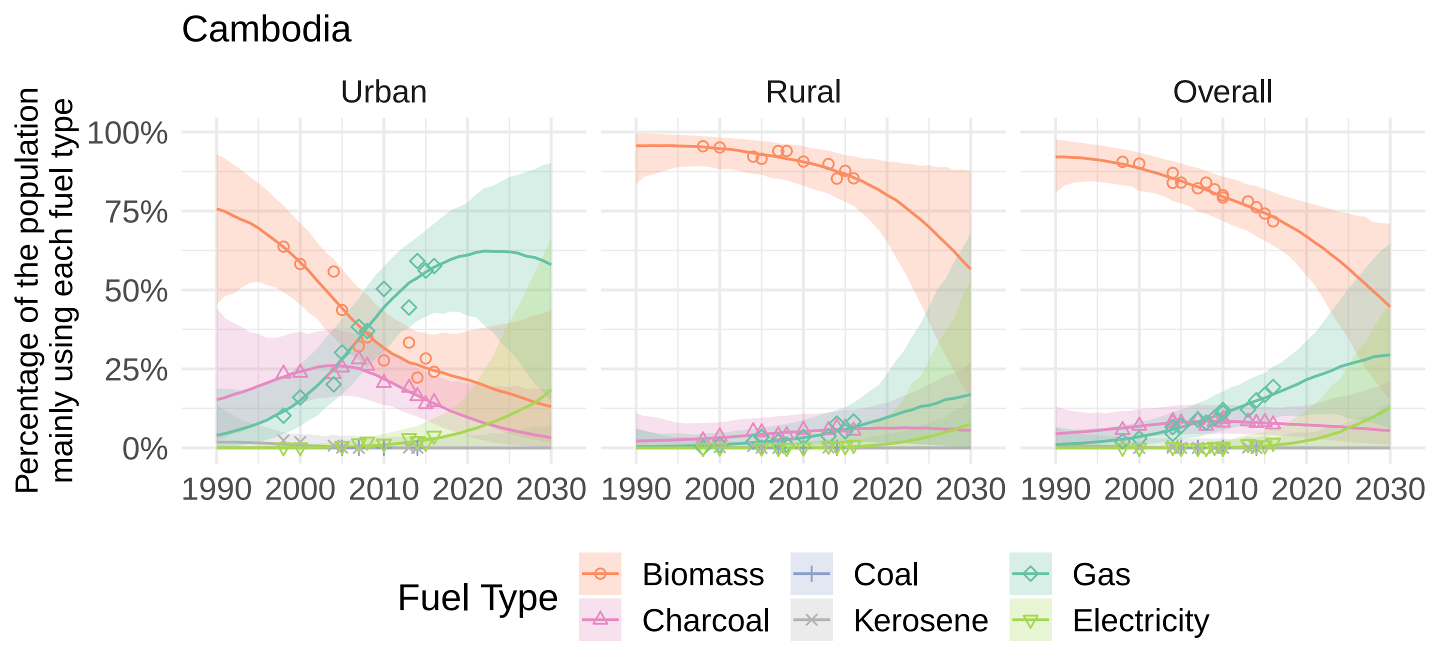

Cambodia
Urban
Rural
Overall
Percentage of the population mainly using each fuel type
100%
75%
50%
25%
0%
1990
2000
2010
2020
2030
Fuel Type
Biomass
Charcoal
Coal
Kerosene
Gas
Electricity

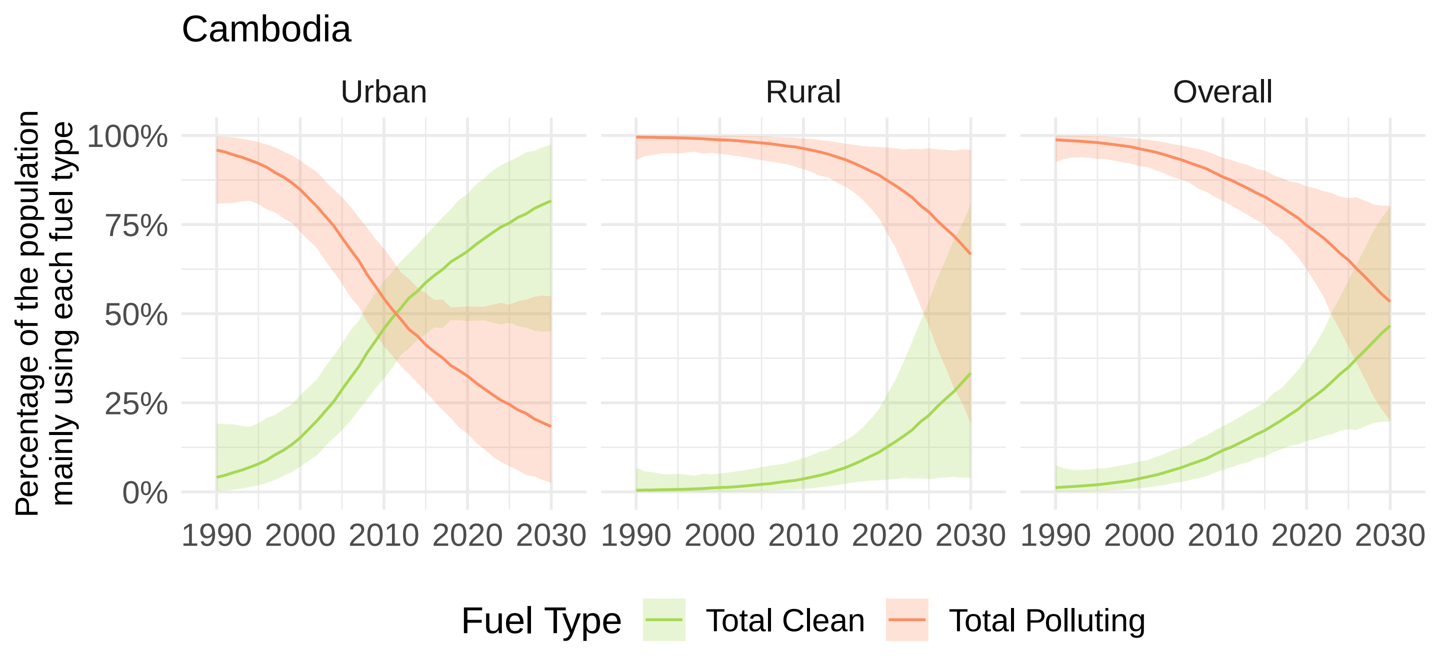
Cambodia
Urban
Rural
Overall
Percentage of the population mainly using each fuel type
100%
75%
50%
25%
0%
1990
2000
2010
2020
2030
Fuel Type
Total Clean
Total Polluting

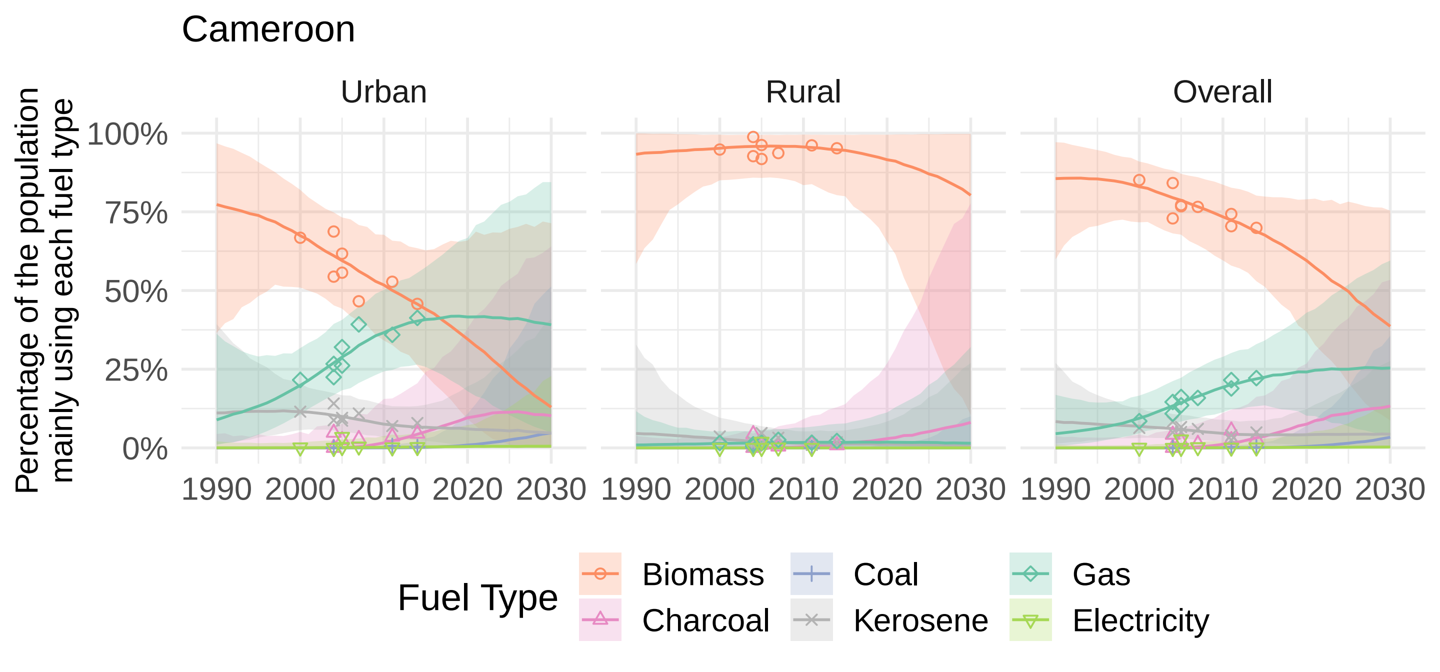
Cameroon
Urban
Rural
Overall
Percentage of the population mainly using each fuel type
100%
75%
50%
25%
0%
1990
2000
2010
2020
2030
Fuel Type
Biomass
Charcoal
Coal
Kerosene
Gas
Electricity

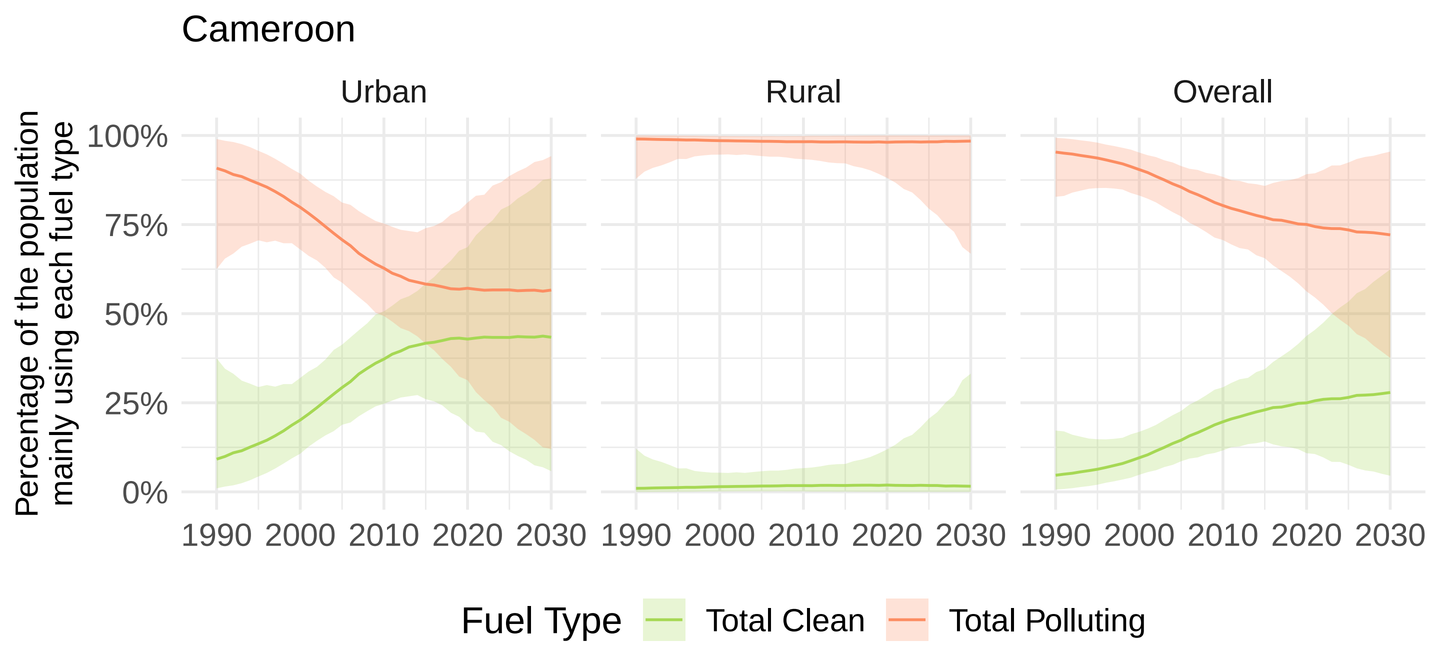

Cameroon
Urban
Rural
Overall
Percentage of the population mainly using each fuel type
100%
75%
50%
25%
0%
1990
2000
2010
2020
2030
Fuel Type
Total Clean
Total Polluting

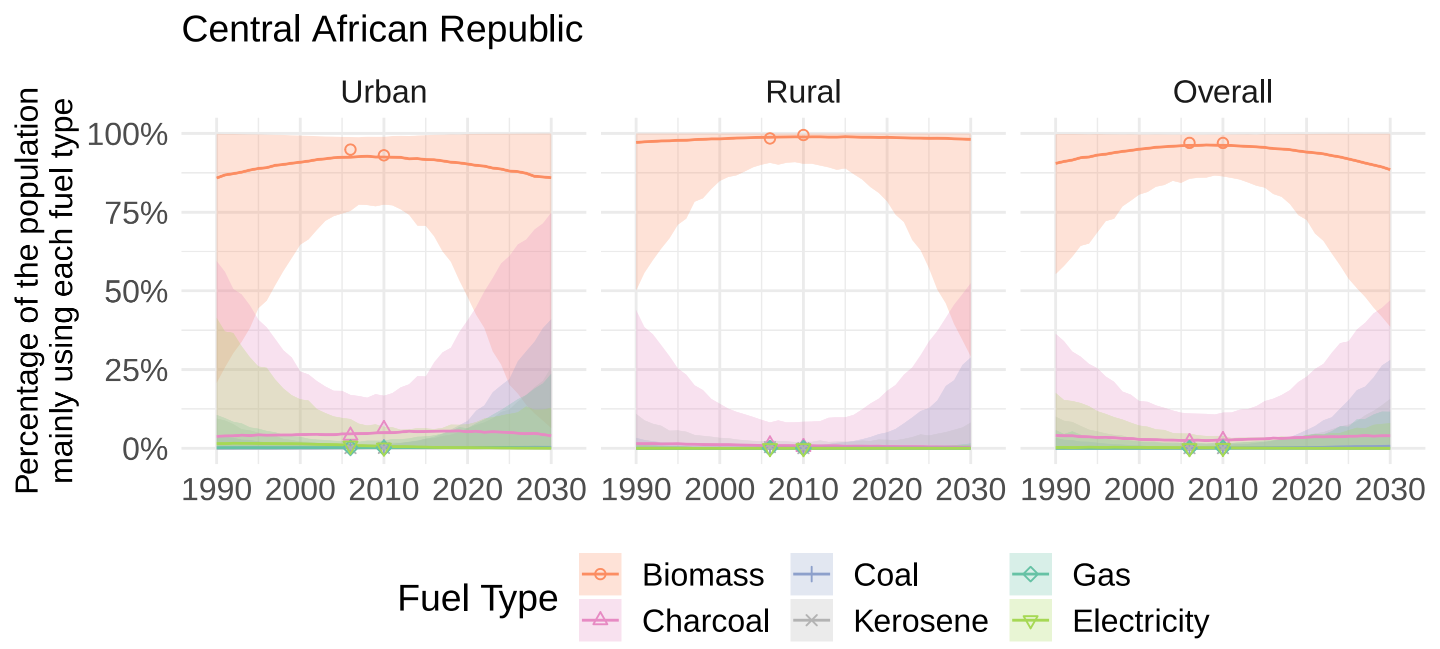

Central African Republic
Urban
Rural
Overall
Percentage of the population mainly using each fuel type
100%
75%
50%
25%
0%
1990
2000
2010
2020
2030
Fuel Type
Biomass
Charcoal
Coal
Kerosene
Gas
Electricity

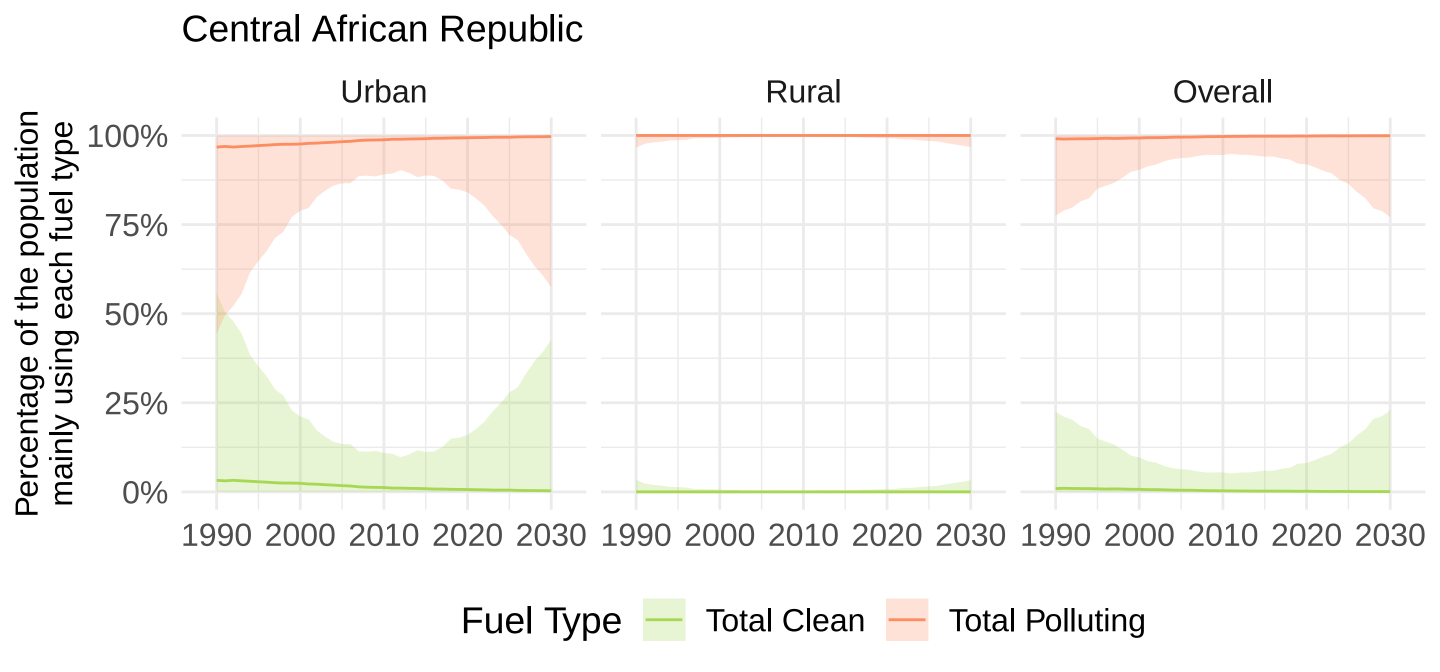

Central African Republic
Urban
Rural
Overall
Percentage of the population mainly using each fuel type
100%
75%
50%
25%
0%
1990
2000
2010
2020
2030
Fuel Type
Total Clean
Total Polluting

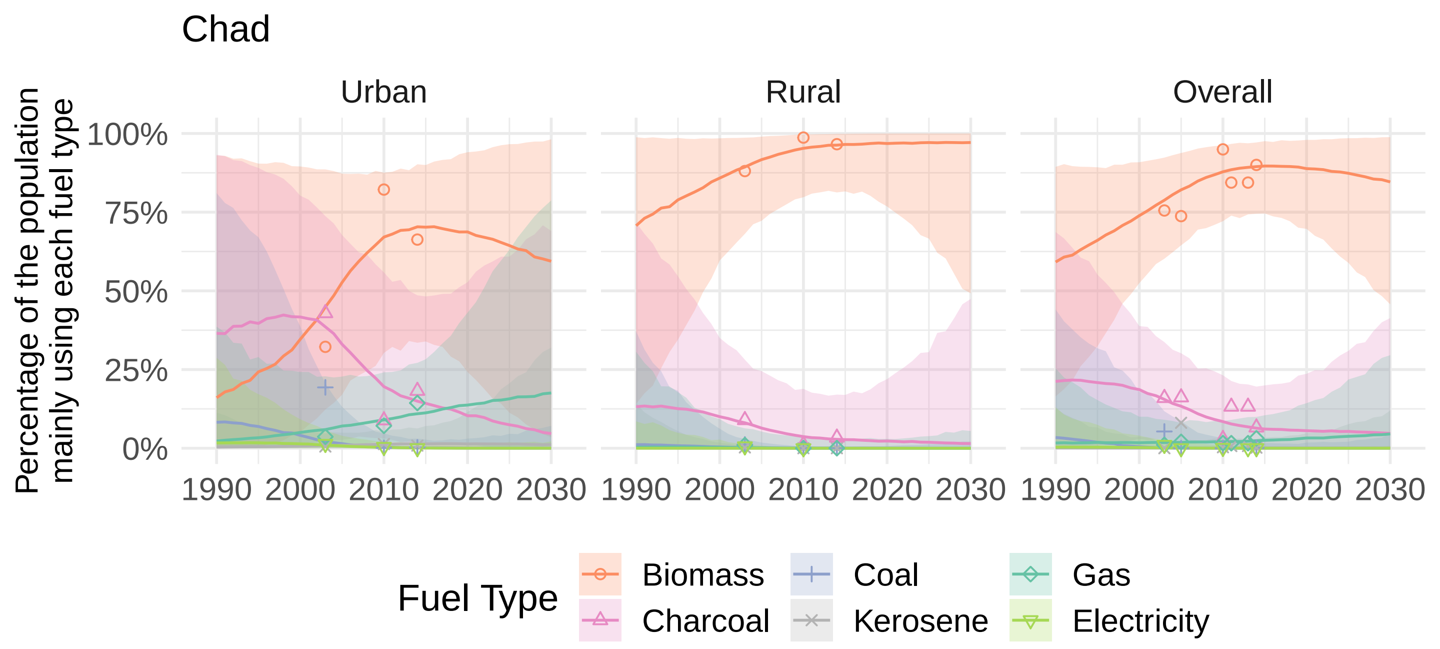
Chad
Urban
Rural
Overall
Percentage of the population mainly using each fuel type
100%
75%
50%
25%
0%
1990
2000
2010
2020
2030
Fuel Type
Biomass
Charcoal
Coal
Kerosene
Gas
Electricity

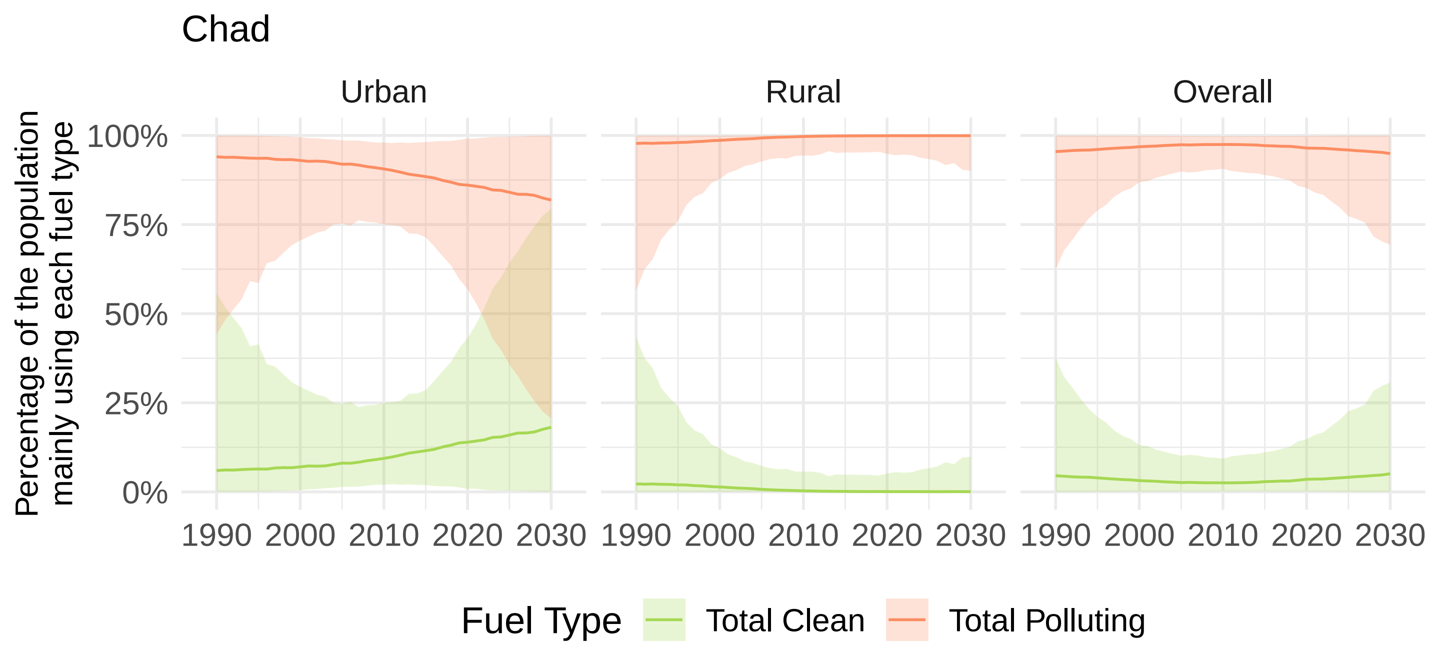

Chad
Urban
Rural
Overall
Percentage of the population mainly using each fuel type
100%
75%
50%
25%
0%
1990
2000
2010
2020
2030
Fuel Type
Total Clean
Total Polluting

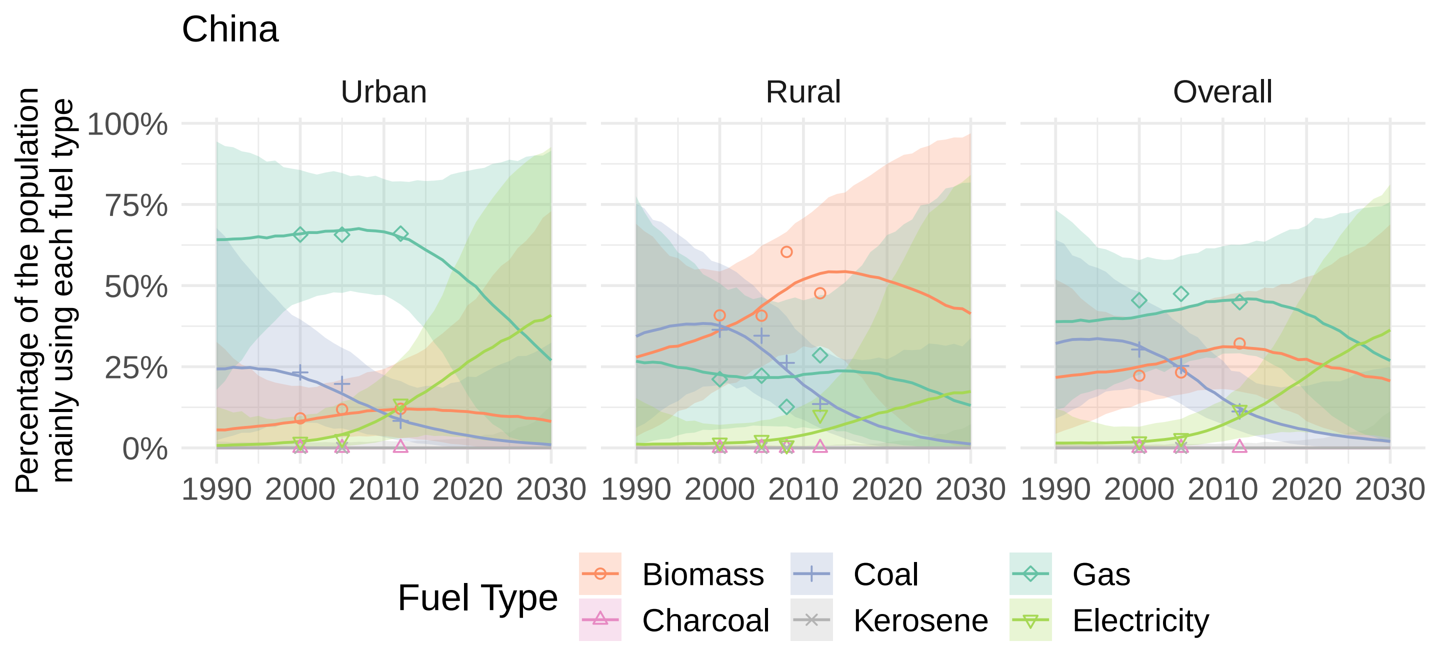

China
Urban
Rural
Overall
Percentage of the population mainly using each fuel type
100%
75%
50%
25%
0%
1990
2000
2010
2020
2030
Fuel Type
Biomass
Charcoal
Coal
Kerosene
Gas
Electricity

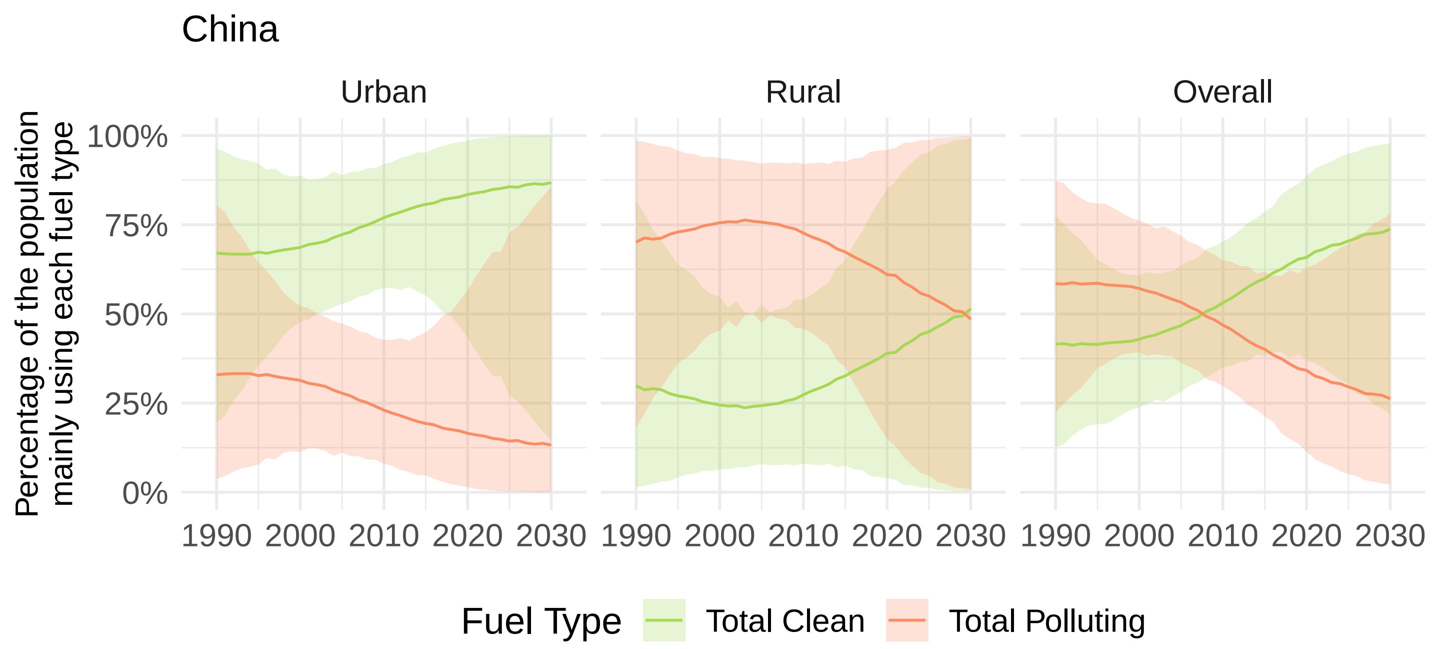

China
Urban
Rural
Overall
Percentage of the population mainly using each fuel type
100%
75%
50%
25%
0%
1990
2000
2010
2020
2030
Fuel Type
Total Clean
Total Polluting

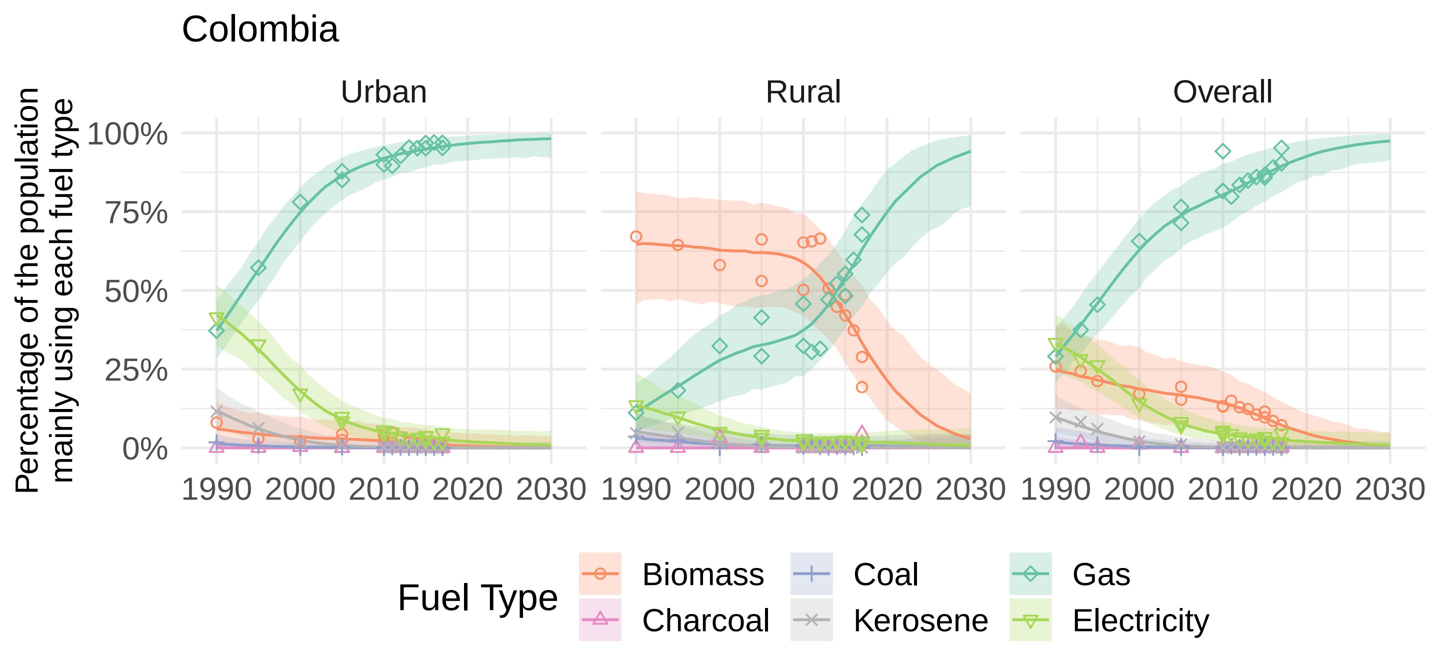

Colombia
Urban
Rural
Overall
Percentage of the population mainly using each fuel type
100%
75%
50%
25%
0%
1990
2000
2010
2020
2030
Fuel Type
Biomass
Charcoal
Coal
Kerosene
Gas
Electricity

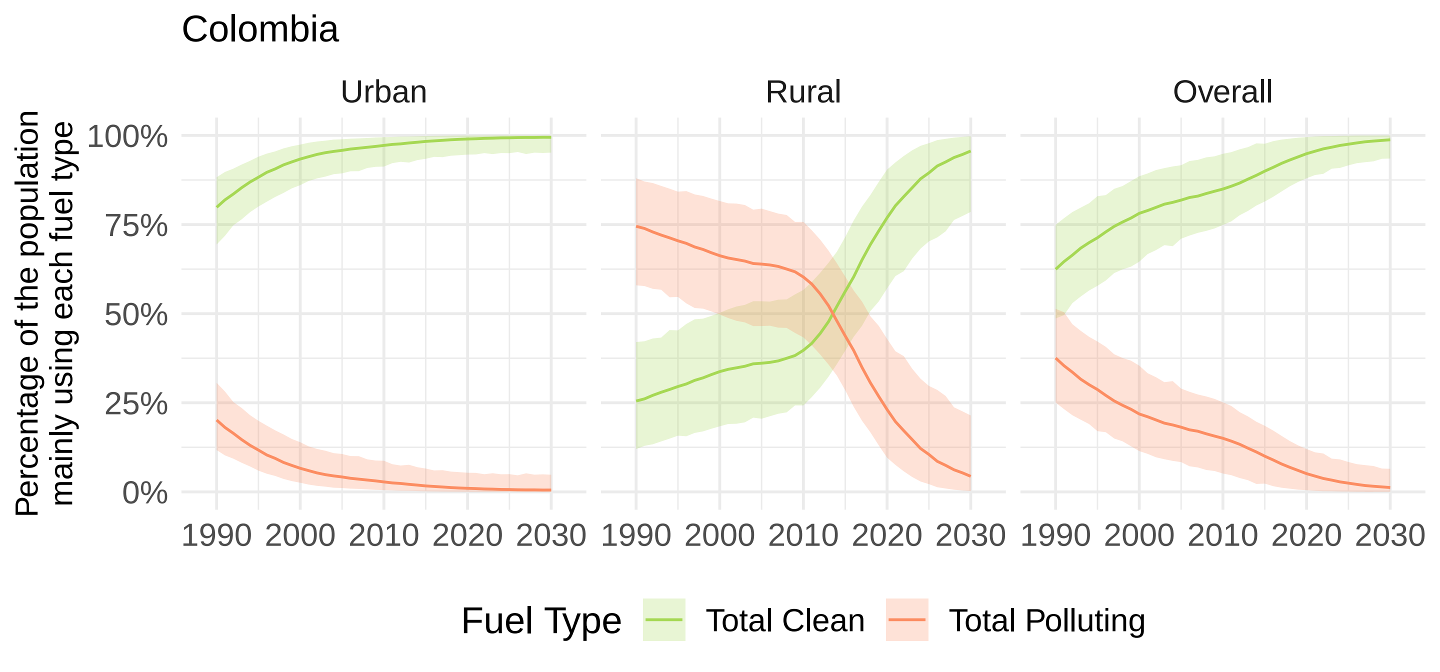

Colombia
Urban
Rural
Overall
Percentage of the population mainly using each fuel type
100%
75%
50%
25%
0%
1990
2000
2010
2020
2030
Fuel Type
Total Clean
Total Polluting

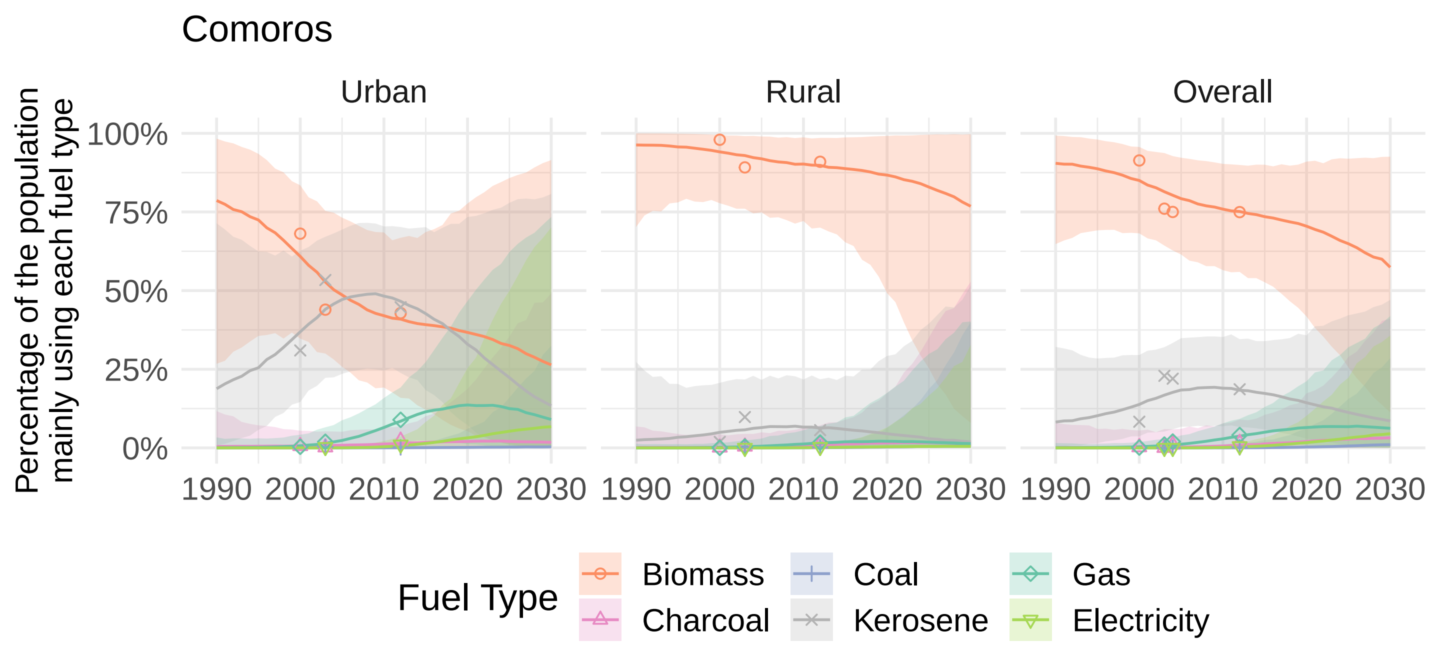
Comoros
Urban
Rural
Overall
Percentage of the population mainly using each fuel type
100%
75%
50%
25%
0%
1990
2000
2010
2020
2030
Fuel Type
Biomass
Charcoal
Coal
Kerosene
Gas
Electricity

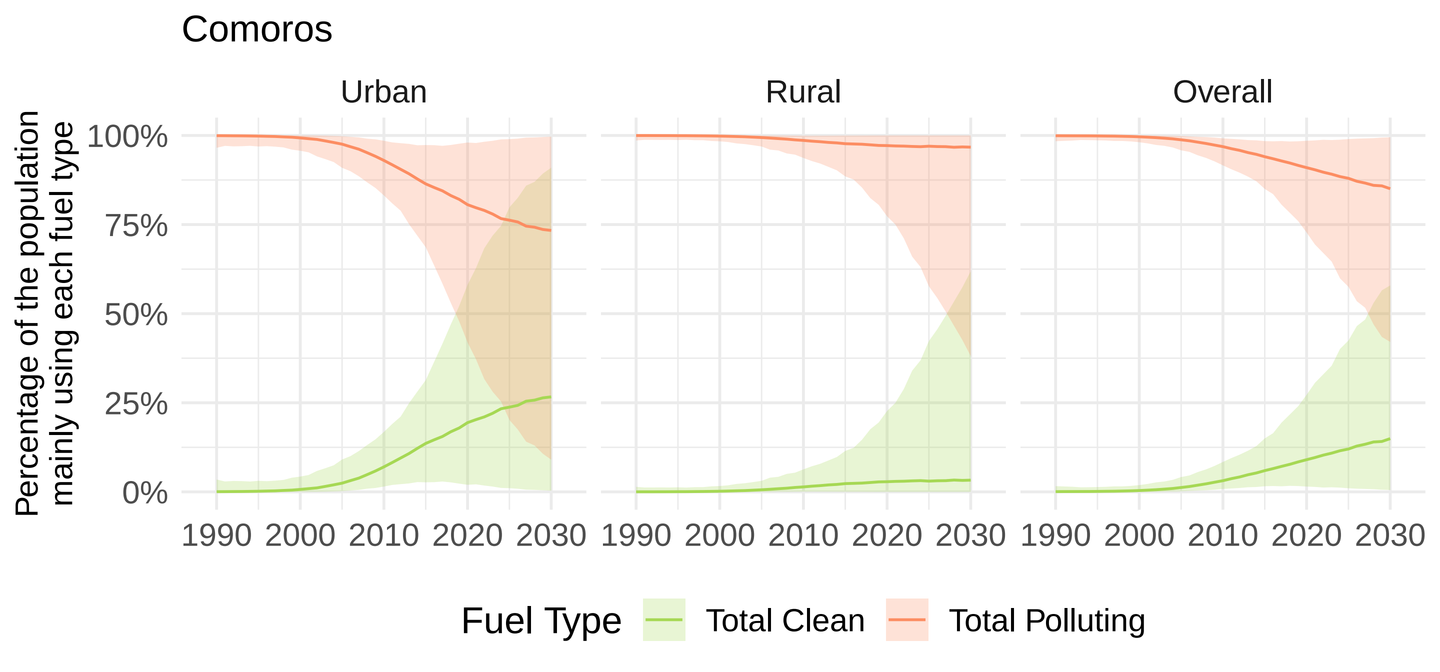
Comoros
Urban
Rural
Overall
Percentage of the population mainly using each fuel type
100%
75%
50%
25%
0%
1990
2000
2010
2020
2030
Fuel Type
Total Clean
Total Polluting

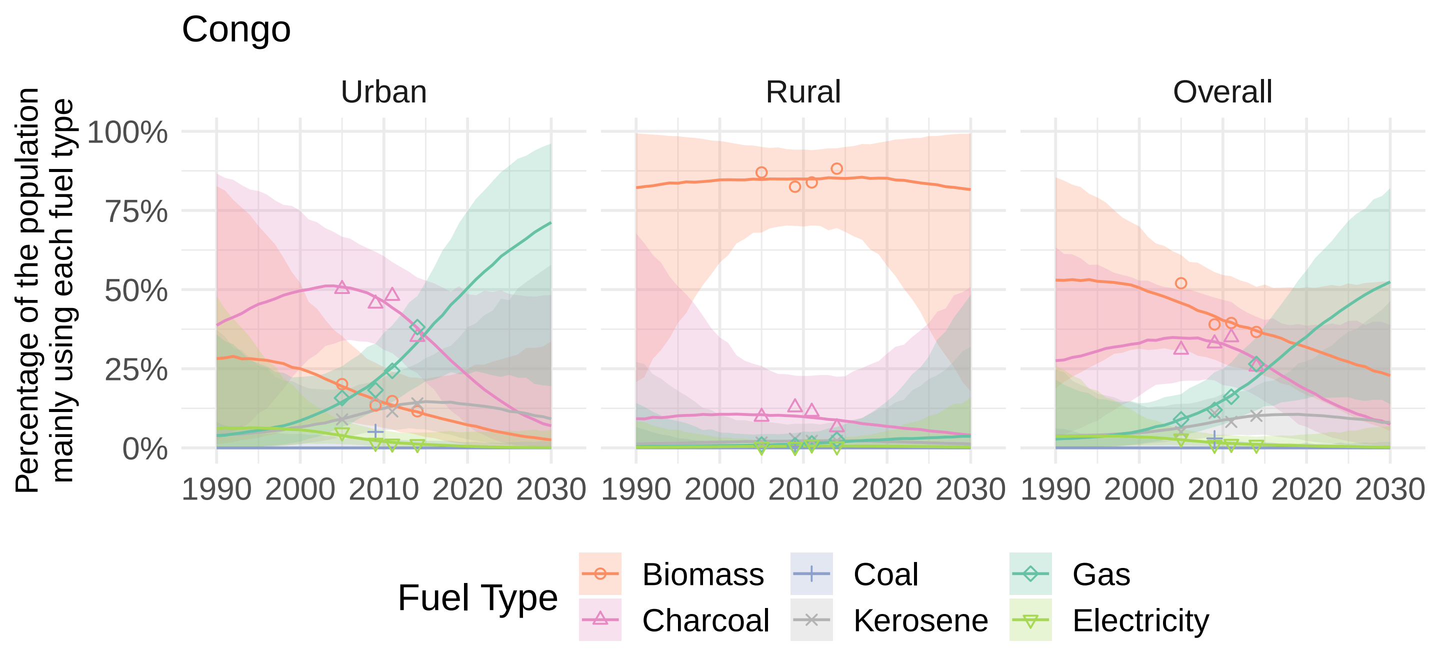
Congo
Urban
Rural
Overall
Percentage of the population mainly using each fuel type
100%
75%
50%
25%
0%
1990
2000
2010
2020
2030
Fuel Type
Biomass
Charcoal
Coal
Kerosene
Gas
Electricity

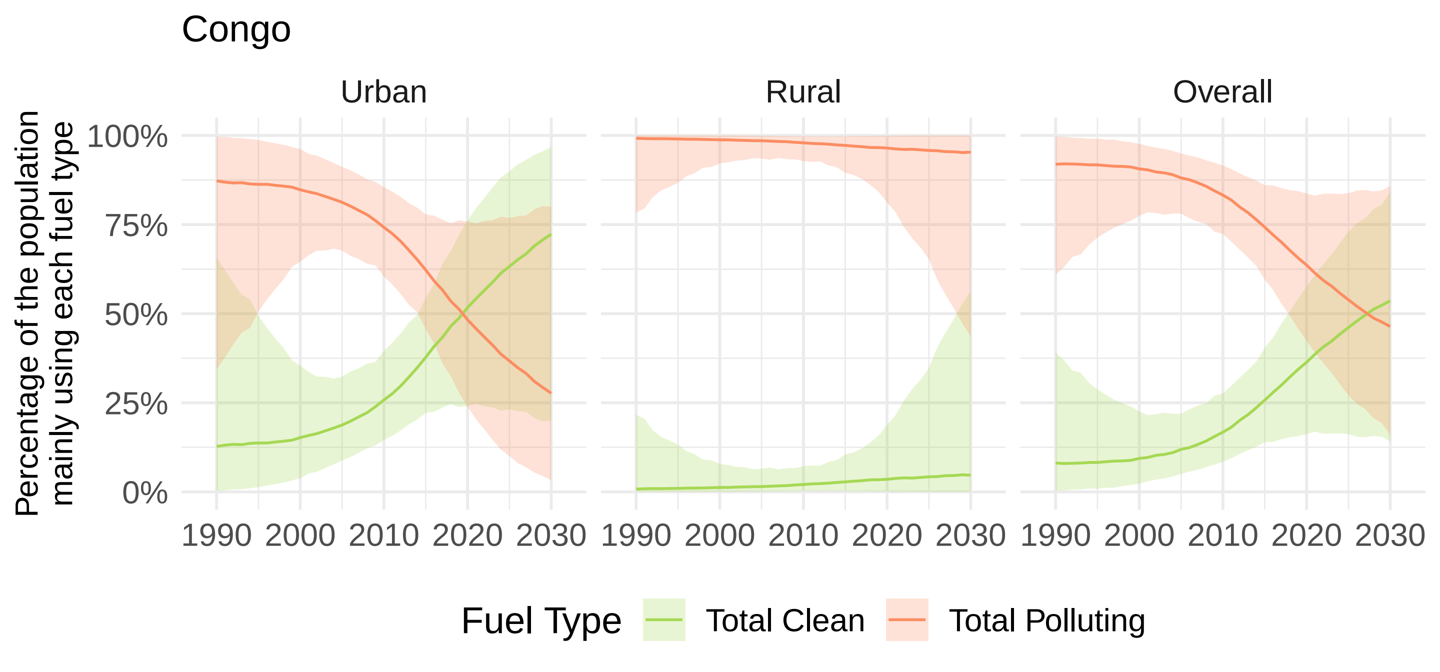

Congo
Urban
Rural
Overall
Percentage of the population mainly using each fuel type
100%
75%
50%
25%
0%
1990
2000
2010
2020
2030
Fuel Type
Total Clean
Total Polluting

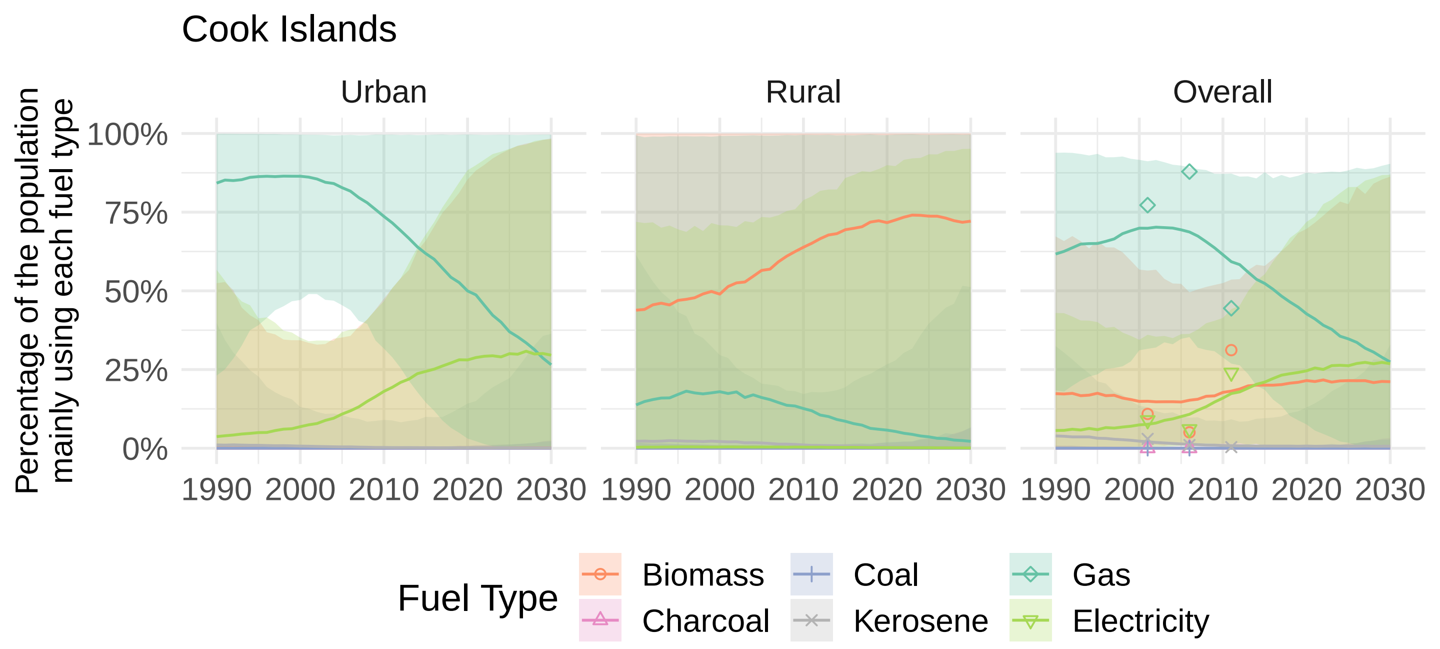
Cook Islands
Urban
Rural
Overall
Percentage of the population mainly using each fuel type
100%
75%
50%
25%
0%
1990
2000
2010
2020
2030
Fuel Type
Biomass
Charcoal
Coal
Kerosene
Gas
Electricity

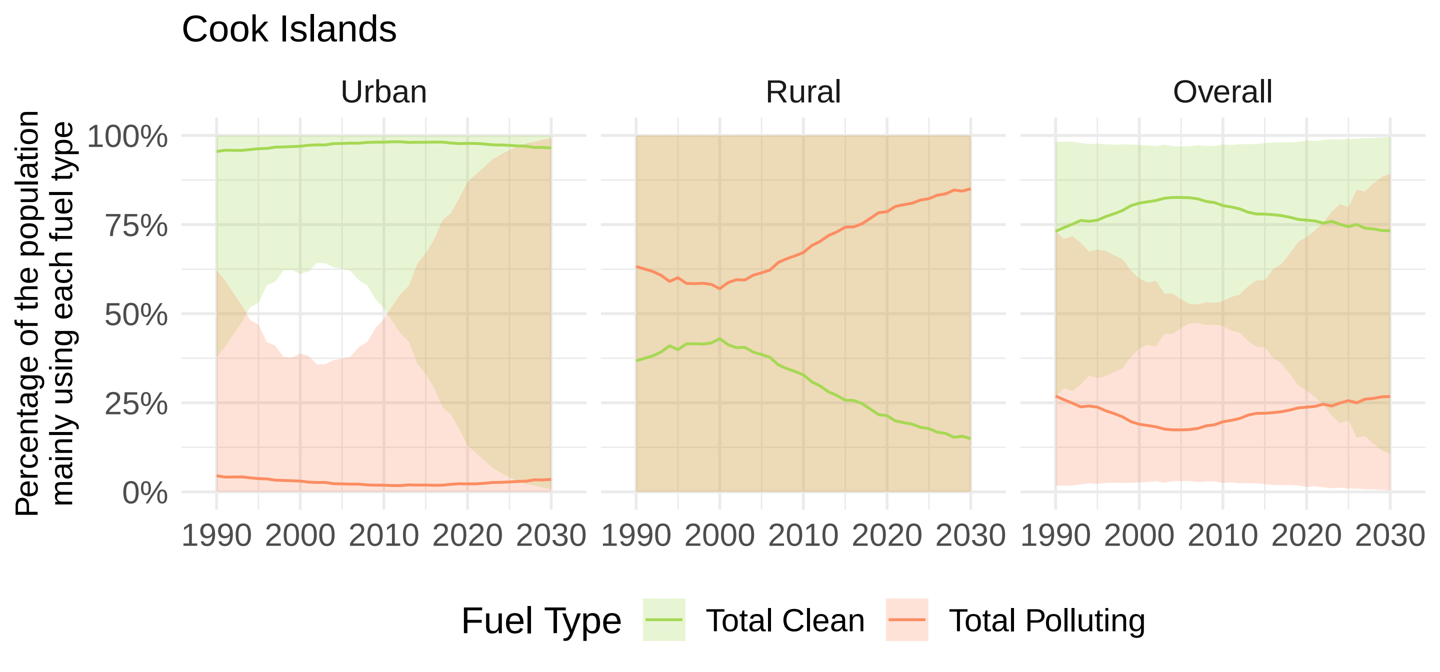

Cook Islands
Urban
Rural
Overall
Percentage of the population mainly using each fuel type
100%
75%
50%
25%
0%
1990
2000
2010
2020
2030
Fuel Type
Total Clean
Total Polluting

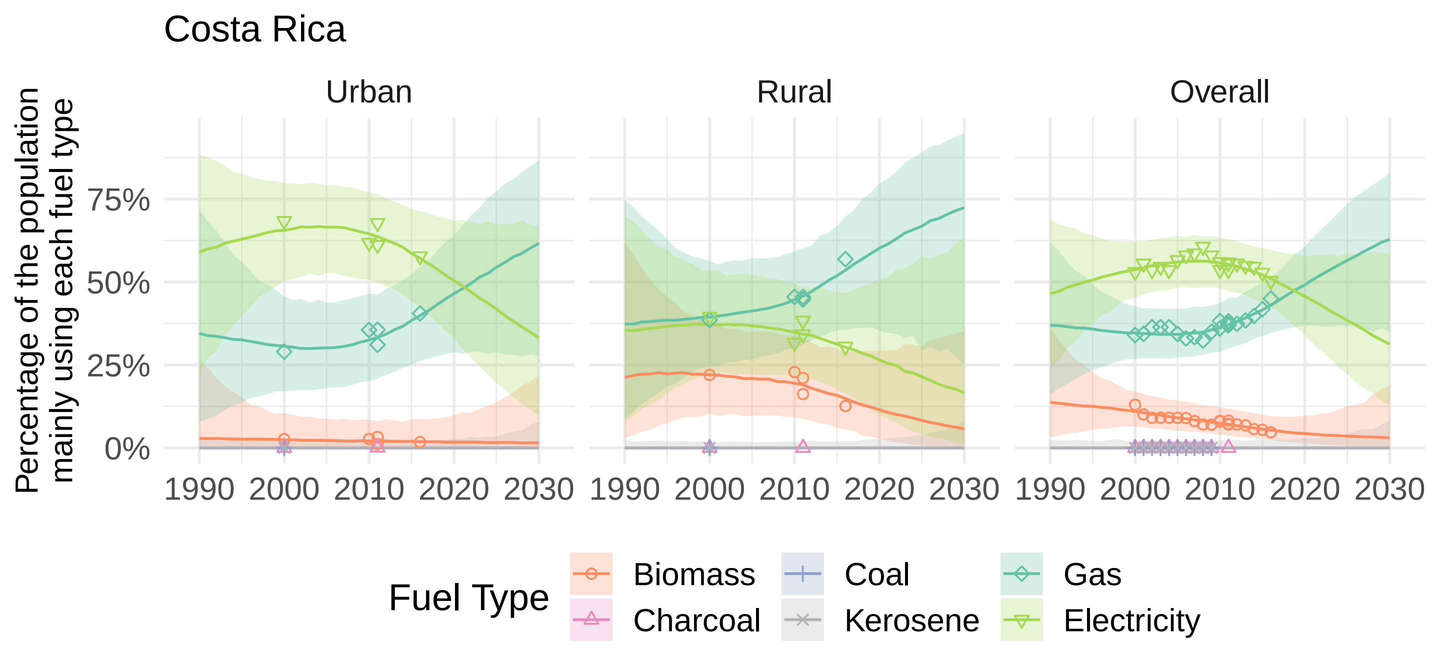
Costa Rica
Urban
Rural
Overall
Percentage of the population mainly using each fuel type
0%
25%
50%
75%
1990
2000
2010
2020
2030
Fuel Type
Biomass
Charcoal
Coal
Kerosene
Gas
Electricity

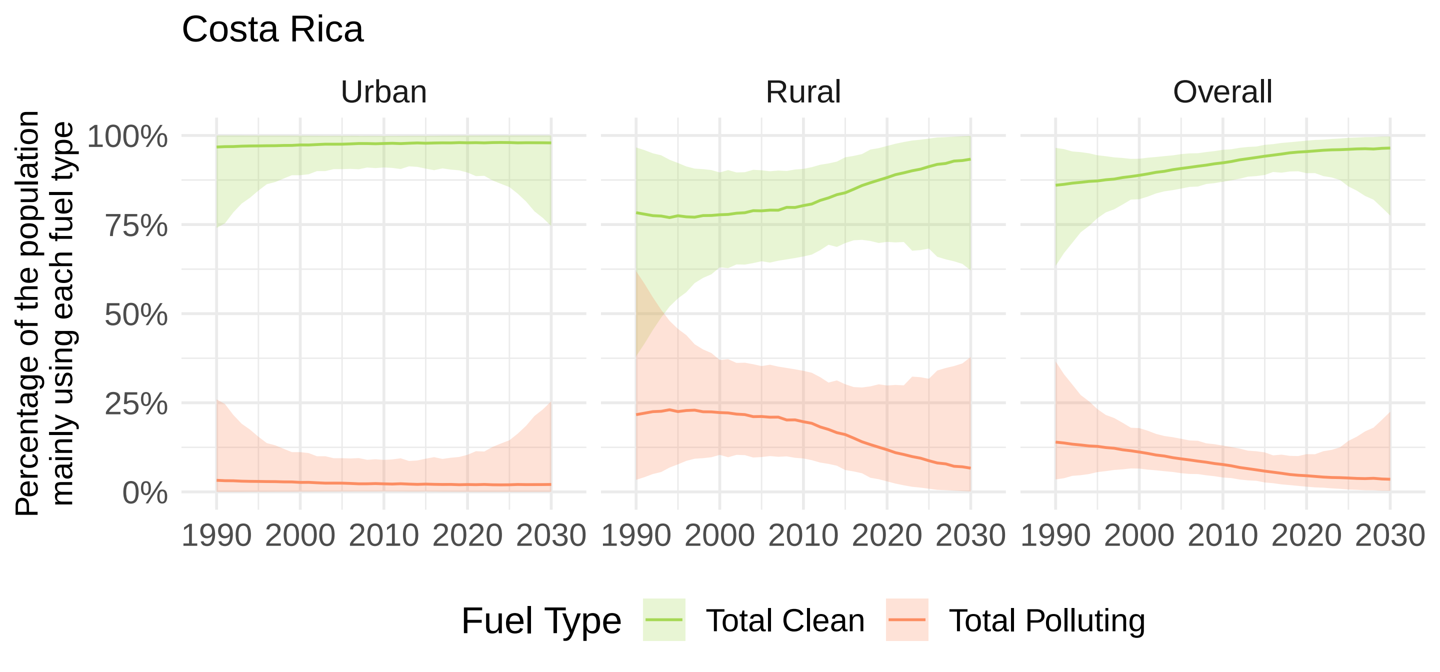
Costa Rica
Urban
Rural
Overall
Percentage of the population mainly using each fuel type
100%
75%
50%
25%
0%
1990
2000
2010
2020
2030
Fuel Type
Total Clean
Total Polluting

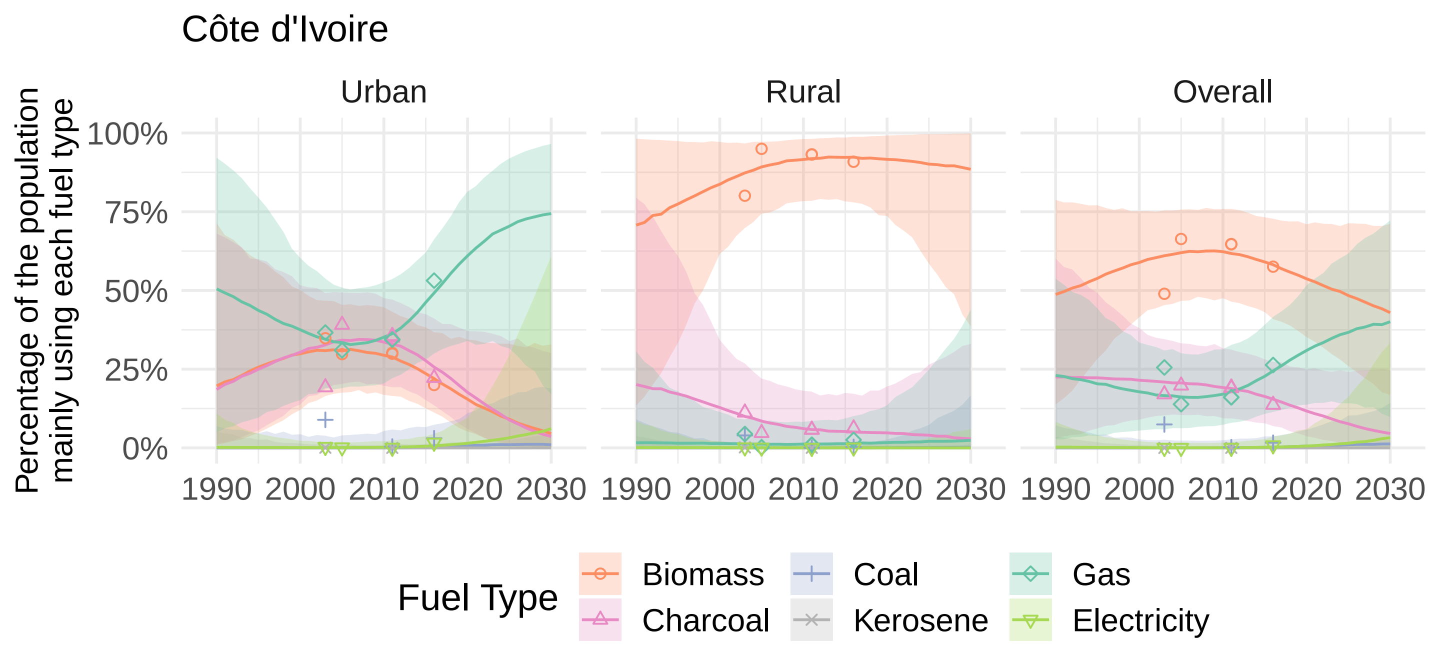

Côte d'Ivoire
Urban
Rural
Overall
Percentage of the population mainly using each fuel type
100%
75%
50%
25%
0%
1990
2000
2010
2020
2030
Fuel Type
Biomass
Charcoal
Coal
Kerosene
Gas
Electricity

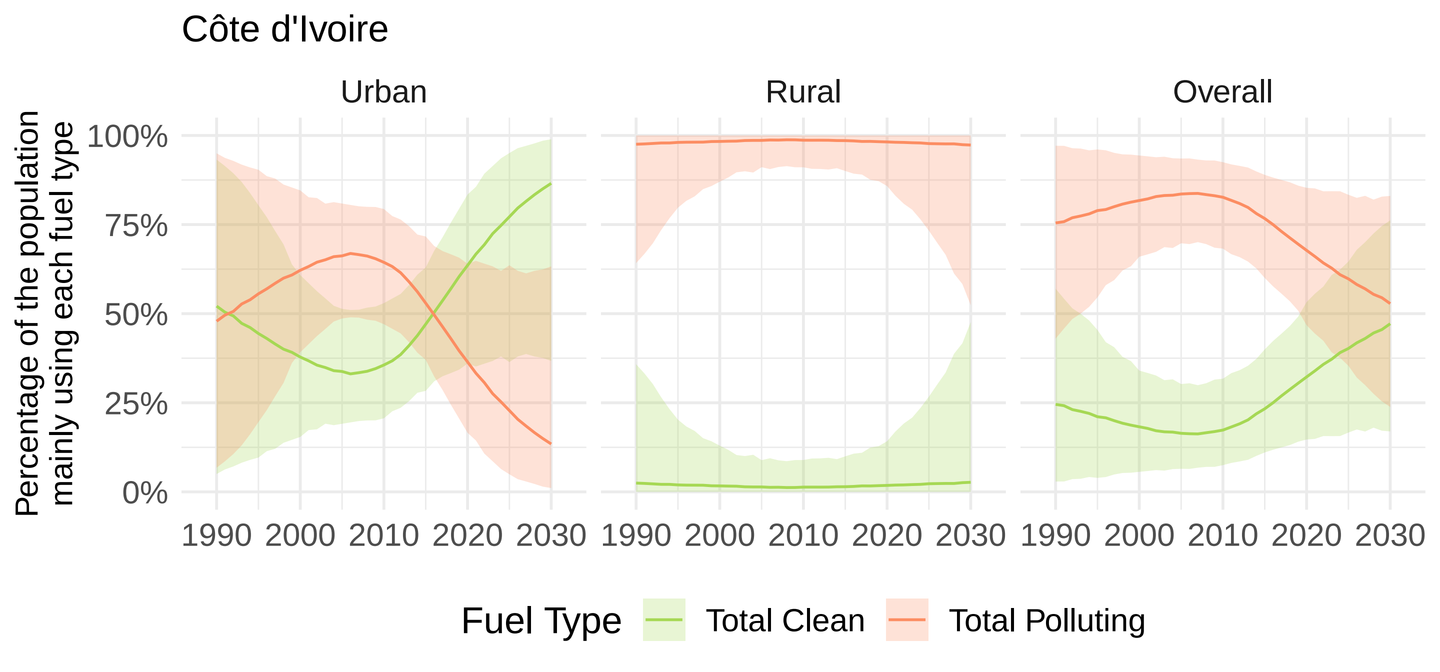
Côte d'Ivoire
Urban
Rural
Overall
Percentage of the population mainly using each fuel type
100%
75%
50%
25%
0%
1990
2000
2010
2020
2030
Fuel Type
Total Clean
Total Polluting

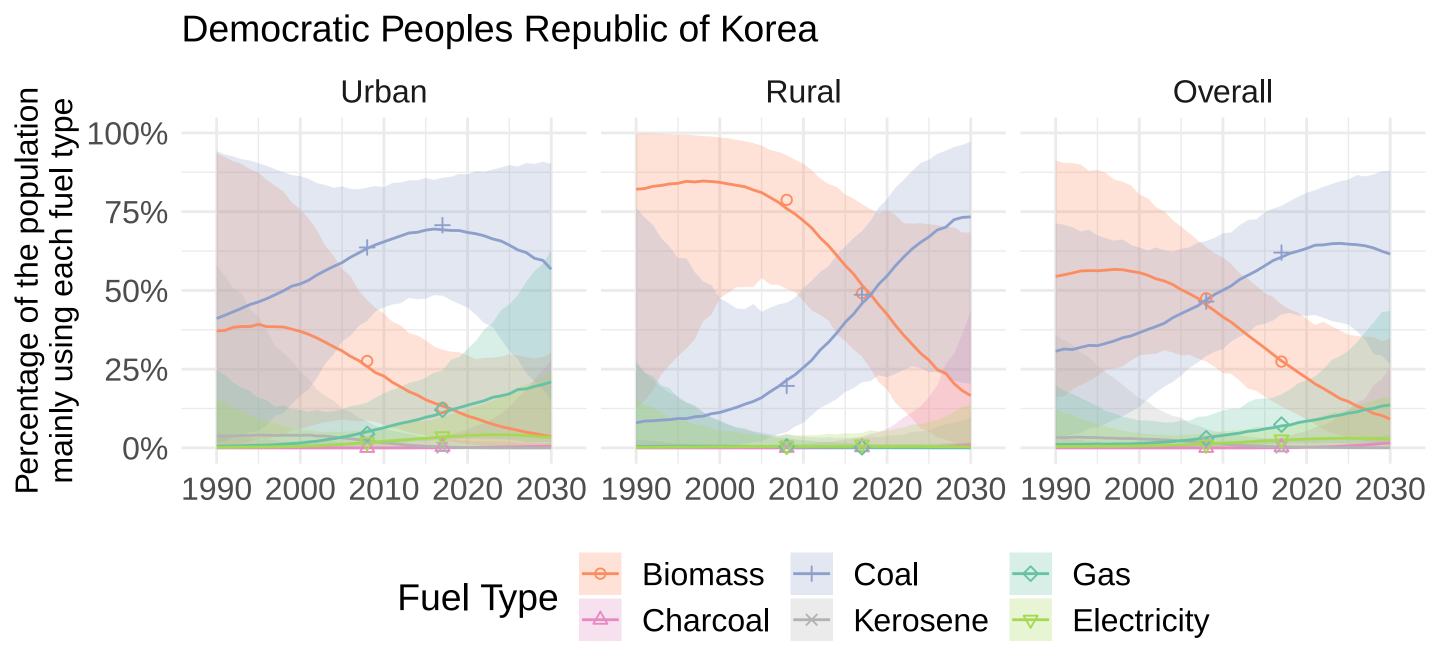
Democratic Peoples Republic of Korea
Urban
Rural
Overall
Percentage of the population mainly using each fuel type
100%
75%
50%
25%
0%
1990
2000
2010
2020
2030
Fuel Type
Biomass
Coal
Gas
Charcoal
Kerosene
Electricity

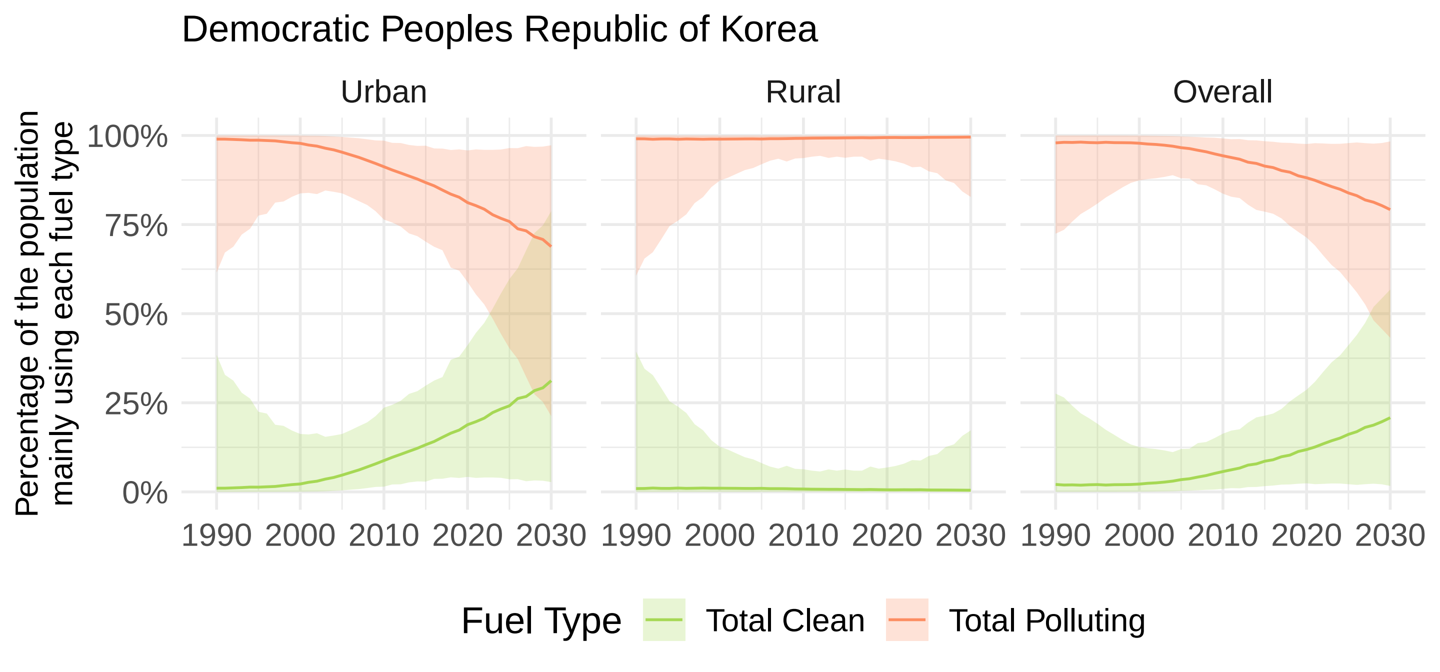
Democratic Peoples Republic of Korea
Urban
Rural
Overall
Percentage of the population mainly using each fuel type
100%
75%
50%
25%
0%
1990
2000
2010
2020
2030
Fuel Type
Total Clean
Total Polluting

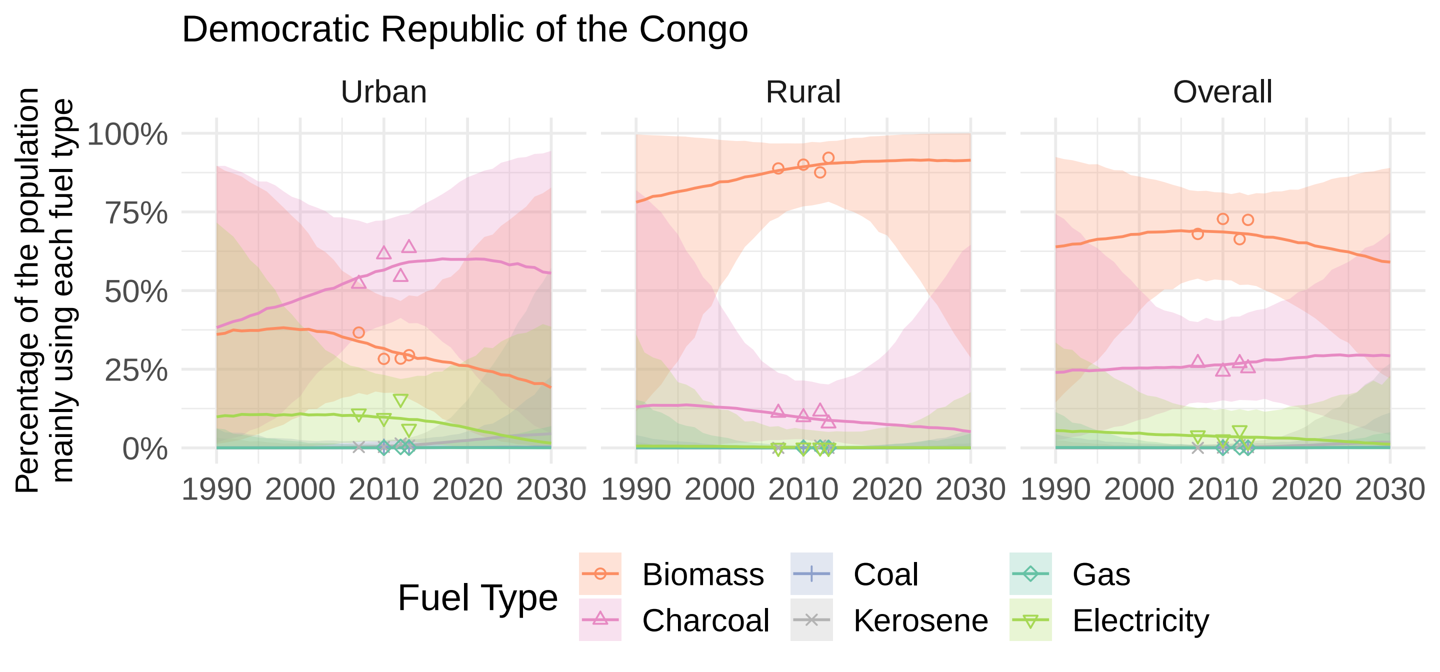

Democratic Republic of the Congo
Urban
Rural
Overall
Percentage of the population mainly using each fuel type
100%
75%
50%
25%
0%
1990
2000
2010
2020
2030
Fuel Type
Biomass
Charcoal
Coal
Kerosene
Gas
Electricity

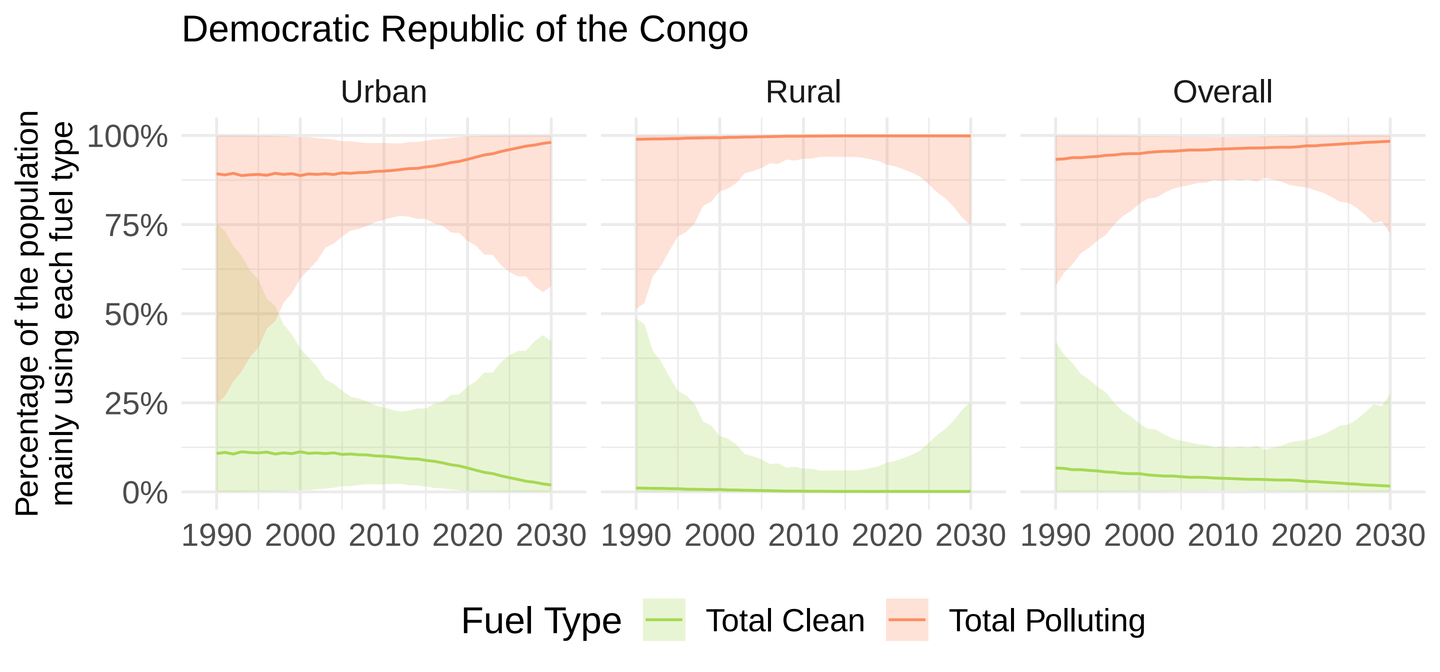

Democratic Republic of the Congo
Urban
Rural
Overall
Percentage of the population mainly using each fuel type
100%
75%
50%
25%
0%
1990
2000
2010
2020
2030
Fuel Type
Total Clean
Total Polluting

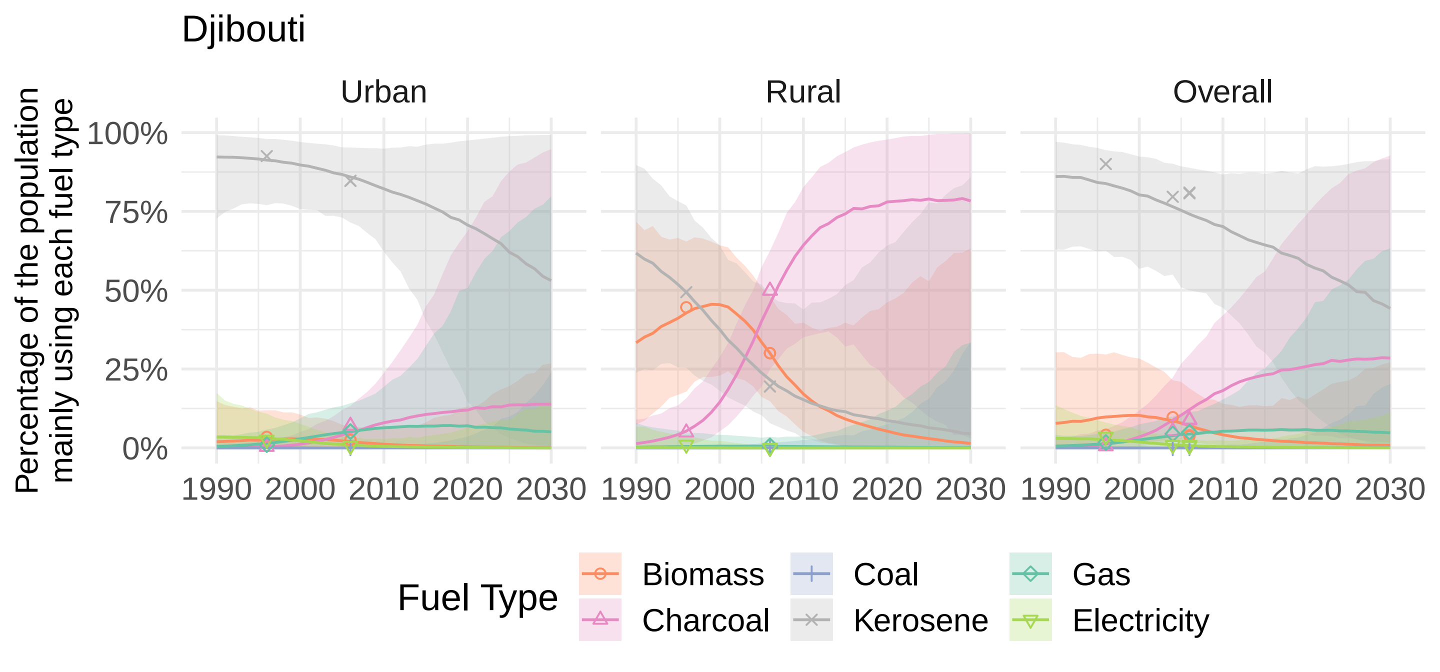

Djibouti
Urban
Rural
Overall
Percentage of the population mainly using each fuel type
100%
75%
50%
25%
0%
1990
2000
2010
2020
2030
Fuel Type
Biomass
Charcoal
Coal
Kerosene
Gas
Electricity

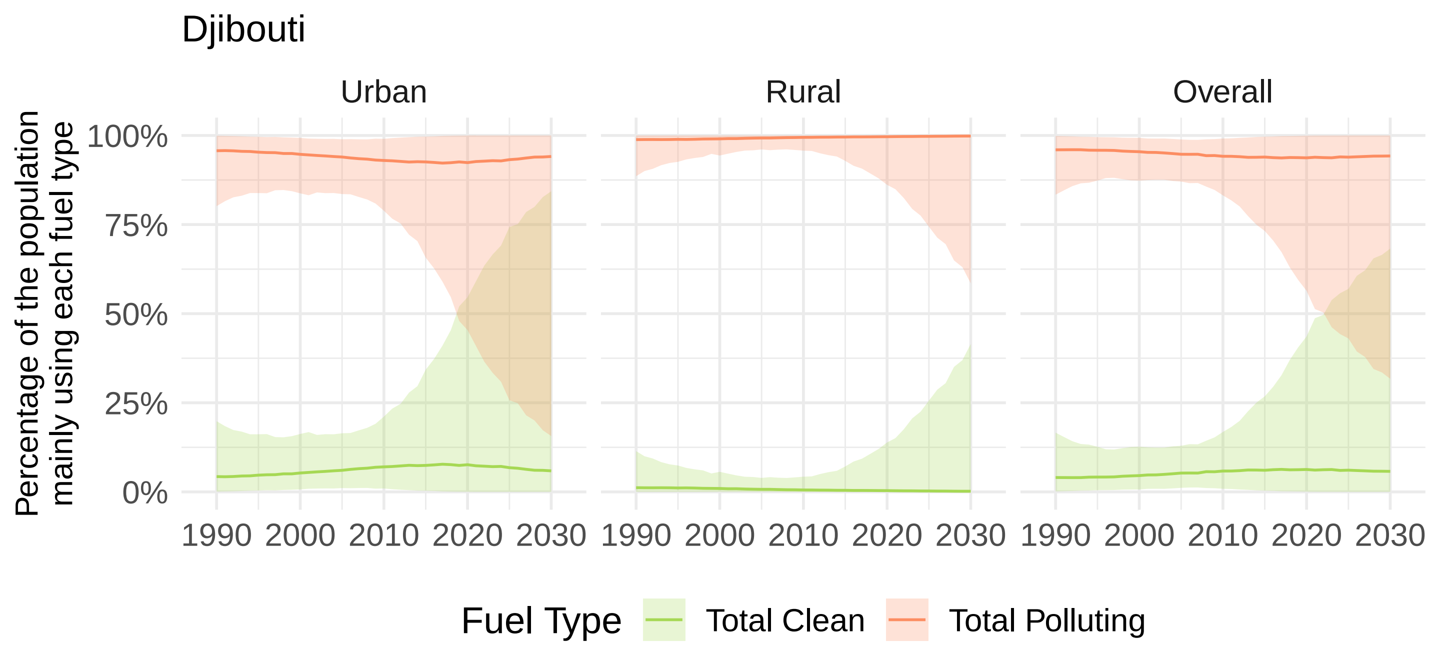

Djibouti
Urban
Rural
Overall
Percentage of the population mainly using each fuel type
100%
75%
50%
25%
0%
1990
2000
2010
2020
2030
Fuel Type
Total Clean
Total Polluting

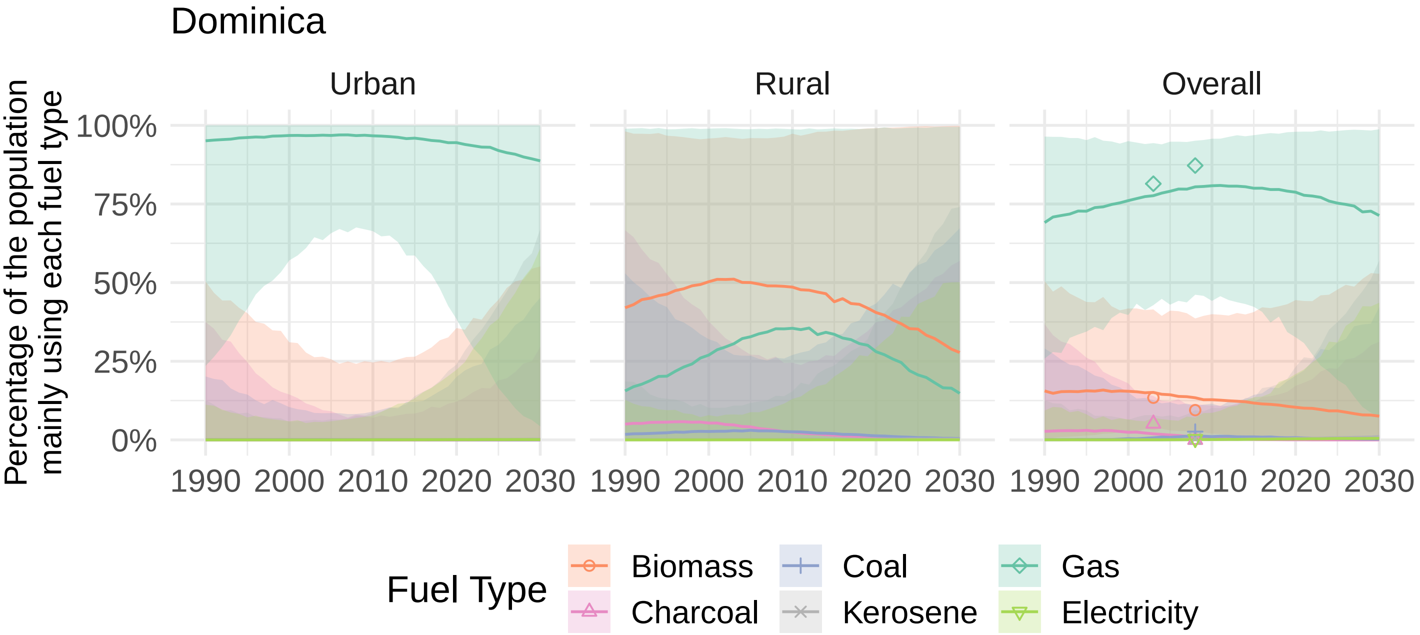
Dominica
Urban
Rural
Overall
Percentage of the population mainly using each fuel type
100%
75%
50%
25%
0%
1990
2000
2010
2020
2030
Fuel Type
Biomass
Charcoal
Coal
Kerosene
Gas
Electricity

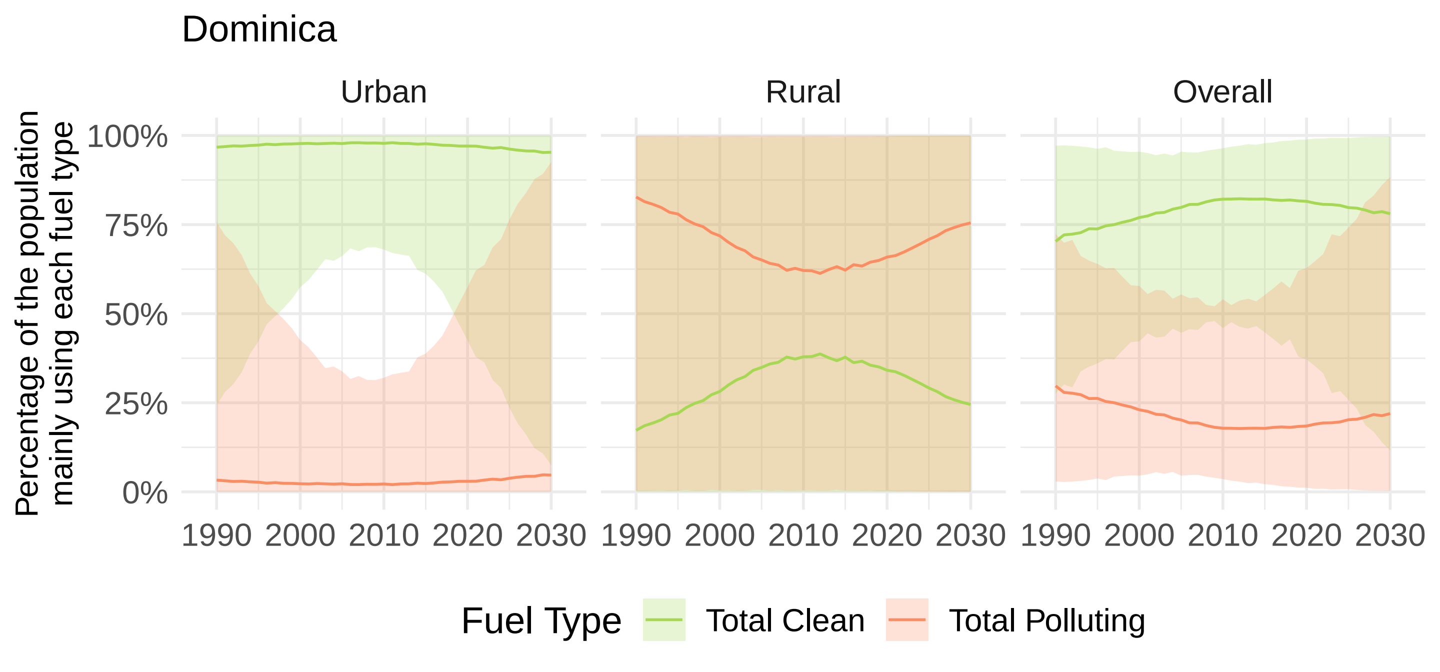
Dominica
Urban
Rural
Overall
Percentage of the population mainly using each fuel type
100%
75%
50%
25%
0%
1990
2000
2010
2020
2030
Fuel Type
Total Clean
Total Polluting

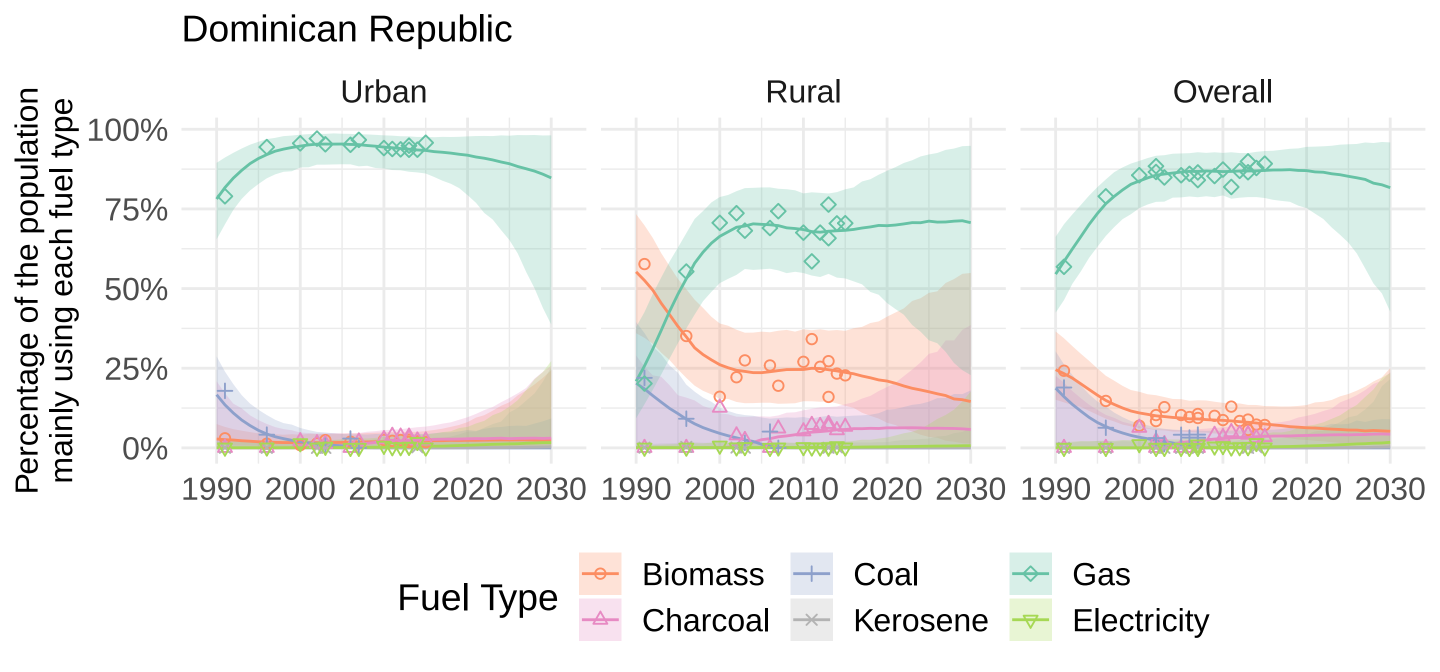

Dominican Republic
Urban
Rural
Overall
Percentage of the population mainly using each fuel type
100%
75%
50%
25%
0%
1990
2000
2010
2020
2030
Fuel Type
Biomass
Charcoal
Coal
Kerosene
Gas
Electricity

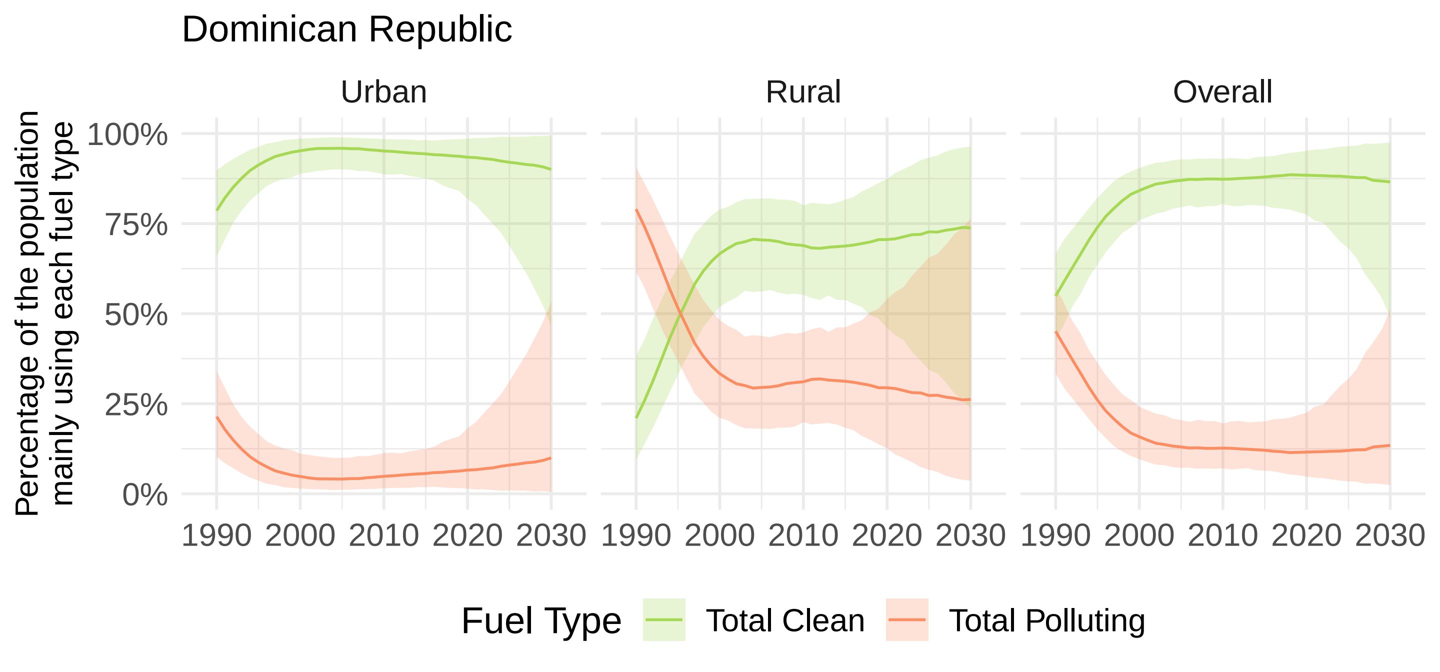

Dominican Republic
Urban
Rural
Overall
Percentage of the population mainly using each fuel type
100%
75%
50%
25%
0%
1990
2000
2010
2020
2030
Fuel Type
Total Clean
Total Polluting

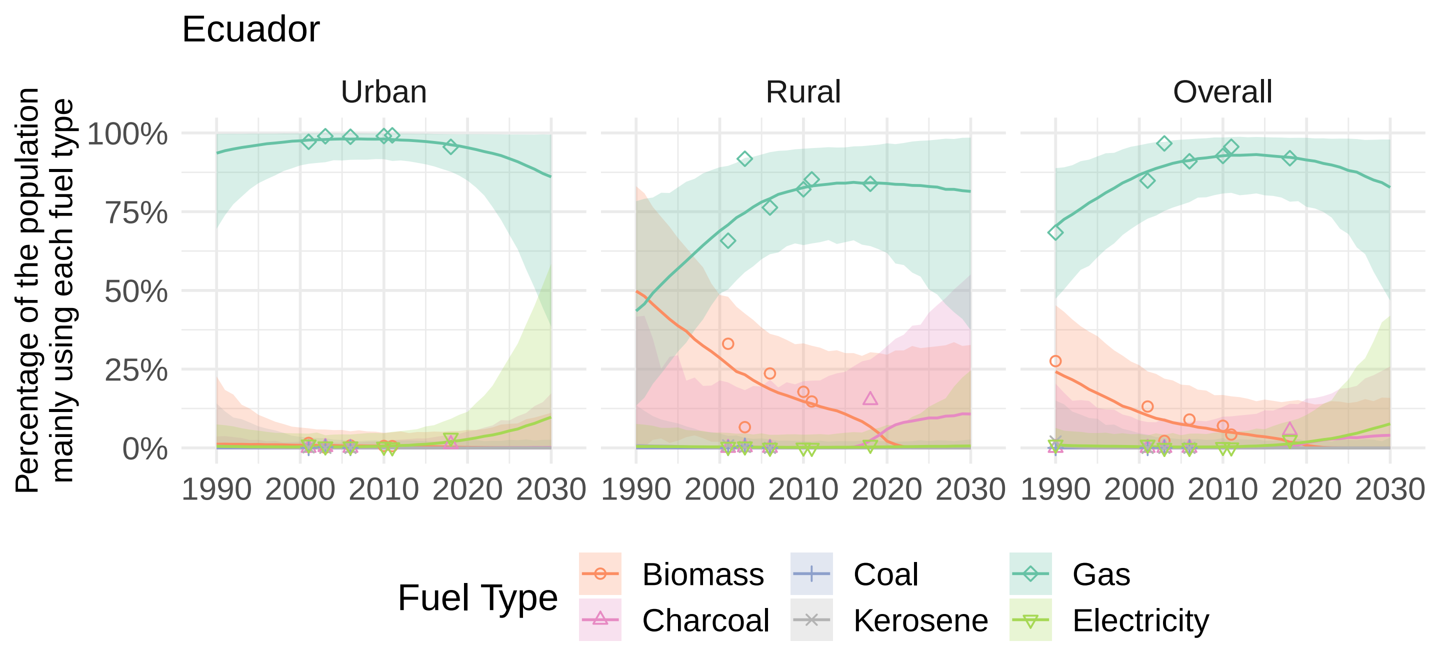
Ecuador
Urban
Rural
Overall
Percentage of the population mainly using each fuel type
100%
75%
50%
25%
0%
1990
2000
2010
2020
2030
Fuel Type
Biomass
Charcoal
Coal
Kerosene
Gas
Electricity

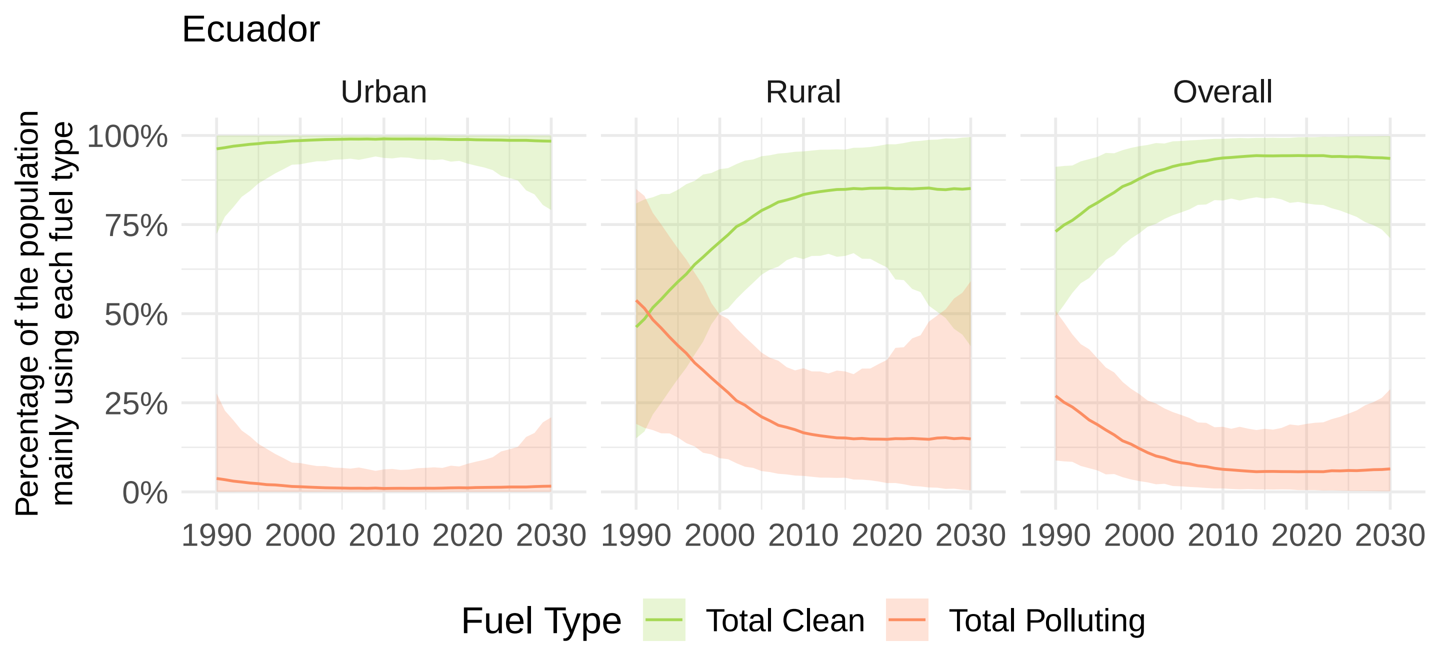

Ecuador
Urban
Rural
Overall
Percentage of the population mainly using each fuel type
100%
75%
50%
25%
0%
1990
2000
2010
2020
2030
Fuel Type
Total Clean
Total Polluting

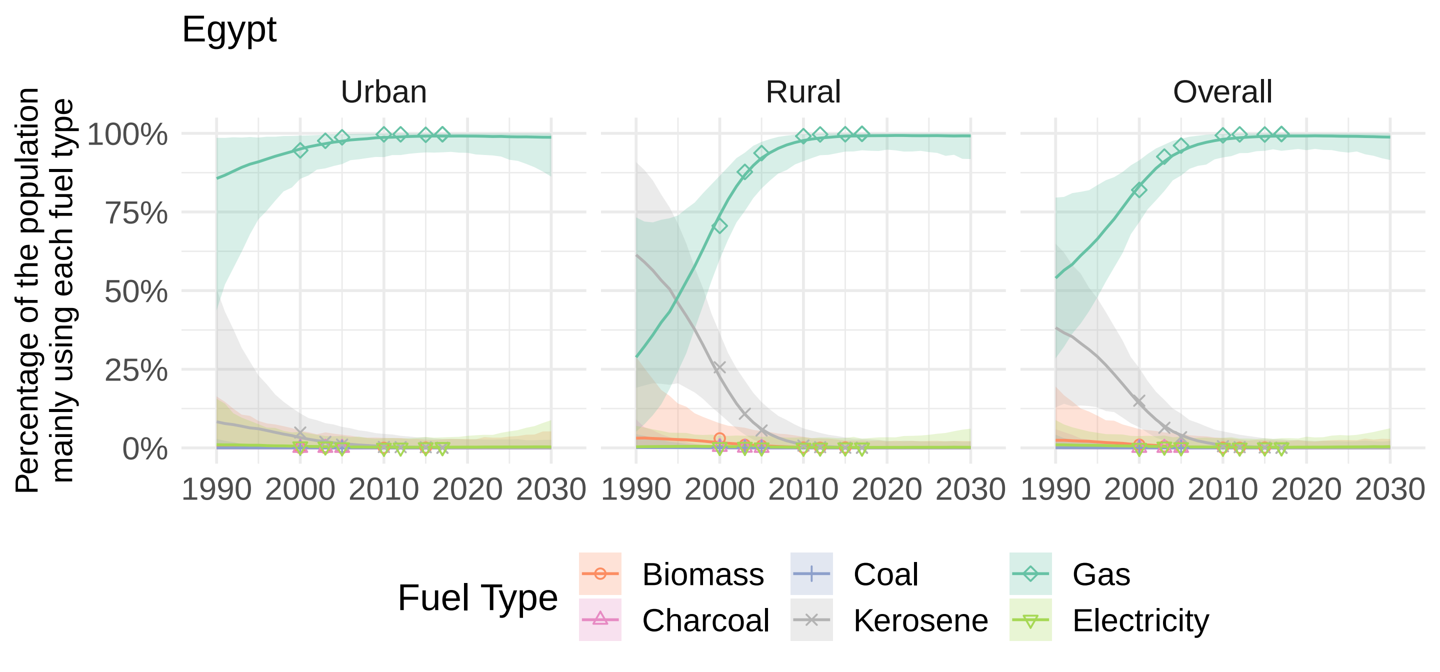
Egypt
Urban
Rural
Overall
Percentage of the population mainly using each fuel type
100%
75%
50%
25%
0%
1990
2000
2010
2020
2030
Fuel Type
Biomass
Charcoal
Coal
Kerosene
Gas
Electricity

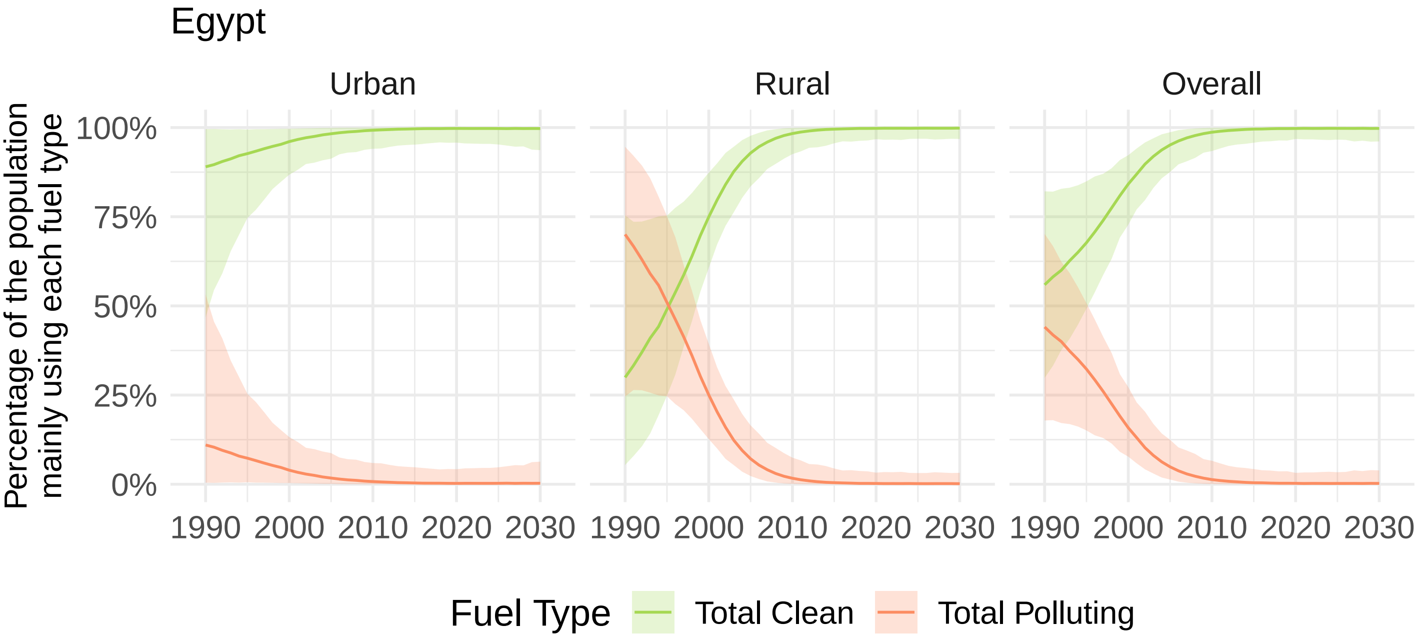

Egypt
Urban
Rural
Overall
Percentage of the population mainly using each fuel type
100%
75%
50%
25%
0%
1990
2000
2010
2020
2030
Fuel Type
Total Clean
Total Polluting

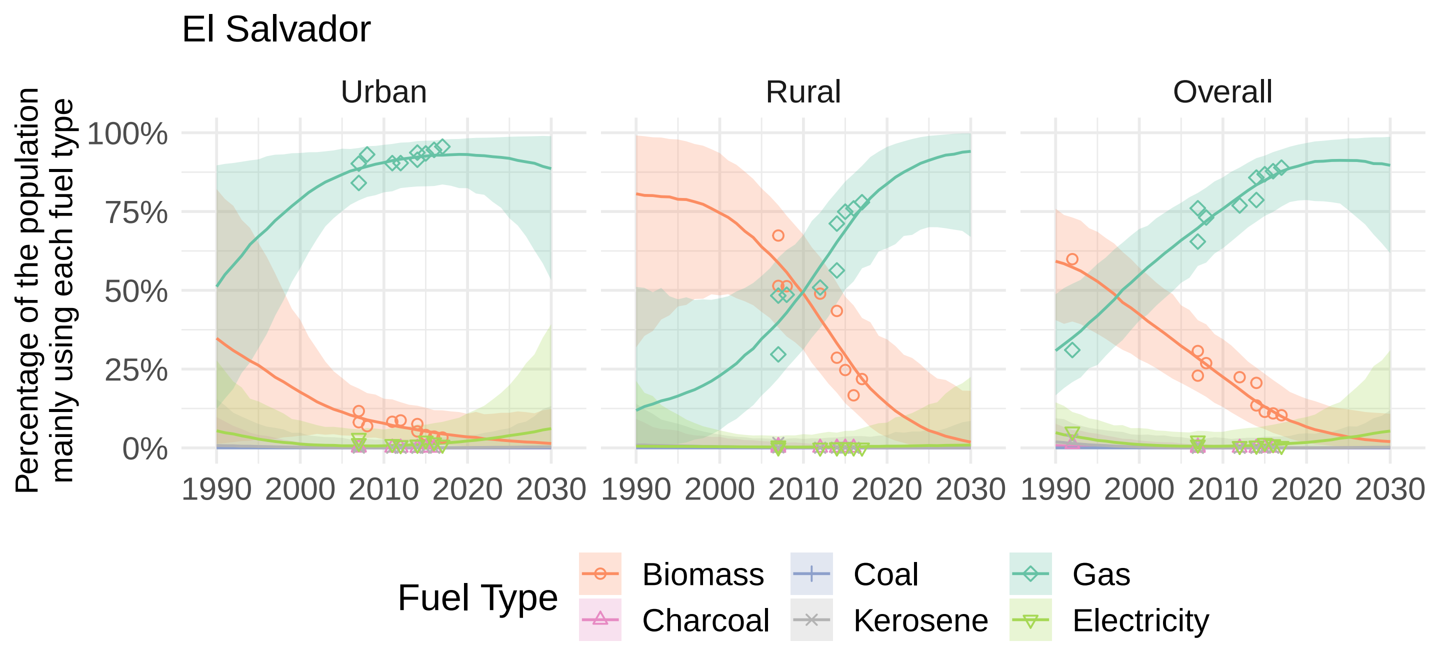
El Salvador
Urban
Rural
Overall
Percentage of the population mainly using each fuel type
100%
75%
50%
25%
0%
1990
2000
2010
2020
2030
Fuel Type
Biomass
Charcoal
Coal
Kerosene
Gas
Electricity

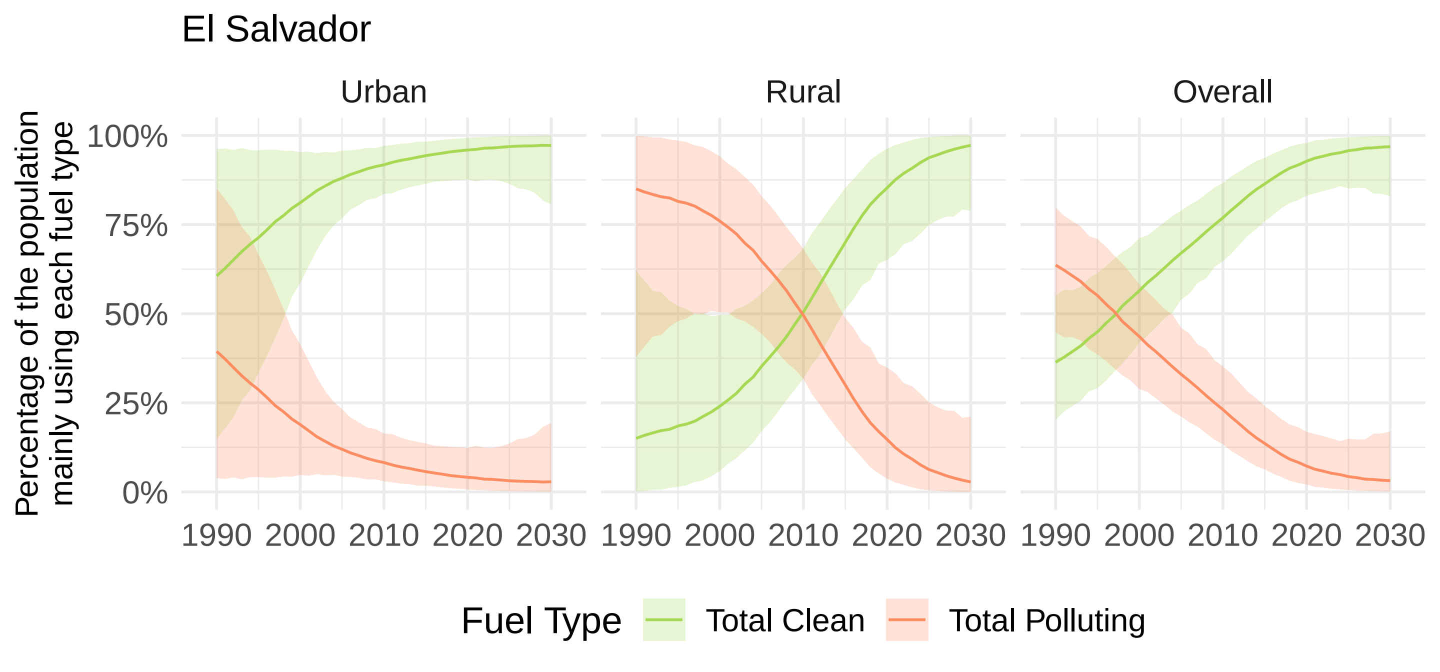
El Salvador
Urban
Rural
Overall
Percentage of the population mainly using each fuel type
100%
75%
50%
25%
0%
1990
2000
2010
2020
2030
Fuel Type
Total Clean
Total Polluting

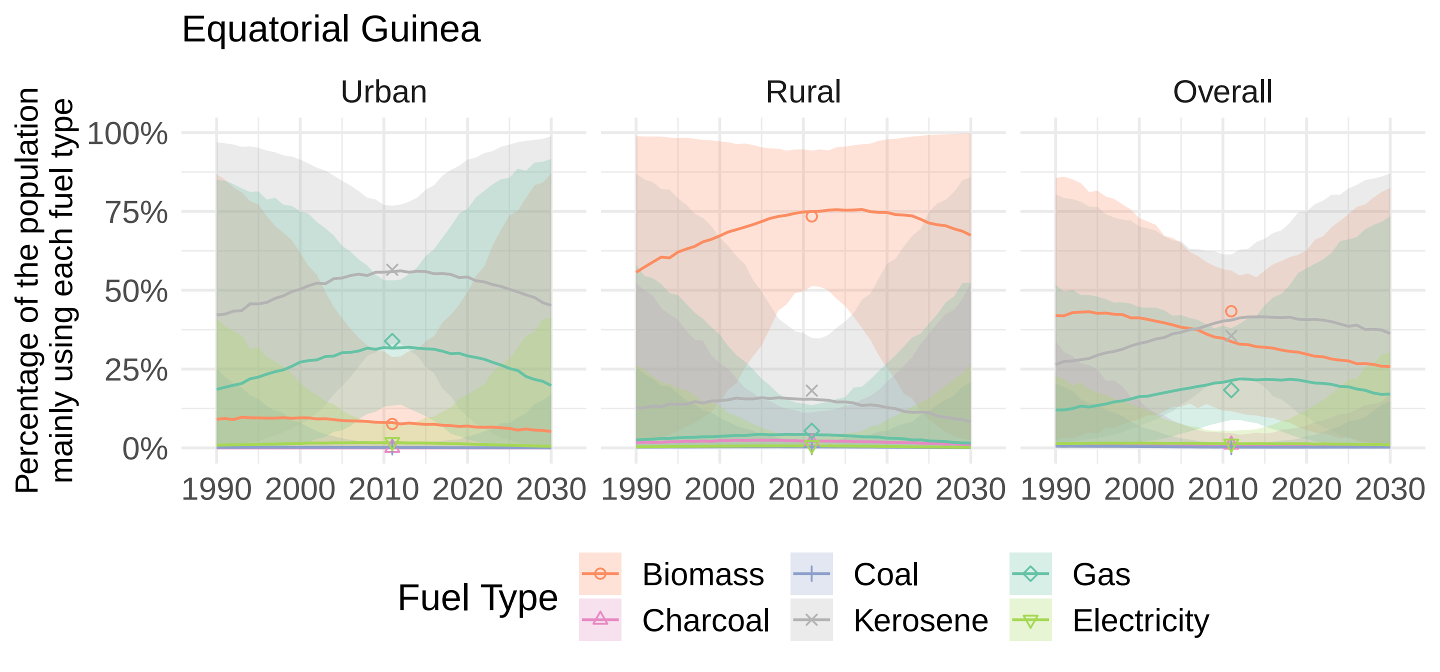
Equatorial Guinea
Urban
Rural
Overall
Percentage of the population mainly using each fuel type
100%
75%
50%
25%
0%
1990
2000
2010
2020
2030
Fuel Type
Biomass
Charcoal
Coal
Kerosene
Gas
Electricity

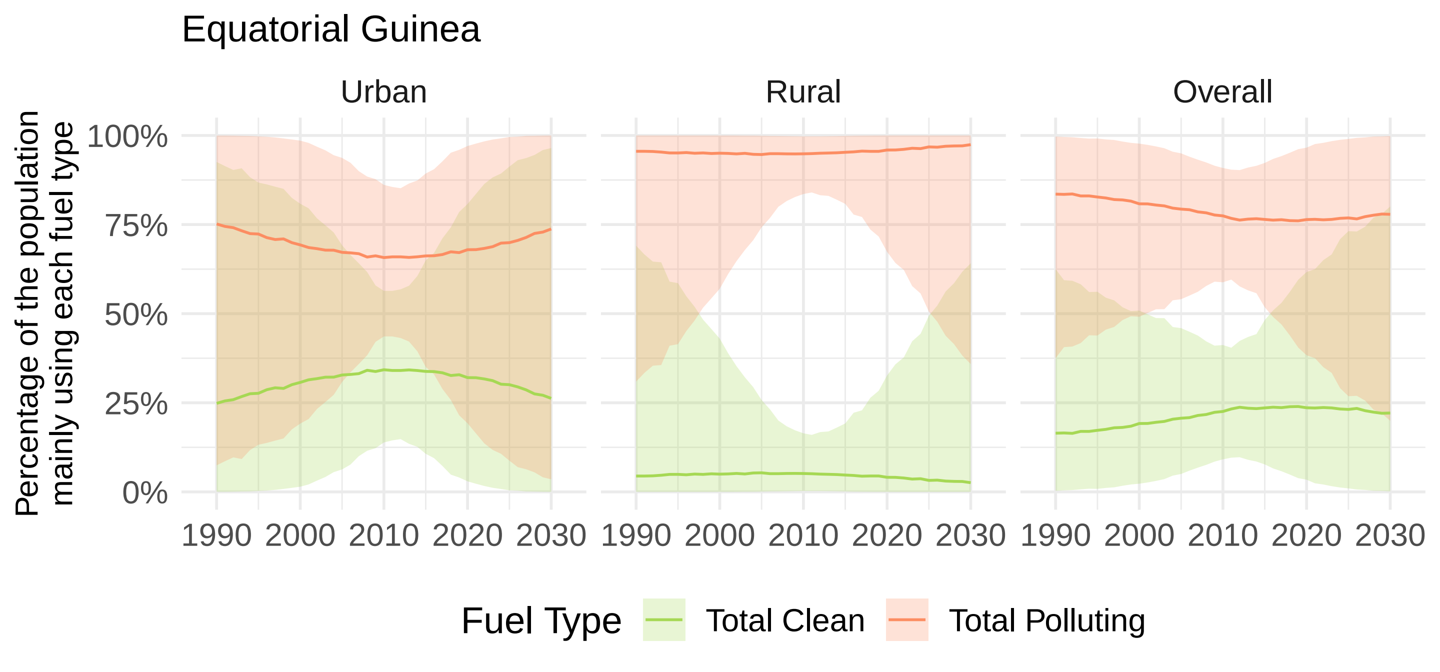
Equatorial Guinea
Urban
Rural
Overall
Percentage of the population mainly using each fuel type
100%
75%
50%
25%
0%
1990
2000
2010
2020
2030
Fuel Type
Total Clean
Total Polluting

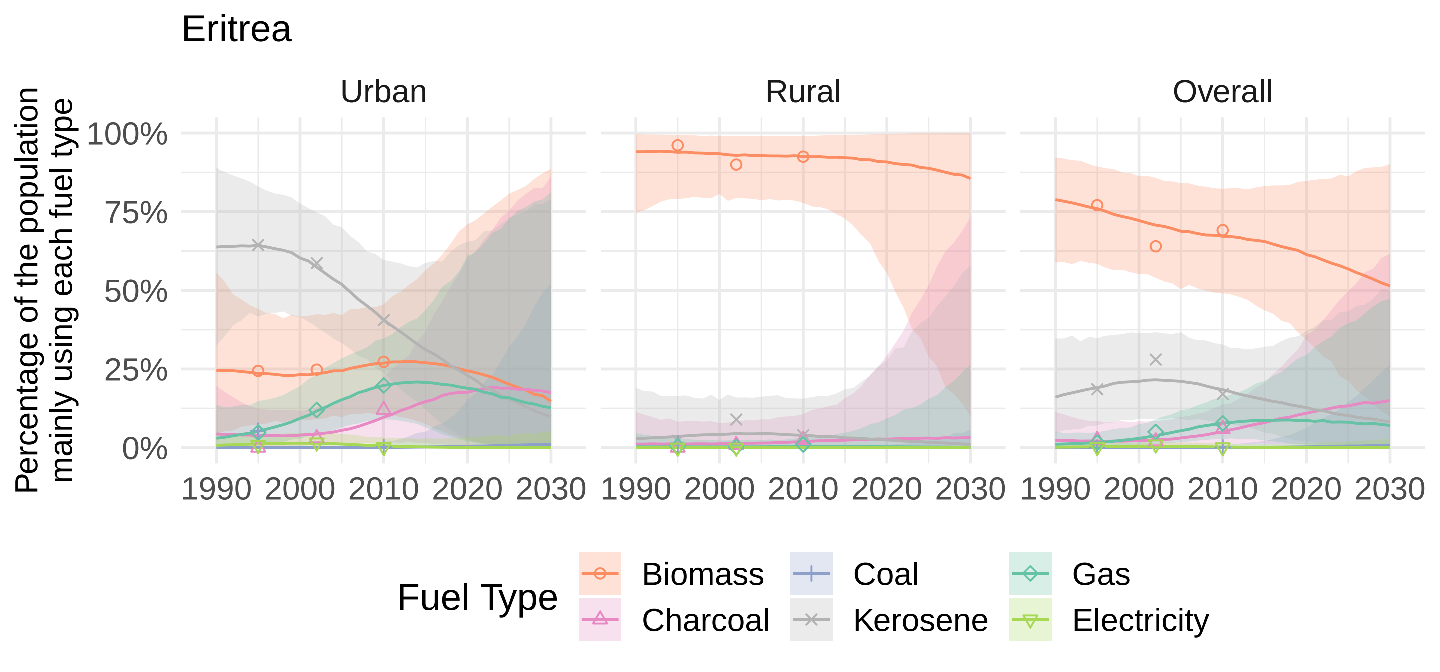
Eritrea
Urban
Rural
Overall
Percentage of the population mainly using each fuel type
100%
75%
50%
25%
0%
1990
2000
2010
2020
2030
Fuel Type
Biomass
Charcoal
Coal
Kerosene
Gas
Electricity

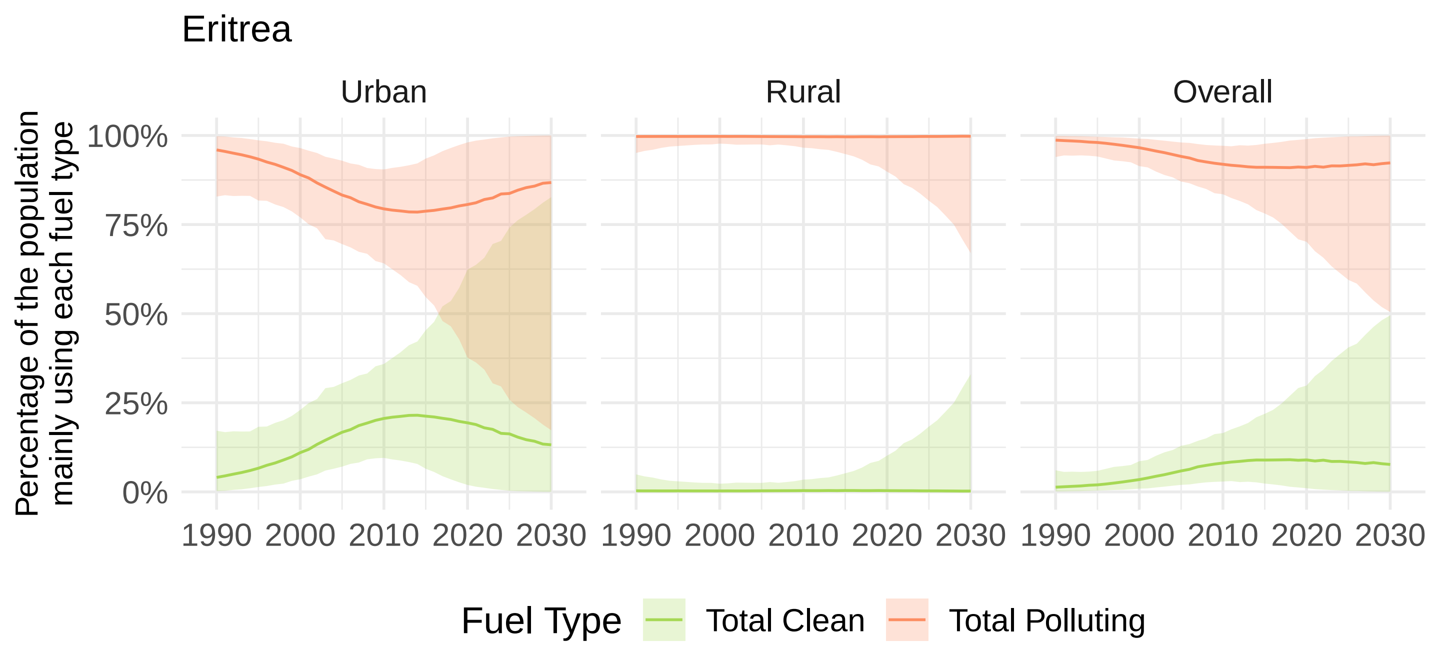

Eritrea
Urban
Rural
Overall
Percentage of the population mainly using each fuel type
100%
75%
50%
25%
0%
1990
2000
2010
2020
2030
Fuel Type
Total Clean
Total Polluting

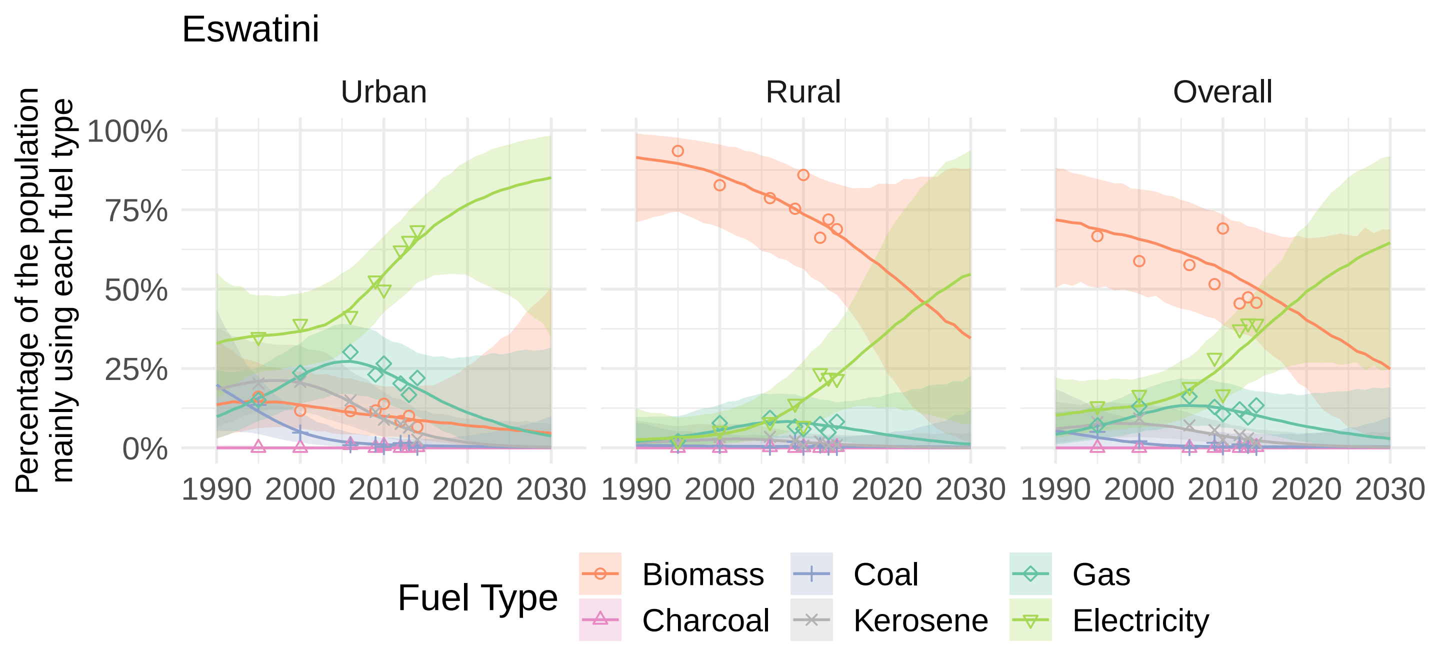

Eswatini
Urban
Rural
Overall
Percentage of the population mainly using each fuel type
100%
75%
50%
25%
0%
1990
2000
2010
2020
2030
Fuel Type
Biomass
Charcoal
Coal
Kerosene
Gas
Electricity

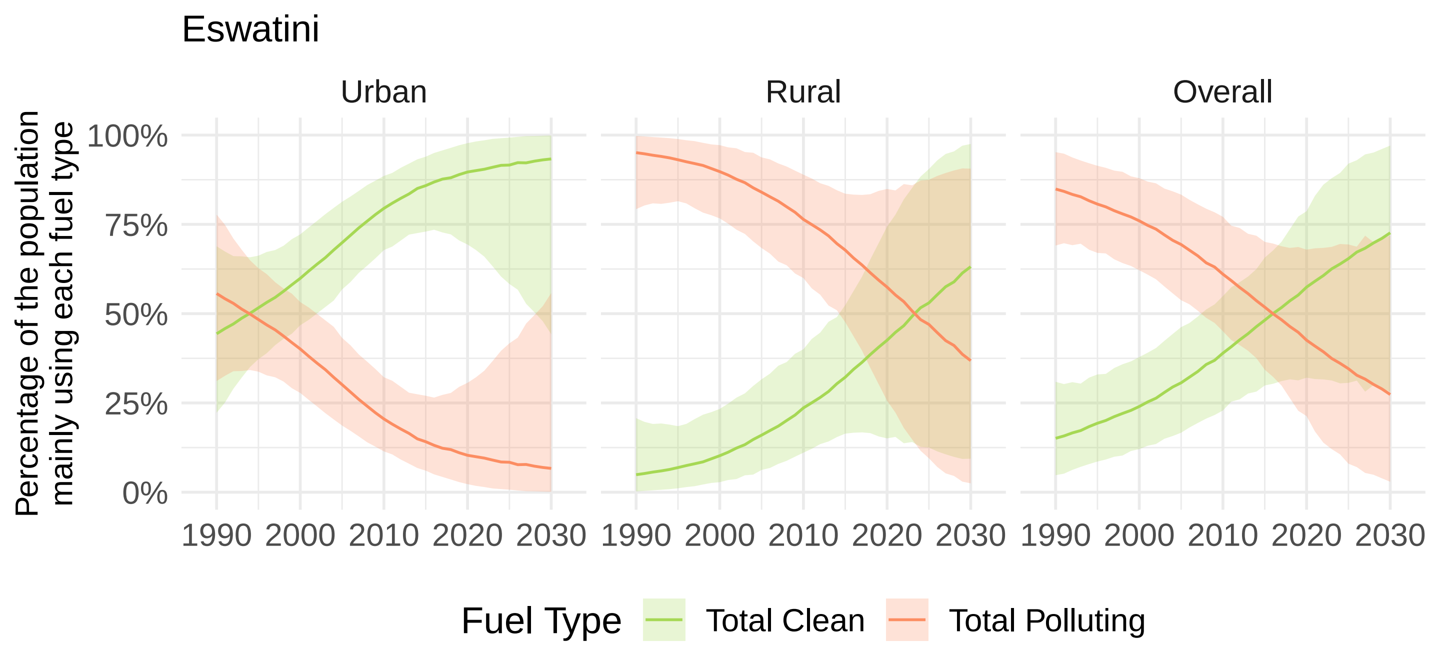
Eswatini
Urban
Rural
Overall
Percentage of the population mainly using each fuel type
100%
75%
50%
25%
0%
1990
2000
2010
2020
2030
Fuel Type
Total Clean
Total Polluting

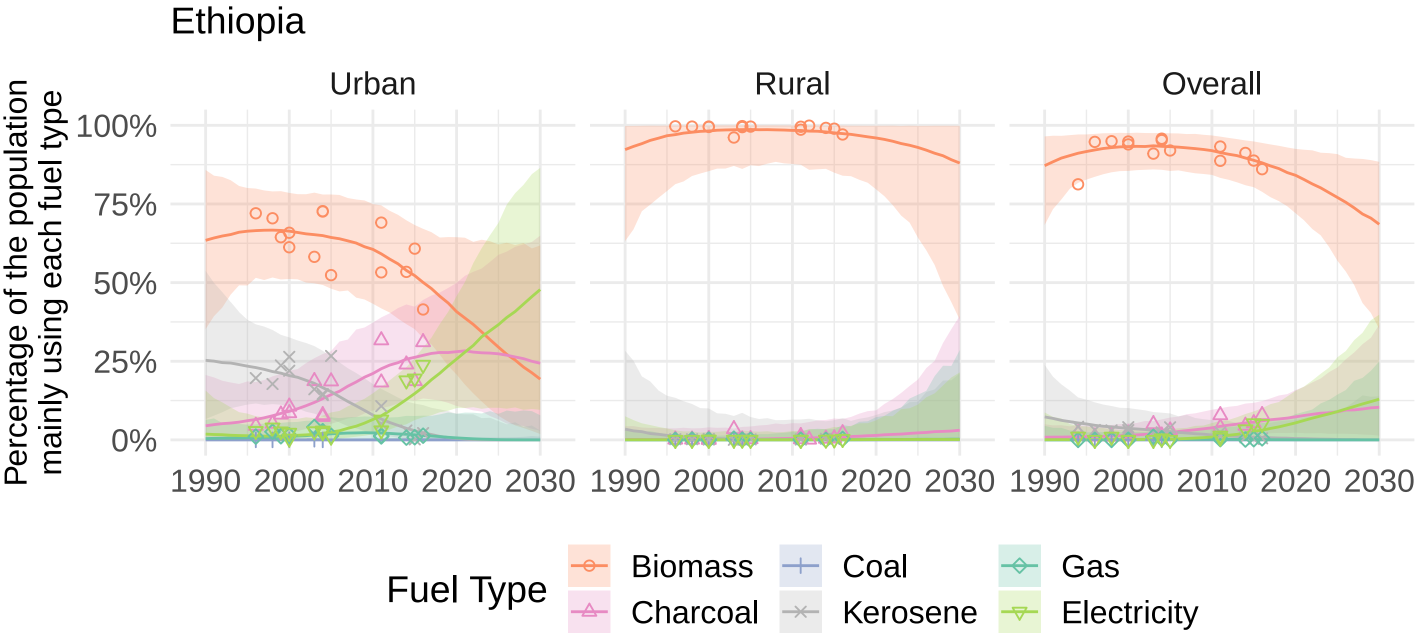
Ethiopia
Urban
Rural
Overall
Percentage of the population mainly using each fuel type
100%
75%
50%
25%
0%
1990
2000
2010
2020
2030
Fuel Type
Biomass
Charcoal
Coal
Kerosene
Gas
Electricity

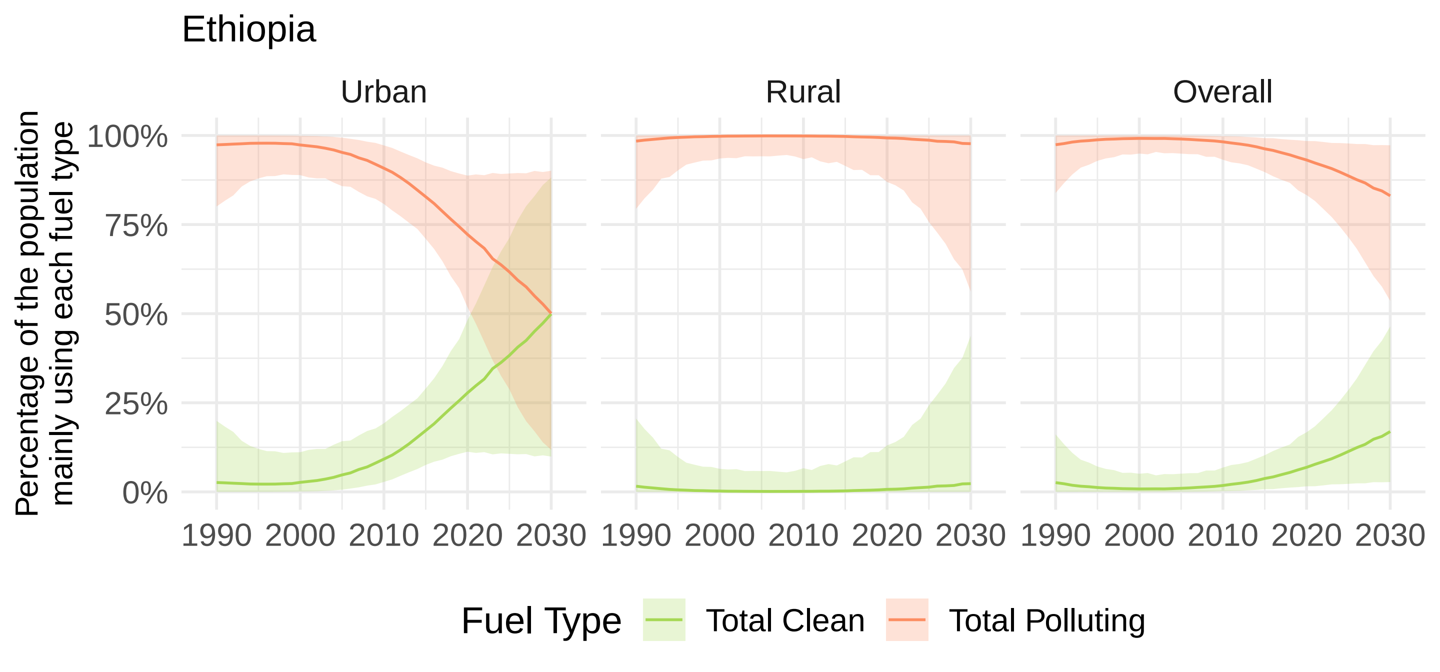
Ethiopia
Urban
Rural
Overall
Percentage of the population mainly using each fuel type
100%
75%
50%
25%
0%
1990
2000
2010
2020
2030
Fuel Type
Total Clean
Total Polluting

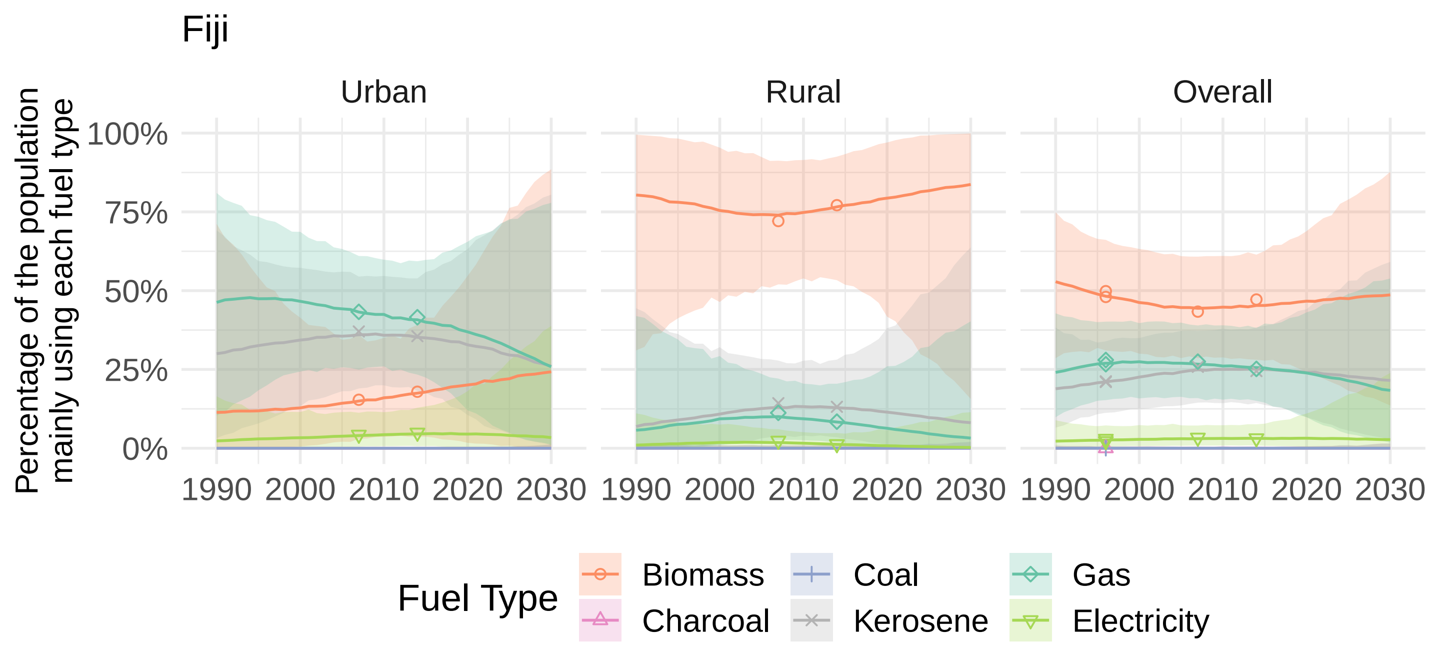

Fiji
Urban
Rural
Overall
Percentage of the population mainly using each fuel type
100%
75%
50%
25%
0%
1990
2000
2010
2020
2030
Fuel Type
Biomass
Charcoal
Coal
Kerosene
Gas
Electricity

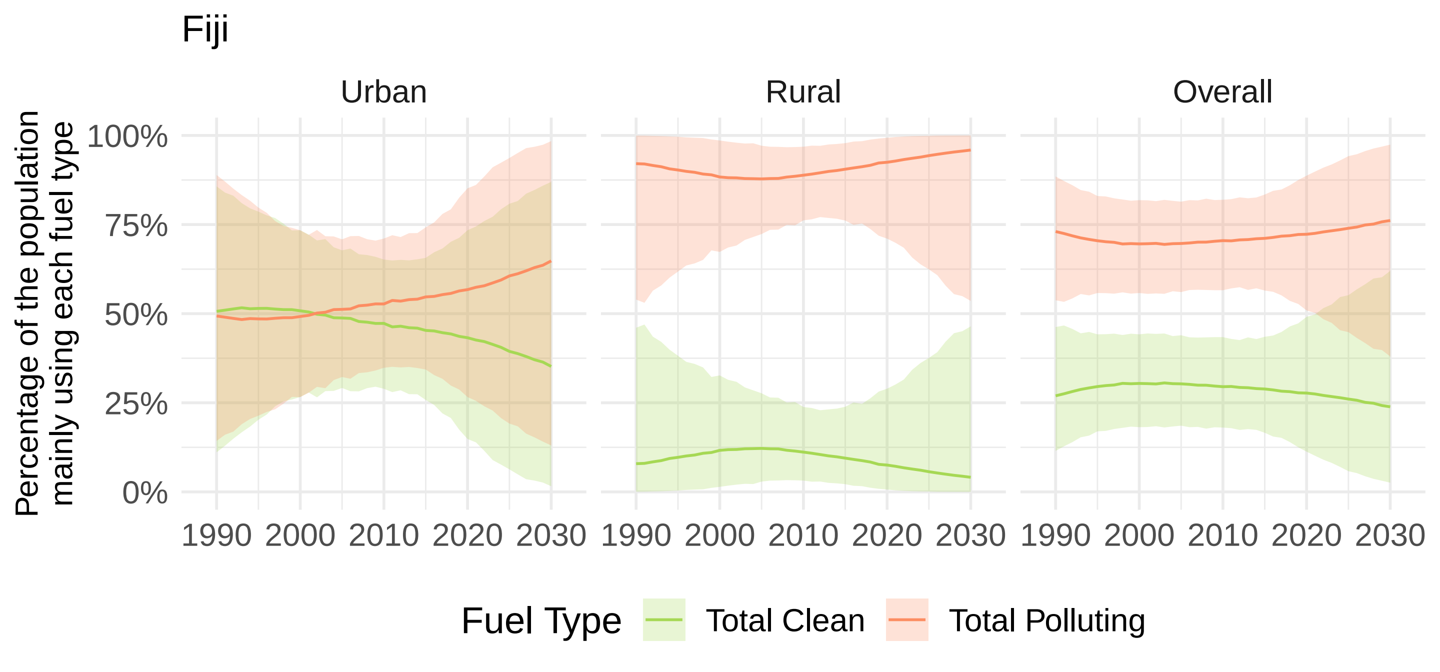

Fiji
Urban
Rural
Overall
Percentage of the population mainly using each fuel type
100%
75%
50%
25%
0%
1990
2000
2010
2020
2030
Fuel Type
Total Clean
Total Polluting

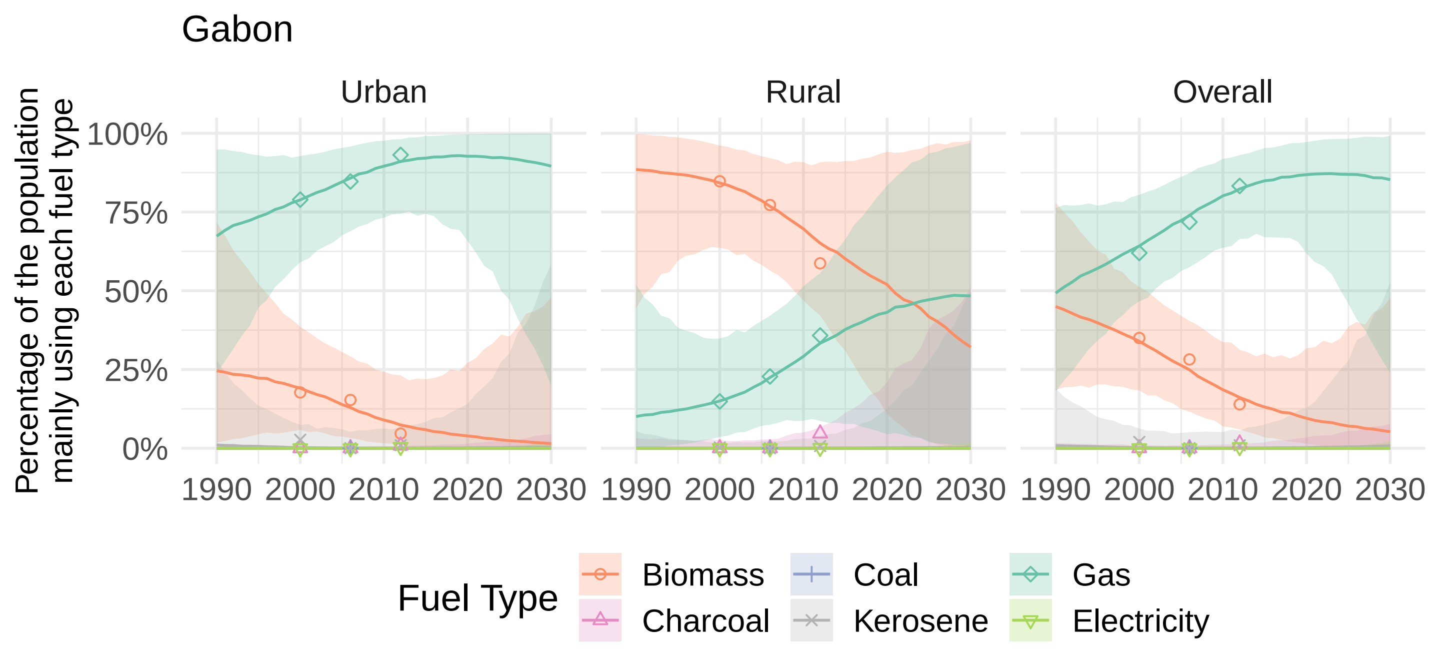

Gabon
Urban
Rural
Overall
Percentage of the population mainly using each fuel type
100%
75%
50%
25%
0%
1990
2000
2010
2020
2030
Fuel Type
Biomass
Charcoal
Coal
Kerosene
Gas
Electricity

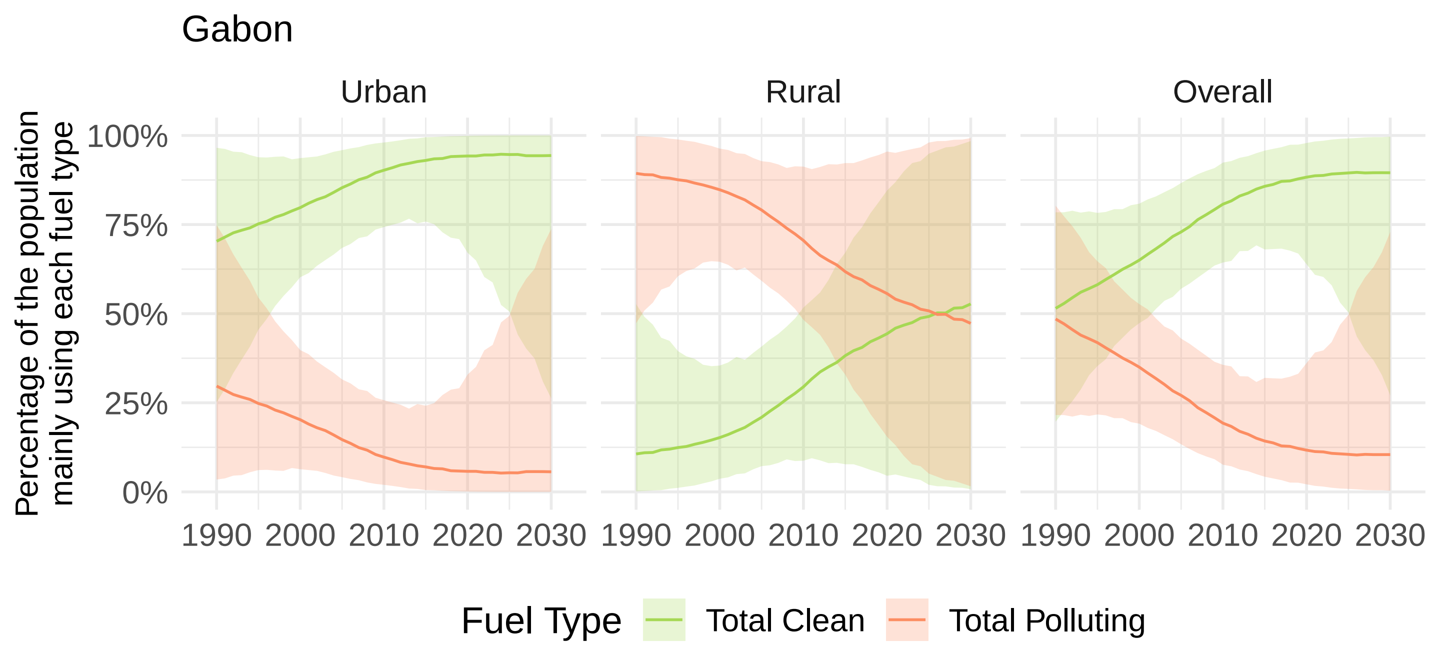

Gabon
Urban
Rural
Overall
Percentage of the population mainly using each fuel type
100%
75%
50%
25%
0%
1990
2000
2010
2020
2030
Fuel Type
Total Clean
Total Polluting

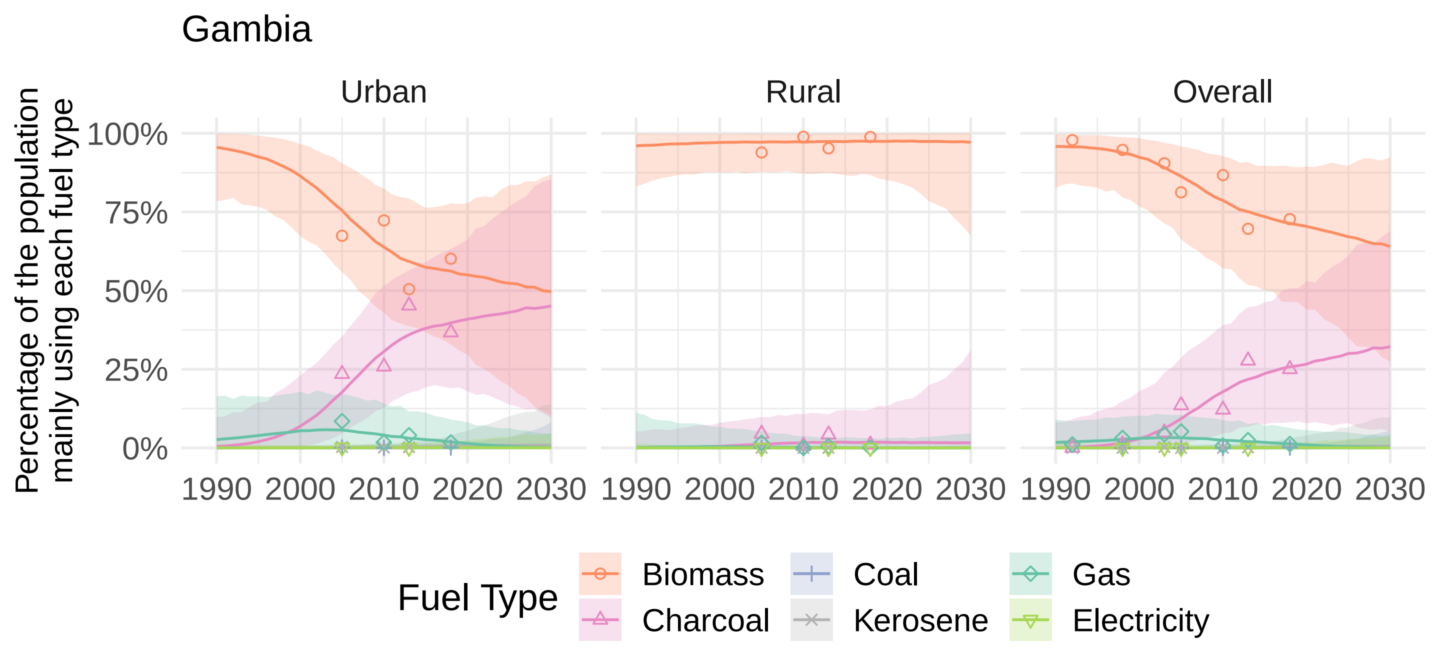

Gambia
Urban
Rural
Overall
Percentage of the population mainly using each fuel type
100%
75%
50%
25%
0%
1990
2000
2010
2020
2030
Fuel Type
Biomass
Charcoal
Coal
Kerosene
Gas
Electricity

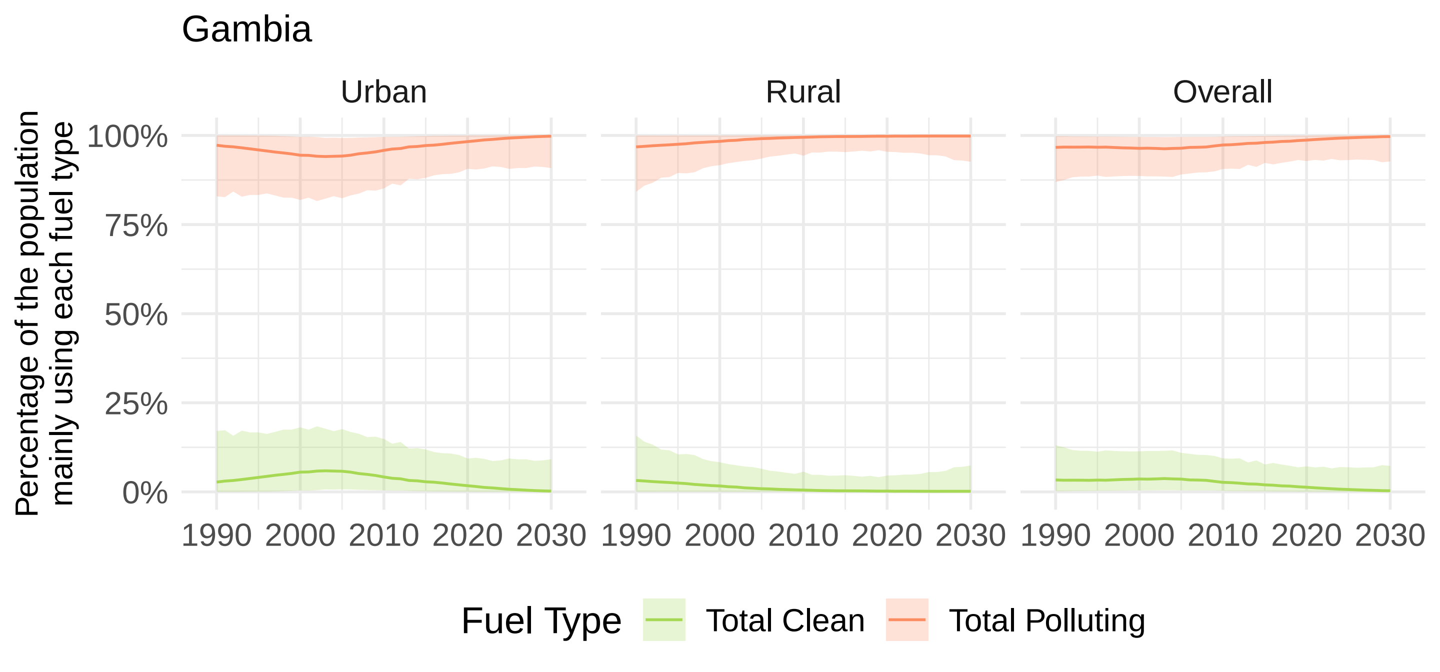

Gambia
Urban
Rural
Overall
Percentage of the population mainly using each fuel type
100%
75%
50%
25%
0%
1990
2000
2010
2020
2030
Fuel Type
Total Clean
Total Polluting

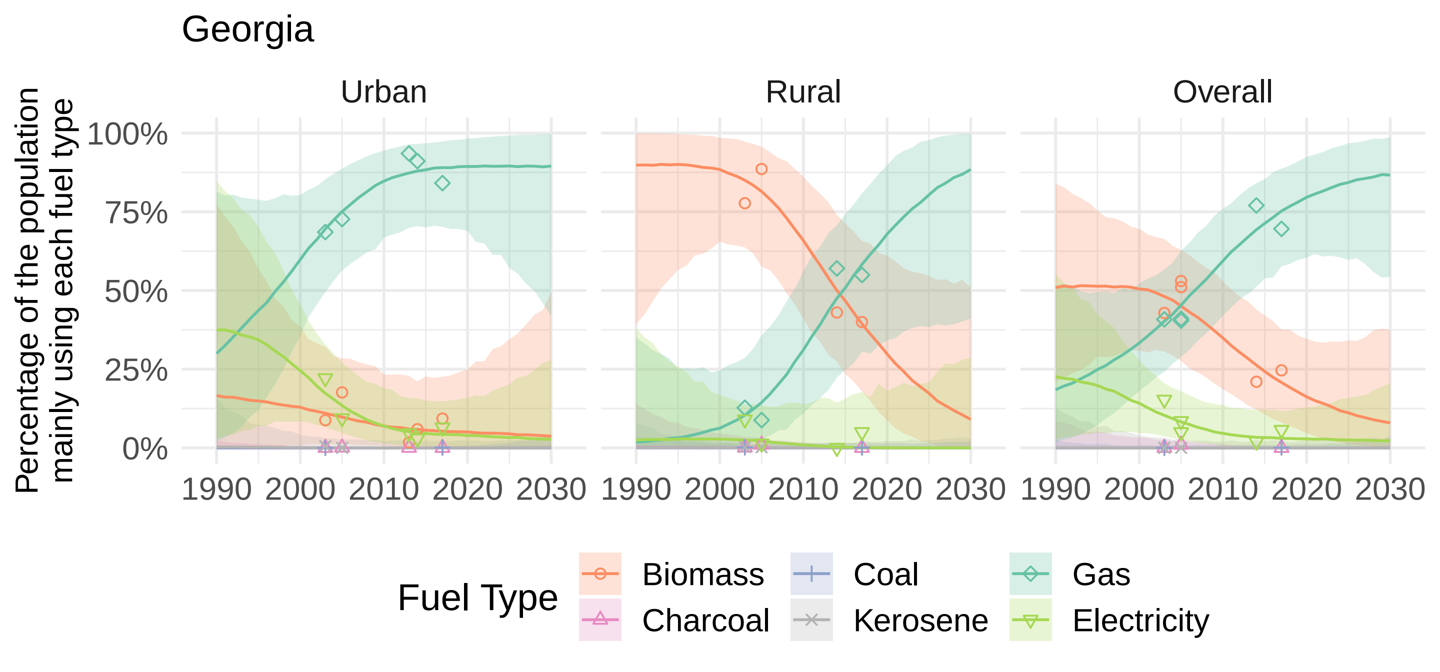

Georgia
Urban
Rural
Overall
Percentage of the population mainly using each fuel type
100%
75%
50%
25%
0%
1990
2000
2010
2020
2030
Fuel Type
Biomass
Charcoal
Coal
Kerosene
Gas
Electricity

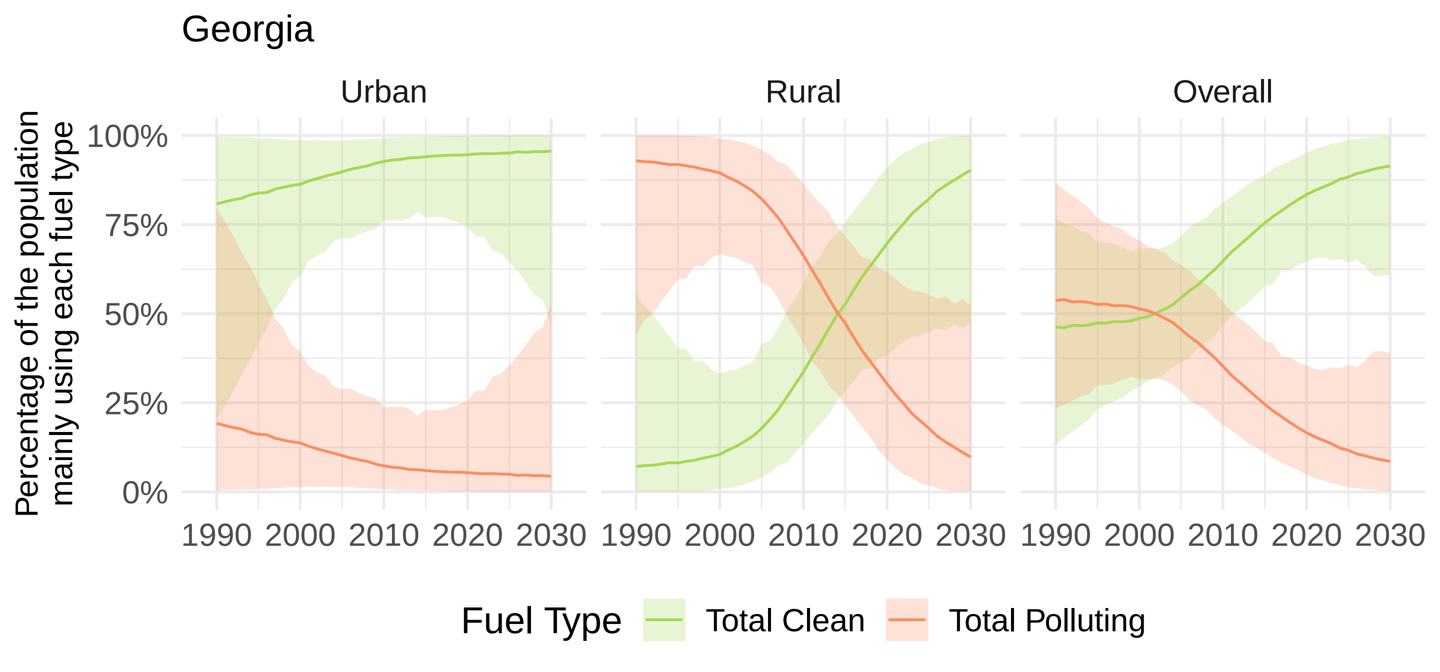
Georgia
Urban
Rural
Overall
Percentage of the population mainly using each fuel type
100%
75%
50%
25%
0%
1990
2000
2010
2020
2030
Fuel Type
Total Clean
Total Polluting

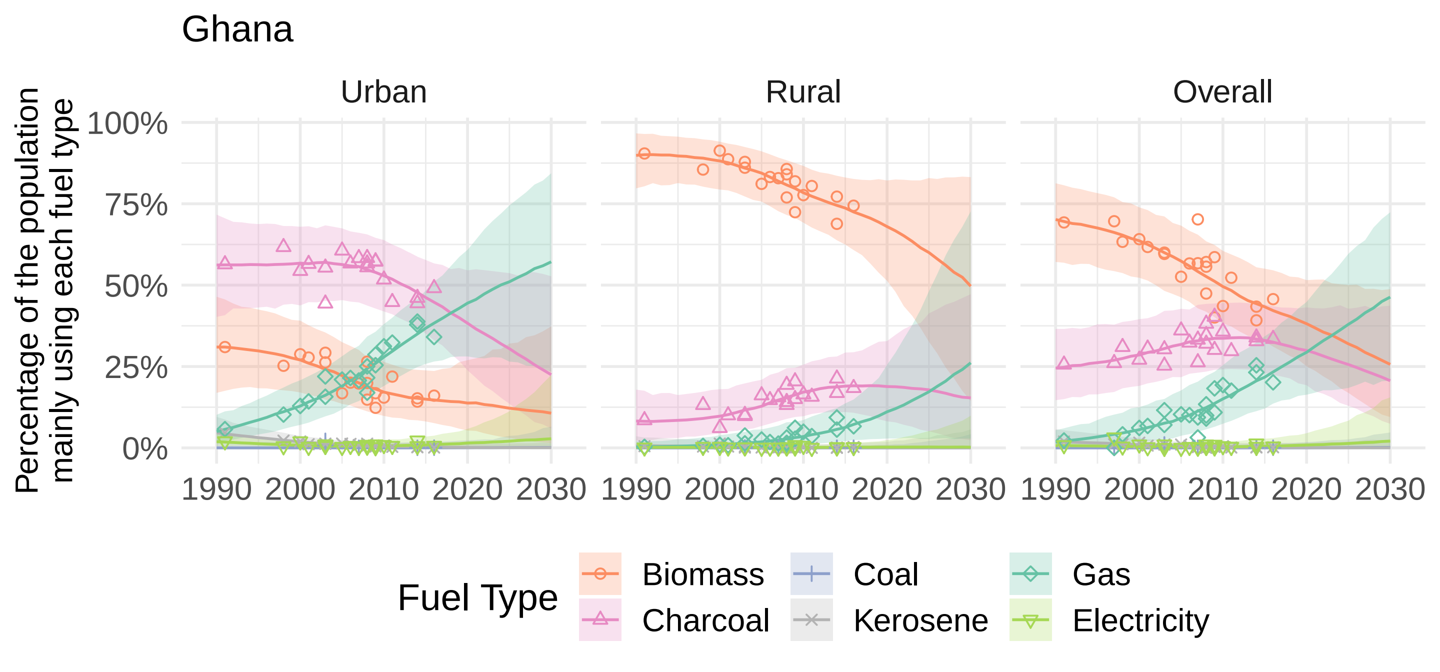

Ghana
Urban
Rural
Overall
Percentage of the population mainly using each fuel type
100%
75%
50%
25%
0%
1990
2000
2010
2020
2030
Fuel Type
Biomass
Charcoal
Coal
Kerosene
Gas
Electricity

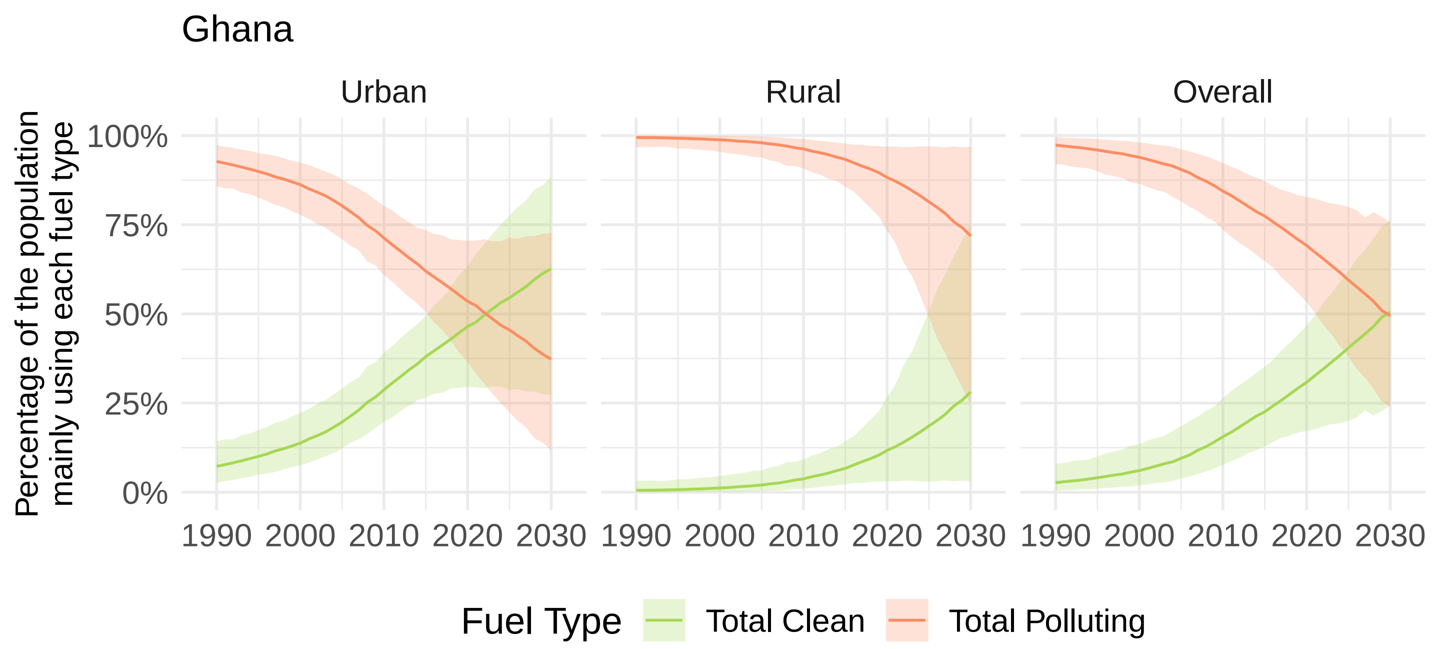
Ghana
Urban
Rural
Overall
Percentage of the population mainly using each fuel type
100%
75%
50%
25%
0%
1990
2000
2010
2020
2030
Fuel Type
Total Clean
Total Polluting

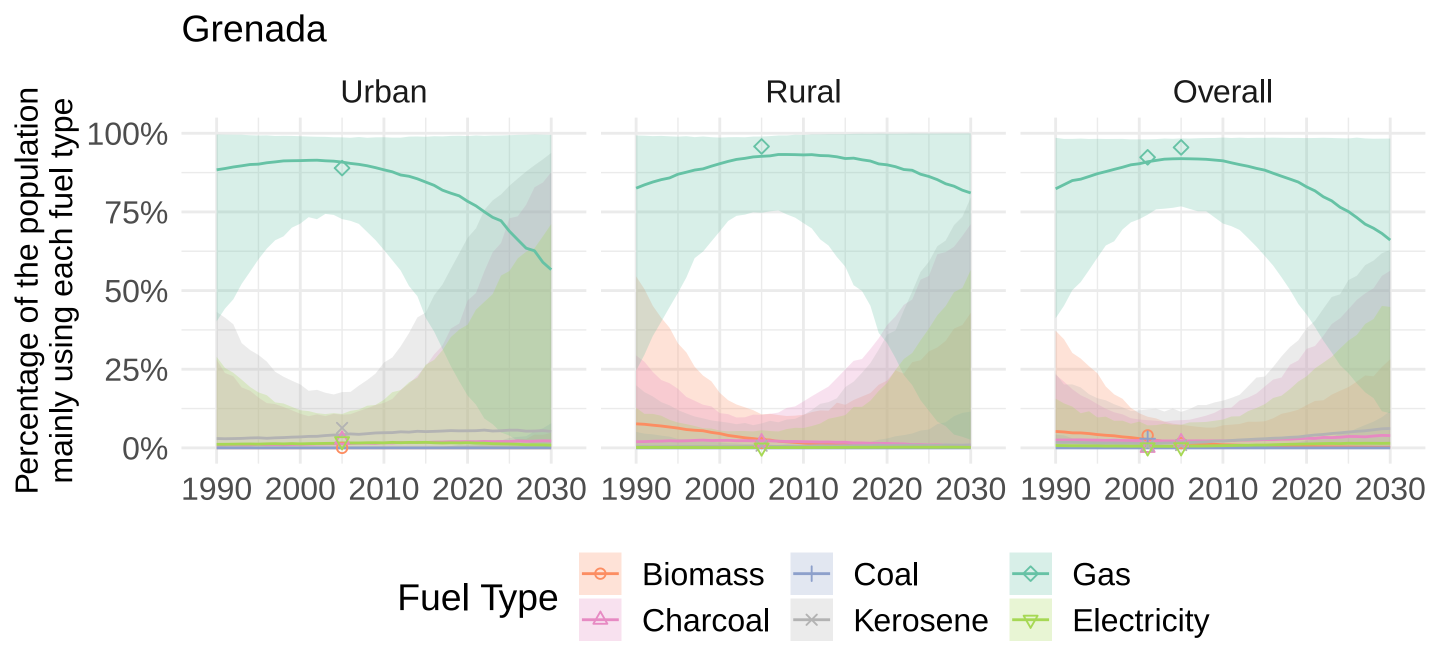

Grenada
Urban
Rural
Overall
Percentage of the population mainly using each fuel type
100%
75%
50%
25%
0%
1990
2000
2010
2020
2030
Fuel Type
Biomass
Charcoal
Coal
Kerosene
Gas
Electricity

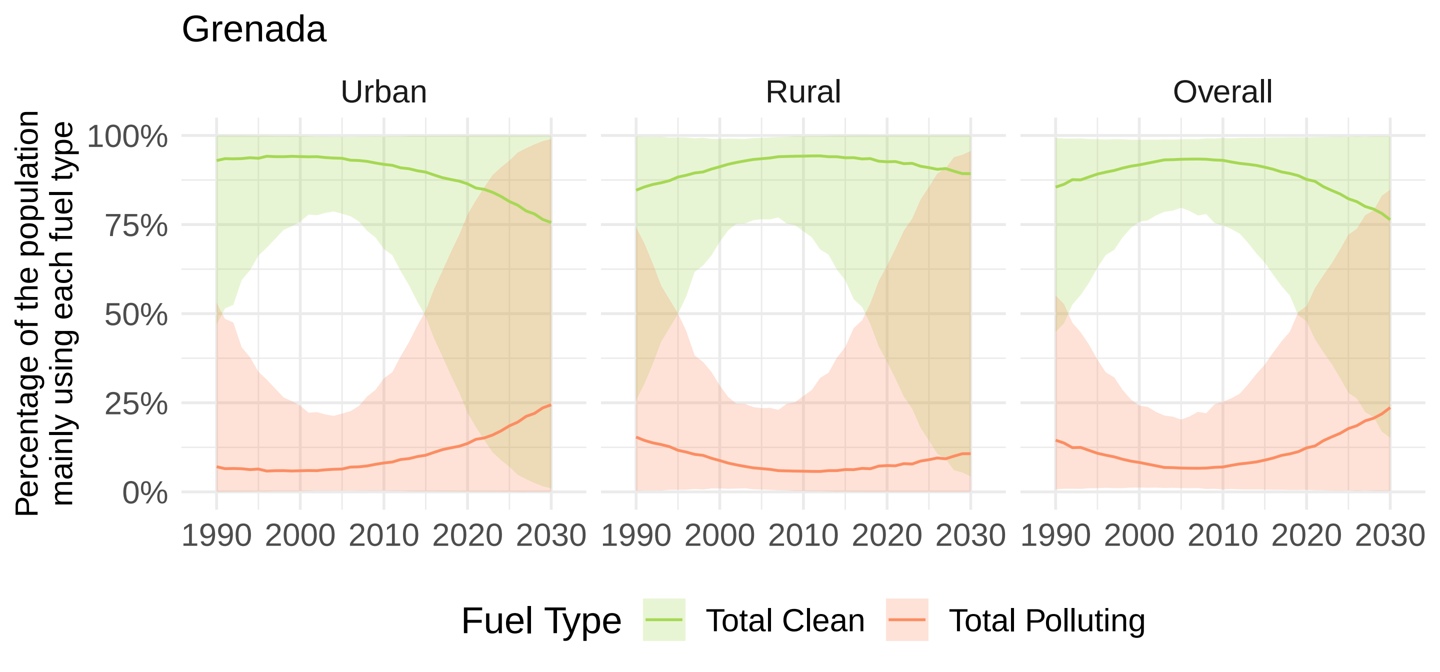
Grenada
Urban
Rural
Overall
Percentage of the population mainly using each fuel type
100%
75%
50%
25%
0%
1990
2000
2010
2020
2030
Fuel Type
Total Clean
Total Polluting

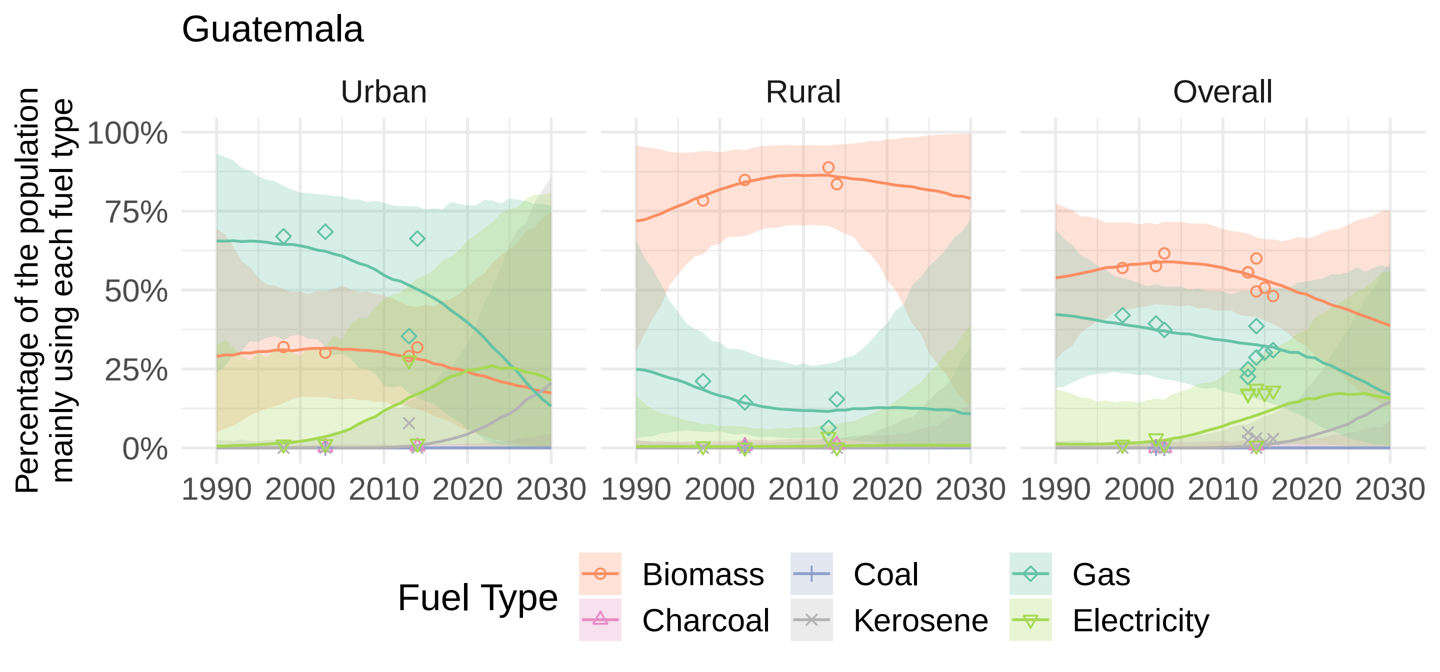
Guatemala
Urban
Rural
Overall
Percentage of the population mainly using each fuel type
100%
75%
50%
25%
0%
1990
2000
2010
2020
2030
Fuel Type
Biomass
Charcoal
Coal
Kerosene
Gas
Electricity

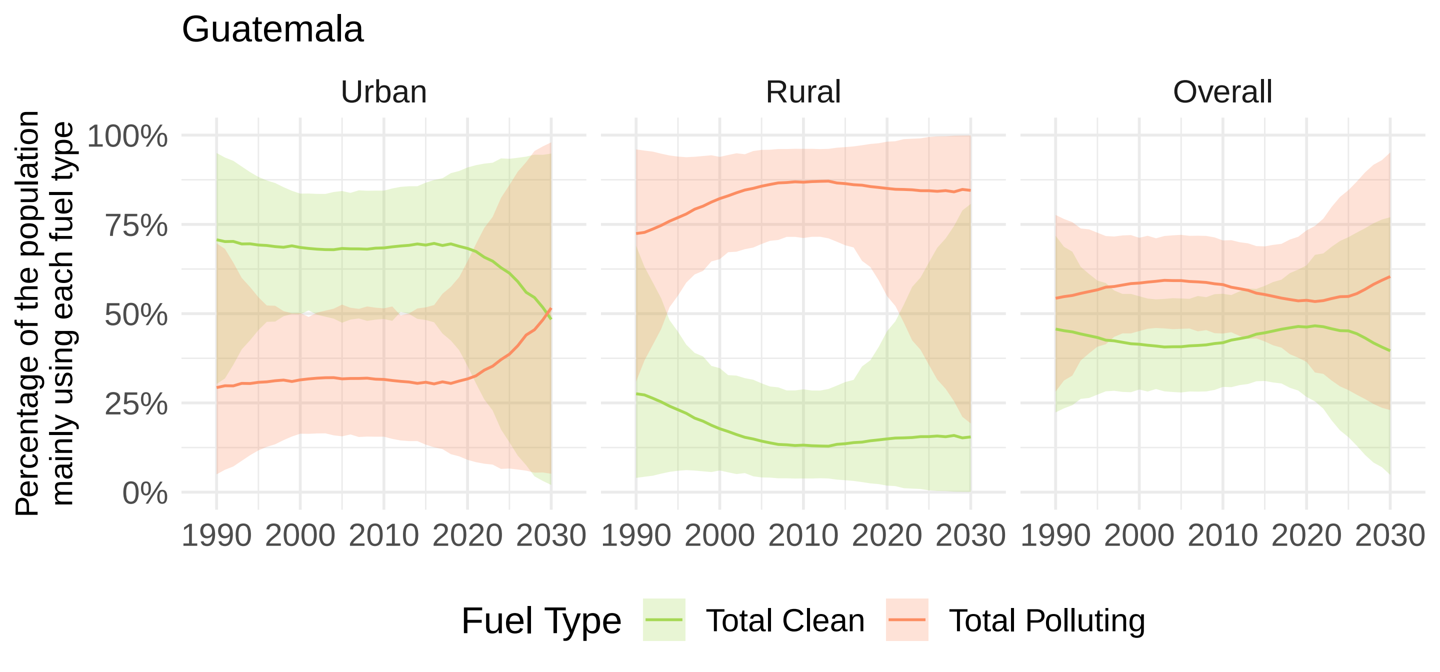
Guatemala
Urban
Rural
Overall
Percentage of the population mainly using each fuel type
100%
75%
50%
25%
0%
1990
2000
2010
2020
2030
Fuel Type
Total Clean
Total Polluting

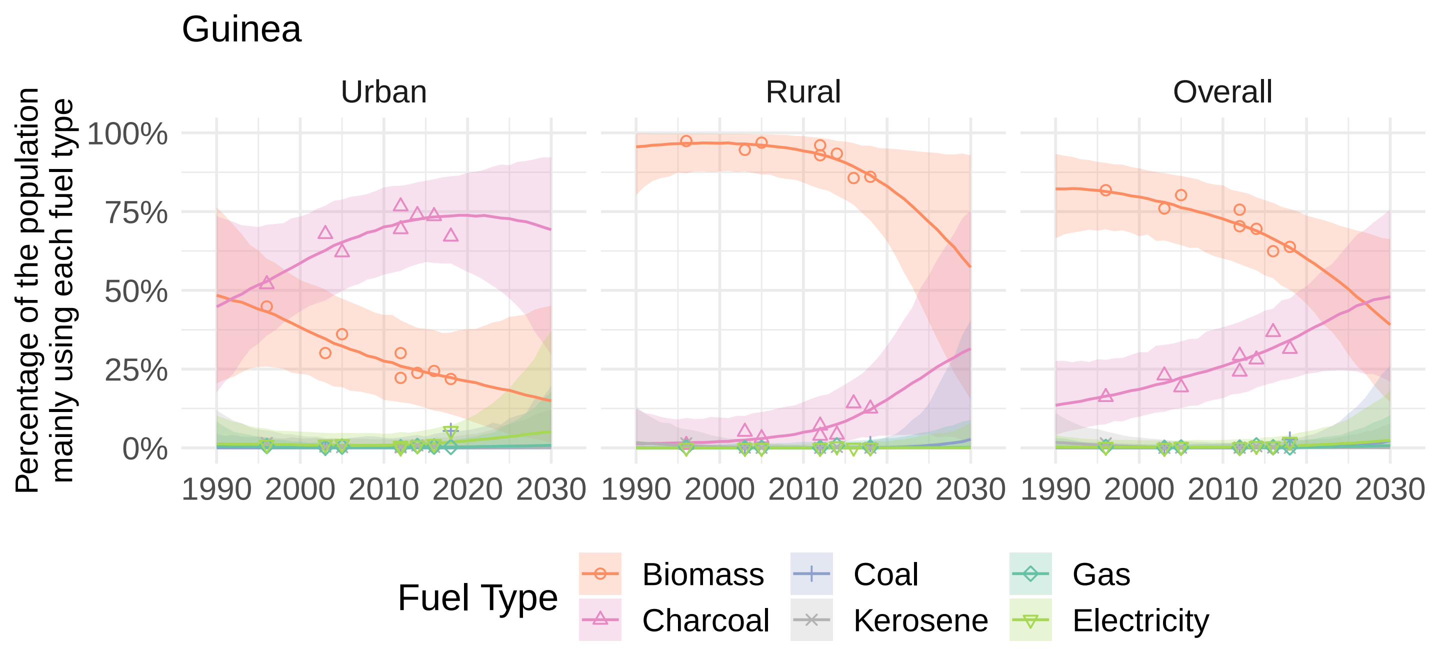
Guinea
Urban
Rural
Overall
Percentage of the population mainly using each fuel type
100%
75%
50%
25%
0%
1990
2000
2010
2020
2030
Fuel Type
Biomass
Charcoal
Coal
Kerosene
Gas
Electricity

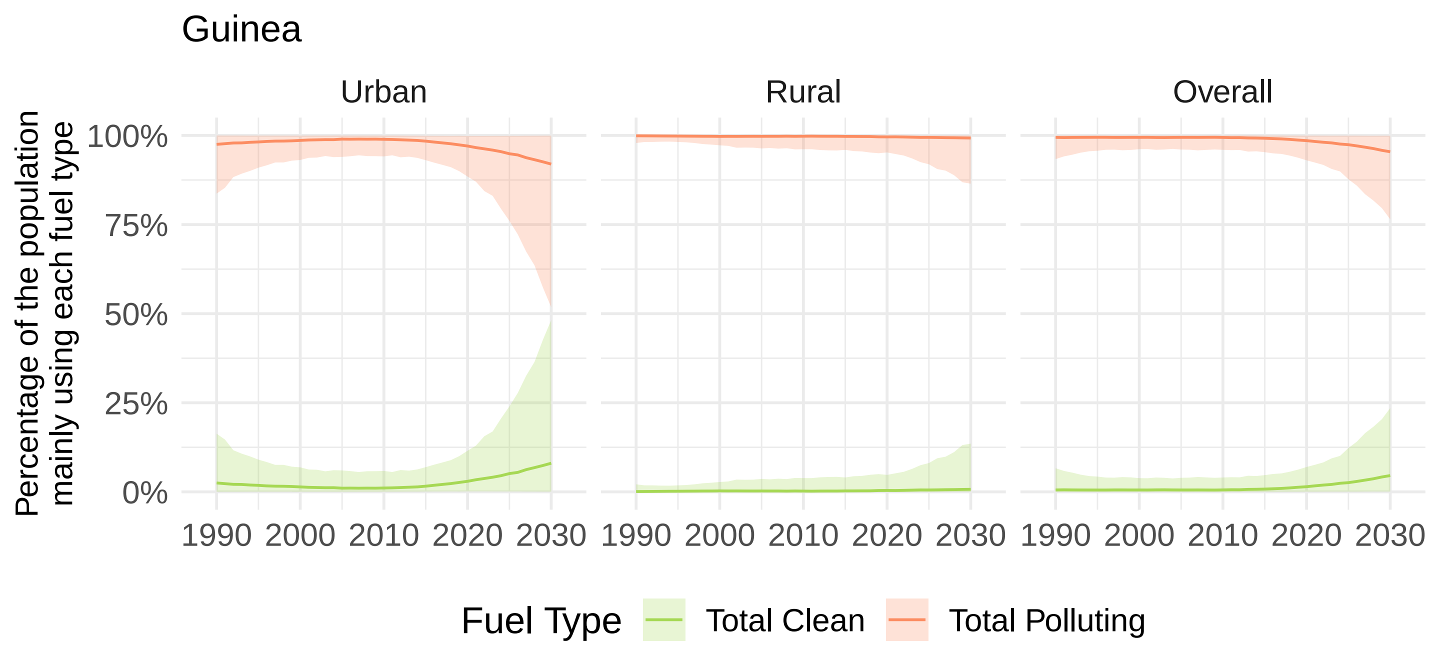

Guinea
Urban
Rural
Overall
Percentage of the population mainly using each fuel type
100%
75%
50%
25%
0%
1990
2000
2010
2020
2030
Fuel Type
Total Clean
Total Polluting

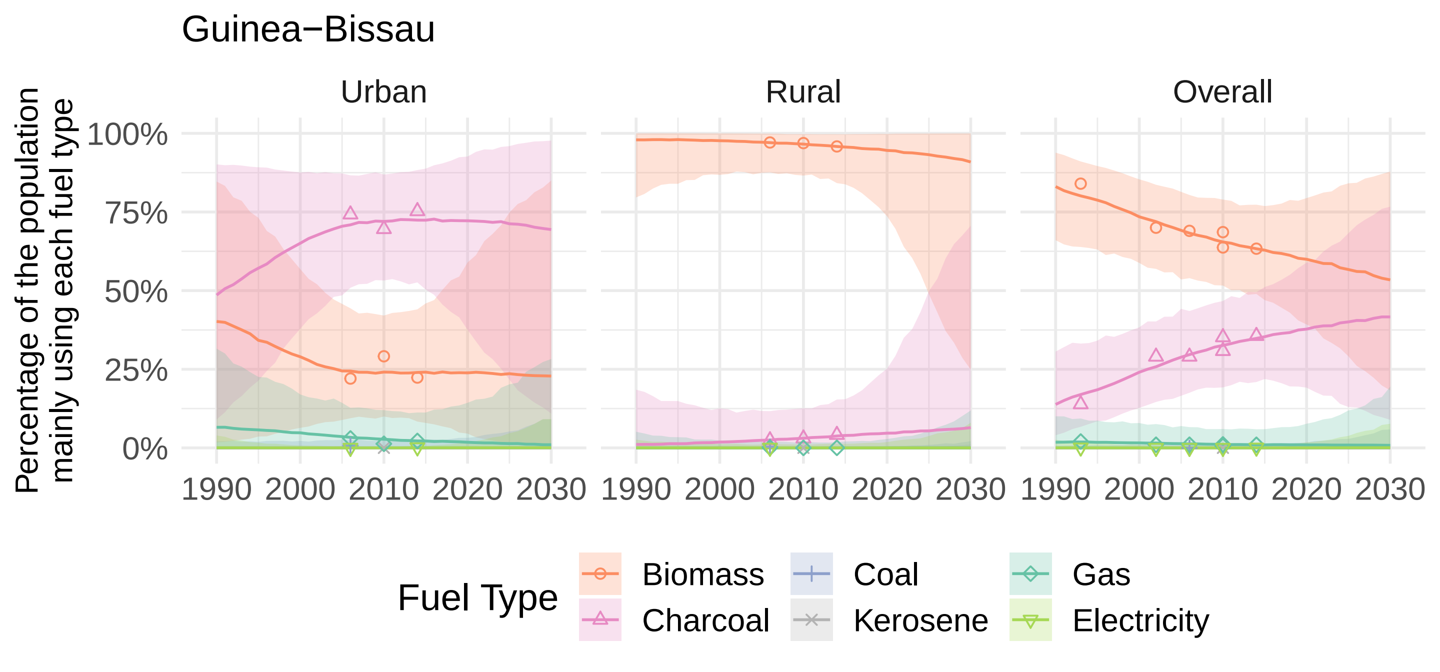

Guinea−Bissau
Urban
Rural
Overall
Percentage of the population mainly using each fuel type
100%
75%
50%
25%
0%
1990
2000
2010
2020
2030
Fuel Type
Biomass
Charcoal
Coal
Kerosene
Gas
Electricity

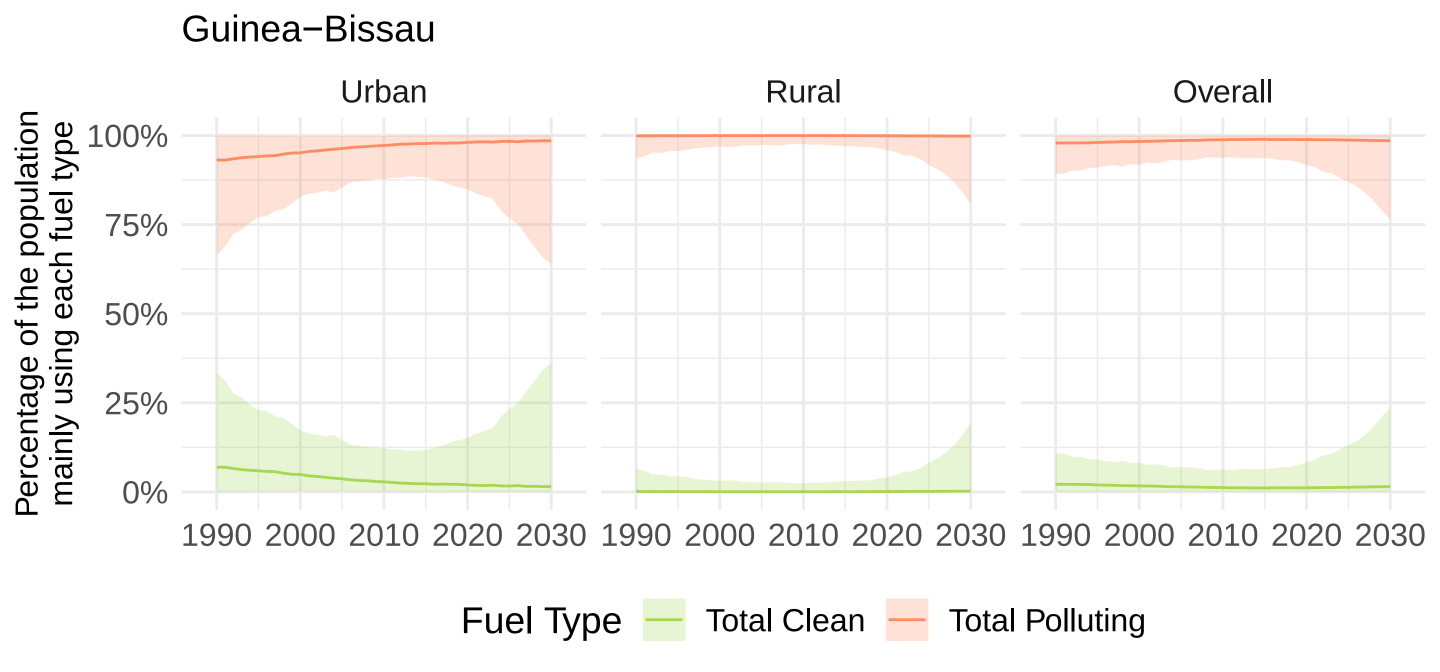

Guinea−Bissau
Urban
Rural
Overall
Percentage of the population mainly using each fuel type
100%
75%
50%
25%
0%
1990
2000
2010
2020
2030
Fuel Type
Total Clean
Total Polluting

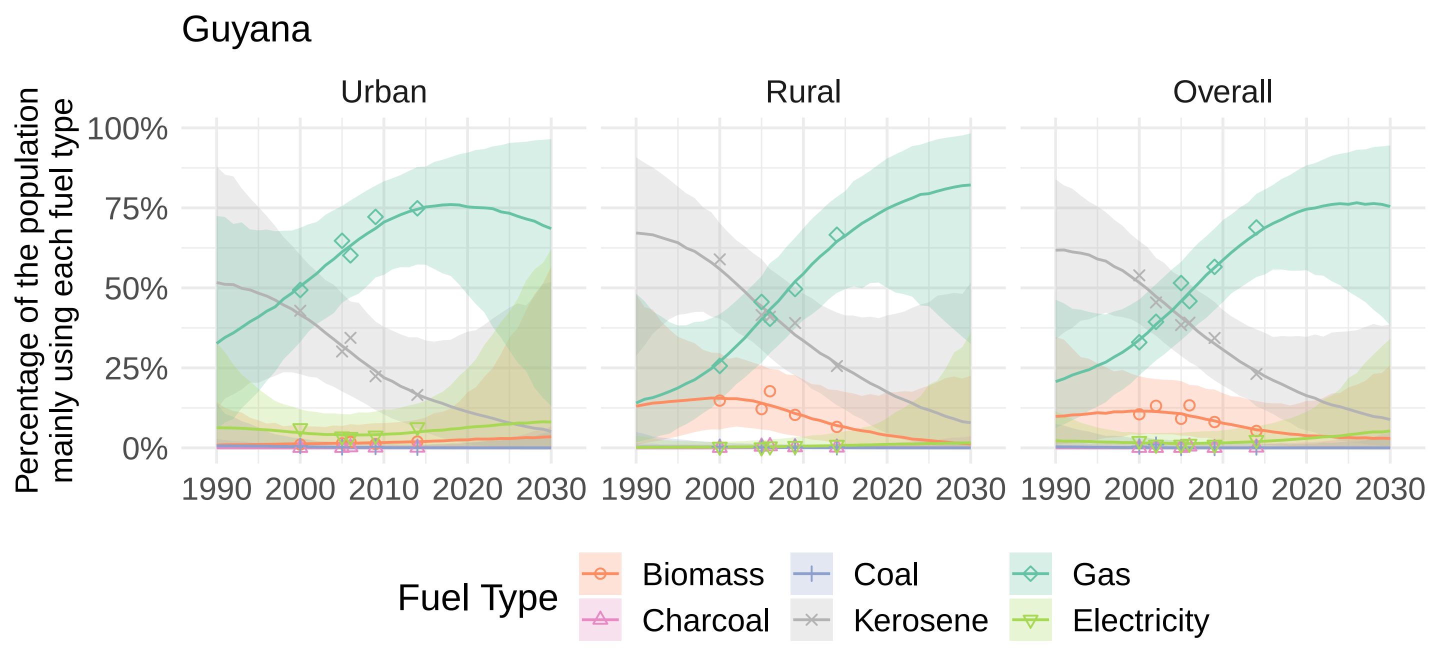

Guyana
Urban
Rural
Overall
Percentage of the population mainly using each fuel type
100%
75%
50%
25%
0%
1990
2000
2010
2020
2030
Fuel Type
Biomass
Charcoal
Coal
Kerosene
Gas
Electricity

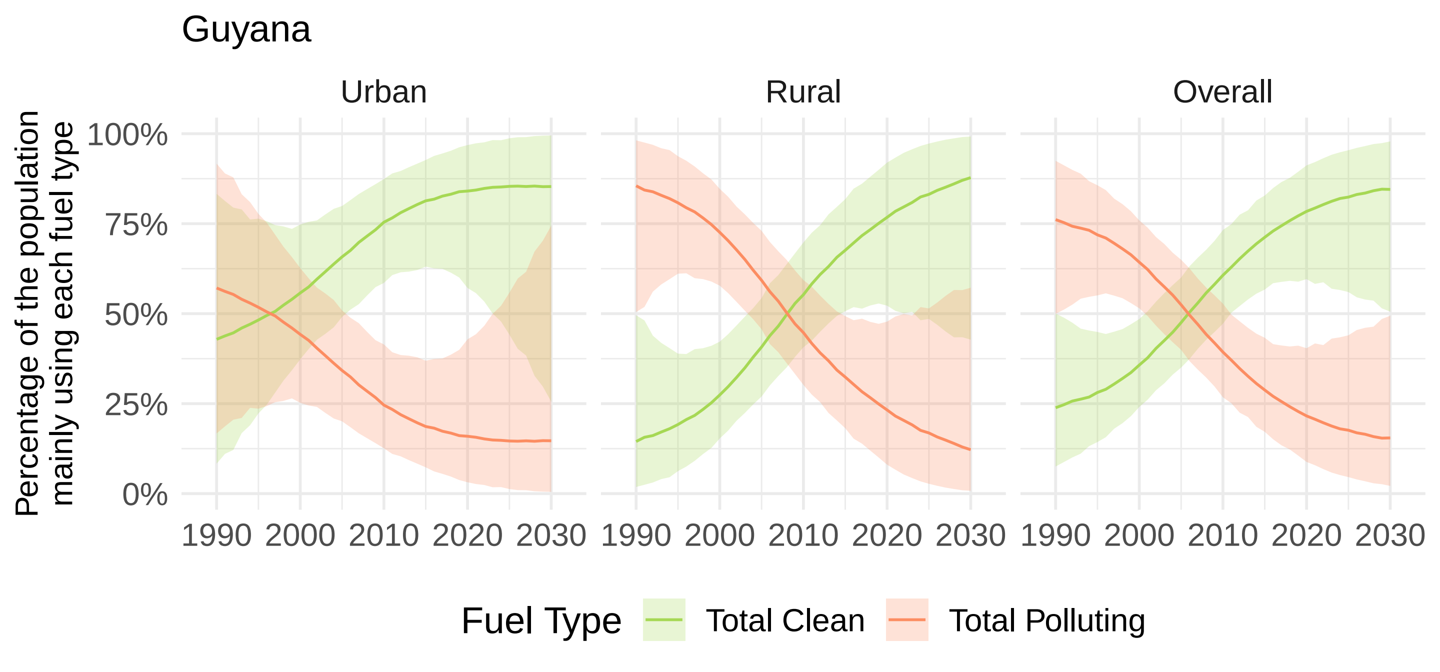

Guyana
Urban
Rural
Overall
Percentage of the population mainly using each fuel type
100%
75%
50%
25%
0%
1990
2000
2010
2020
2030
Fuel Type
Total Clean
Total Polluting

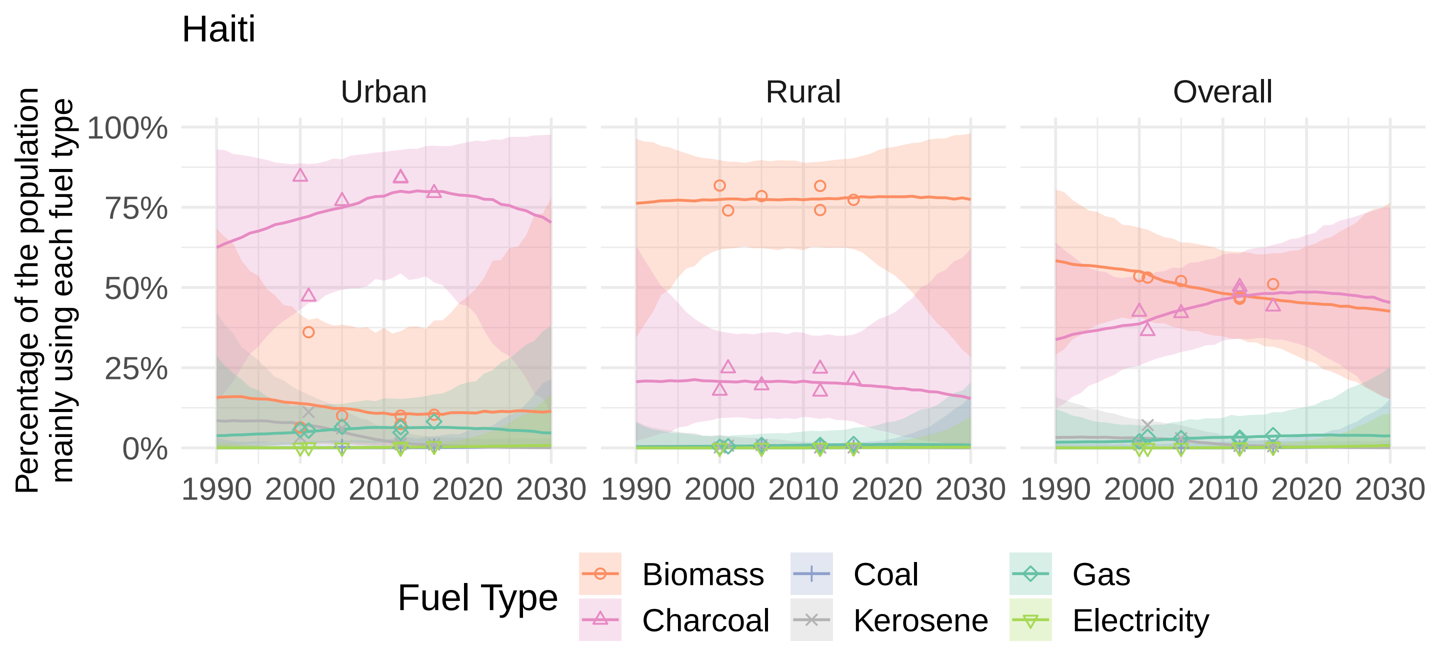

Haiti
Urban
Rural
Overall
Percentage of the population mainly using each fuel type
100%
75%
50%
25%
0%
1990
2000
2010
2020
2030
Fuel Type
Biomass
Charcoal
Coal
Kerosene
Gas
Electricity

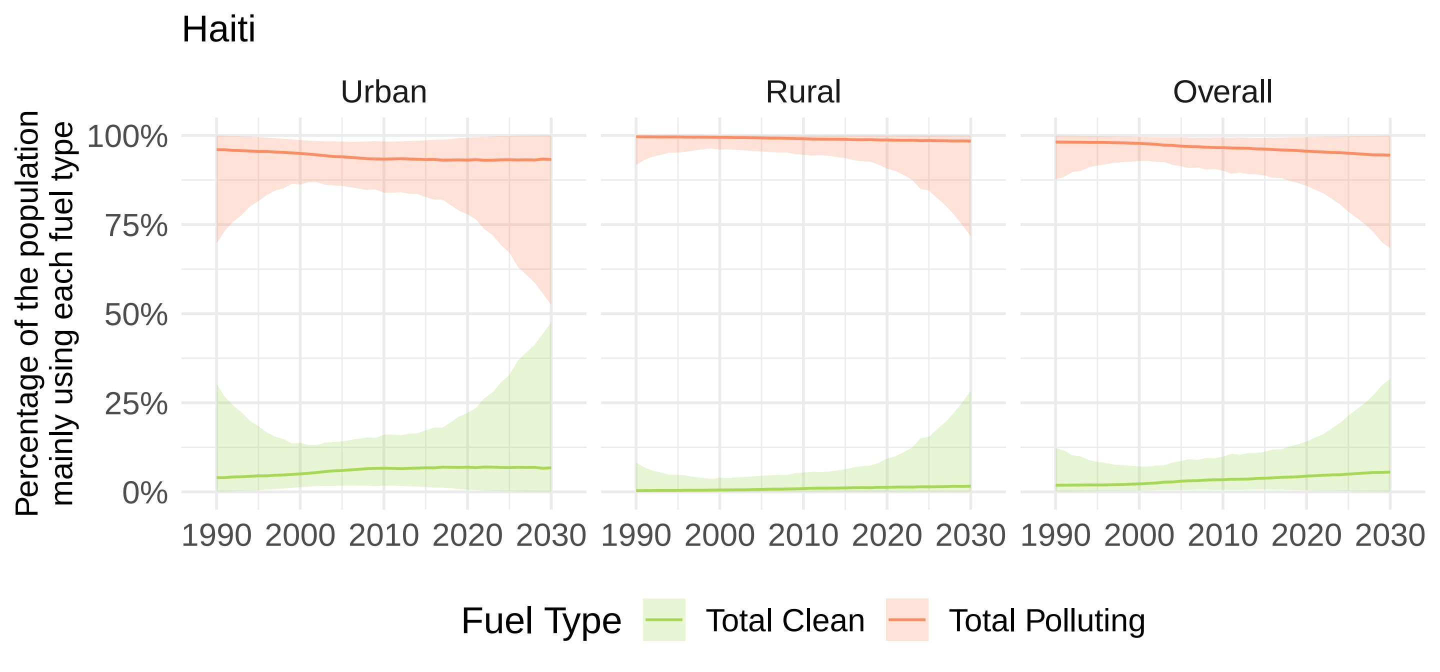

Haiti
Urban
Rural
Overall
Percentage of the population mainly using each fuel type
100%
75%
50%
25%
0%
1990
2000
2010
2020
2030
Fuel Type
Total Clean
Total Polluting

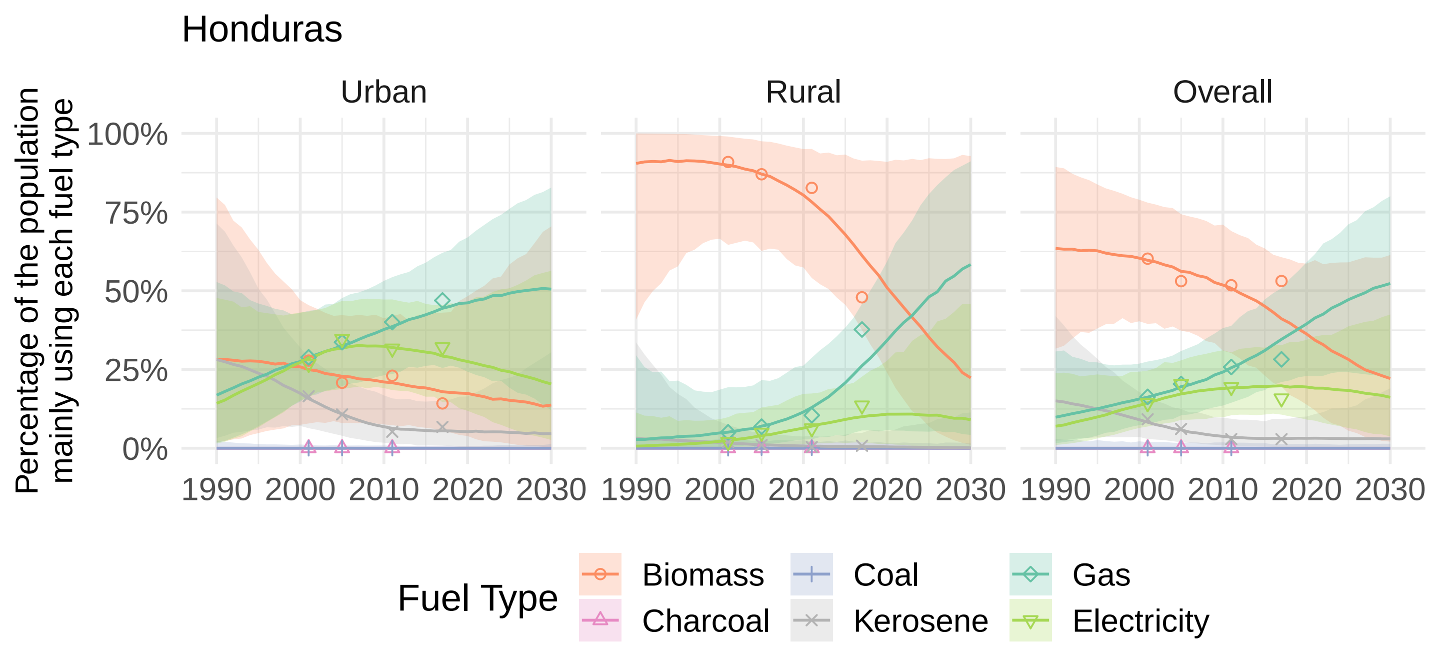
Honduras
Urban
Rural
Overall
Percentage of the population mainly using each fuel type
100%
75%
50%
25%
0%
1990
2000
2010
2020
2030
Fuel Type
Biomass
Charcoal
Coal
Kerosene
Gas
Electricity

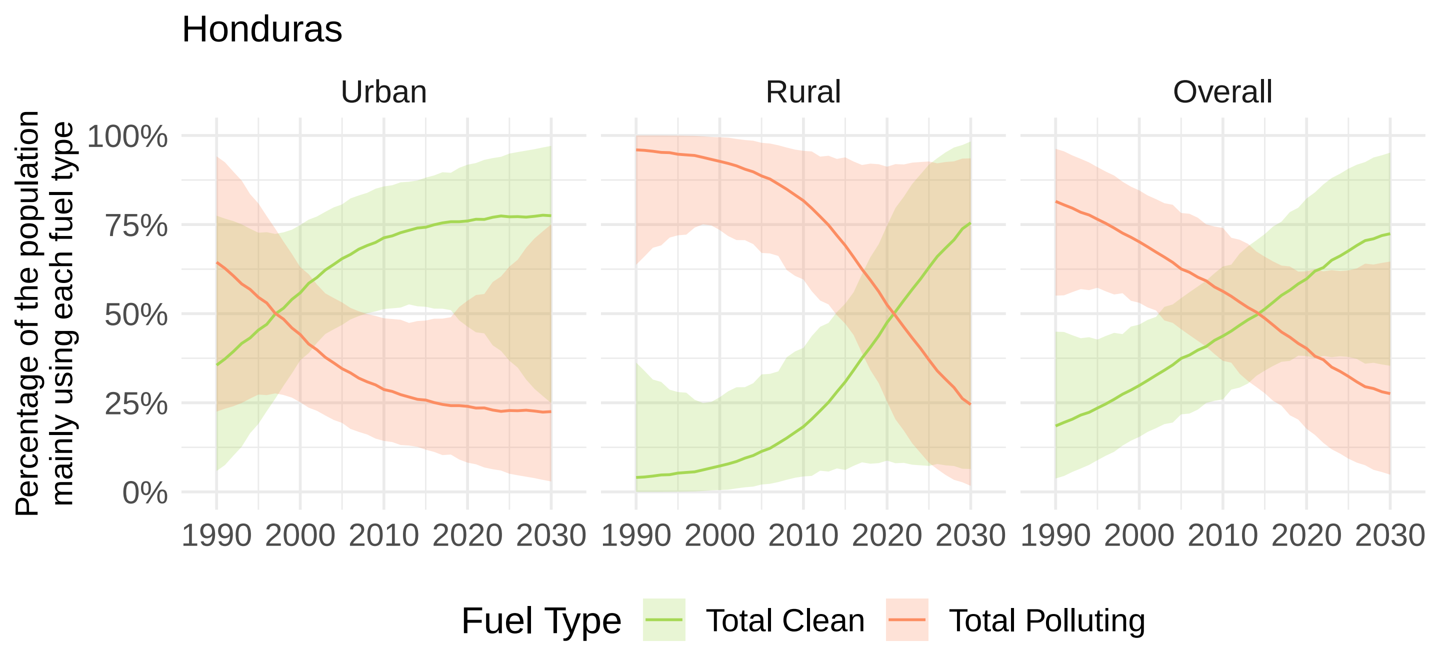

Honduras
Urban
Rural
Overall
Percentage of the population mainly using each fuel type
100%
75%
50%
25%
0%
1990
2000
2010
2020
2030
Fuel Type
Total Clean
Total Polluting

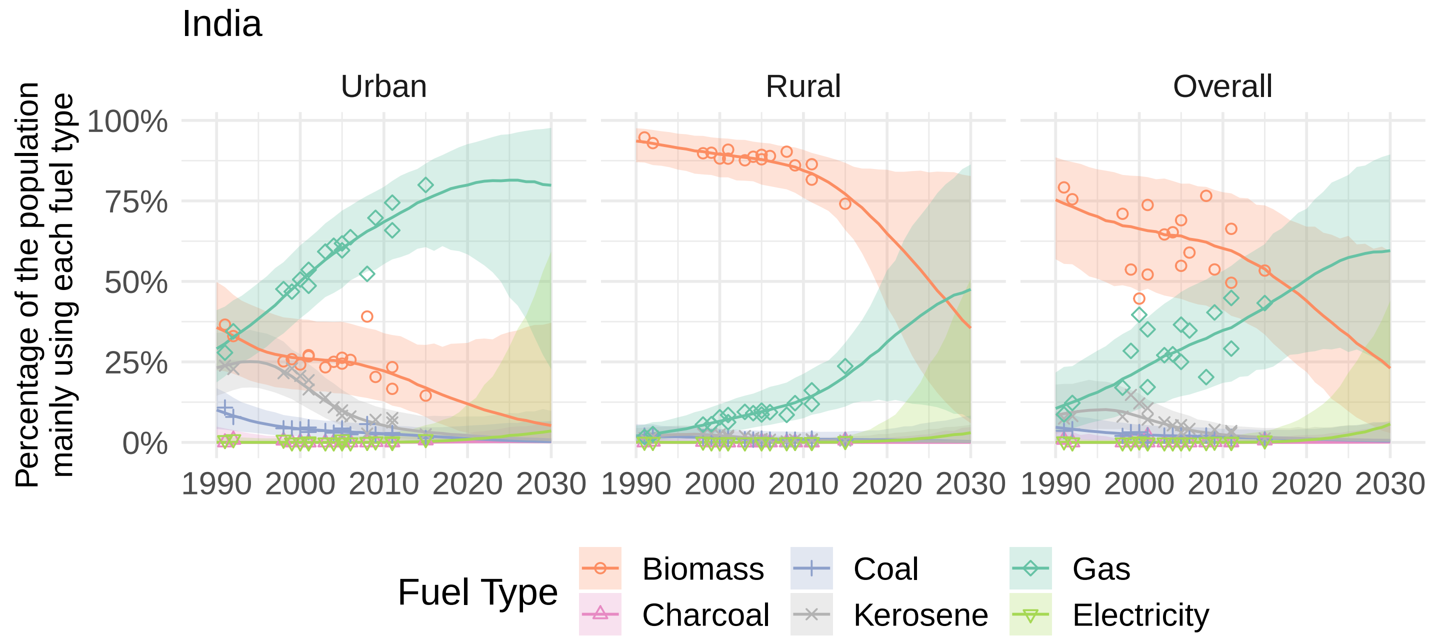
India
Urban
Rural
Overall
Percentage of the population mainly using each fuel type
100%
75%
50%
25%
0%
1990
2000
2010
2020
2030
Fuel Type
Biomass
Charcoal
Coal
Kerosene
Gas
Electricity

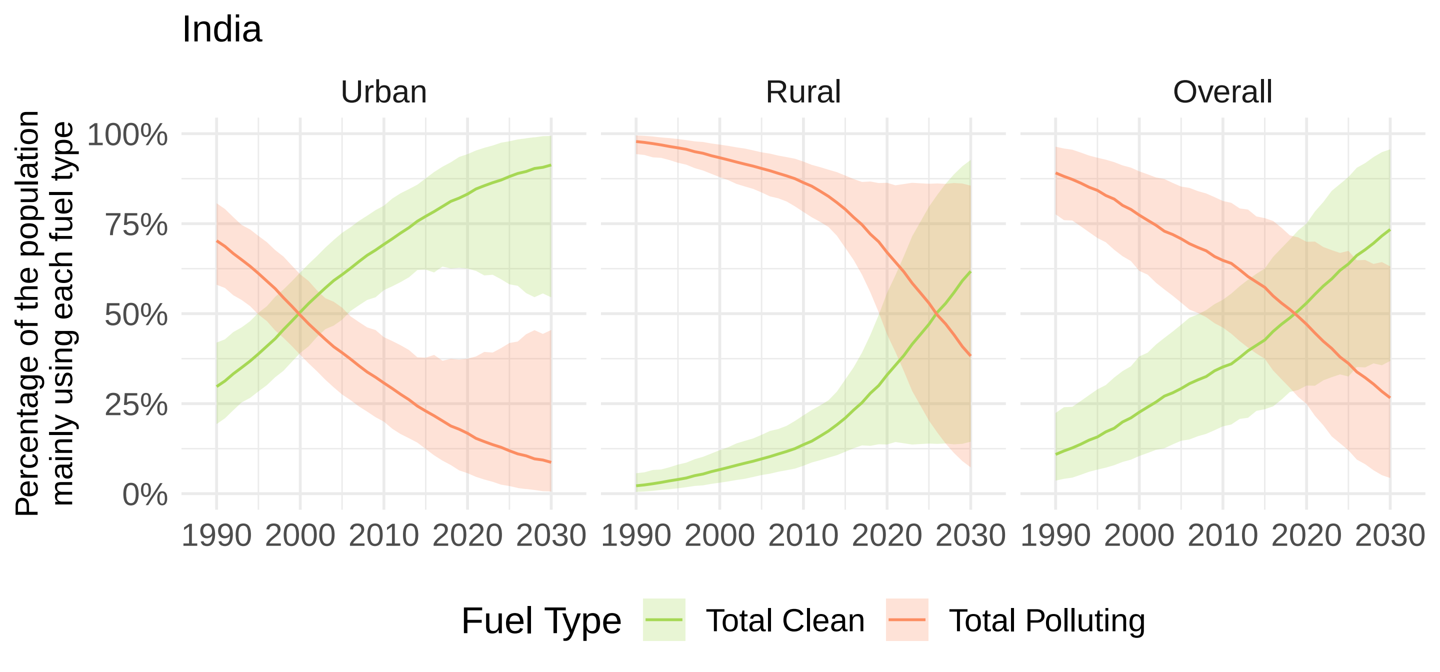

India
Urban
Rural
Overall
Percentage of the population mainly using each fuel type
100%
75%
50%
25%
0%
1990
2000
2010
2020
2030
Fuel Type
Total Clean
Total Polluting

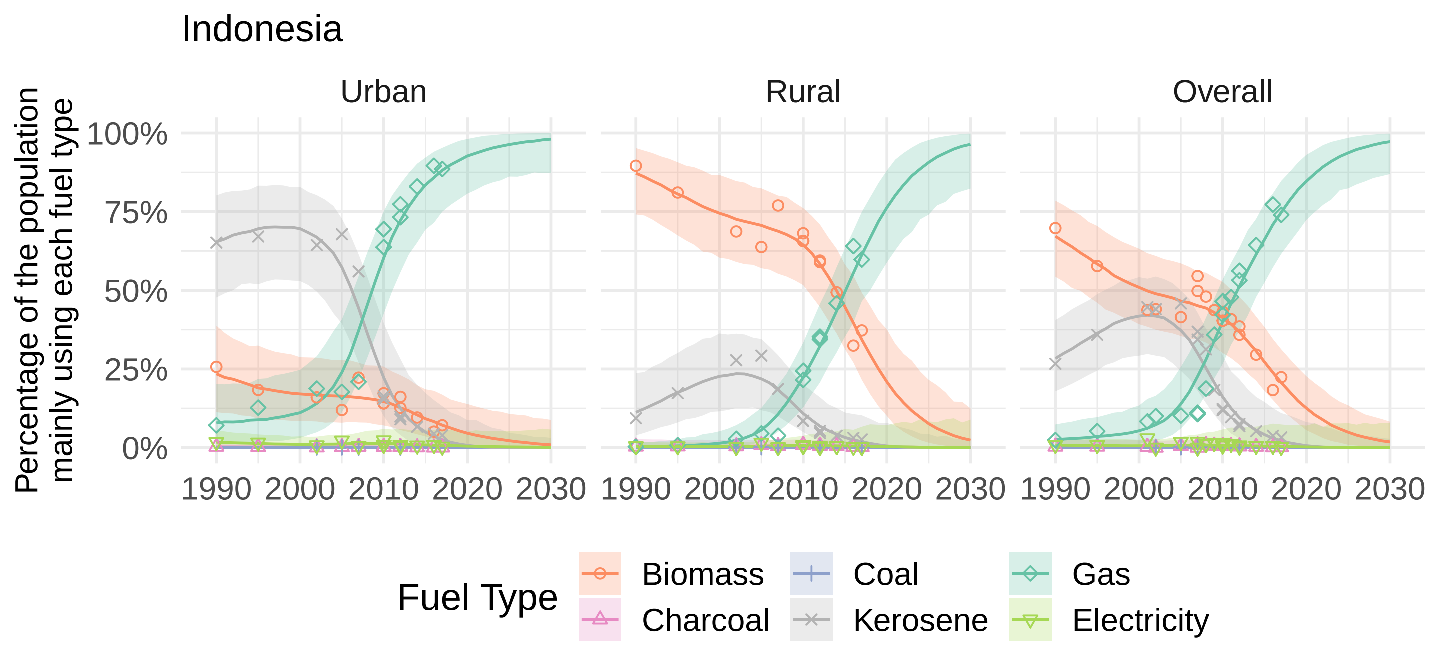

Indonesia
Urban
Rural
Overall
Percentage of the population mainly using each fuel type
100%
75%
50%
25%
0%
1990
2000
2010
2020
2030
Fuel Type
Biomass
Charcoal
Coal
Kerosene
Gas
Electricity

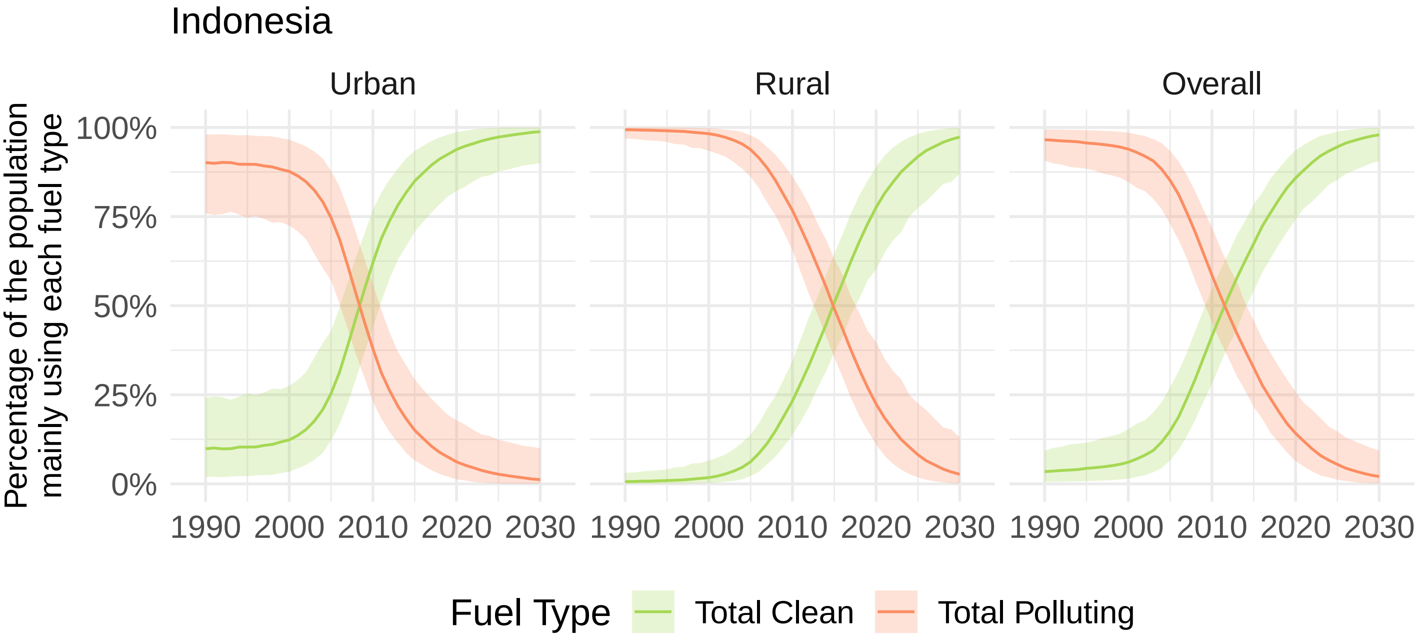
Indonesia
Urban
Rural
Overall
Percentage of the population mainly using each fuel type
100%
75%
50%
25%
0%
1990
2000
2010
2020
2030
Fuel Type
Total Clean
Total Polluting

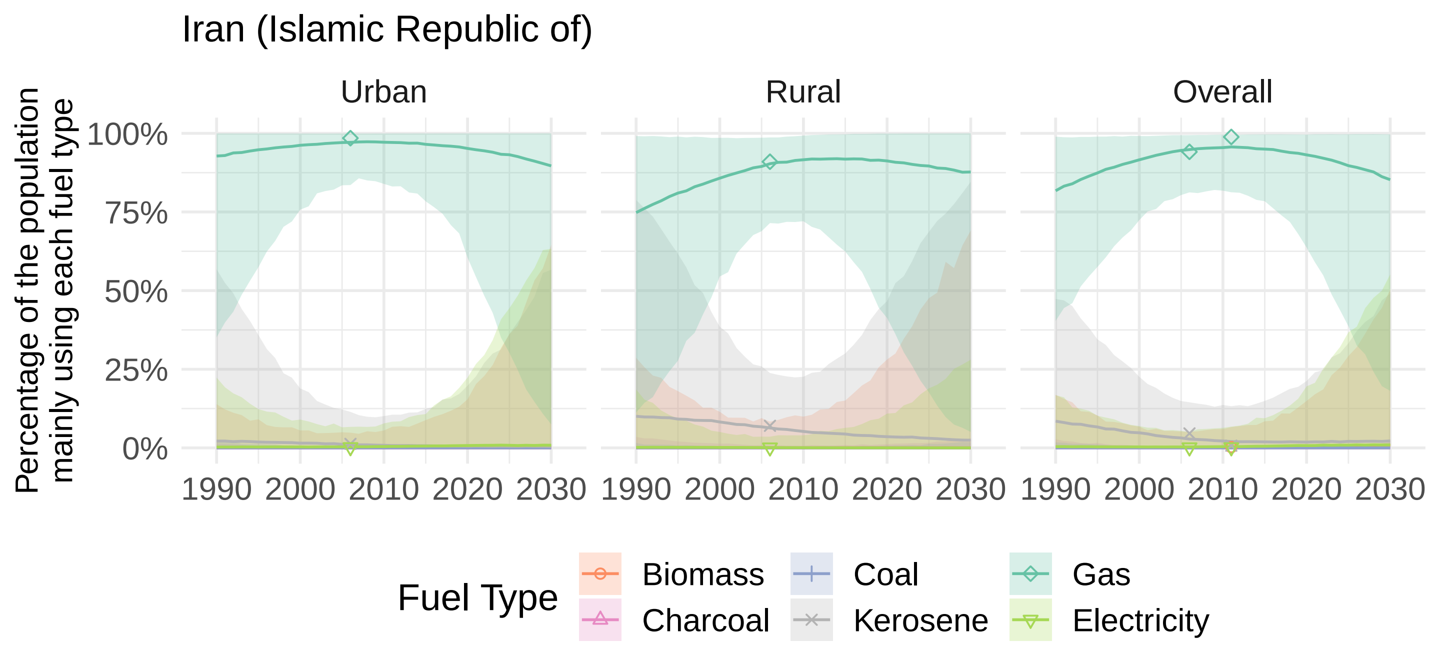

Iran (Islamic Republic of)
Urban
Rural
Overall
Percentage of the population mainly using each fuel type
100%
75%
50%
25%
0%
1990
2000
2010
2020
2030
Fuel Type
Biomass
Charcoal
Coal
Kerosene
Gas
Electricity

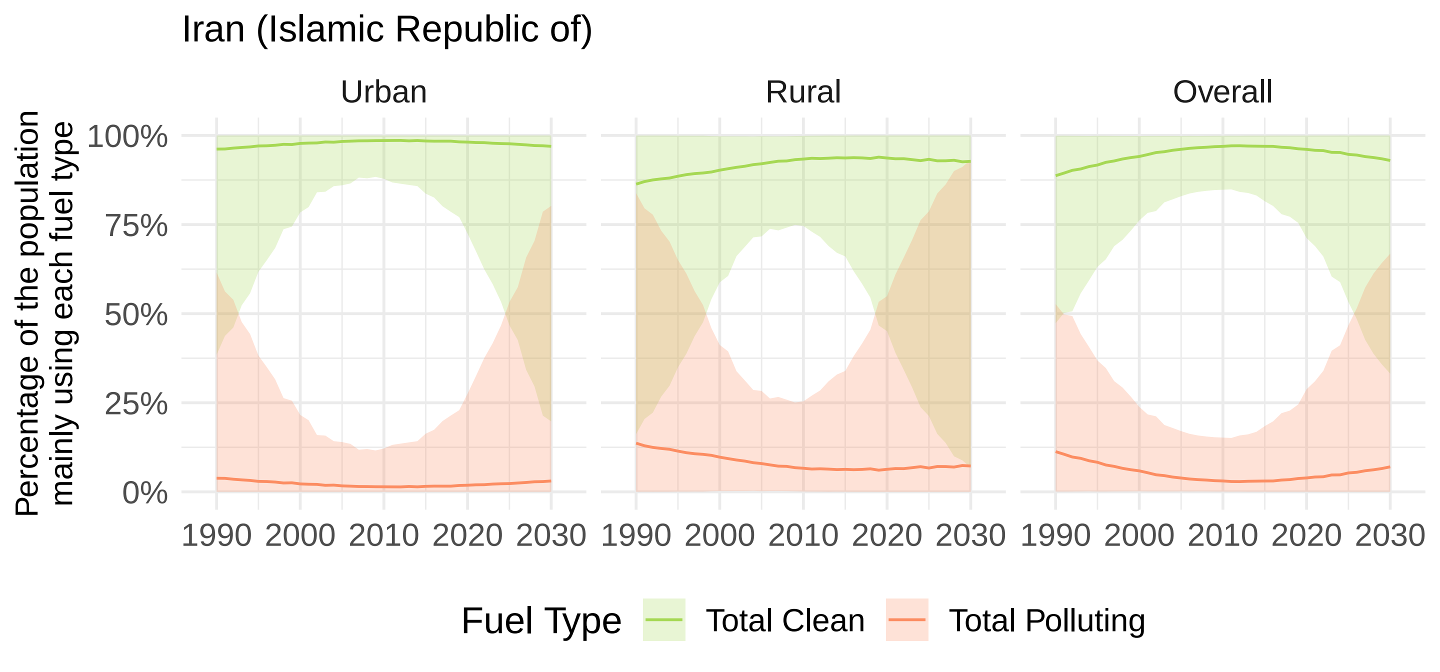
Iran (Islamic Republic of)
Urban
Rural
Overall
Percentage of the population mainly using each fuel type
100%
75%
50%
25%
0%
1990
2000
2010
2020
2030
Fuel Type
Total Clean
Total Polluting

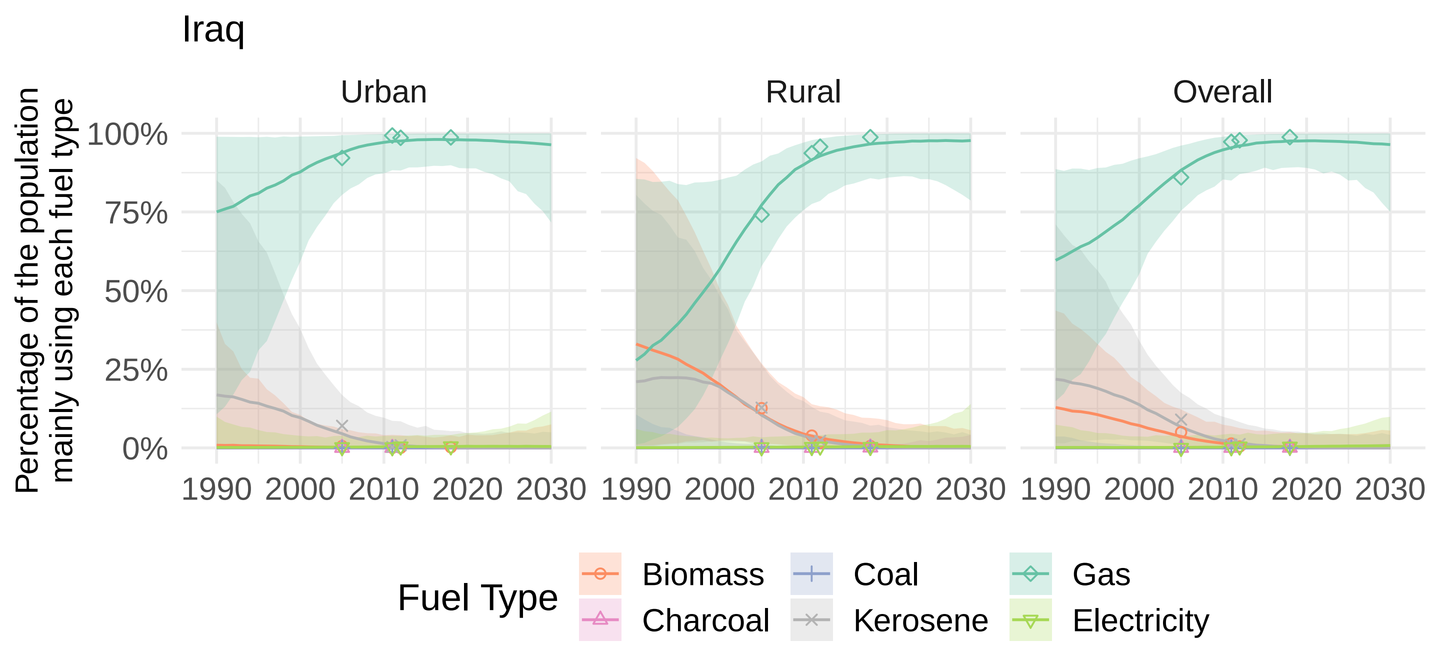

Iraq
Urban
Rural
Overall
Percentage of the population mainly using each fuel type
100%
75%
50%
25%
0%
1990
2000
2010
2020
2030
Fuel Type
Biomass
Charcoal
Coal
Kerosene
Gas
Electricity

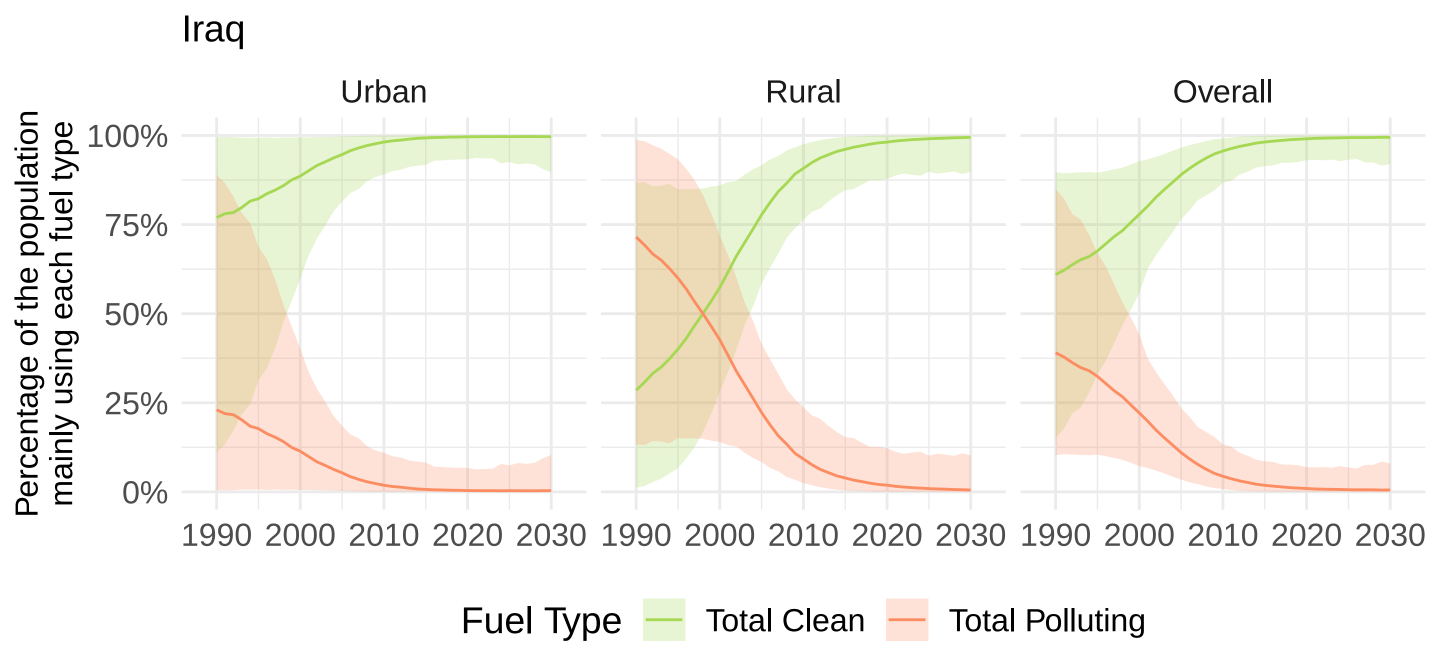

Iraq
Urban
Rural
Overall
Percentage of the population mainly using each fuel type
100%
75%
50%
25%
0%
1990
2000
2010
2020
2030
Fuel Type
Total Clean
Total Polluting

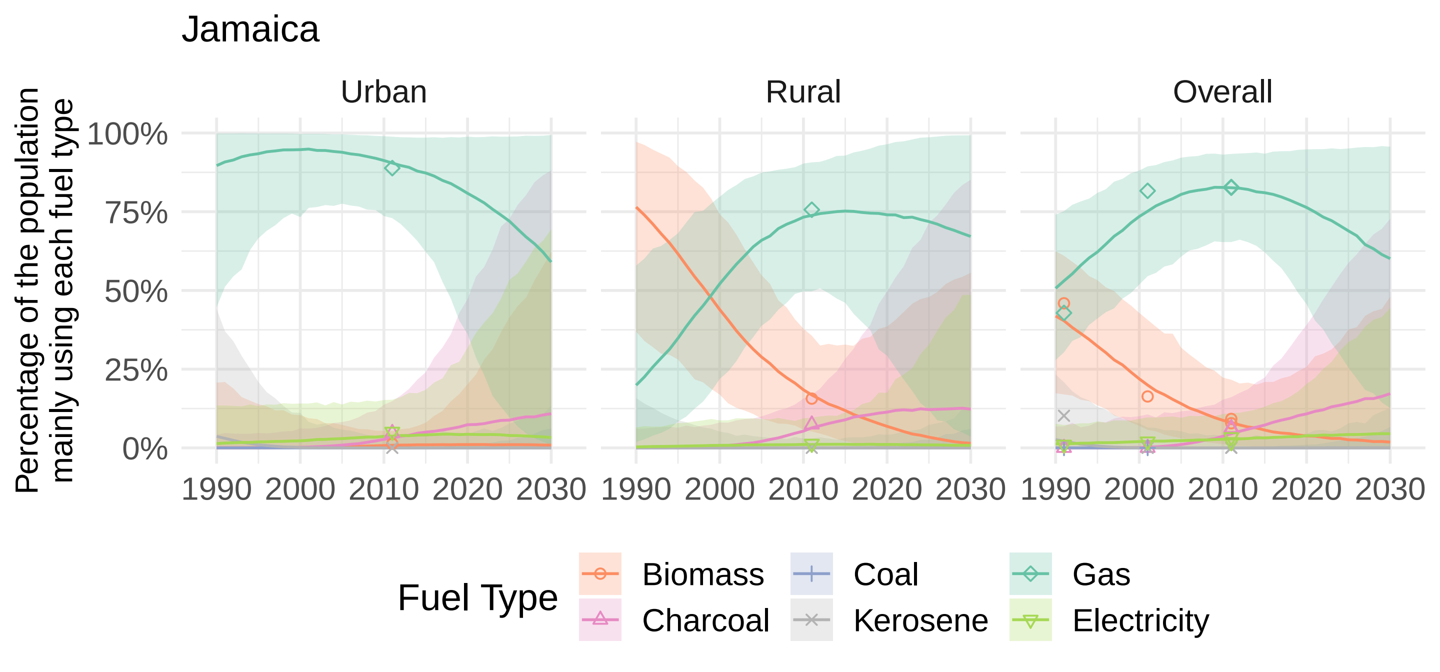

Jamaica
Urban
Rural
Overall
Percentage of the population mainly using each fuel type
100%
75%
50%
25%
0%
1990
2000
2010
2020
2030
Fuel Type
Biomass
Charcoal
Coal
Kerosene
Gas
Electricity

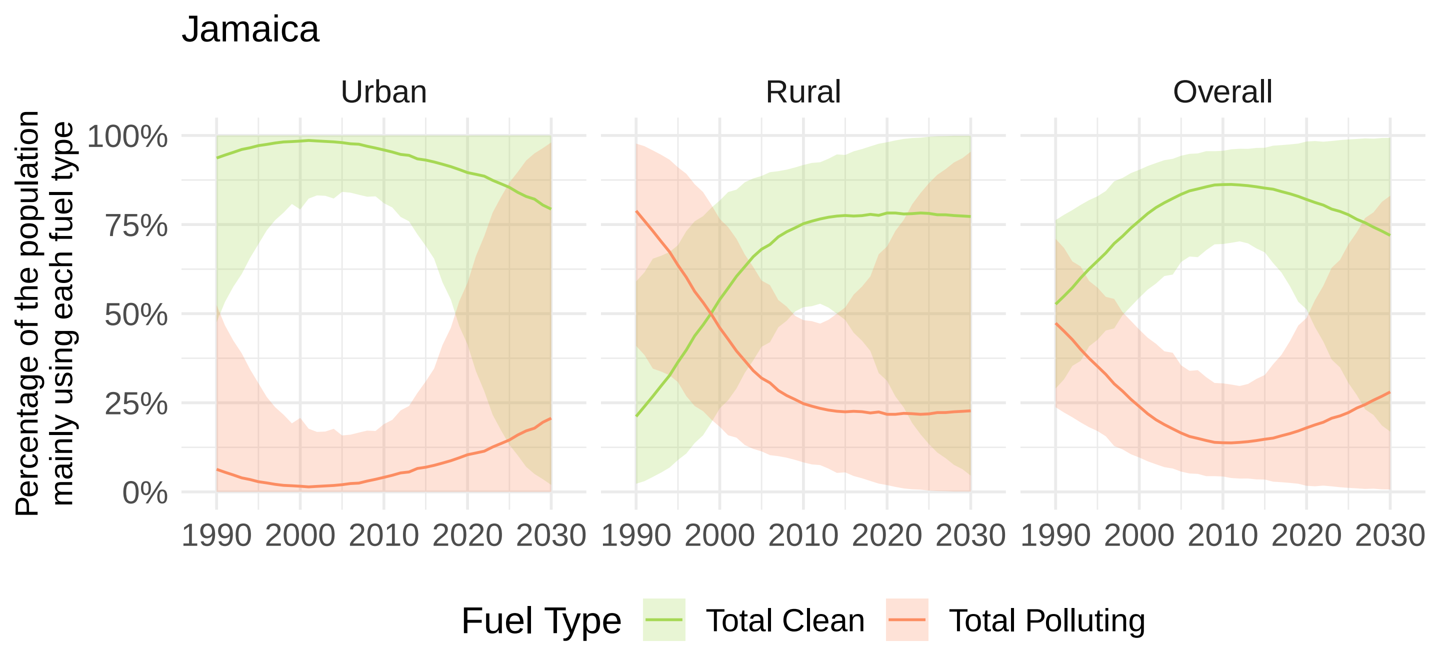
Jamaica
Urban
Rural
Overall
Percentage of the population mainly using each fuel type
100%
75%
50%
25%
0%
1990
2000
2010
2020
2030
Fuel Type
Total Clean
Total Polluting

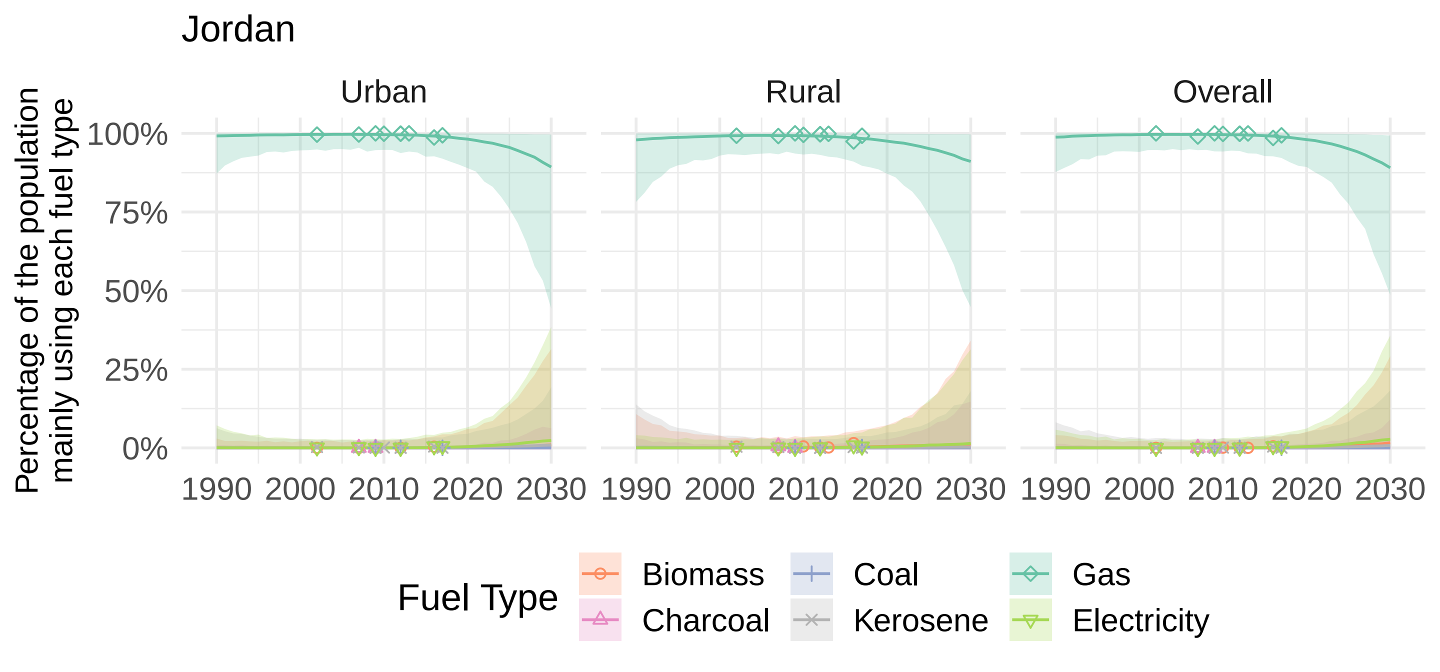
Jordan
Urban
Rural
Overall
Percentage of the population mainly using each fuel type
100%
75%
50%
25%
0%
1990
2000
2010
2020
2030
Fuel Type
Biomass
Charcoal
Coal
Kerosene
Gas
Electricity

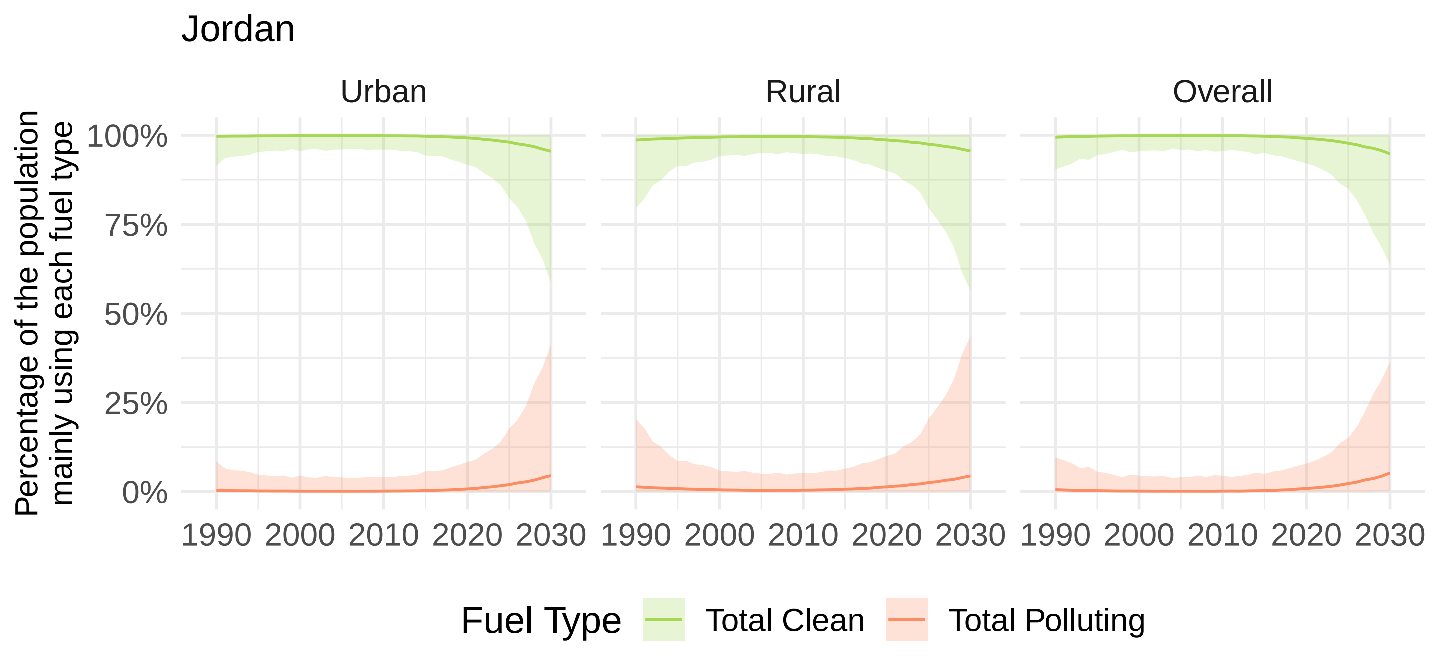
Jordan
Urban
Rural
Overall
Percentage of the population mainly using each fuel type
100%
75%
50%
25%
0%
1990
2000
2010
2020
2030
Fuel Type
Total Clean
Total Polluting

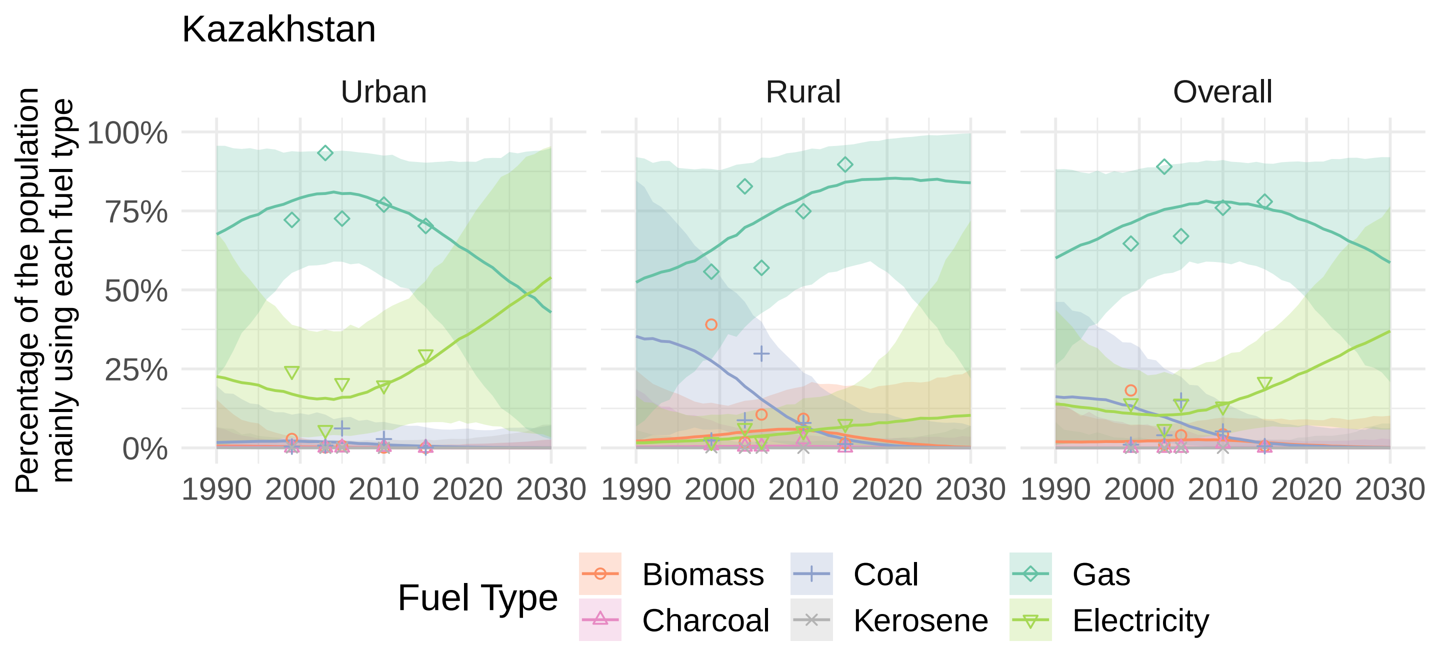

Kazakhstan
Urban
Rural
Overall
Percentage of the population mainly using each fuel type
100%
75%
50%
25%
0%
1990
2000
2010
2020
2030
Fuel Type
Biomass
Charcoal
Coal
Kerosene
Gas
Electricity

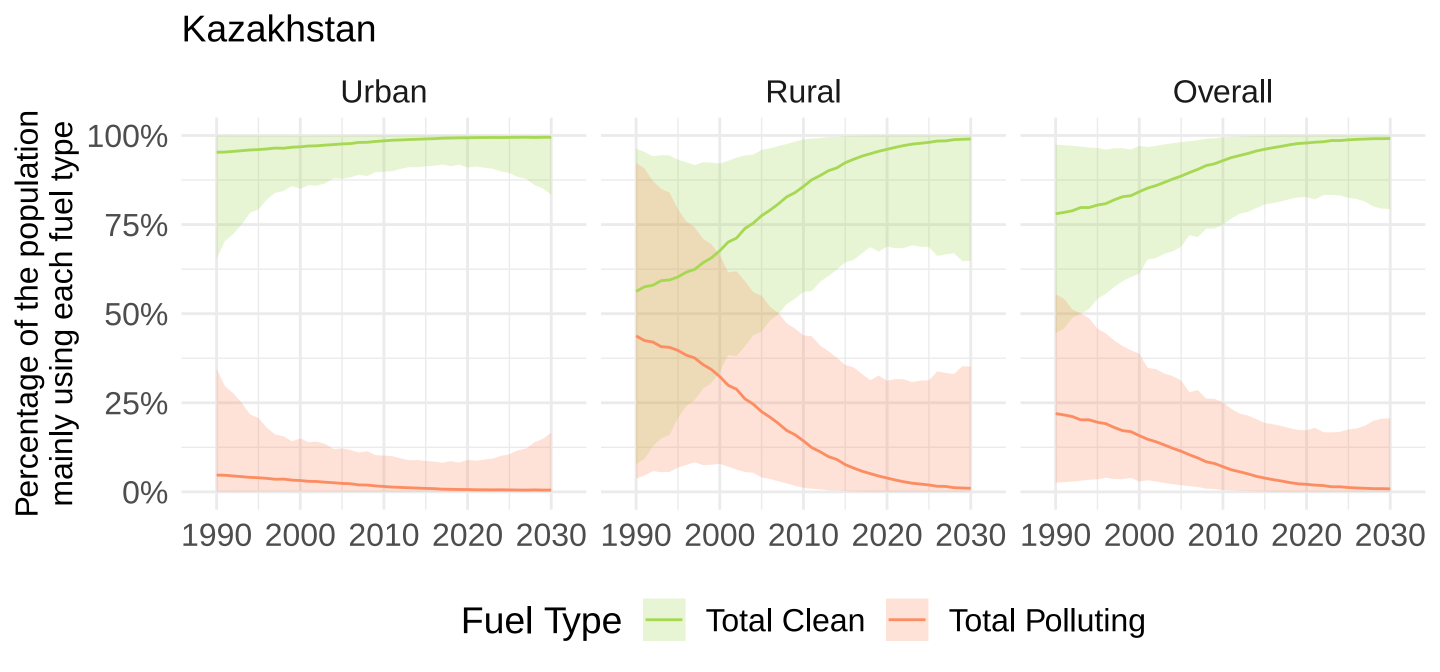

Kazakhstan
Urban
Rural
Overall
Percentage of the population mainly using each fuel type
100%
75%
50%
25%
0%
1990
2000
2010
2020
2030
Fuel Type
Total Clean
Total Polluting

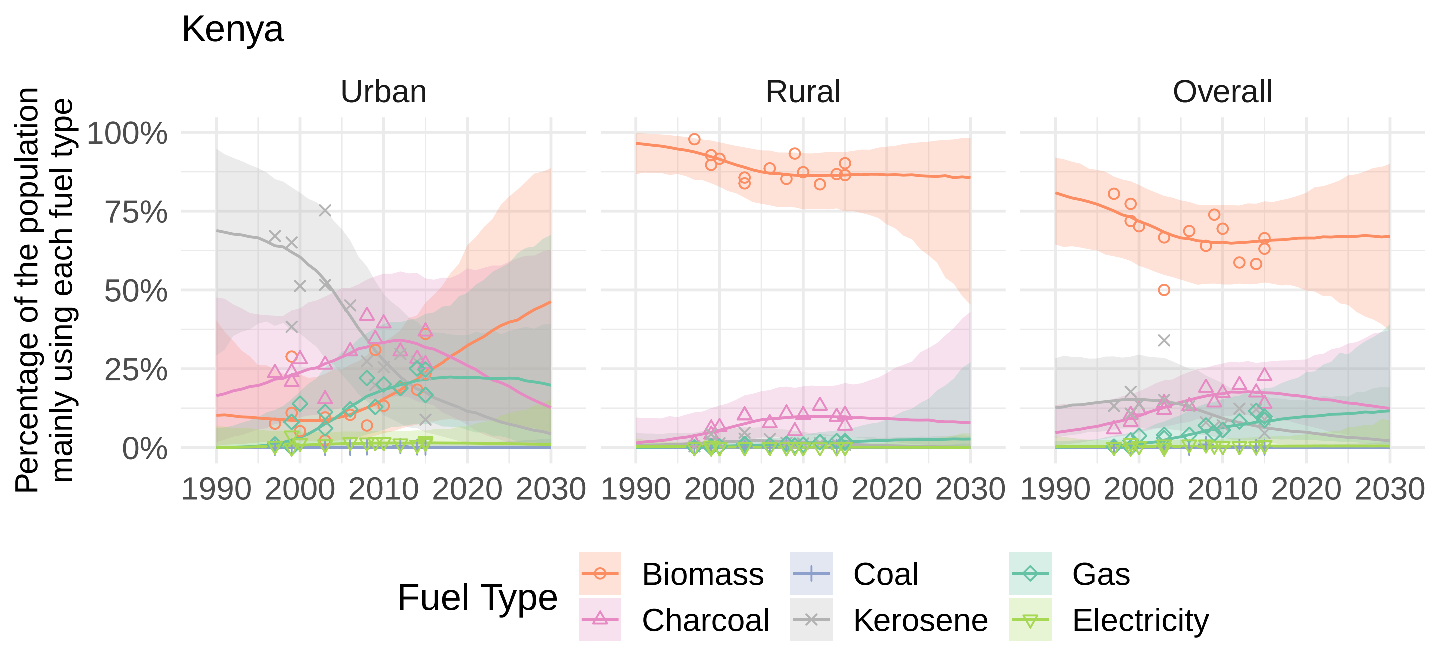

Kenya
Urban
Rural
Overall
Percentage of the population mainly using each fuel type
100%
75%
50%
25%
0%
1990
2000
2010
2020
2030
Fuel Type
Biomass
Charcoal
Coal
Kerosene
Gas
Electricity

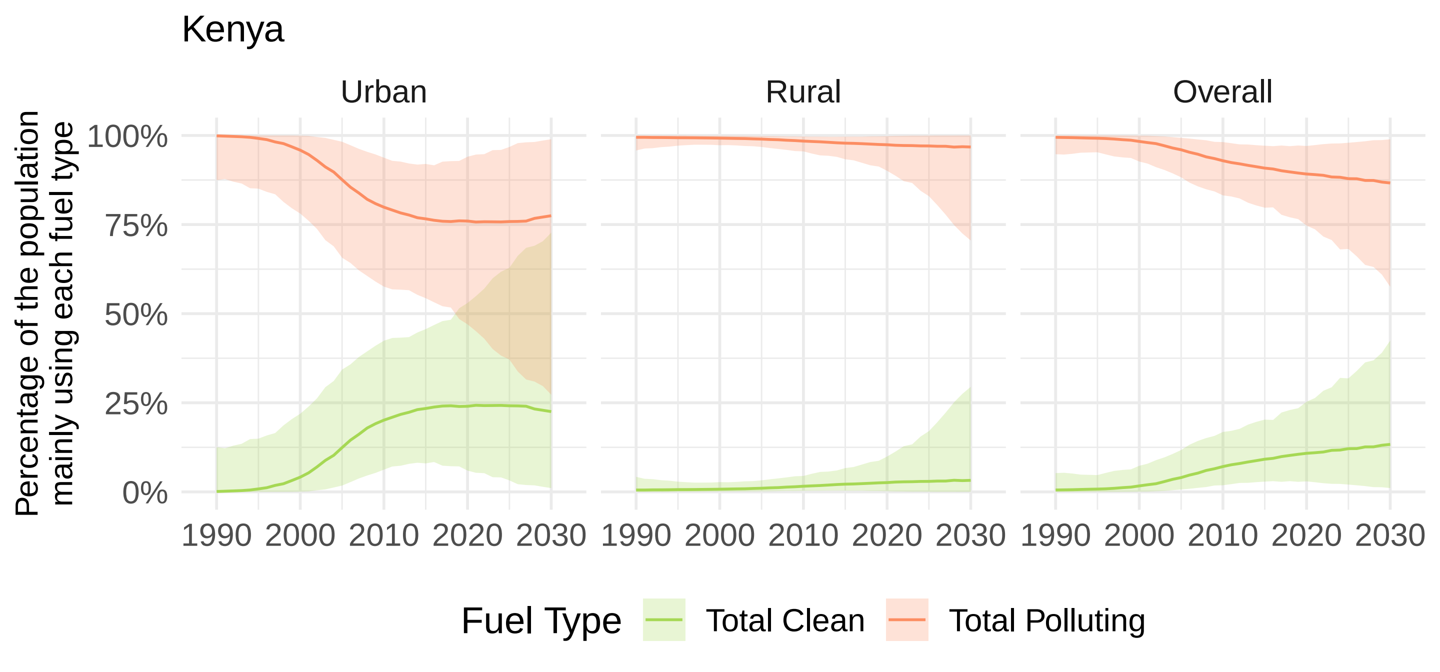

Kenya
Urban
Rural
Overall
Percentage of the population mainly using each fuel type
100%
75%
50%
25%
0%
1990
2000
2010
2020
2030
Fuel Type
Total Clean
Total Polluting

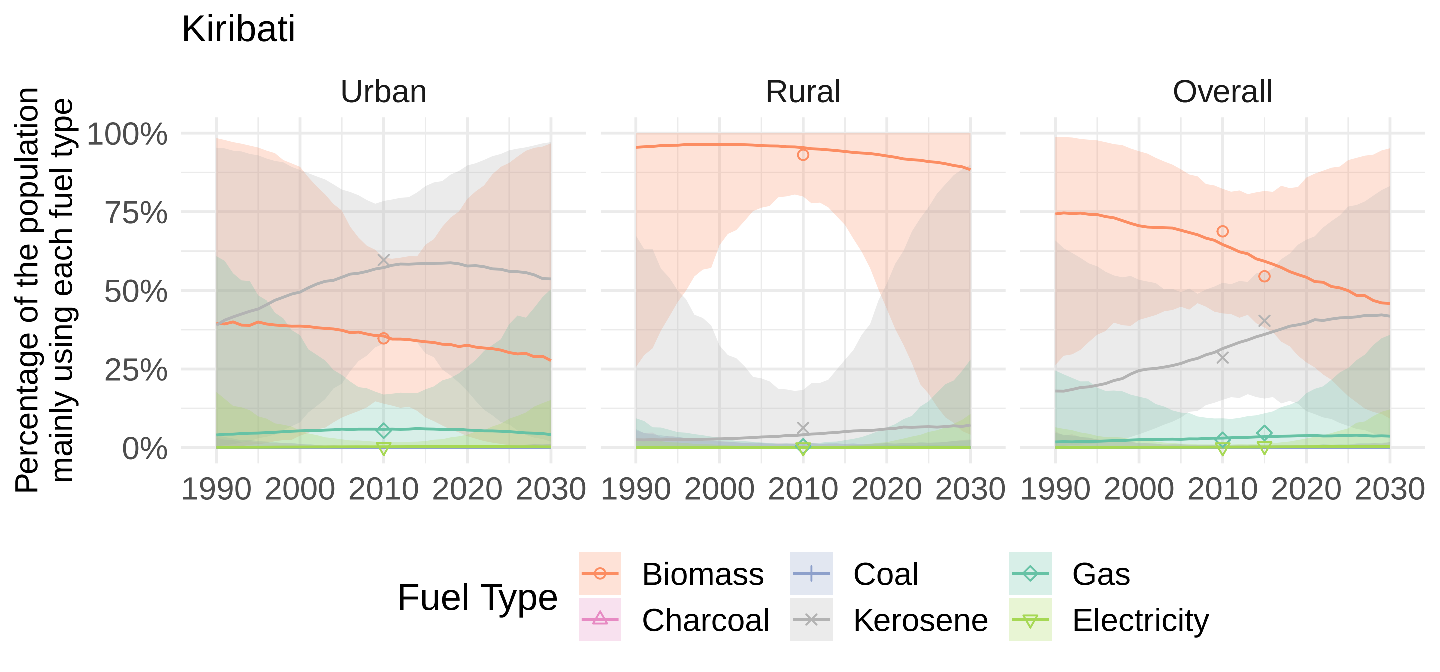
Kiribati
Urban
Rural
Overall
Percentage of the population mainly using each fuel type
100%
75%
50%
25%
0%
1990
2000
2010
2020
2030
Fuel Type
Biomass
Charcoal
Coal
Kerosene
Gas
Electricity

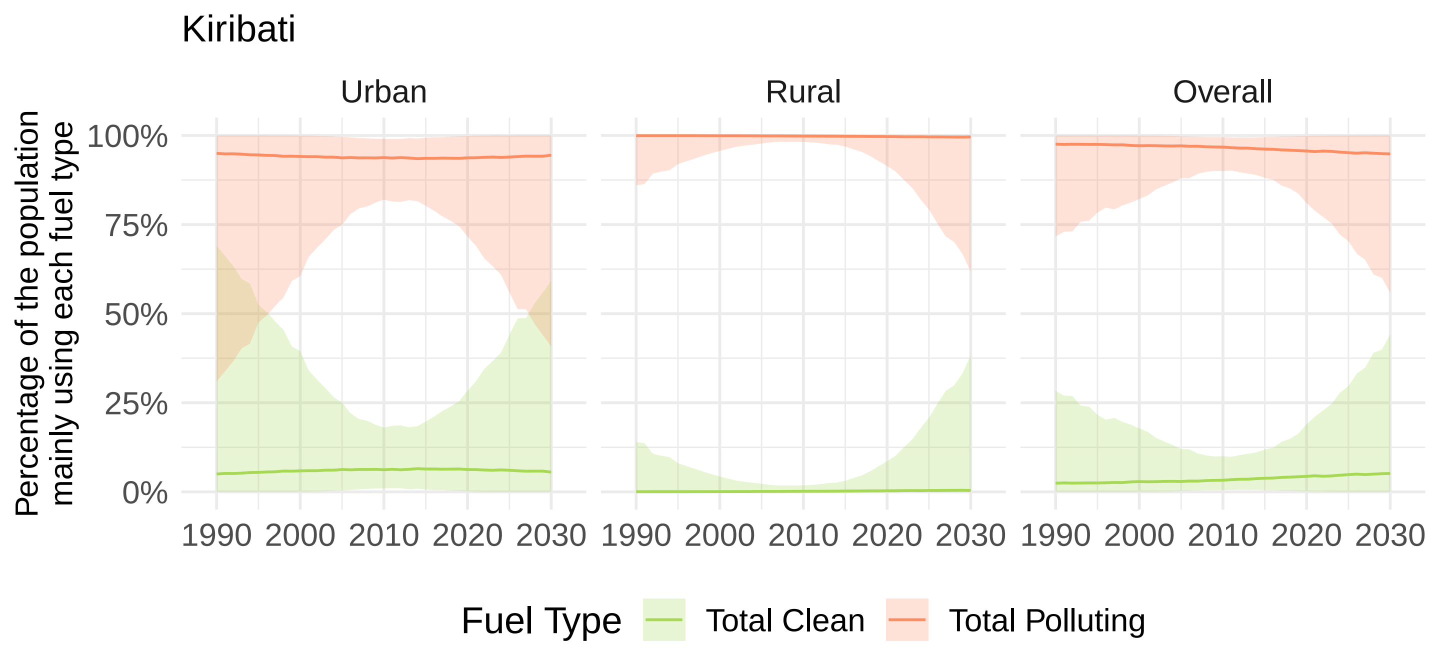
Kiribati
Urban
Rural
Overall
Percentage of the population mainly using each fuel type
100%
75%
50%
25%
0%
1990
2000
2010
2020
2030
Fuel Type
Total Clean
Total Polluting

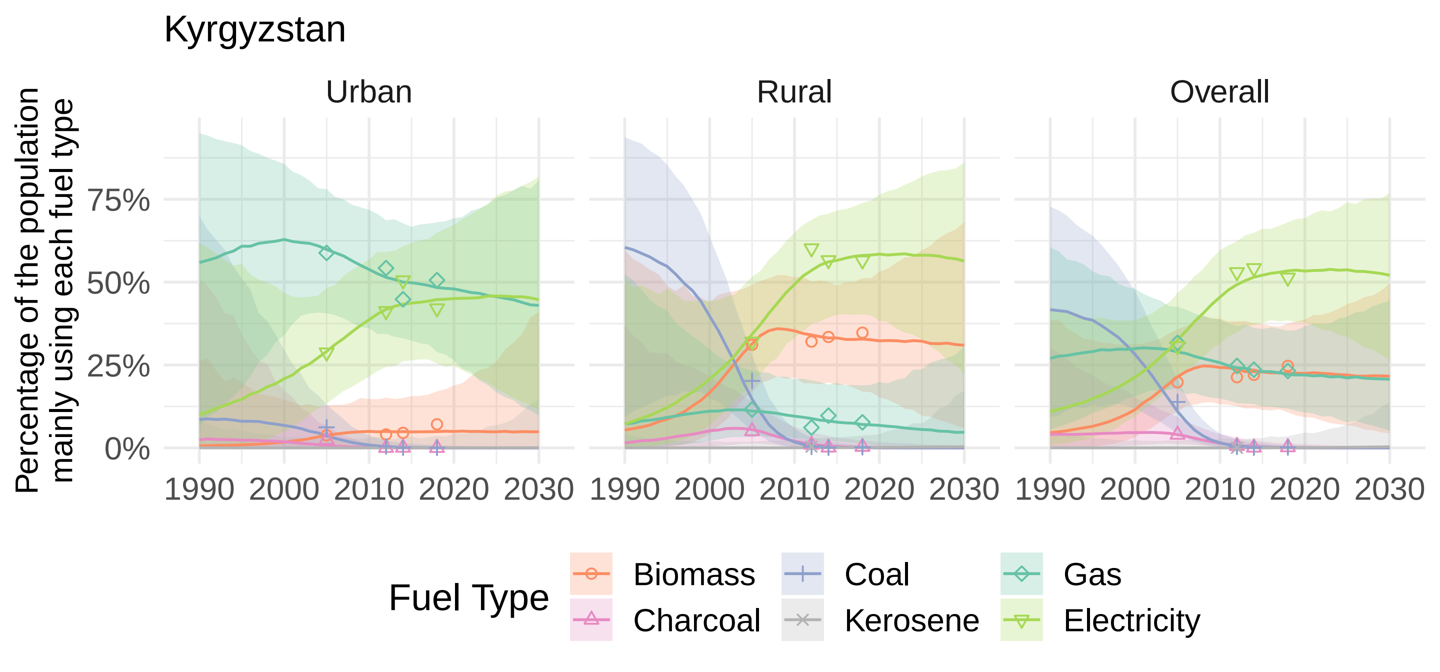
Kyrgyzstan
Urban
Rural
Overall
Percentage of the population mainly using each fuel type
0%
25%
50%
75%
1990
2000
2010
2020
2030
Fuel Type
Biomass
Charcoal
Coal
Kerosene
Gas
Electricity

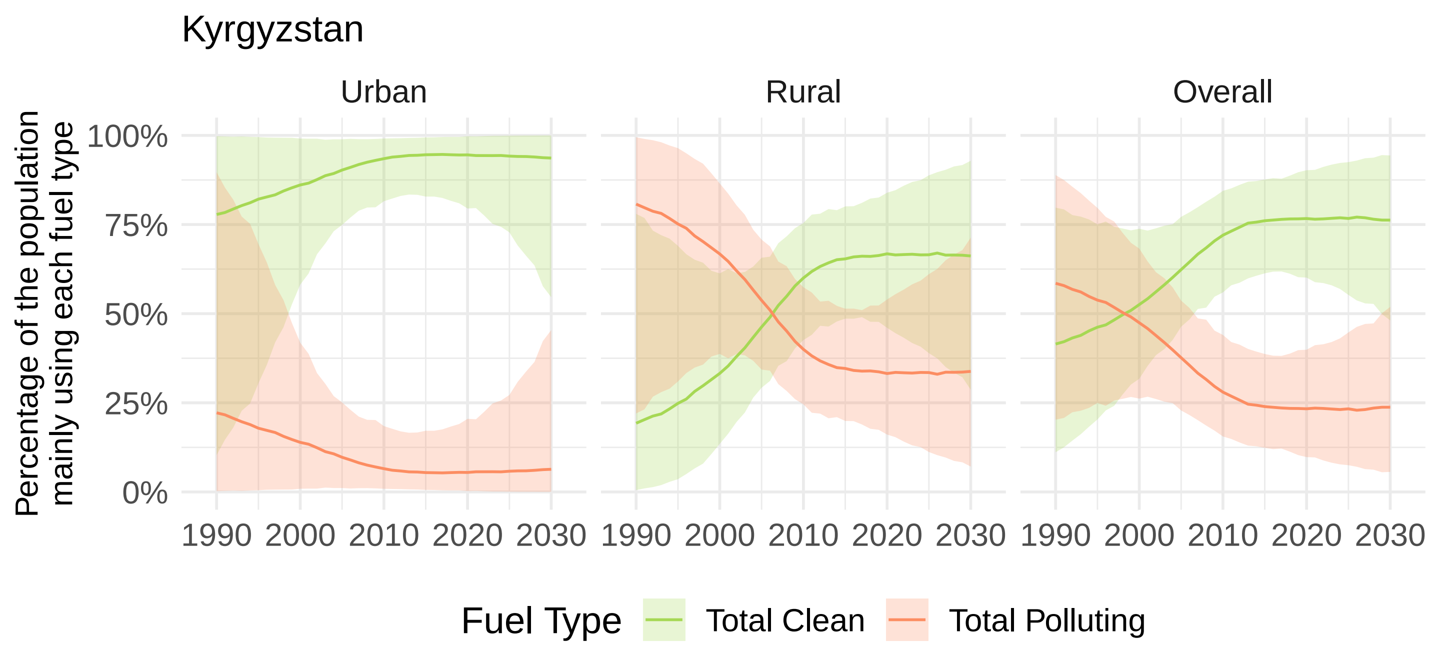

Kyrgyzstan
Urban
Rural
Overall
Percentage of the population mainly using each fuel type
100%
75%
50%
25%
0%
1990
2000
2010
2020
2030
Fuel Type
Total Clean
Total Polluting

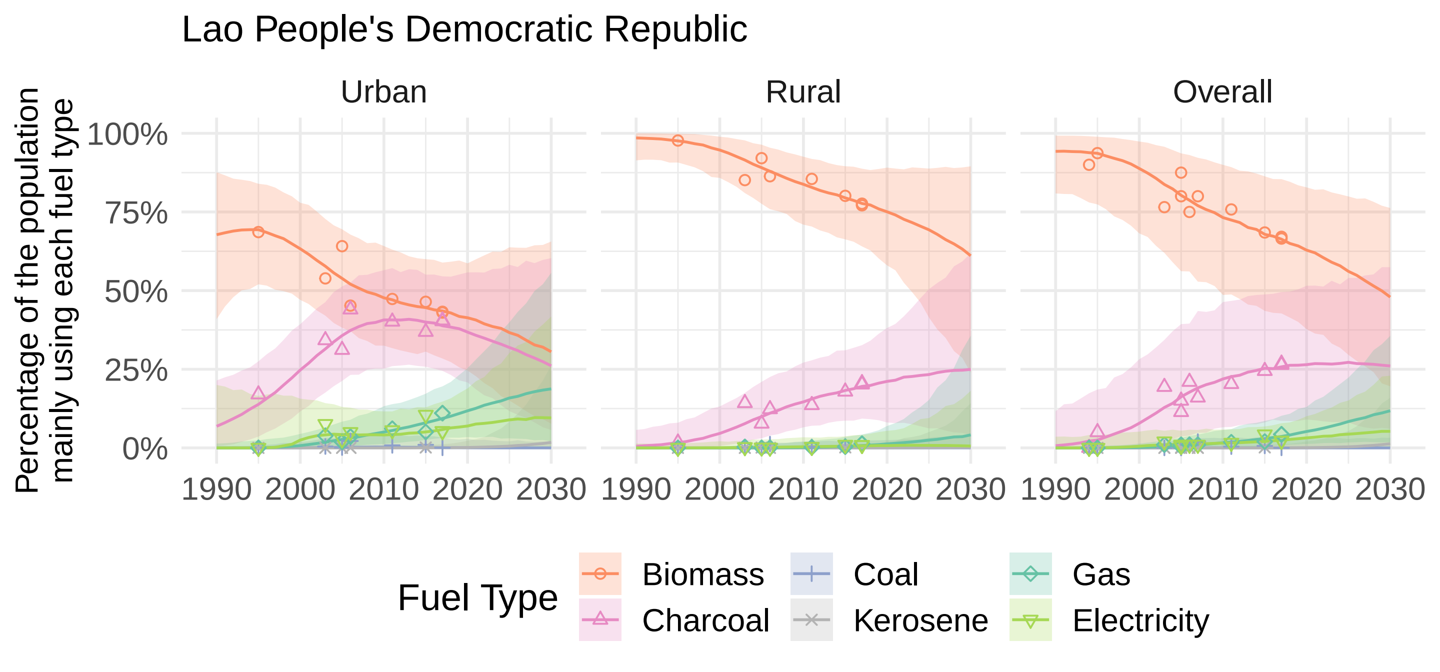
Lao People's Democratic Republic
Urban
Rural
Overall
Percentage of the population mainly using each fuel type
100%
75%
50%
25%
0%
1990
2000
2010
2020
2030
Fuel Type
Biomass
Charcoal
Coal
Kerosene
Gas
Electricity

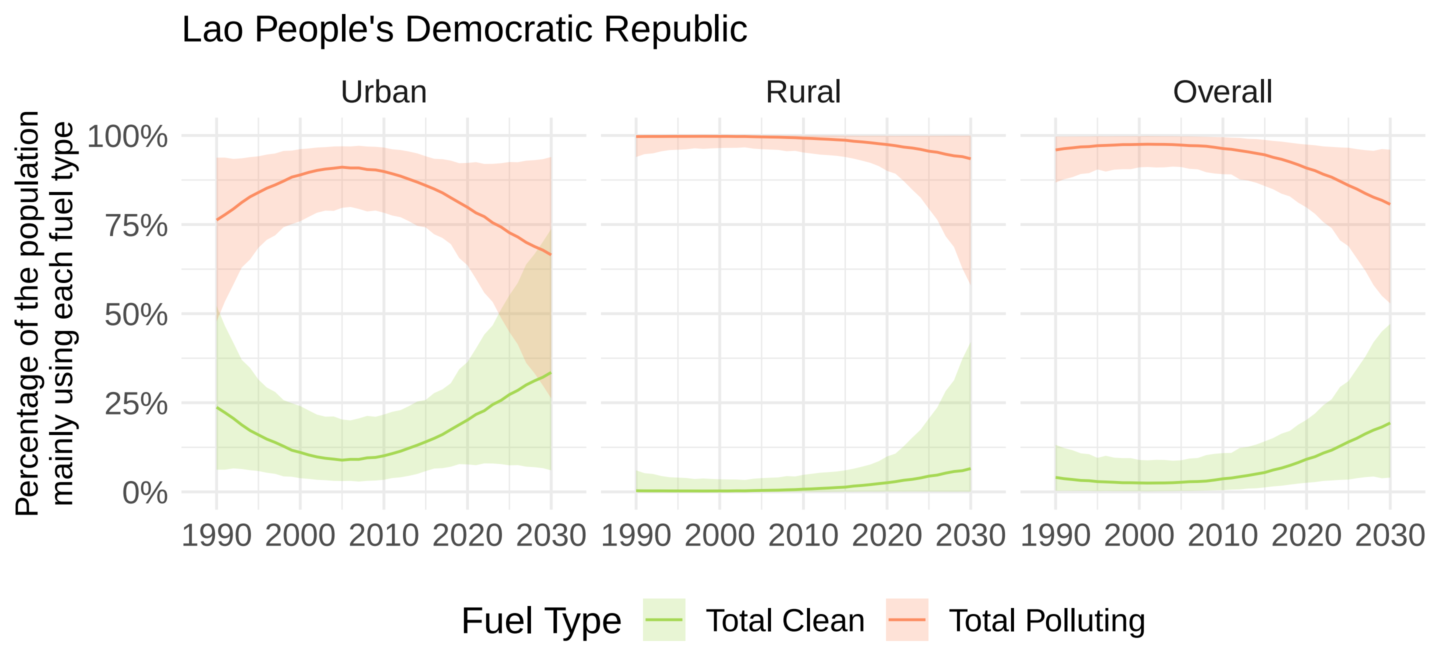
Lao People's Democratic Republic
Urban
Rural
Overall
Percentage of the population mainly using each fuel type
100%
75%
50%
25%
0%
1990
2000
2010
2020
2030
Fuel Type
Total Clean
Total Polluting

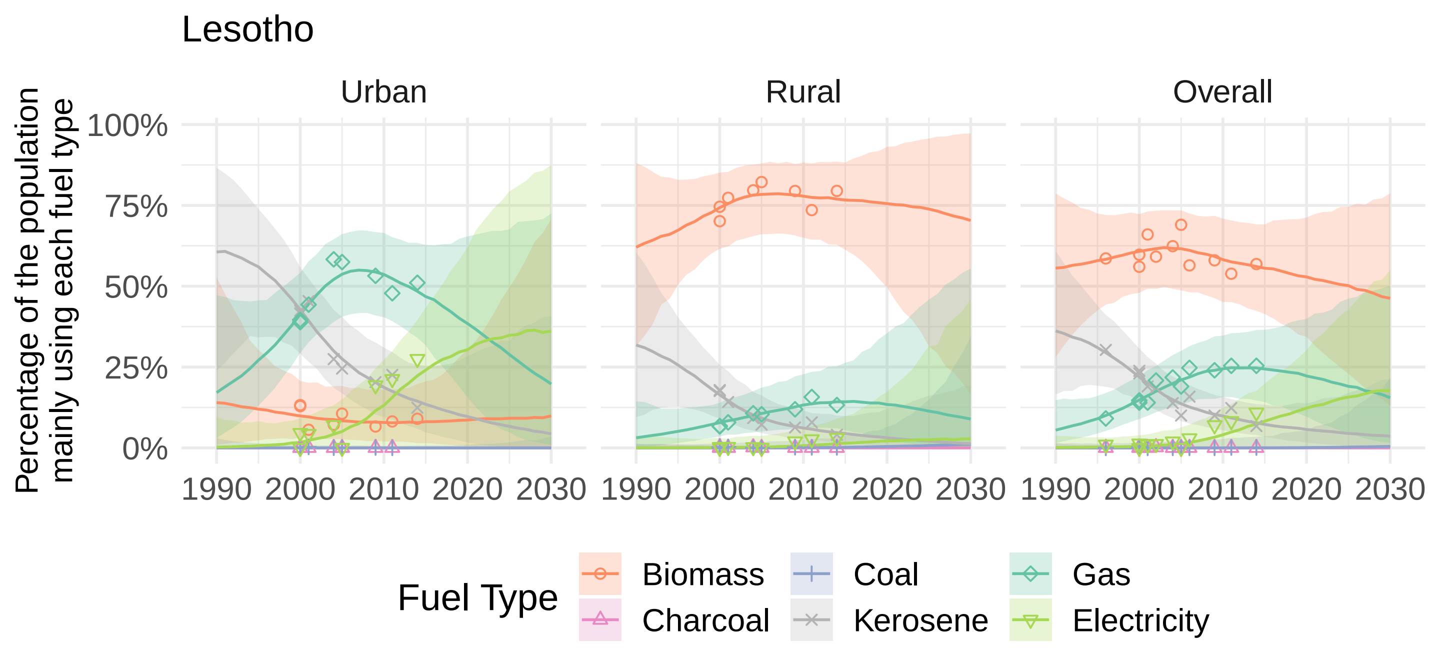

Lesotho
Urban
Rural
Overall
Percentage of the population mainly using each fuel type
100%
75%
50%
25%
0%
1990
2000
2010
2020
2030
Fuel Type
Biomass
Charcoal
Coal
Kerosene
Gas
Electricity

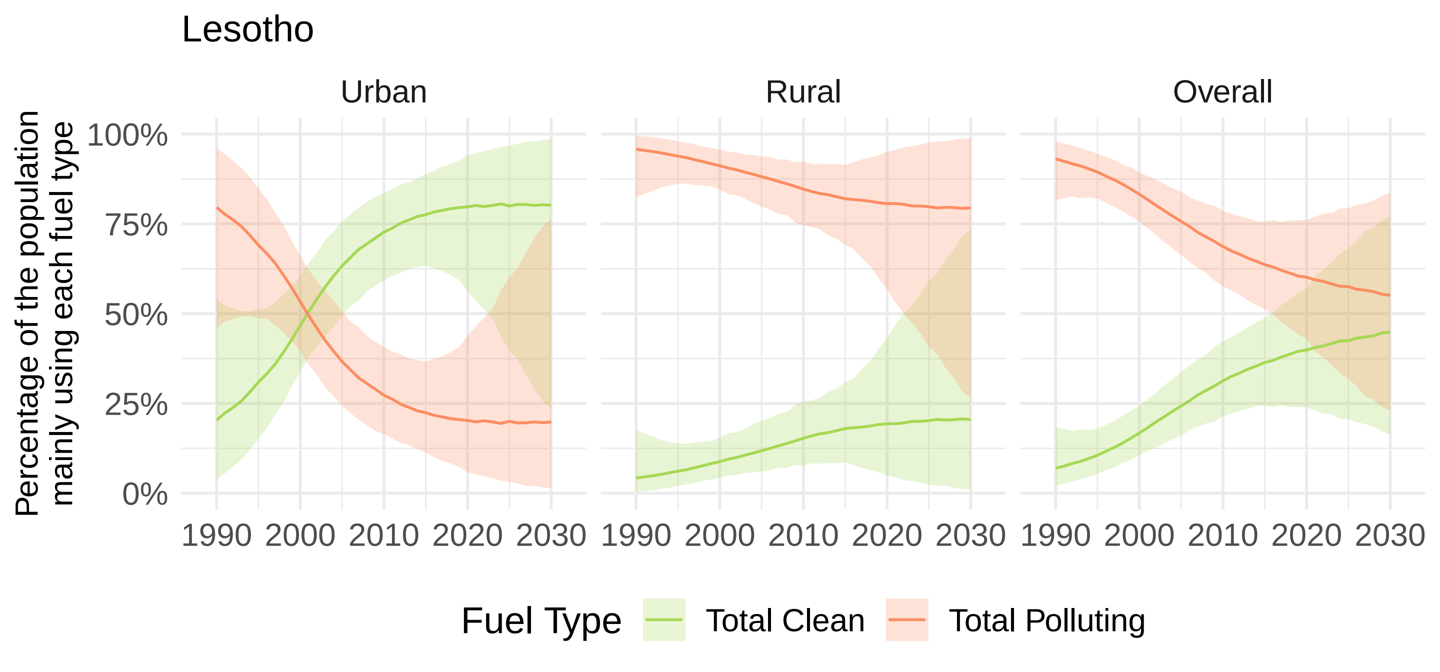

Lesotho
Urban
Rural
Overall
Percentage of the population mainly using each fuel type
100%
75%
50%
25%
0%
1990
2000
2010
2020
2030
Fuel Type
Total Clean
Total Polluting

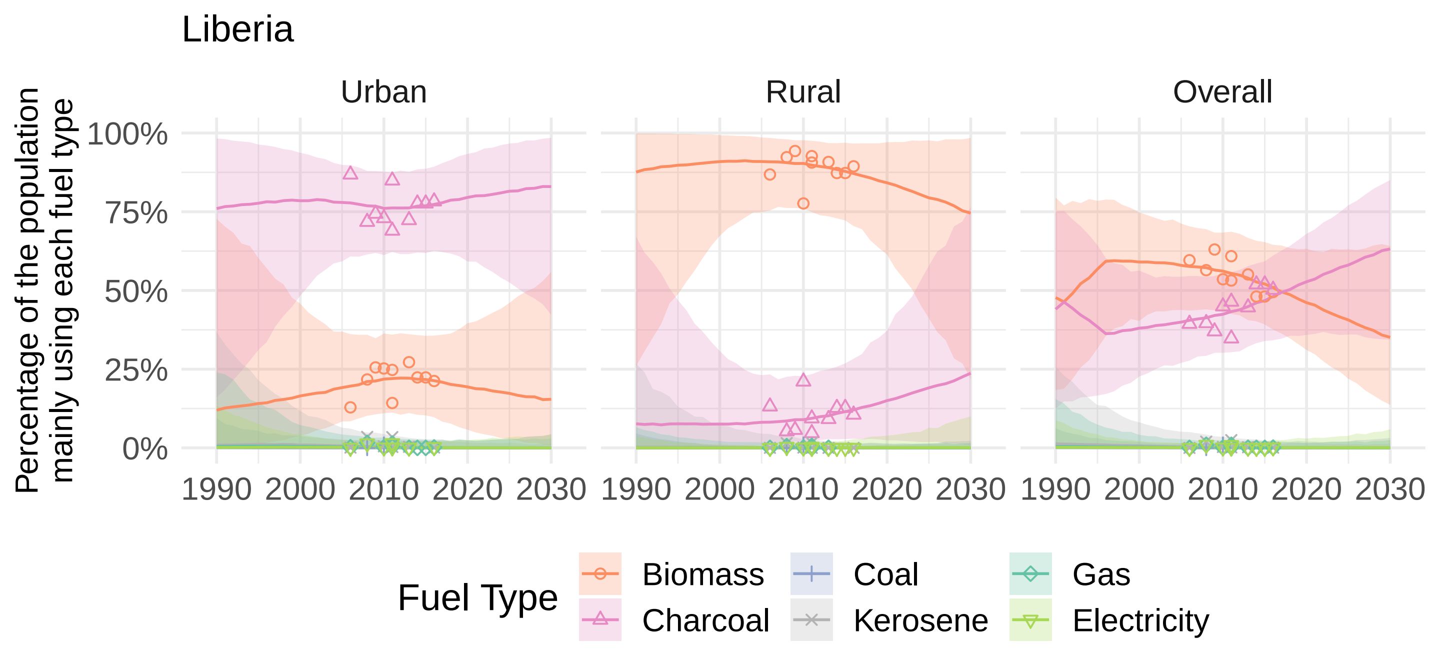

Liberia
Urban
Rural
Overall
Percentage of the population mainly using each fuel type
100%
75%
50%
25%
0%
1990
2000
2010
2020
2030
Fuel Type
Biomass
Charcoal
Coal
Kerosene
Gas
Electricity

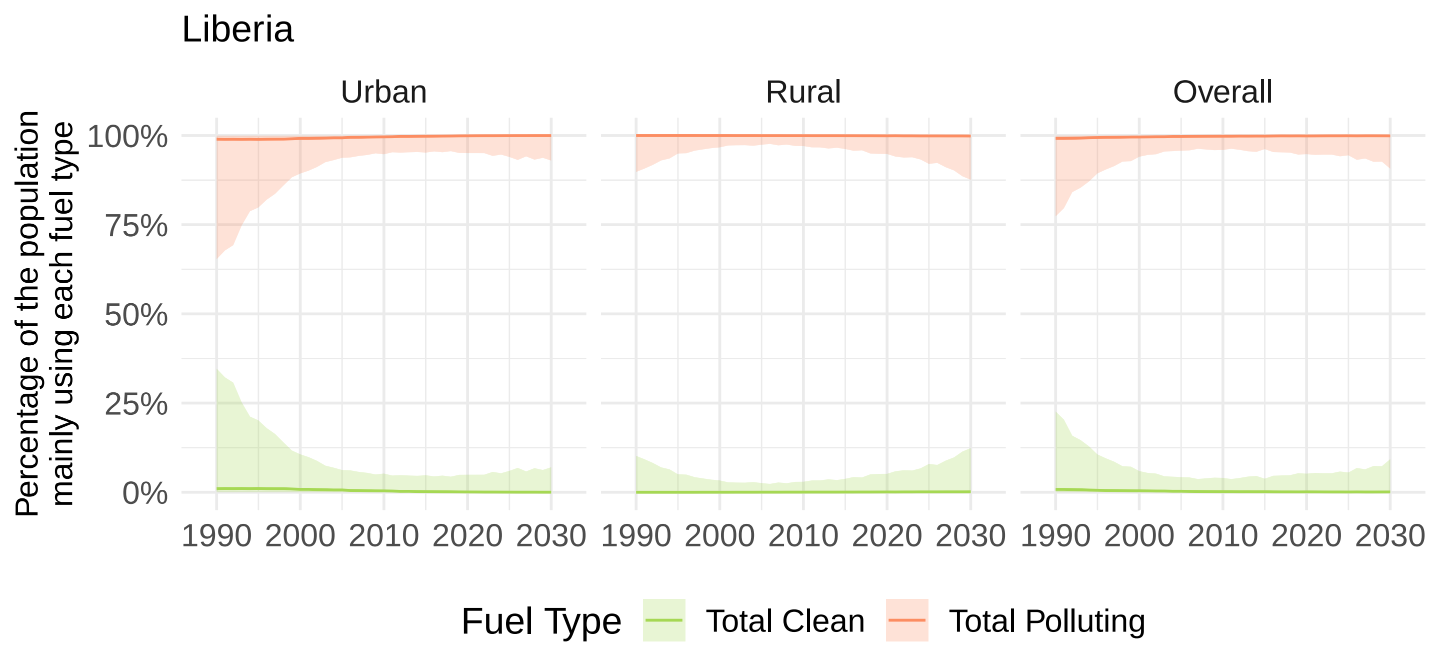

Liberia
Urban
Rural
Overall
Percentage of the population mainly using each fuel type
100%
75%
50%
25%
0%
1990
2000
2010
2020
2030
Fuel Type
Total Clean
Total Polluting

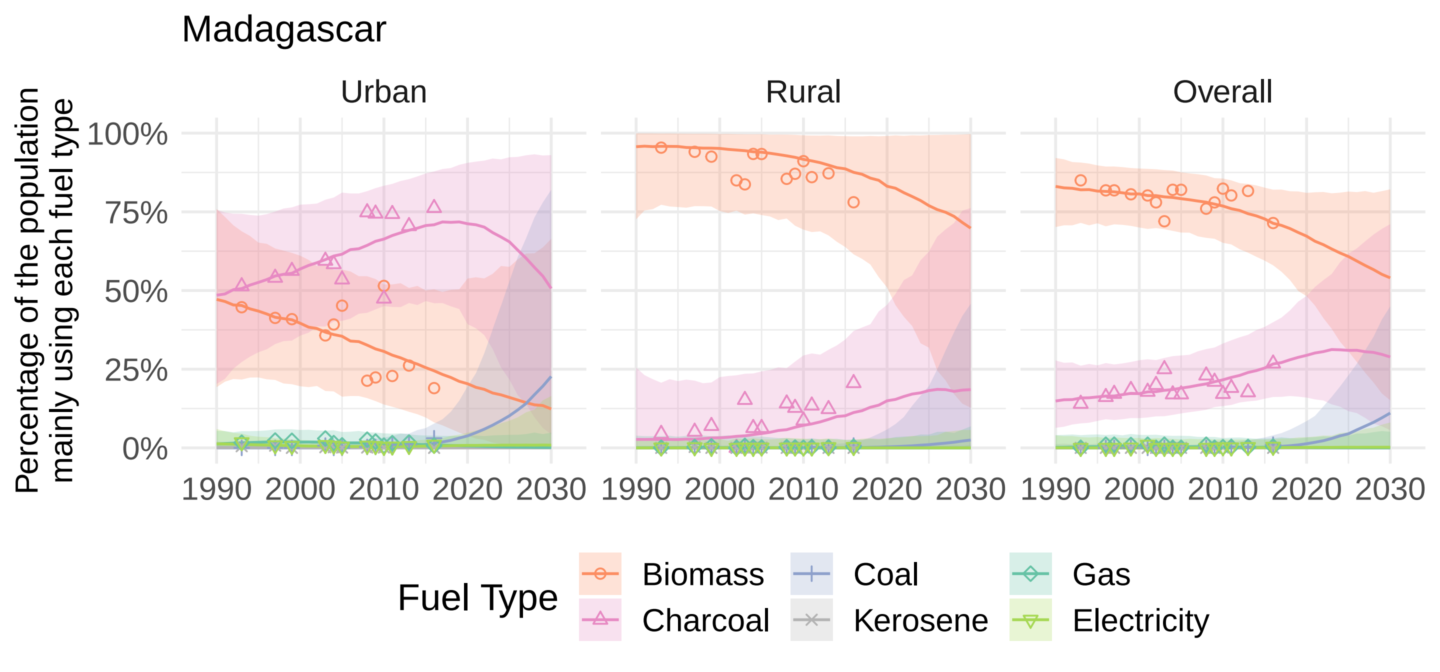
Madagascar
Urban
Rural
Overall
Percentage of the population mainly using each fuel type
100%
75%
50%
25%
0%
1990
2000
2010
2020
2030
Fuel Type
Biomass
Charcoal
Coal
Kerosene
Gas
Electricity

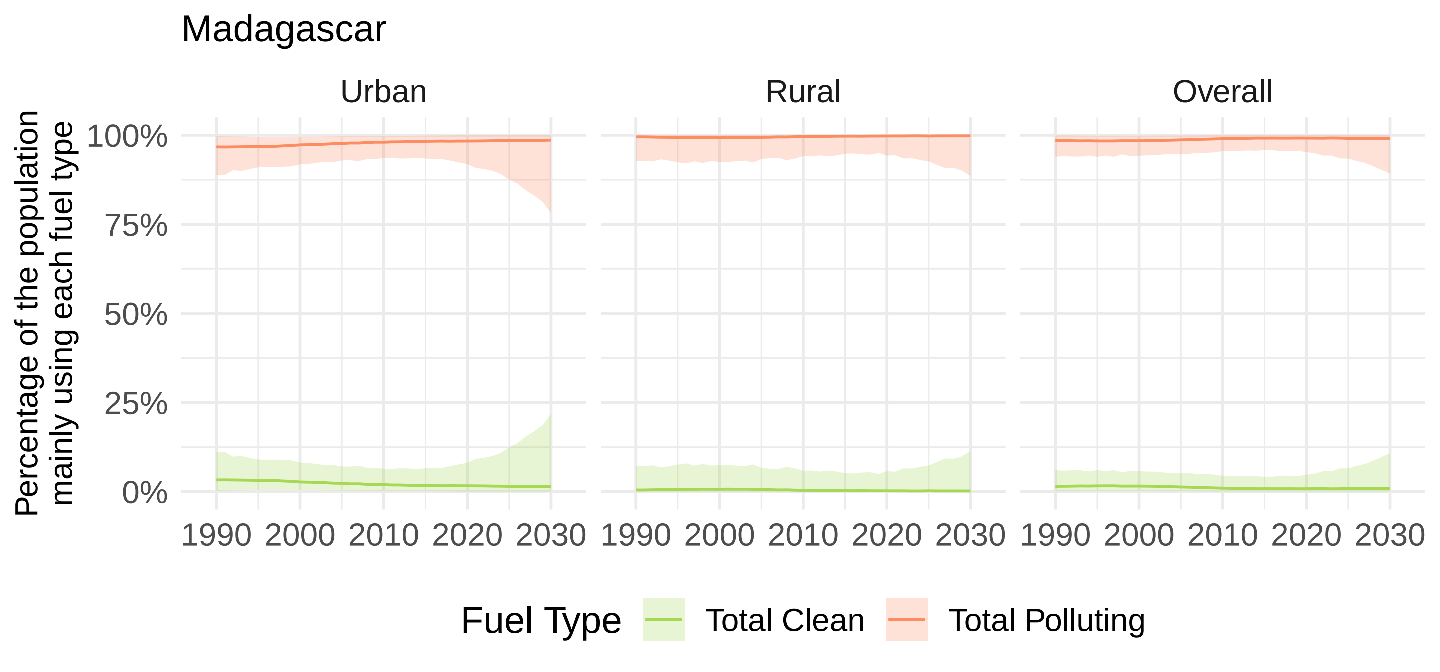

Madagascar
Urban
Rural
Overall
Percentage of the population mainly using each fuel type
100%
75%
50%
25%
0%
1990
2000
2010
2020
2030
Fuel Type
Total Clean
Total Polluting

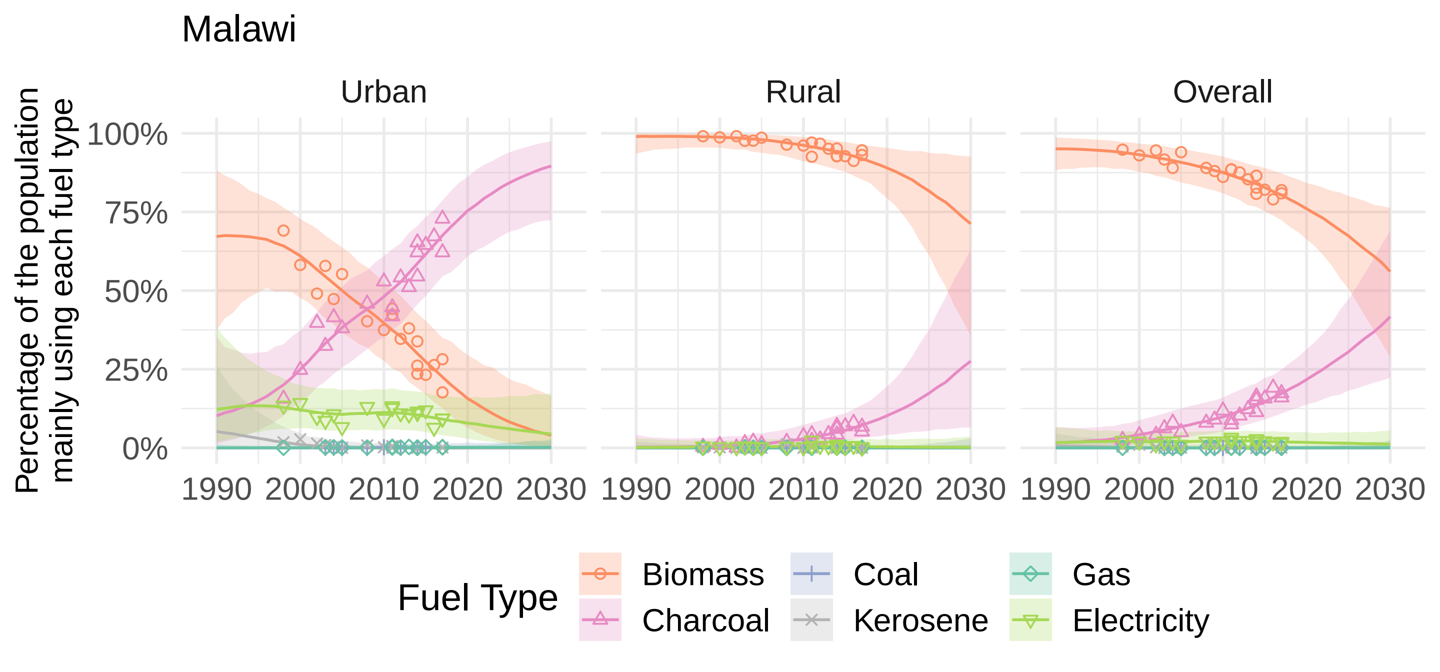
Malawi
Urban
Rural
Overall
Percentage of the population mainly using each fuel type
100%
75%
50%
25%
0%
1990
2000
2010
2020
2030
Fuel Type
Biomass
Charcoal
Coal
Kerosene
Gas
Electricity

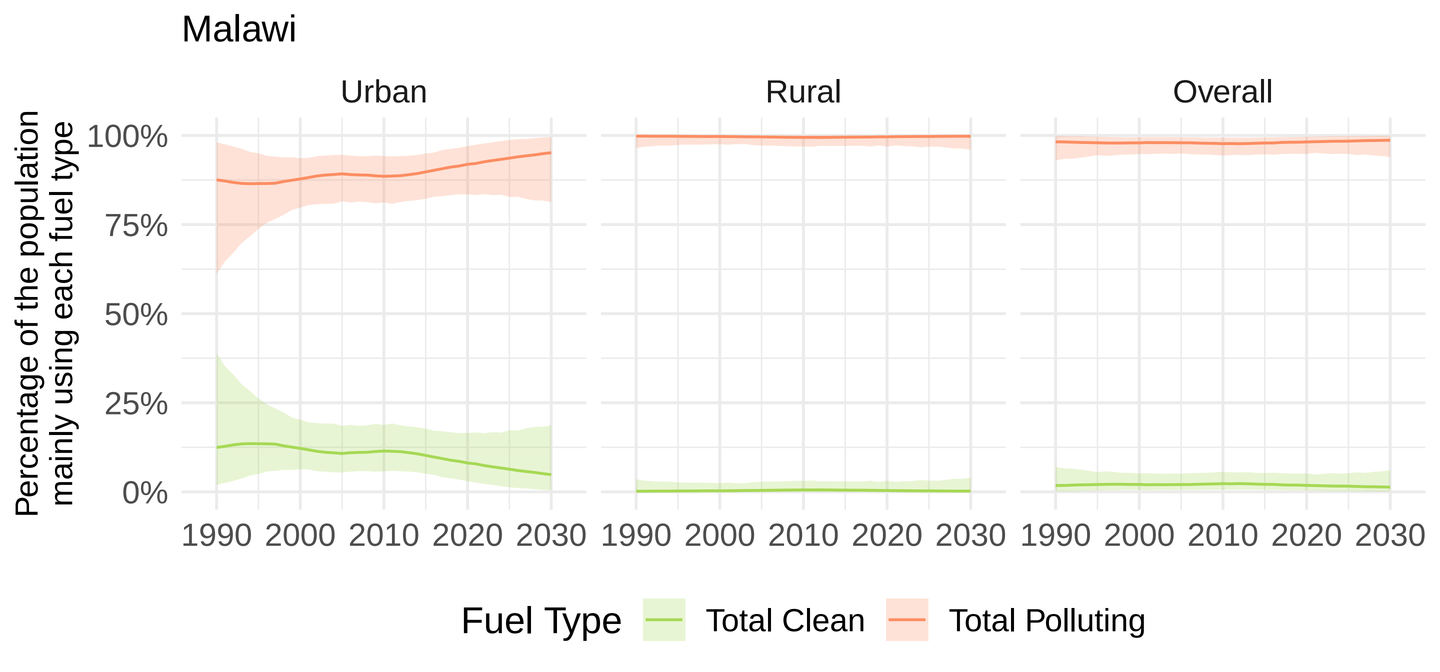
Malawi
Urban
Rural
Overall
Percentage of the population mainly using each fuel type
100%
75%
50%
25%
0%
1990
2000
2010
2020
2030
Fuel Type
Total Clean
Total Polluting

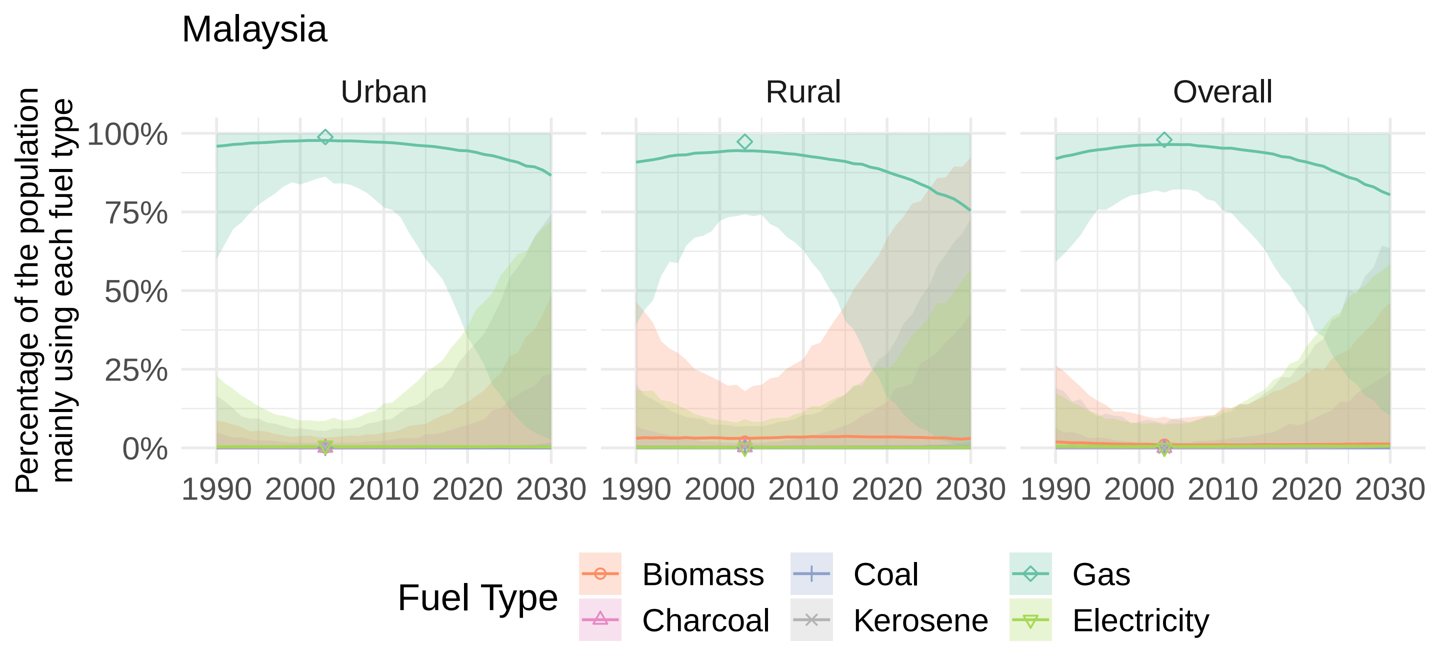

Malaysia
Urban
Rural
Overall
Percentage of the population mainly using each fuel type
100%
75%
50%
25%
0%
1990
2000
2010
2020
2030
Fuel Type
Biomass
Charcoal
Coal
Kerosene
Gas
Electricity

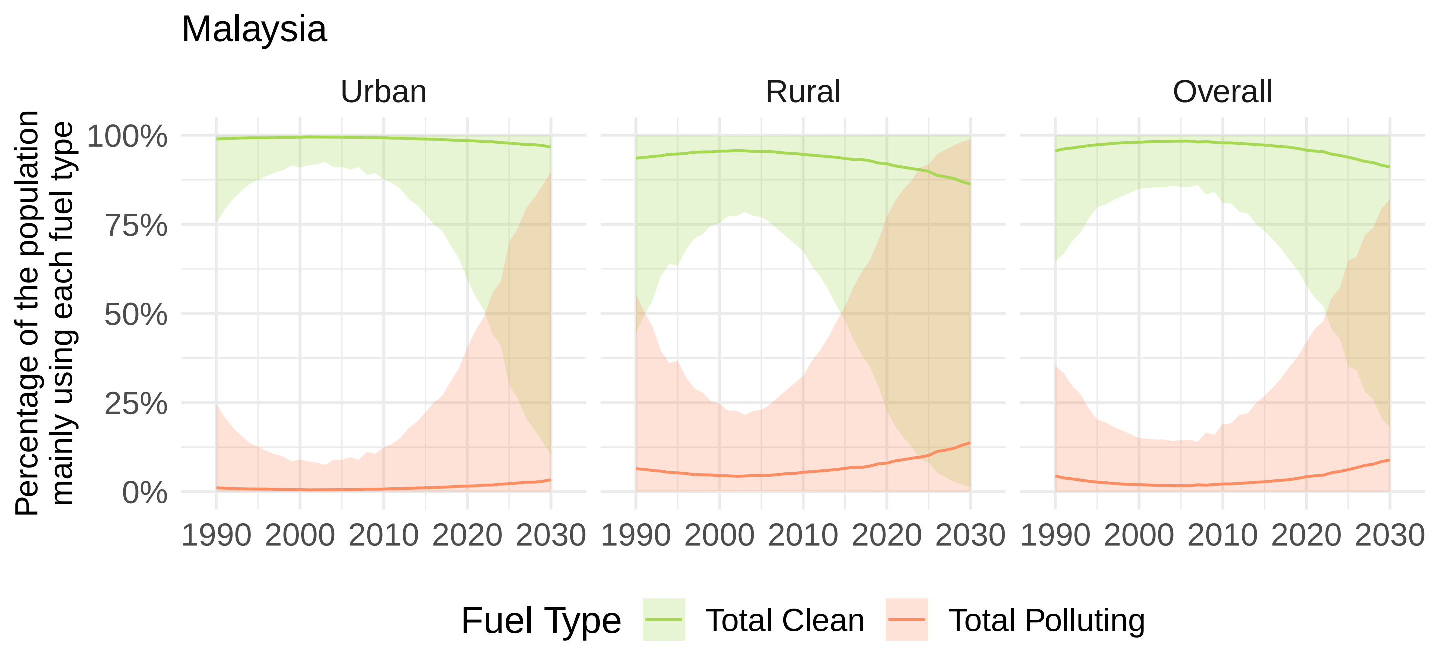
Malaysia
Urban
Rural
Overall
Percentage of the population mainly using each fuel type
100%
75%
50%
25%
0%
1990
2000
2010
2020
2030
Fuel Type
Total Clean
Total Polluting

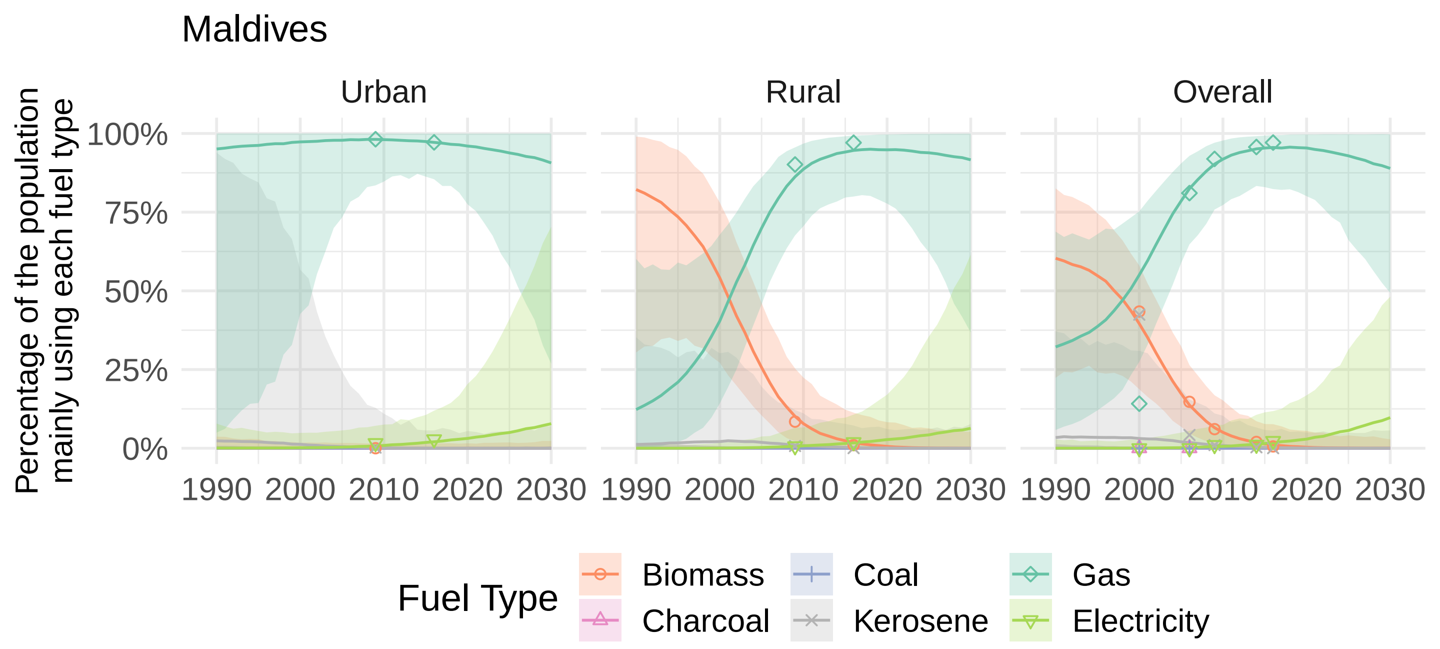
Maldives
Urban
Rural
Overall
Percentage of the population mainly using each fuel type
100%
75%
50%
25%
0%
1990
2000
2010
2020
2030
Fuel Type
Biomass
Charcoal
Coal
Kerosene
Gas
Electricity

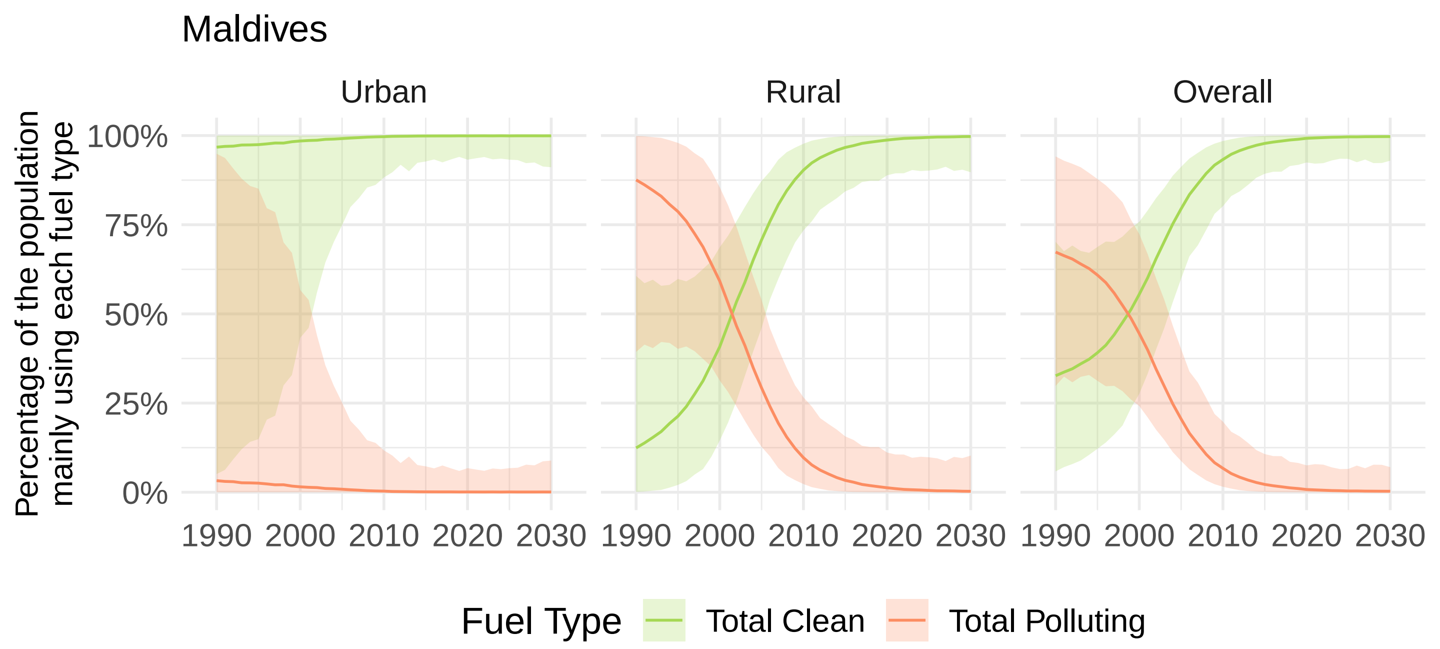

Maldives
Urban
Rural
Overall
Percentage of the population mainly using each fuel type
100%
75%
50%
25%
0%
1990
2000
2010
2020
2030
Fuel Type
Total Clean
Total Polluting

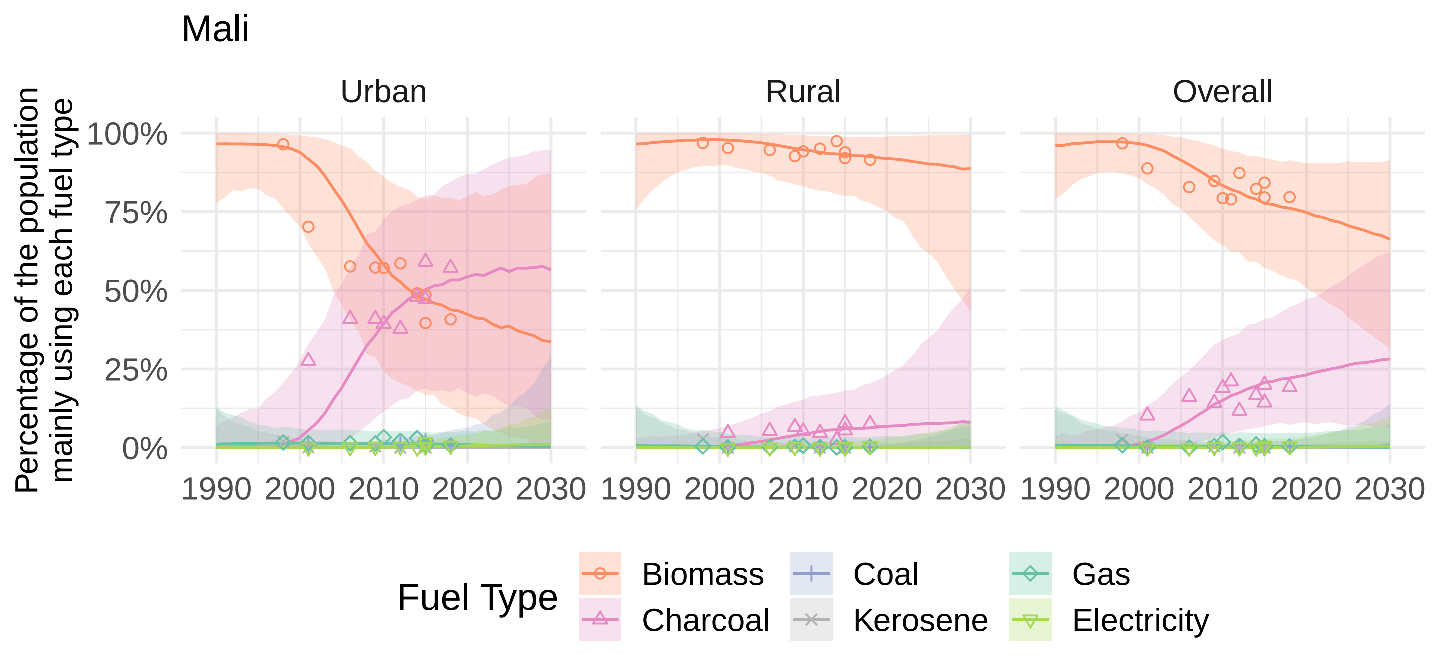
Mali
Urban
Rural
Overall
Percentage of the population mainly using each fuel type
100%
75%
50%
25%
0%
1990
2000
2010
2020
2030
Fuel Type
Biomass
Charcoal
Coal
Kerosene
Gas
Electricity

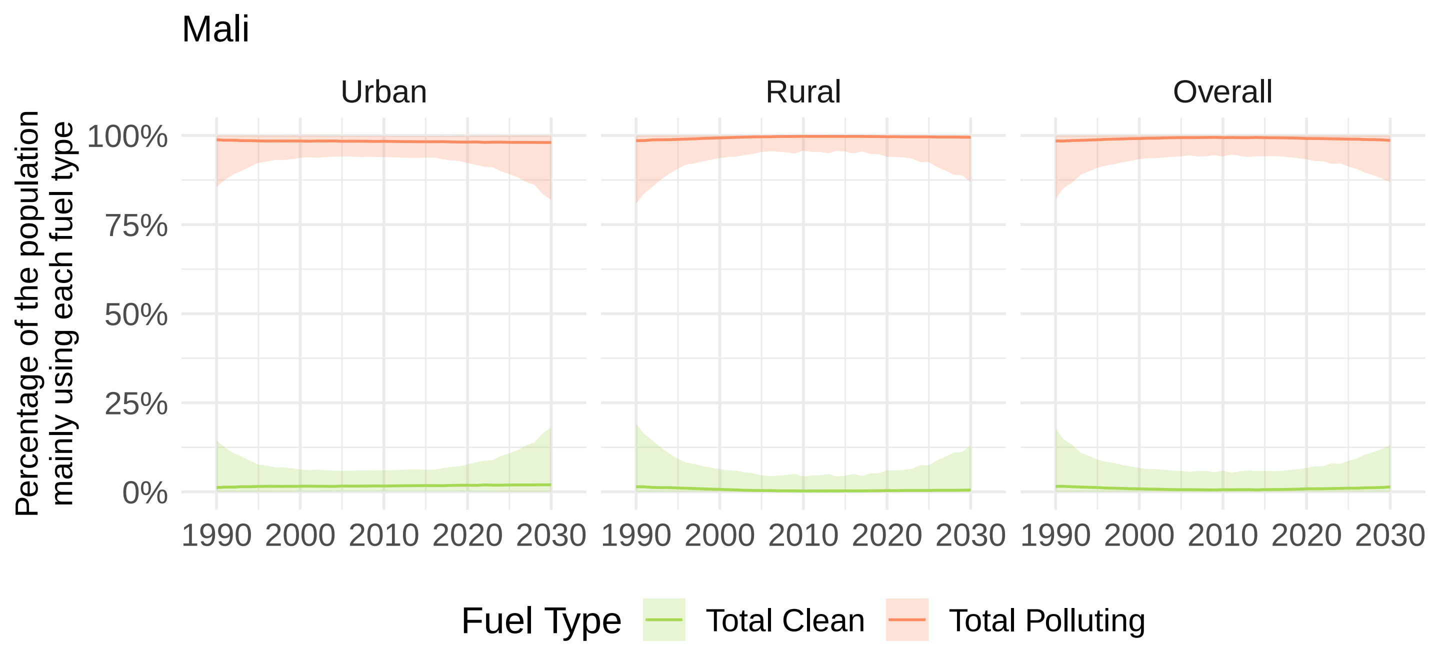
Mali
Urban
Rural
Overall
Percentage of the population mainly using each fuel type
100%
75%
50%
25%
0%
1990
2000
2010
2020
2030
Fuel Type
Total Clean
Total Polluting

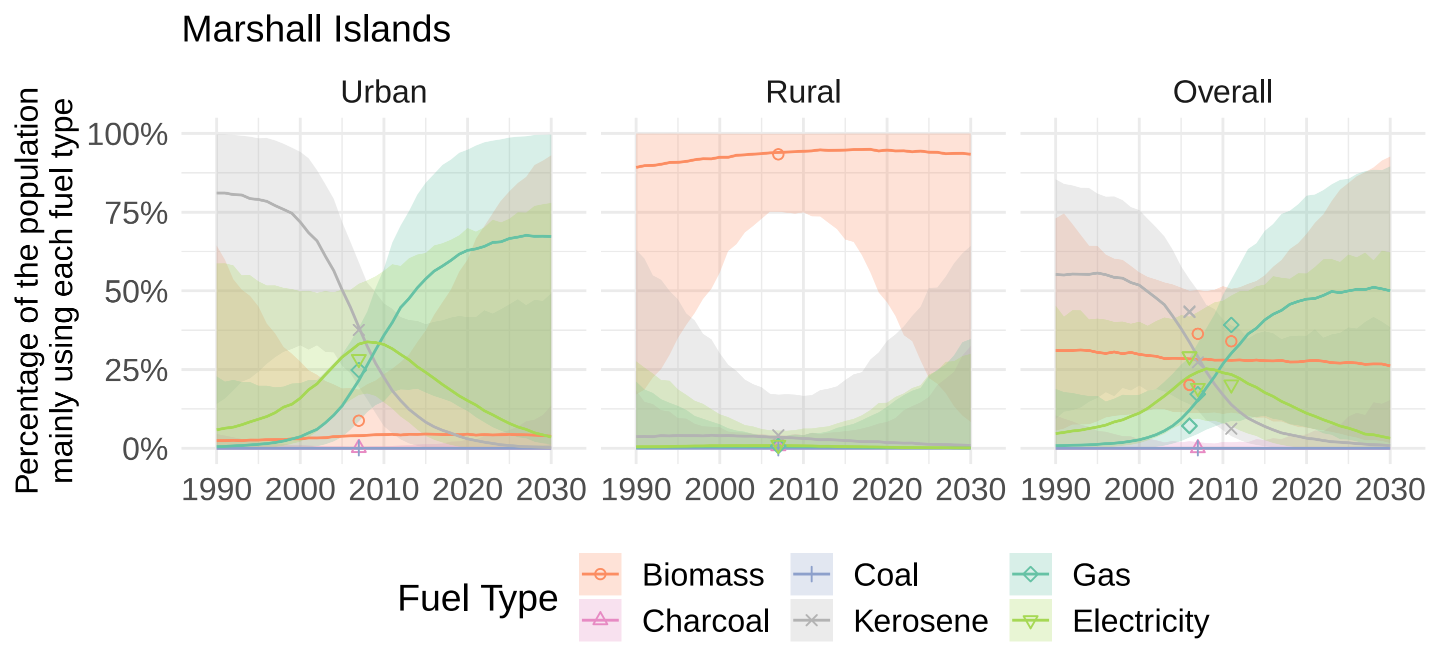
Marshall Islands
Urban
Rural
Overall
Percentage of the population mainly using each fuel type
100%
75%
50%
25%
0%
1990
2000
2010
2020
2030
Fuel Type
Biomass
Charcoal
Coal
Kerosene
Gas
Electricity

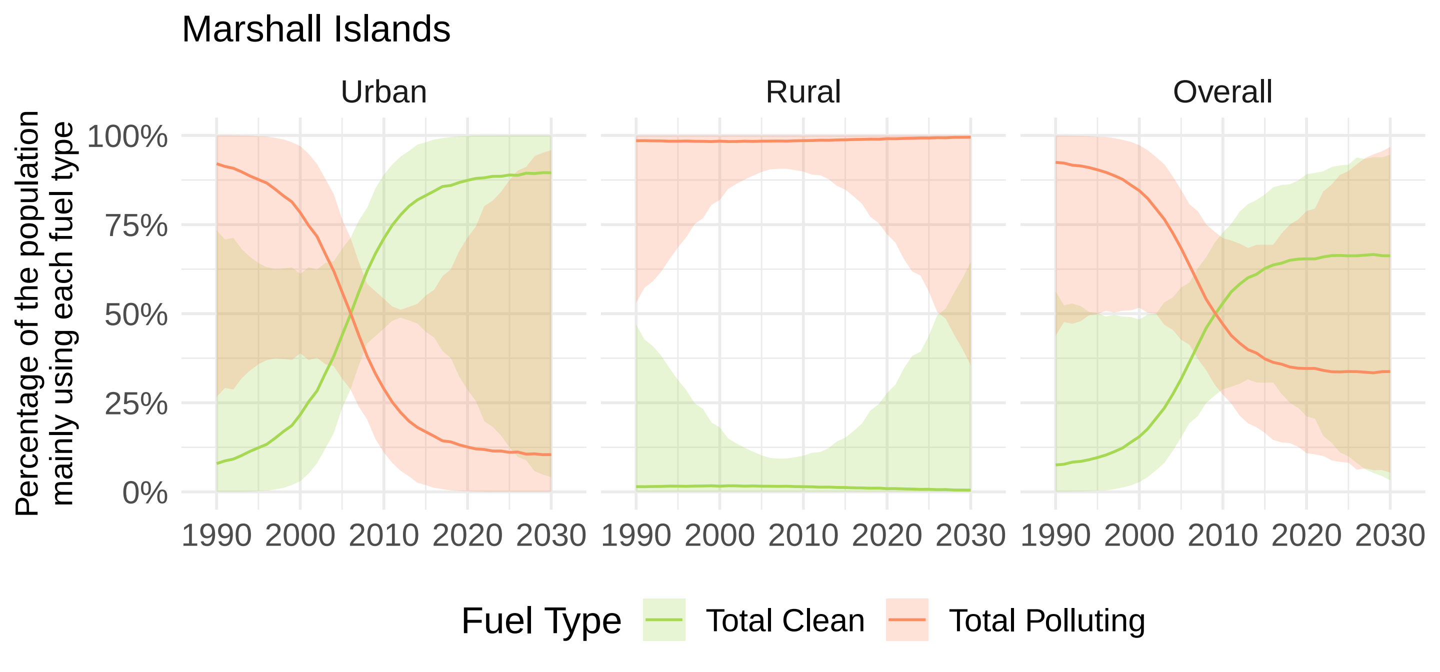
Marshall Islands
Urban
Rural
Overall
Percentage of the population mainly using each fuel type
100%
75%
50%
25%
0%
1990
2000
2010
2020
2030
Fuel Type
Total Clean
Total Polluting

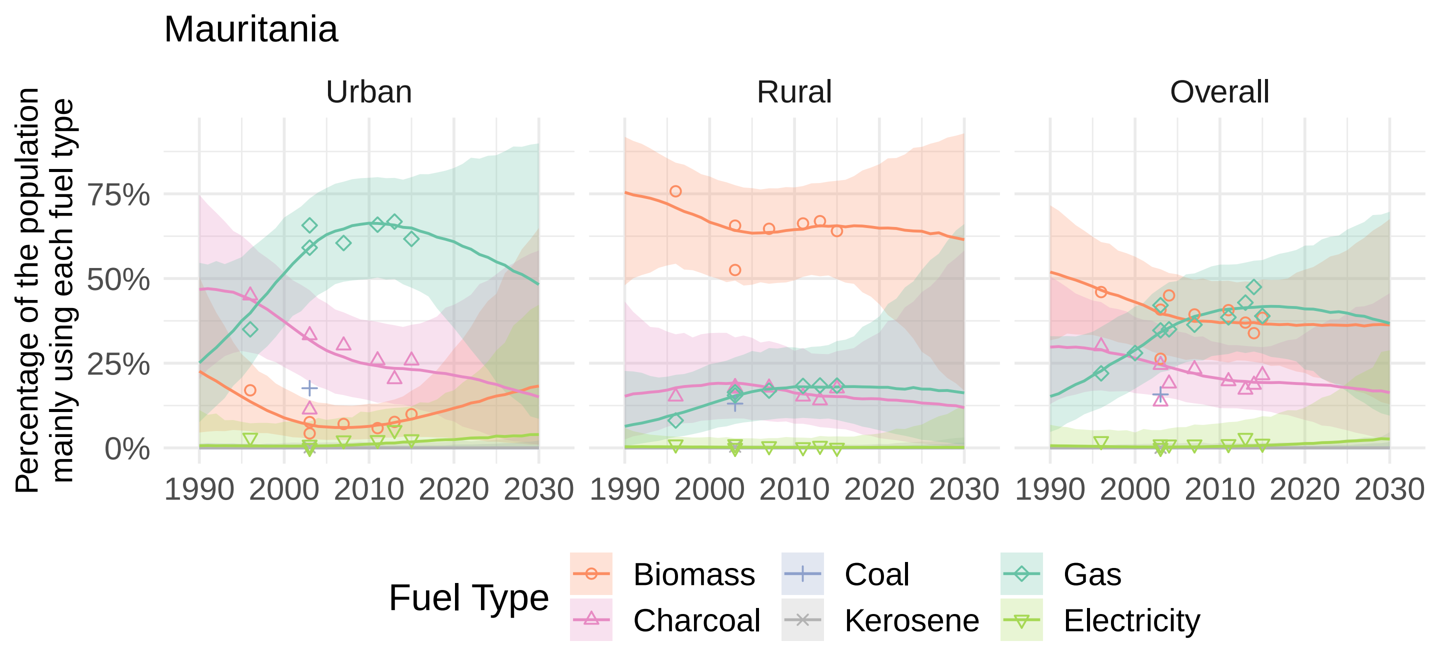

Mauritania
Urban
Rural
Overall
Percentage of the population mainly using each fuel type
75%
50%
25%
0%
1990
2000
2010
2020
2030
Fuel Type
Biomass
Charcoal
Coal
Kerosene
Gas
Electricity

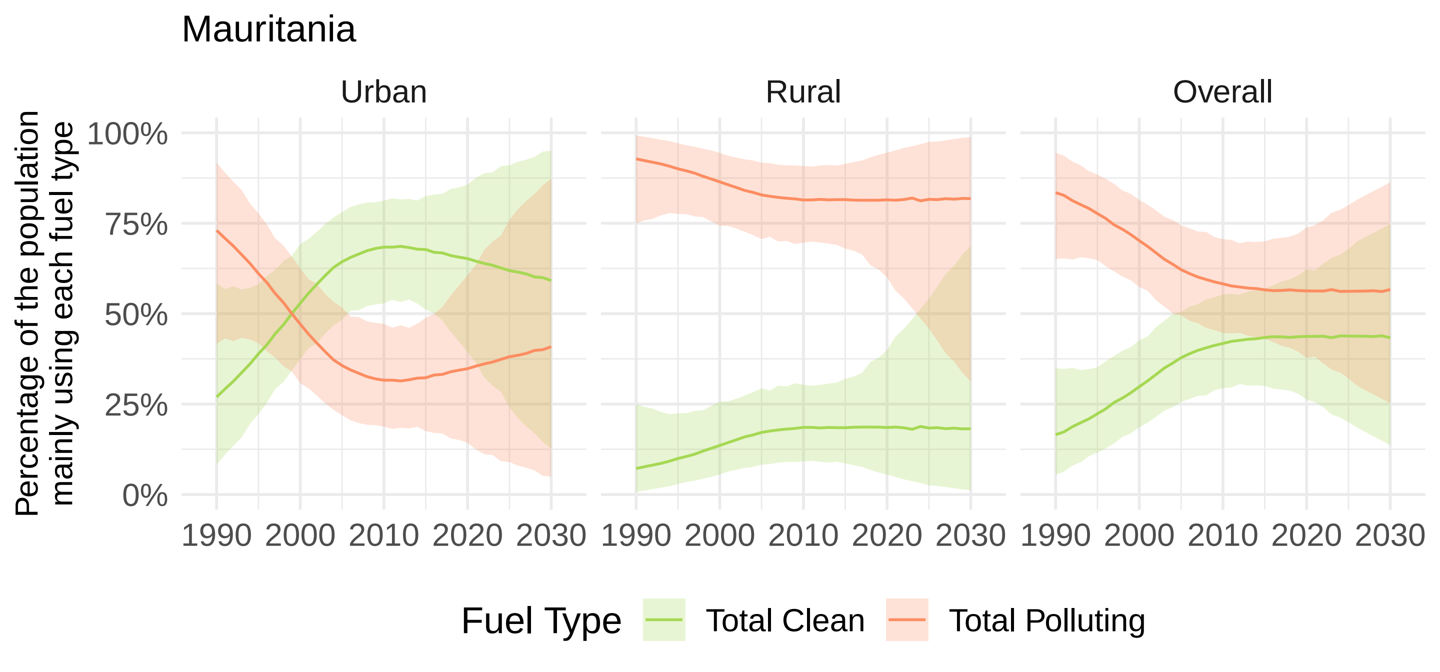

Mauritania
Urban
Rural
Overall
Percentage of the population mainly using each fuel type
100%
75%
50%
25%
0%
1990
2000
2010
2020
2030
Fuel Type
Total Clean
Total Polluting

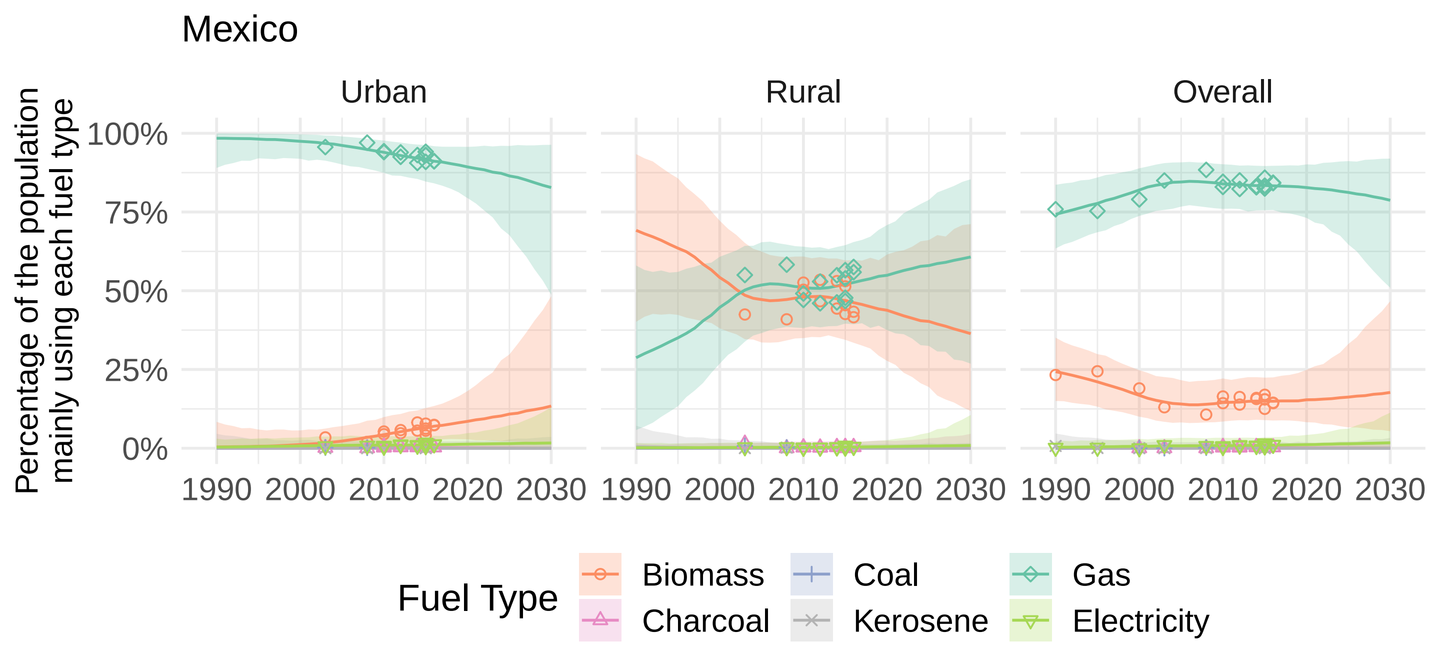
Mexico
Urban
Rural
Overall
Percentage of the population mainly using each fuel type
100%
75%
50%
25%
0%
1990
2000
2010
2020
2030
Fuel Type
Biomass
Charcoal
Coal
Kerosene
Gas
Electricity

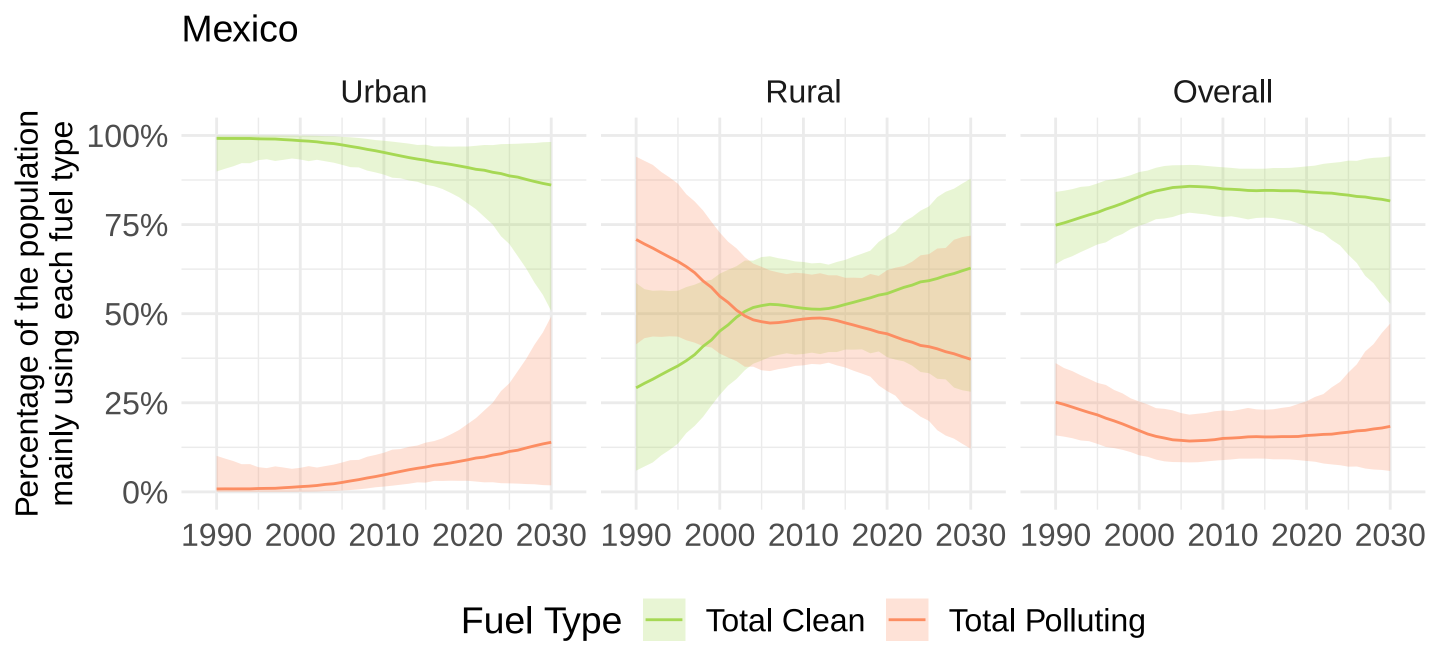

Mexico
Urban
Rural
Overall
Percentage of the population mainly using each fuel type
100%
75%
50%
25%
0%
1990
2000
2010
2020
2030
Fuel Type
Total Clean
Total Polluting

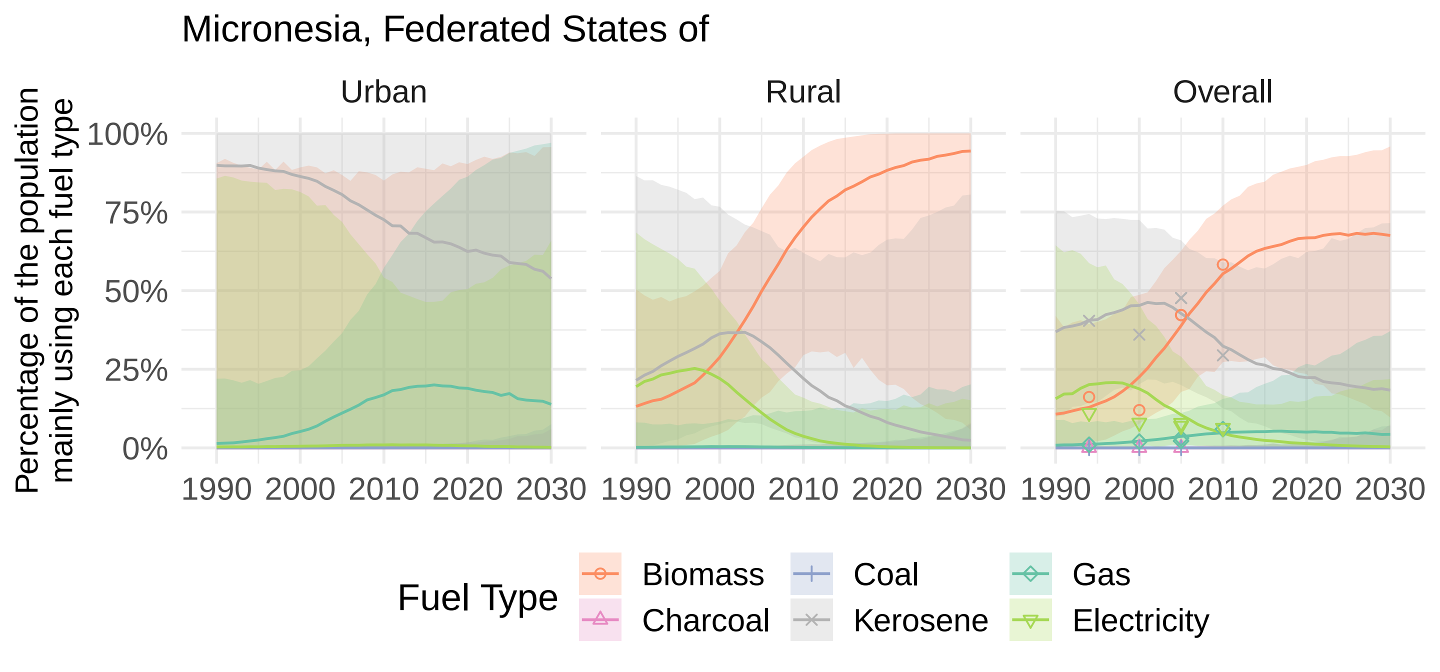

Micronesia, Federated States of
Urban
Rural
Overall
Percentage of the population mainly using each fuel type
100%
75%
50%
25%
0%
1990
2000
2010
2020
2030
Fuel Type
Biomass
Charcoal
Coal
Kerosene
Gas
Electricity

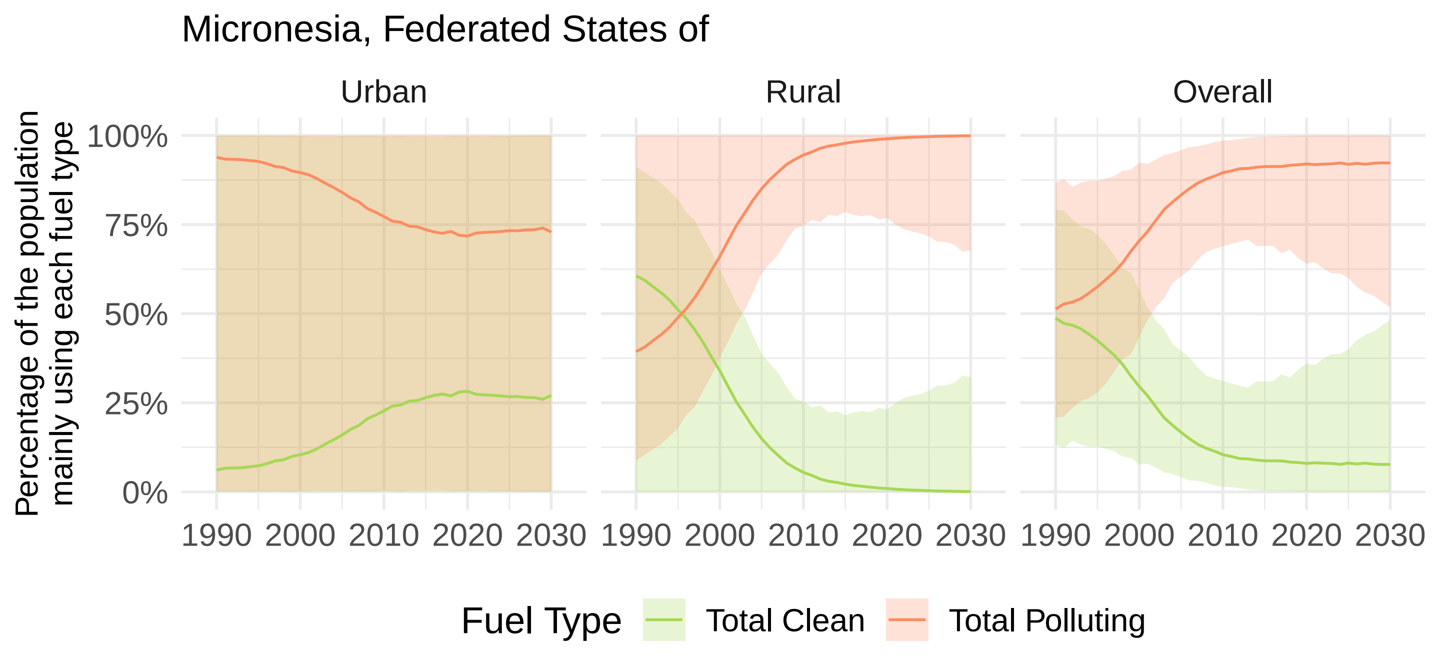
Micronesia, Federated States of
Urban
Rural
Overall
Percentage of the population mainly using each fuel type
100%
75%
50%
25%
0%
1990
2000
2010
2020
2030
Fuel Type
Total Clean
Total Polluting

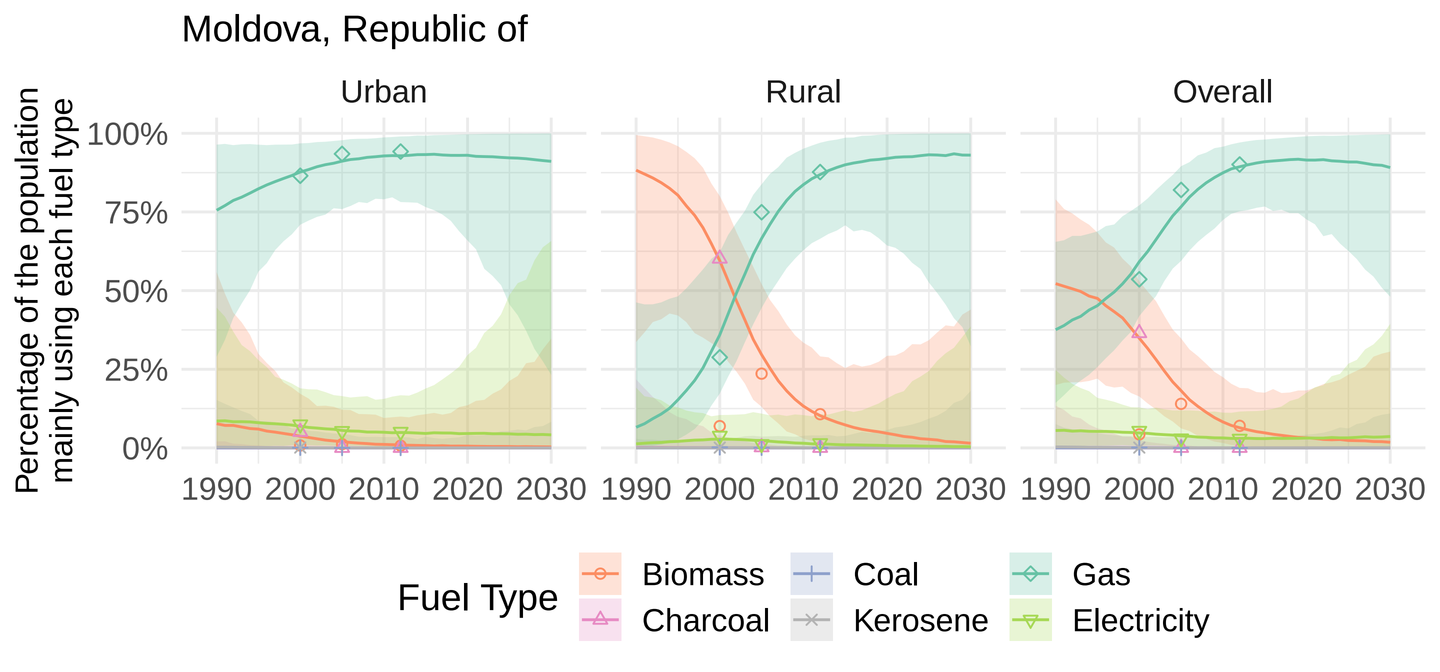

Moldova, Republic of
Urban
Rural
Overall
Percentage of the population mainly using each fuel type
100%
75%
50%
25%
0%
1990
2000
2010
2020
2030
Fuel Type
Biomass
Charcoal
Coal
Kerosene
Gas
Electricity

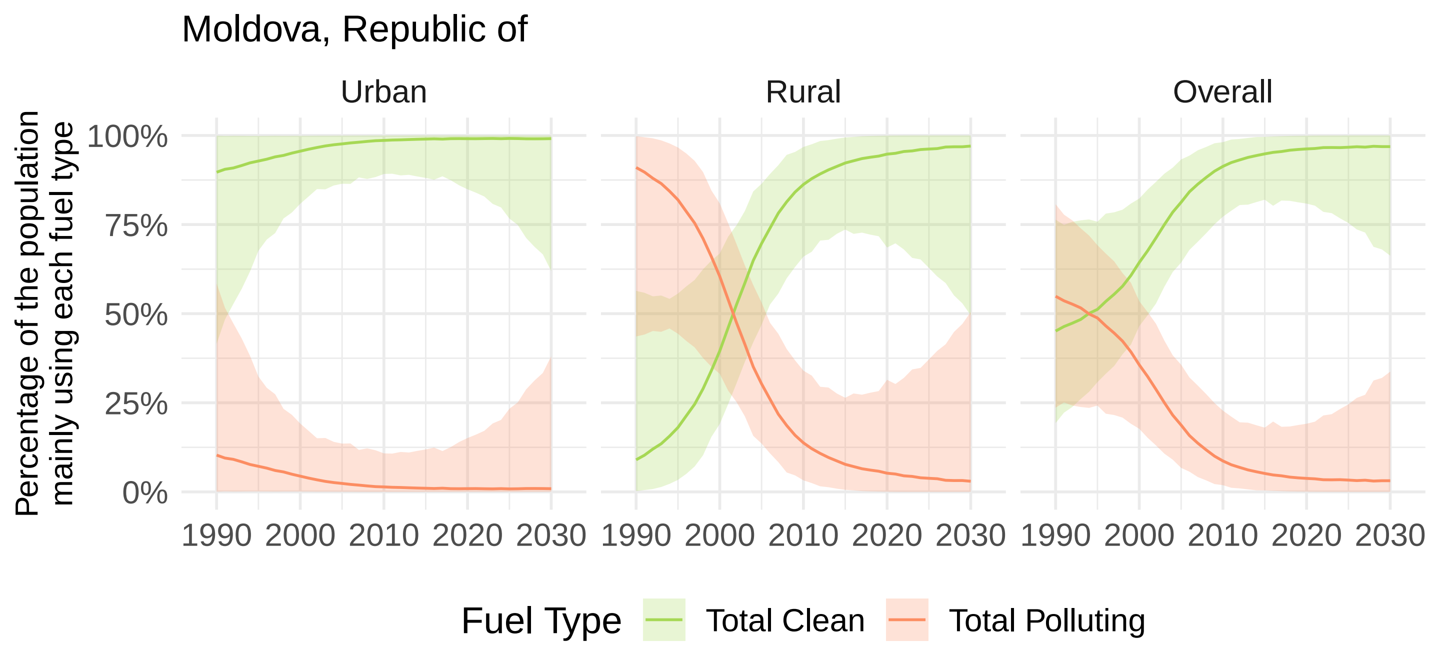
Moldova, Republic of
Urban
Rural
Overall
Percentage of the population mainly using each fuel type
100%
75%
50%
25%
0%
1990
2000
2010
2020
2030
Fuel Type
Total Clean
Total Polluting

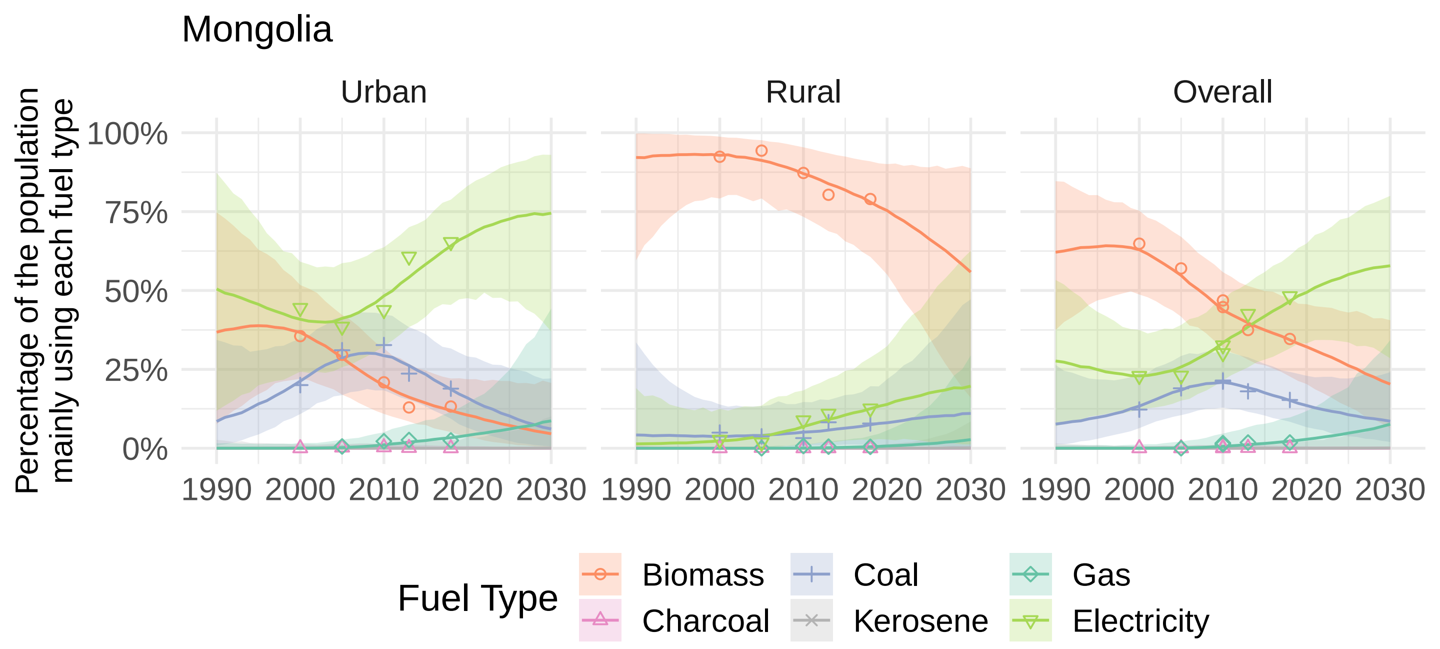
Mongolia
Urban
Rural
Overall
Percentage of the population mainly using each fuel type
100%
75%
50%
25%
0%
1990
2000
2010
2020
2030
Fuel Type
Biomass
Charcoal
Coal
Kerosene
Gas
Electricity

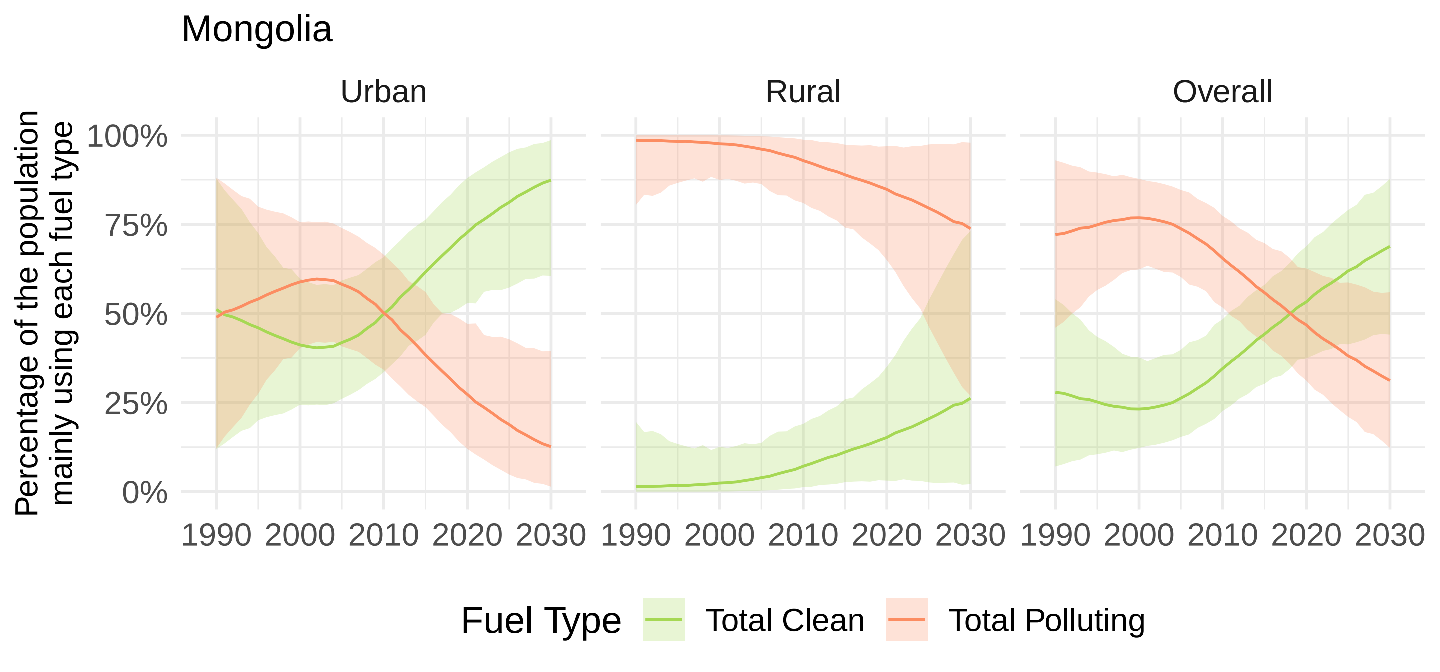
Mongolia
Urban
Rural
Overall
Percentage of the population mainly using each fuel type
100%
75%
50%
25%
0%
1990
2000
2010
2020
2030
Fuel Type
Total Clean
Total Polluting

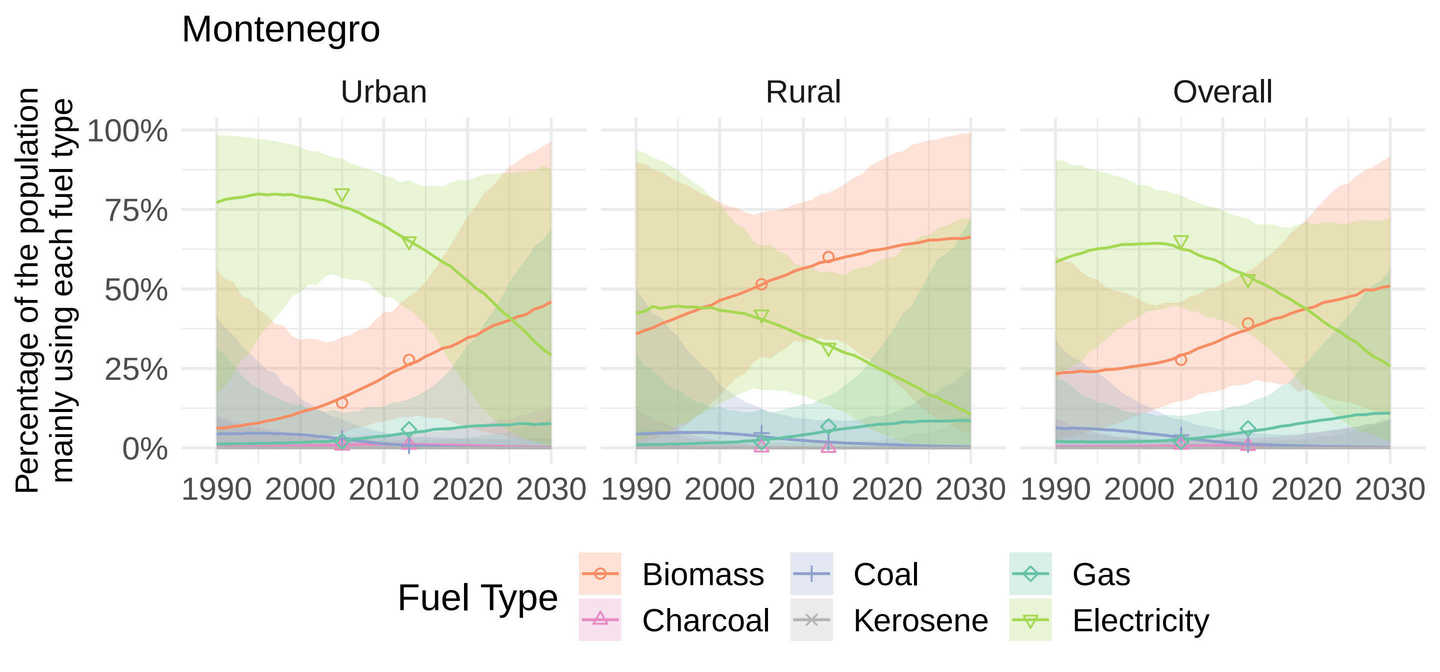
Montenegro
Urban
Rural
Overall
Percentage of the population mainly using each fuel type
100%
75%
50%
25%
0%
1990
2000
2010
2020
2030
Fuel Type
Biomass
Charcoal
Coal
Kerosene
Gas
Electricity

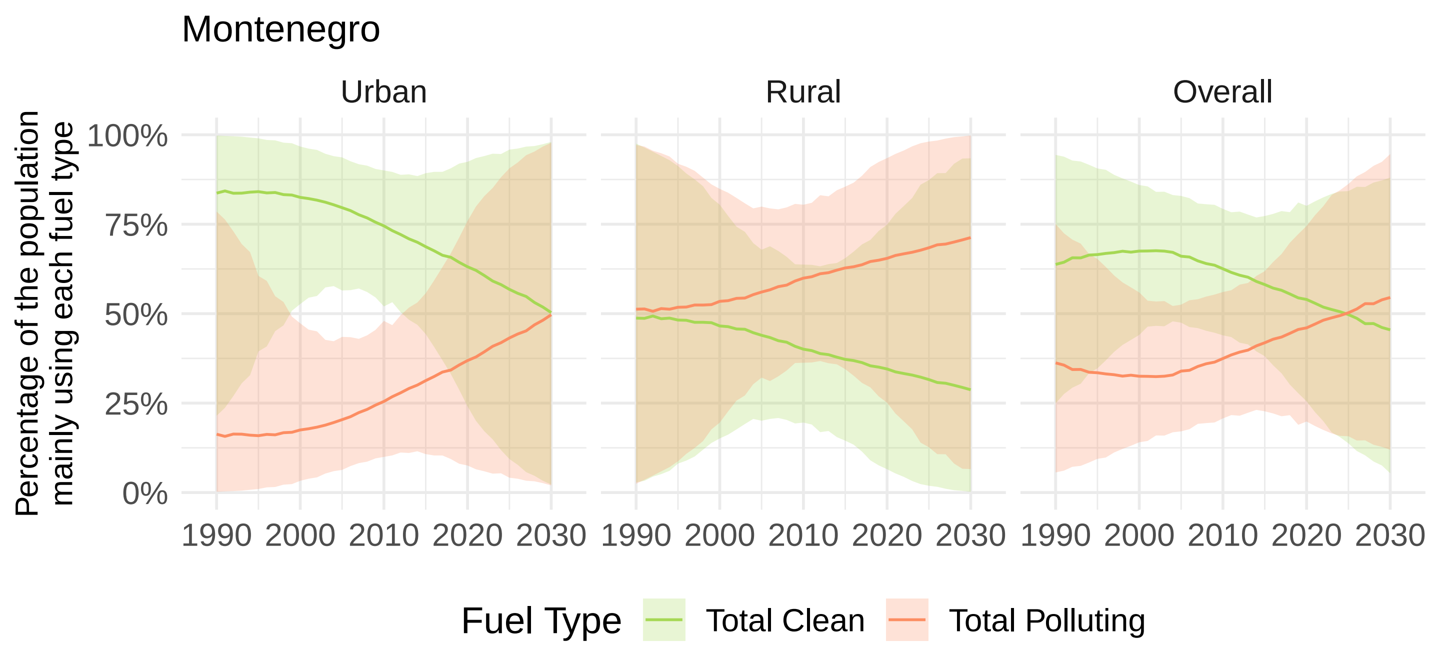

Montenegro
Urban
Rural
Overall
Percentage of the population mainly using each fuel type
100%
75%
50%
25%
0%
1990
2000
2010
2020
2030
Fuel Type
Total Clean
Total Polluting

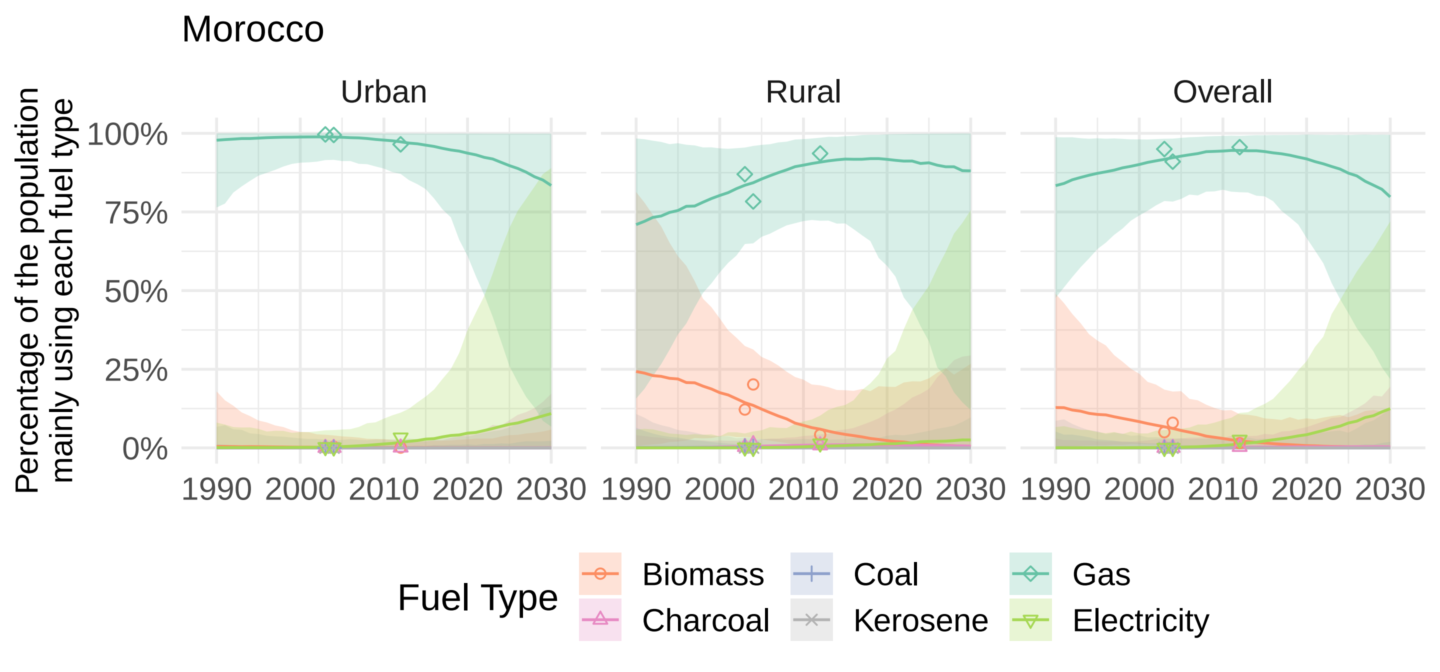
Morocco
Urban
Rural
Overall
Percentage of the population
mainly using each fuel type
100%
75%
50%
25%
0%
1990
2000
2010
2020
2030
Fuel Type
Biomass
Charcoal
Coal
Kerosene
Gas
Electricity

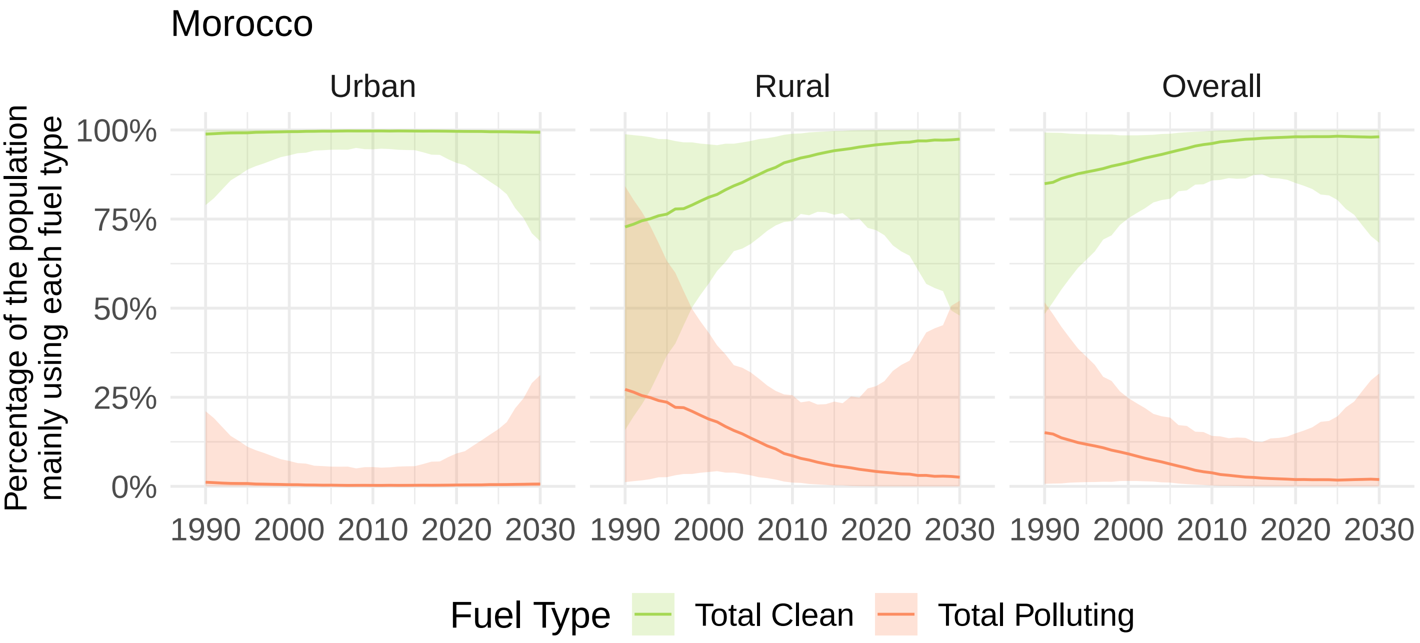

Morocco
Urban
Rural
Overall
Percentage of the population mainly using each fuel type
100%
75%
50%
25%
0%
1990
2000
2010
2020
2030
Fuel Type
Total Clean
Total Polluting

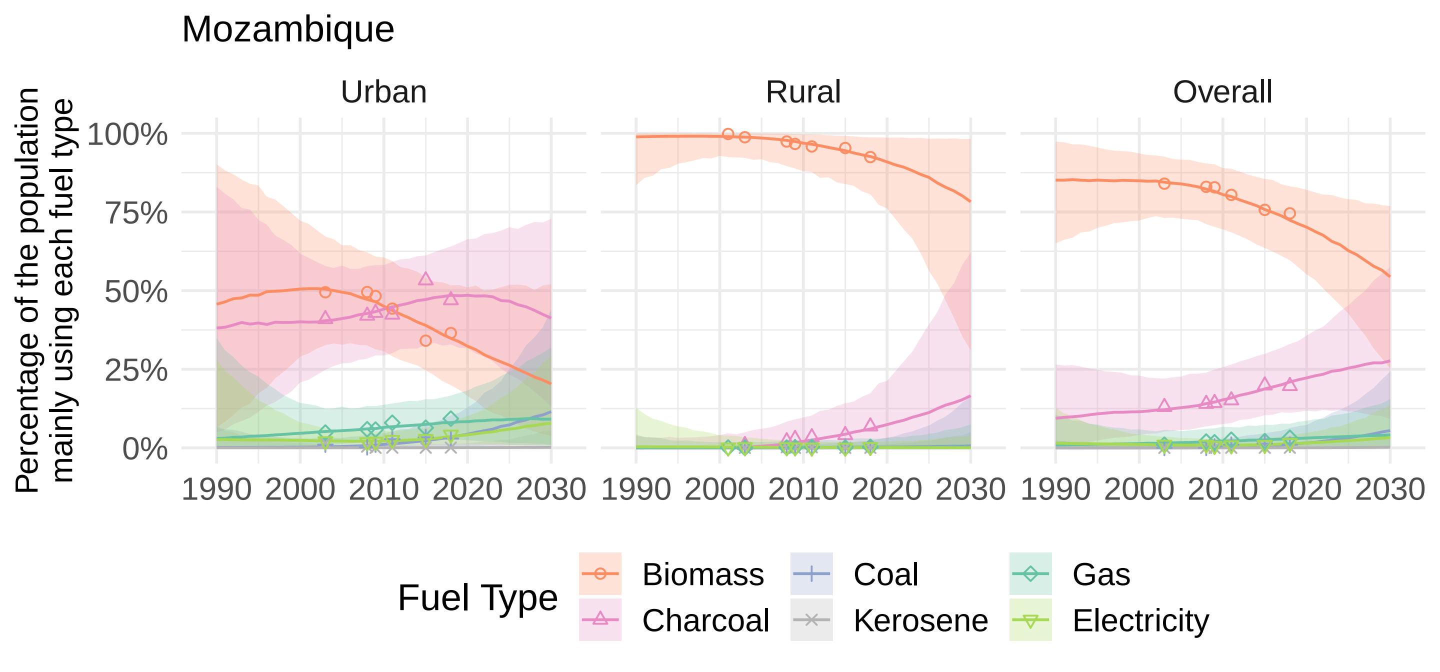
Mozambique
Urban
Rural
Overall
Percentage of the population mainly using each fuel type
100%
75%
50%
25%
0%
1990
2000
2010
2020
2030
Fuel Type
Biomass
Charcoal
Coal
Kerosene
Gas
Electricity

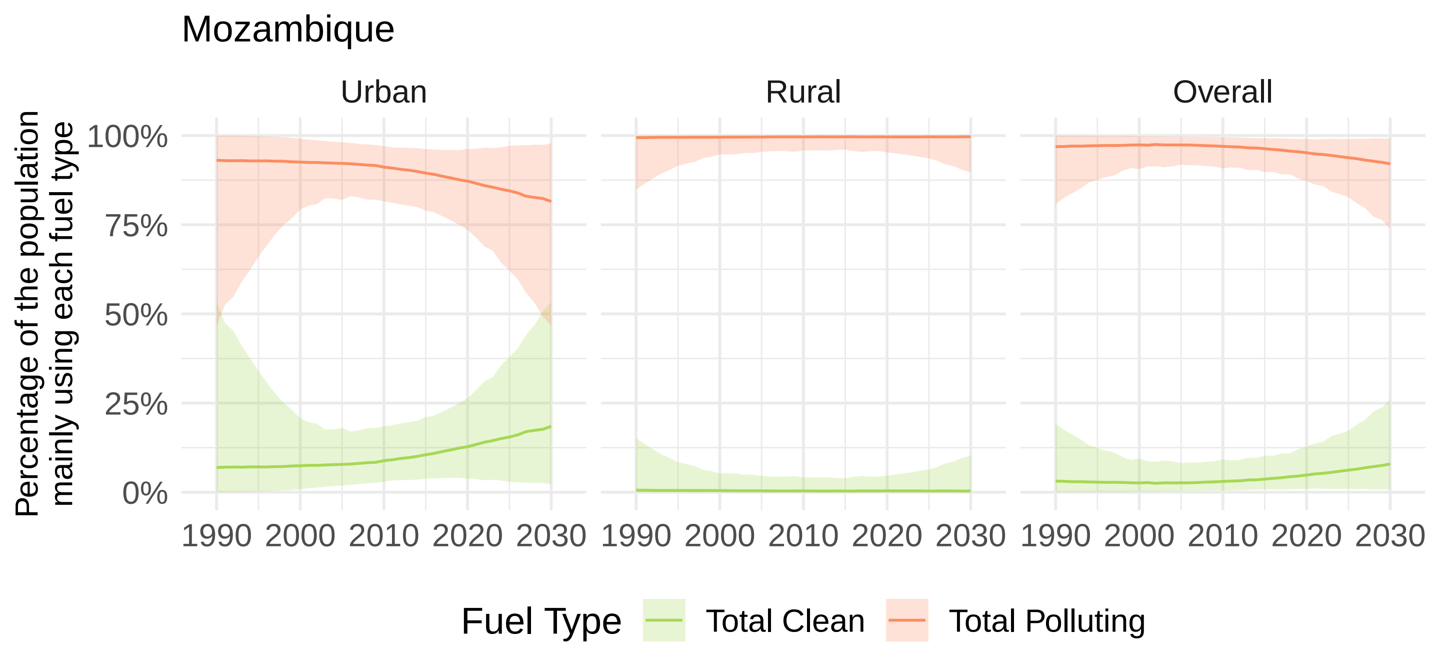

Mozambique
Urban
Rural
Overall
Percentage of the population mainly using each fuel type
100%
75%
50%
25%
0%
1990
2000
2010
2020
2030
Fuel Type
Total Clean
Total Polluting

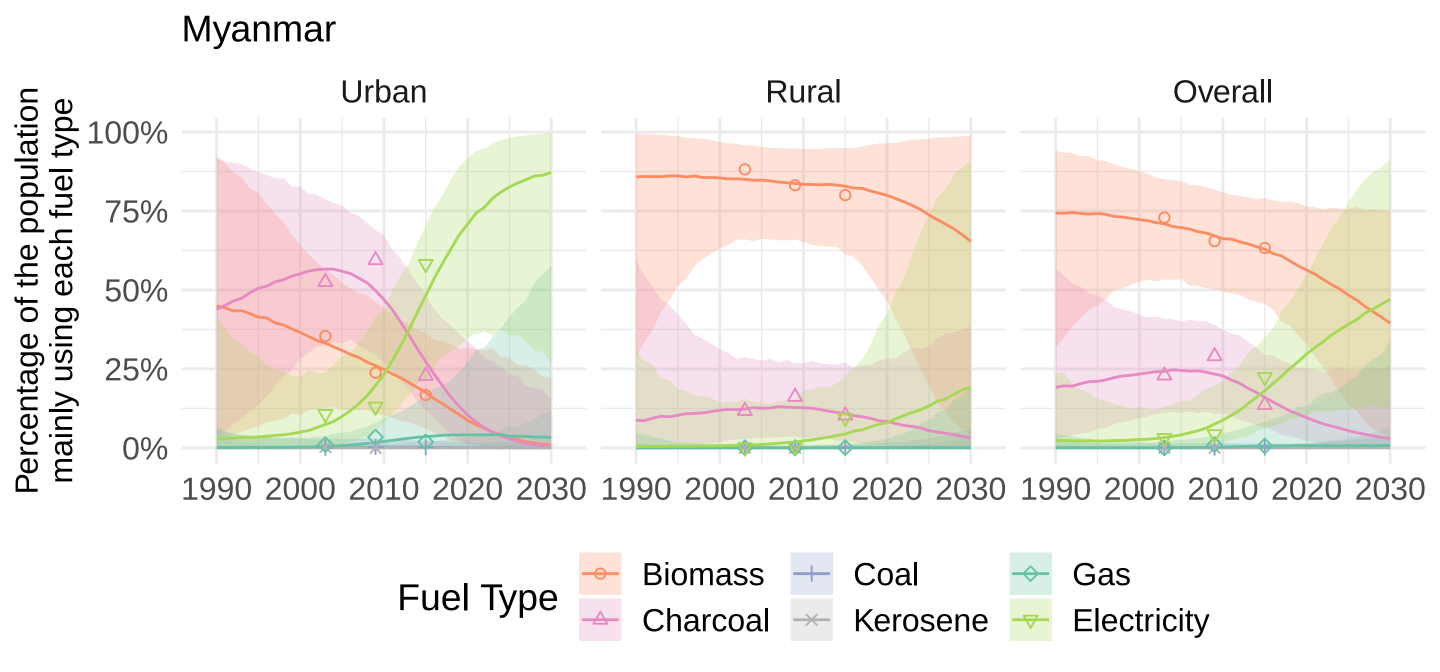
Myanmar
Urban
Rural
Overall
Percentage of the population mainly using each fuel type
100%
75%
50%
25%
0%
1990
2000
2010
2020
2030
Fuel Type
Biomass
Charcoal
Coal
Kerosene
Gas
Electricity

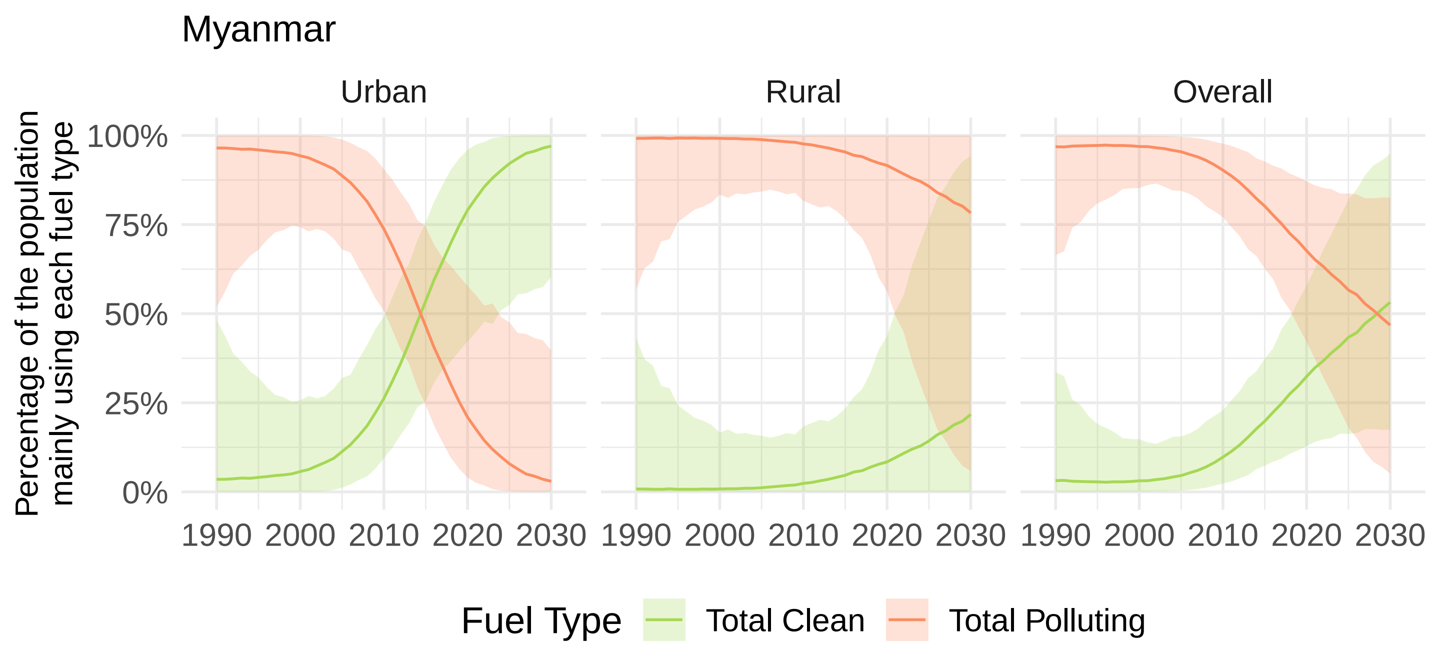
Myanmar
Urban
Rural
Overall
Percentage of the population mainly using each fuel type
100%
75%
50%
25%
0%
1990
2000
2010
2020
2030
Fuel Type
Total Clean
Total Polluting

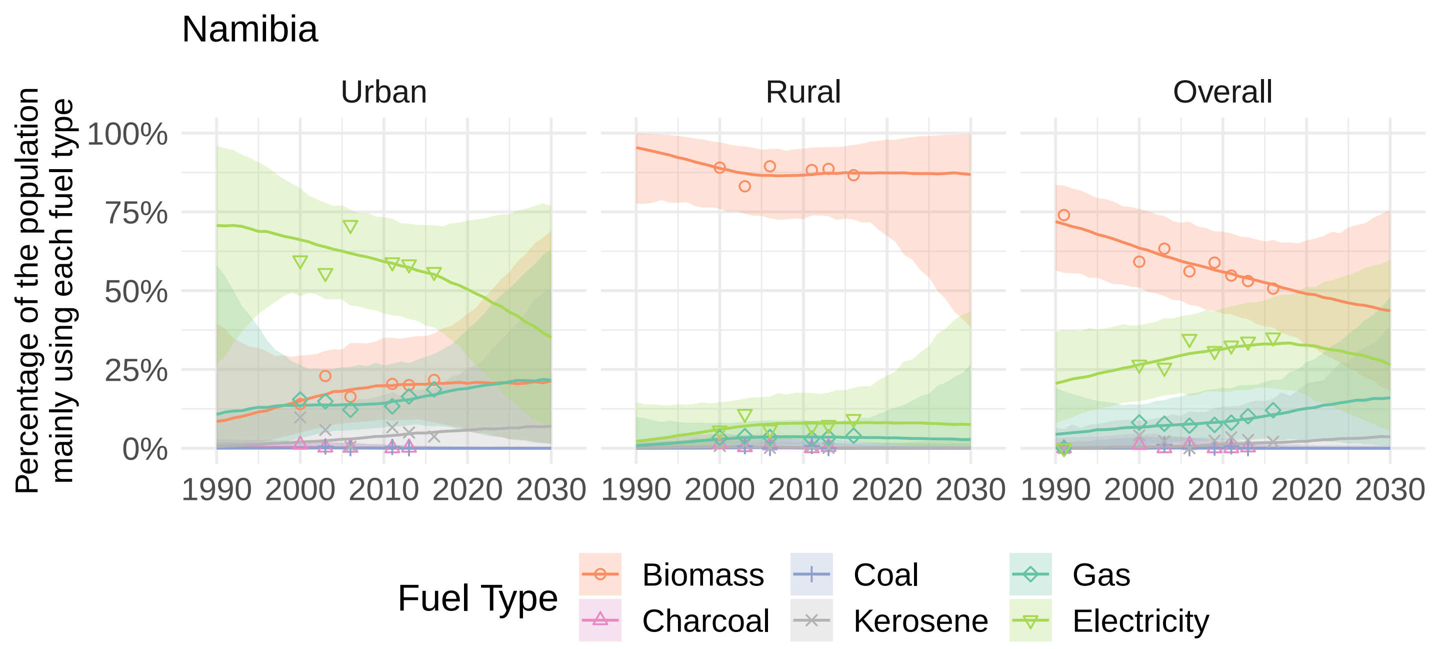

Namibia
Urban
Rural
Overall
Percentage of the population mainly using each fuel type
100%
75%
50%
25%
0%
1990
2000
2010
2020
2030
Fuel Type
Biomass
Charcoal
Coal
Kerosene
Gas
Electricity

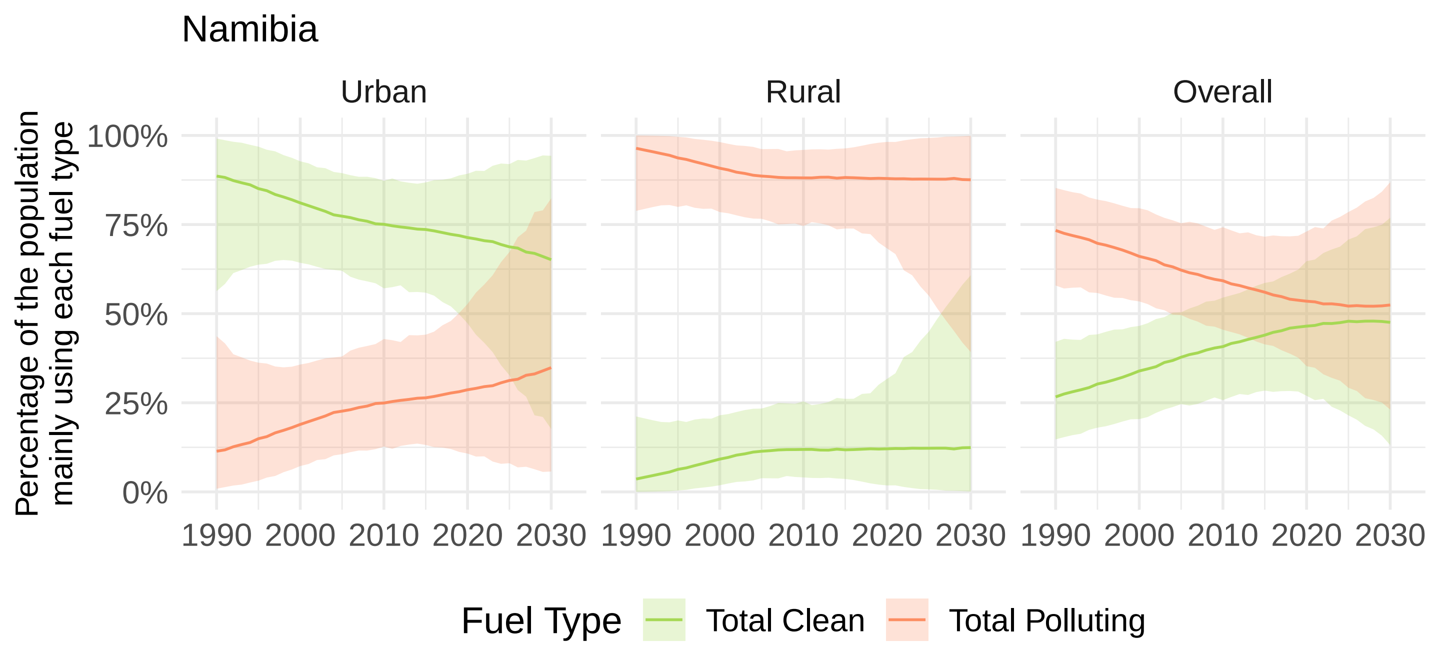

Namibia
Urban
Rural
Overall
Percentage of the population mainly using each fuel type
100%
75%
50%
25%
0%
1990
2000
2010
2020
2030
Fuel Type
Total Clean
Total Polluting

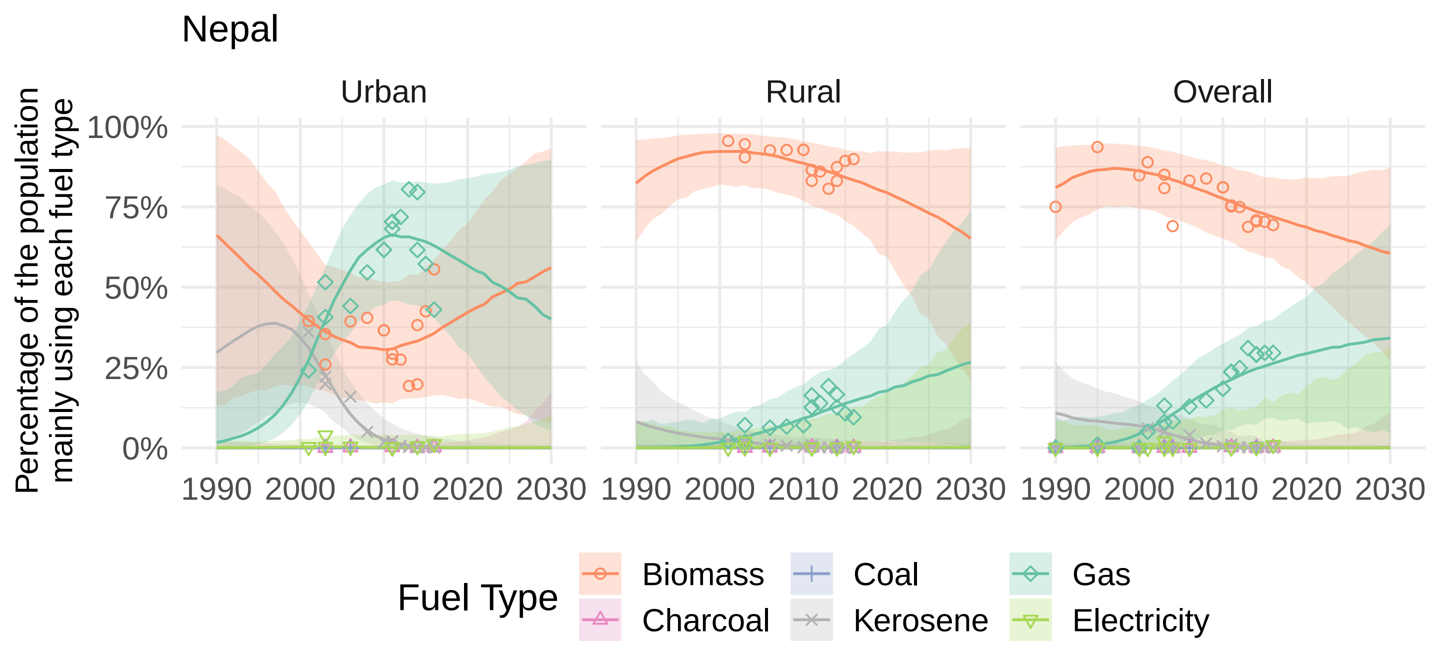
Nepal
Urban
Rural
Overall
Percentage of the population mainly using each fuel type
100%
75%
50%
25%
0%
1990
2000
2010
2020
2030
Fuel Type
Biomass
Charcoal
Coal
Kerosene
Gas
Electricity

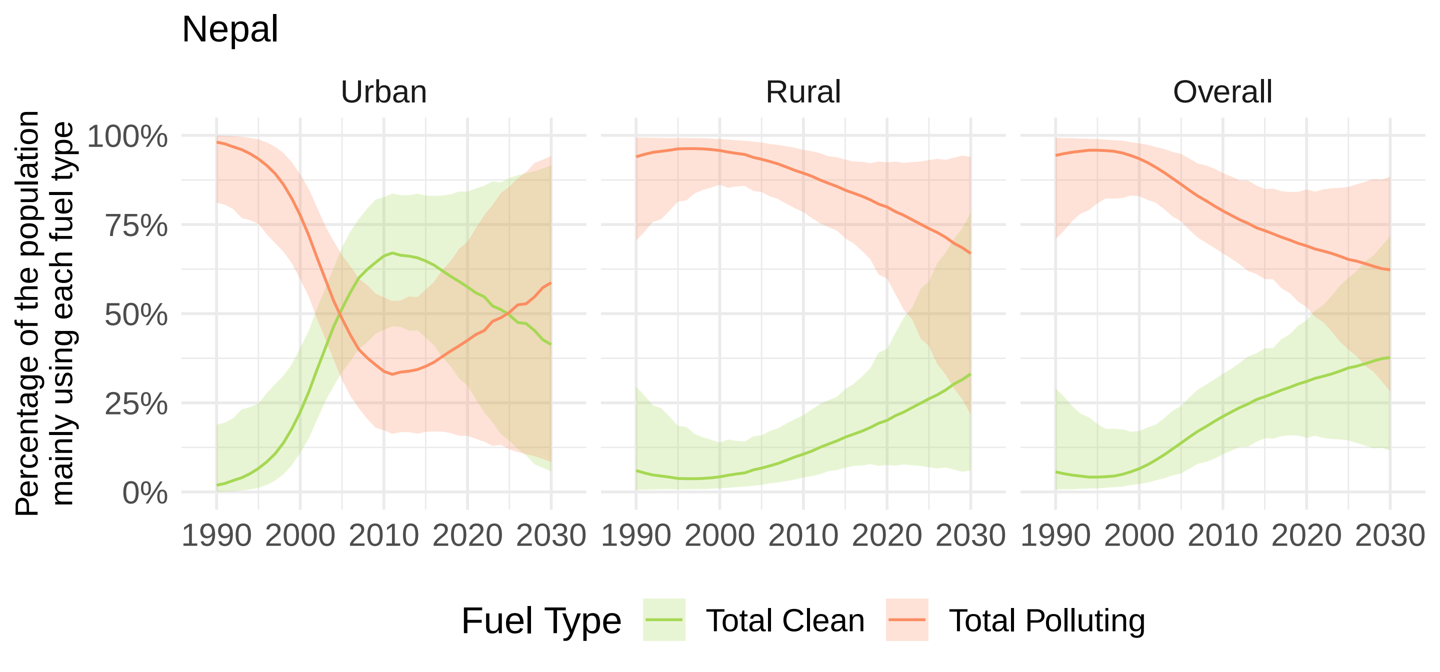
Nepal
Urban
Rural
Overall
Percentage of the population mainly using each fuel type
100%
75%
50%
25%
0%
1990
2000
2010
2020
2030
Fuel Type
Total Clean
Total Polluting

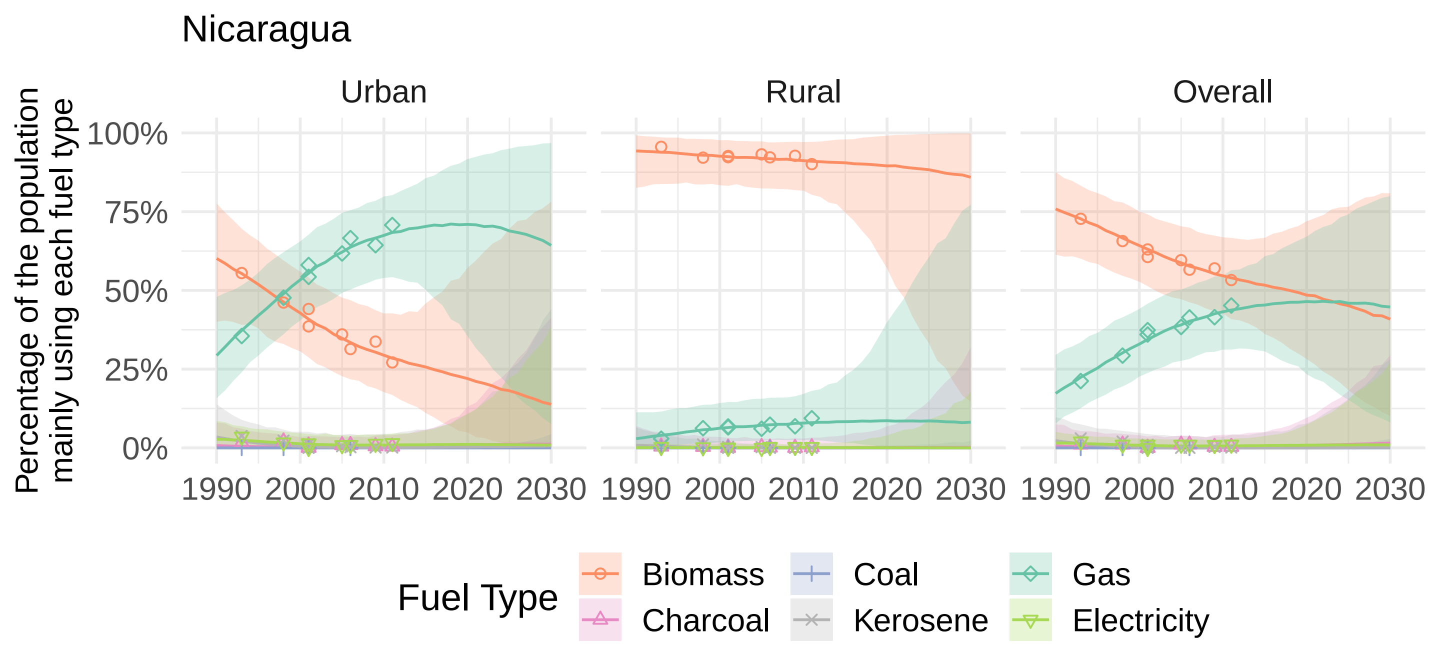
Nicaragua
Urban
Rural
Overall
Percentage of the population mainly using each fuel type
100%
75%
50%
25%
0%
1990
2000
2010
2020
2030
Fuel Type
Biomass
Charcoal
Coal
Kerosene
Gas
Electricity

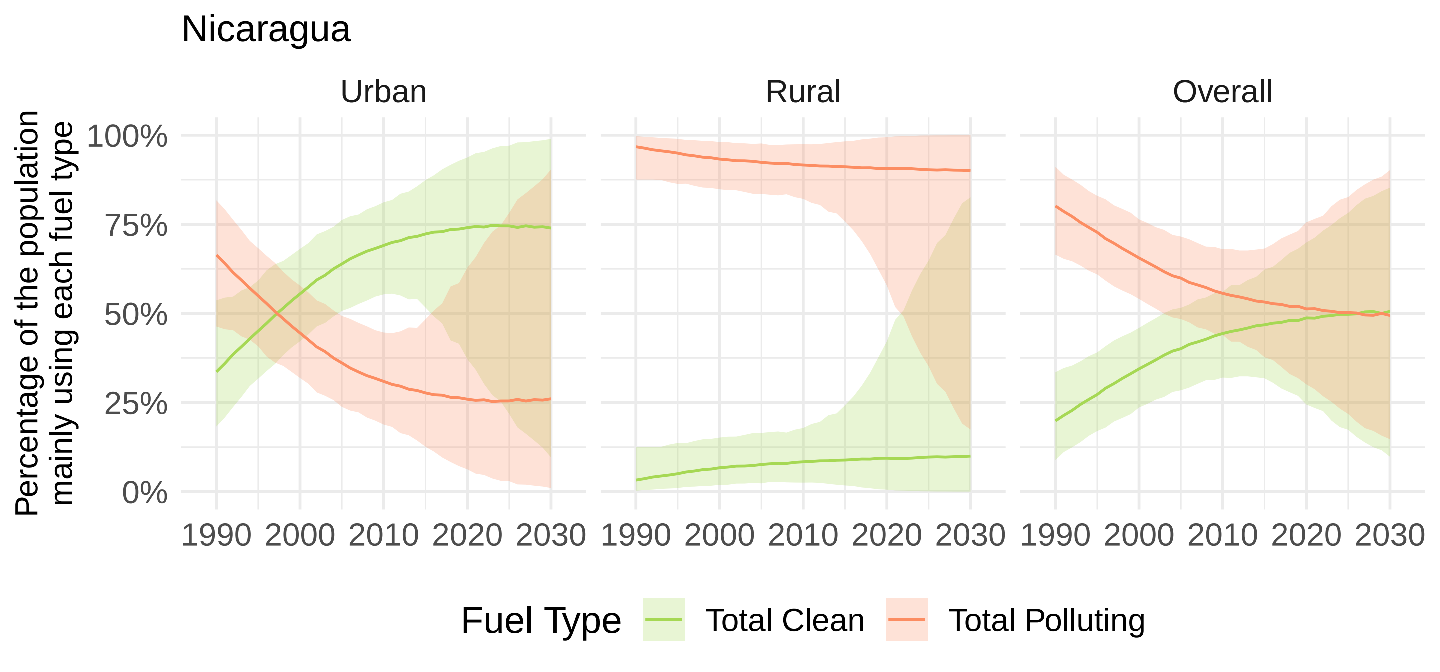

Nicaragua
Urban
Rural
Overall
Percentage of the population mainly using each fuel type
100%
75%
50%
25%
0%
1990
2000
2010
2020
2030
Fuel Type
Total Clean
Total Polluting

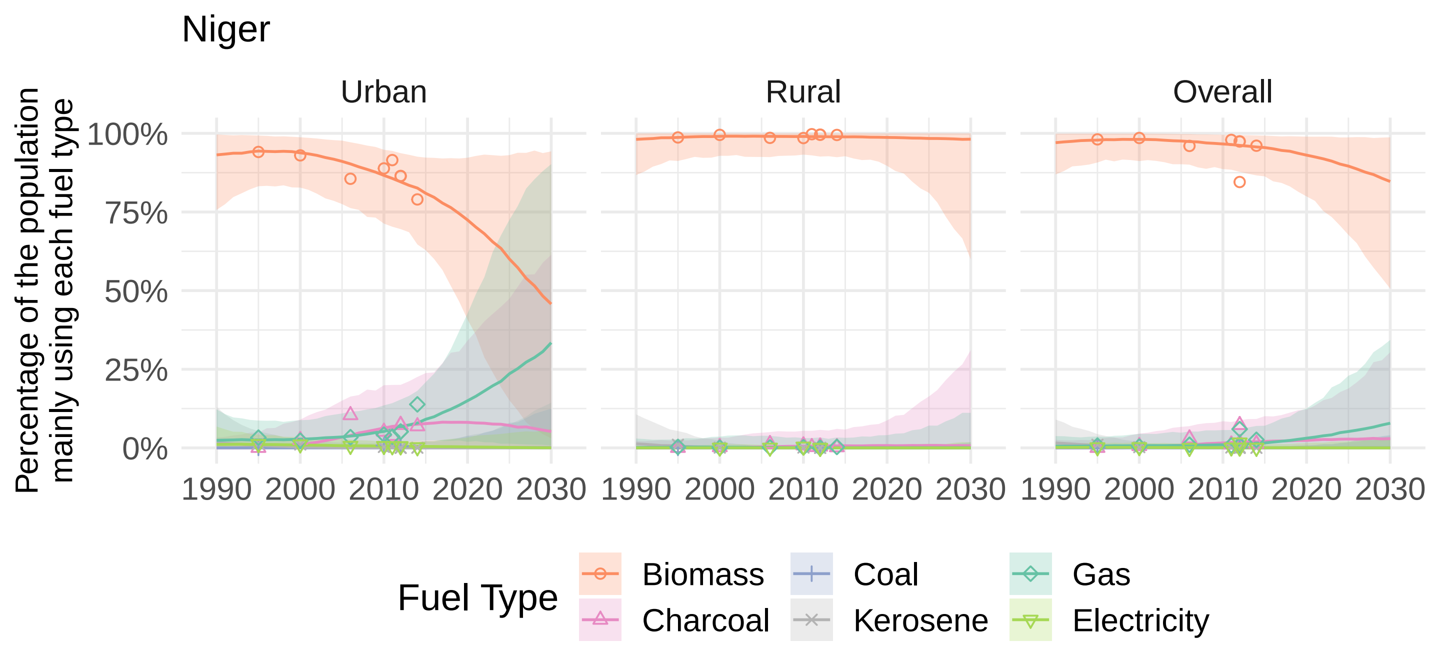
Niger
Urban
Rural
Overall
Percentage of the population mainly using each fuel type
100%
75%
50%
25%
0%
1990
2000
2010
2020
2030
Fuel Type
Biomass
Charcoal
Coal
Kerosene
Gas
Electricity

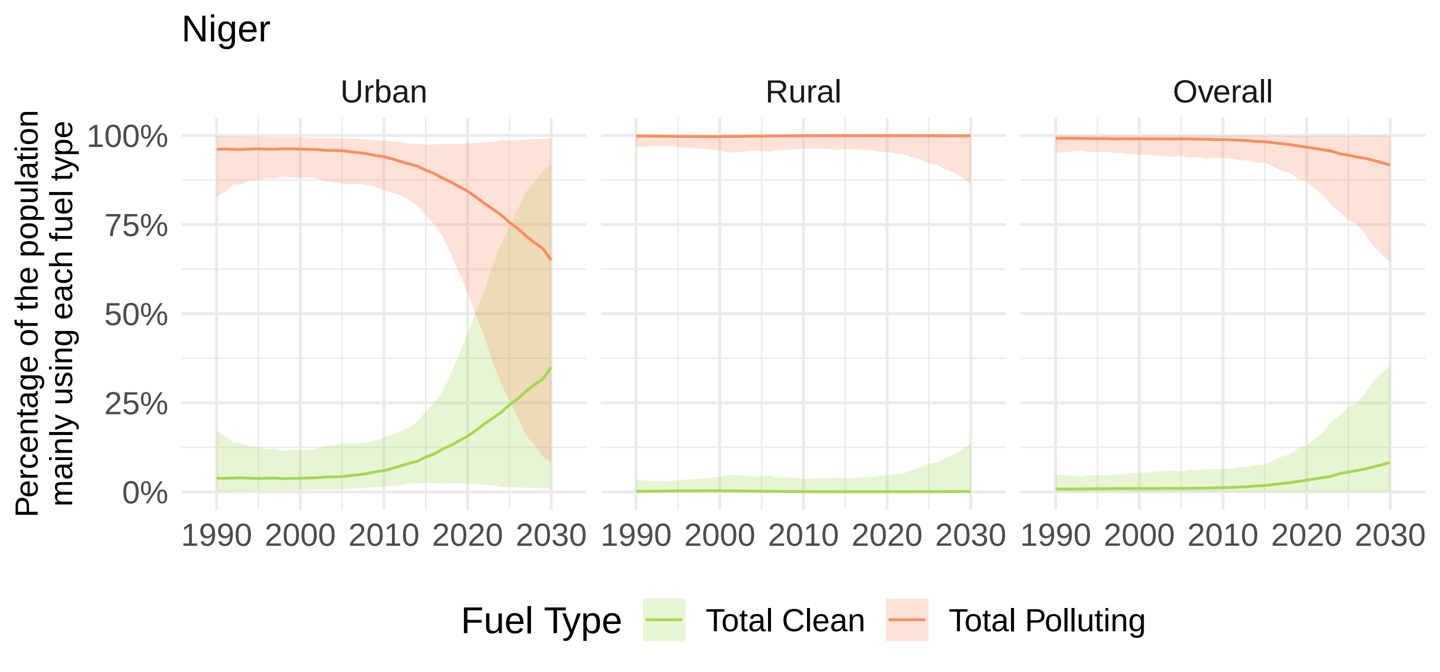

Niger
Urban
Rural
Overall
Percentage of the population mainly using each fuel type
100%
75%
50%
25%
0%
1990
2000
2010
2020
2030
Fuel Type
Total Clean
Total Polluting

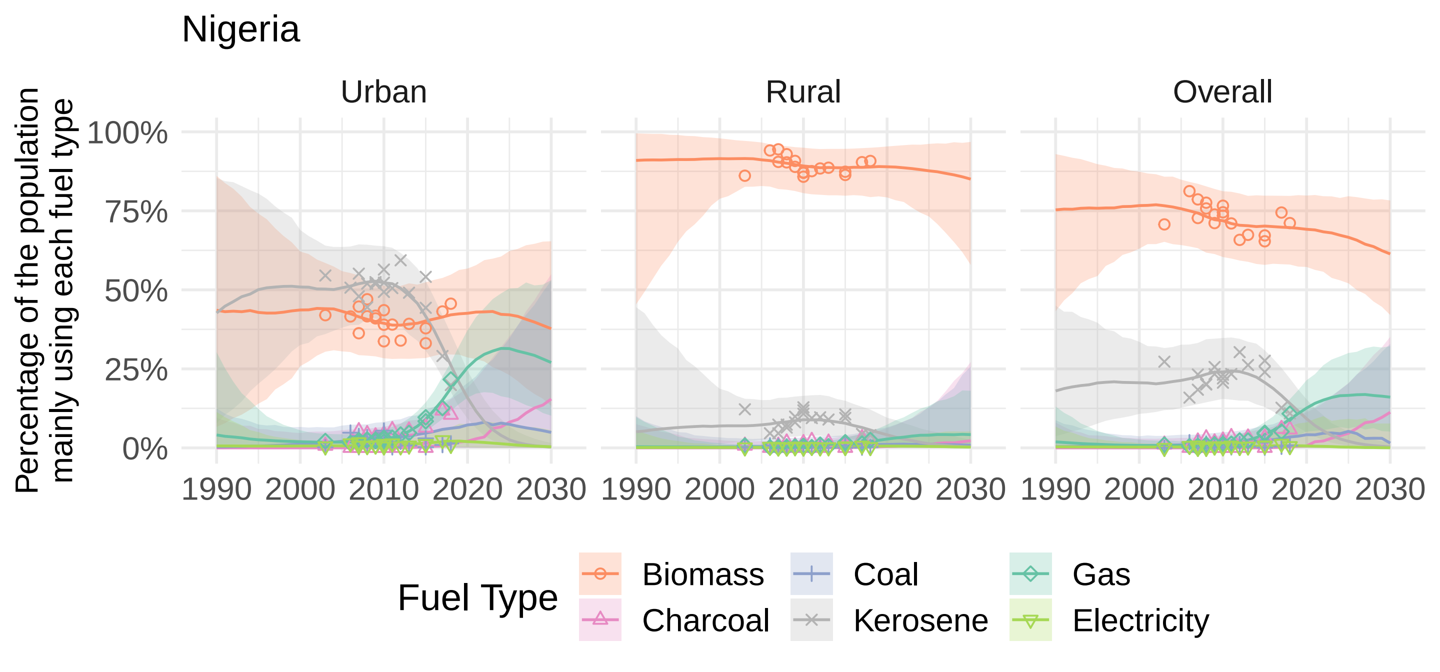

Nigeria
Urban
Rural
Overall
Percentage of the population mainly using each fuel type
100%
75%
50%
25%
0%
1990
2000
2010
2020
2030
Fuel Type
Biomass
Charcoal
Coal
Kerosene
Gas
Electricity

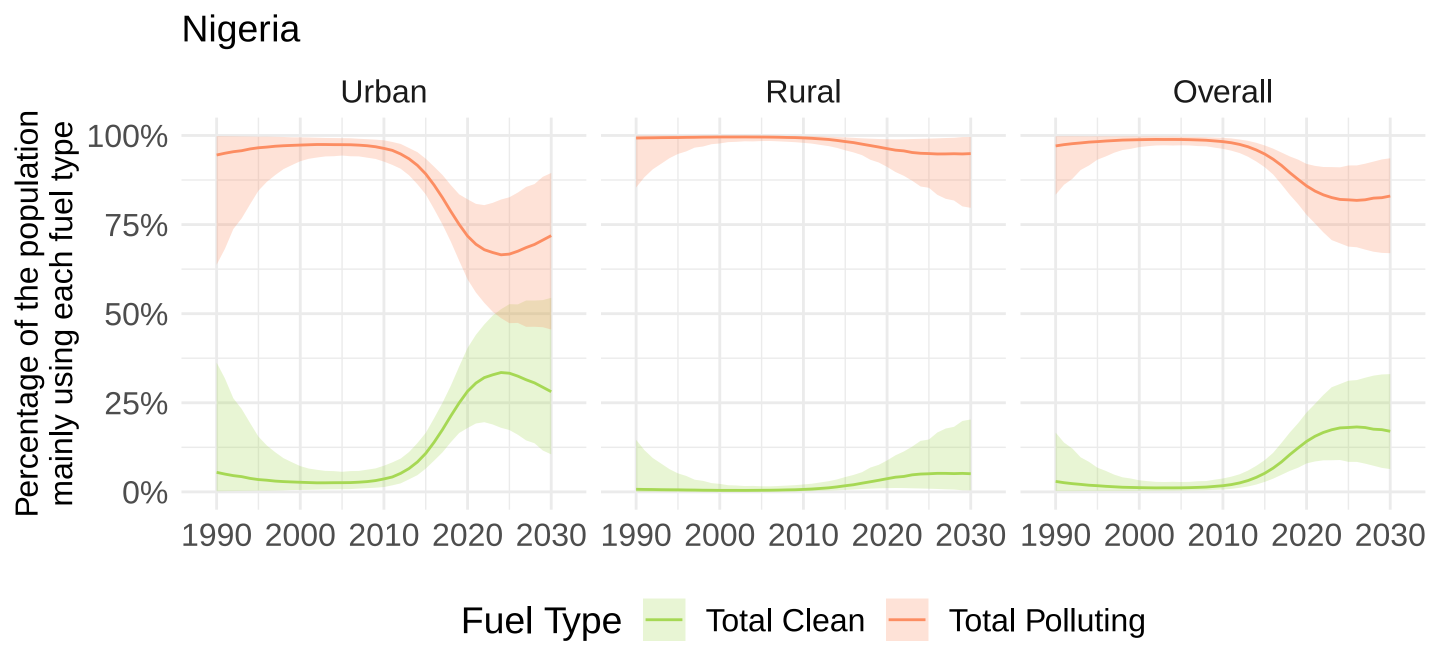

Nigeria
Urban
Rural
Overall
Percentage of the population mainly using each fuel type
100%
75%
50%
25%
0%
1990
2000
2010
2020
2030
Fuel Type
Total Clean
Total Polluting

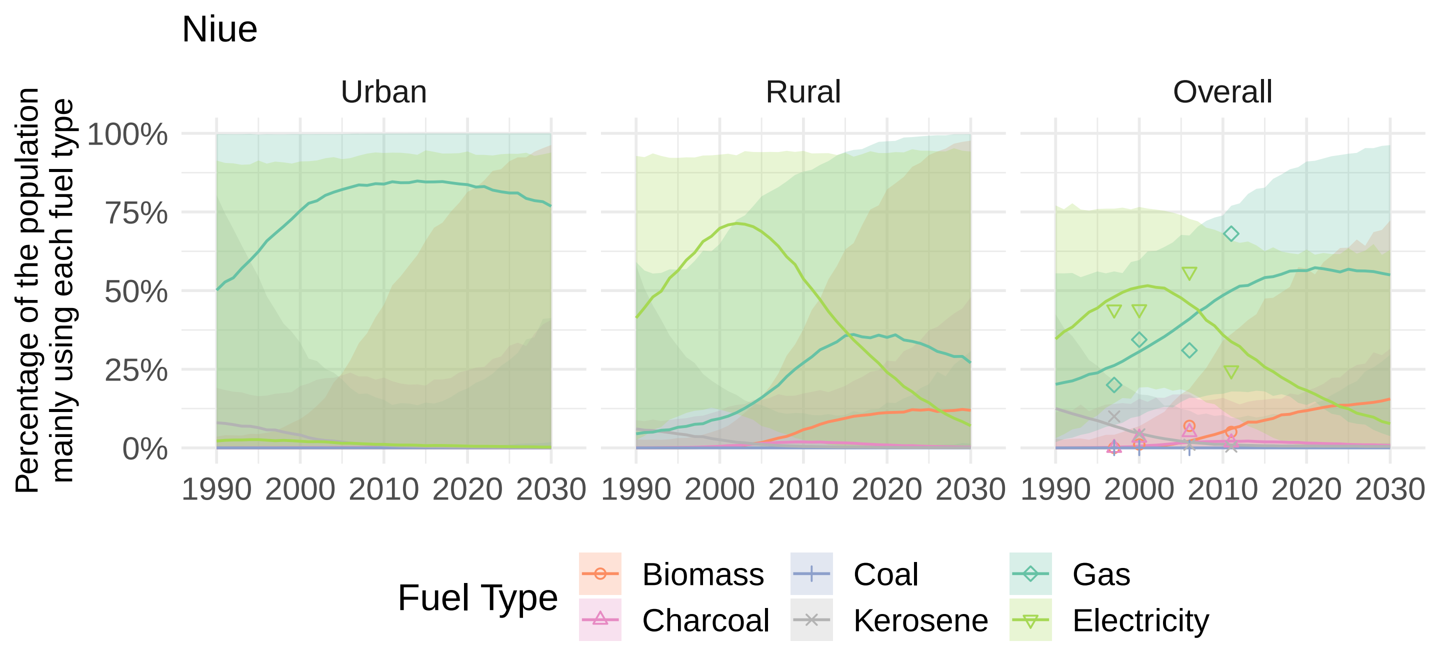
Niue
Urban
Rural
Overall
Percentage of the population mainly using each fuel type
100%
75%
50%
25%
0%
1990
2000
2010
2020
2030
Fuel Type
Biomass
Charcoal
Coal
Kerosene
Gas
Electricity

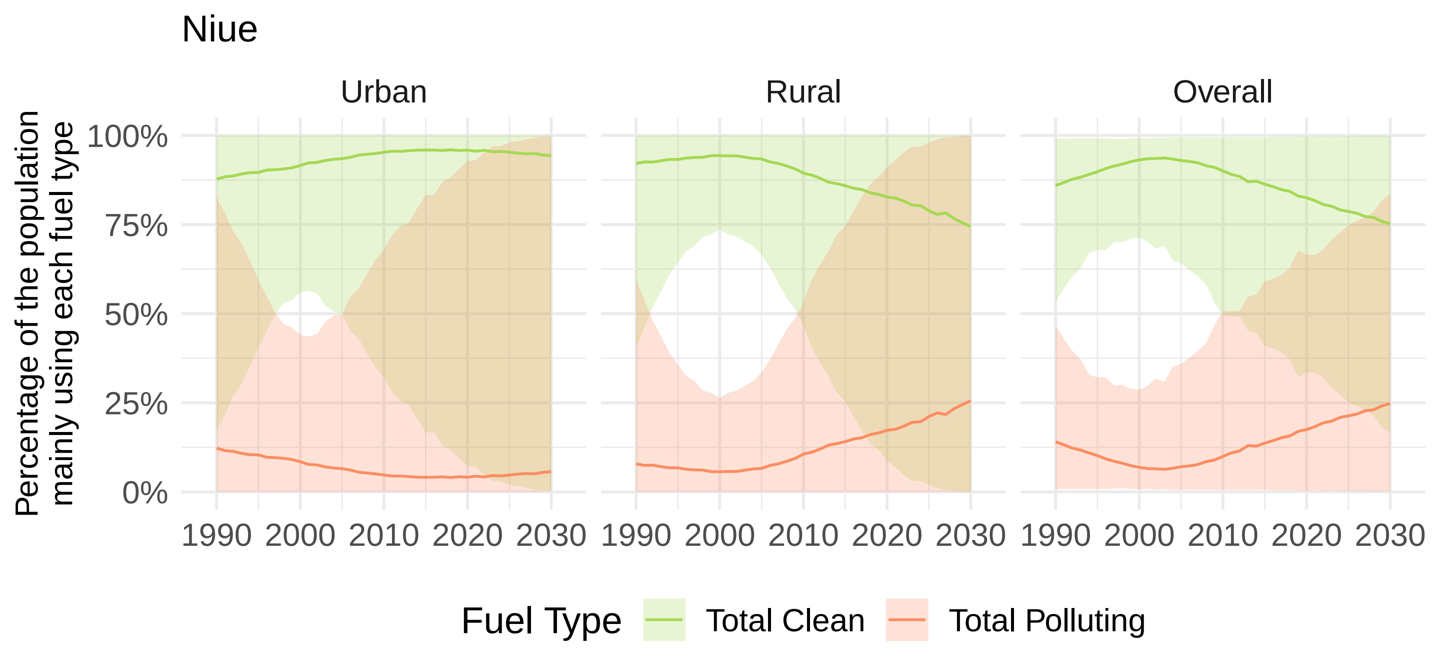
Niue
Urban
Rural
Overall
Percentage of the population mainly using each fuel type
100%
75%
50%
25%
0%
1990
2000
2010
2020
2030
Fuel Type
Total Clean
Total Polluting

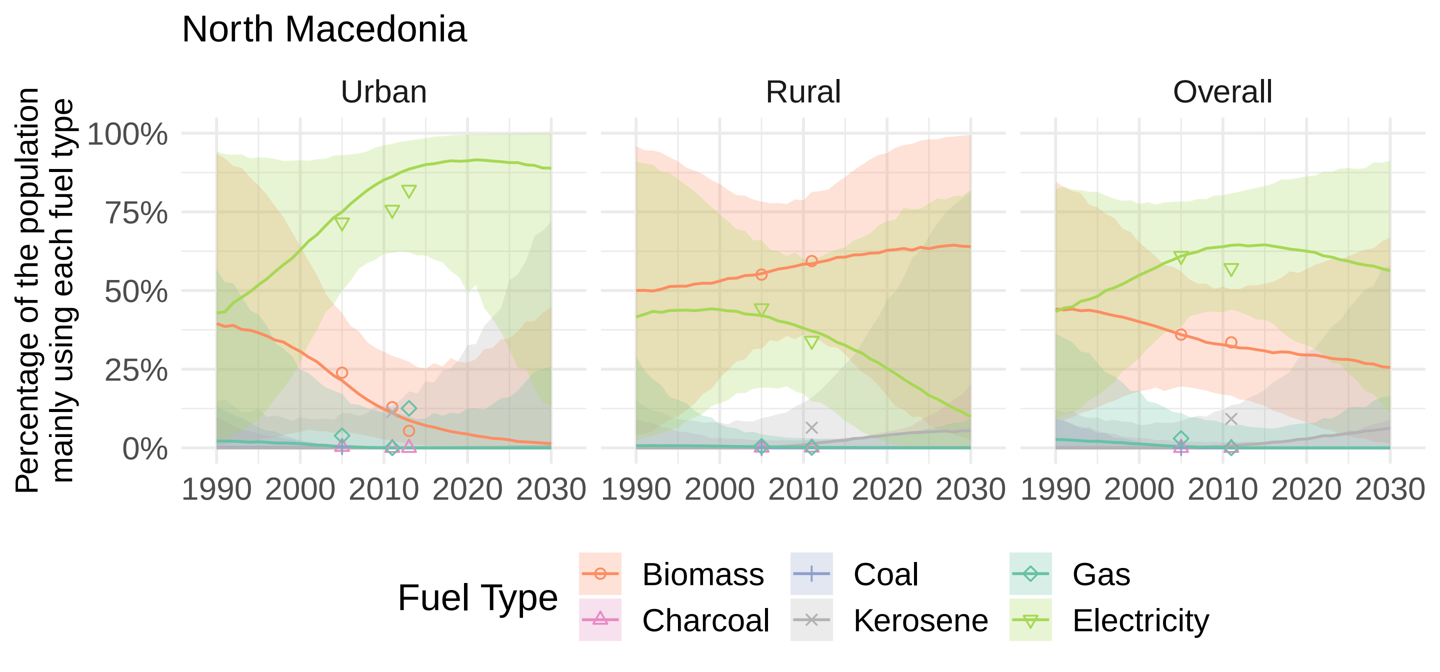
North Macedonia
Urban
Rural
Overall
Percentage of the population mainly using each fuel type
100%
75%
50%
25%
0%
1990
2000
2010
2020
2030
Fuel Type
Biomass
Charcoal
Coal
Kerosene
Gas
Electricity

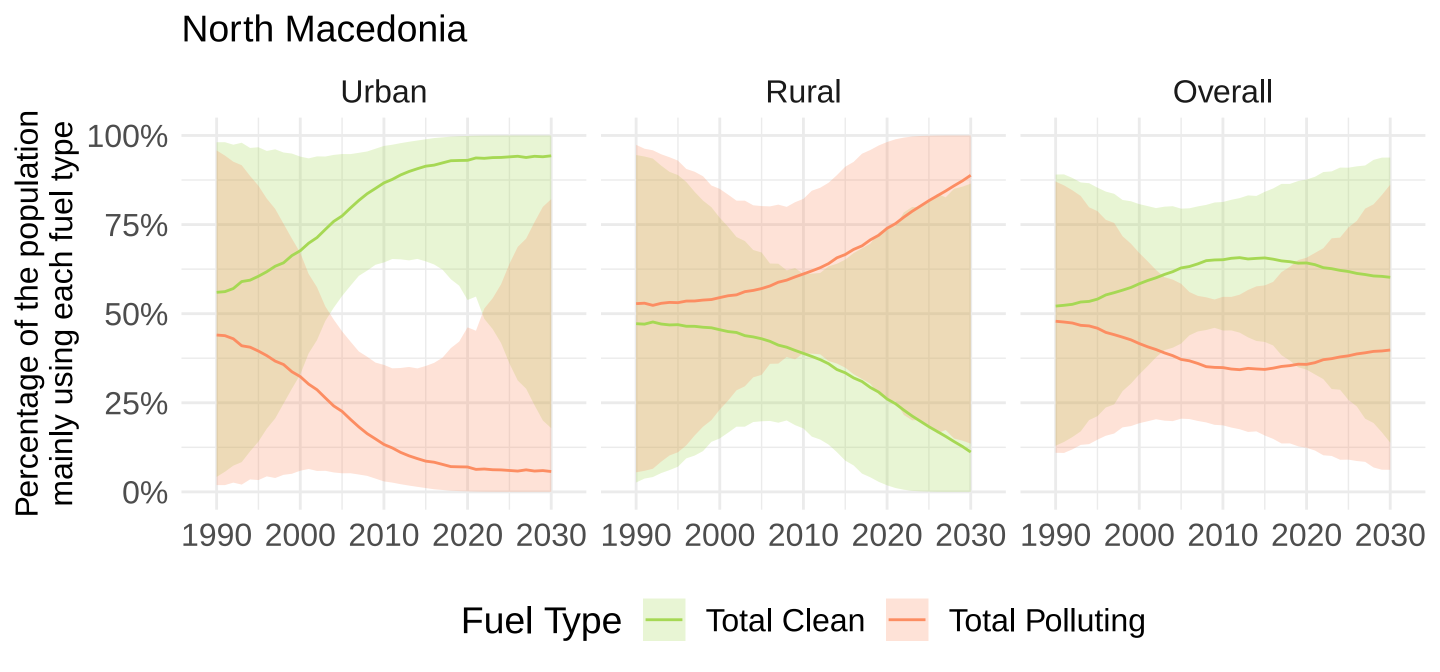
North Macedonia
Urban
Rural
Overall
Percentage of the population mainly using each fuel type
100%
75%
50%
25%
0%
1990
2000
2010
2020
2030
Fuel Type
Total Clean
Total Polluting

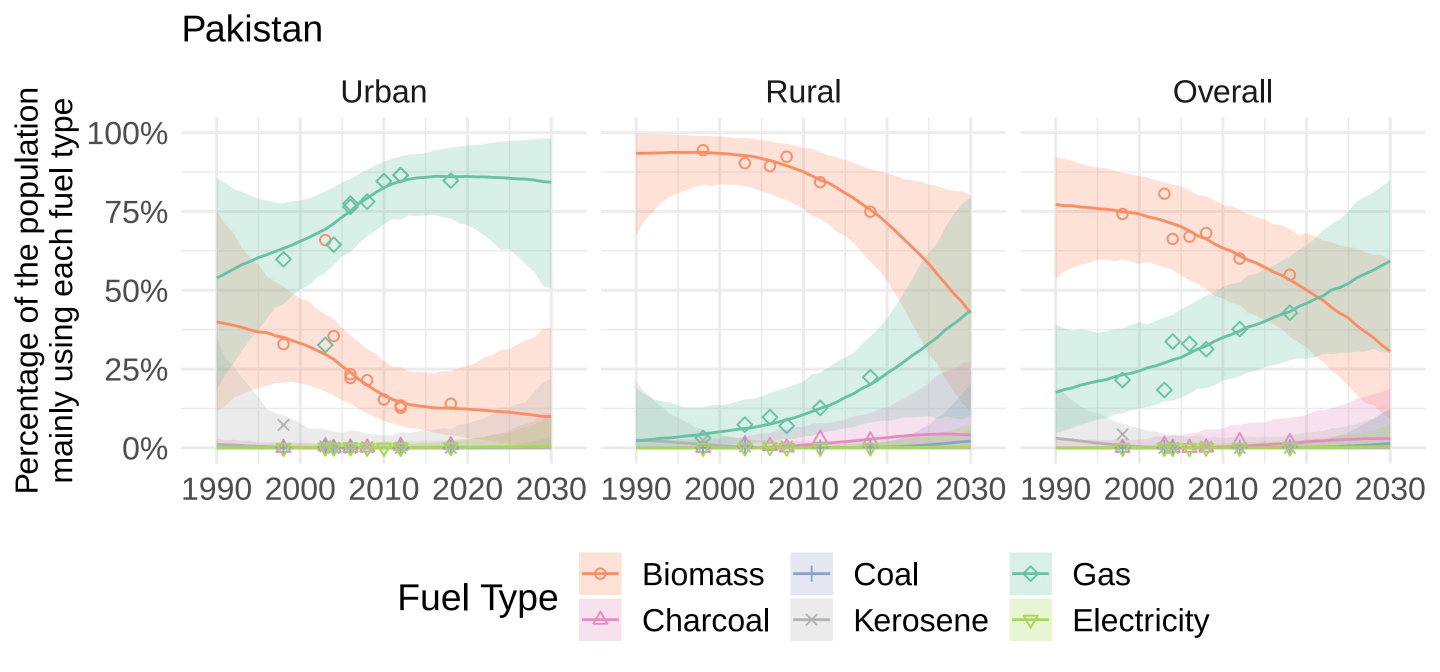

Pakistan
Urban
Rural
Overall
Percentage of the population mainly using each fuel type
100%
75%
50%
25%
0%
1990
2000
2010
2020
2030
Fuel Type
Biomass
Charcoal
Coal
Kerosene
Gas
Electricity

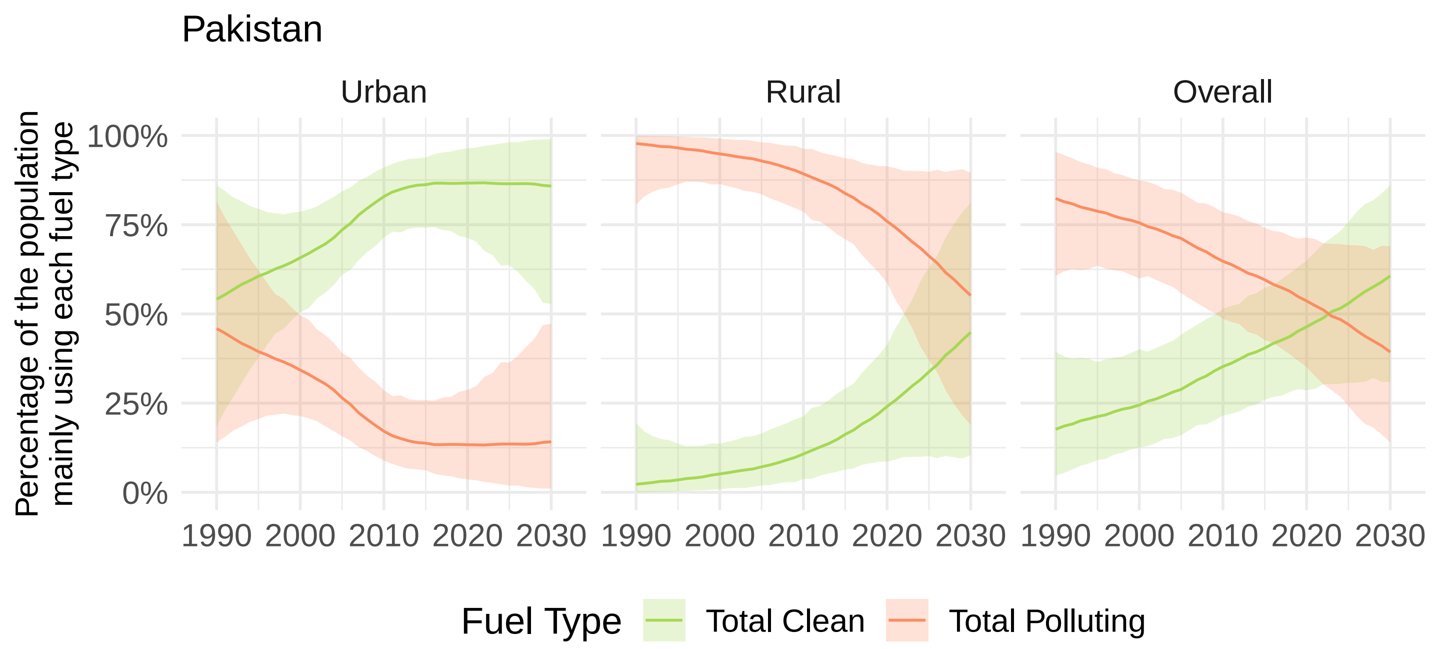

Pakistan
Urban
Rural
Overall
Percentage of the population mainly using each fuel type
100%
75%
50%
25%
0%
1990
2000
2010
2020
2030
Fuel Type
Total Clean
Total Polluting

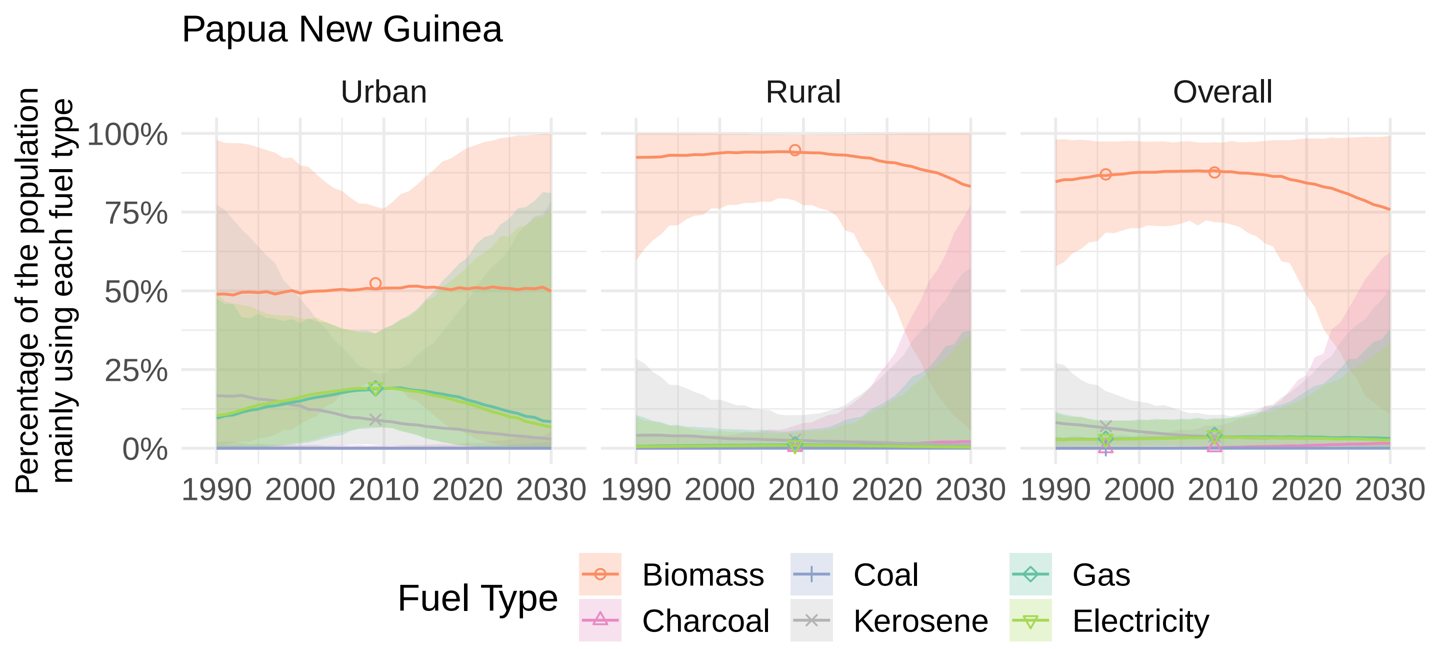
Papua New Guinea
Urban
Rural
Overall
Percentage of the population mainly using each fuel type
100%
75%
50%
25%
0%
1990
2000
2010
2020
2030
Fuel Type
Biomass
Charcoal
Coal
Kerosene
Gas
Electricity

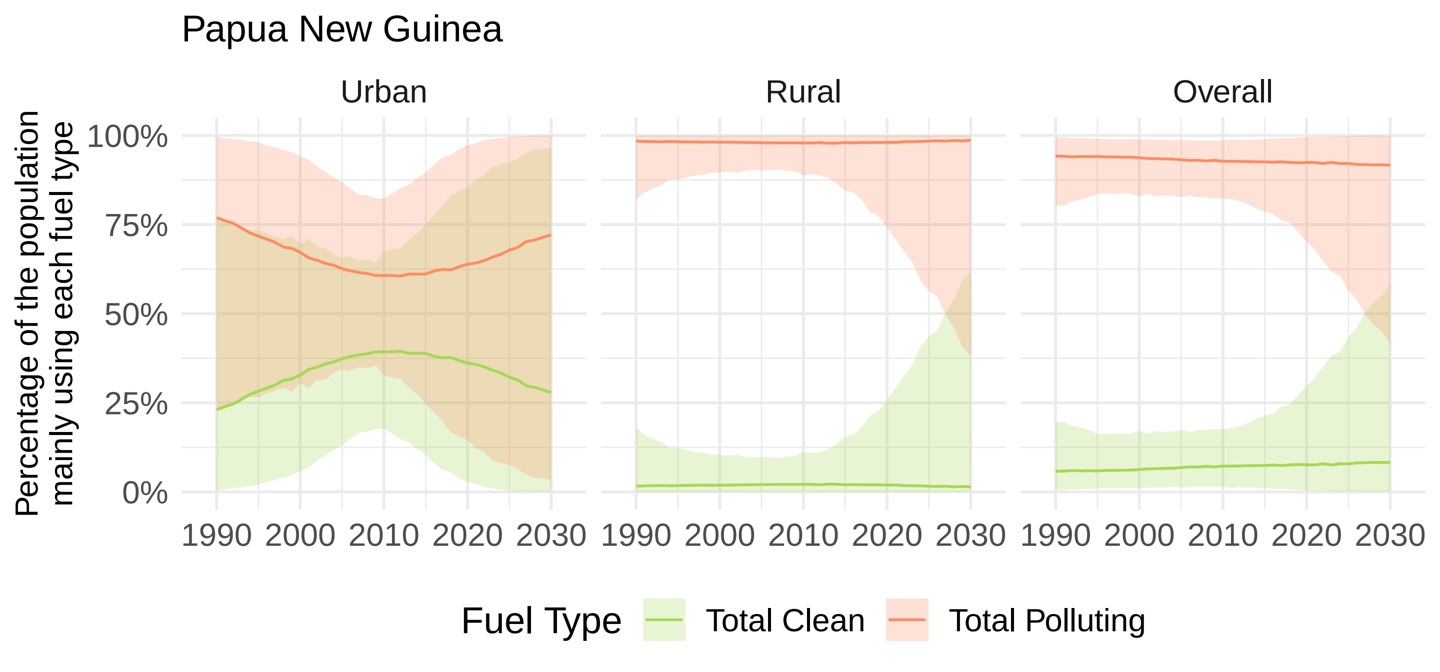
Papua New Guinea
Urban
Rural
Overall
Percentage of the population mainly using each fuel type
100%
75%
50%
25%
0%
1990
2000
2010
2020
2030
Fuel Type
Total Clean
Total Polluting

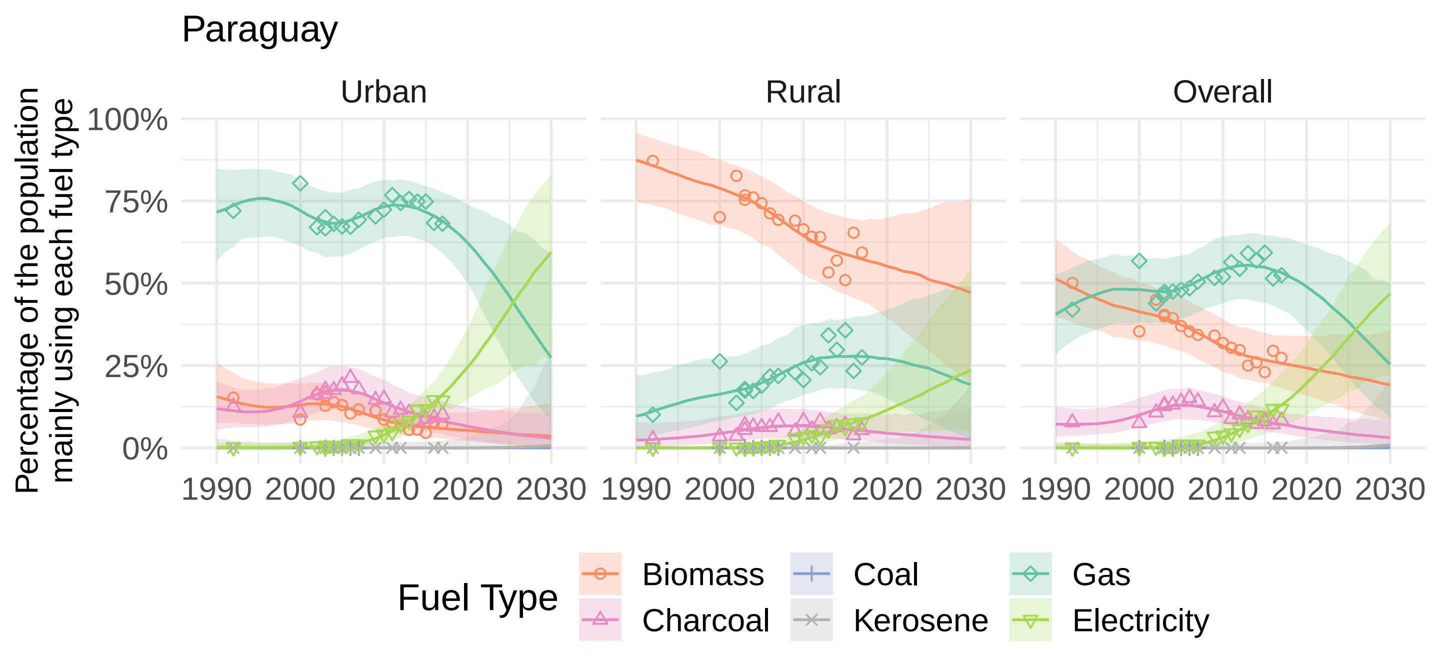

Paraguay
Urban
Rural
Overall
Percentage of the population mainly using each fuel type
100%
75%
50%
25%
0%
1990
2000
2010
2020
2030
Fuel Type
Biomass
Charcoal
Coal
Kerosene
Gas
Electricity

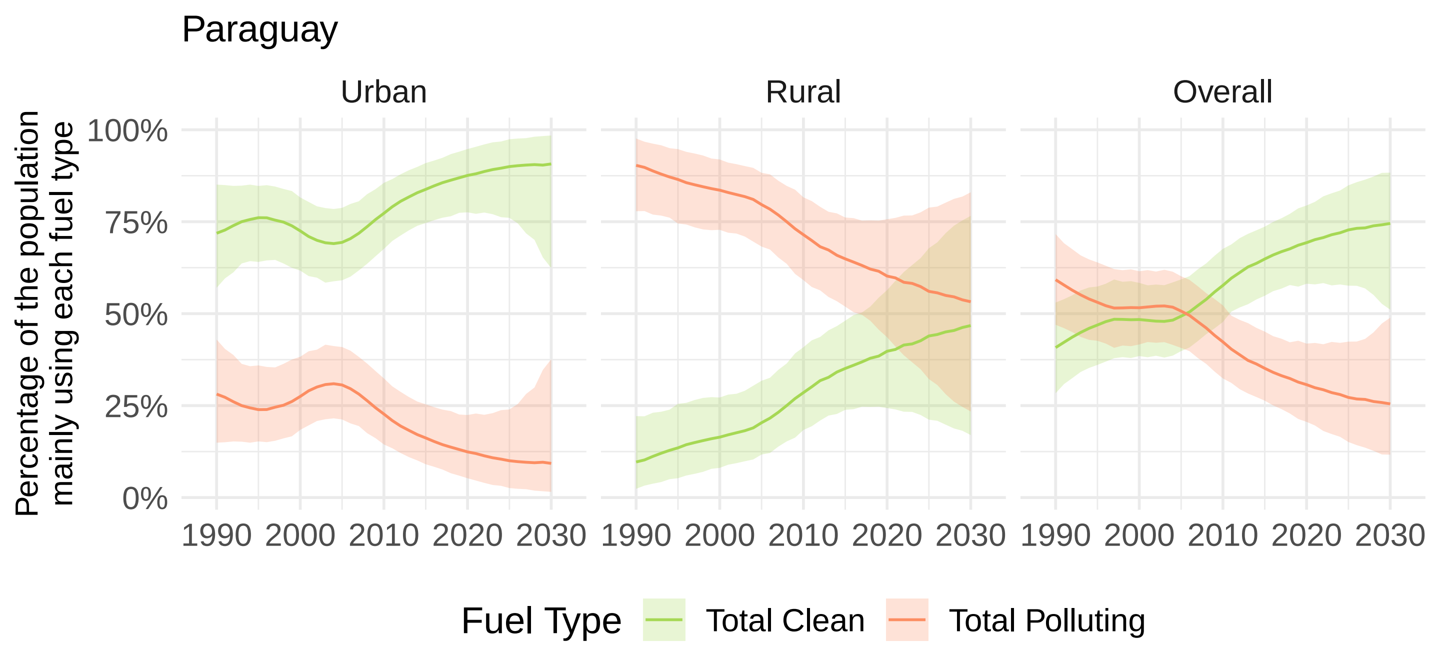

Paraguay
Urban
Rural
Overall
Percentage of the population mainly using each fuel type
100%
75%
50%
25%
0%
1990
2000
2010
2020
2030
Fuel Type
Total Clean
Total Polluting

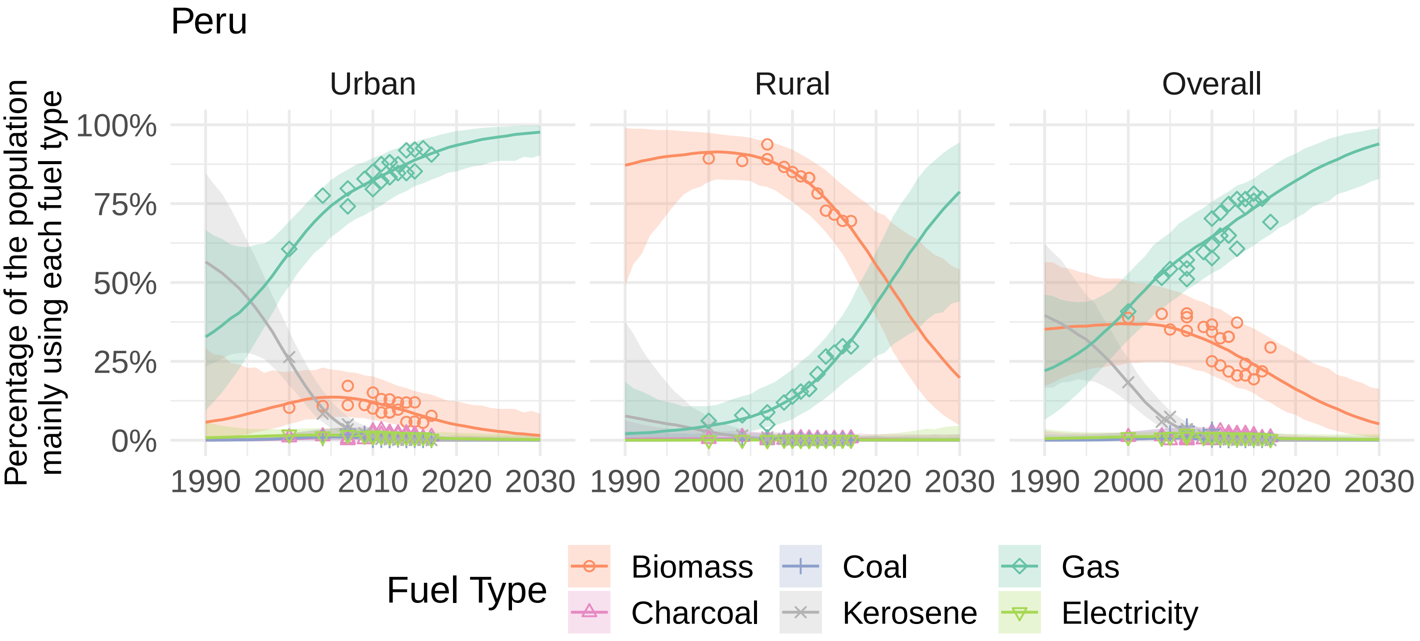

Peru
Urban
Rural
Overall
Percentage of the population mainly using each fuel type
100%
75%
50%
25%
0%
1990
2000
2010
2020
2030
Fuel Type
Biomass
Charcoal
Coal
Kerosene
Gas
Electricity

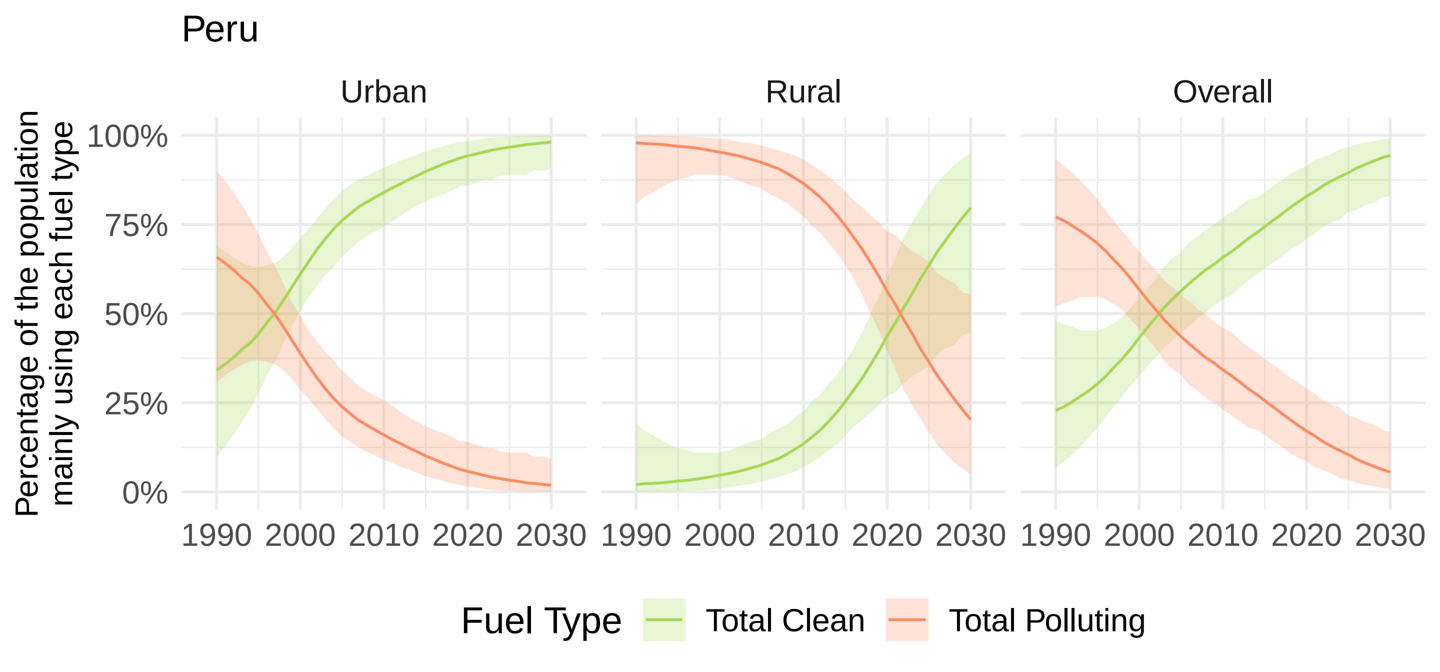
Peru
Urban
Rural
Overall
Percentage of the population mainly using each fuel type
100%
75%
50%
25%
0%
1990
2000
2010
2020
2030
Fuel Type
Total Clean
Total Polluting

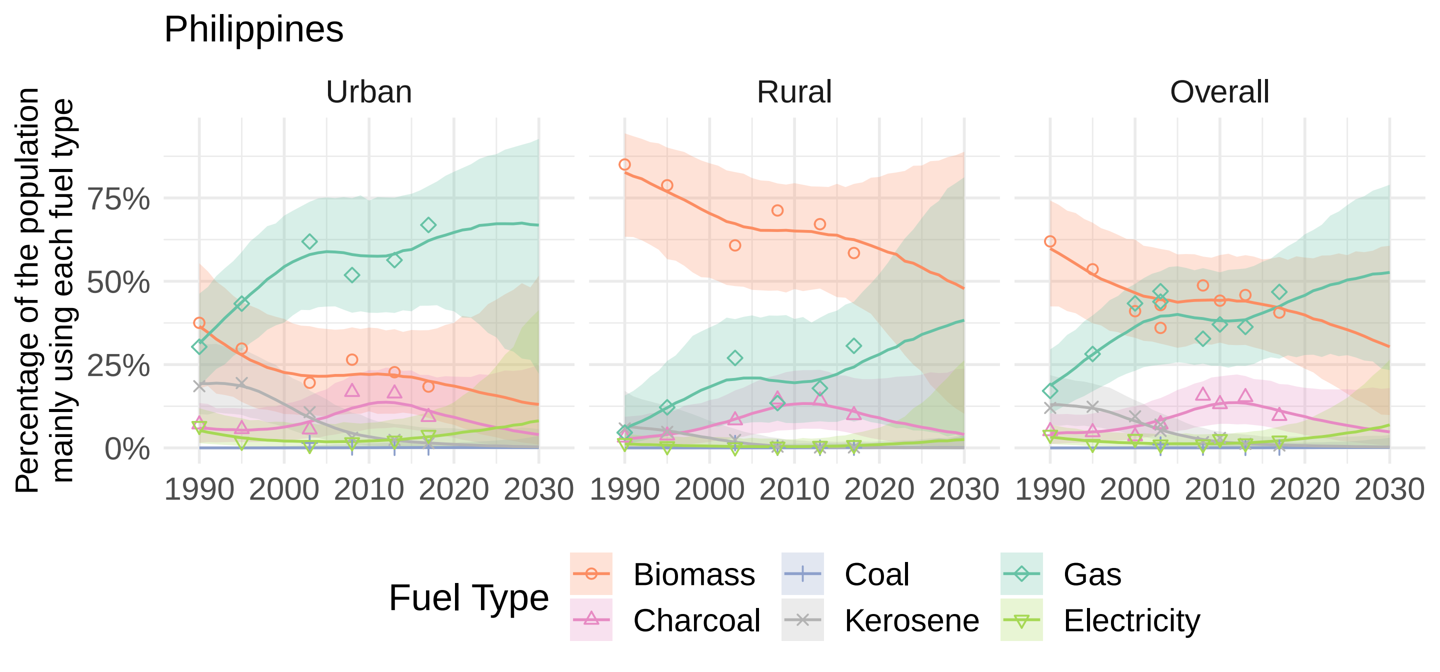

Philippines
Urban
Rural
Overall
Percentage of the population mainly using each fuel type
0%
25%
50%
75%
1990
2000
2010
2020
2030
Fuel Type
Biomass
Charcoal
Coal
Kerosene
Gas
Electricity

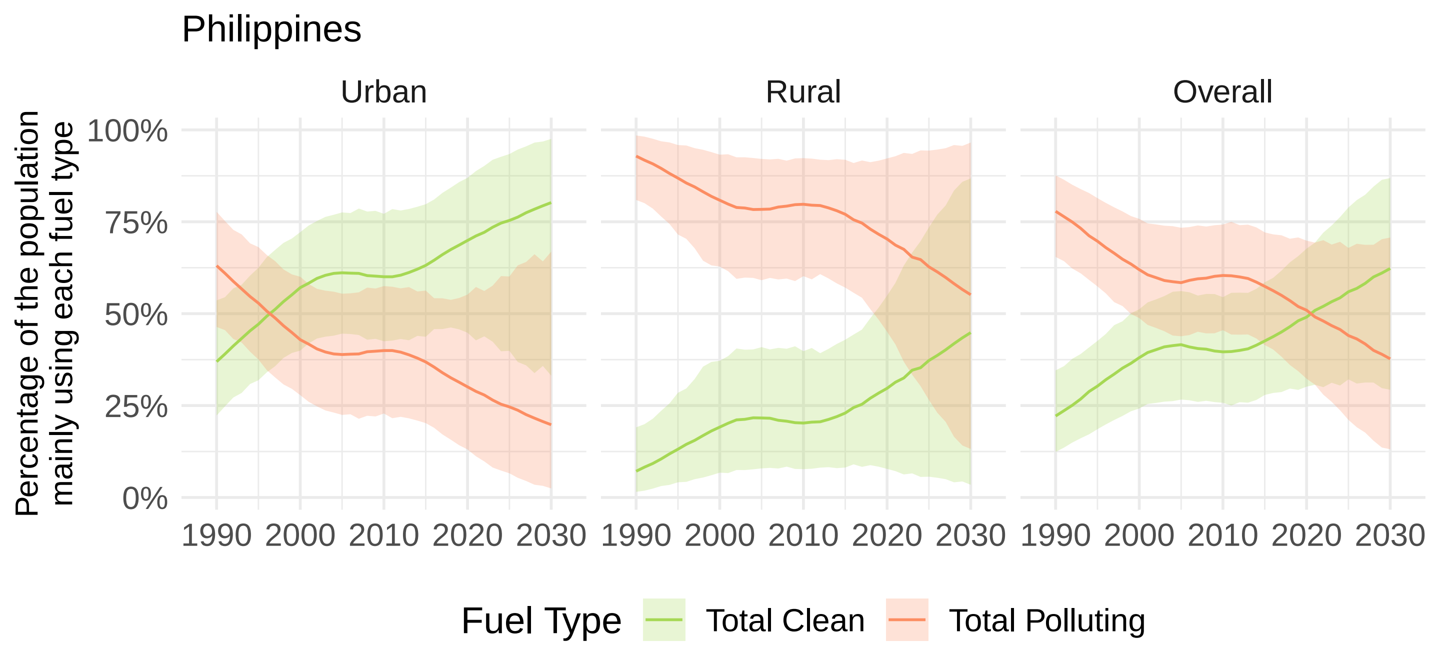

Philippines
Urban
Rural
Overall
Percentage of the population mainly using each fuel type
100%
75%
50%
25%
0%
1990
2000
2010
2020
2030
Fuel Type
Total Clean
Total Polluting

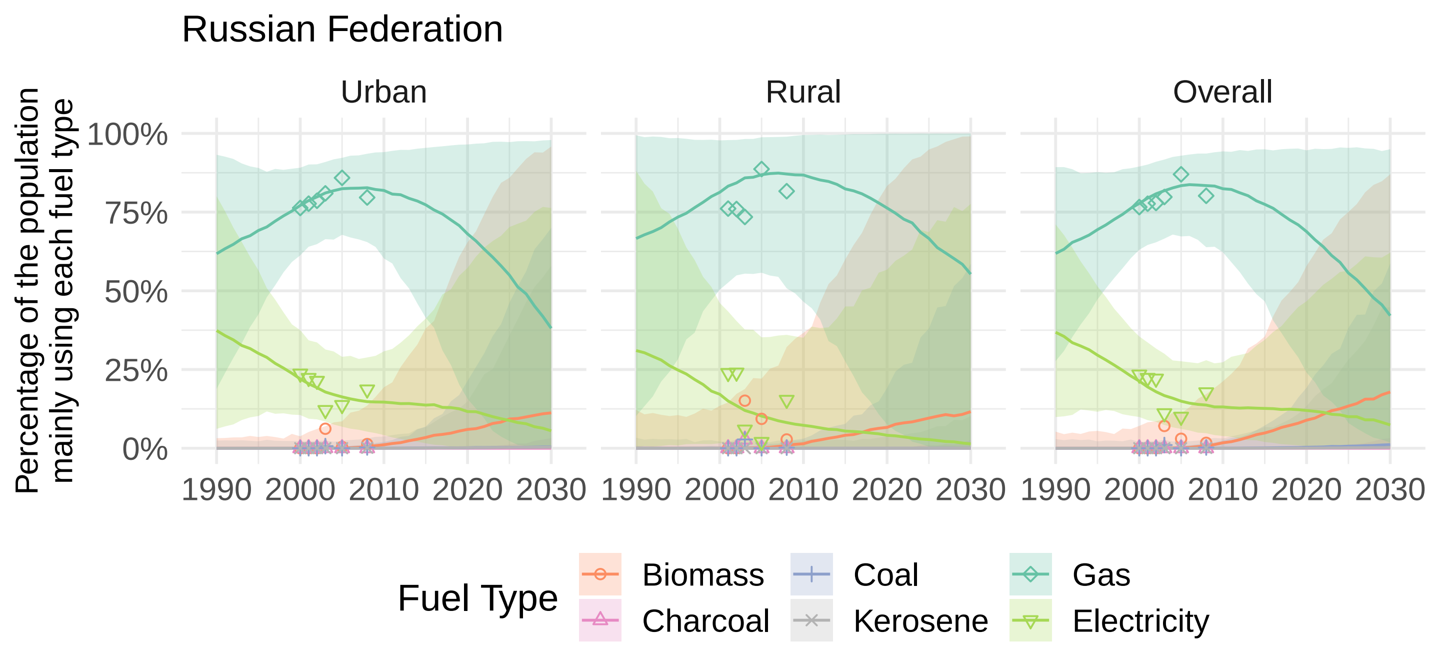
Russian Federation
Urban
Rural
Overall
Percentage of the population
mainly using each fuel type
100%
75%
50%
25%
0%
1990
2000
2010
2020
2030
Fuel Type
Biomass
Charcoal
Coal
Kerosene
Gas
Electricity

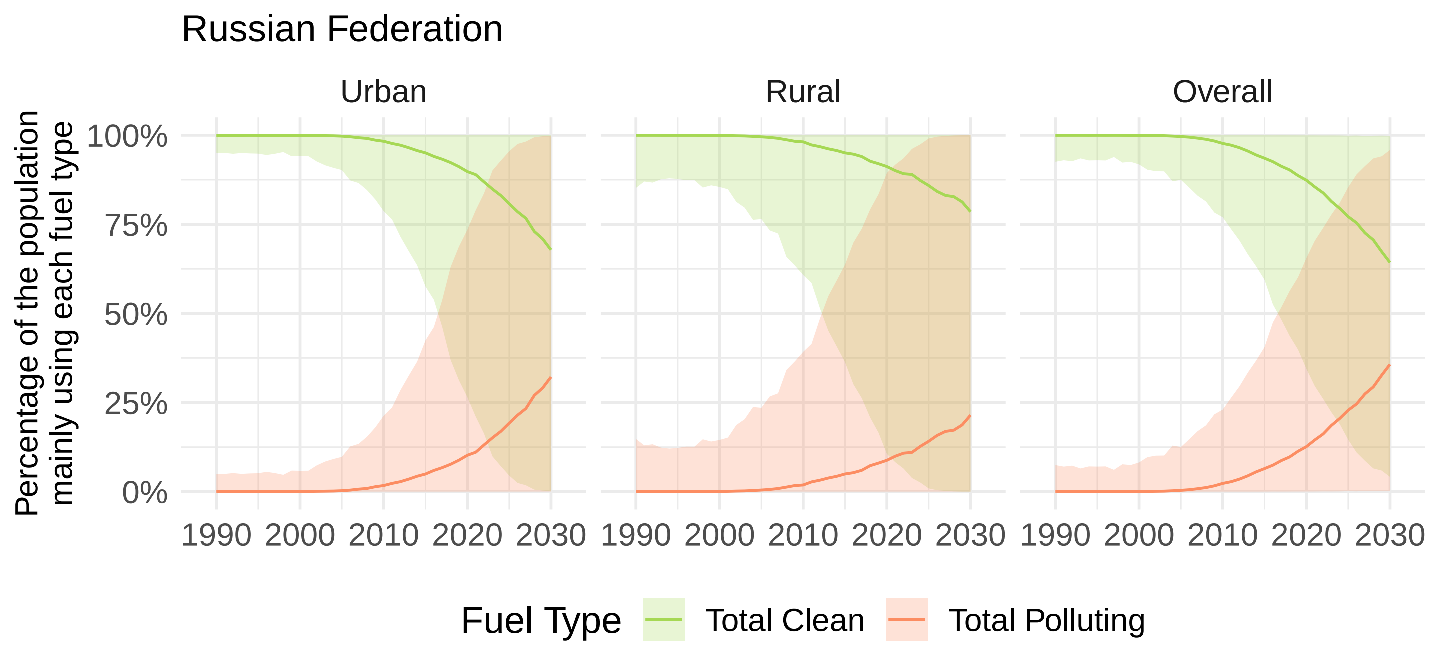
Russian Federation
Urban
Rural
Overall
Percentage of the population mainly using each fuel type
100%
75%
50%
25%
0%
1990
2000
2010
2020
2030
Fuel Type
Total Clean
Total Polluting

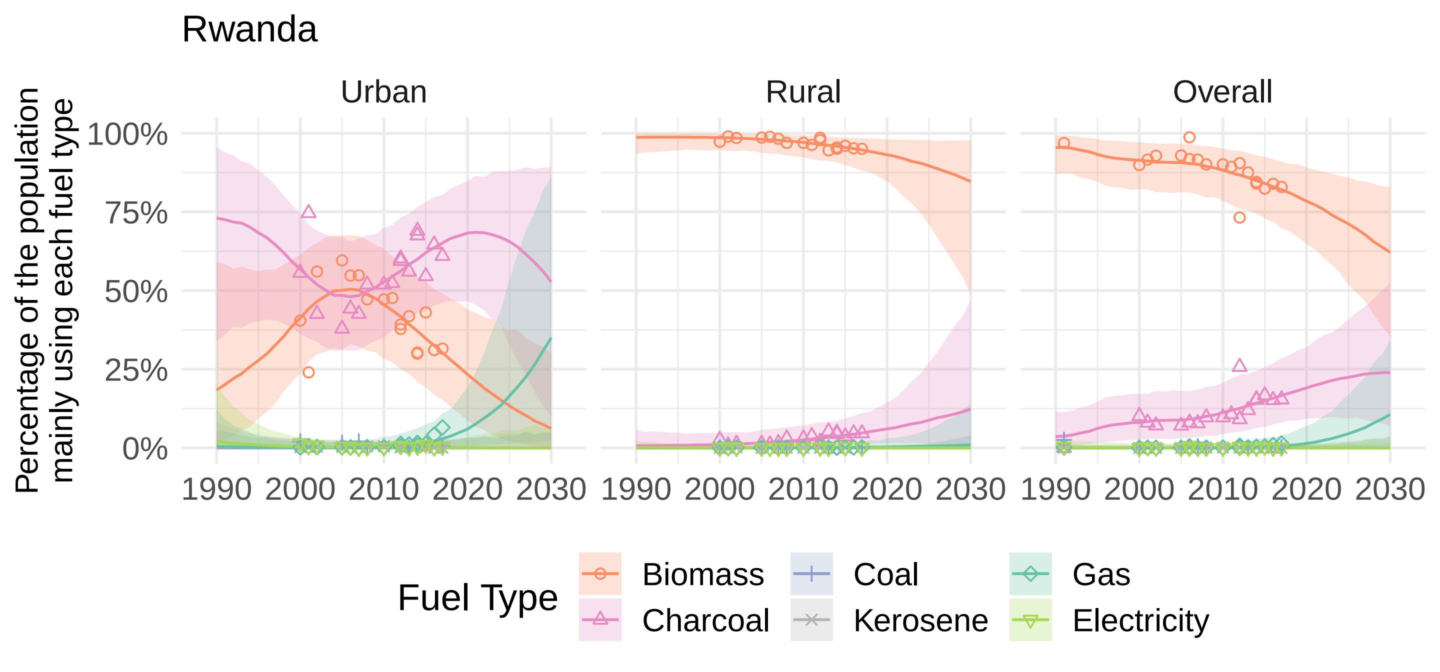
Rwanda
Urban
Rural
Overall
Percentage of the population mainly using each fuel type
100%
75%
50%
25%
0%
1990
2000
2010
2020
2030
Fuel Type
Biomass
Charcoal
Coal
Kerosene
Gas
Electricity

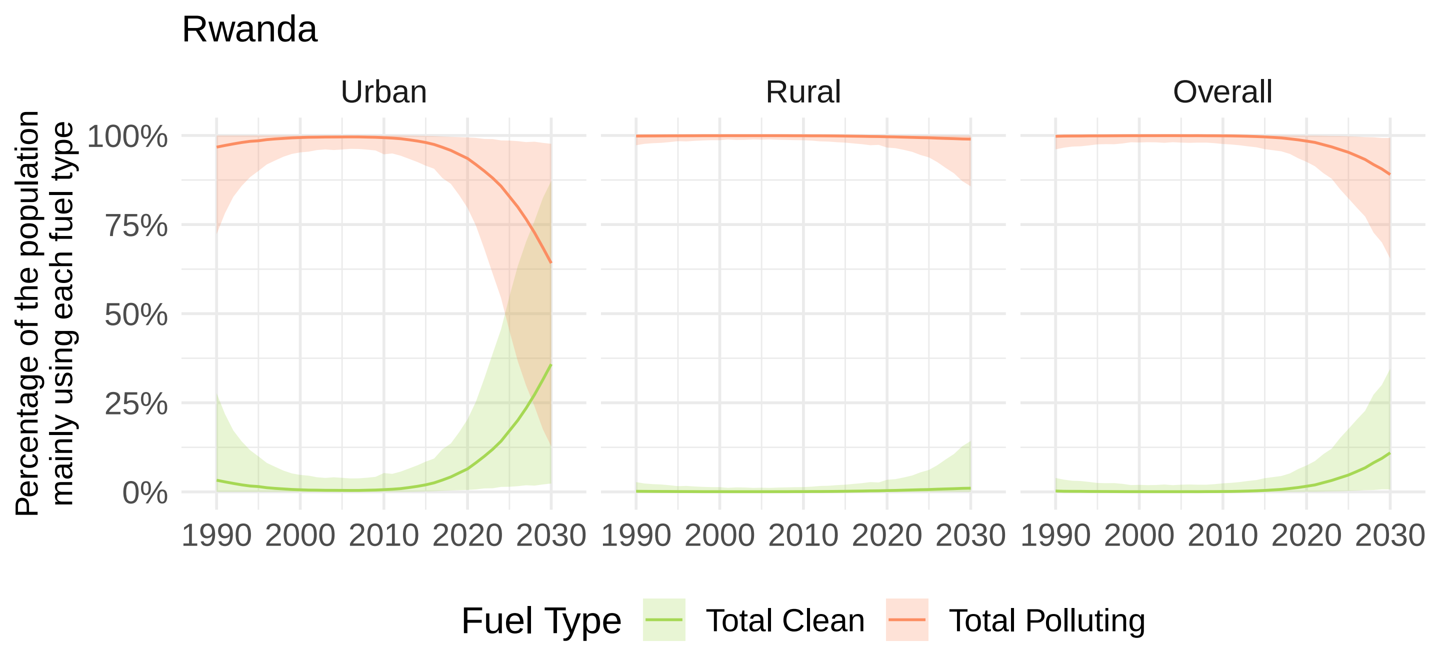

Rwanda
Urban
Rural
Overall
Percentage of the population mainly using each fuel type
100%
75%
50%
25%
0%
1990
2000
2010
2020
2030
Fuel Type
Total Clean
Total Polluting

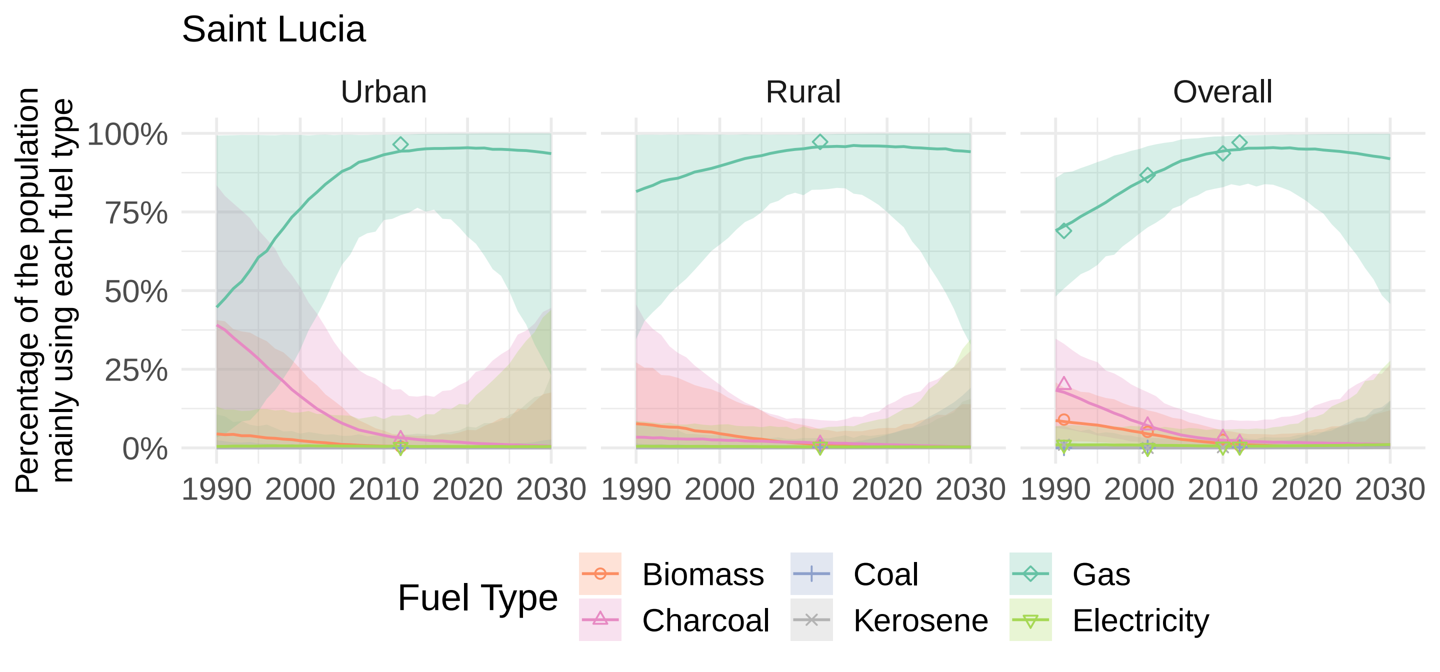

Saint Lucia
Urban
Rural
Overall
Percentage of the population mainly using each fuel type
100%
75%
50%
25%
0%
1990
2000
2010
2020
2030
Fuel Type
Biomass
Charcoal
Coal
Kerosene
Gas
Electricity

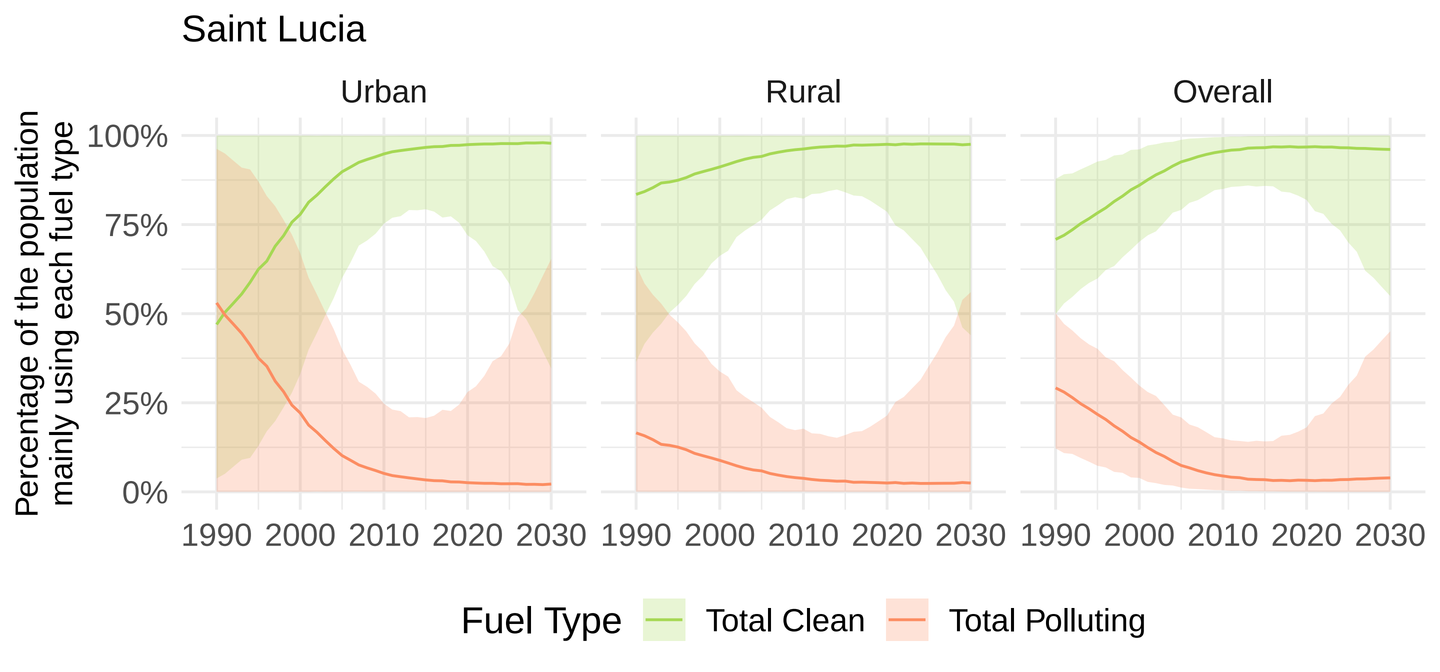
Saint Lucia
Urban
Rural
Overall
Percentage of the population mainly using each fuel type
100%
75%
50%
25%
0%
1990
2000
2010
2020
2030
Fuel Type
Total Clean
Total Polluting

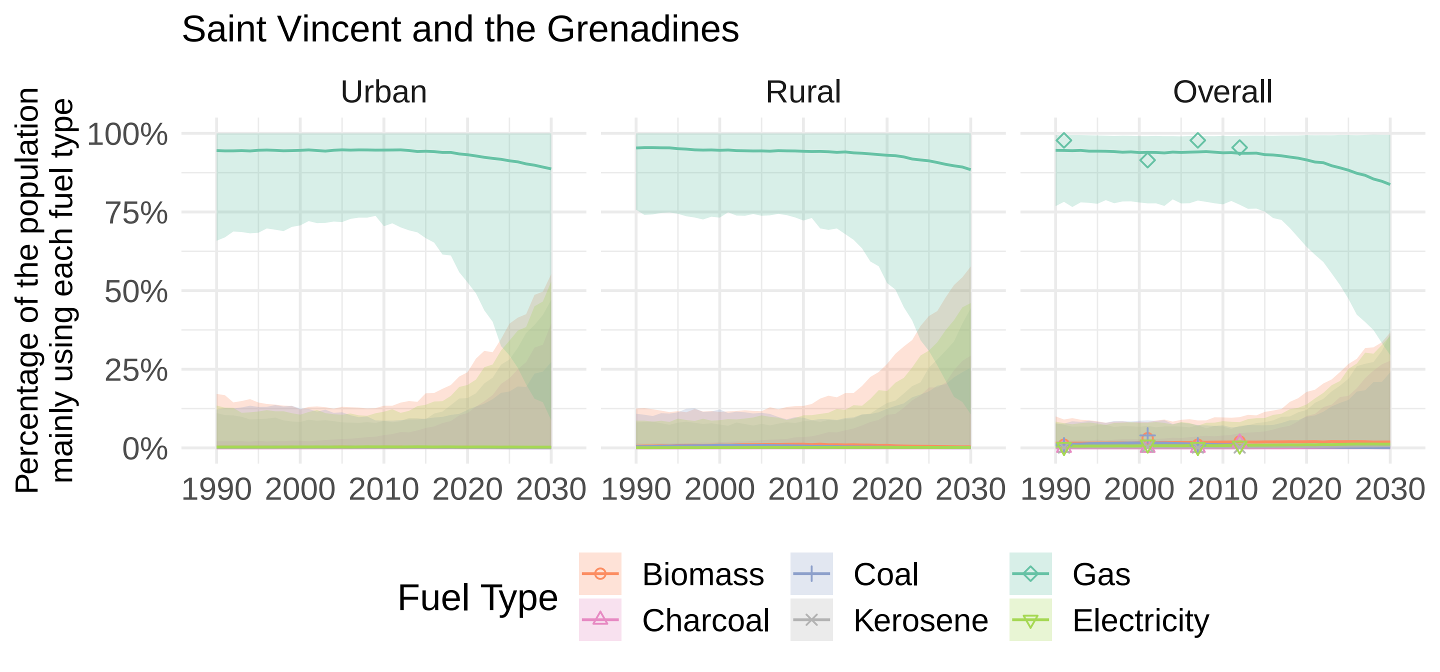
Saint Vincent and the Grenadines
Urban
Rural
Overall
Percentage of the population mainly using each fuel type
100%
75%
50%
25%
0%
1990
2000
2010
2020
2030
Fuel Type
Biomass
Charcoal
Coal
Kerosene
Gas
Electricity

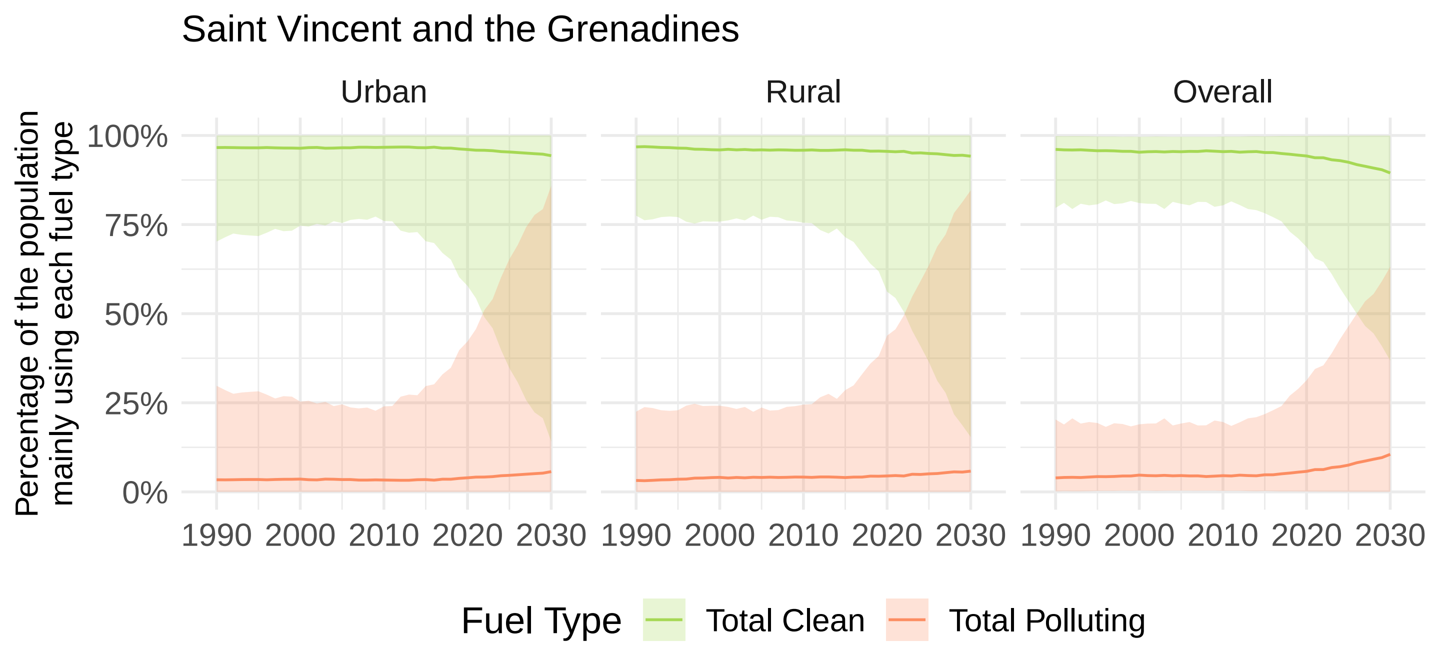
Saint Vincent and the Grenadines
Urban
Rural
Overall
Percentage of the population mainly using each fuel type
100%
75%
50%
25%
0%
1990
2000
2010
2020
2030
Fuel Type
Total Clean
Total Polluting

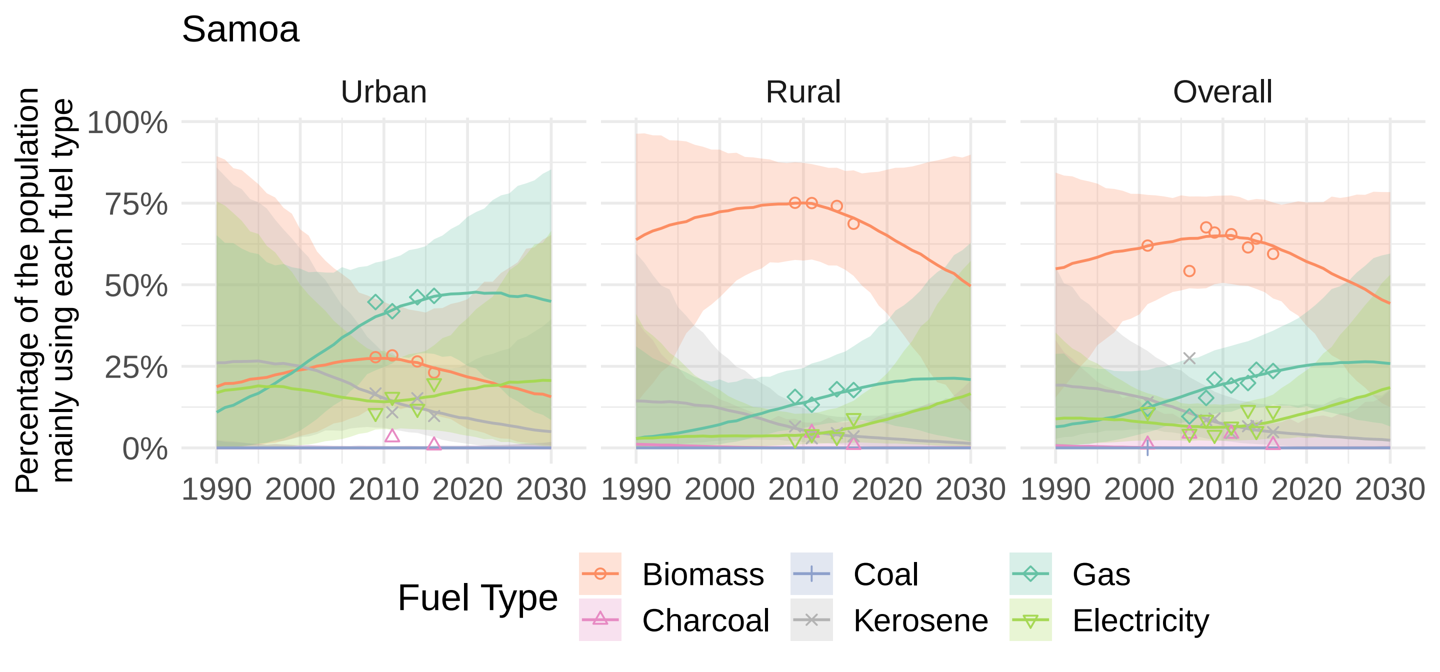

Samoa
Urban
Rural
Overall
Percentage of the population mainly using each fuel type
100%
75%
50%
25%
0%
1990
2000
2010
2020
2030
Fuel Type
Biomass
Charcoal
Coal
Kerosene
Gas
Electricity

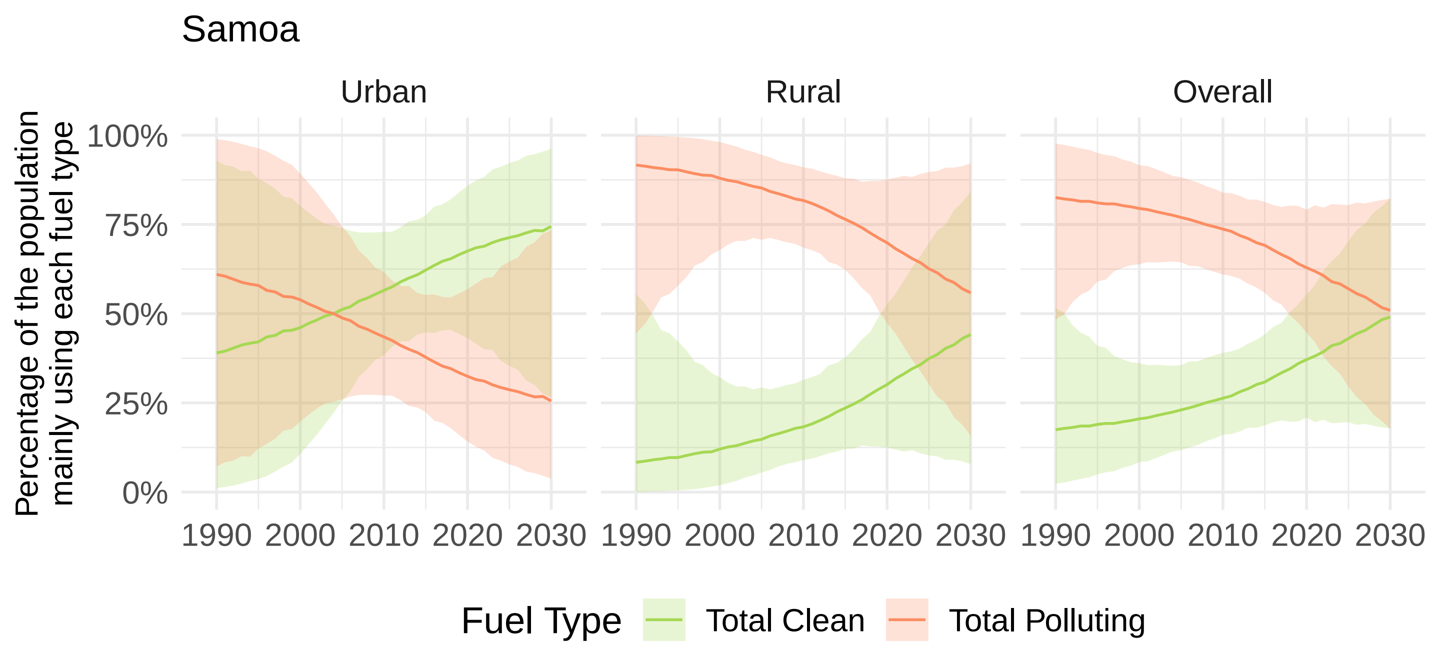

Samoa
Urban
Rural
Overall
Percentage of the population mainly using each fuel type
100%
75%
50%
25%
0%
1990
2000
2010
2020
2030
Fuel Type
Total Clean
Total Polluting

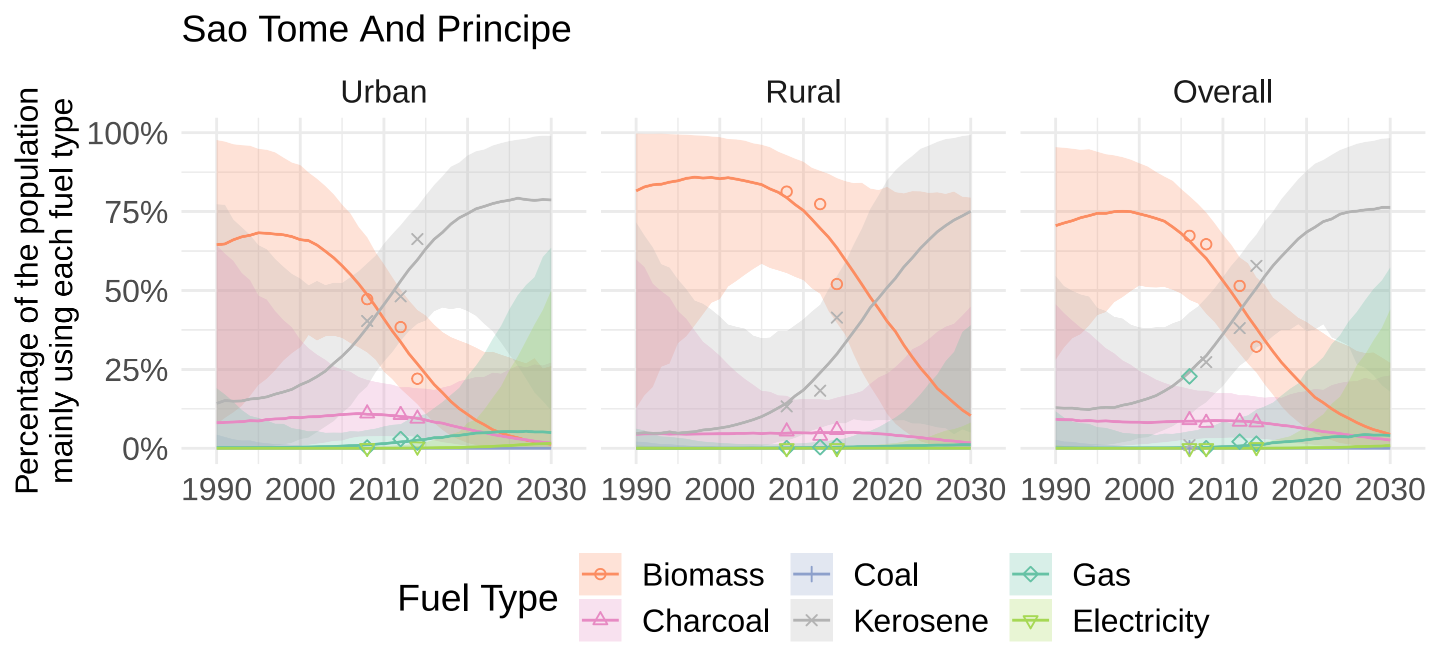
Sao Tome And Principe
Urban
Rural
Overall
Percentage of the population mainly using each fuel type
100%
75%
50%
25%
0%
1990
2000
2010
2020
2030
Fuel Type
Biomass
Charcoal
Coal
Kerosene
Gas
Electricity

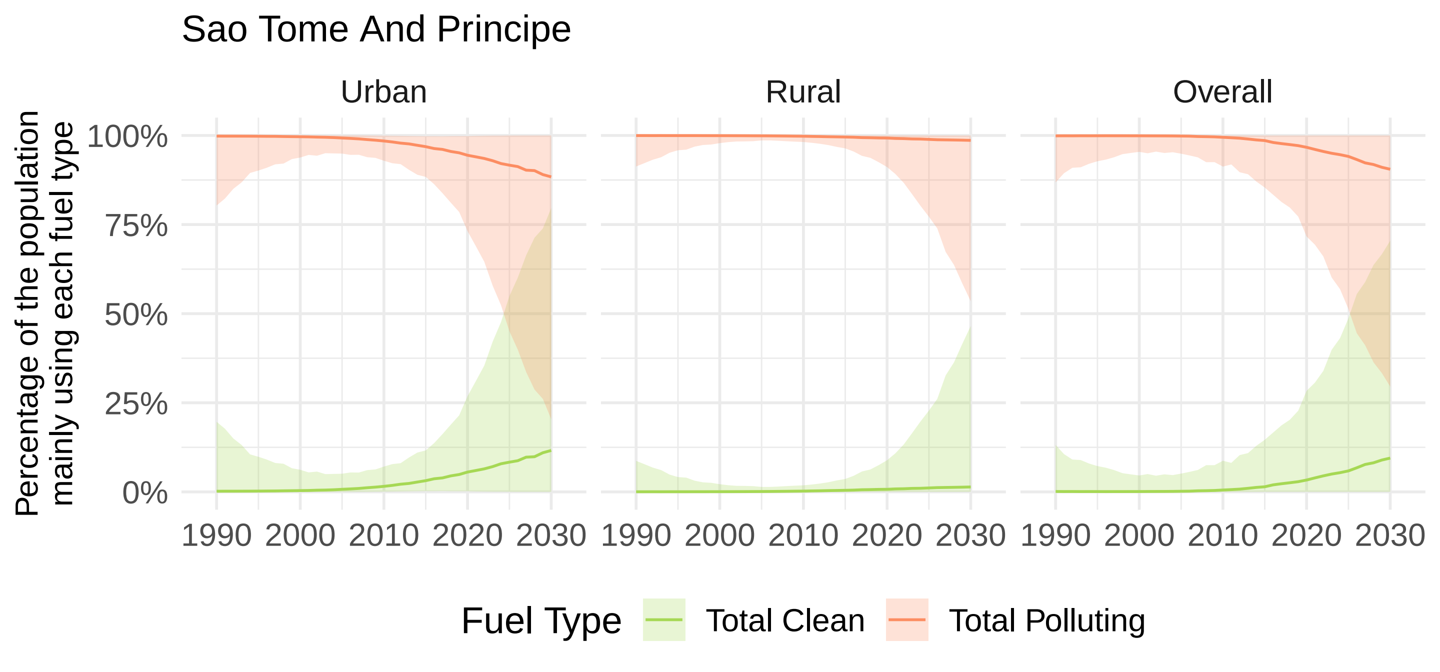
Sao Tome And Principe
Urban
Rural
Overall
Percentage of the population mainly using each fuel type
100%
75%
50%
25%
0%
1990
2000
2010
2020
2030
Fuel Type
Total Clean
Total Polluting

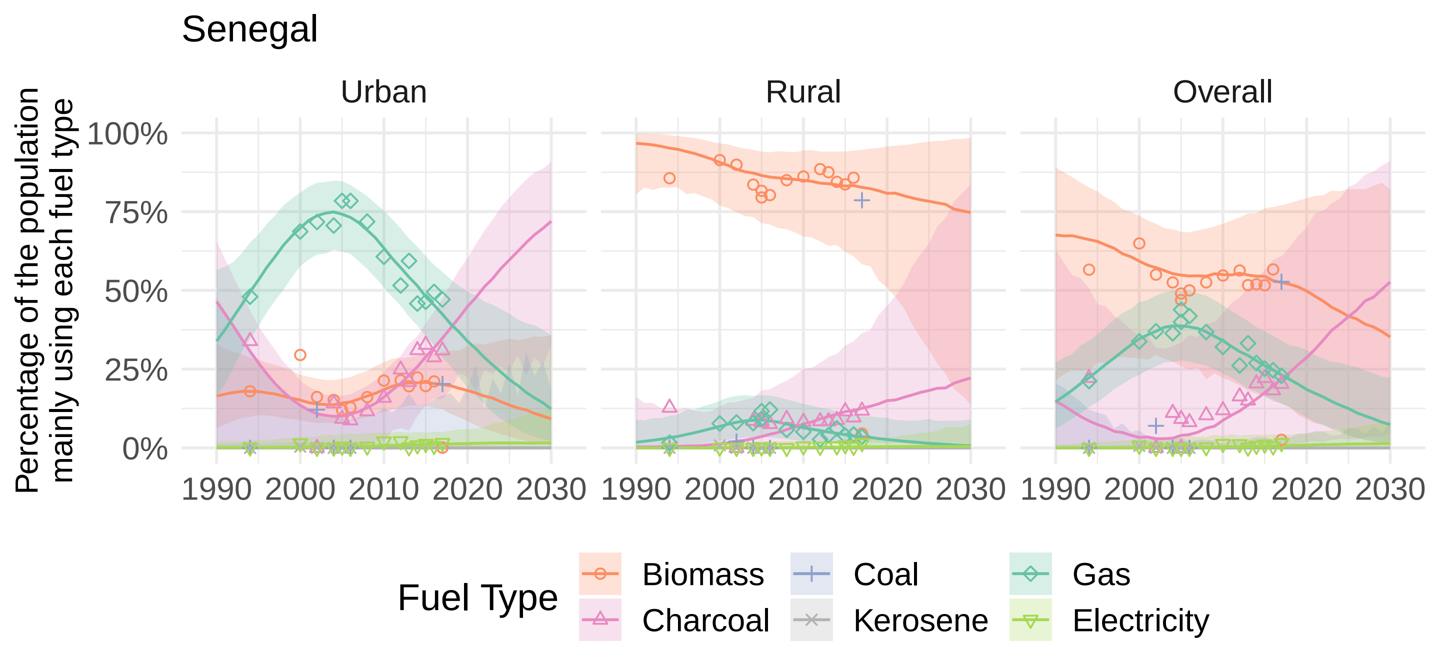

Senegal
Urban
Rural
Overall
Percentage of the population mainly using each fuel type
100%
75%
50%
25%
0%
1990
2000
2010
2020
2030
Fuel Type
Biomass
Charcoal
Coal
Kerosene
Gas
Electricity

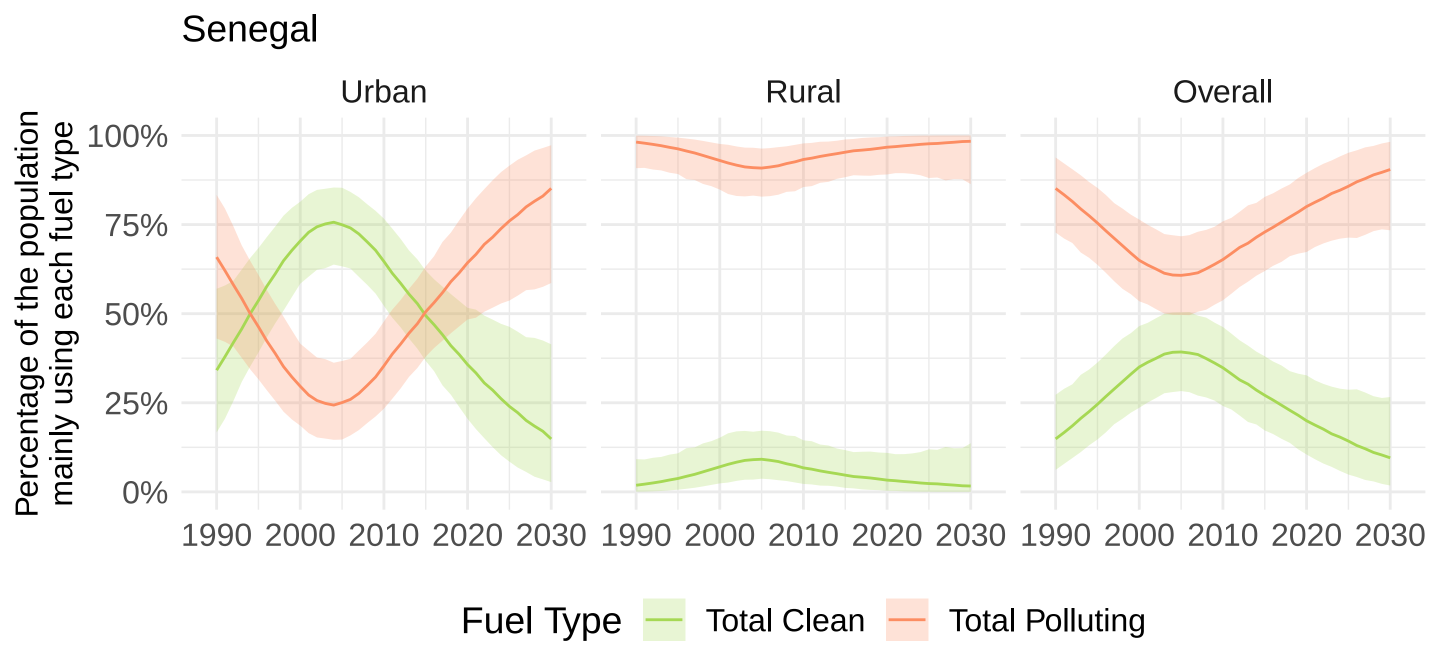

Senegal
Urban
Rural
Overall
Percentage of the population mainly using each fuel type
100%
75%
50%
25%
0%
1990
2000
2010
2020
2030
Fuel Type
Total Clean
Total Polluting

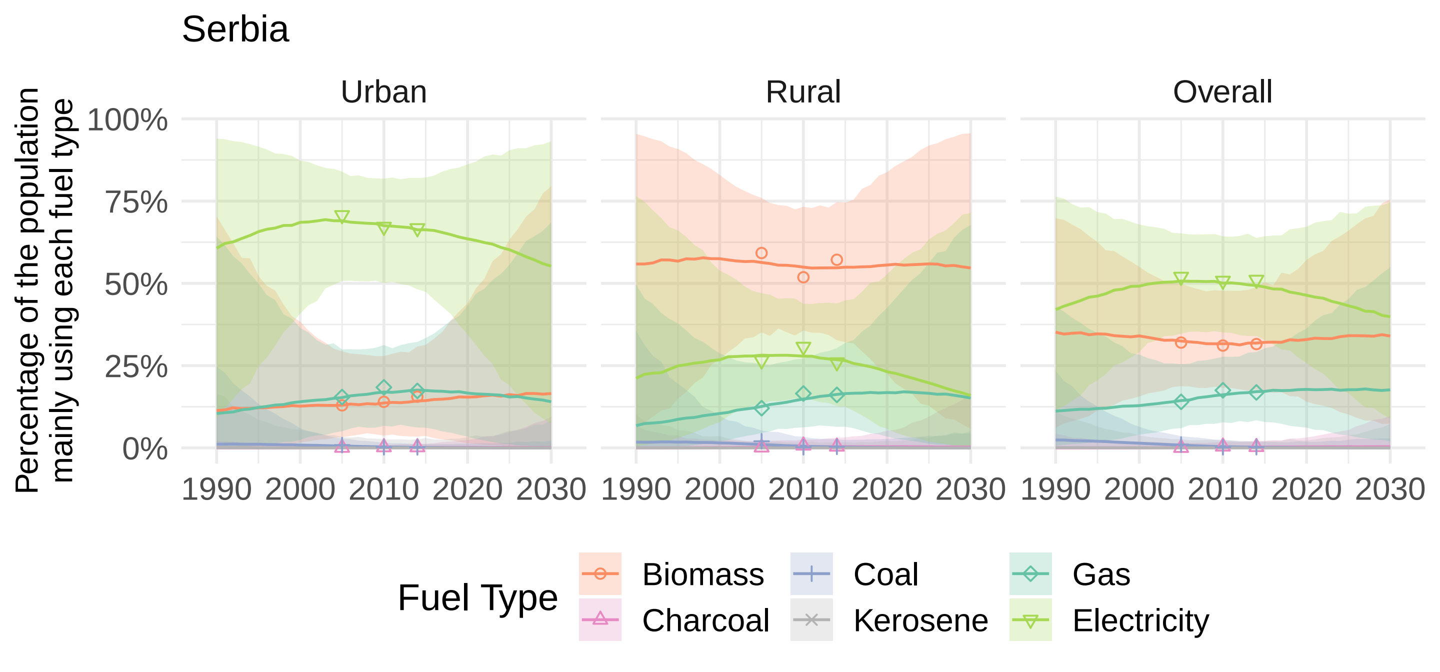

Serbia
Urban
Rural
Overall
Percentage of the population
mainly using each fuel type
100%
75%
50%
25%
0%
1990
2000
2010
2020
2030
Fuel Type
Biomass
Charcoal
Coal
Kerosene
Gas
Electricity

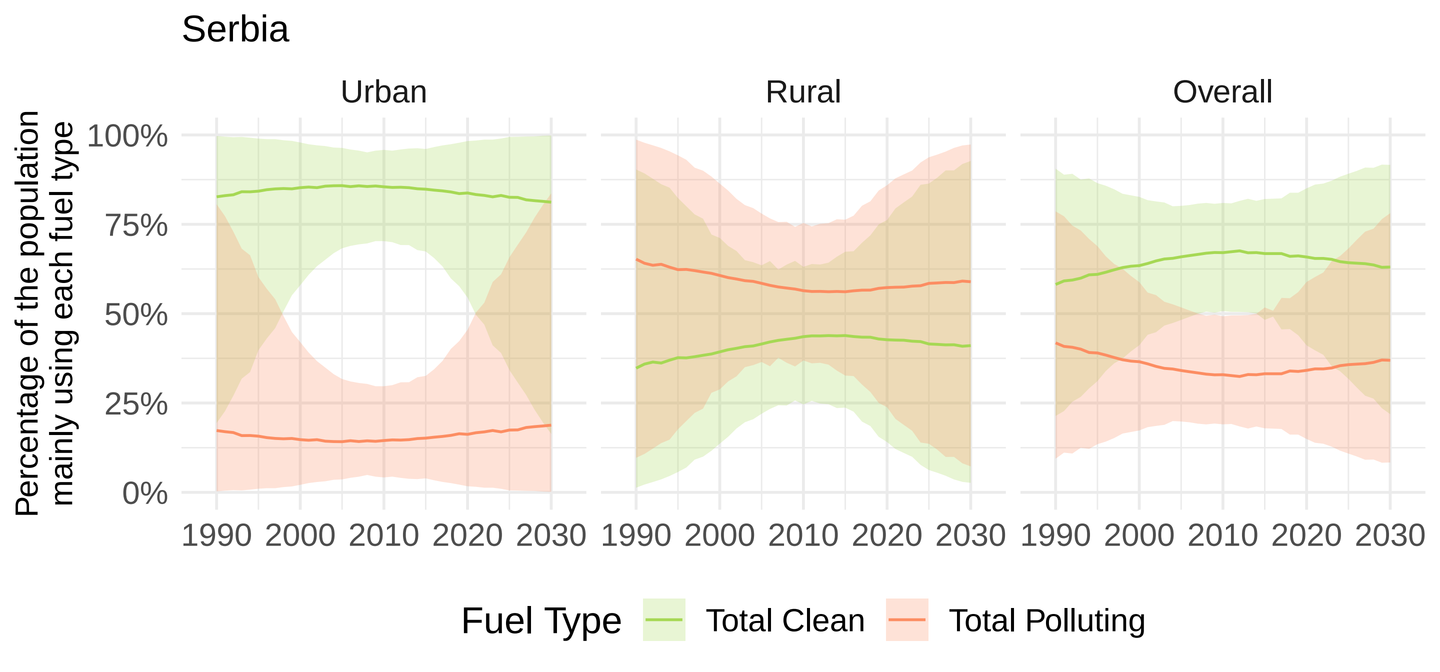
Serbia
Urban
Rural
Overall
Percentage of the population mainly using each fuel type
100%
75%
50%
25%
0%
1990
2000
2010
2020
2030
Fuel Type
Total Clean
Total Polluting

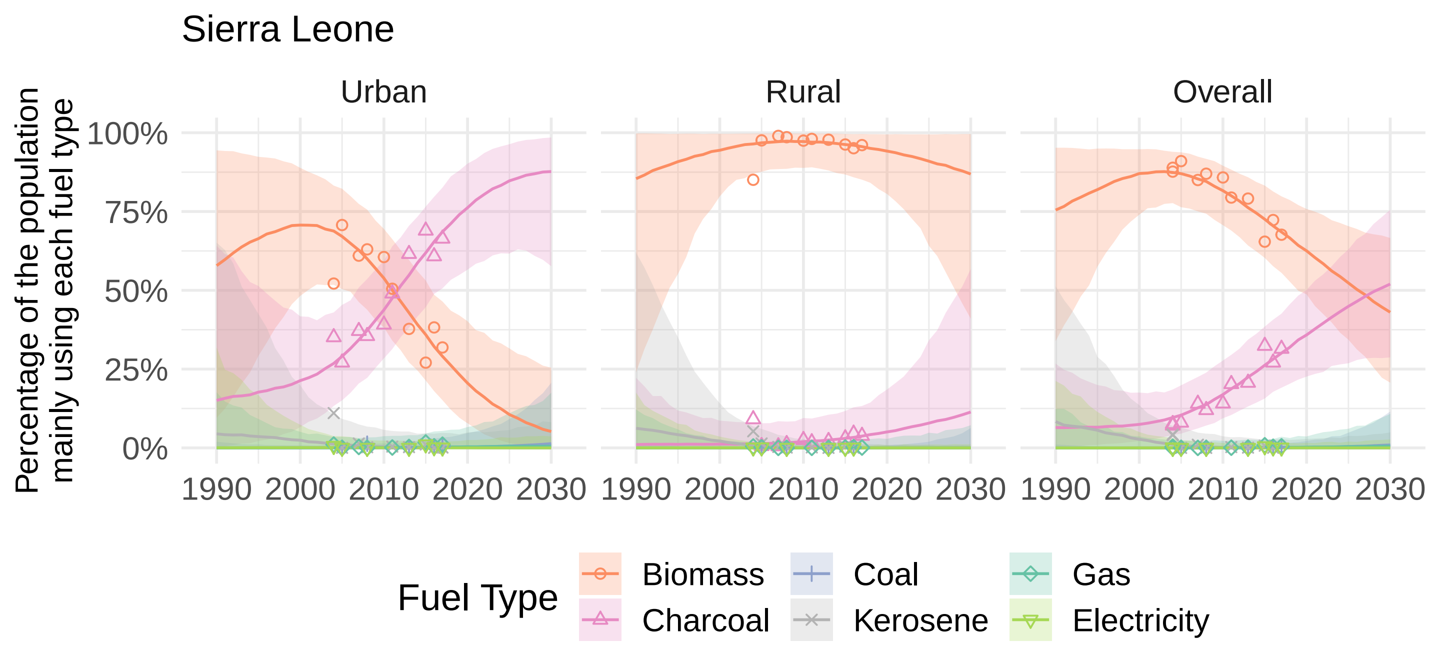
Sierra Leone
Urban
Rural
Overall
Percentage of the population mainly using each fuel type
100%
75%
50%
25%
0%
1990
2000
2010
2020
2030
Fuel Type
Biomass
Charcoal
Coal
Kerosene
Gas
Electricity

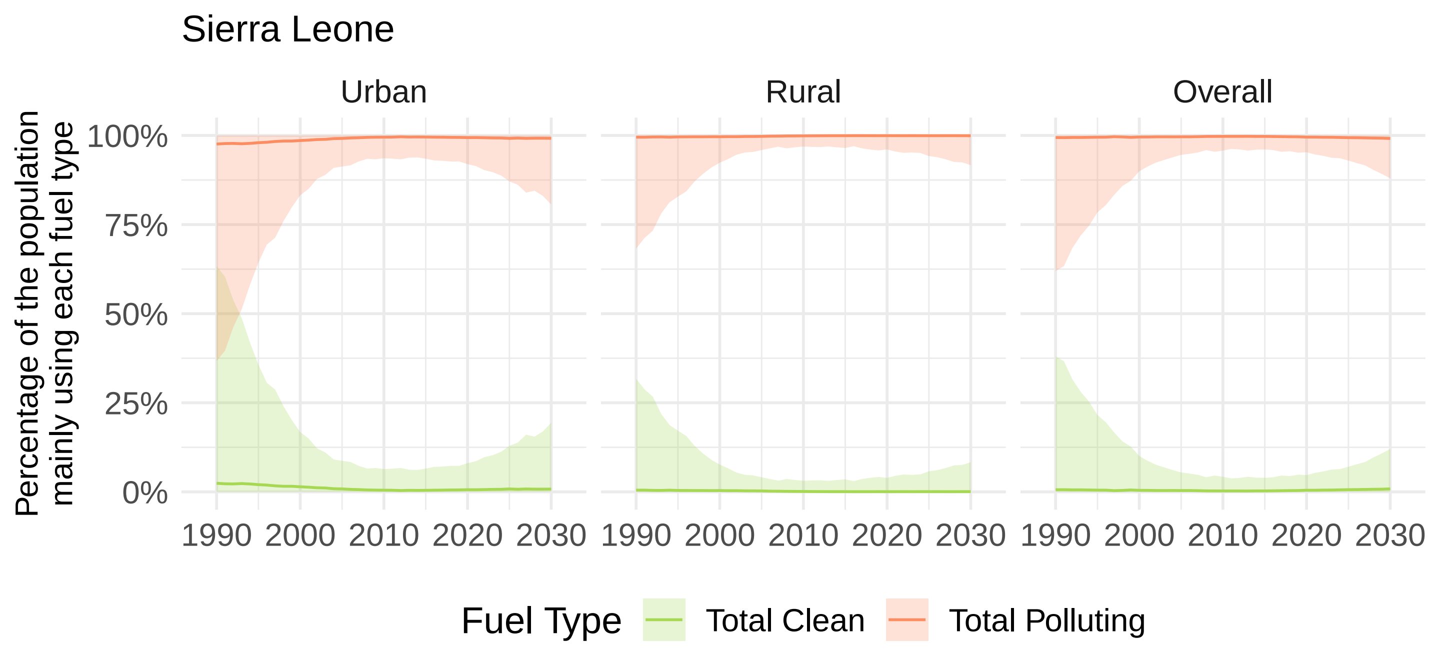

Sierra Leone
Urban
Rural
Overall
Percentage of the population mainly using each fuel type
100%
75%
50%
25%
0%
1990
2000
2010
2020
2030
Fuel Type
Total Clean
Total Polluting

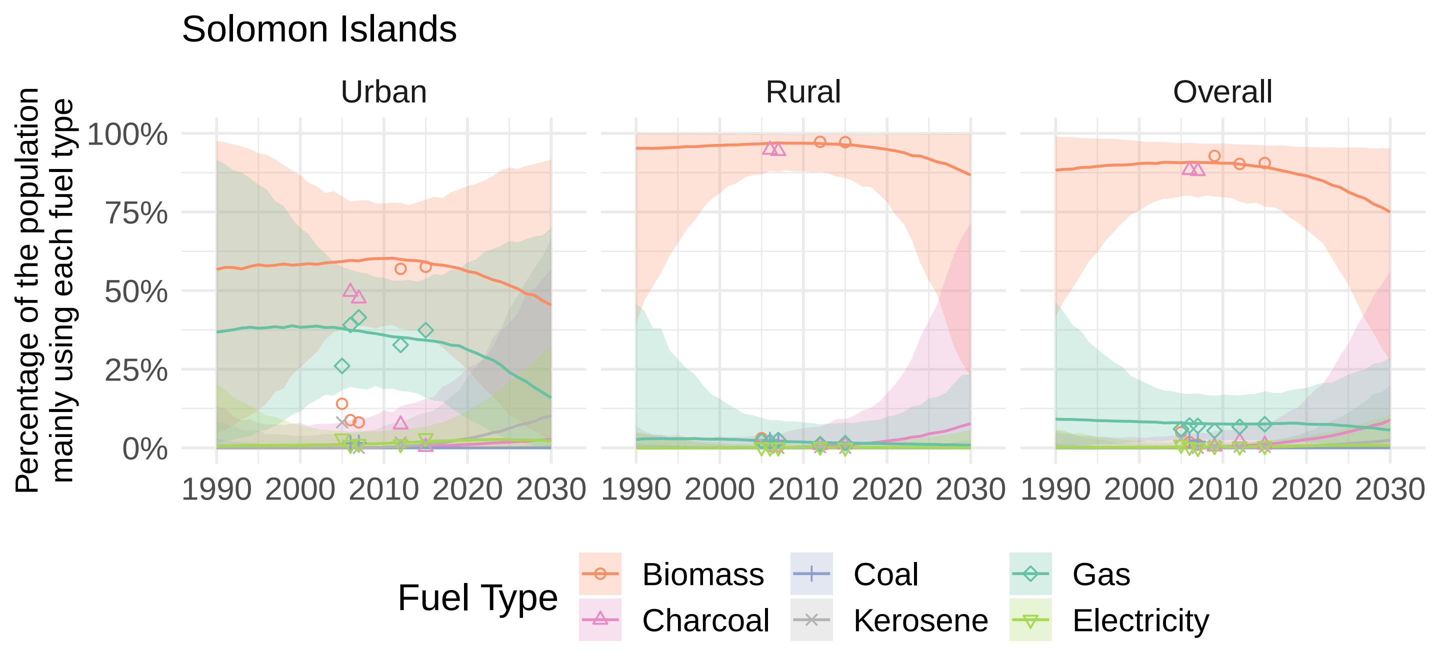
Solomon Islands
Urban
Rural
Overall
Percentage of the population mainly using each fuel type
100%
75%
50%
25%
0%
1990
2000
2010
2020
2030
Fuel Type
Biomass
Charcoal
Coal
Kerosene
Gas
Electricity

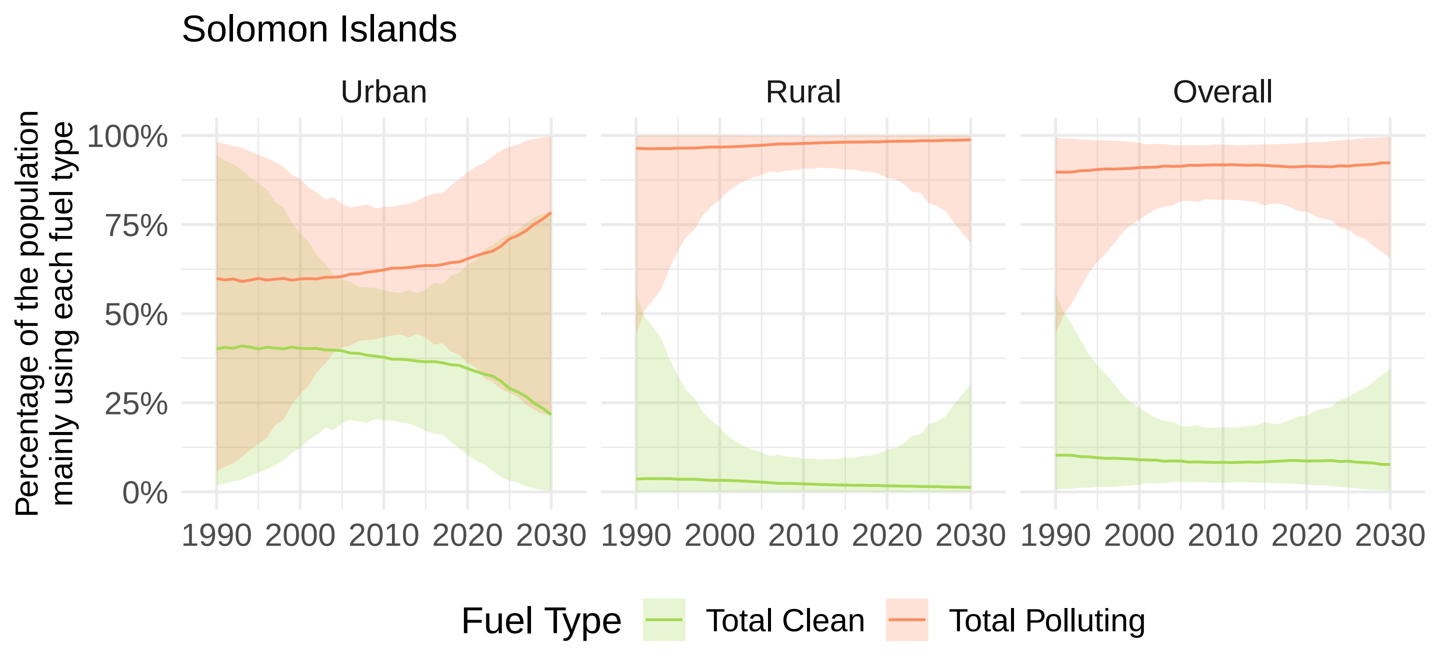
Solomon Islands
Urban
Rural
Overall
Percentage of the population mainly using each fuel type
100%
75%
50%
25%
0%
1990
2000
2010
2020
2030
Fuel Type
Total Clean
Total Polluting

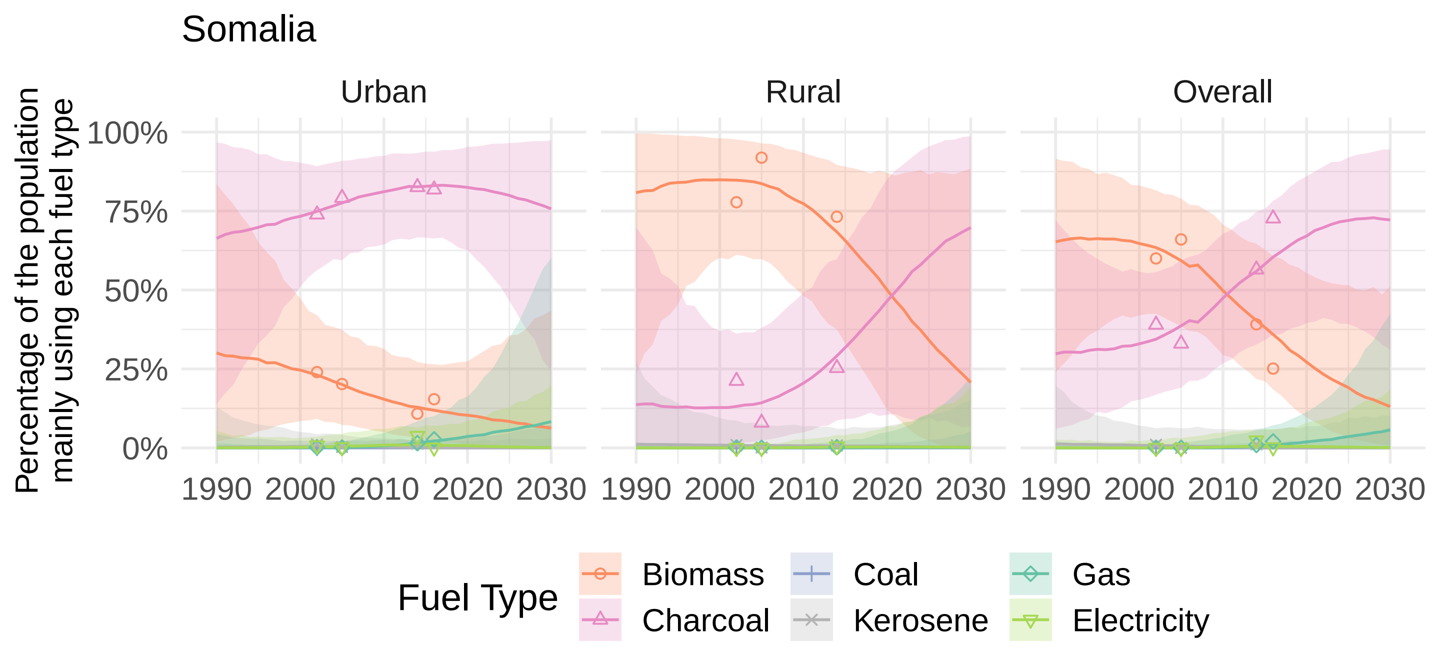
Somalia
Urban
Rural
Overall
Percentage of the population mainly using each fuel type
100%
75%
50%
25%
0%
1990
2000
2010
2020
2030
Fuel Type
Biomass
Charcoal
Coal
Kerosene
Gas
Electricity

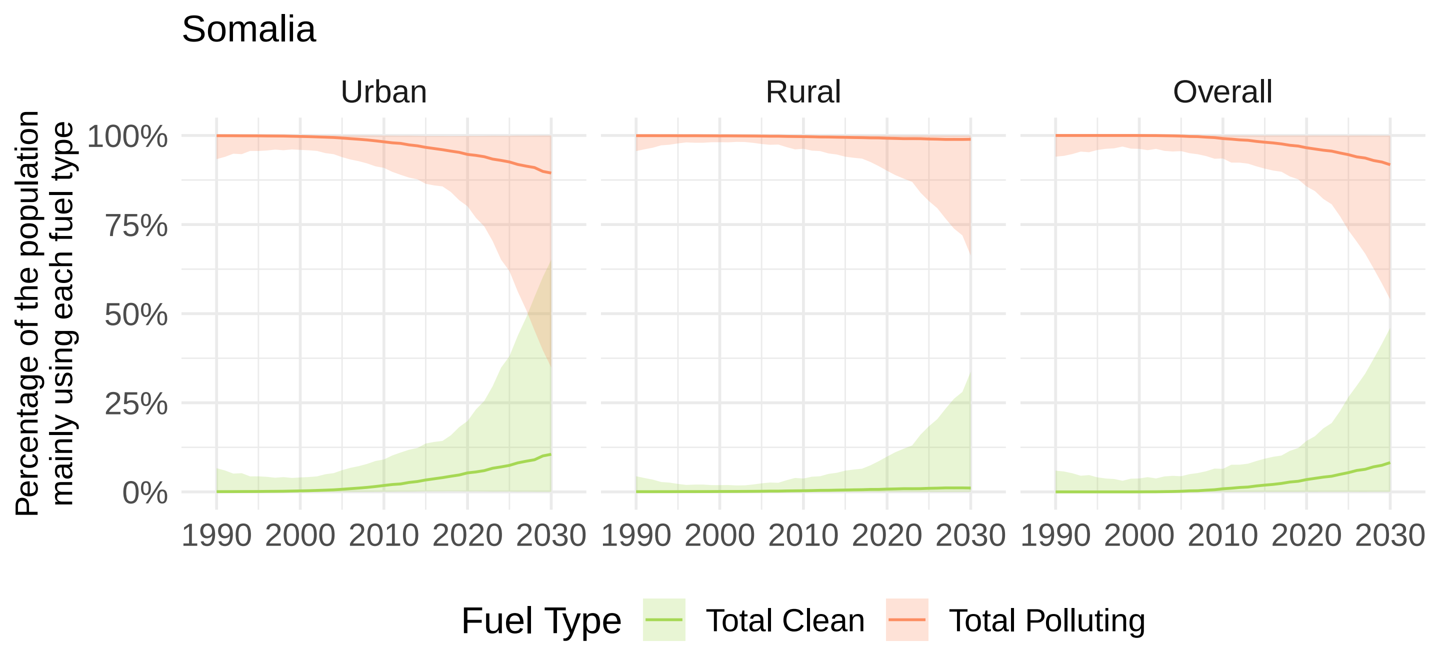

Somalia
Urban
Rural
Overall
Percentage of the population mainly using each fuel type
100%
75%
50%
25%
0%
1990
2000
2010
2020
2030
Fuel Type
Total Clean
Total Polluting

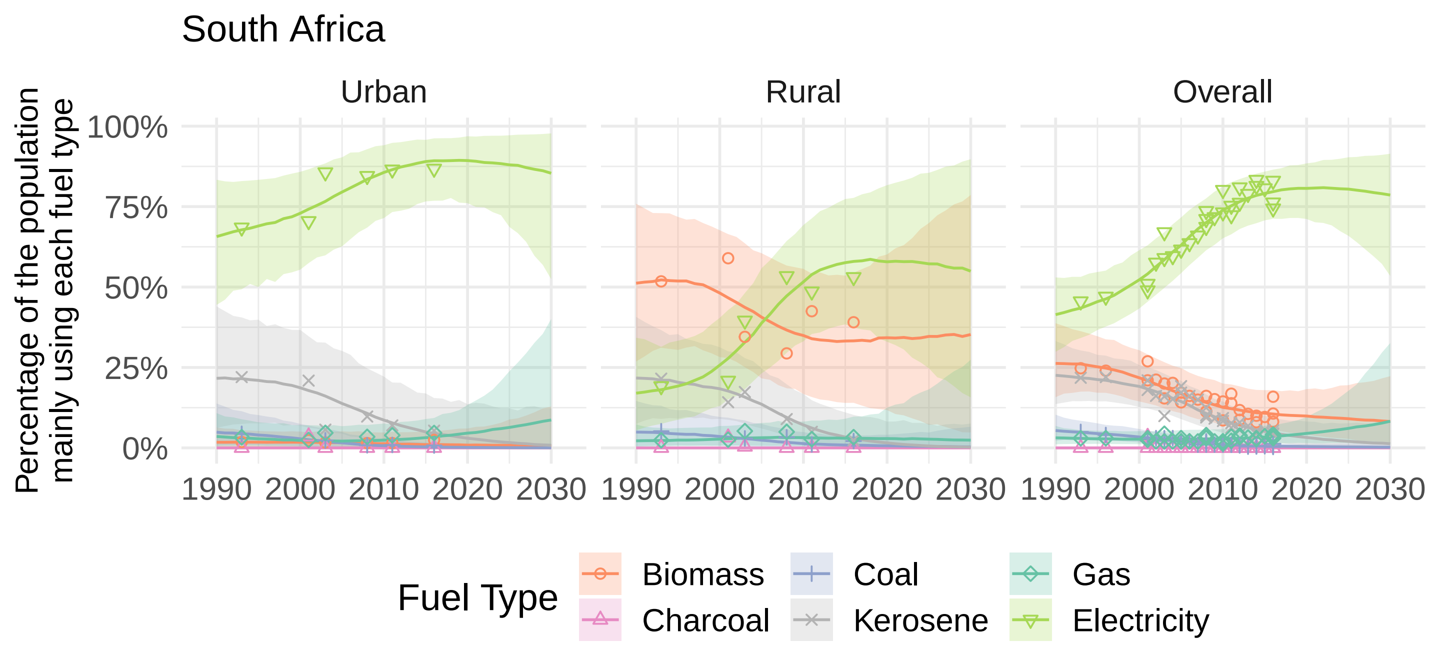
South Africa
Urban
Rural
Overall
Percentage of the population mainly using each fuel type
100%
75%
50%
25%
0%
1990
2000
2010
2020
2030
Fuel Type
Biomass
Charcoal
Coal
Kerosene
Gas
Electricity

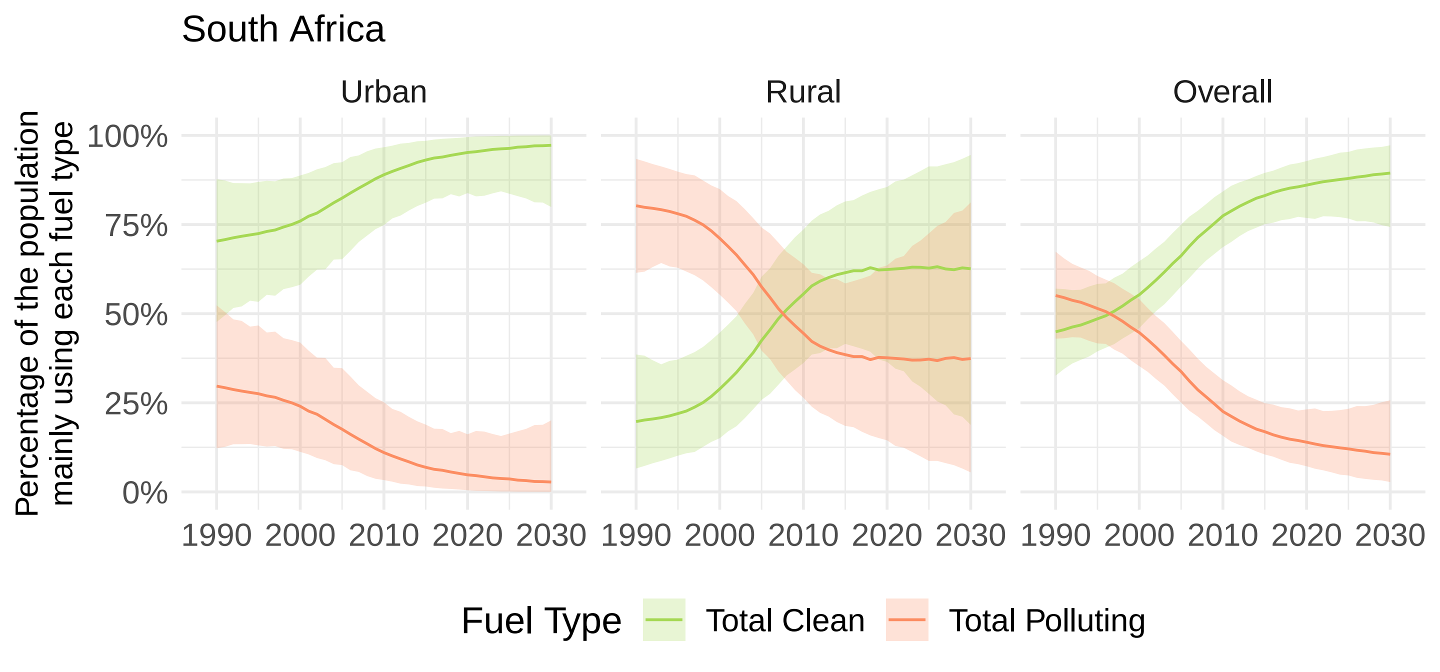
South Africa
Urban
Rural
Overall
Percentage of the population mainly using each fuel type
100%
75%
50%
25%
0%
1990
2000
2010
2020
2030
Fuel Type
Total Clean
Total Polluting

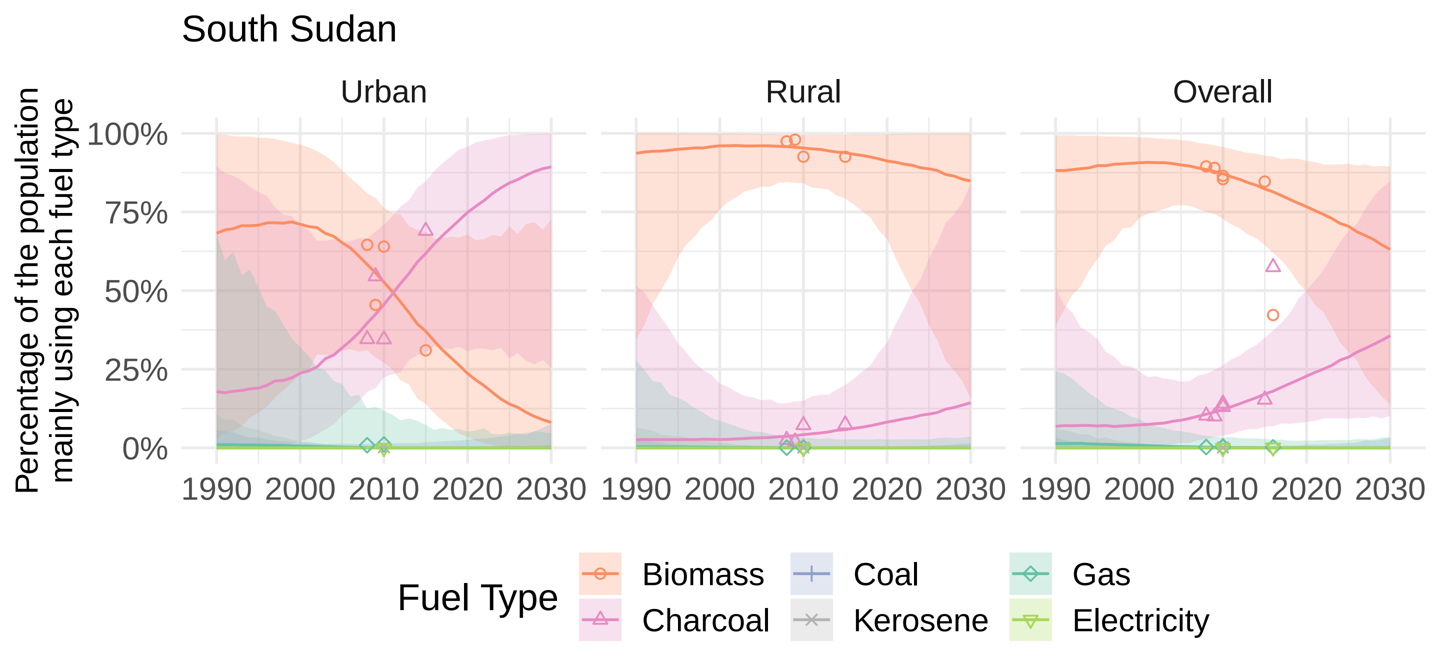
South Sudan
Urban
Rural
Overall
Percentage of the population mainly using each fuel type
100%
75%
50%
25%
0%
1990
2000
2010
2020
2030
Fuel Type
Biomass
Charcoal
Coal
Kerosene
Gas
Electricity

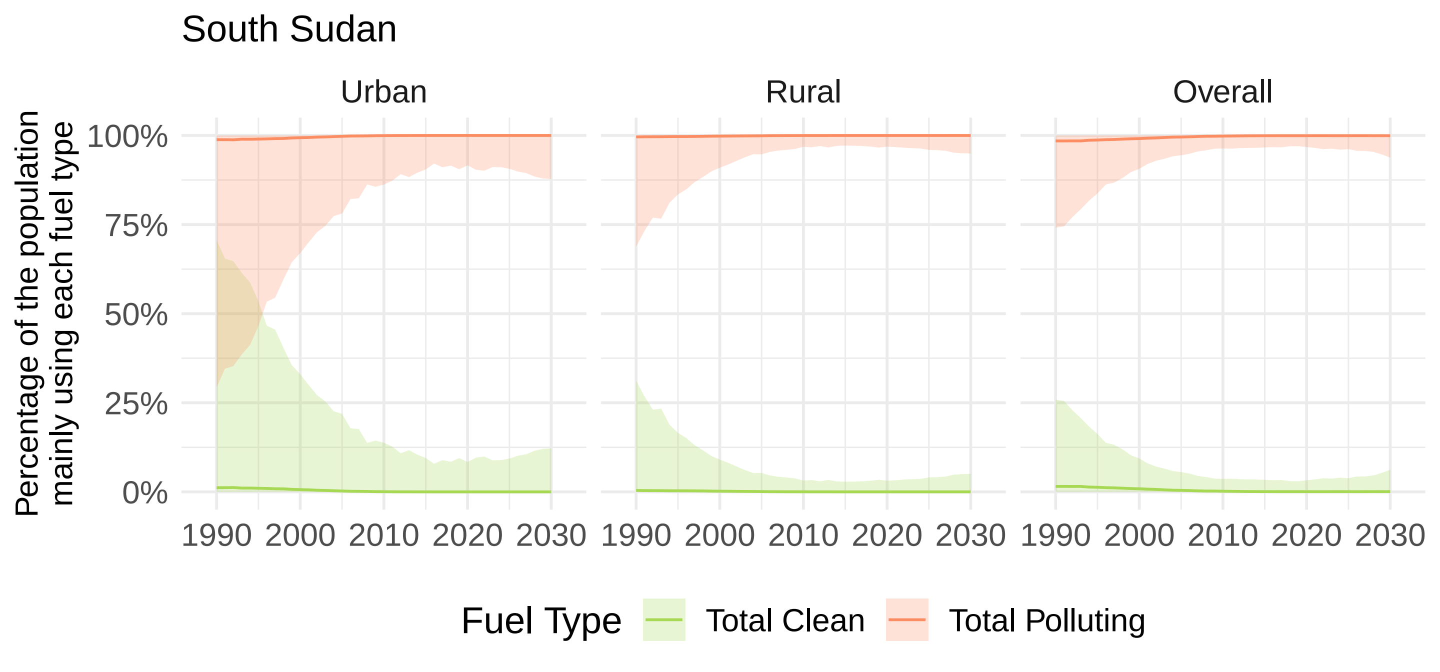
South Sudan
Urban
Rural
Overall
Percentage of the population mainly using each fuel type
100%
75%
50%
25%
0%
1990
2000
2010
2020
2030
Fuel Type
Total Clean
Total Polluting

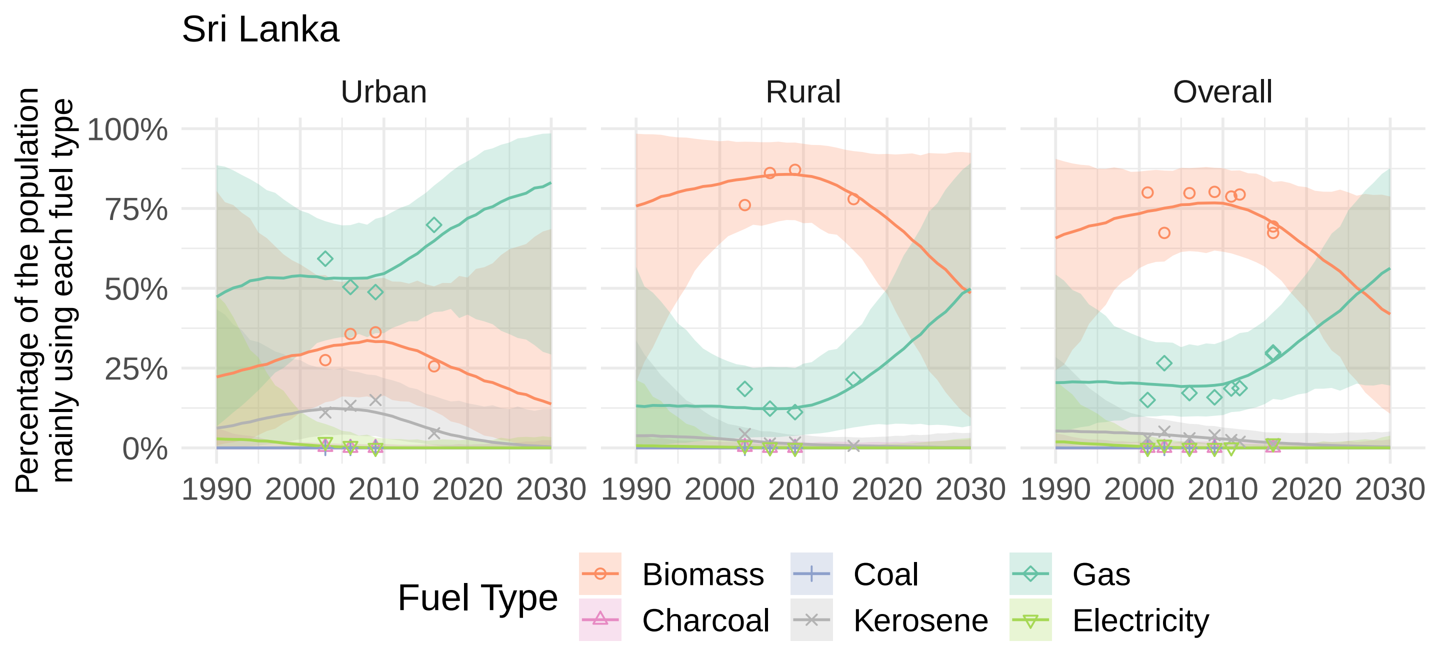
Sri Lanka
Urban
Rural
Overall
Percentage of the population mainly using each fuel type
100%
75%
50%
25%
0%
1990
2000
2010
2020
2030
Fuel Type
Biomass
Charcoal
Coal
Kerosene
Gas
Electricity

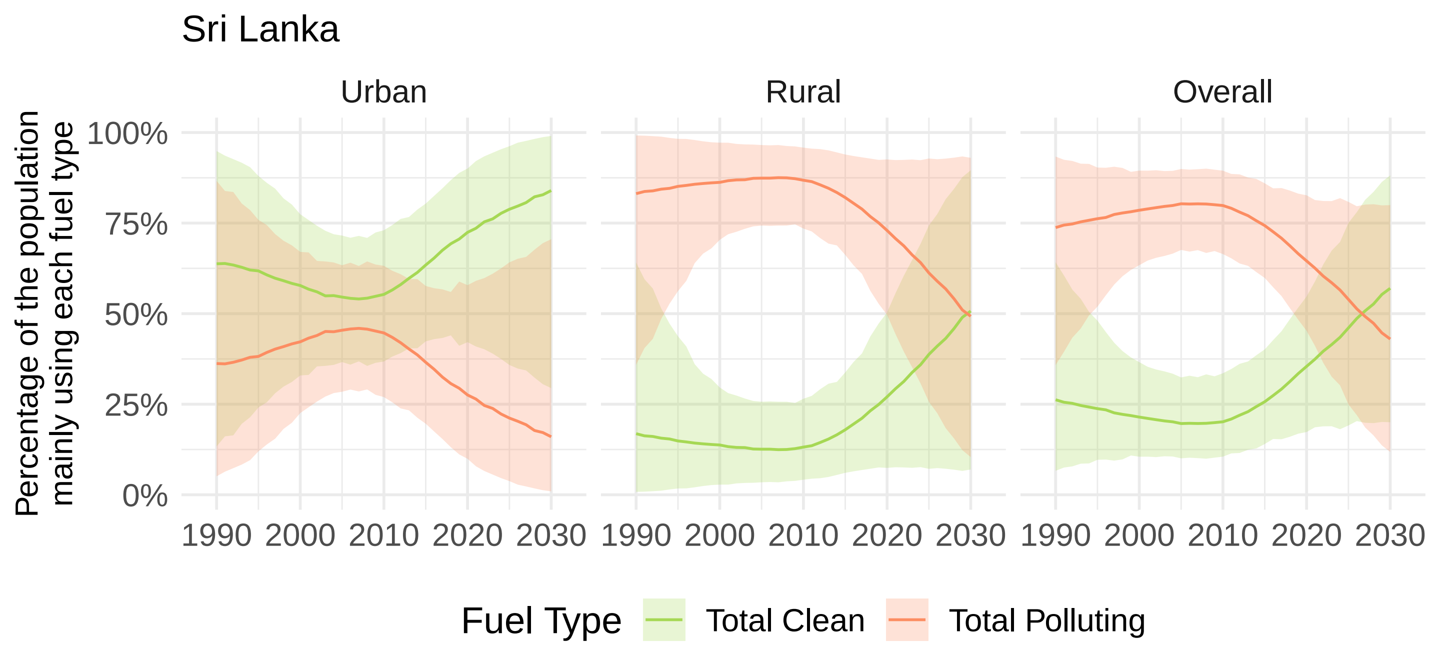
Sri Lanka
Urban
Rural
Overall
Percentage of the population
mainly using each fuel type
100%
75%
50%
25%
0%
1990
2000
2010
2020
2030
Fuel Type
Total Clean
Total Polluting

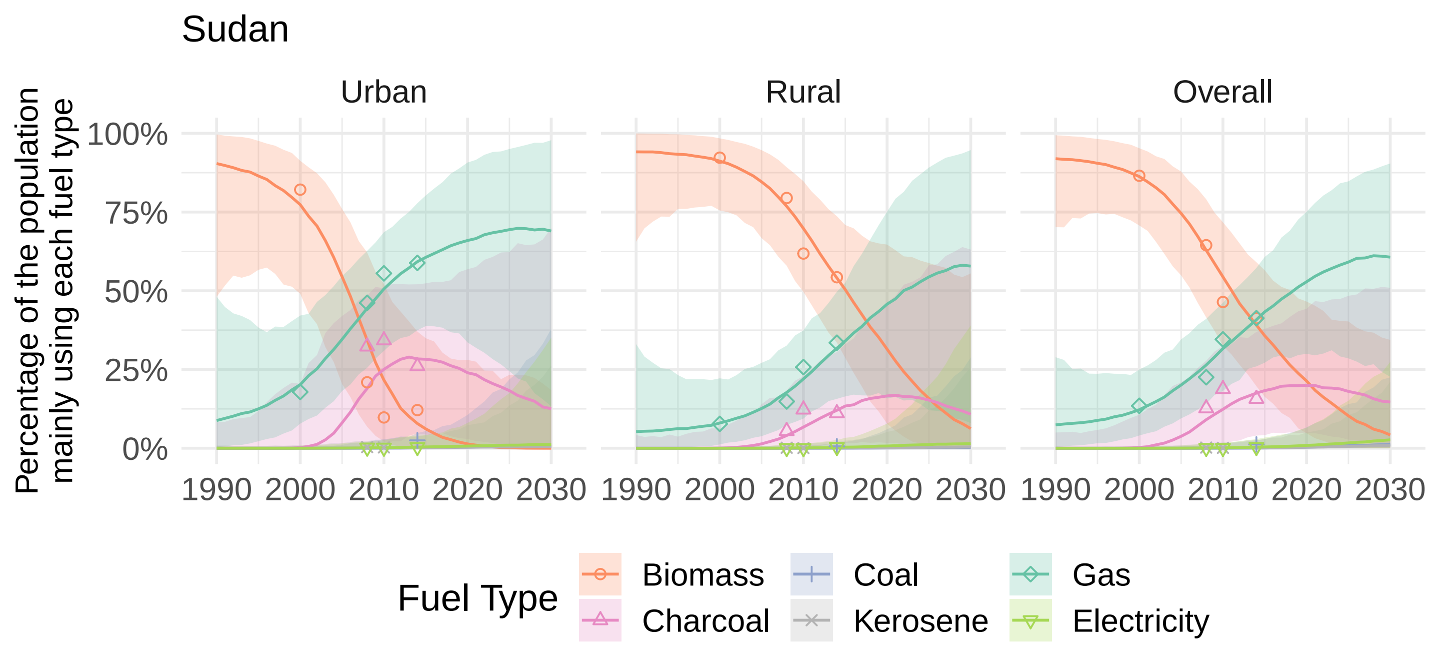

Sudan
Urban
Rural
Overall
Percentage of the population mainly using each fuel type
100%
75%
50%
25%
0%
1990
2000
2010
2020
2030
Fuel Type
Biomass
Charcoal
Coal
Kerosene
Gas
Electricity

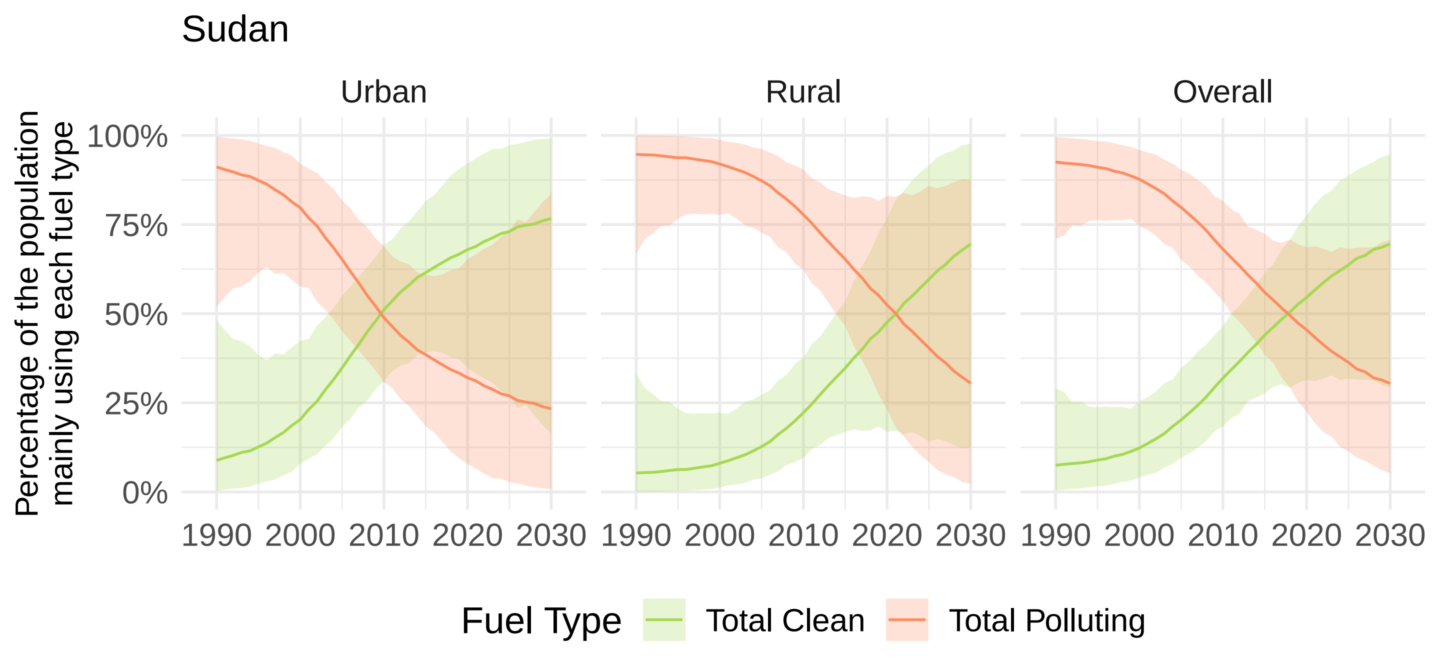

Sudan
Urban
Rural
Overall
Percentage of the population mainly using each fuel type
100%
75%
50%
25%
0%
1990
2000
2010
2020
2030
Fuel Type
Total Clean
Total Polluting

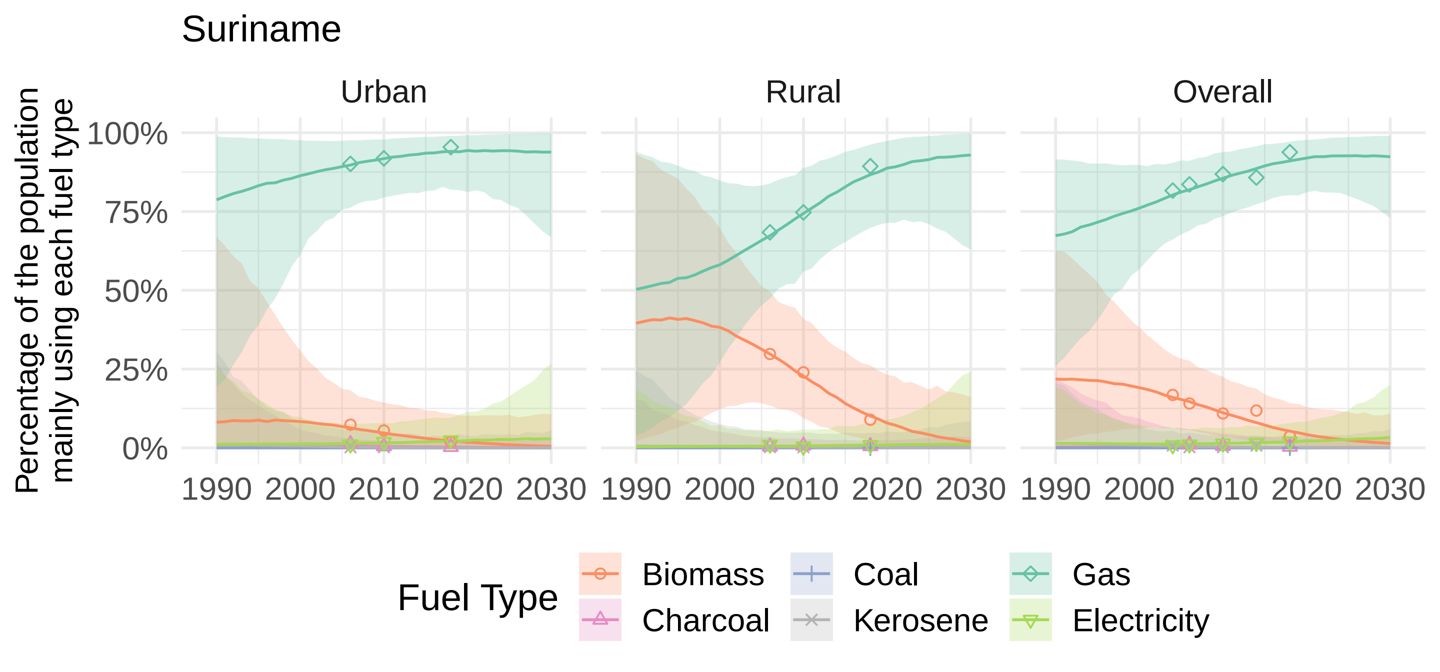

Suriname
Urban
Rural
Overall
Percentage of the population mainly using each fuel type
100%
75%
50%
25%
0%
1990
2000
2010
2020
2030
Fuel Type
Biomass
Charcoal
Coal
Kerosene
Gas
Electricity

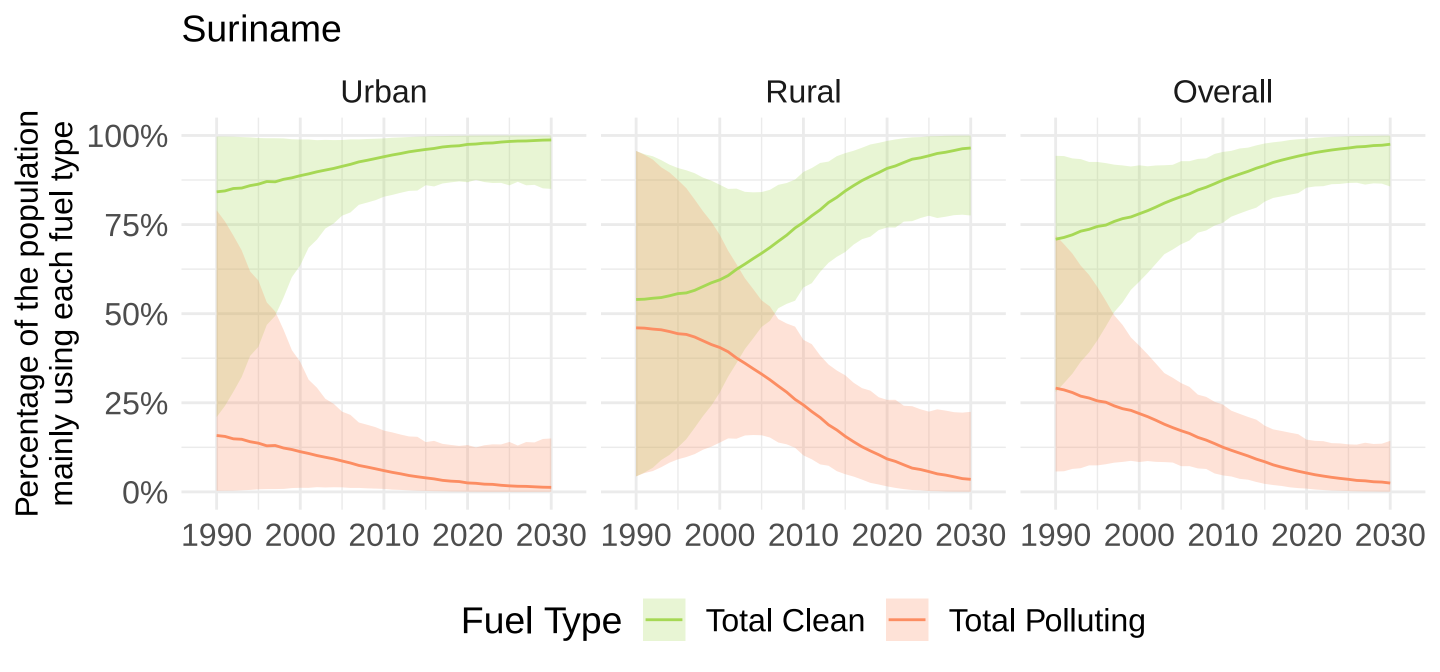
Suriname
Urban
Rural
Overall
Percentage of the population mainly using each fuel type
100%
75%
50%
25%
0%
1990
2000
2010
2020
2030
Fuel Type
Total Clean
Total Polluting

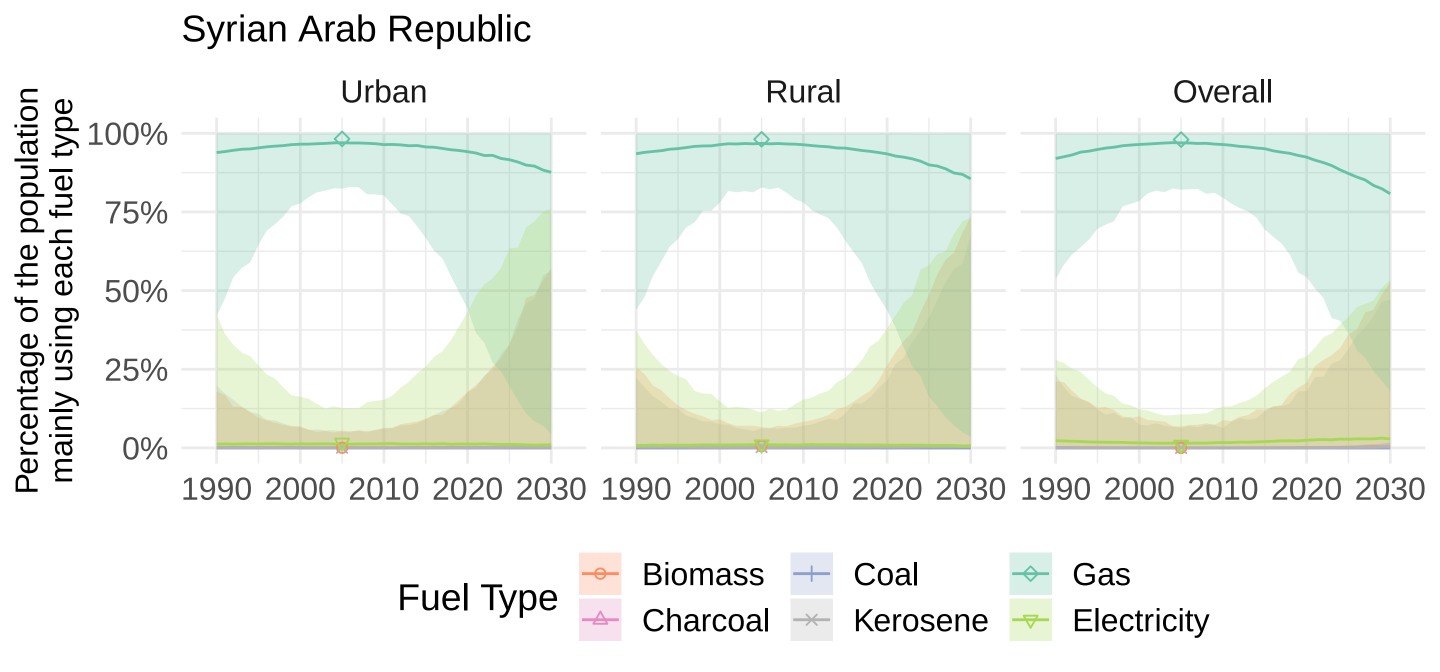
Syrian Arab Republic
Urban
Rural
Overall
Percentage of the population mainly using each fuel type
100%
75%
50%
25%
0%
1990
2000
2010
2020
2030
Fuel Type
Biomass
Charcoal
Coal
Kerosene
Gas
Electricity

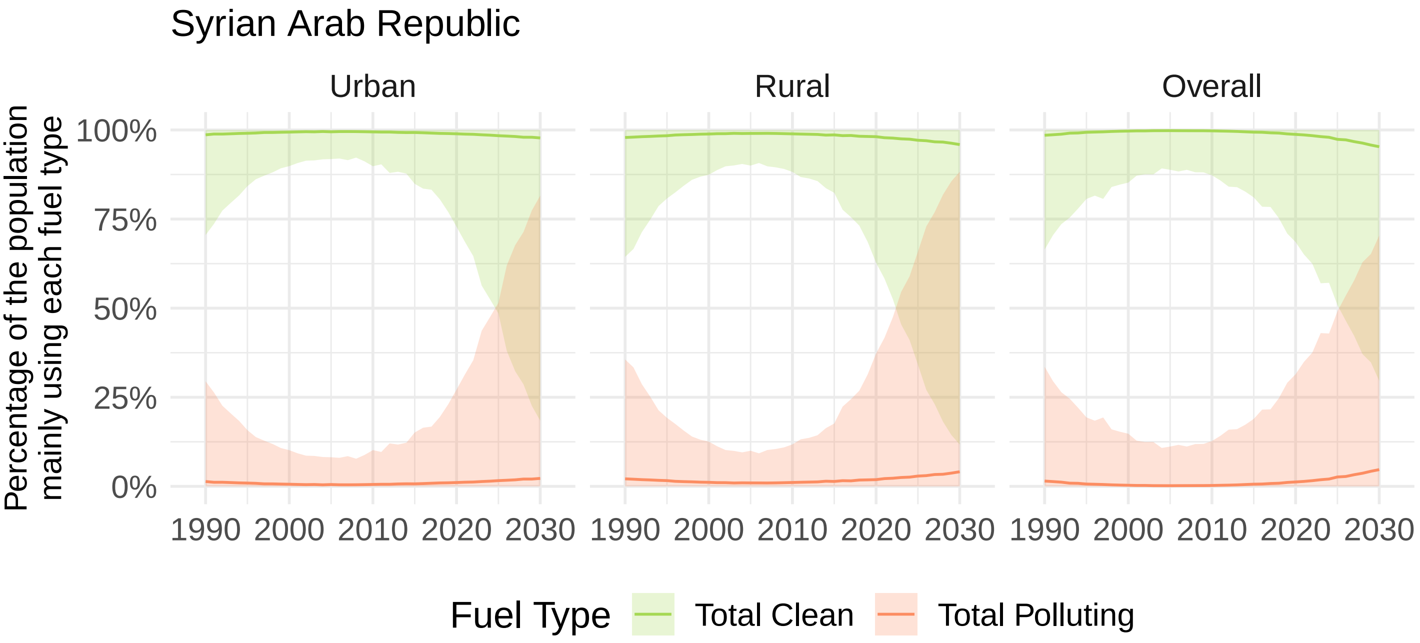

Syrian Arab Republic
Urban
Rural
Overall
Percentage of the population mainly using each fuel type
100%
75%
50%
25%
0%
1990
2000
2010
2020
2030
Fuel Type
Total Clean
Total Polluting

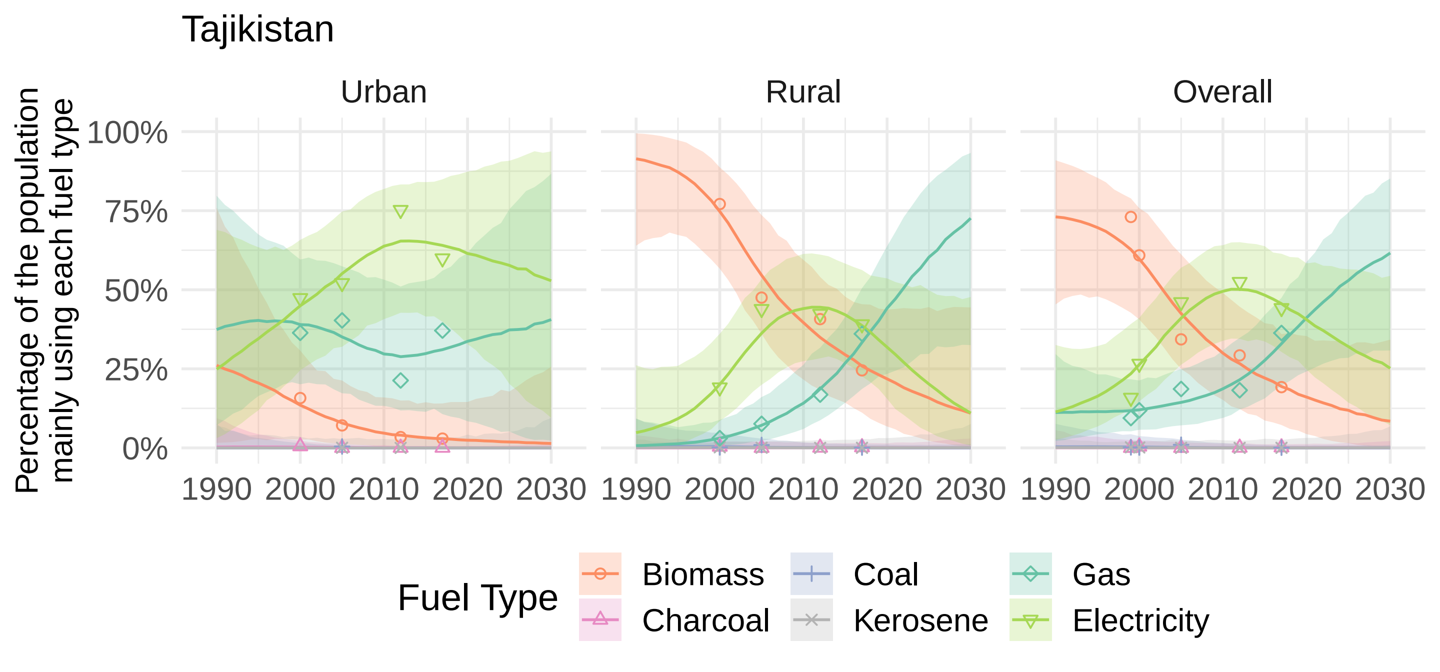

Tajikistan
Urban
Rural
Overall
Percentage of the population mainly using each fuel type
100%
75%
50%
25%
0%
1990
2000
2010
2020
2030
Fuel Type
Biomass
Charcoal
Coal
Kerosene
Gas
Electricity

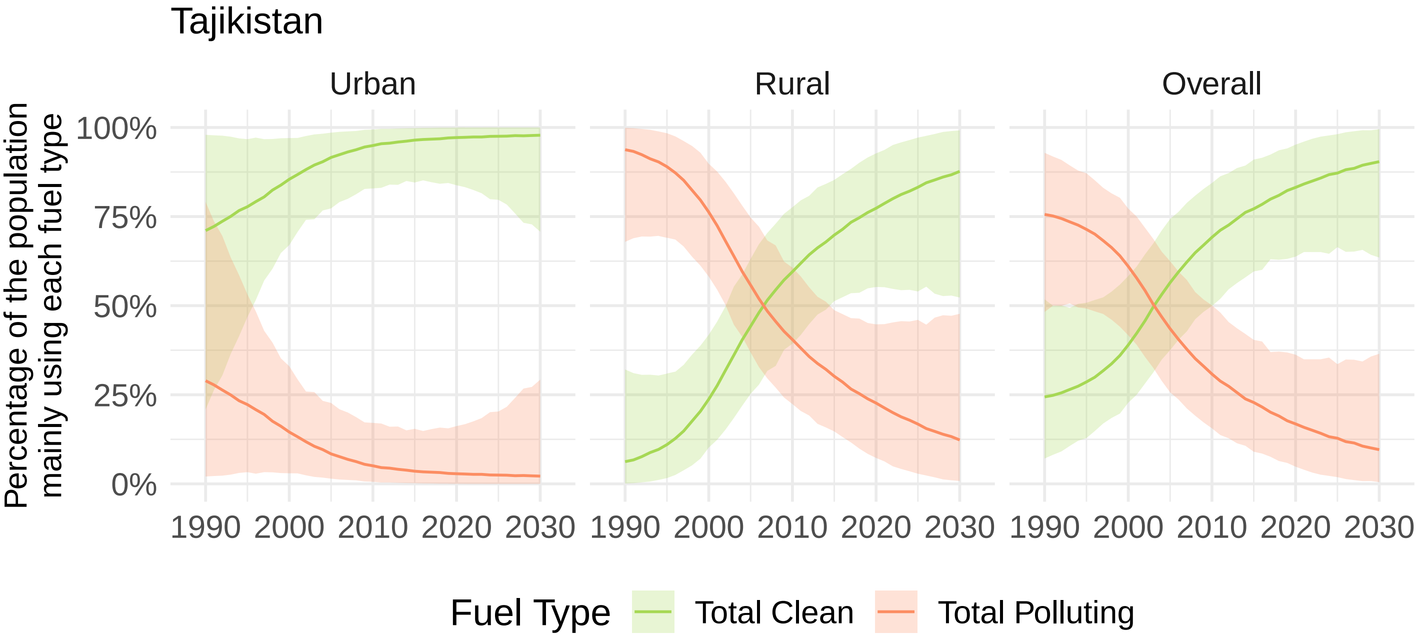
Tajikistan
Urban
Rural
Overall
Percentage of the population mainly using each fuel type
100%
75%
50%
25%
0%
1990
2000
2010
2020
2030
Fuel Type
Total Clean
Total Polluting

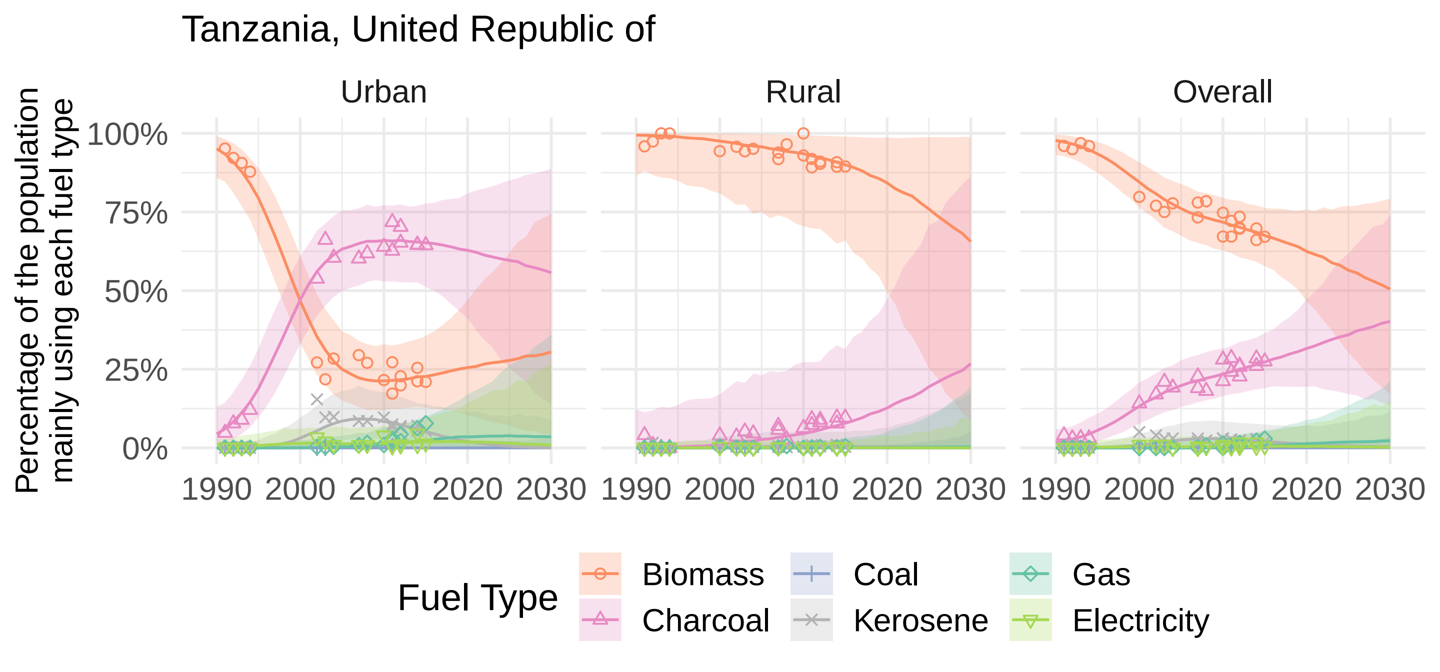

Tanzania, United Republic of
Urban
Rural
Overall
Percentage of the population mainly using each fuel type
100%
75%
50%
25%
0%
1990
2000
2010
2020
2030
Fuel Type
Biomass
Charcoal
Coal
Kerosene
Gas
Electricity

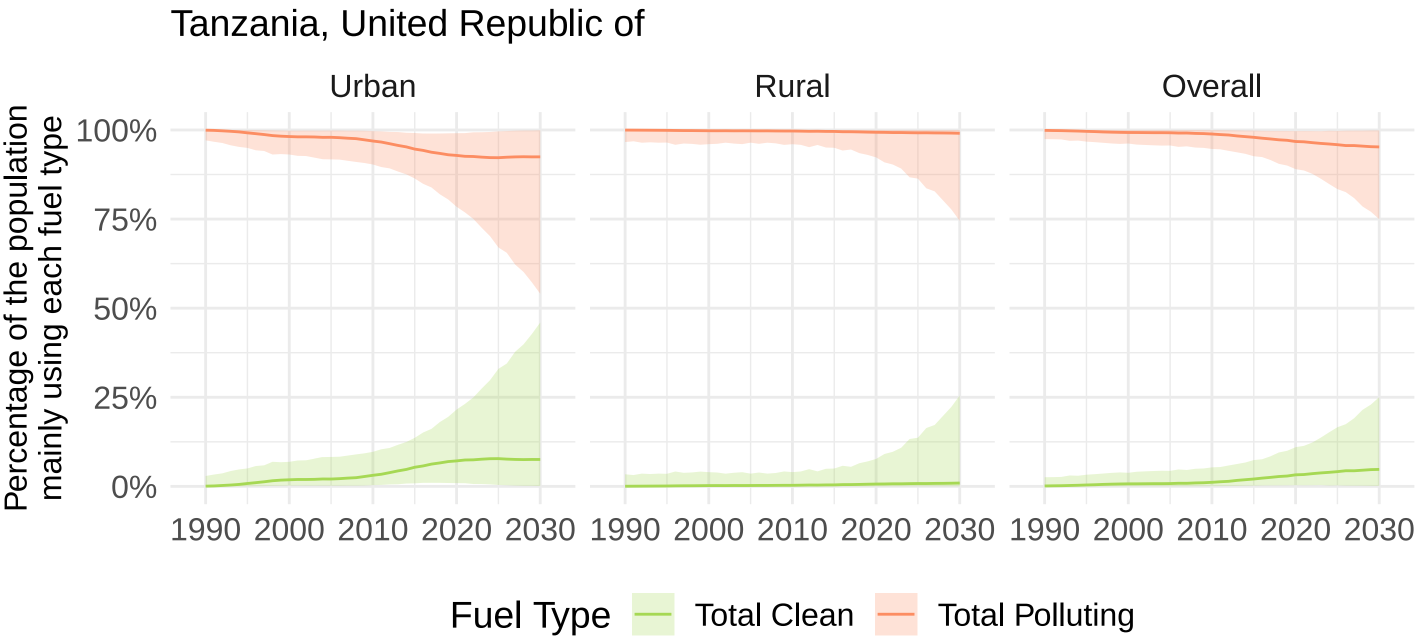
Tanzania, United Republic of
Urban
Rural
Overall
Percentage of the population mainly using each fuel type
100%
75%
50%
25%
0%
1990
2000
2010
2020
2030
Fuel Type
Total Clean
Total Polluting

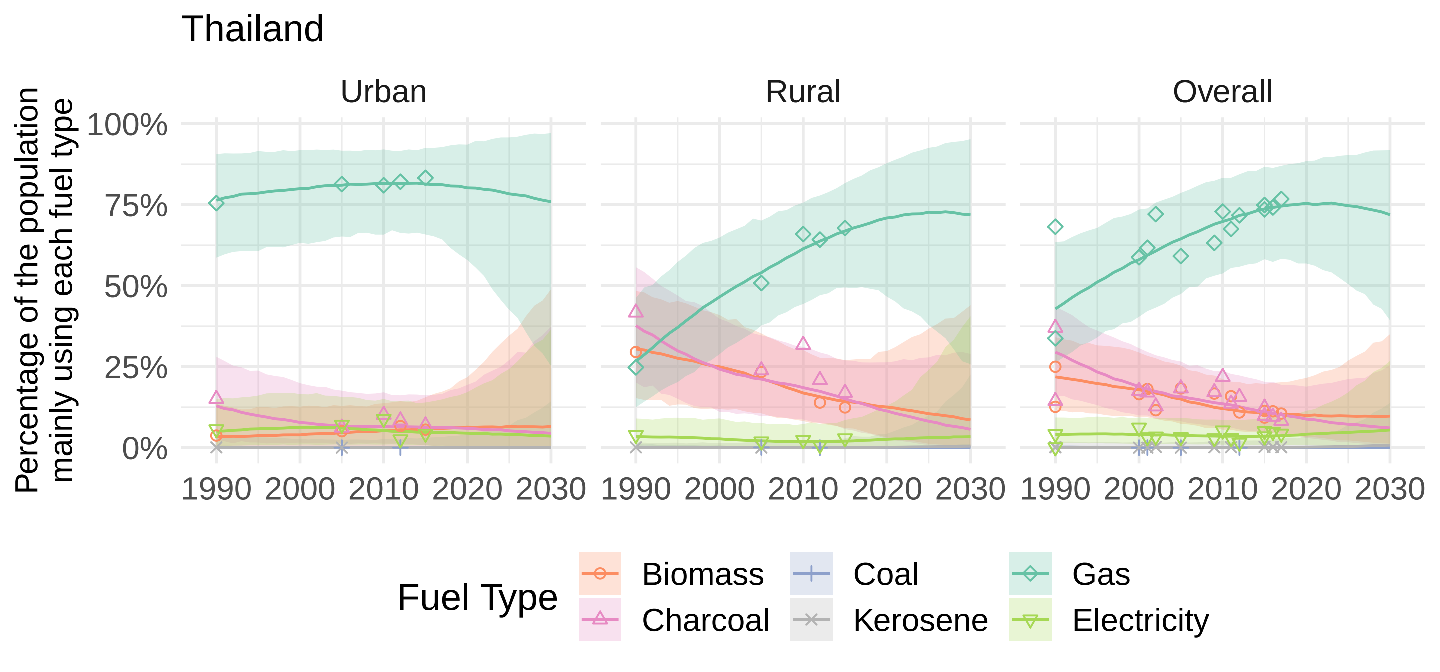
Thailand
Urban
Rural
Overall
Percentage of the population mainly using each fuel type
100%
75%
50%
25%
0%
1990
2000
2010
2020
2030
Fuel Type
Biomass
Charcoal
Coal
Kerosene
Gas
Electricity

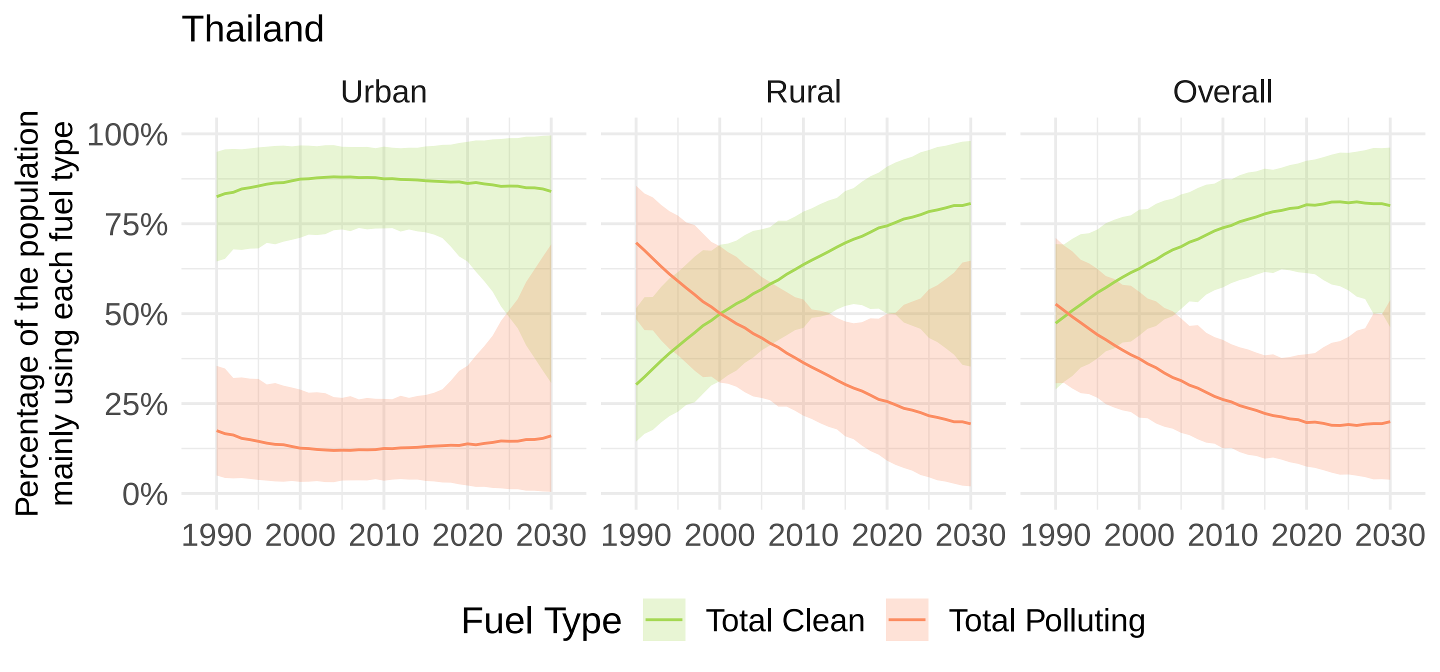
Thailand
Urban
Rural
Overall
Percentage of the population mainly using each fuel type
100%
75%
50%
25%
0%
1990
2000
2010
2020
2030
Fuel Type
Total Clean
Total Polluting

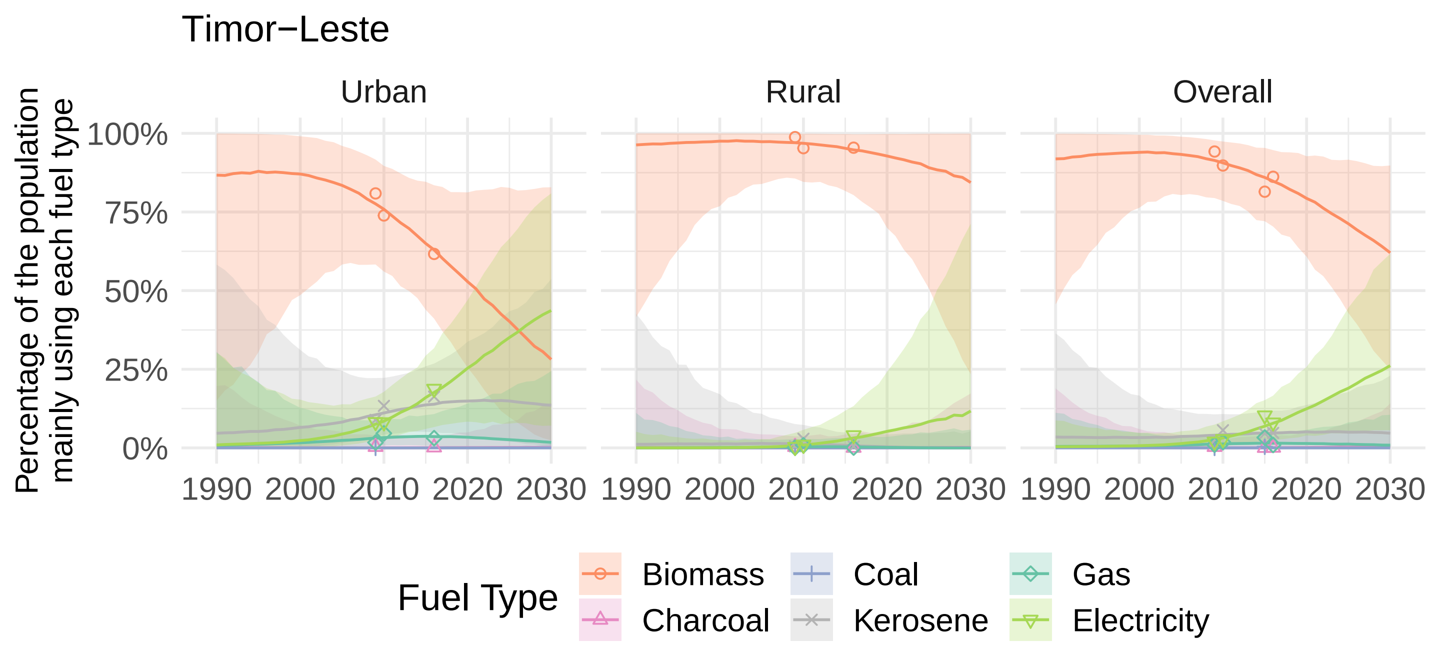
Timor−Leste
Urban
Rural
Overall
Percentage of the population mainly using each fuel type
100%
75%
50%
25%
0%
1990
2000
2010
2020
2030
Fuel Type
Biomass
Charcoal
Coal
Kerosene
Gas
Electricity

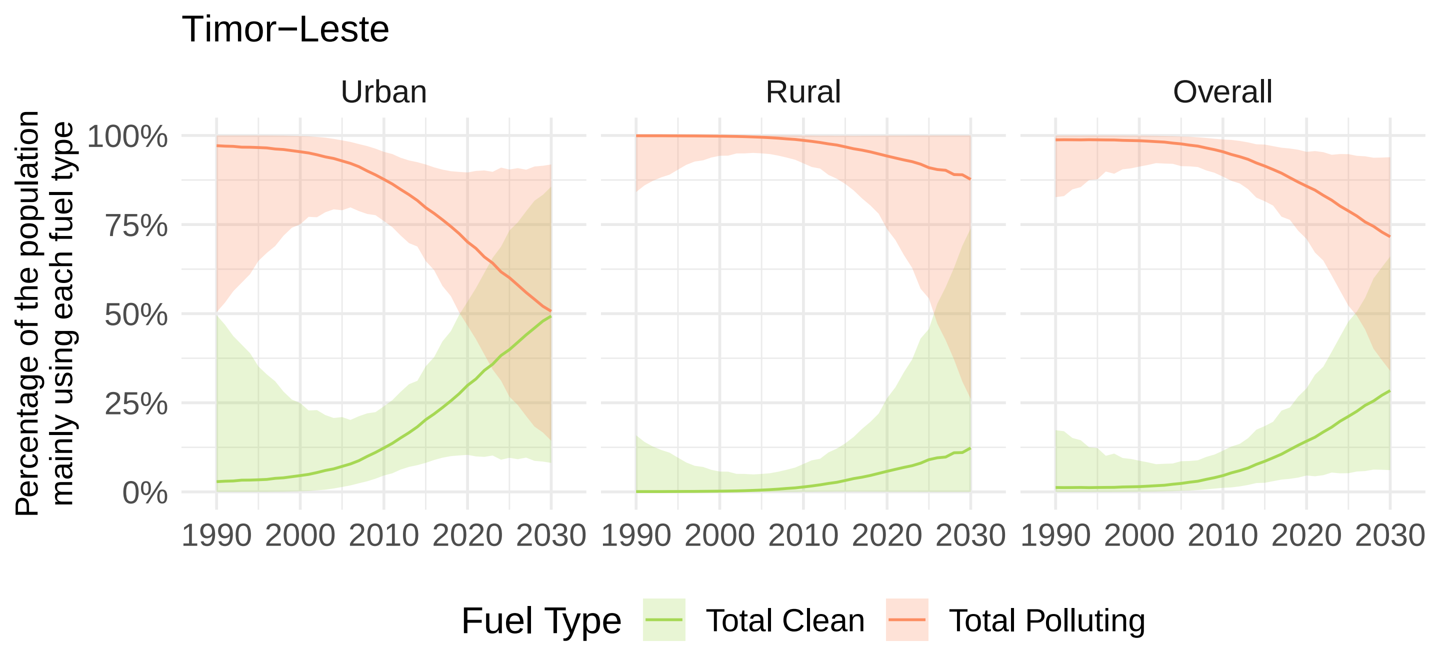
Timor−Leste
Urban
Rural
Overall
Percentage of the population mainly using each fuel type
100%
75%
50%
25%
0%
1990
2000
2010
2020
2030
Fuel Type
Total Clean
Total Polluting

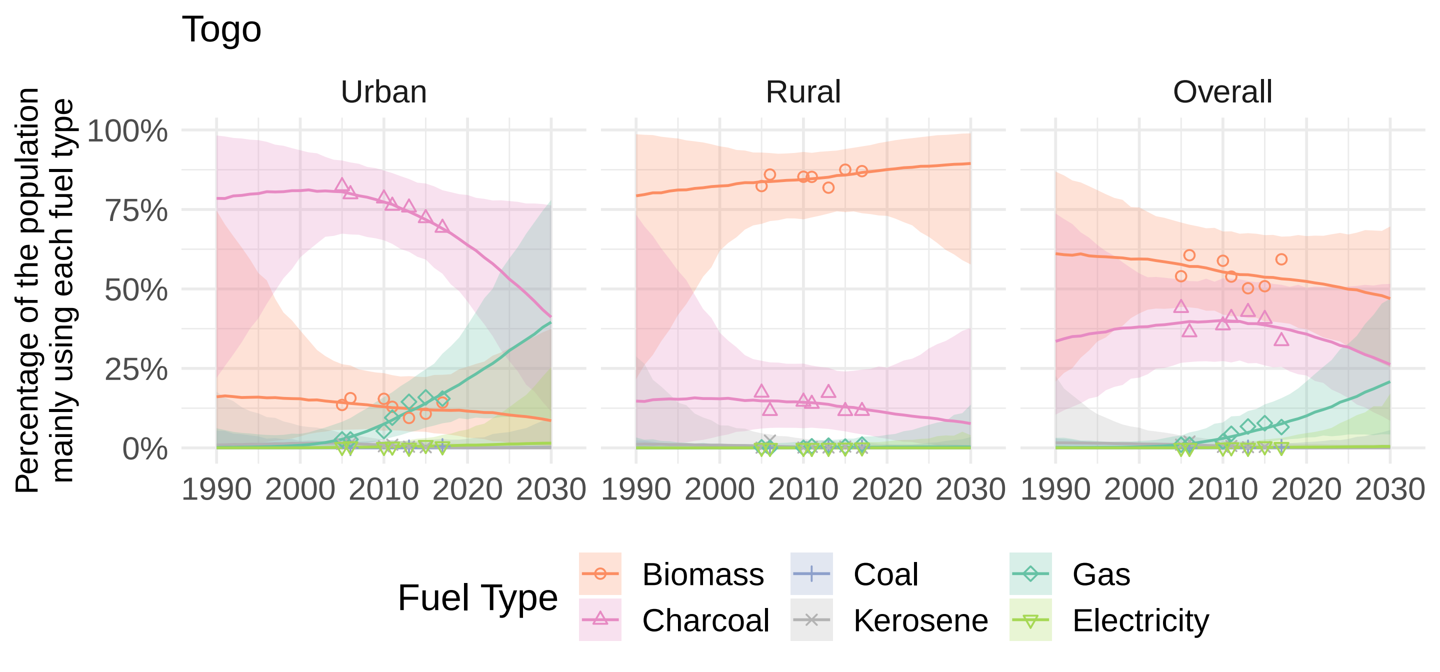

Togo
Urban
Rural
Overall
Percentage of the population mainly using each fuel type
100%
75%
50%
25%
0%
1990
2000
2010
2020
2030
Fuel Type
Biomass
Charcoal
Coal
Kerosene
Gas
Electricity

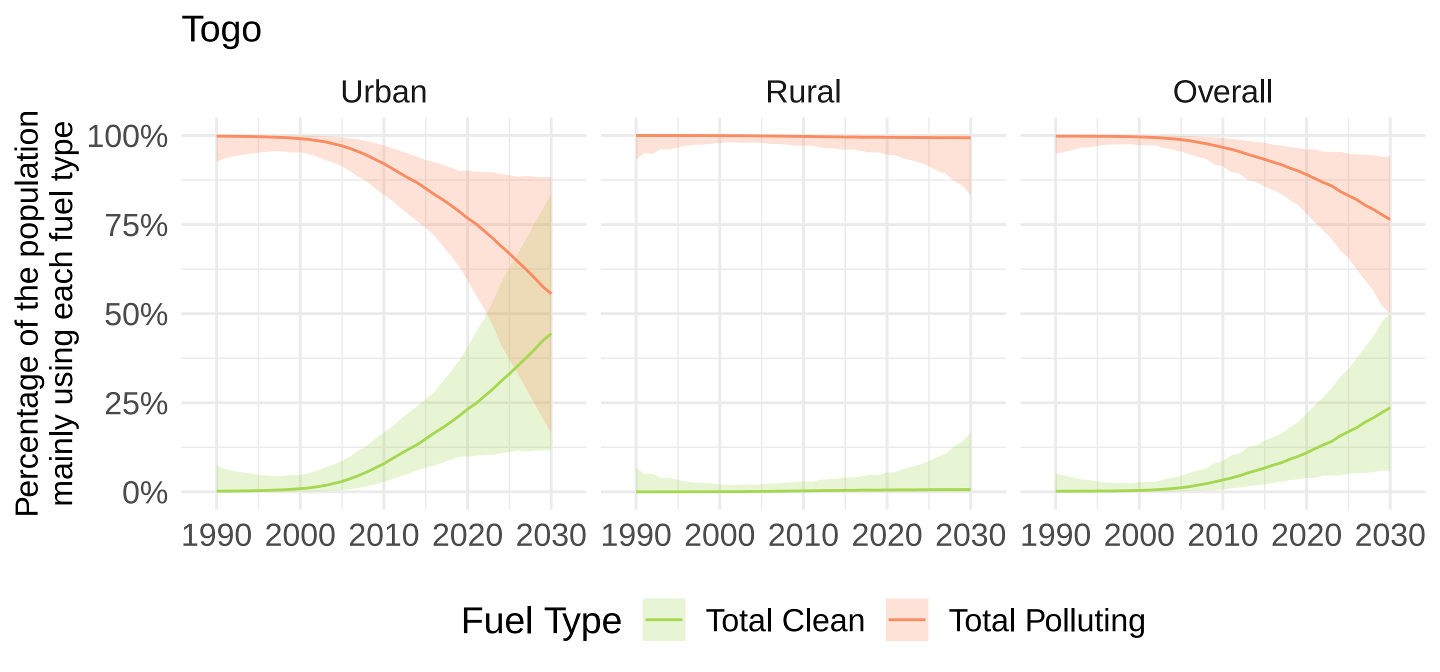

Togo
Urban
Rural
Overall
Percentage of the population mainly using each fuel type
100%
75%
50%
25%
0%
1990
2000
2010
2020
2030
Fuel Type
Total Clean
Total Polluting

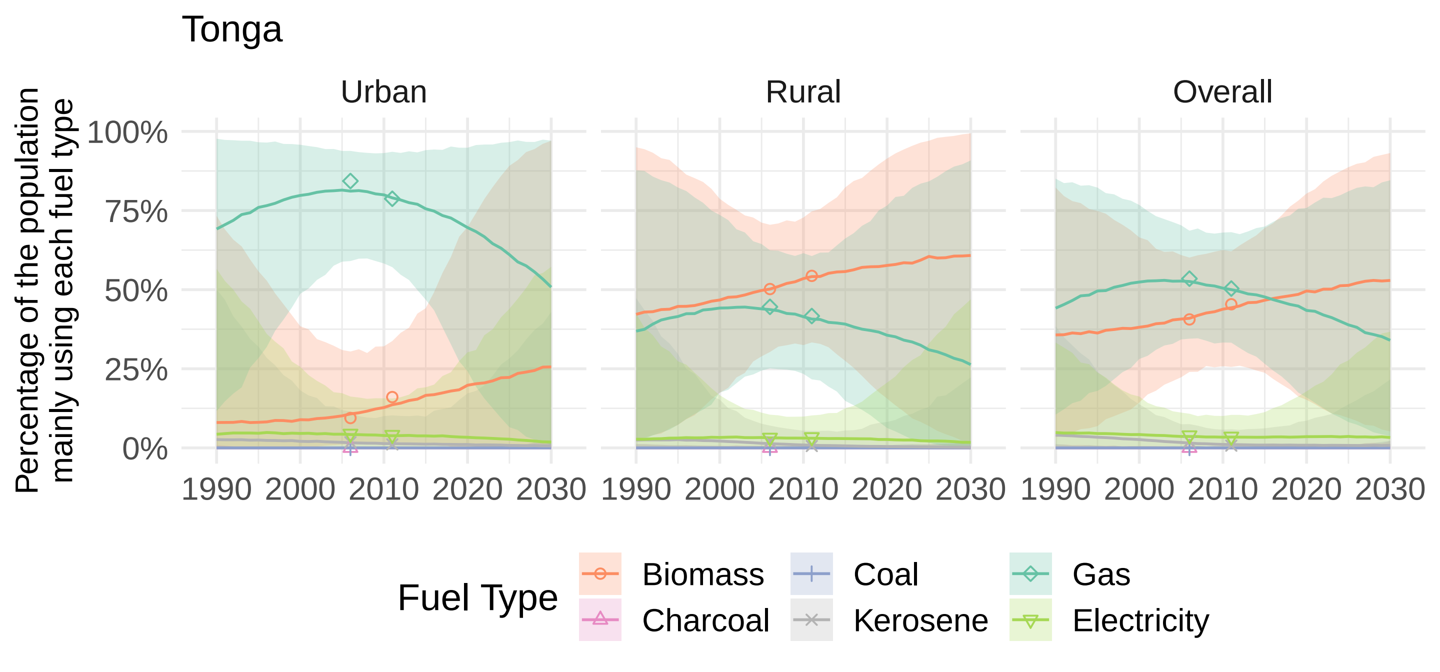
Tonga
Urban
Rural
Overall
Percentage of the population mainly using each fuel type
100%
75%
50%
25%
0%
1990
2000
2010
2020
2030
Fuel Type
Biomass
Charcoal
Coal
Kerosene
Gas
Electricity

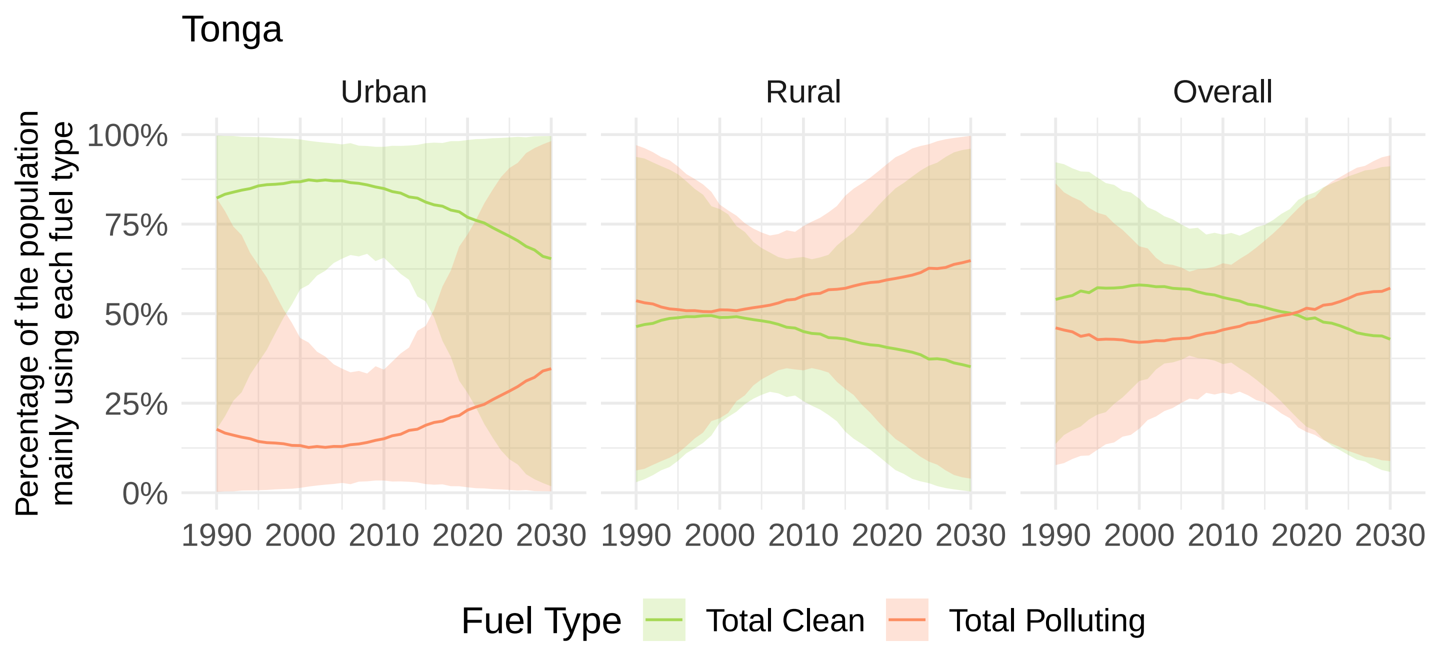

Tonga
Urban
Rural
Overall
Percentage of the population mainly using each fuel type
100%
75%
50%
25%
0%
1990
2000
2010
2020
2030
Fuel Type
Total Clean
Total Polluting

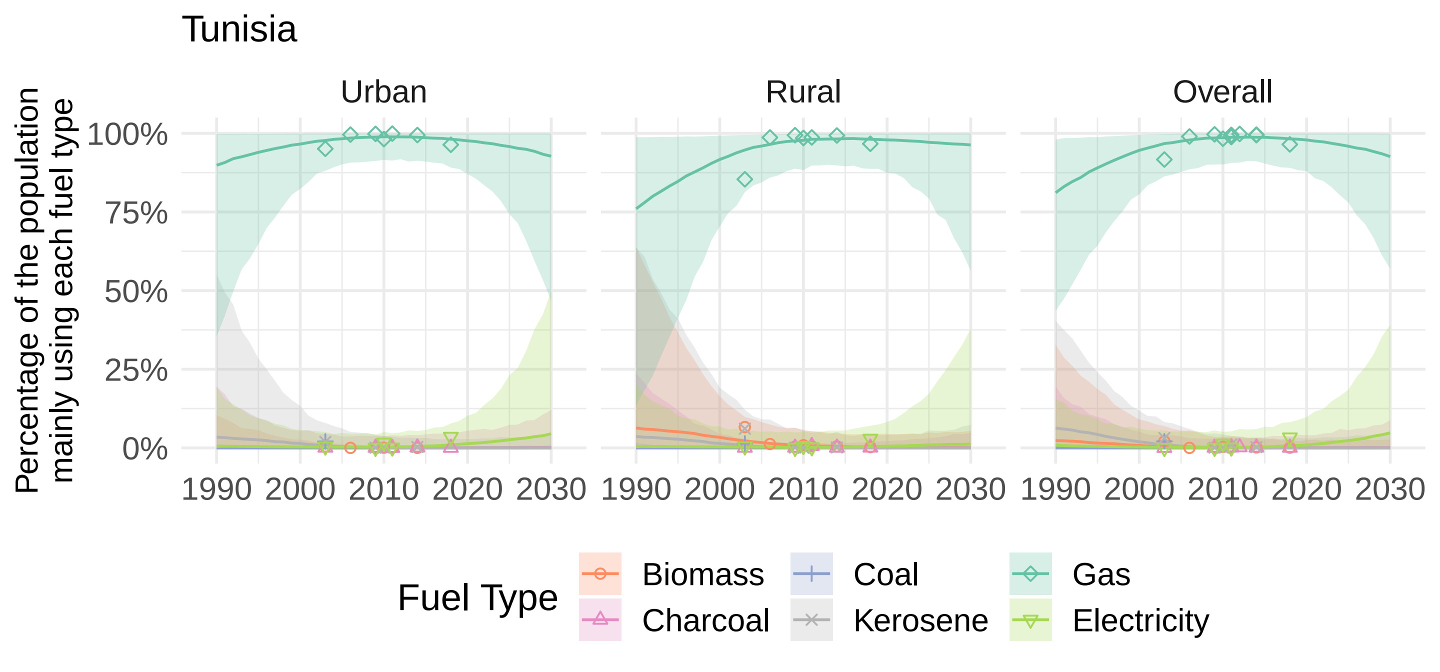
Tunisia
Urban
Rural
Overall
Percentage of the population mainly using each fuel type
100%
75%
50%
25%
0%
1990
2000
2010
2020
2030
Fuel Type
Biomass
Charcoal
Coal
Kerosene
Gas
Electricity

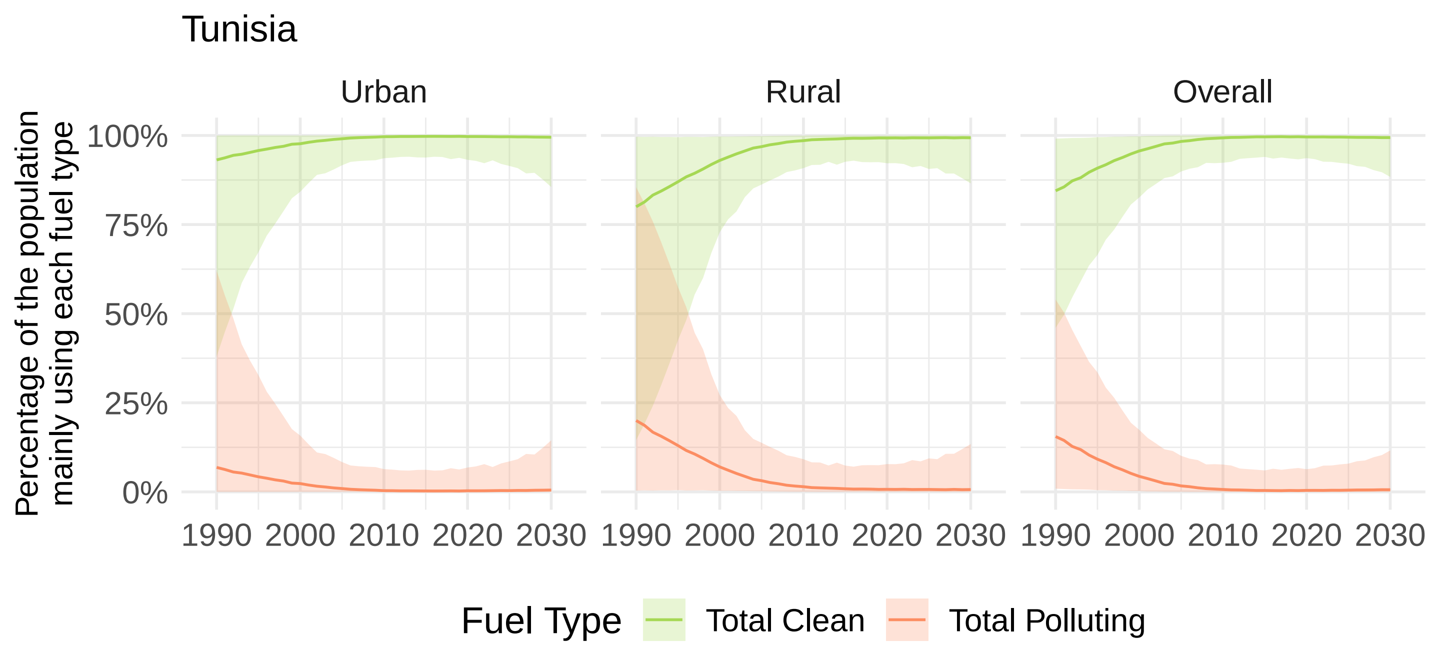

Tunisia
Urban
Rural
Overall
Percentage of the population mainly using each fuel type
100%
75%
50%
25%
0%
1990
2000
2010
2020
2030
Fuel Type
Total Clean
Total Polluting

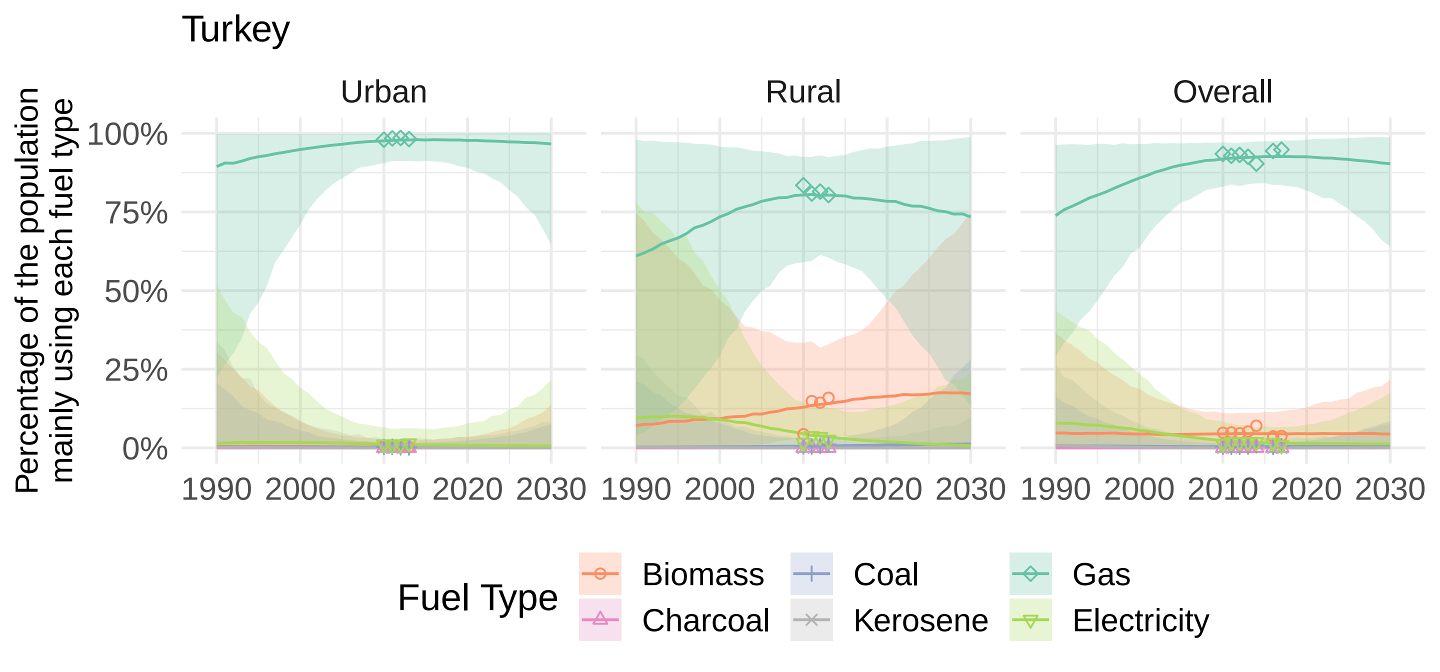

Turkey
Urban
Rural
Overall
Percentage of the population mainly using each fuel type
100%
75%
50%
25%
0%
1990
2000
2010
2020
2030
Fuel Type
Biomass
Charcoal
Coal
Kerosene
Gas
Electricity

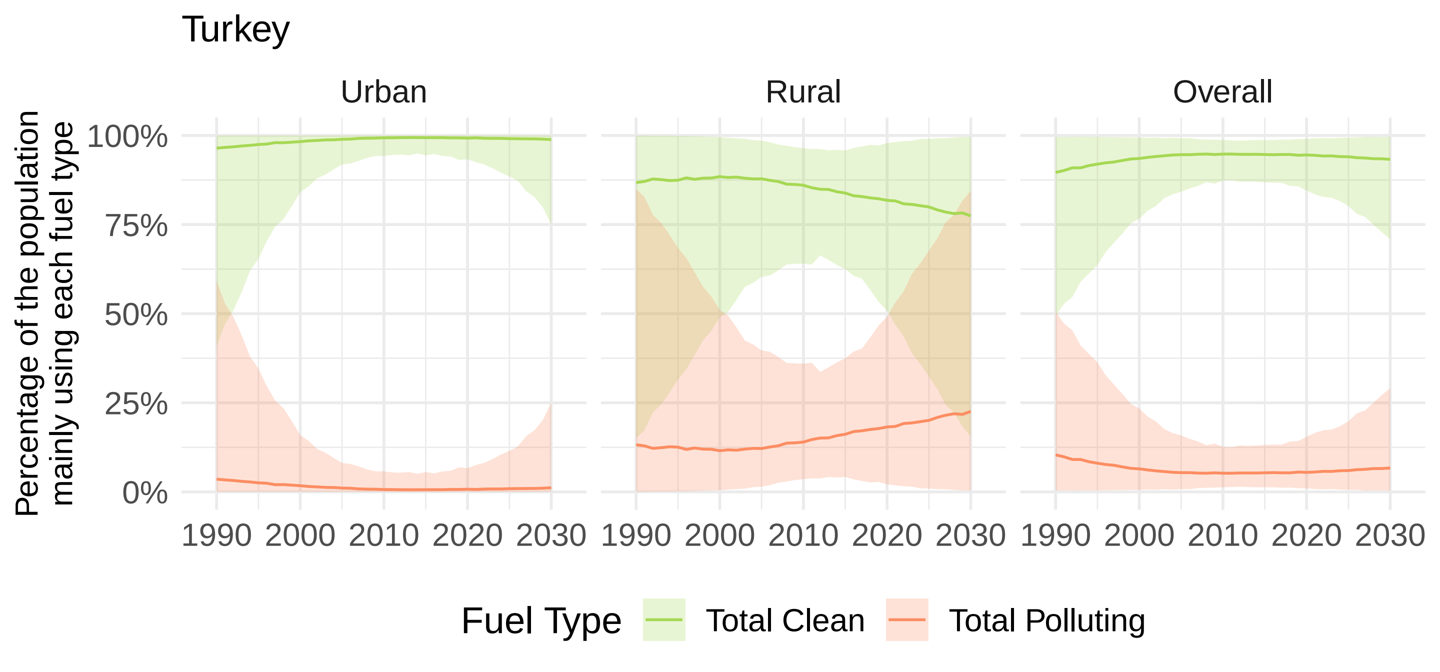
Turkey
Urban
Rural
Overall
Percentage of the population mainly using each fuel type
100%
75%
50%
25%
0%
1990
2000
2010
2020
2030
Fuel Type
Total Clean
Total Polluting

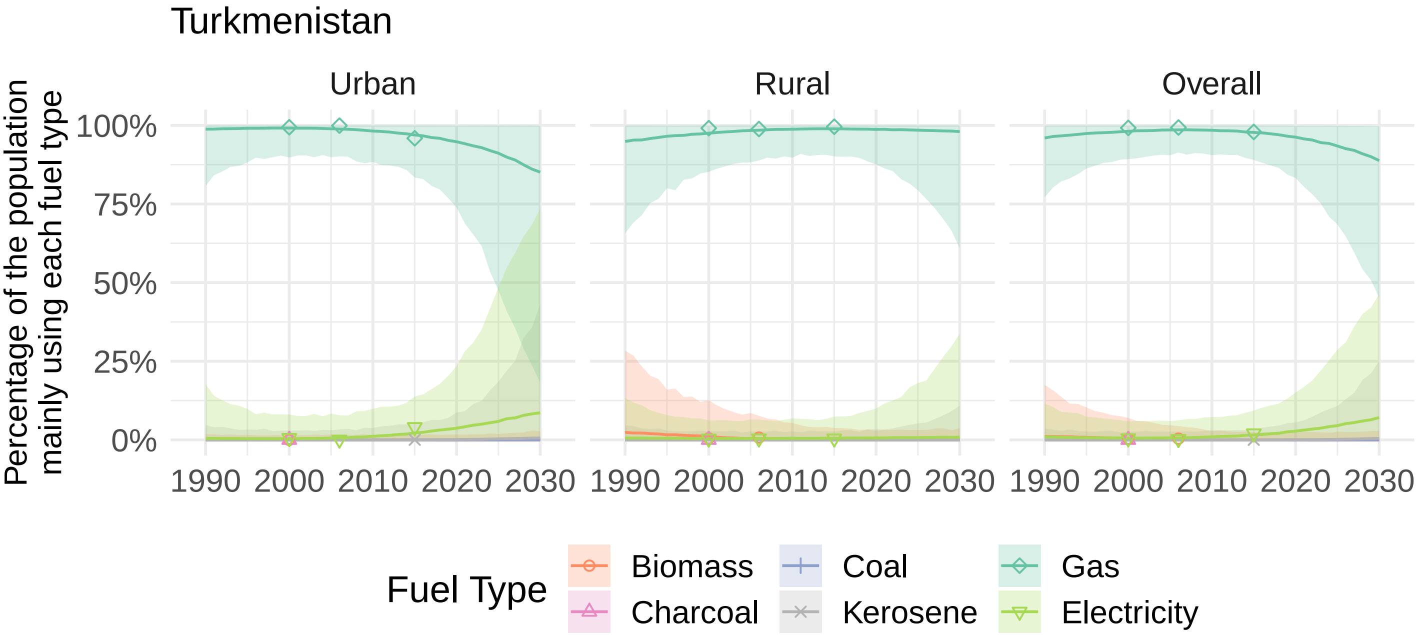
Turkmenistan
Urban
Rural
Overall
Percentage of the population mainly using each fuel type
100%
75%
50%
25%
0%
1990
2000
2010
2020
2030
Fuel Type
Biomass
Charcoal
Coal
Kerosene
Gas
Electricity

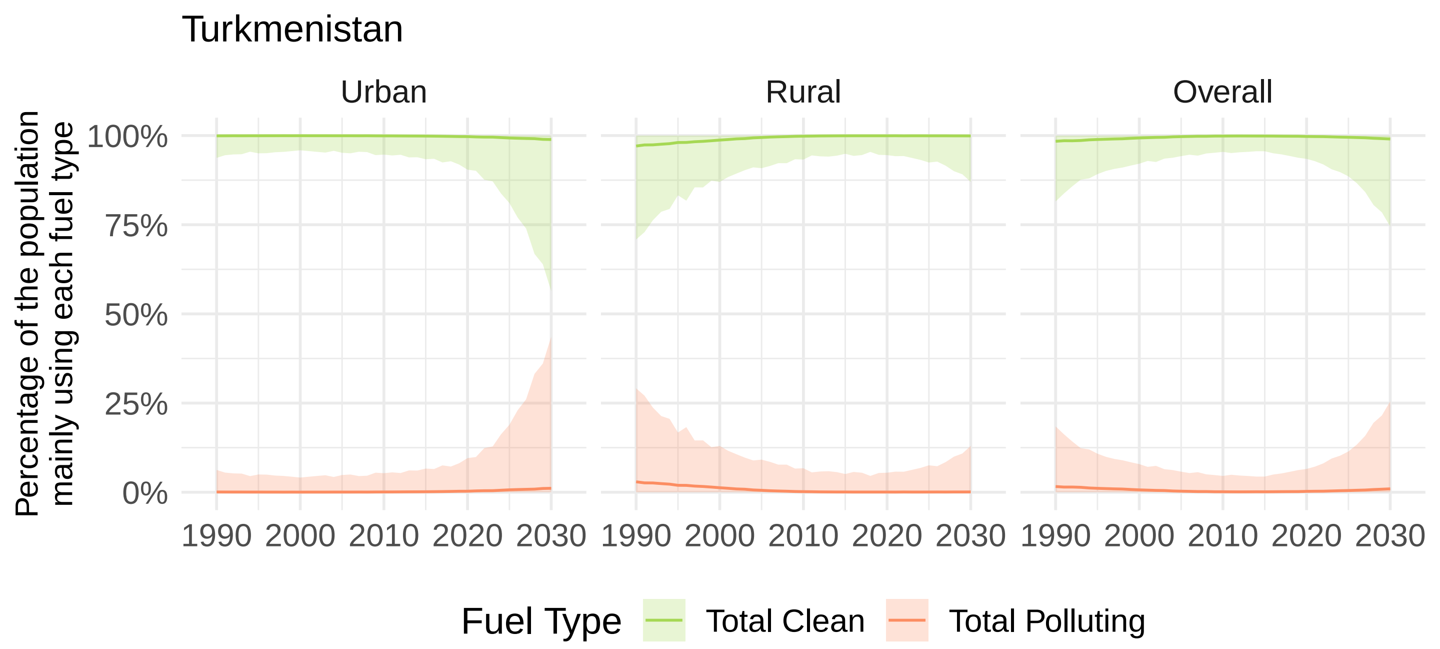
Turkmenistan
Urban
Rural
Overall
Percentage of the population mainly using each fuel type
100%
75%
50%
25%
0%
1990
2000
2010
2020
2030
Fuel Type
Total Clean
Total Polluting

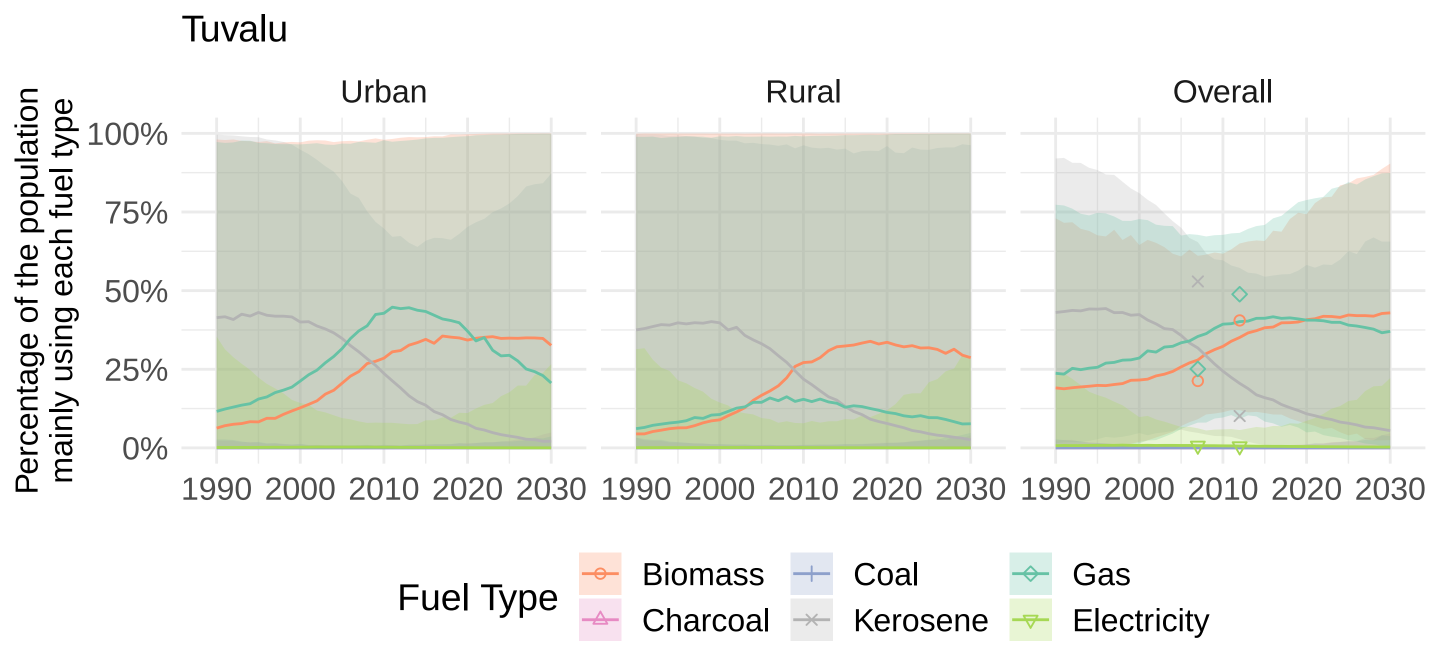

Tuvalu
Urban
Rural
Overall
Percentage of the population mainly using each fuel type
100%
75%
50%
25%
0%
1990
2000
2010
2020
2030
Fuel Type
Biomass
Charcoal
Coal
Kerosene
Gas
Electricity

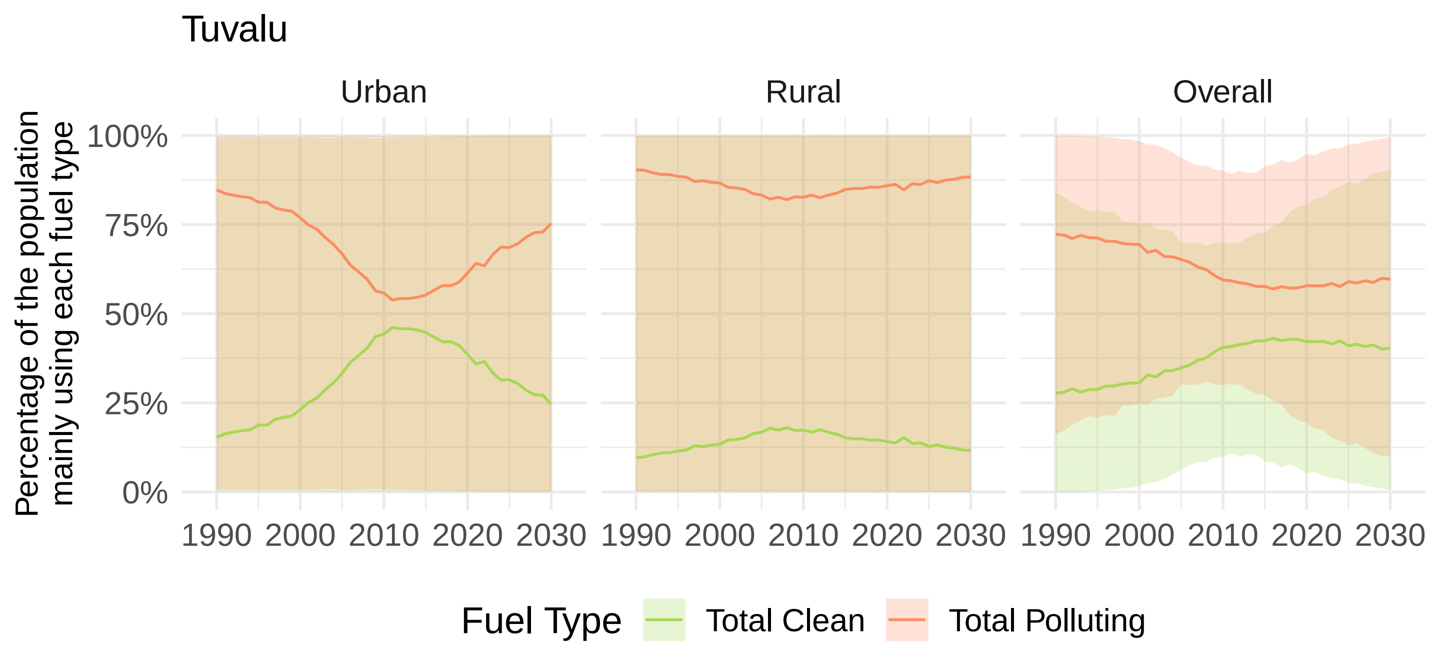
Tuvalu
Urban
Rural
Overall
Percentage of the population
mainly using each fuel type
100%
75%
50%
25%
0%
1990
2000
2010
2020
2030
Fuel Type
Total Clean
Total Polluting

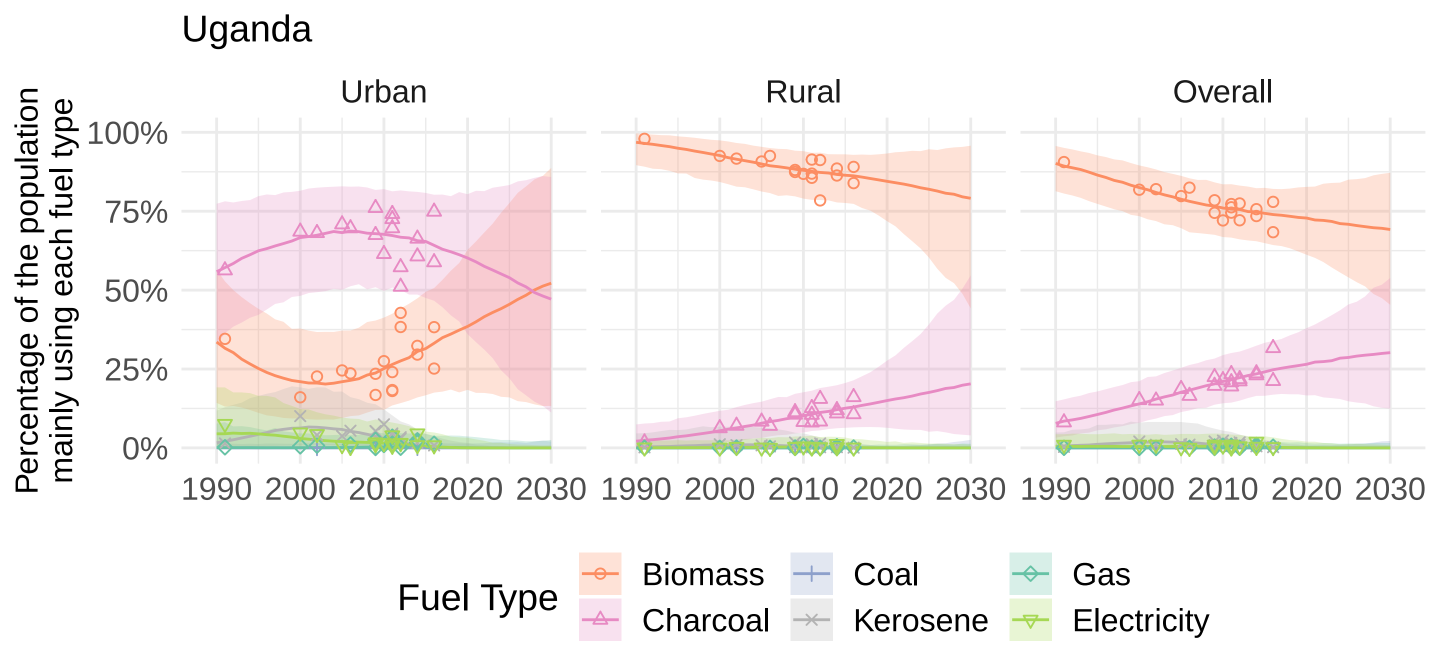
Uganda
Urban
Rural
Overall
Percentage of the population mainly using each fuel type
100%
75%
50%
25%
0%
1990
2000
2010
2020
2030
Fuel Type
Biomass
Charcoal
Coal
Kerosene
Gas
Electricity

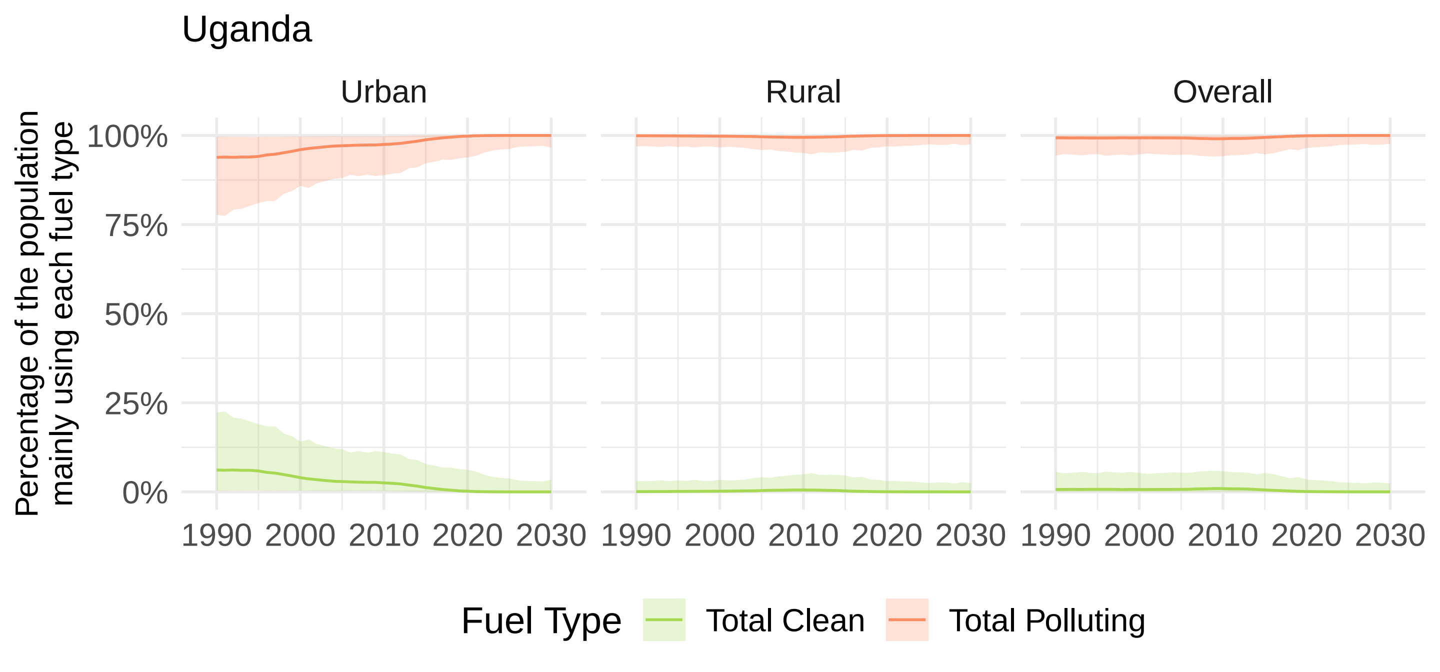
Uganda
Urban
Rural
Overall
Percentage of the population mainly using each fuel type
100%
75%
50%
25%
0%
1990
2000
2010
2020
2030
Fuel Type
Total Clean
Total Polluting

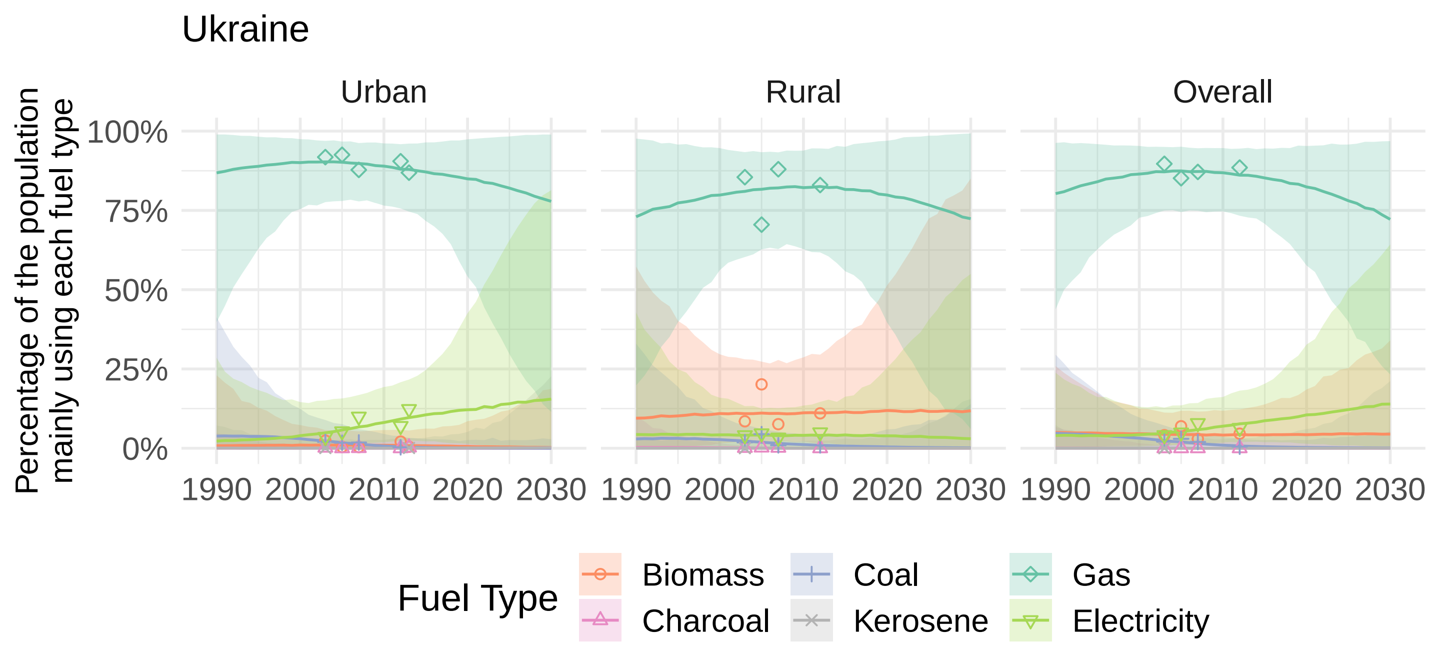
Ukraine
Urban
Rural
Overall
Percentage of the population
mainly using each fuel type
100%
75%
50%
25%
0%
1990
2000
2010
2020
2030
Fuel Type
Biomass
Charcoal
Coal
Kerosene
Gas
Electricity

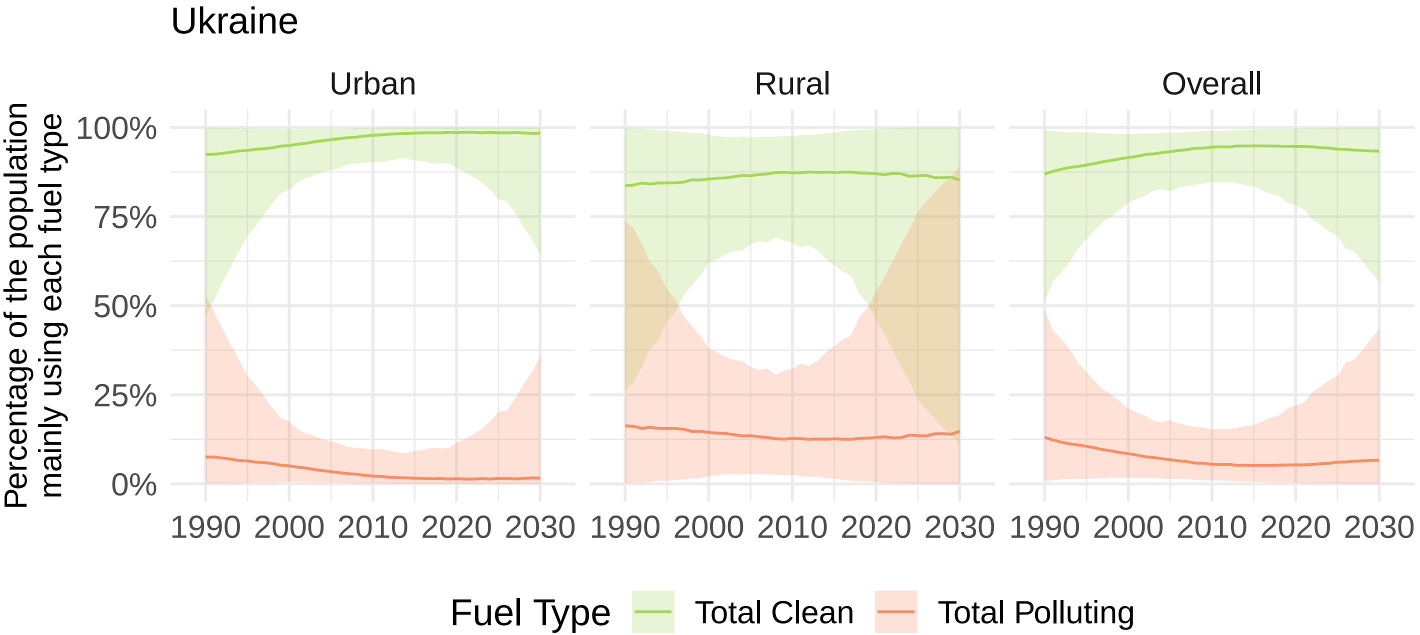
Ukraine
Urban
Rural
Overall
Percentage of the population mainly using each fuel type
100%
75%
50%
25%
0%
1990
2000
2010
2020
2030
Fuel Type
Total Clean
Total Polluting

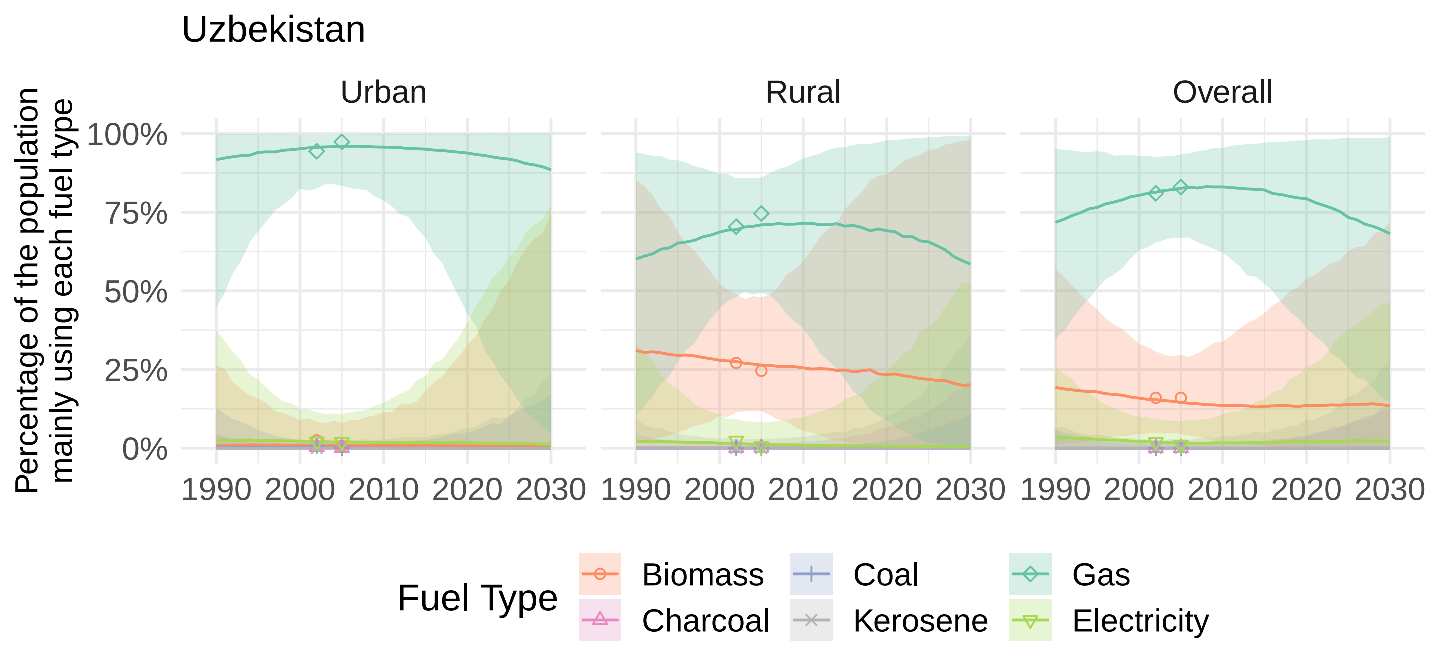
Uzbekistan
Urban
Rural
Overall
Percentage of the population mainly using each fuel type
100%
75%
50%
25%
0%
1990
2000
2010
2020
2030
Fuel Type
Biomass
Charcoal
Coal
Kerosene
Gas
Electricity

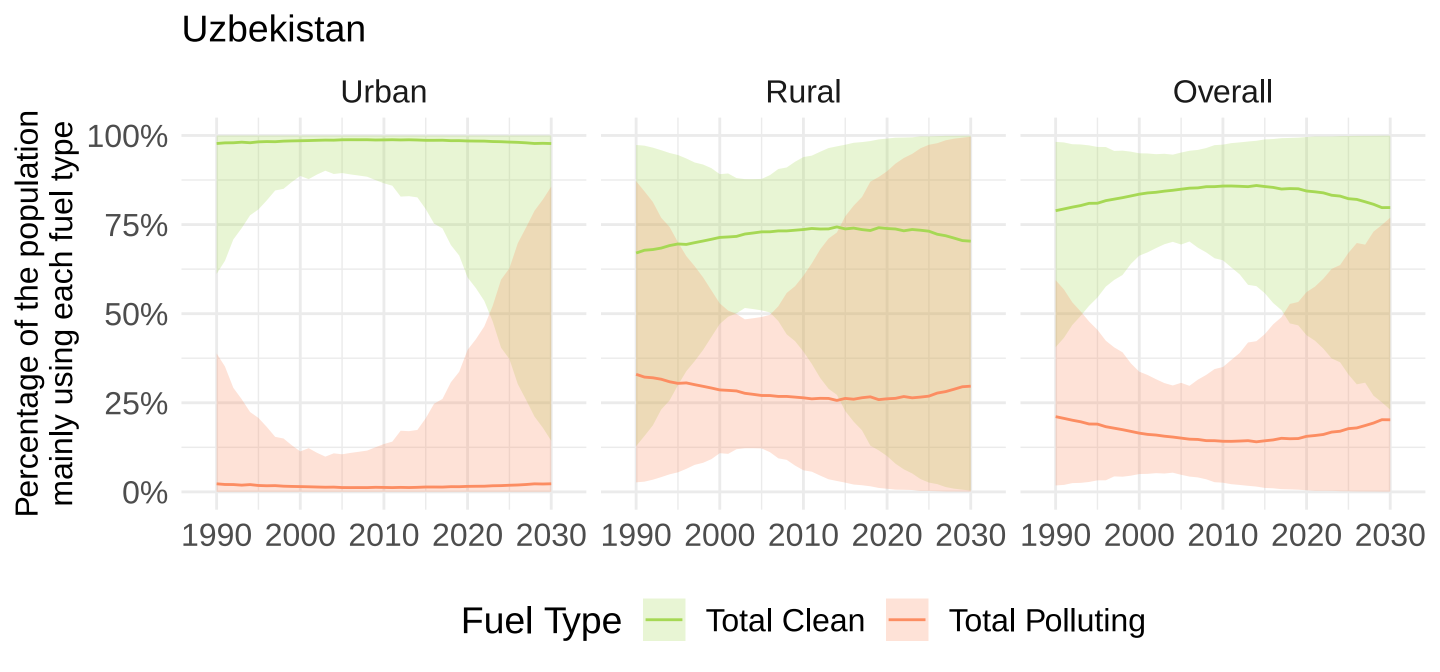
Uzbekistan
Urban
Rural
Overall
Percentage of the population mainly using each fuel type
100%
75%
50%
25%
0%
1990
2000
2010
2020
2030
Fuel Type
Total Clean
Total Polluting

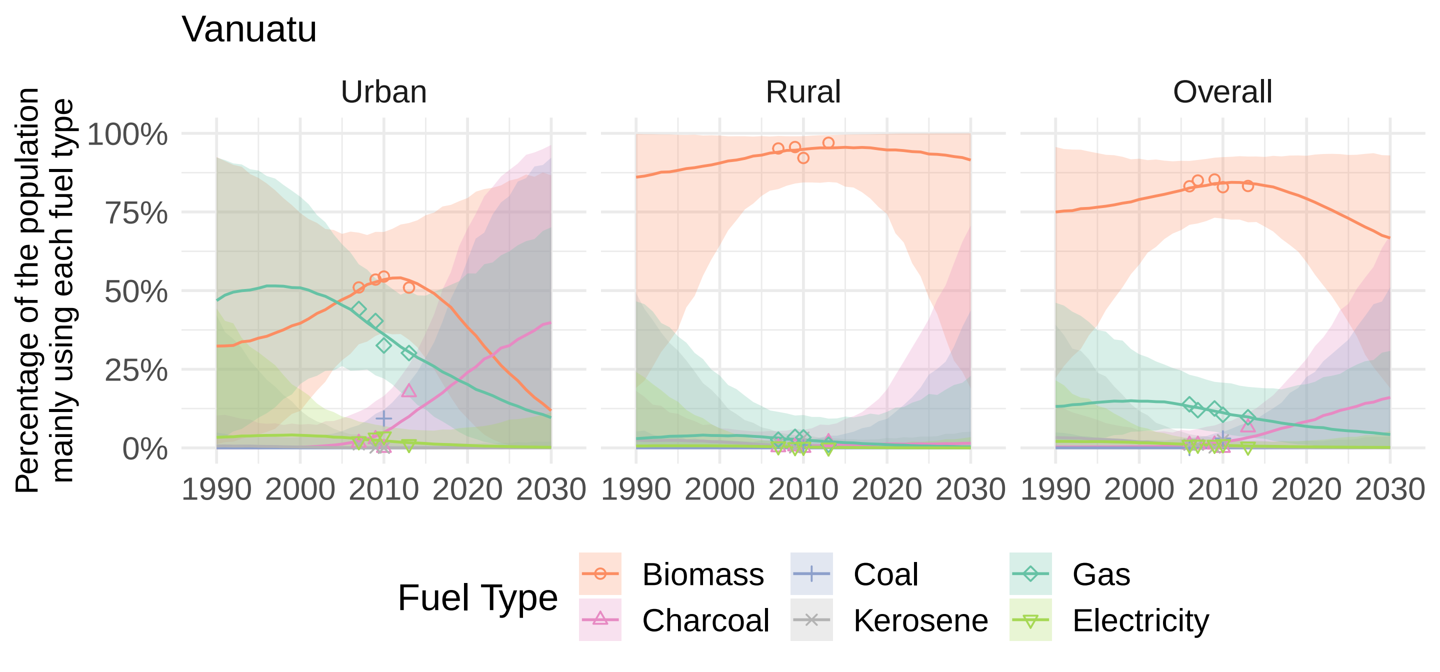
Vanuatu
Urban
Rural
Overall
Percentage of the population mainly using each fuel type
100%
75%
50%
25%
0%
1990
2000
2010
2020
2030
Fuel Type
Biomass
Charcoal
Coal
Kerosene
Gas
Electricity

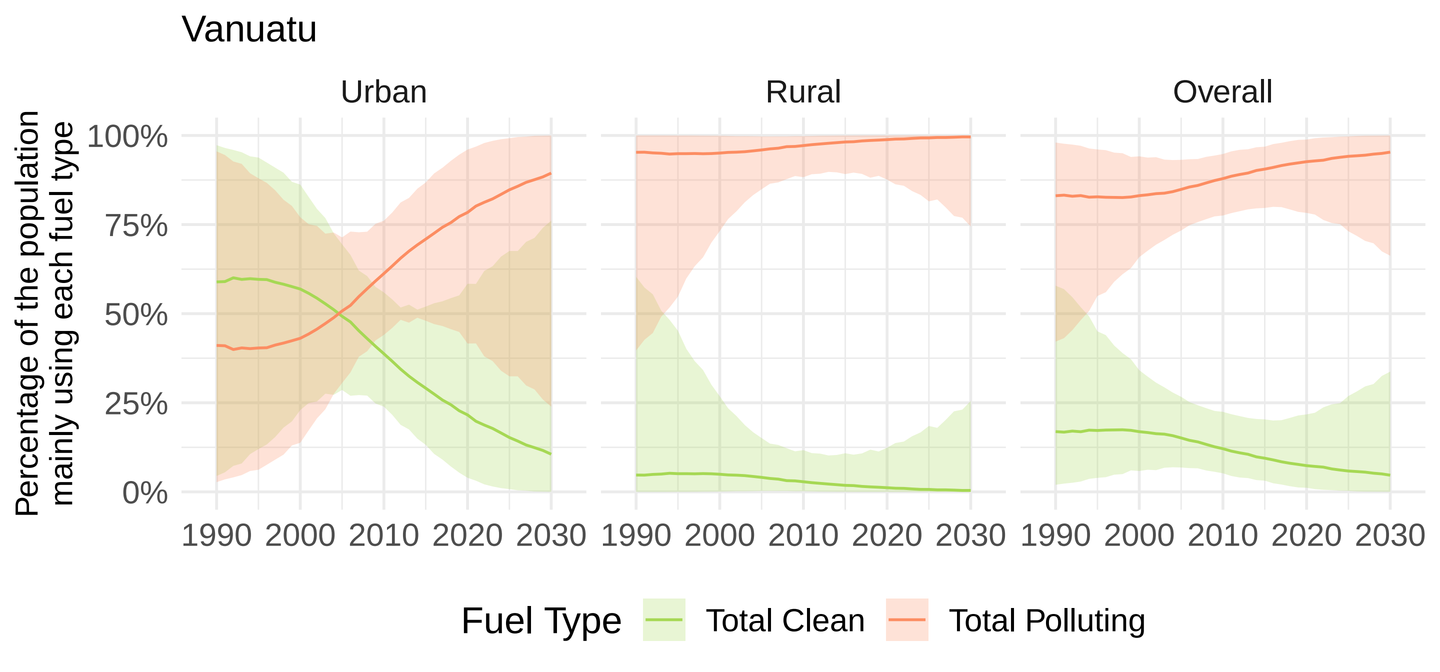
Vanuatu
Urban
Rural
Overall
Percentage of the population mainly using each fuel type
100%
75%
50%
25%
0%
1990
2000
2010
2020
2030
Fuel Type
Total Clean
Total Polluting

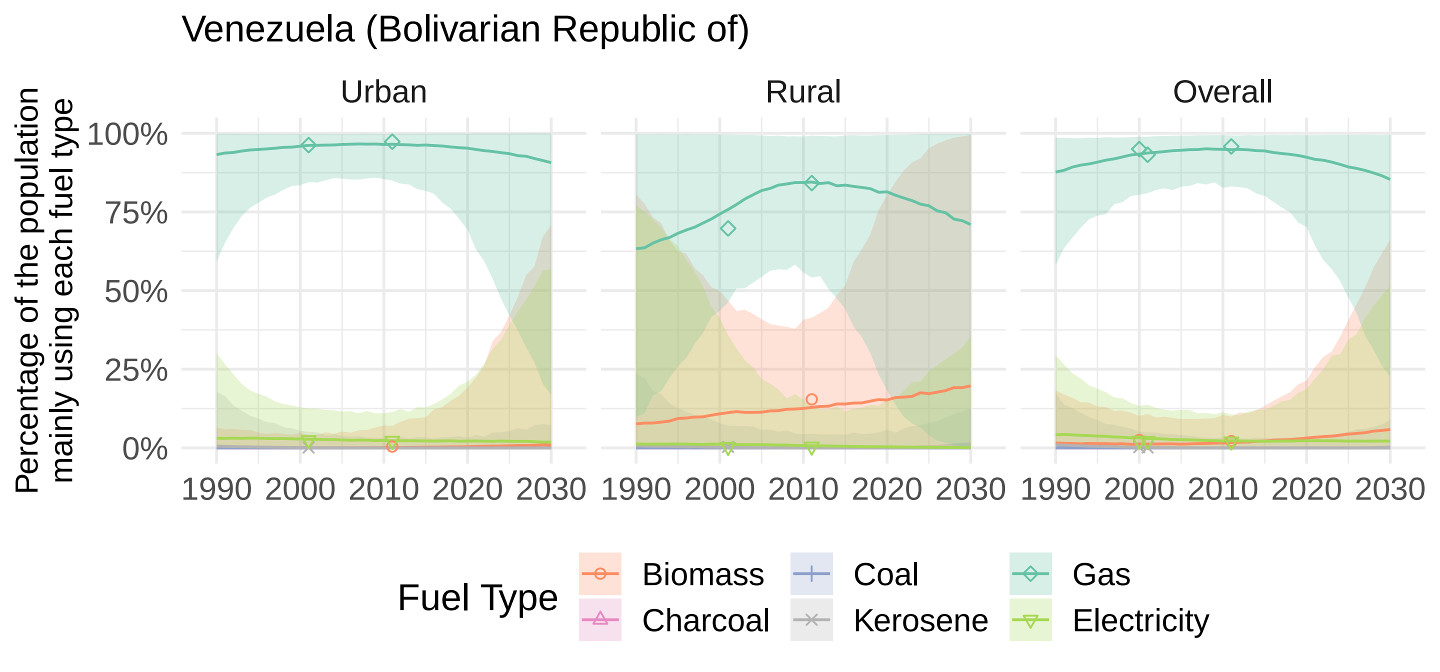

Venezuela (Bolivarian Republic of)
Urban
Rural
Overall
Percentage of the population mainly using each fuel type
100%
75%
50%
25%
0%
1990
2000
2010
2020
2030
Fuel Type
Biomass
Charcoal
Coal
Kerosene
Gas
Electricity

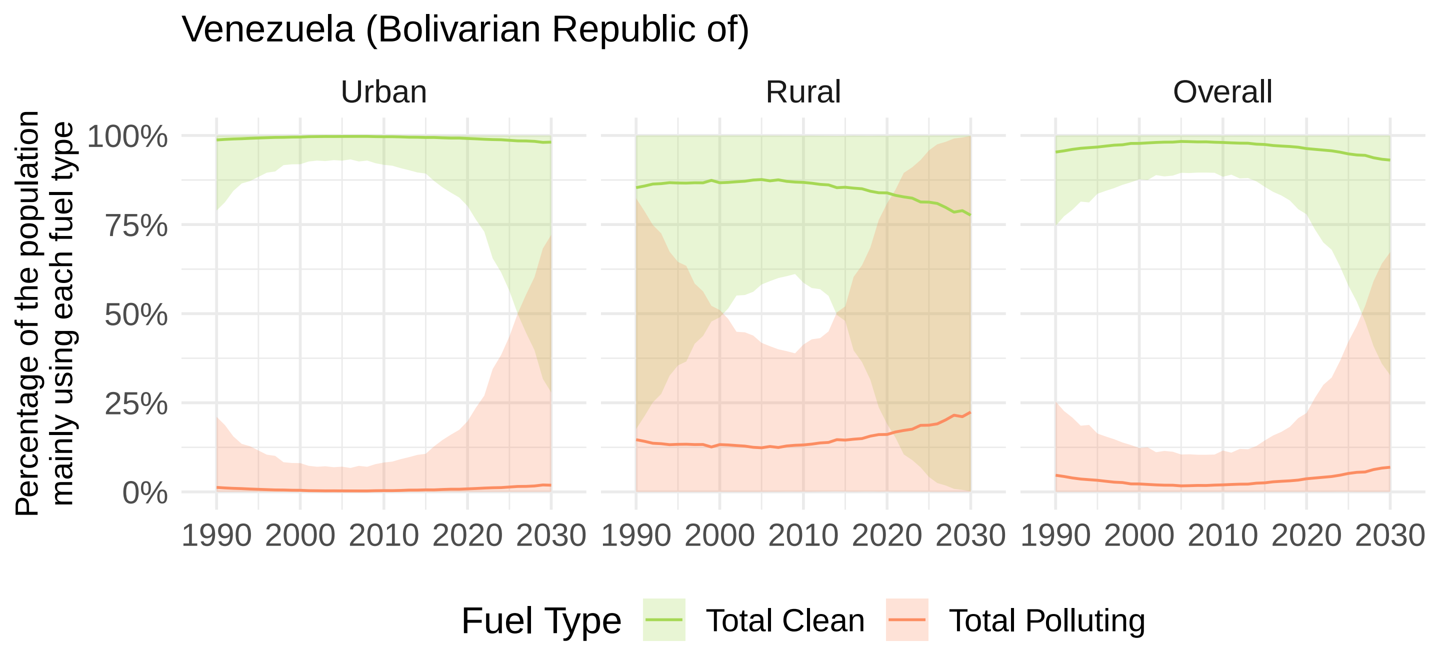
Venezuela (Bolivarian Republic of)
Urban
Rural
Overall
Percentage of the population mainly using each fuel type
100%
75%
50%
25%
0%
1990
2000
2010
2020
2030
Fuel Type
Total Clean
Total Polluting

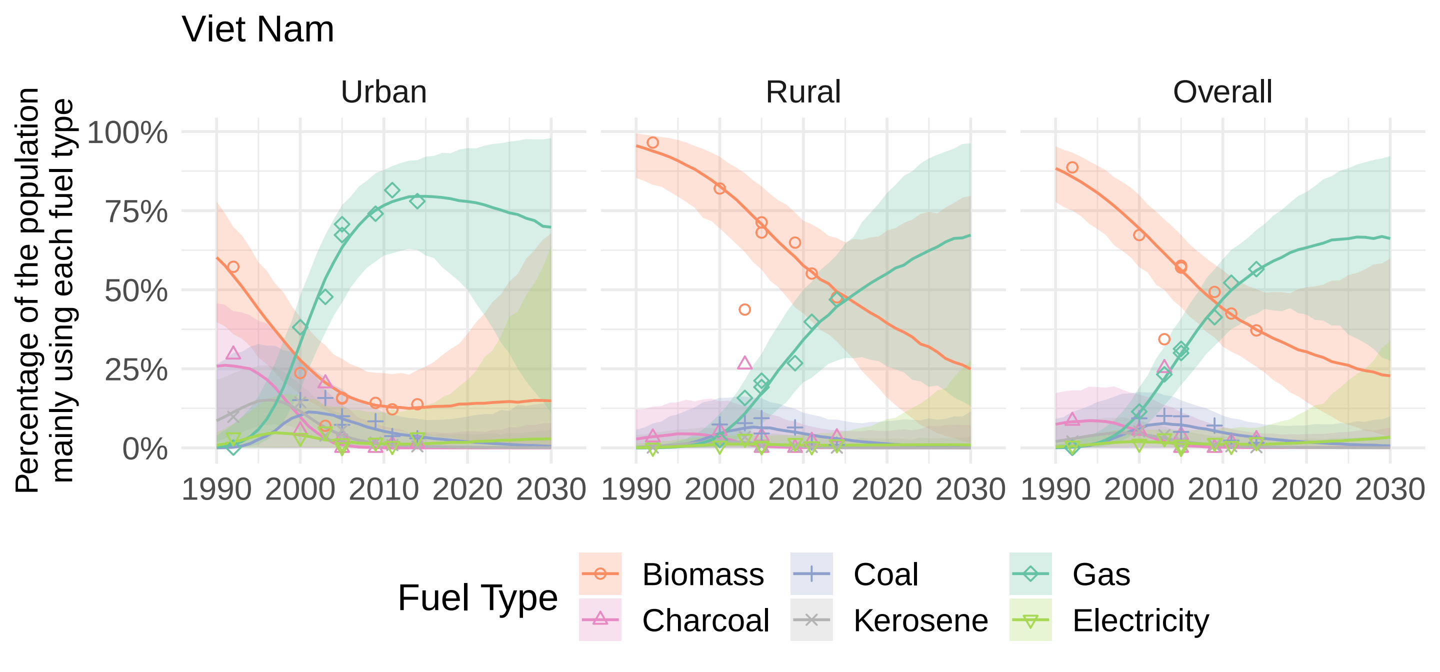

Viet Nam
Urban
Rural
Overall
Percentage of the population mainly using each fuel type
100%
75%
50%
25%
0%
1990
2000
2010
2020
2030
Fuel Type
Biomass
Charcoal
Coal
Kerosene
Gas
Electricity

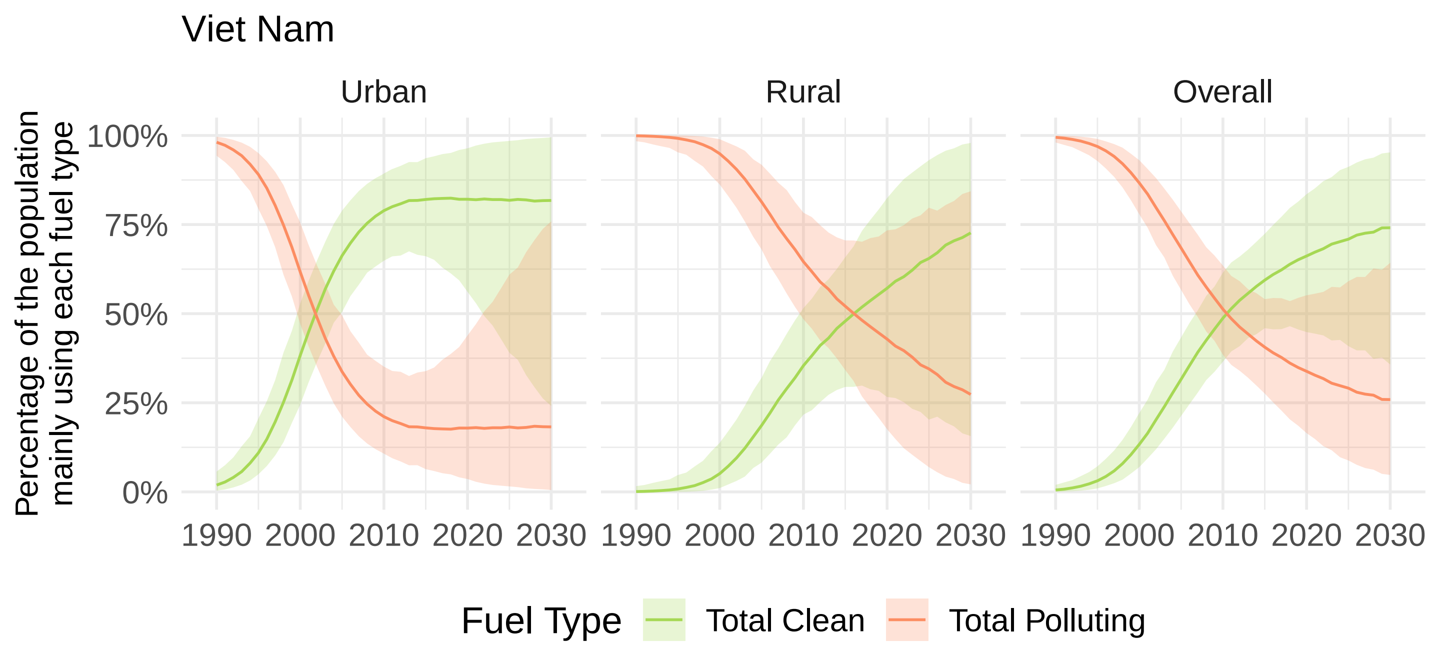
Viet Nam
Urban
Rural
Overall
Percentage of the population mainly using each fuel type
100%
75%
50%
25%
0%
1990
2000
2010
2020
2030
Fuel Type
Total Clean
Total Polluting

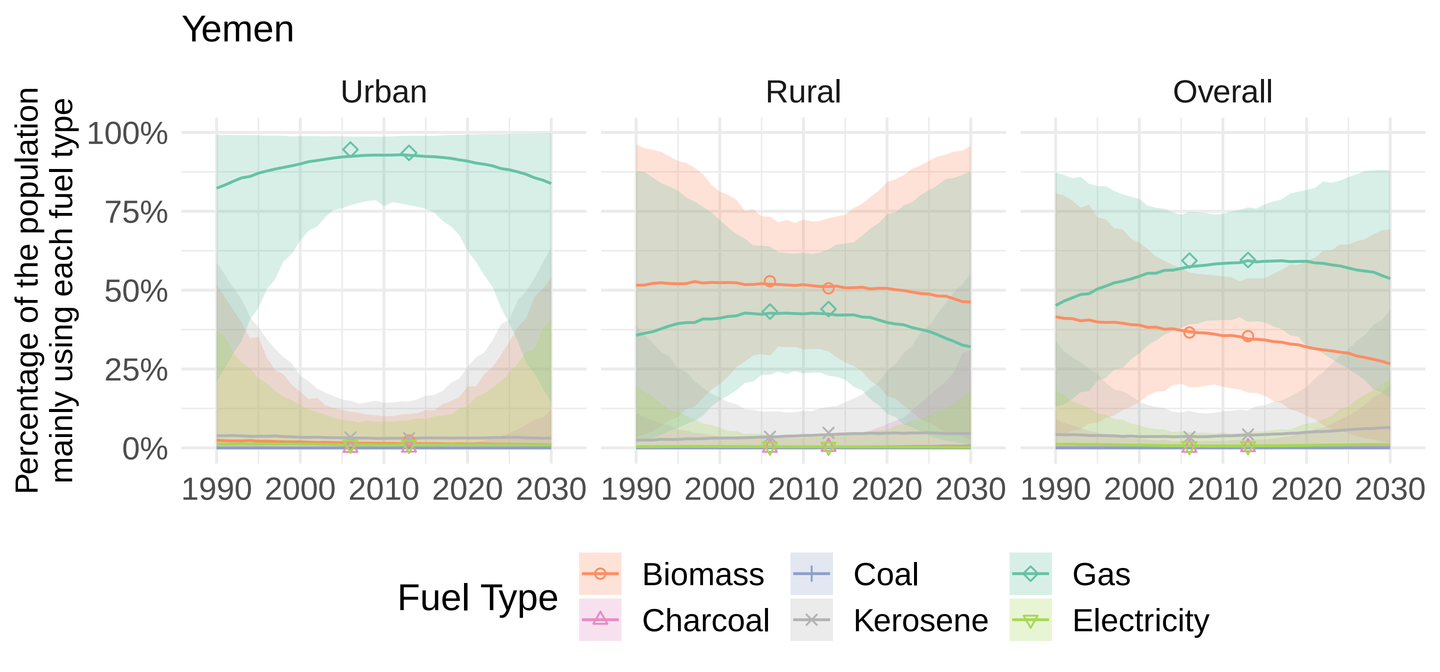

Yemen
Urban
Rural
Overall
Percentage of the population mainly using each fuel type
100%
75%
50%
25%
0%
1990
2000
2010
2020
2030
Fuel Type
Biomass
Charcoal
Coal
Kerosene
Gas
Electricity

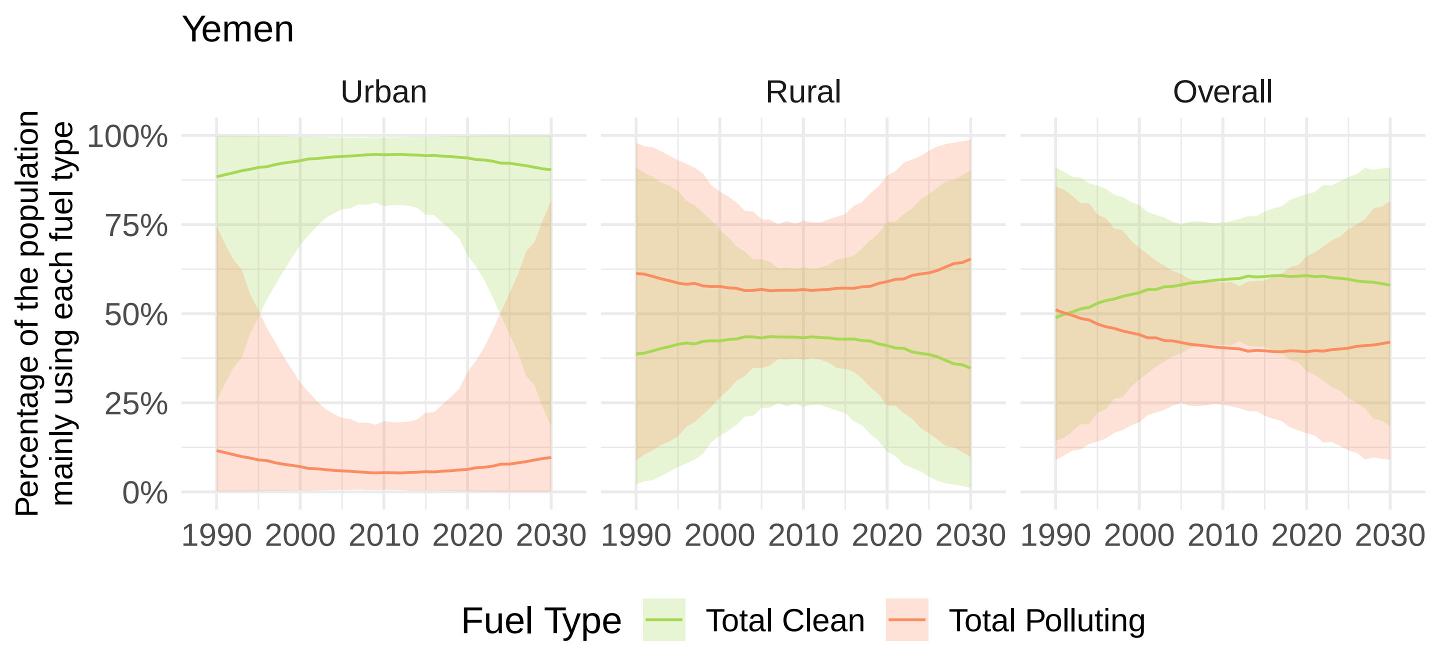
Yemen
Urban
Rural
Overall
Percentage of the population mainly using each fuel type
100%
75%
50%
25%
0%
1990
2000
2010
2020
2030
Fuel Type
Total Clean
Total Polluting

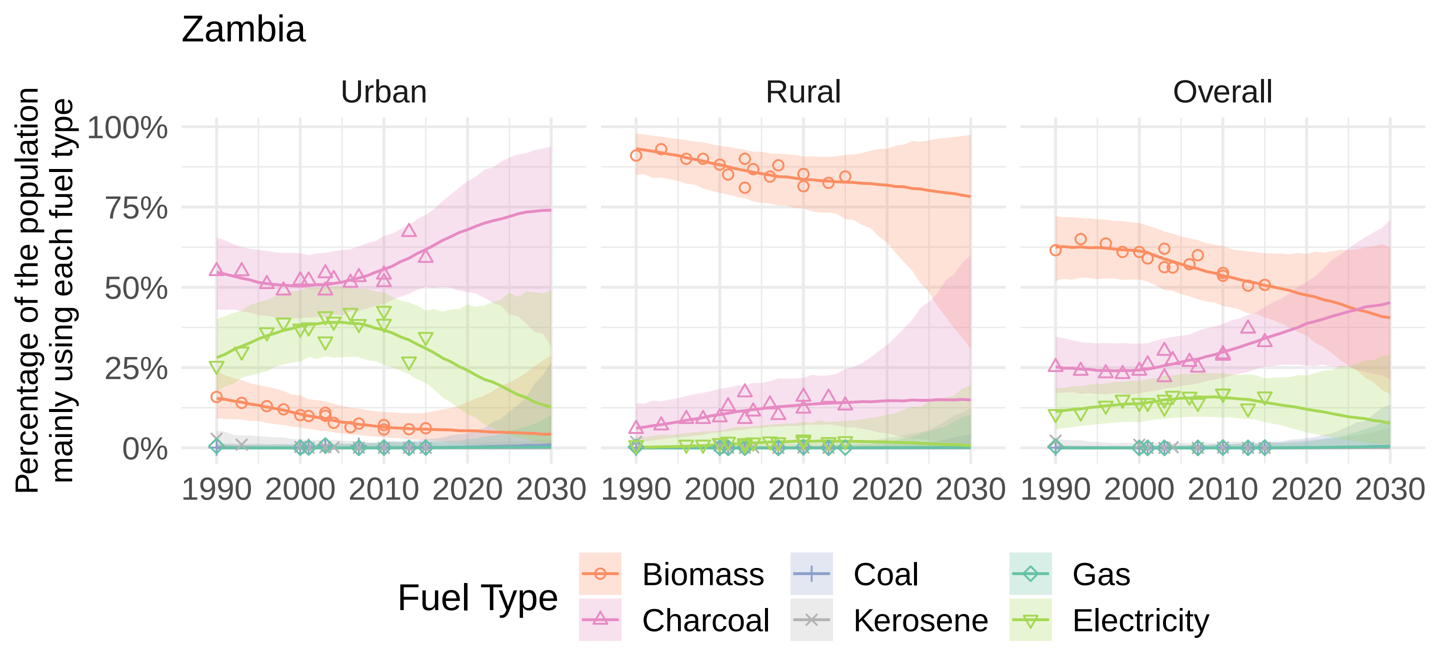
Zambia
Urban
Rural
Overall
Percentage of the population mainly using each fuel type
100%
75%
50%
25%
0%
1990
2000
2010
2020
2030
Fuel Type
Biomass
Charcoal
Coal
Kerosene
Gas
Electricity

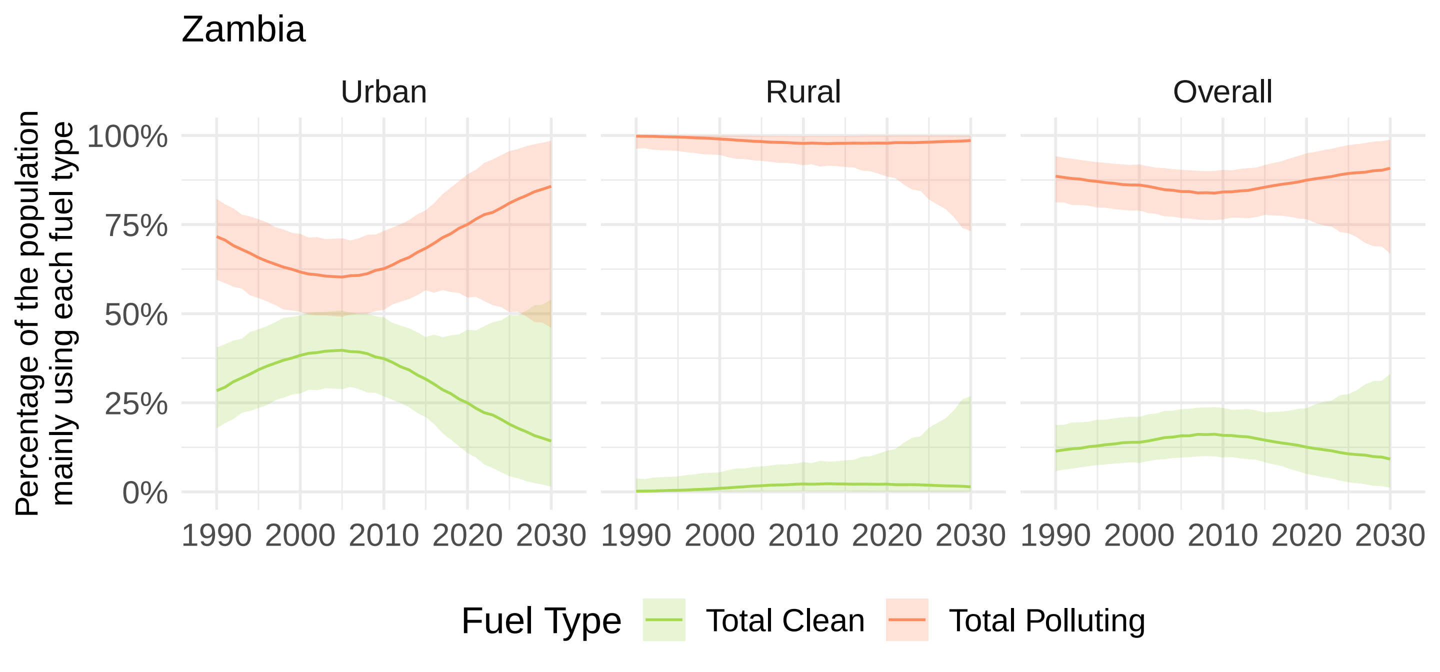

Zambia
Urban
Rural
Overall
Percentage of the population mainly using each fuel type
100%
75%
50%
25%
0%
1990
2000
2010
2020
2030
Fuel Type
Total Clean
Total Polluting

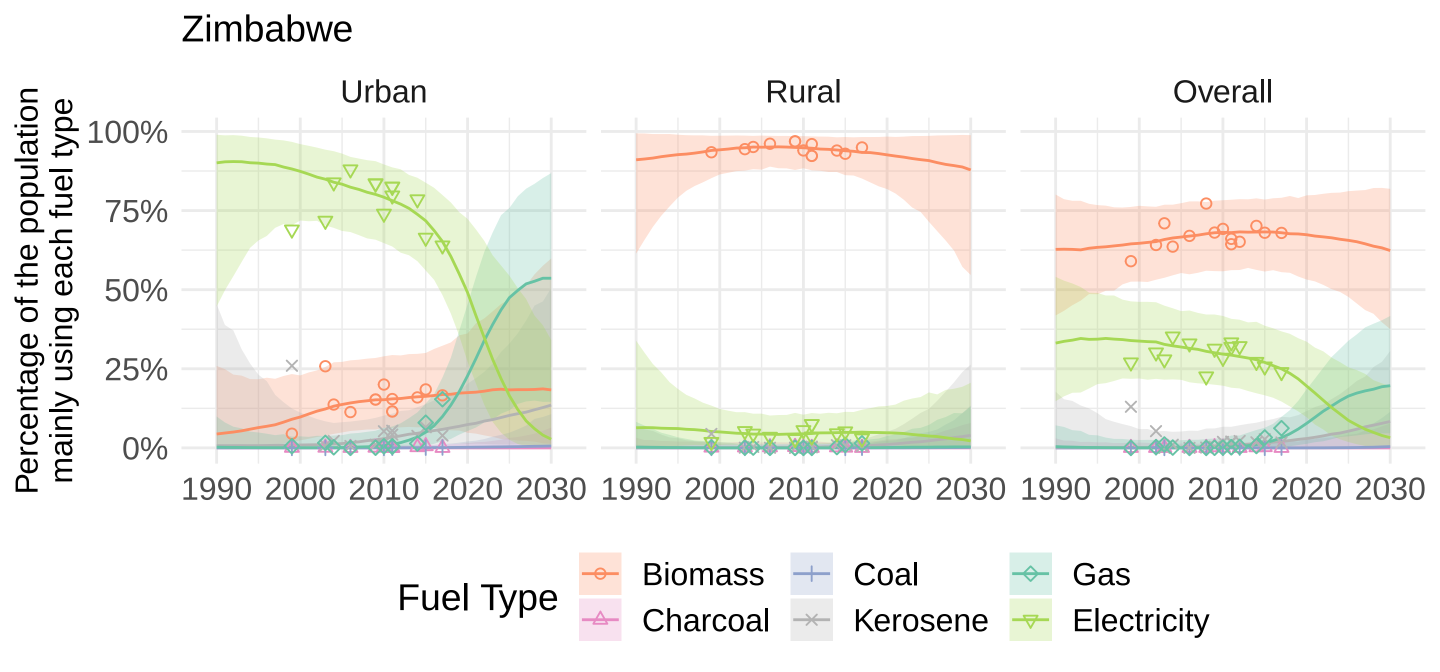
Zimbabwe
Urban
Rural
Overall
Percentage of the population mainly using each fuel type
100%
75%
50%
25%
0%
1990
2000
2010
2020
2030
Fuel Type
Biomass
Charcoal
Coal
Kerosene
Gas
Electricity

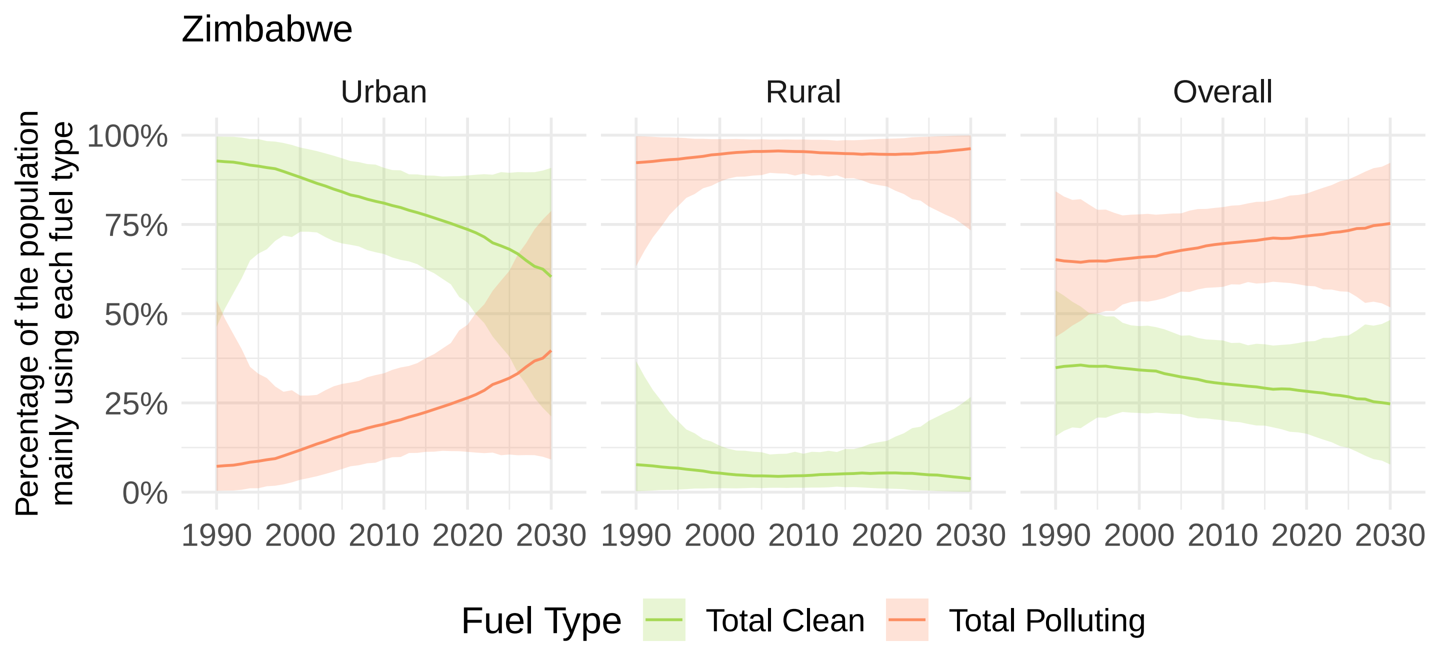
Zimbabwe
Urban
Rural
Overall
Percentage of the population mainly using each fuel type
100%
75%
50%
25%
0%
1990
2000
2010
2020
2030
Fuel Type
Total Clean
Total Polluting

B

SDG Region and Global Plots

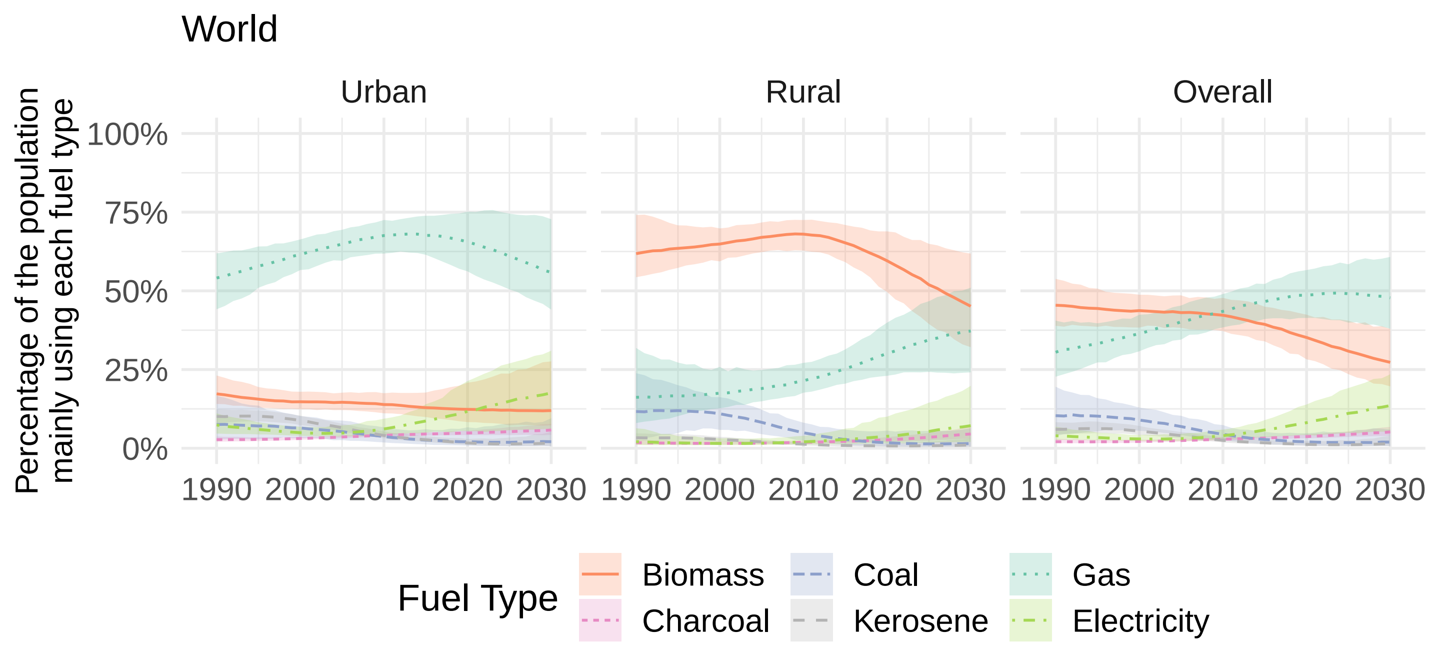

World
Urban
Rural
Overall
Percentage of the population mainly using each fuel type
100%
75%
50%
25%
0%
1990
2000
2010
2020
2030
Fuel Type
Biomass
Charcoal
Coal
Kerosene
Gas
Electricity

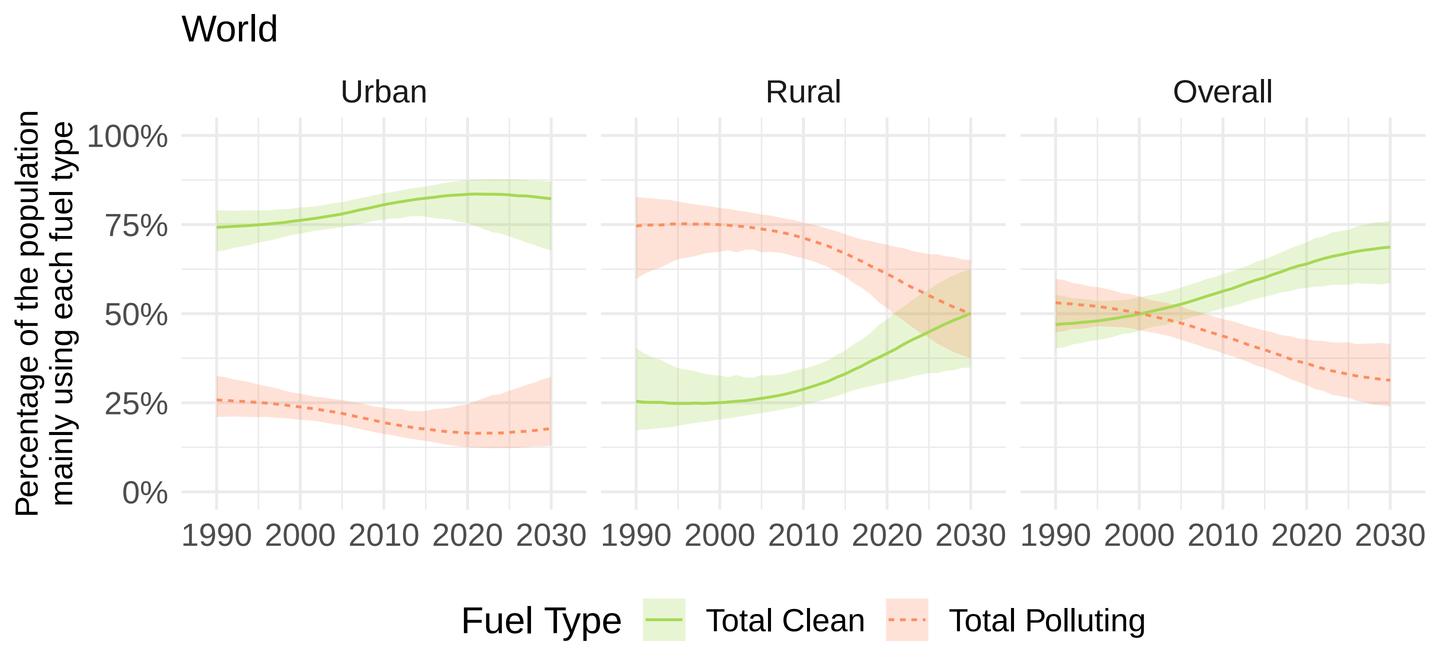

World
Urban
Rural
Overall
Percentage of the population mainly using each fuel type
100%
75%
50%
25%
0%
1990
2000
2010
2020
2030
Fuel Type
Total Clean
Total Polluting

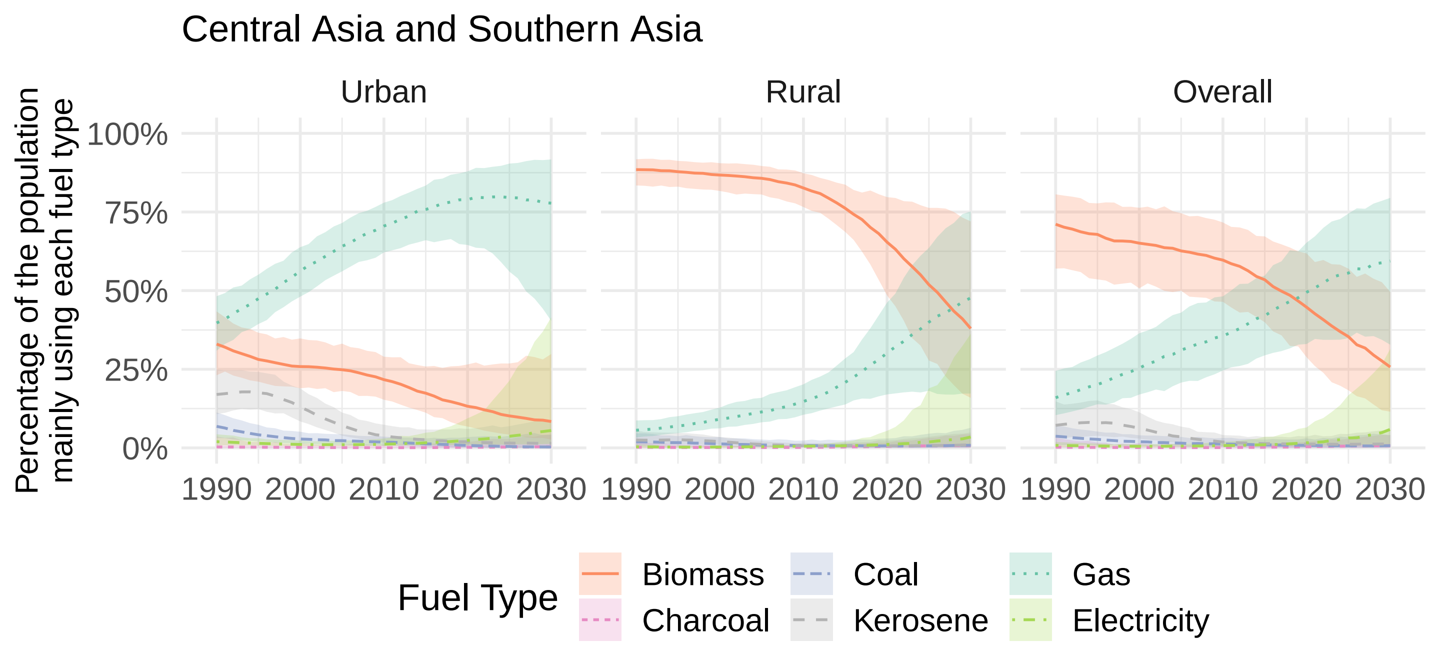

Central Asia and Southern Asia
Urban
Rural
Overall
Percentage of the population
mainly using each fuel type
100%
75%
50%
25%
0%
1990
2000
2010
2020
2030
Fuel Type
Biomass
Charcoal
Coal
Kerosene
Gas
Electricity

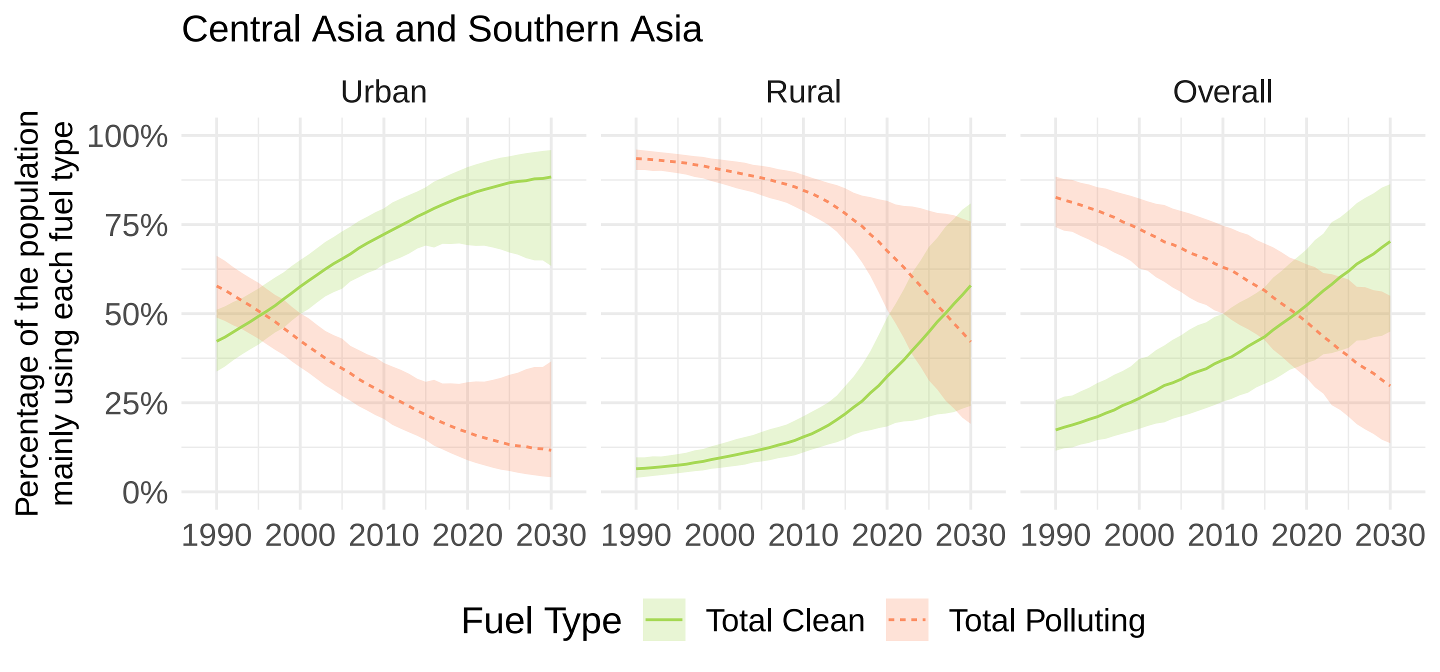

Central Asia and Southern Asia
Urban
Rural
Overall
Percentage of the population mainly using each fuel type
100%
75%
50%
25%
0%
1990
2000
2010
2020
2030
Fuel Type
Total Clean
Total Polluting

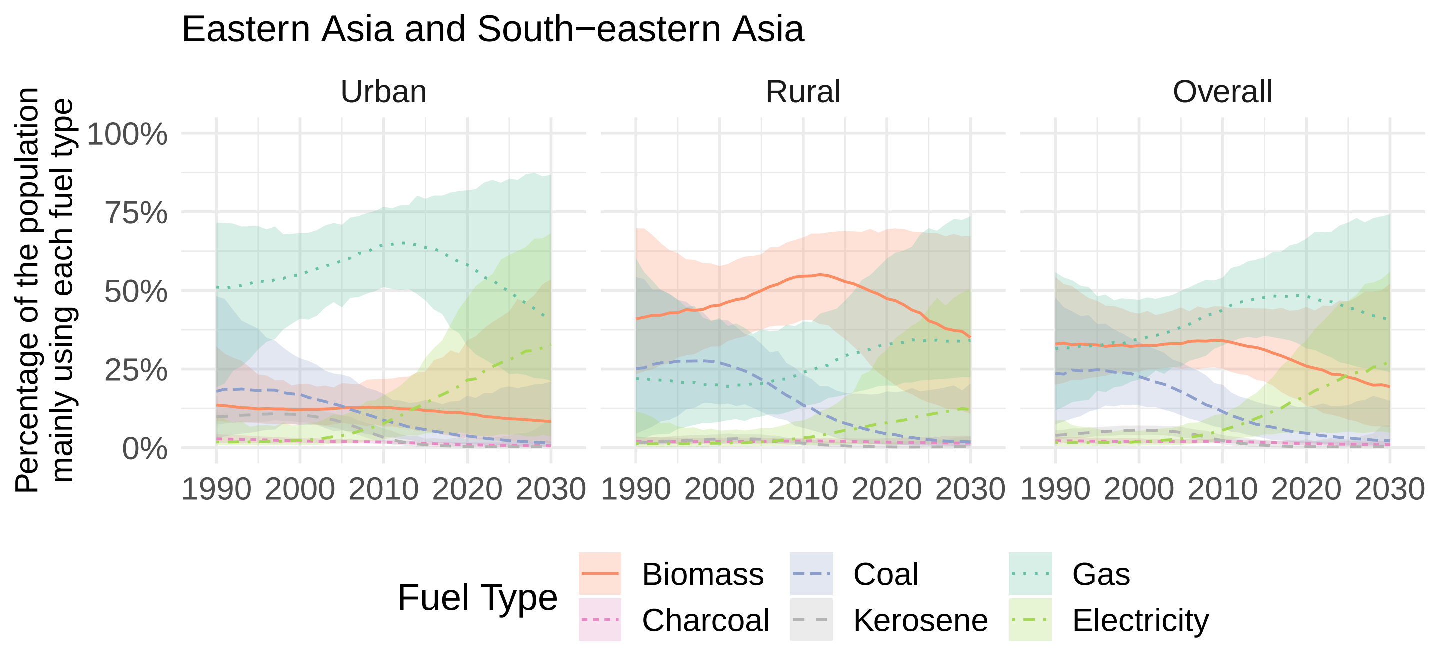

Eastern Asia and South−eastern Asia
Urban
Rural
Overall
Percentage of the population mainly using each fuel type
100%
75%
50%
25%
0%
1990
2000
2010
2020
2030
Fuel Type
Biomass
Charcoal
Coal
Kerosene
Gas
Electricity

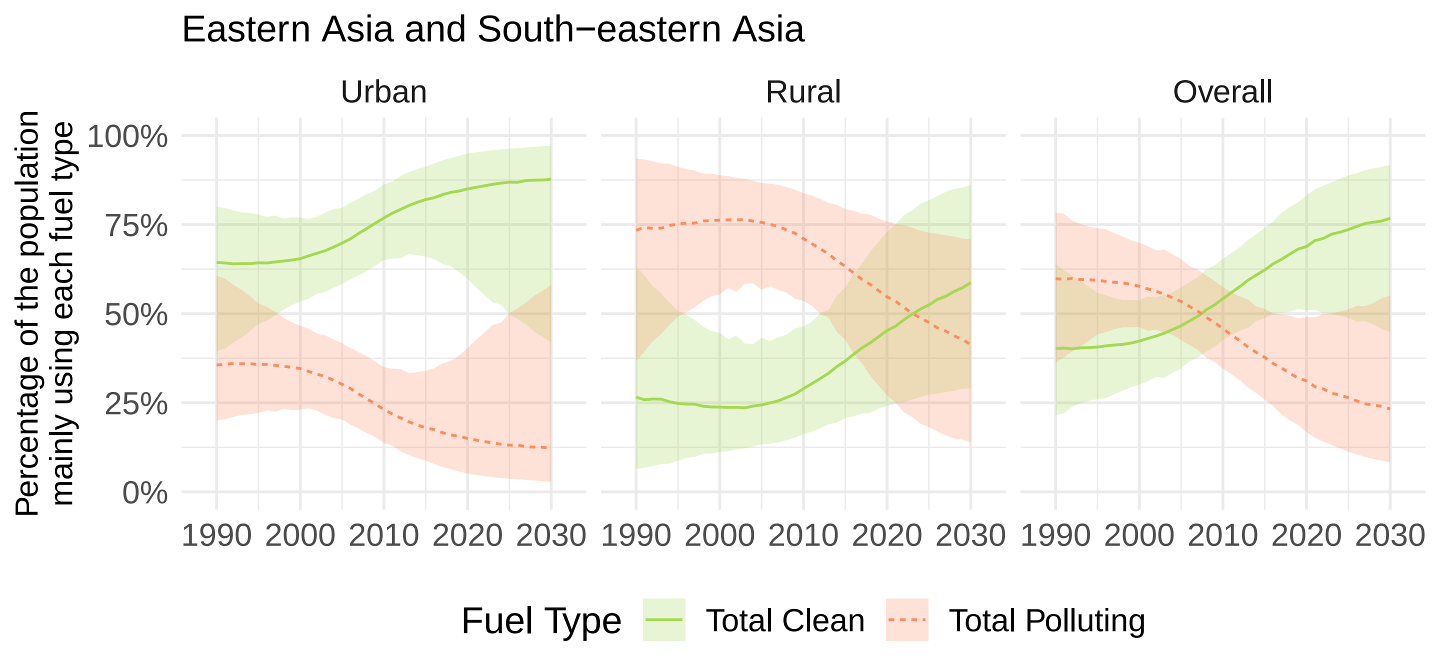

Eastern Asia and South−eastern Asia
Urban
Rural
Overall
Percentage of the population mainly using each fuel type
100%
75%
50%
25%
0%
1990
2000
2010
2020
2030
Fuel Type
Total Clean
Total Polluting

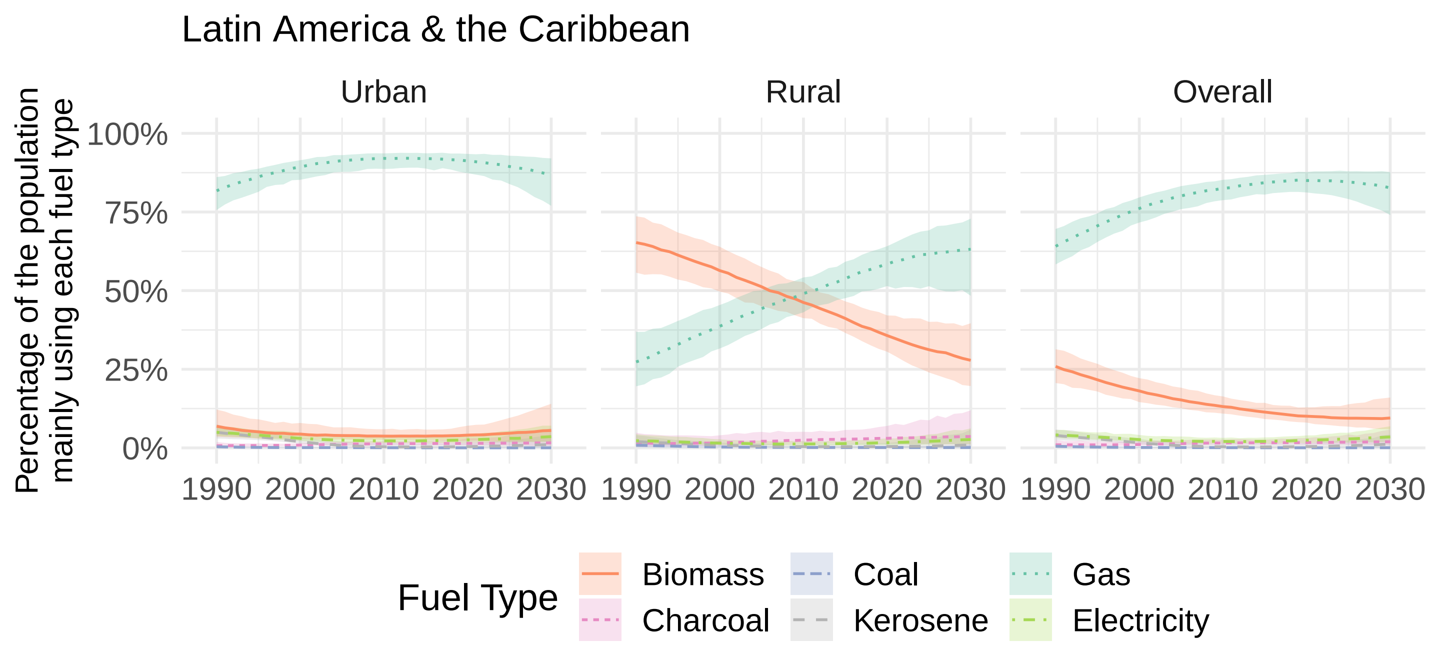
Latin America & the Caribbean
Urban
Rural
Overall
Percentage of the population mainly using each fuel type
100%
75%
50%
25%
0%
1990
2000
2010
2020
2030
Fuel Type
Biomass
Charcoal
Coal
Kerosene
Gas
Electricity

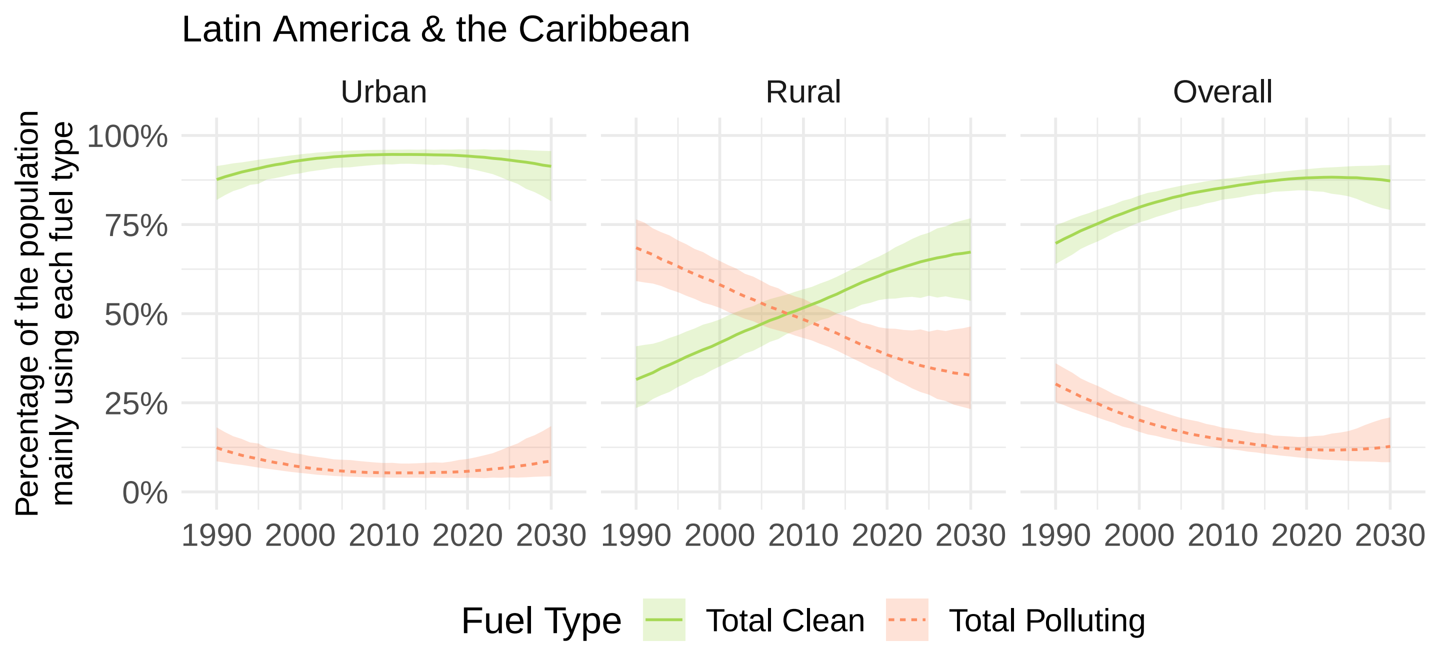

Latin America & the Caribbean
Urban
Rural
Overall
Percentage of the population mainly using each fuel type
100%
75%
50%
25%
0%
1990
2000
2010
2020
2030
Fuel Type
Total Clean
Total Polluting

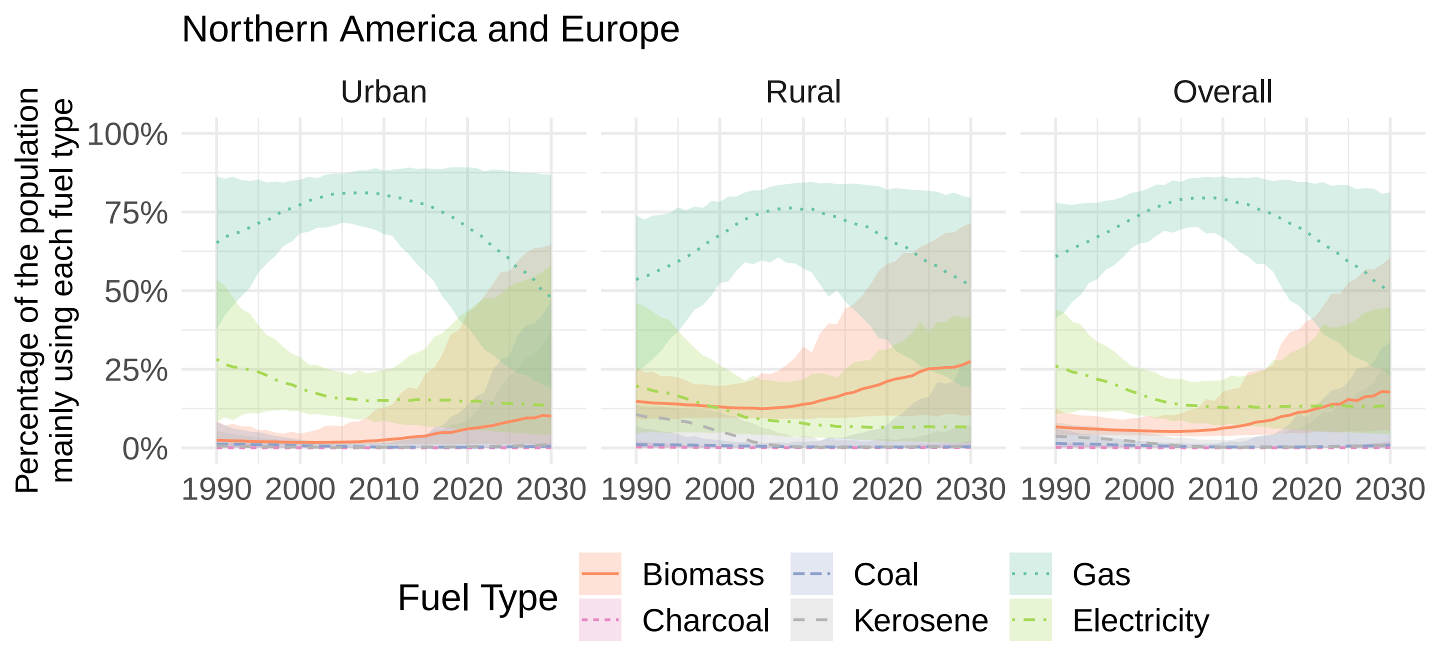
Northern America and Europe
Urban
Rural
Overall
Percentage of the population mainly using each fuel type
100%
75%
50%
25%
0%
1990
2000
2010
2020
2030
Fuel Type
Biomass
Charcoal
Coal
Kerosene
Gas
Electricity

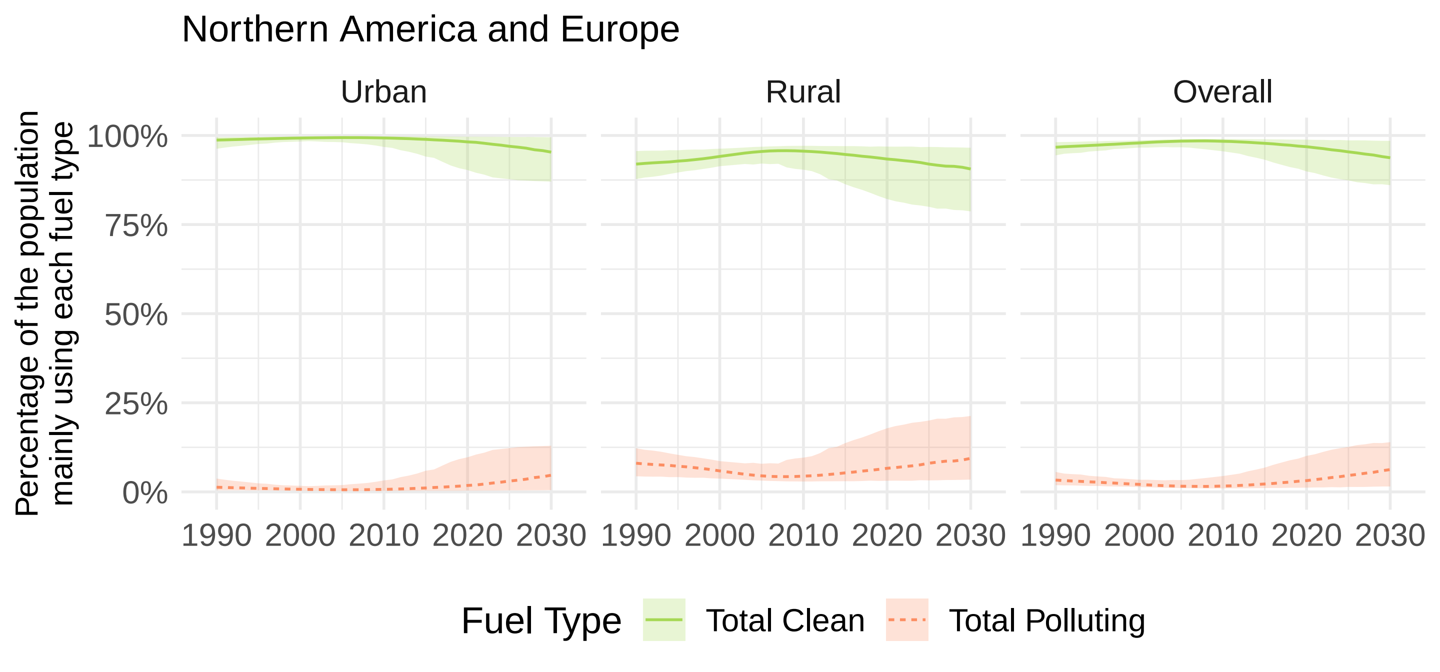
Northern America and Europe
Urban
Rural
Overall
Percentage of the population mainly using each fuel type
100%
75%
50%
25%
0%
1990
2000
2010
2020
2030
Fuel Type
Total Clean
Total Polluting

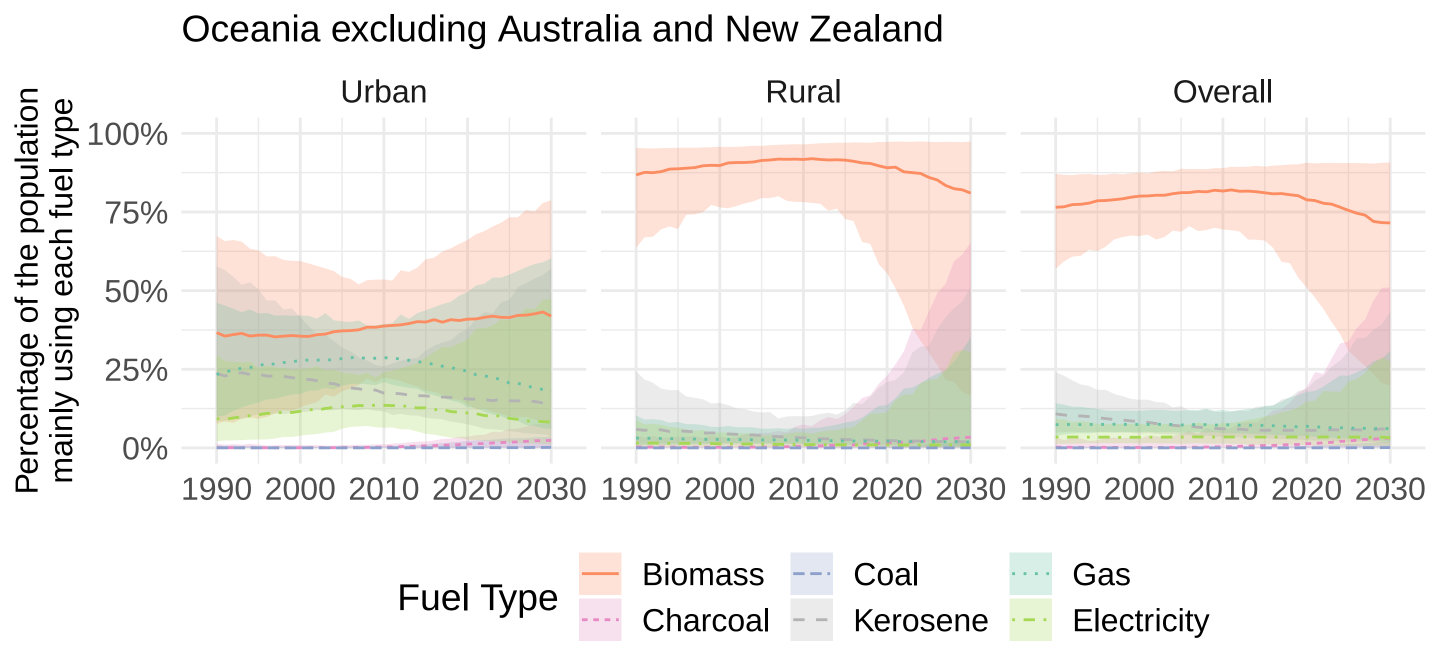

Oceania excluding Australia and New Zealand
Urban
Rural
Overall
Percentage of the population mainly using each fuel type
100%
75%
50%
25%
0%
1990
2000
2010
2020
2030
Fuel Type
Biomass
Charcoal
Coal
Kerosene
Gas
Electricity

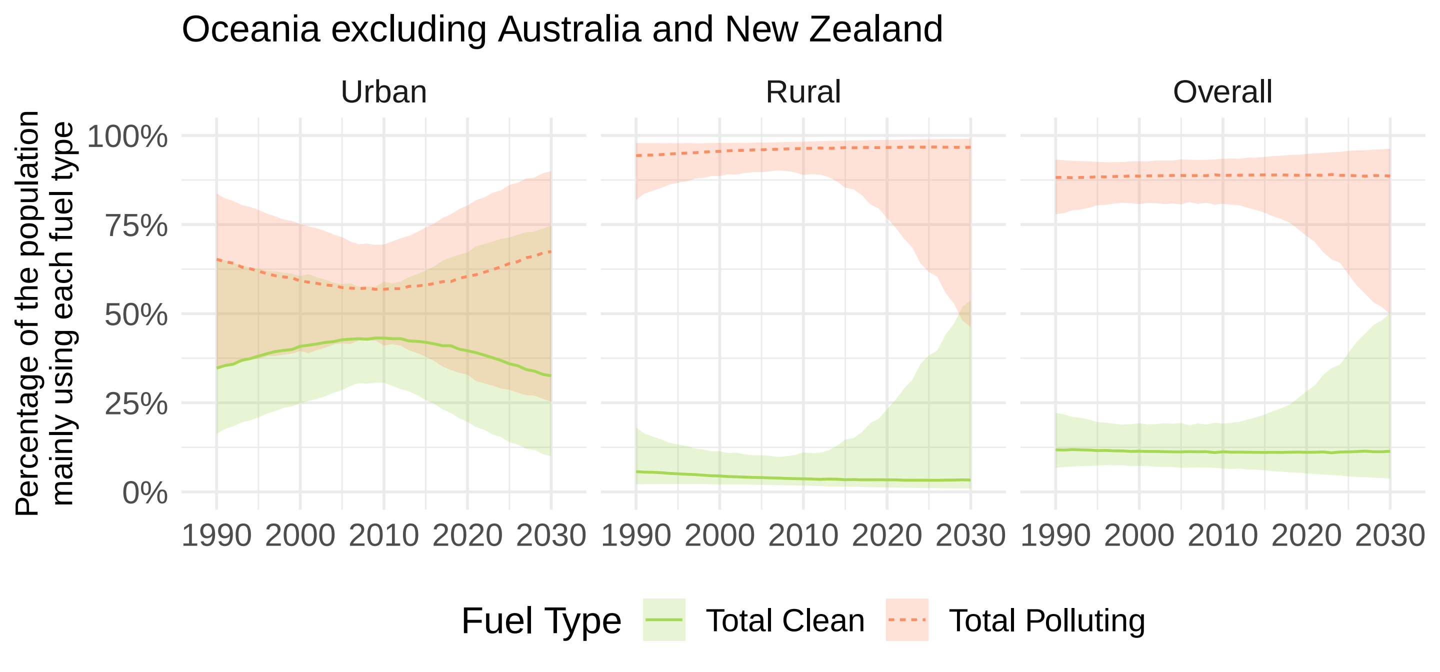

Oceania excluding Australia and New Zealand
Urban
Rural
Overall
Percentage of the population mainly using each fuel type
100%
75%
50%
25%
0%
1990
2000
2010
2020
2030
Fuel Type
Total Clean
Total Polluting

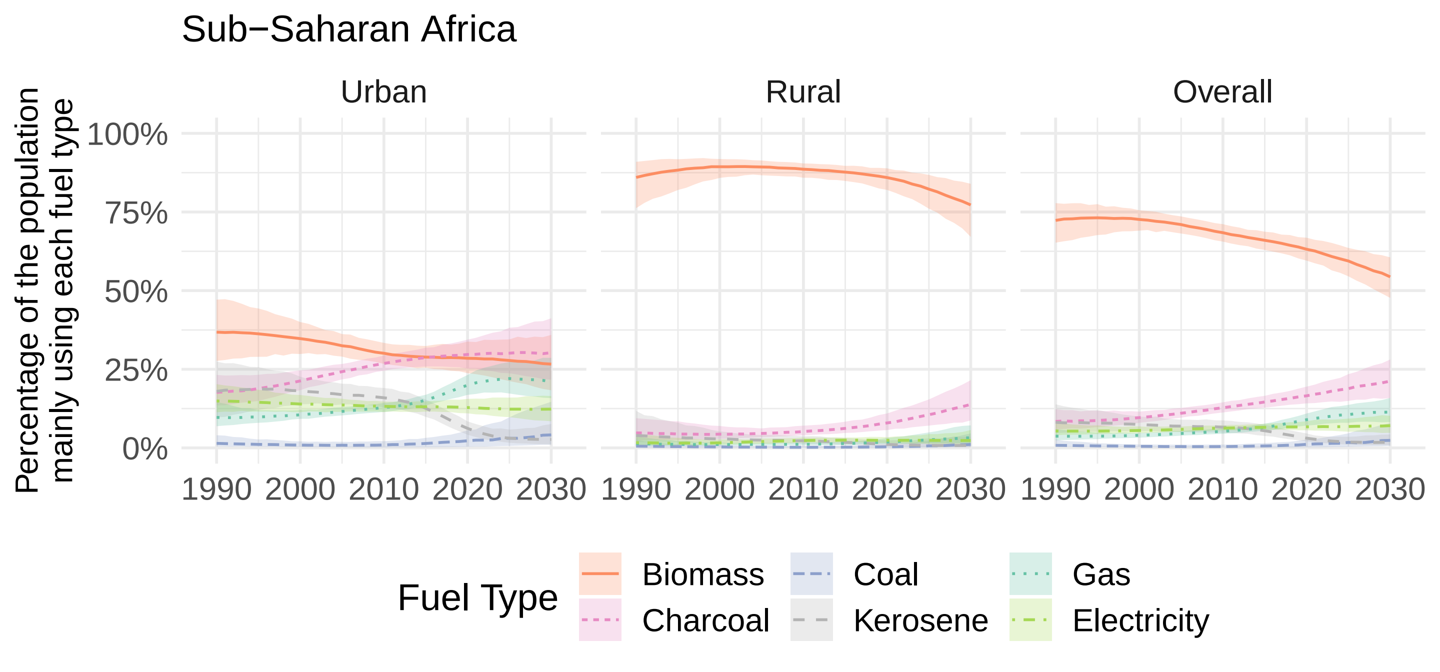

Sub−Saharan Africa
Urban
Rural
Overall
Percentage of the population mainly using each fuel type
100%
75%
50%
25%
0%
1990
2000
2010
2020
2030
Fuel Type
Biomass
Charcoal
Coal
Kerosene
Gas
Electricity

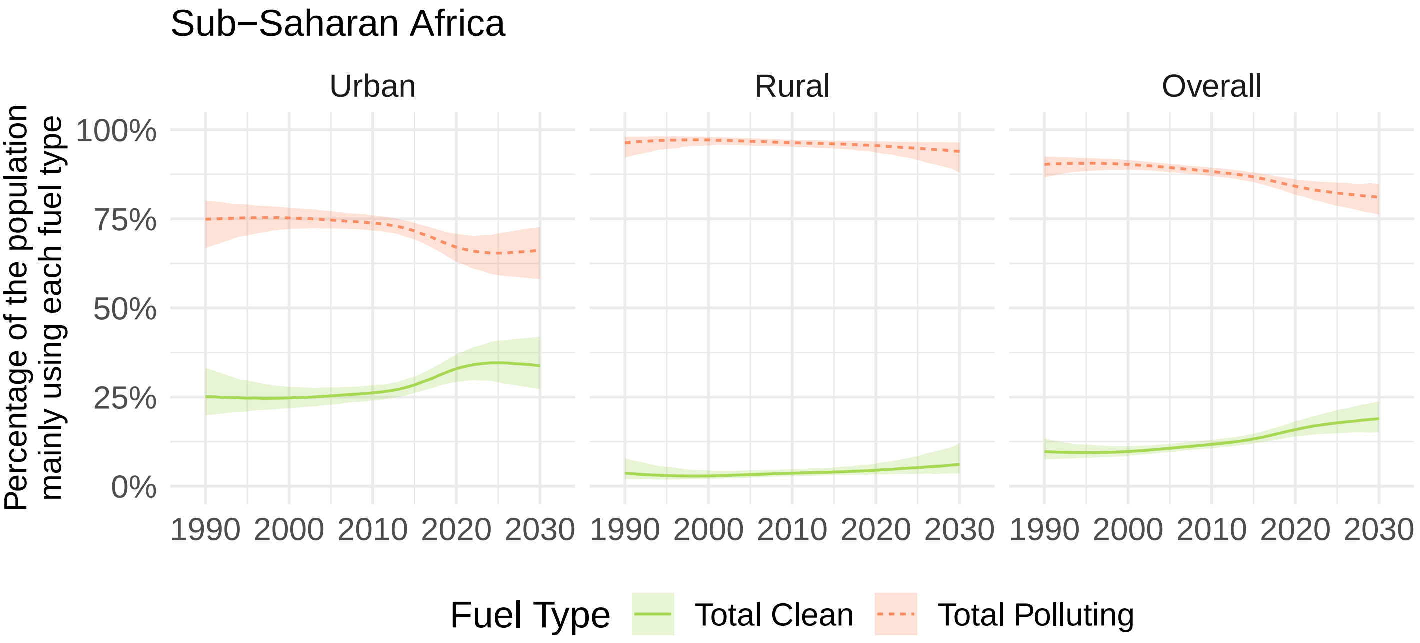

Sub−Saharan Africa
Urban
Rural
Overall
Percentage of the population mainly using each fuel type
100%
75%
50%
25%
0%
1990
2000
2010
2020
2030
Fuel Type
Total Clean
Total Polluting

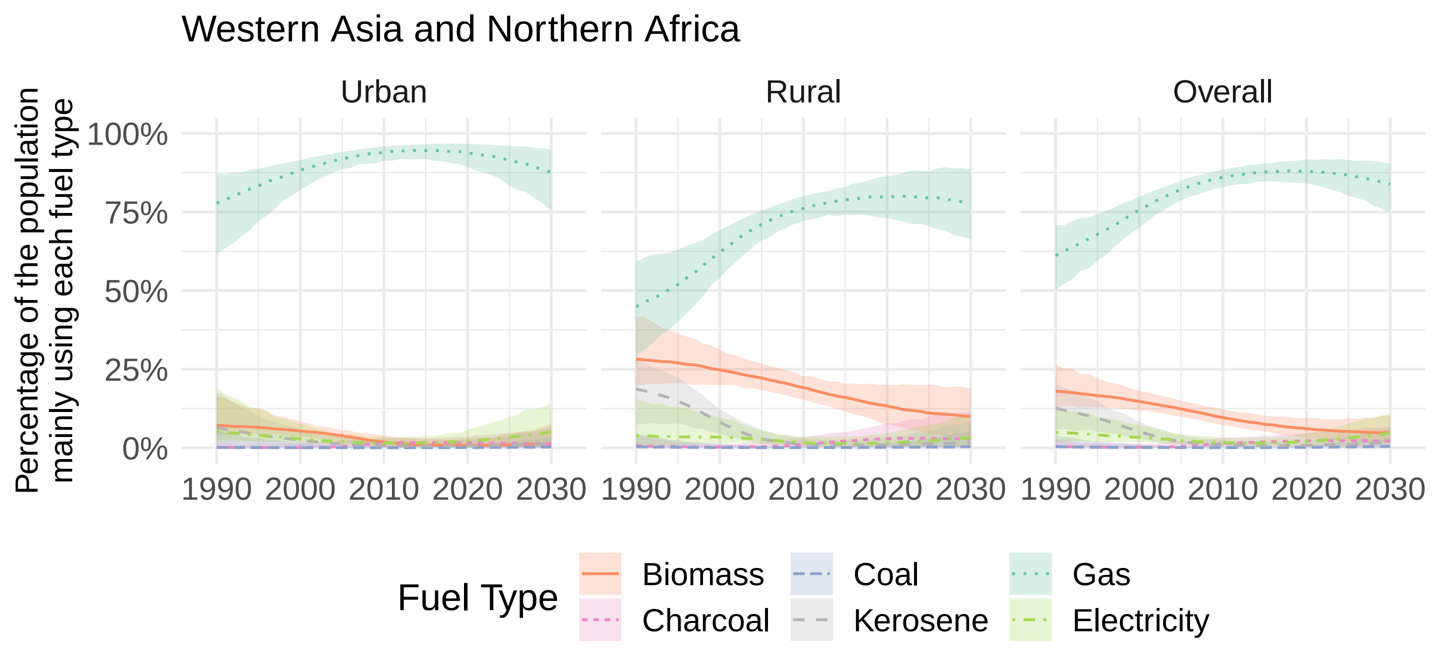

Western Asia and Northern Africa
Urban
Rural
Overall
Percentage of the population mainly using each fuel type
100%
75%
50%
25%
0%
1990
2000
2010
2020
2030
Fuel Type
Biomass
Charcoal
Coal
Kerosene
Gas
Electricity

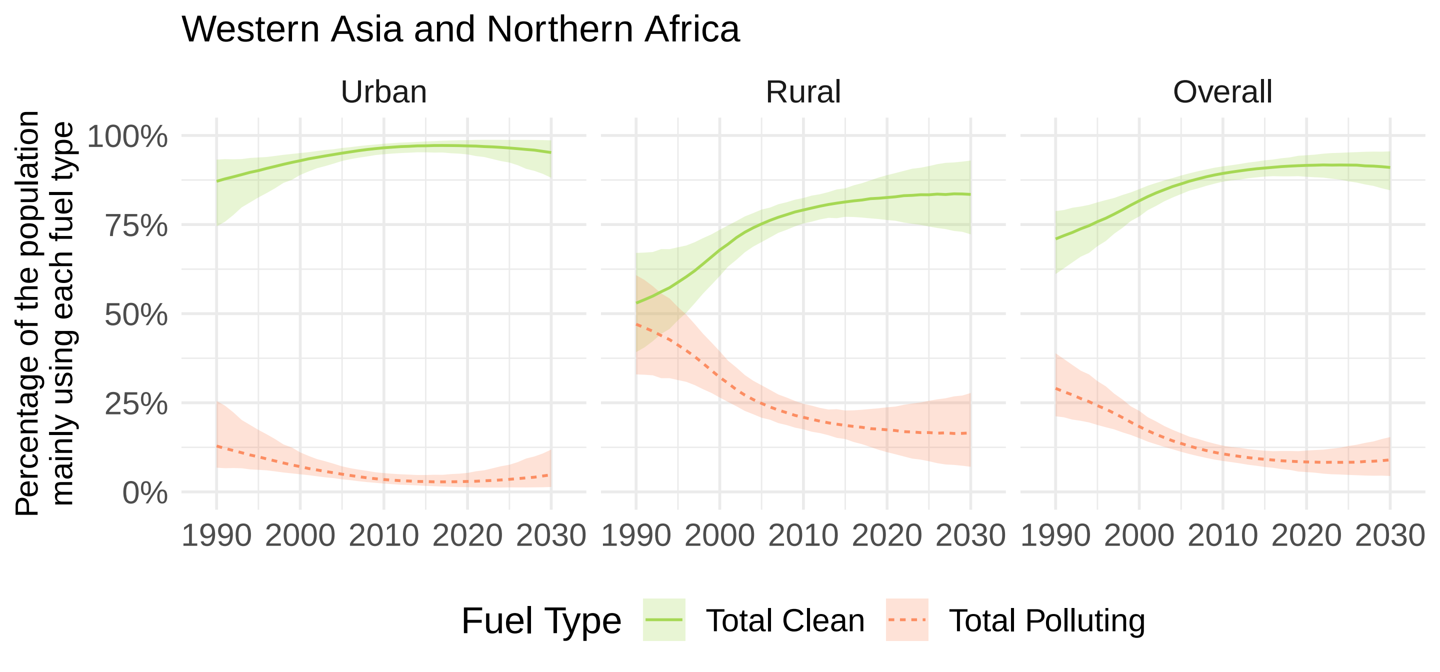
Western Asia and Northern Africa
Urban
Rural
Overall
Percentage of the population mainly using each fuel type
100%
75%
50%
25%
0%
1990
2000
2010
2020
2030
Fuel Type
Total Clean
Total Polluting

C

WHO Region Plots

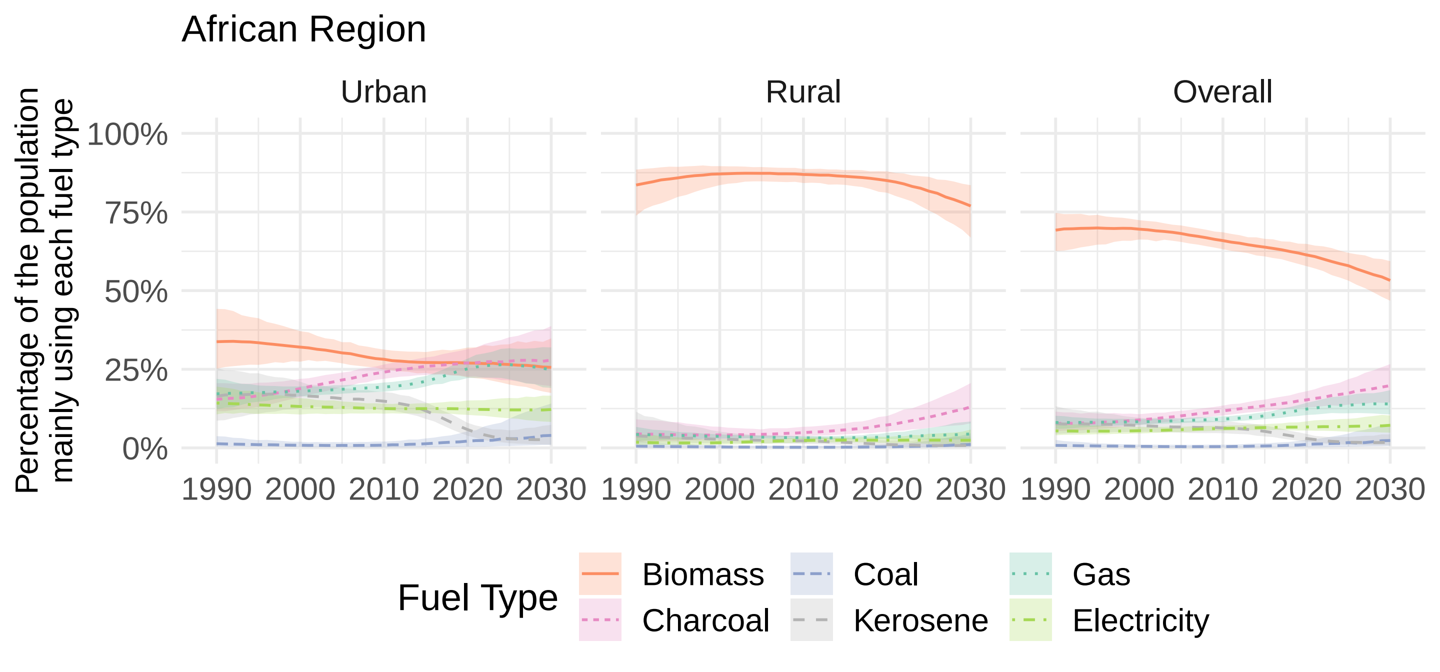
African Region
Urban
Rural
Overall
Percentage of the population mainly using each fuel type
100%
75%
50%
25%
0%
1990
2000
2010
2020
2030
Fuel Type
Biomass
Charcoal
Coal
Kerosene
Gas
Electricity

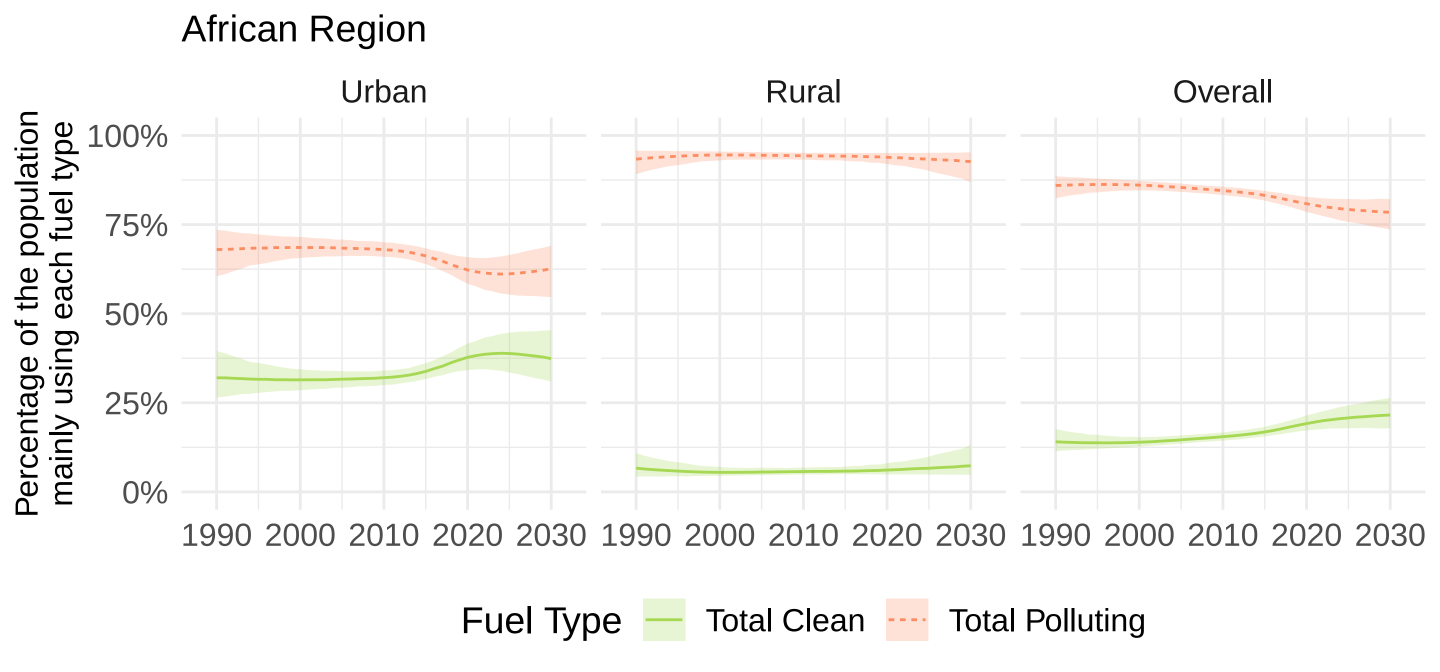

African Region
Urban
Rural
Overall
Percentage of the population
mainly using each fuel type
100%
75%
50%
25%
0%
1990
2000
2010
2020
2030
Fuel Type
Total Clean
Total Polluting

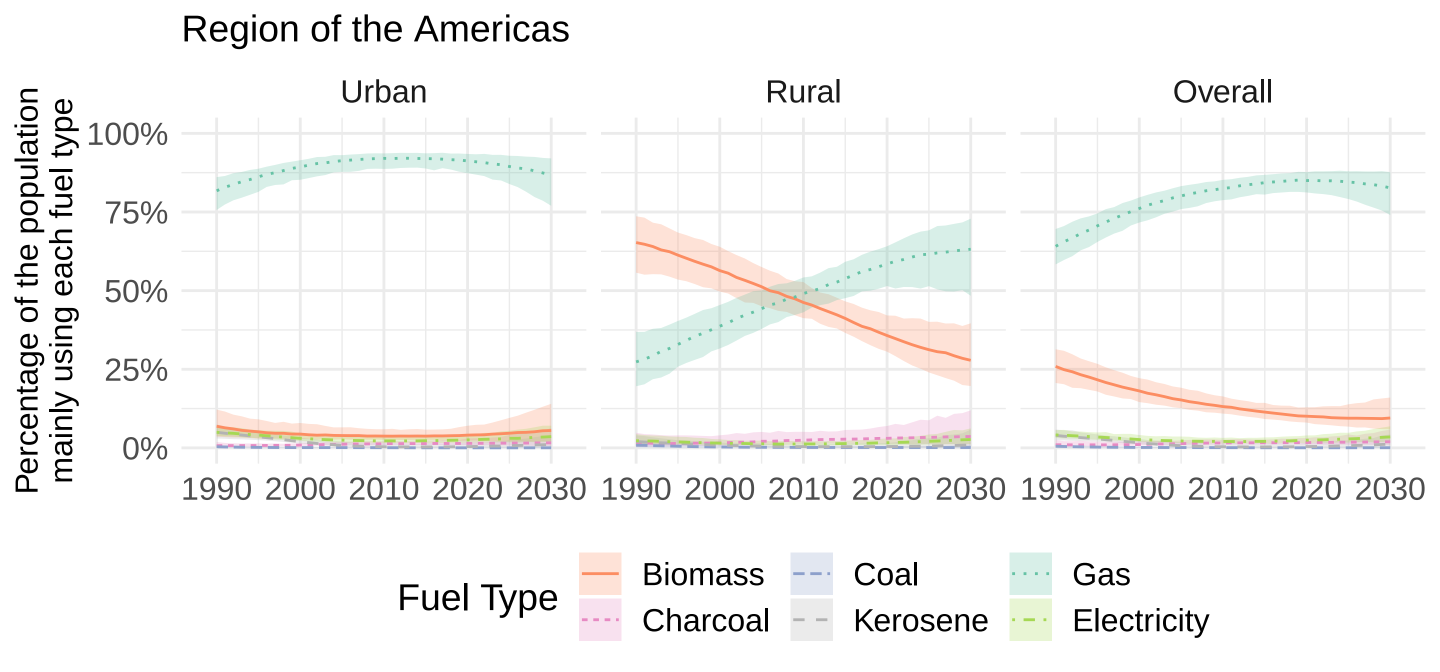
Region of the Americas
Urban
Rural
Overall
Percentage of the population mainly using each fuel type
100%
75%
50%
25%
0%
1990
2000
2010
2020
2030
Fuel Type
Biomass
Charcoal
Coal
Kerosene
Gas
Electricity

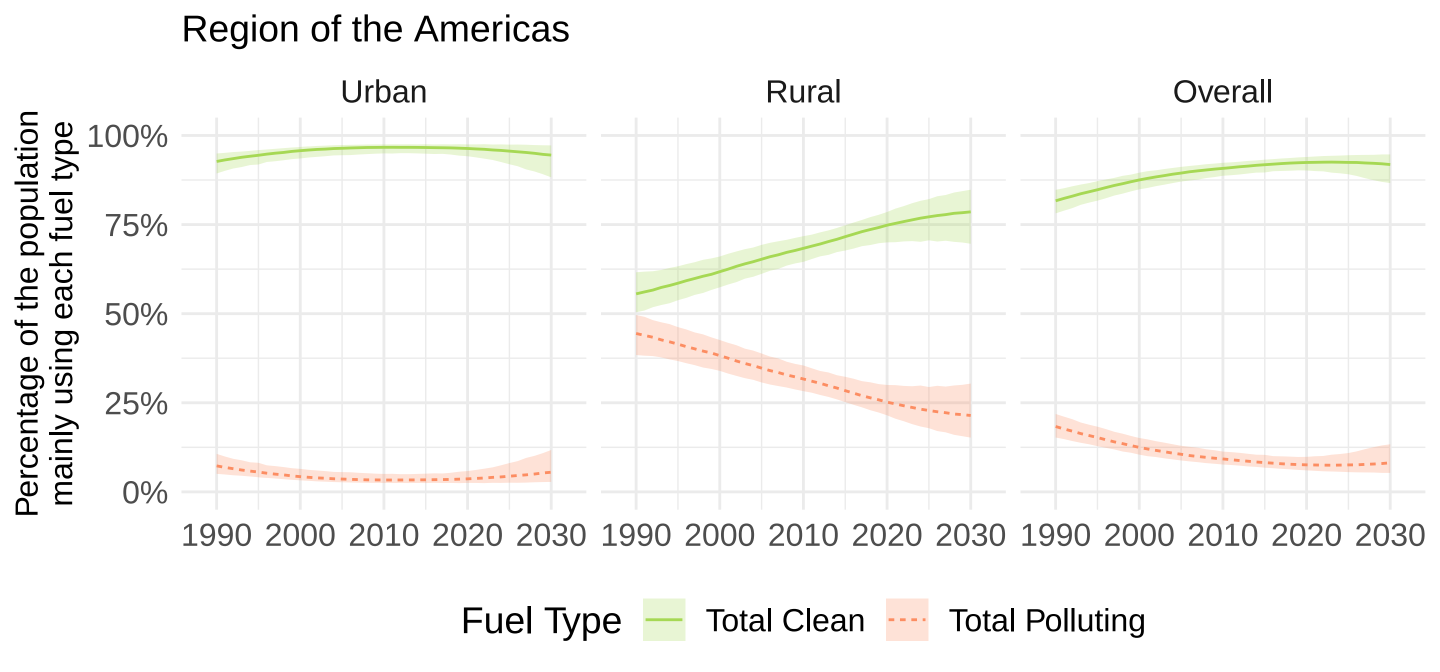

Region of the Americas
Urban
Rural
Overall
Percentage of the population mainly using each fuel type
100%
75%
50%
25%
0%
1990
2000
2010
2020
2030
Fuel Type
Total Clean
Total Polluting

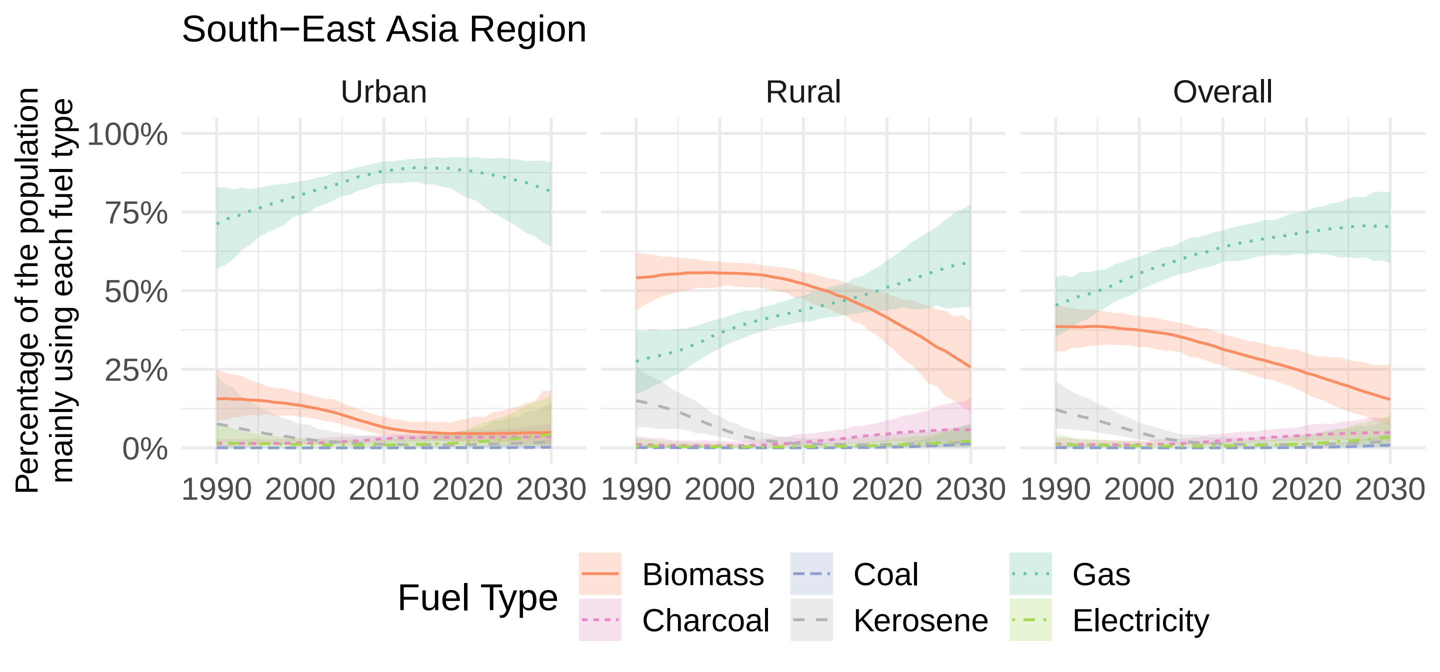

South−East Asia Region
Urban
Rural
Overall
Percentage of the population mainly using each fuel type
100%
75%
50%
25%
0%
1990
2000
2010
2020
2030
Fuel Type
Biomass
Charcoal
Coal
Kerosene
Gas
Electricity

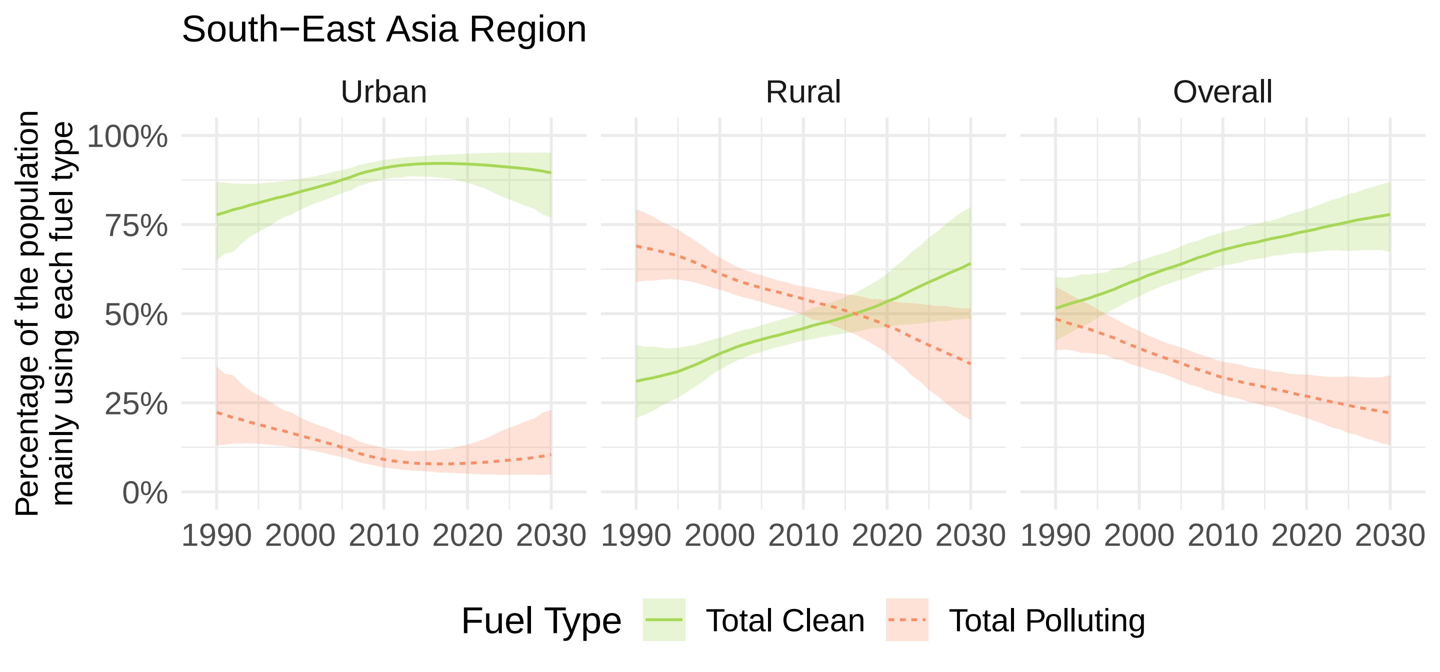
South−East Asia Region
Urban
Rural
Overall
Percentage of the population mainly using each fuel type
100%
75%
50%
25%
0%
1990
2000
2010
2020
2030
Fuel Type
Total Clean
Total Polluting

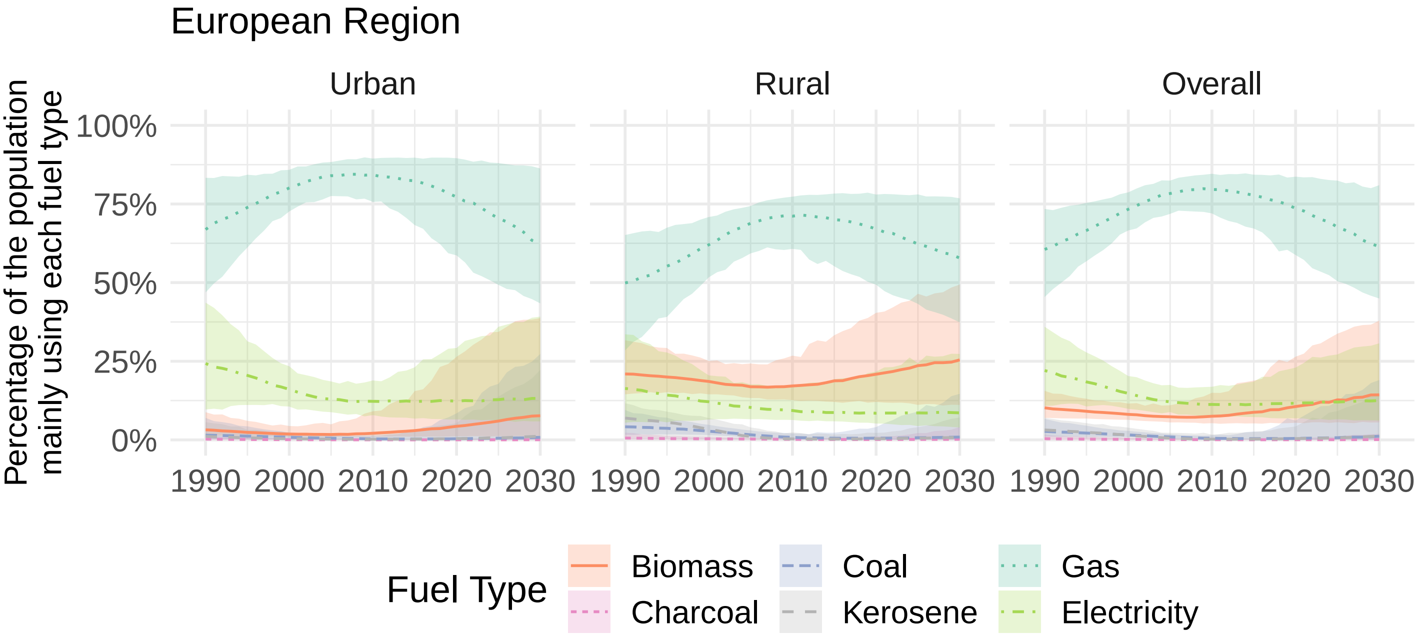

European Region
Urban
Rural
Overall
Percentage of the population mainly using each fuel type
100%
75%
50%
25%
0%
1990
2000
2010
2020
2030
Fuel Type
Biomass
Charcoal
Coal
Kerosene
Gas
Electricity

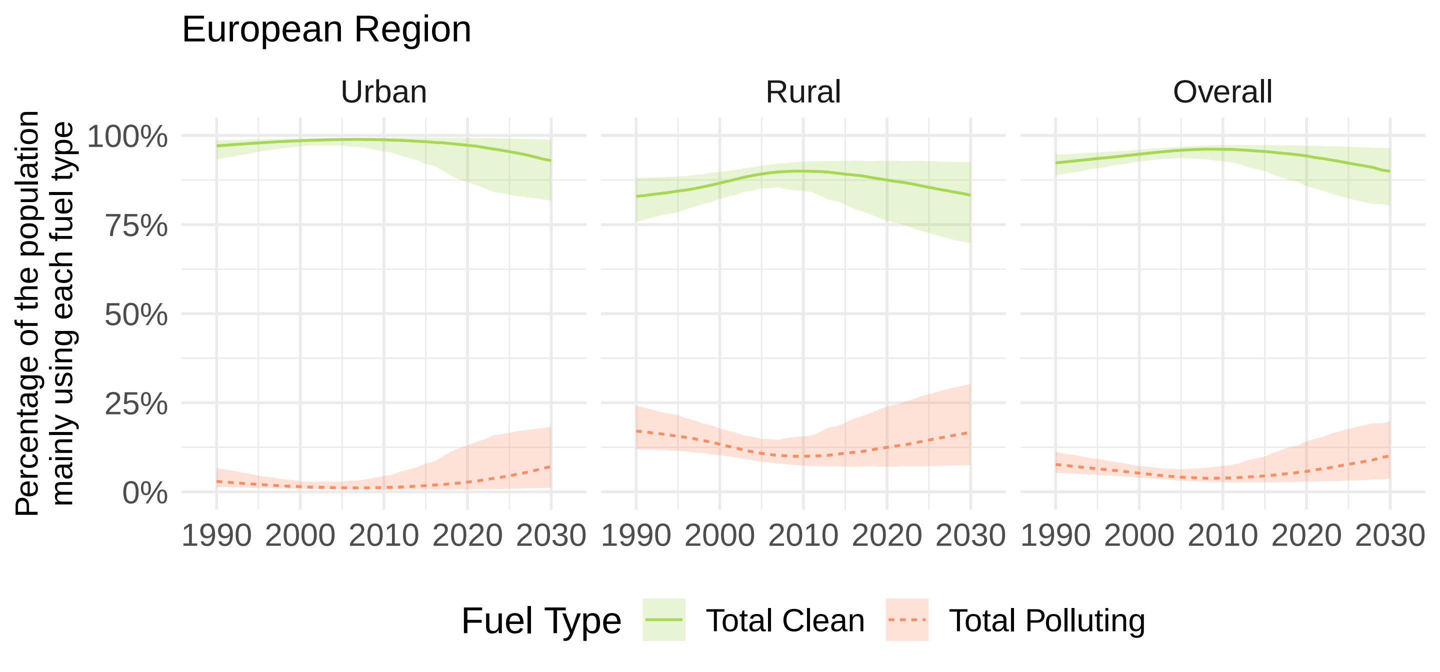
European Region
Urban
Rural
Overall
Percentage of the population mainly using each fuel type
100%
75%
50%
25%
0%
1990
2000
2010
2020
2030
Fuel Type
Total Clean
Total Polluting

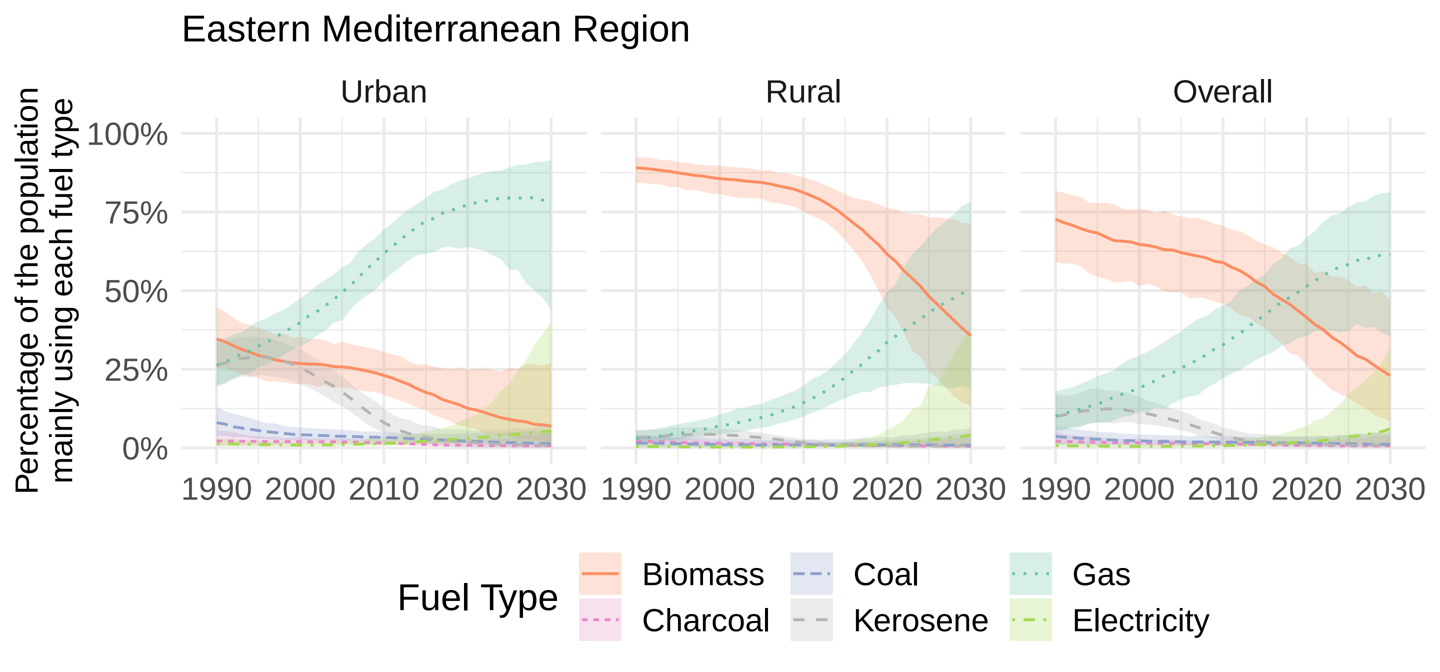

Eastern Mediterranean Region
Urban
Rural
Overall
Percentage of the population mainly using each fuel type
100%
75%
50%
25%
0%
1990
2000
2010
2020
2030
Fuel Type
Biomass
Charcoal
Coal
Kerosene
Gas
Electricity

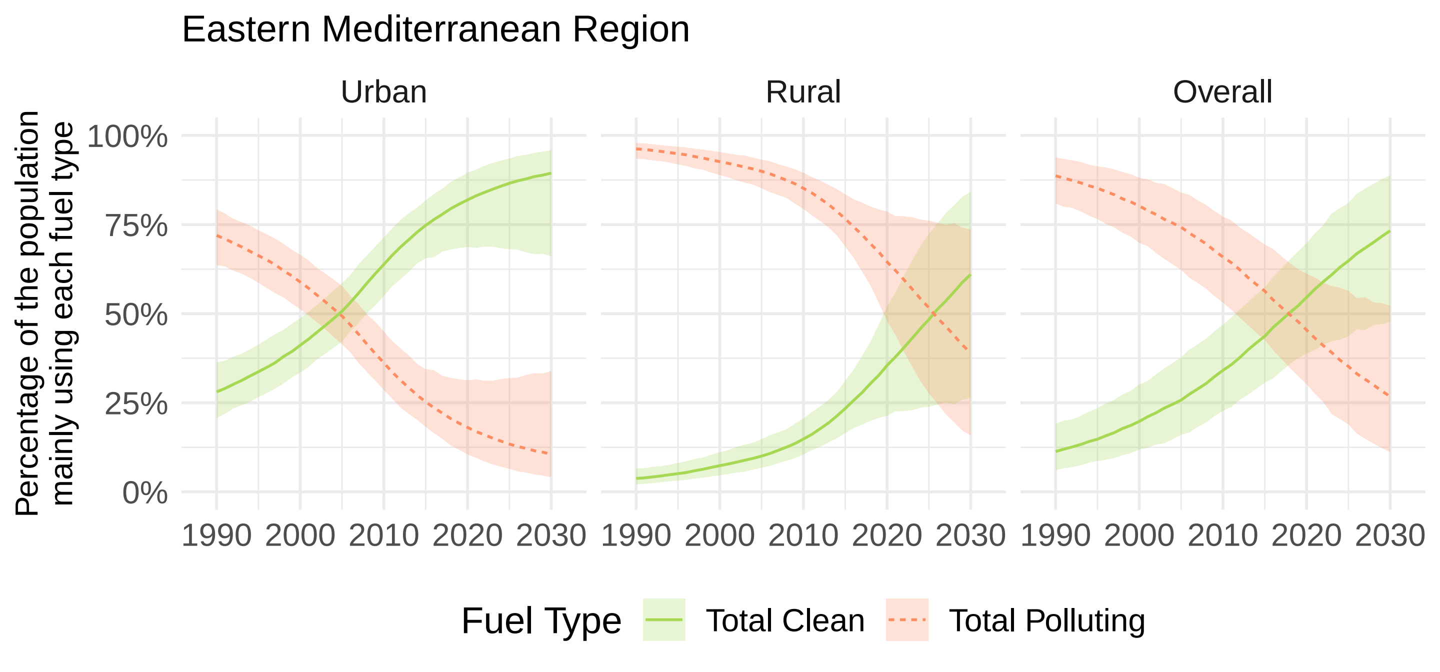
Eastern Mediterranean Region
Urban
Rural
Overall
Percentage of the population mainly using each fuel type
100%
75%
50%
25%
0%
1990
2000
2010
2020
2030
Fuel Type
Total Clean
Total Polluting

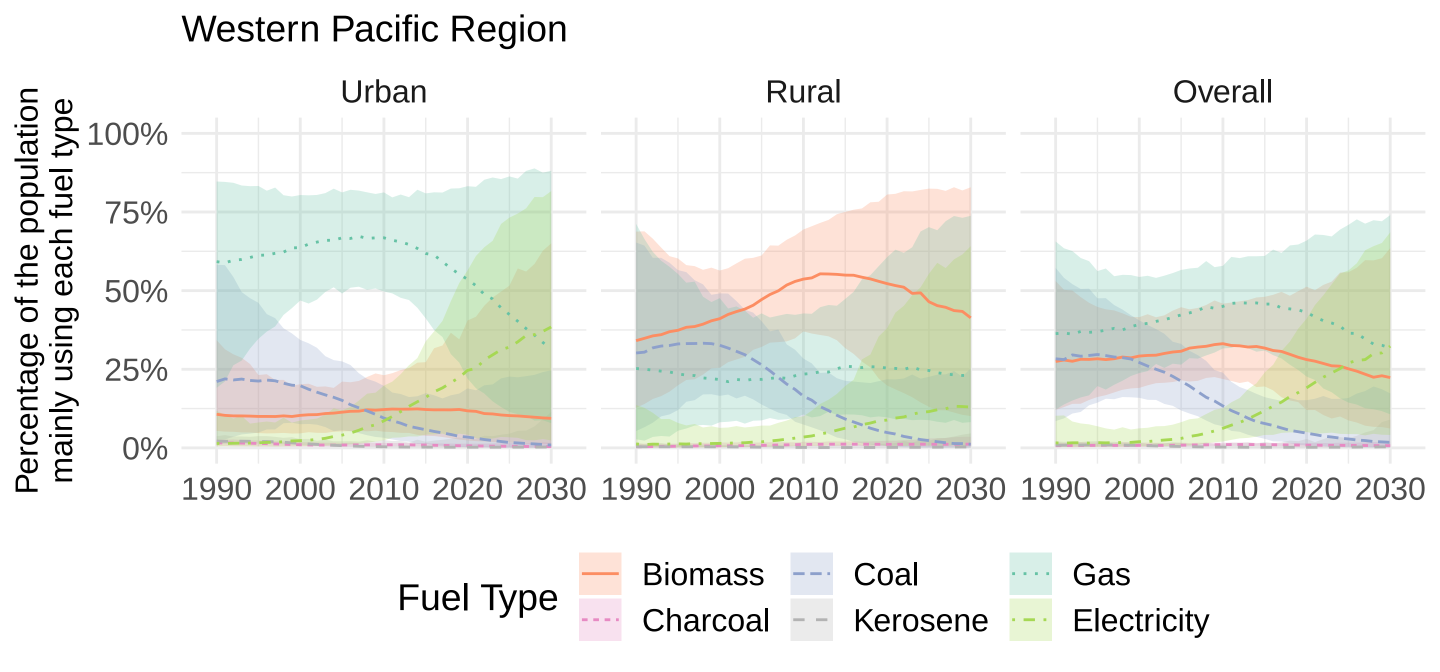
Western Pacific Region
Urban
Rural
Overall
Percentage of the population mainly using each fuel type
100%
75%
50%
25%
0%
1990
2000
2010
2020
2030
Fuel Type
Biomass
Charcoal
Coal
Kerosene
Gas
Electricity

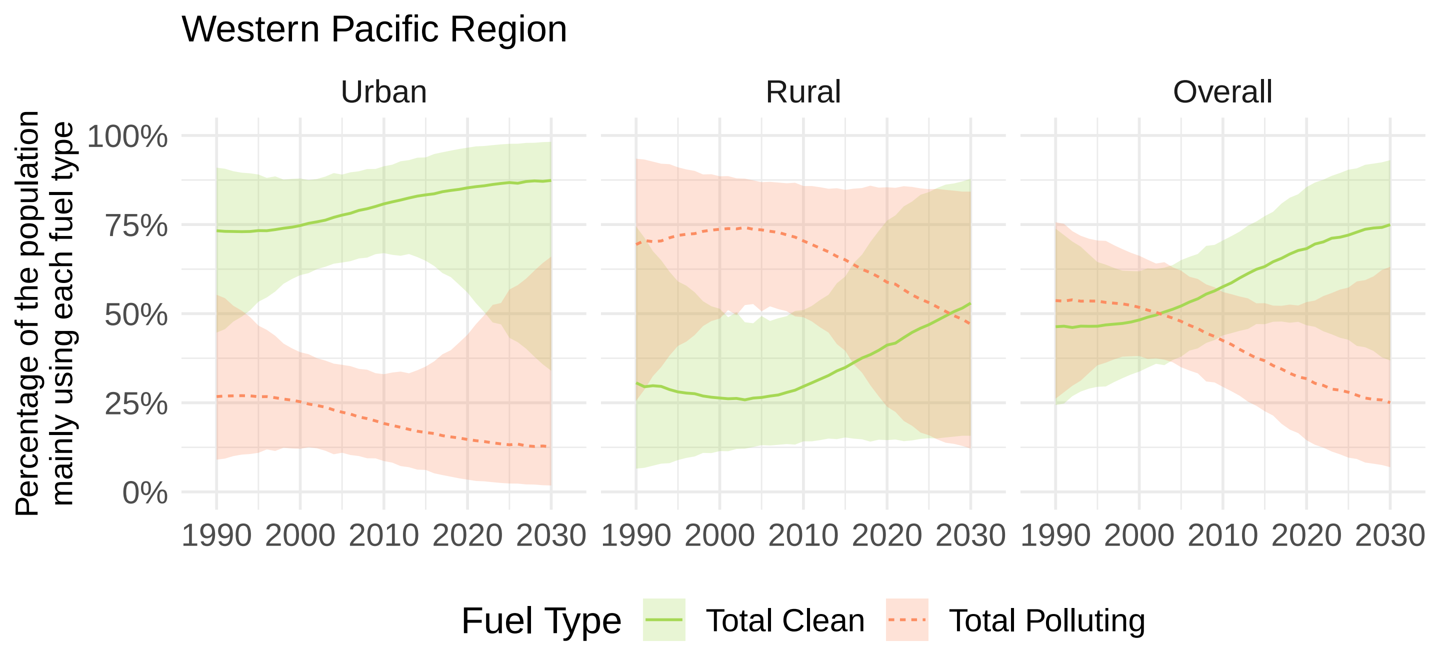
Western Pacific Region
Urban
Rural
Overall
Percentage of the population mainly using each fuel type
100%
75%
50%
25%
0%
1990
2000
2010
2020
2030
Fuel Type
Total Clean
Total Polluting
