# Supplementary material for: Household cooking fuel estimates at global and country level for 1990 to 2030
Source: Nat Commun. 2021 Oct 4;12:5793. doi: 10.1038/s41467-021-26036-x (PMC8490351; doi:10.1038/s41467-021-26036-x)
Supplement: Supplementary file 3 — Description of Additional Supplementary Files [file 41467_2021_26036_MOESM3_ESM.pdf]

Description of Additional Supplementary Files

File Name: Supplementary Data 1
Description: Country estimates (% and population) of clean, polluting, and specific fuel use, with urban and rural disaggregation, 1990-2030 (xlsx spreadsheet).

File Name: Supplementary Data 2
Description: Global and SDG regional estimates (% and population) of clean, polluting, and specific fuel use, with urban and rural disaggregation, 1990-2030 (xlsx spreadsheet).

File Name: Supplementary Data 3
Description: WHO regional estimates (% and population) of clean, polluting, and specific fuel use, with urban and rural disaggregation, 1990-2030 (xlsx spreadsheet).

File Name: Supplementary Software 1
Description: Custom R code to implement the Global Household Energy Model and reproduce our estimates/analyses.
