# Supplementary material for: Household cooking fuel estimates at global and country level for 1990 to 2030
Source: Nat Commun. 2021 Oct 4;12:5793. doi: 10.1038/s41467-021-26036-x (PMC8490351; doi:10.1038/s41467-021-26036-x)
Supplement: Supplementary file 7 — Supplementary Software 1 [file 41467_2021_26036_MOESM7_ESM.zip › Code/Instructions.docx]

All programs are executed from the main script (HAP_Master.R).

Please set your R working directory to the main folder (the one containing this document).
