# Supplementary material for: Household cooking fuel estimates at global and country level for 1990 to 2030
Source: Nat Commun. 2021 Oct 4;12:5793. doi: 10.1038/s41467-021-26036-x (PMC8490351; doi:10.1038/s41467-021-26036-x)
Supplement: Supplementary file 8 — Reporting Summary [file 41467_2021_26036_MOESM8_ESM.pdf]

nature research

Corresponding author(s): Oliver Stoner

Last updated by author(s): Jul 8, 2021

Reporting Summary

Nature Research wishes to improve the reproducibility of the work that we publish. This form provides structure for consistency and transparency in reporting. For further information on Nature Research policies, see our Editorial Policies and the Editorial Policy Checklist.

Statistics

For all statistical analyses, confirm that the following items are present in the figure legend, table legend, main text, or Methods section.

n/a	Confirmed	
☐	☒	The exact sample size (*n*) for each experimental group/condition, given as a discrete number and unit of measurement
☒	☐	A statement on whether measurements were taken from distinct samples or whether the same sample was measured repeatedly
☒	☐	The statistical test(s) used AND whether they are one- or two-sided<br>*Only common tests should be described solely by name; describe more complex techniques in the Methods section.*
☒	☐	A description of all covariates tested
☐	☒	A description of any assumptions or corrections, such as tests of normality and adjustment for multiple comparisons
☐	☒	A full description of the statistical parameters including central tendency (e.g. means) or other basic estimates (e.g. regression coefficient) AND variation (e.g. standard deviation) or associated estimates of uncertainty (e.g. confidence intervals)
☒	☐	For null hypothesis testing, the test statistic (e.g. *F*, *t*, *r*) with confidence intervals, effect sizes, degrees of freedom and *P* value noted<br>*Give P values as exact values whenever suitable.*
☐	☒	For Bayesian analysis, information on the choice of priors and Markov chain Monte Carlo settings
☒	☐	For hierarchical and complex designs, identification of the appropriate level for tests and full reporting of outcomes
☒	☐	Estimates of effect sizes (e.g. Cohen's *d*, Pearson's *r*), indicating how they were calculated

Our web collection on statistics for biologists contains articles on many of the points above.

Software and code

Policy information about availability of computer code

Data collection	Surveys in the WHO Household Energy Database were downloaded manually and collated using Microsoft Excel (versopn 16.50) and occasionally Stata/SE (version 15.1).
Data analysis	All data analysis was carried out using the R programming language. Custom R code (provided for download as supporting information) was developed to define and implement the WHO Global Household Energy Model and to process model outputs. The following additional R packages were loaded; abind (version 1.4-5); coda (0.19-3); doParallel (1.0.15); ggfan (0.1.3); ggplot2 (3.3.0); grid (4.0.0); gridExtra (2.3); mgcv (1.8-33); nimble (0.10.1); openxlsx (4.1.5); Rcolorbrewer (1.1-2); readxl (1.3.1); reshape2 (1.4.4); rgdal (1.5-16); scales (1.1.0); and tidyverse (1.3.0).

For manuscripts utilizing custom algorithms or software that are central to the research but not yet described in published literature, software must be made available to editors and reviewers. We strongly encourage code deposition in a community repository (e.g. GitHub). See the Nature Research guidelines for submitting code & software for further information.

Data

Policy information about availability of data

All manuscripts must include a data availability statement. This statement should provide the following information, where applicable:

- Accession codes, unique identifiers, or web links for publicly available datasets
- A list of figures that have associated raw data
- A description of any restrictions on data availability

The datasets generated during and/or analysed during the current study are available from the corresponding author on reasonable request.

Field-specific reporting

Please select the one below that is the best fit for your research. If you are not sure, read the appropriate sections before making your selection.

☐ Life sciences ☒ Behavioural & social sciences ☐ Ecological, evolutionary & environmental sciences

For a reference copy of the document with all sections, see nature.com/documents/nr-reporting-summary-flat.pdf

Behavioural & social sciences study design

All studies must disclose on these points even when the disclosure is negative.

Study description	The study is secondary data analysis of the WHO household energy database (early 2020 version), a collection of nationally-representative household surveys reporting the proportion of the population using different fuels and technologies as their main energy source for cooking. The data are quantitative values between 0 and 1, representing the reported proportion of the population mainly use each fuel type. The study aims to estimate use of different fuels for cooking at country, regional and global level for 1990-2030, using a Bayesian hierarchical model.<br><br>The following responses relate to how we selected and analyzed the data provided by the Household Energy Database, rather than the process by which surveys are conducted.
Research sample	The scope of the study is all low- and middle-income countries where suitable survey data are available (subject to exclusion criteria detailed below). For high-income countries, we make the assumption that 100% of the population mainly uses clean fuels for cooking when deriving regional and global estimates.<br><br>The main sources of household survey data include: National Living Standards or other national surveys; Demographic and Health Surveys (DHS); national censuses, Multi-indicator Cluster Surveys (MICS); World Health Surveys; Living Standard Measurement Surveys income expenditure surveys, or other national surveys supported by the World Bank; and Surveys on global AGEING (SAGE).
Sampling strategy	The version of the WHO household energy database used contains 1353 household surveys, with 1136 used in our study. The Bayesian hierarchical modelling approach is designed to appropriately quantify uncertainty associated with the number of survey data points in each country. If survey data are limited for a given country, or if data points are lacking for a given time window, the 95% prediction/uncertainty intervals provided alongside all point estimates as supporting information are correspondingly wider.
Data collection	The WHO household energy database is composed of statistics on cooking fuel use extracted from nationally representative surveys and censuses. If micro-data from the survey is available, it is processed using Stata/SE (version 15.1) to extract the proportion of households or population mainly relying on a given fuel to cook. If cooking fuel data is given in tabulated form, the data is manually input into the database. In some cases, cooking data extracted from surveys that are not nationally representative is included in the database, for example if they exclude disputed territories. Survey data included in the database must contain household or population level data.
Timing	The collection of new surveys was frozen (for the purposes of this analysis) in December 2019, with data cleaning continuing up to February 2020. Collection of surveys into the database started at least 20 years ago.
Data exclusions	We excluded surveys from before 1990, and only included data from surveys providing individual fuel breakdowns and with less than 15% of the population in total categorized as “missing”, “not cooking in the household” or “mainly cooking with ‘other’ fuels”. Surveys are also excluded if the fuel categories they report are not mutually-exclusive. These criteria were established in 2017-2018, fully prior to conducting the analysis presented here. There was no differentiation in the model between surveys that reported only household- or population-weighted fuel use estimates. Where surveys reported both household- and population-weighted estimates, only population-weighted estimates were used, in order to best estimate the population reliant on different cooking fuels. Only surveys for low- and middle-income countries were used in the analysis.
Non-participation	Many surveys report a small proportion of responses as "missing", "not cooking in the household" or "mainly cooking with 'other' fuels. Where applicable, reported values for the main fuel categories were divided by one minus the total of these categories.
Randomization	Randomization is not relevant to our study as we are not conducting any kind of experiment or measuring the effect of any interventions.

Reporting for specific materials, systems and methods

We require information from authors about some types of materials, experimental systems and methods used in many studies. Here, indicate whether each material, system or method listed is relevant to your study. If you are not sure if a list item applies to your research, read the appropriate section before selecting a response.

Materials & experimental systems

n/a	Involved in the study
☒	☐ Antibodies
☒	☐ Eukaryotic cell lines
☒	☐ Palaeontology and archaeology
☒	☐ Animals and other organisms
☒	☐ Human research participants
☒	☐ Clinical data
☒	☐ Dual use research of concern

Methods

n/a	Involved in the study
☒	☐ ChIP-seq
☒	☐ Flow cytometry
☒	☐ MRI-based neuroimaging
